# Molecular Dynamics Simulation of Water Confinement in Disordered Aluminosilicate Subnanopores

**DOI:** 10.1038/s41598-018-22015-3

**Published:** 2018-02-28

**Authors:** Takahiro Ohkubo, Stéphane Gin, Marie Collin, Yasuhiko Iwadate

**Affiliations:** 1 Graduate School of Engineering, Chiba University, 1-33 Yayoi-cho Inage-ku, Chiba, 263-8522 Japan; 2 CEA DEN DE2D SEVT, Laboratoire d’étude du Comportement à Long Terme, 30207 Bagnols-sur-Céze, France

## Abstract

The porous structure and mass transport characteristics of disordered silicate porous media were investigated via a geometry based analysis of water confined in the pores. Disordered silicate porous media were constructed to mimic the dissolution behavior of an alkali aluminoborosilicate glass, i.e., soluble Na and B were removed from the bulk glass, and then water molecules and Na were introduced into the pores to provide a complex porous structure filled with water. This modelling approach revealed large surface areas of disordered porous media. In addition, a number of isolated water molecules were observed in the pores, despite accessible porous connectivity. As the fraction of mobile water was approximately 1%, the main water dynamics corresponded to vibrational motion in a confined space. This significantly reduced water mobility was due to strong hydrogen-bonding water-surface interactions resulting from the large surface area. This original approach provides a method for predicting the porous structure and water transport characteristics of disordered silicate porous media.

## Introduction

Water confined in the nanopores of silicate compounds has been widely studied to understand the specific structural and dynamical properties of such compounds. Silicate minerals and glasses are known to include water, and hydrolysis reactions can occur on the surface^1–4^. However, predicting these reactions, which are important for mineral formation and carbon cycling related to CO_2_ in air, is extremely complicated^5,6^. In addition to natural minerals, confined water within artificial silicate materials, such as ordered silicates^7^ and synthetic clay minerals, is also attractive for chemical engineering applications^8^. In such confined spaces, water properties including its structure, melting point, and mobility differ significantly from those of bulk water^9–11^. Consequently, theoretical and experimental methods have been employed to investigate the structural and dynamical properties of water confined in well-organized nanopores of varying sizes, geometries, and surface hydrophilic/hydrophobic features^12–14^.

The properties of water in organized pores with one-dimensional tubes and two-dimensional nanosheets have been established using experimental and computational techniques. Various experimental techniques, including quasielastic neutron scattering (QENS)^15^, vibrational spectroscopy, and NMR have been used to investigate the properties of fluids in confined spaces^16–19^. The translational and rotational dynamics of water are highly sensitive to size and surface charges. Reduced diffusion and rotational motion, two typical confinement effects, have been observed by QENS and ^1^H NMR relaxation studies. Molecular dynamics (MD) simulations are also powerful in investigating the structure and dynamics of water in pores. MD simulations using cell sizes of 10 nm in length are now routinely used to gain insight into molecular behavior and mechanical properties. By constructing ideally shaped pores, the pore size and shape dependences of water properties have been systematically investigated and compared with experimental data^12,20^. Although these MD studies have highlighted the importance of nanopore geometry, an understanding of water in nanopores with disordered shapes is still required.

For silicate glass systems, when soluble elements are released into solution by contact with water, the remaining silicate network forms a complicated porous structure filled with water^21^. Molecular modeling of this structure is extremely difficult owing to its complexity. Surfaces with a high fractal dimension are formed by hydrolysis and condensation reactions at water interfaces^22^, and the resultant structures are hardly predictable. Therefore, the water properties in simplified ordered pores are not applicable to understanding the confinement effects in such systems. Confined water in disordered silicate-water systems has only been discussed in a few MD simulations^23–25^. Water in disordered calcium silicate hydrated gels shows slower diffusion than water in ordered pores^23^.

In this study, we modeled an aluminosilicate gel that might form by aqueous alternation of an alkali aluminoborosilicate glass. The glass, which has a composition of SiO_2_, B_2_O_3_, Al_2_O_3_, and Na_2_O, is a typical engineering material used for electronic device displays, windows, and the immobilization of nuclear waste^5^. Simplified compositions have been studied to better understand corrosion mechanisms^26–28^. The formation of altered layers on these glasses is attributed to the release of soluble elements, such as Na and B, into solution, followed by the repolymerization of the silicate network^29^. The random silicate networks of these glasses are necessary for their future performance and critical in designing their composition and manufacturing processes. For nuclear waste immobilization, the structure and transport properties of the altered layer on the glass surface is important for understanding the long-term performance when the glass is in contact with water^5^. Electron microscopy has revealed that nanoporous surface layers form during glass corrosion. When formed under silica-saturated conditions near neutral pH, these layers are passivating, and thus control the release of soluble elements from the glass^30,31^.

A recent glass corrosion study revealed that the pore size of international standard glass (ISG) in silica-saturated solution at pH 7 is ≤1 nm^32^. The apparent diffusion coefficients of water on the order of 10^−20^ m/s^2^ were obtained from time-dependent elemental profiles within the surface layers^32^. This value is significantly lower than that for nanometer-scale ordered pores^12–15^. The aim of this study was to investigate the regime between nanometer-scale ordered and disordered pores to provide insight into the porous structure and dynamics of water confined in disordered spaces.

## Computational Method

To construct a model of disordered aluminosilicate gels, four steps were used that followed realistic phenomena during borosilicate glass dissolution. First, a bulk glass model was made with the desired composition; second, soluble elements were removed from the bulk glass; third, Si was substituted with Al and water molecules and Na^+^ were introduced to match previously obtained experimental results^32^; finally, the system was equilibrated. Al substitution for Si in the third step permits comparison with available experimental data. Details are described later in this section. A well-organized three-body potential function was used for the first step. The second and third steps were continuously performed using the final structure from the first step. Finally, a MD simulation with ClayFF force fields was conducted using the structure obtained from the third step. All MD simulations were performed using LAMMPS code^33^. Detailed descriptions of each step are given below.

Bulk glass with an experimental density of 2.5 g/cm^3^ was constructed with a composition of 67.8SiO_2_-18.0B_2_O_3_-14.2Na_2_O and 3004 atoms (Si: 606, B: 322, Na: 254, O: 1822). This molar ratio and density match those of ISG^5^. The potential function for the MD simulations of the bulk glass used Born-Mayer-Huggins (BMH) potential parameters. An additional three-body potential was used for the O-Si-O, O-B-O, and Si-O-Si angles. The two- and three-potential parameters, taken from references^34,35^, were developed to reproduce first-neighbor distances and the B coordination number. Glass construction was performed by a melt-quenching process. The 3004 atoms were randomly distributed in the cell. The initial structure was melted at 3000 K for 100 ps, and then the temperature was decreased to 1773 K at 12.27 K/ps. After reaching energy equilibrium during a 100 ps run at 1773 K, a solid glass was prepared at 300 K by quenching the molten glass from 1773 to 300 K at 14.73 K/ps. All bulk glass simulations were performed in the NVT ensemble (constant number of particles, constant volume, and constant temperature)^36,37^. The final glass structure comprised tetrahedral Si with four coordination sites and three- and four-coordinated B.

In the second step, B and Na, as soluble elements in the glass, were removed to create a disordered silicate network. Free O atoms resulting from a lack of B were also removed, and nonbridging O atoms were terminated with H.

The third step involved the substitution of Si with Al and the introduction of water molecules into the molecular model obtained from the second step. The atomic composition was matched to the experimentally observed surface layer of ISG glass after dissolution for 209 days at 90 °C^32^. The altered layer contained Al. Therefore, 67Si was randomly replaced with Al to match the experimental data; the replacement of SiO_4_ tetrahedra bonded to substituted AlO_4_ tetrahedra was avoided according to the Al-O-Al avoidance rule suggested by Loewenstein^38^. H_2_O, H_3_O^+^, and Na were also randomly introduced into the void space, avoiding overlap with other atoms. This operation was performed with an atomic distance threshold of at least 2.0 Å to avoid unphysical interactions and structural breakage at the beginning of the simulations. The number of Na^+^ in the gels was 28, as determined from the concentration profile of Na in the altered layer^32^. A small amount of Na absorbed in the altered layer was also reported by solid-state NMR spectroscopy^39^.

We targeted three disordered silicate gels with different water contents (5.4, 18.1, and 31.3 wt%). Recently, the water molecule content of altered layers on ISG glass at pH 7 and 90 °C was estimated by thermal gravimetric analysis (total water content) and ^1^H NMR (water speciation). The determined value of 8 wt% falls within the range studied here^29^. Substitution of Si with Al caused the system to be negatively charged; therefore, the excess negative charge was compensated by Na^+^ and the introduction of H_3_O^+^ instead of H_2_O. The number of molecules and atoms in these systems are summarized in Table 1.

**Table 1:** Number of atoms and molecules in disordered gels with water contents of 5.4% (wc05), 18.1% (wc18), and 31.2% (wc31). The average box sizes are also listed.

	Si	Al	H_2_O	H_3_O^+^	Na	O	OH	Volume Å^3^
wc05	537	67	100	39	28	350	830	39079
wc18	529	67	500	39	28	328	846	47012
wc31	508	67	1050	39	28	296	898	63574

MD simulations were performed for the three models using the ClayFF force field^40^. The original ClayFF potential parameters cause a charge unbalance when hydroxyl oxygens are bonded to Al. Modified ClayFF potential parameters were developed by Kerisit *et al*.^41^; these predict contact between the silicate structure and water on the surface. Interatomic interactions were modeled with the SPC/E water model^42^. The potential model of Kusaka *et al*. used to model H_3_O^+^ was developed by fitting the potential parameters for the interaction of the hydronium ion with H_2_O to the experimental enthalpy of hydration for up to six water molecules^43^. The validity of this model was also confirmed by Kerisit *et al*.^41^. The geometries of H_2_O and H_3_O^+^ were treated as rigid using the SHAKE algorithm^44^. All potential parameters are listed in Tables S1–S4 in the Supplementary information.

All gel simulations were performed in the NPT ensemble (constant number of particles, constant pressure, and constant temperature) at 300 K and 1 atm^36,37,45^. The temperature was held constant by using the Nosé-Hoover thermostat^46^. The electrostatic interactions were calculated using the particle-particle-particle mesh method^47^ with a relative error of 0.0001 Kcal/mol/Å in the force and a real-space cutoff of 9 Å. The same cutoff was used for short-range interactions. For the initial structure, the atom trajectories were generated by the Verlet algorithm for 5000 steps with a time step of 0.001 fs. The time step was gradually increased to 0.001, 0.01, and 0.1 fs with each run of 5000 steps. A time step of 1 fs was finally employed for long simulation runs.

This simulation procedure produced unrealistic Si coordination numbers (CN = 2 and 3) owing to volume expansion in the initial simulation periods. We removed these unrealistic Si and Al units and the resultant free O, whereas the nonbridging O atoms generated by this treatment were terminated with H. After this treatment, the simulation was restarted from the new structure with a time step of 1 fs. This operation was repeated until unrealistic Si and Al coordination numbers were not formed during long simulations, that is, all Si and Al had four-coordinated tetrahedral structures and all nonbridging O atoms were hydroxyl oxygens. The final compositions of Si and O are listed in Table 1. After energy equilibrium was reached (~50 ns), a MD simulation for sampling atomic configurations was performed for 10^9^ steps (1 *μ*s). The sampled atomic configurations were analyzed to understand the structural and dynamical properties.

Static structures were also generated by decreasing the temperature to 1 K with a time step of 1 fs in the NVT ensemble (constant number of particles, constant volume, and constant temperature) after a 1 *μ*s simulation run under the average cell size of an NPT run. After 50000 steps at 1 K, the final atomic configurations were used for structural analysis of the pores based on the Voronoi network using Zeo++ ^48^.

For comparison, MD simulations of bulk water comprising 512 H_2_O molecules were performed using the same water potential parameters as for the aluminosilicate gel.

## Results and Discussion

### Structural properties

The structural properties of the three gels were investigated from the perspective of porous spaces and water structures. Here, the pores were defined as the volumes not occupied by framework atoms (Si, Al, and O). Pore analyses were performed on the static structures described in the Computational Methods section.

Voronoi-based surface reconstruction was used to define the boundary between pores and the volume occupied by the framework atoms, which was calculated from the finite atomic sizes of Si, Al, and O. Atomic sizes corresponding to the covalent radii were employed to define the atomic volumes. We used 1.16, 1.26, and 0.63 Å for Si, Al, and O, respectively^49^. Structural analyses of the pores were performed by determining the pore volumes, surface areas, and pore size distributions. Radial distribution functions and the cluster size distributions of water were estimated to understand the local structure and connectivity of water in confined spaces.

**Figure 1:**
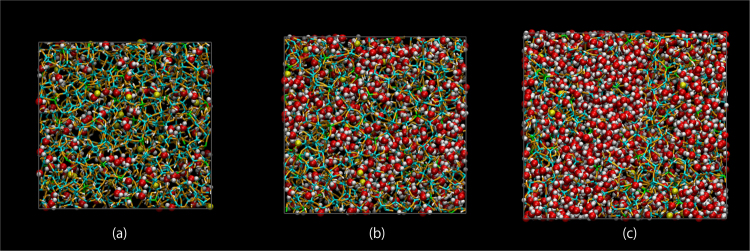
Atomic configurations of wc05 (**a**), wc18 (**b**), and wc31 (**c**). Cyan, lime, orange, and ivory bars correspond to Si, Al, O, and H, respectively, in the frameworks of the gels. Yellow, red, and white balls indicate Na, O, and H, respectively, of water and hydronium. Average side lengths of wc05, wc18, and wc31 are (34.537,33.169234.113), (36.389,35.2147,36.687), and (40.546,36.998,42.379) in Å, respectively. All angles of the unit cells are 90 degree.

Figure 1 shows a visualization of the atoms and bonds in wc05, wc18, and wc31 at the final step. Atomic configurations and cell parameters are also provided in the Supplementary information in CIF format. Protons on hydroxyl groups are oriented towards O on water or hydronium molecules in the pores, suggesting the formation of OH-water hydrogen bonds. Population of hydrogen bonded water with surface hydroxyl hydrogen or bridging oxygen was estimated for all water and hydronium. We used the H-O cutoff distance of 2.0 Å corresponding to hydrogen-bond formation. The time-averaged populations of hydrogen bonded water in wc05, wc18, and wc31 were 98.8, 92.7, and 74.1%, respectively. Most of water molecules in wc05 and wc18 have hydrogen bonding to atoms on the pore surface. The shapes of the water-filled pores are highly complex compared to those previously reported in MD studies of ordered silicate materials including water^12–14^. Vacant pores without water molecules and a few isolated water molecules were also identified in wc05. These water molecules were trapped in small spaces formed within the aluminosilicate network. Confined water molecules in disordered silicate channels were previously modeled from layered calcium silicate hydrate (CSH) by MD simulations at an unrealistically high temperature (1500 K)^23^. However, the degree of disorder in the modeled CSH structure is much lower than that in this study. For example, the layered channel structure of water-filled pores was retained, even when the silicate structure was destroyed by high-temperature annealing. In comparison, the building steps described in the computational method are suitable for modeling highly complex gels.

The Si and Al populations in bridging Q^*n*^ structures (*n* is the number of bridging O) and the densities of wc05, wc18, and wc31 are shown in Table 2. Different Q^*n*^ species with different water contents arise from the elimination of unrealistic Si units formed by volume expansion during the initial NPT MD runs. The eliminated numbers of Si were 69, 77, and 98 for wc05, wc18, and wc31, respectively, corresponding to 11, 13, and 16% of the 606 Si in the bulk glass. Thus, the extents of the disordered silicate network and the bulk glass structure were nearly equal. The density, which was calculated from the average volume during the simulation run, was decreased with increasing water content, indicating that volume expansion was caused by introducing excess water.

**Table 2:** Densities and populations (%) of Si- and Al-Q^*n*^ species in wc03, wc18, and wc31.

	Density (g/cm^3^)	Si					Al				
		Q^0^	Q^1^	Q^2^	Q^3^	Q^4^	Q^0^	Q^1^	Q^2^	Q^3^	Q^4^
wc05	1.993	2.2	11.5	27.4	38.9	19.9	0.0	10.4	35.8	35.8	17.9
wc18	1.898	2.3	11.5	28.9	38.8	18.5	1.5	14.9	35.8	32.8	14.9
wc31	1.640	2.6	14.6	31.1	37.0	14.8	1.5	19.4	43.3	28.4	7.5

Accessible and inaccessible pore volumes were evaluated by the Monte Carlo approach with a probe molecule using Zeo++^48^. In these analyses, a random position in the cell was chosen, and the position was checked to determine whether the probe molecule overlapped with the framework atoms. We used a probe molecule size of 1.41 Å, corresponding to the mean van der Waals radius of water molecules assigned from the Bjerrum four-point charge model for water^50^. If the probe molecule did not overlap the framework atoms, the space was considered a pore. This procedure was iterated until convergence; 100,000 iterations permitted estimation to three significant figures within 1% error. Furthermore, the identified pores were classified as accessible or inaccessible volumes based on whether the pores could connect with each other or were isolated, respectively. The pore connections were considered under periodic conditions, that is, connections between pores at edge positions in the simulation box and pores at the opposite side were estimated. The fraction of the accessible volume filled with water was estimated with the same procedure, where water was approximated as a sphere with a radius of 1.41 Å.

The surface area of the probe molecule is also a characteristic parameter that can be used to understand pore geometry, which is directly related to surface interactions between framework atoms and water molecules. In addition to the accessible and inaccessible pore volumes, the accessible and inaccessible surface areas were calculated using probe molecules with a size of 1.41 Å using Zeo++^48^.

The accessible and inaccessible pore volumes (AV and NAV) and accessible and inaccessible surface areas (SA and NSA) for wc05, wc18, and wc31 are listed in Table 3. The specific surface area (SSA) was calculated from the SA and the mass of framework atoms. The total accessible pore volume is increased with increasing water content because the unit cell expands in the presence of excess water molecules. The accessible volume of 70% in wc05 corresponds to vacant pores without water. The detected pore volumes of wc05, wc18, and wc31 mainly comprise accessible volume, indicating that small channels connect the pores. The porosity (%) was also estimated by dividing the pore volume by the total volume. Average pore diameters were calculated based on the accessible volume and the surface area by assuming cylindrically shaped pores with diameters proportional to the surface-to-volume ratio (4 × AV/SA).

**Table 3:** Total volume (V) and characteristic parameters of the space, excluding framework atoms in the cell.

	V (Å^3^)	AV (Å^3^)	NAV (Å^3^)	SA (Å^2^)	NSA (Å^2^)	P (%)	D (Å)	F (%)	SSA (m^2^/g)
wc05	39079	399	5.47	1240	89.2	10.2	1.29	70	1710
wc18	47012	1020	0.940	1830	17.3	21.8	2.24	100	2540
wc31	63574	2570	2.86	2260	25.9	40.5	4.56	100	3200

AV: accessible volume; NAV: ina ccessible volume; SA: accessible surface area; NSA: inaccessible surface area. Porosity (P) and diameter (D) were calculated as 100 × AV/V and 4 × AV/SA, respectively. Fraction of AV filled with water (F) was estimated from the structure with water present. Specific surface area (SSA) corresponds to SA per mass of framework atoms.

The obtained porosities and average pore diameters were compared with those of ordered porous materials, such as two-dimensional clay sheets and one-dimensional MCM-41. For a typical clay sheet, the thicknesses of a hydrated layer and the framework layer are 3.2 and 9.2 Å, respectively^51^. From this geometry, the porosity and pore diameter (4 × AV/SA) with two hydrated layers in the clay sheet are 59% and 12.8 Å, respectively, which are higher than those of the modeled structure in this study. Unlike hydrated clays, MCM-41 is a typical ordered material with one-dimensional pores that range in size from 20 to 100 Å^52^, one to two orders of magnitude larger than those in this study. According to the International Union of Pure and Applied Chemistry (IUPAC) system^53^, pores with sizes below 25 Å are classified as micropores. Unfortunately, few systematic studies have analyzed porous materials with pore sizes of approximately 10 Å. To our best knowledge, this study is the first to model highly complex porous materials to characterize structure and confined water.

**Figure 2:**
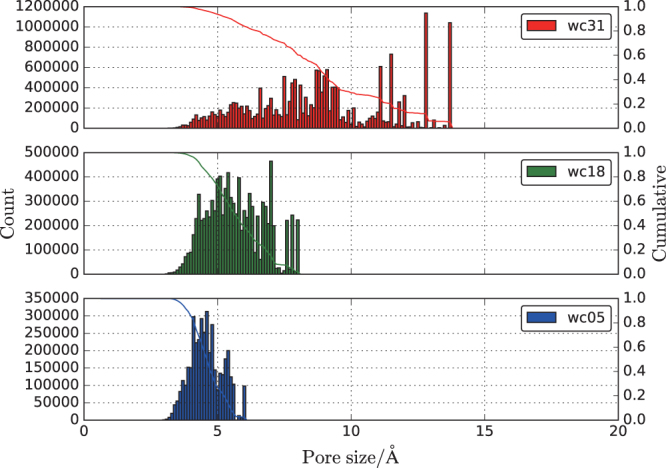
Pore size distribution of count on left axis and cumulation on right axis of wc05, wc18, and wc31. Pores are defined as the spaces not occupied by the gel framework (Si, Al, O, and OH).

Pore size distributions in the accessible volume were also calculated to understand the porous structure. Further, a position in the accessible volume was randomly chosen and analyzed to find the largest encapsulating sphere without framework atoms. The choice of the random position was iterated, the largest encapsulating sphere was stored, and any overlap with other encapsulating spheres was detected. We confirmed that 50000 and 100000 iterations yielded the same pore size distribution profiles. The pore size distributions and cumulation in wc05, wc18, and wc31 are displayed in Fig. 2. A Gaussian-like distribution centered at a pore size of 4 Å is identified in wc05. Higher counts on the larger pore size side are observed at around 8 and 13 Å in wc18 and wc31, respectively. This trend suggests that water molecules aggregate to form water channels that ease transport. To directly estimate water aggregation, the cluster size distribution is calculated.

**Figure 3:**
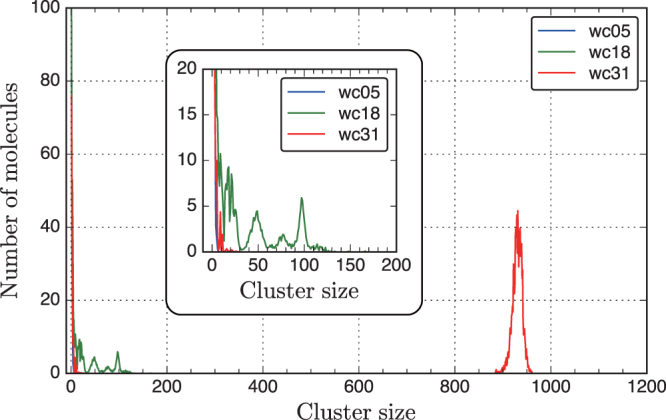
Cluster size distributions of water in wc05, wc18, and wc31 with a cutoff distance of 3.05 Å. The vertical axis shows the number of molecules with a given cluster size. The inset shows the distribution of small cluster sizes ranging from 0 to 200.

**Figure 4:**
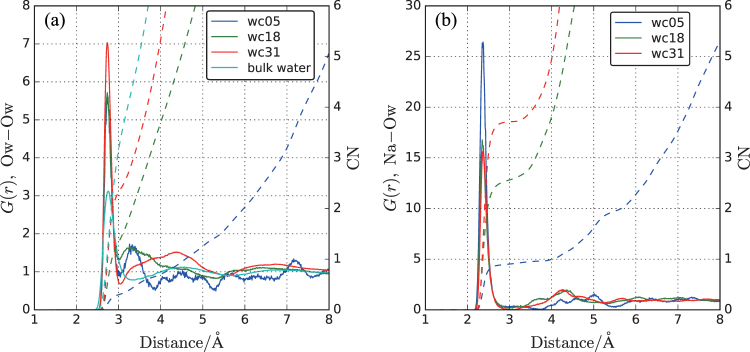
Radial distribution functions *G*(*r*) (solid lines, left axis) and running coordination number, CN (dashed lines, right axis) of wc05, wc18, and wc31. (**a**) The Ow (H_2_O)–Ow (H_2_O); (**b**) Na–Ow (H_2_O). The Ow (H_2_O)–Ow (H2O) *G*(*r*) of bulk water is also shown in the left figure for comparison.

To characterize the connectivity of water-filled pores, we calculated the cluster size distribution of water (Fig. 3), where a cluster was defined as the number of connected water molecules under periodic conditions. For example, if three water molecules were connected within the cutoff distance, the cluster size was 3. The cluster size distribution is indicated by a histogram of the number of water molecules; that is, for four clusters with cluster size 3, the height of the cluster size 3 is 12 in the histogram. We determined the cutoff distance of 3.05 Å from the O-O distance of the first peak in the H_2_O-H_2_O radial distribution functions (Fig. 4), which roughly includes all water molecules in the first hydration shell. The time-averaged cluster size was obtained from all configurations during the simulation period.

All cluster sizes observed for wc05 were less than 10. Several peaks corresponding to cluster sizes of 0–150 were observed for wc18. These peaks with higher cluster sizes are consistent with the pore size distributions shown in Fig. 2. Many water molecules belong to small cluster sizes of less than 10. By combining the information in Figs 2 and 3, we can describe the morphology of the water network in hydrated gels. At low water content (wc05), there are many small isolated water clusters and vacant pores. At higher water contents (wc18 and wc31), a few large water clusters govern the water distribution. However, the cluster size distribution is significantly inhomogeneous.

The local structure of confined water in the gel is investigated using the radial distribution functions, *G*(*r*), between water O atoms (Fig. 4(a)). For comparison, the corresponding *G*(*r*) of bulk water obtained from the SPC/E water model^42^ is also displayed. The O-O distances of the first peak in wc05, wc18, and wc31 (2.73–2.74 Å) are independent of water content. The first minimum at 3.05 Å is used as the cutoff for estimating the cluster size distribution in Fig. 3. The position of the first peak of bulk water is 2.76 Å, similar to that calculated from another potential model of bulk water^48^. Although the O-O distance of confined water is slightly shorter than that of bulk water, the confinement effect on the water-water distance is not significant. However, clear second peaks are observed at approximately 3.3 Å in wc05 and wc18, and 4.4 Å in wc31, which are not confirmed in bulk water. The position of the second peak in wc31 is almost identical to that of bulk water, indicating that the aggregated structure of water in relatively large clusters is similar to that of bulk water. The shorter distance of the second peaks in wc05 and wc18 suggests closely packed water clusters in the pores. As the water content increases, this second peak is weakened and almost disappears in wc31. The peak positions are consistent with those for water in Nafion membranes with 5% water content, which are solid polymer materials showing high water diffusivity^54–56^.

The coordination number of water molecules attached to other water molecules is increased with increasing water content, approaching that of bulk water. The coordination number of 4 observed in bulk water at 3.22 Å is not allowed in wc05 and wc18. These small coordination numbers are influenced by geometrical effects caused by the large surface areas and small volumes in confined pores. The coordination structure of water around Na is depicted in Fig. 4(b). The Na^+^-H_2_O O distances and coordination numbers in Na aqueous solution, determined experimentally and by MD simulation, are 2.29–2.40 Å and 4.3–6.2, respectively^57–60^. The coordination number of water around Na^+^ calculated for the first coordination sphere at 3 Å increases with increasing water content (0.903, 2.56, and 3.71). In contrast, the Na^+^-H_2_O O distance is not influenced by water content (2.35–2.37 Å) and agrees with the value in bulk Na aqueous solution. Structural changes of Na^+^ solvation in a confined space affect the coordination number rather than the coordination distance.

### Dynamical properties of water

The dynamical properties of water in the gels were evaluated through the square displacements and van Hove functions of water molecules.

**Figure 5:**
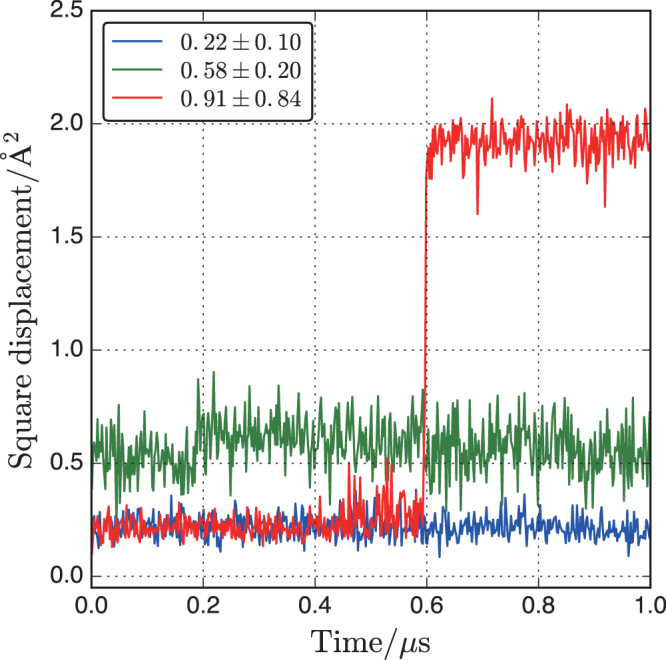
Typical square displacement behavior of three water molecules in wc18. The values in the legend indicate the averages and standard deviations.

**Figure 6:**
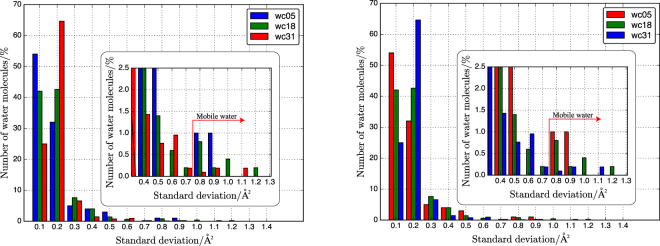
Histogram of standard deviations for square displacement of water molecules in wc05, wc18, and wc31. The inset shows expanded data ranging from 0.3 to 1.3 Å^2^.

The square displacement (SD) of an individual water molecule is defined as

1 $${{\rm{SD}}}_{i}(t)=|{r}_{i}(t)-{r}_{i}{\mathrm{(0)|}}^{2}$$

where *r*_*i*_(*t*) is the position of each O atom *i* in water at time *t*. The examples of three water molecules in wc18 are shown in Fig. 5. Water corresponding to the blue and green lines is relatively less displaced and can be considered “immobile water”. In comparison, water corresponding to the red line experiences hopping at 0.6 *μ*s, and can be considered “mobile water”. The distinction between mobile and immobile water molecules is based on the histogram of the standard deviation of the SD, as shown in Fig. 6. Most water molecules have deviations of 0.1 and 0.2, independent of the water content, mainly because water molecules move freely in the confined spaces formed by neighboring water molecules and the surface of the aluminosilicate network. A small gap is observed at a standard deviation of 0.8 Å^2^ in the inset of Fig. 6. As shown in Fig. 5, standard deviations above 0.8 Å^2^ correspond to hopping water molecules in the simulation period (1 *μ*s). Therefore, we used a standard deviation of 0.8 Å^2^ to distinguish mobile and immobile water. The fractions of mobile water are 1.0, 0.8, and 0.4% for wc05, wc18, and wc31, respectively. The higher fraction of mobile water in wc05 suggests that water can hop into a vacant pore via a stochastic process. However, these mobile water molecules are not a major component.

**Figure 7:**
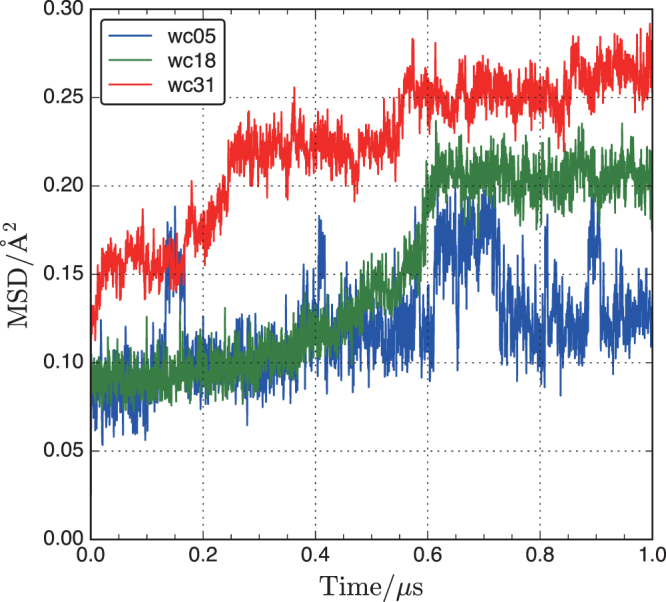
Mean square displacement of water in wc05, wc18, and wc31.

The mean square displacement (MSD) of H_2_O in wc05, wc18, and wc31 is calculated from the SD averaged over the total number of water molecules (Fig. 7):

2 $${\rm{MSD}}(t)=\frac{1}{N}\sum _{i=1}^{N}{|{r}_{i}(t)-{r}_{i}\mathrm{(0)}|}^{2}.$$

A nonlinear increase of MSD is observed for wc18. However, the MSD of water in wc05 frequently shows singular jumps with an interval of approximately 0.2 *μ*s. This behavior suggests that there is frequent cooperation between hopping water molecules. For wc18, water molecules only move ballistically up to 0.6 *μ*s, ∝ *t*^2^. Similar behavior has been reported for water in calcium-silicate-hydrate and on amorphous silica surfaces^24,61^. Step-like increases of water are also confirmed in wc31, but the plateau and slope period is approximately 0.2 *μ*s, which is shorter than that in wc18. Thus, the behavior of the MSD is close to the Einstein relation (∝ *t*) observed in liquids.

To improve the MSD statistics, time averaging of <MSD> during *t* = 500 ns at multiple time origins (*r*_*i*_(0)) with 5 ns time shifts was performed for wc31. Self-diffusion coefficients can immediately be calculated from the Einstein relation, *D* = MSD/6*t*, for a linear region at long times (250 ns period). Unfortunately, the <MSD> of water in wc05 and wc18 were not linear functions of time, indicating that the simulation period was insufficient to estimate *D*. The *D* value for water in wc31 is 8 × 10^−16^ m/s^2^, which is extremely small compared to the reported *D* values for water in ordered silicate materials such as clay sheets and MCM-41^12,20^. Tortuous diffusion paths arising from the disordered porous structure rather than the straight diffusion paths in ordered silicate materials affect the *D* value considerably.

MD simulations of silica nanopores with various pore sizes revealed that the confinement effect of *D* for water gradually decreases for pore sizes less than 5 Å^12,13^. This trend is inconsistent with the result of this study; that is, the translation of water is dramatically limited regardless of the pore size. Possible reasons for this difference include the geometry of the diffusion path and the large surface area arising from the complex pore shapes. Interactions between OH on the pore surface and water effectively suppress water displacement and form hydrogen bonds between water and OH. Complex pore shapes with large surface areas thus allow the development of more hydrogen bonds than ordered pore shapes. Although the space is classified as accessible (Table 3), large surface areas for forming hydrogen bonds limit the accessibility of any space. The rotational correlation function of water, *C*(*t*), which is an index of hydrogen bonds, is defined as follows:

3 $$C(t)=\frac{1}{2N}\sum _{i}^{N}(\mathrm{3[}{\cos }^{2}\theta (\tau )-1)=\frac{1}{2N}\sum _{i}^{N}(\mathrm{3[}{d}_{i}(\tau )\cdot {d}_{i}{\mathrm{(0)]}}^{2}-1)$$

where *d*_*i*_(*τ*) is the dipole axis vector of water molecule *i*. We calculated *C*(*t*) for water molecules during the simulation run. *C*(*t*) for wc05, wc18, and wc31 are shown in Fig. S1 in the Supplementary information. The rotational dynamics of water molecules were clearly restricted by the developed hydrogen bonds.

Experimentally, *D* for water was observed from the elemental profile of H in an altered layer on a glass surface after glass dissolution in water^32^. The *D* value of 8 × 10^−20^ m/s^2^ was four orders of magnitude less than the diffusivity in wc31. It is worth noting that water diffusivity is strongly influenced by the water content; i.e., the water content is probably lower than that in wc31^32^, which causes strong interactions with the solid surface because of the small cluster size. Thus, water molecules are confined in a sub-nanometer space with ultra-low diffusivity.

**Figure 8:**
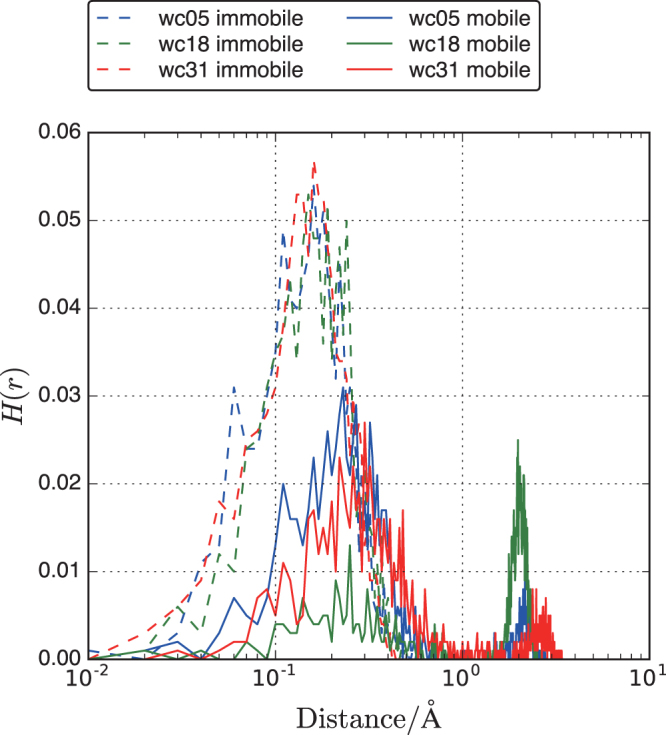
Van Hove function at *t* = 500 ns for immobile (dashed) and mobile (solid) water in wc05, wc18, and wc31.

The van Hove correlation function (*H*(*r*, *t*)), which is a better expression for understanding water hopping in pores, is defined as follows:

4 $$H(r,t)=\frac{1}{N}\sum _{i}^{N} < \delta (r-|{r}_{i}(t+\tau )-{r}_{i}(\tau )| > $$

where *r*_*i*_ is the coordinates of oxygen atom *i* in water, *δ* denotes the Kronecker function, and the brackets represent time averaging at multiple time origins, *τ*. We calculated *H*(*r*, *t*) for water molecules at *t* = 500 ns. *H*(*r*, *t*) for mobile and immobile water in wc05, wc18, and wc31 are shown in Fig. 8. The peak centered at 0.2 Å in *H*(*r*, *t*) for immobile water in wc05, wc18, and wc31 corresponds to vibrational motion in a confined space. However, mobile water mainly shows two peaks at approximately 0.3 and 2 Å. The latter distance implies escape from a cage-like confined space, which corresponds to the apparent pore size for water diffusion. This distance cannot be clearly differentiated from the pore size distribution shown in Fig. 2. Therefore, the diffusion energy barrier for transition from the immobile to the mobile state is governed by surface interactions rather than pore geometry. This concept can be used to understand ultra-confined water in disordered pores.

## Conclusions

Various gels formed by glass dissolution were carefully studied using the MD method for long periods of 1 *μ*s. The molecular structures of aluminosilicate porous gels including water were modeled from borosilicate glass by removing soluble elements, mimicking the chemical processes of glass alteration following contact with water. The aluminosilicate porous network comprised four-coordinated Si and Al tetrahedra with nonbridging O terminated with H. Three water contents (5, 13, and 31%) were considered to understand the structures and dynamics of confined water.

Structural analyses of porous structures were carried out by examining accessible and inaccessible volumes and surface areas using a probe molecule (1.41 Å diameter). The pores were classified as accessible, with sub-nanometer sizes and extremely large surface areas compared to those of ordered silicate materials. Local structures of water and Na^+^ revealed unusually low coordination numbers for confined water. Cluster sizes, corresponding to the connectivity of the water channels, were dramatically increased with increasing water content. Indeed, water channels were identified in snapshots of the materials with 31% water content.

The mobility of water molecules was extremely limited, despite the formation of water clusters. Mobile and immobile water molecules were identified based on the square displacement of water for 1 *μ*s. The amount of water experiencing hopping events was less than 1%, regardless of the water content. The mean hopping distance of water molecules was around 2 Å, as determined from the van Hove function. Importantly, this hopping distance was independent of the water content. Although the pores were classified as accessible and water formed clusters as transport channels, strong interactions between water and neighboring atoms suppressed water mobility. This feature is characteristic of disordered aluminosilicate gels with large surface areas.

Overall, this work targeted the equilibrium state of water in ultra-confining disordered aluminosilicate networks. As proton association-dissociation of the surface hydroxyl groups is an important reaction for the formation of aluminosilicate networks, we will focus on the kinetics of this process in our future work.

## Electronic supplementary material

Supplementary Information
