# Supplementary material for: Molecular Dynamics Simulation of Water Confinement in Disordered Aluminosilicate Subnanopores
Source: Sci Rep. 2018 Feb 28;8:3761. doi: 10.1038/s41598-018-22015-3 (PMC5830603; doi:10.1038/s41598-018-22015-3)
Supplement: Supplementary file 1 — Supplementary Information [file 41598_2018_22015_MOESM1_ESM.pdf]

Supplementary information for: Molecular Dynamics Simulation of Water Confinement in Disordered Aluminosilicate Subnanopores

Takahiro Ohkubo,[*,†] Stéphane Gin,[‡] Marie Collin,[‡] and Yasuhiko Iwadate[†]

†*Graduate School of Engineering, Chiba University, 1-33 Yayoi-cho Inage-ku, Chiba 263-8522, Japan*

‡*CEA DEN DE2D SEVT, Laboratoire d'étude du Comportement à Long Terme, 30207 Bagnols-sur-Céze, France*

E-mail: ohkubo.takahiro@faculty.chiba-u.jp

Phone: +81 (0)43 2903435. Fax: +81 (0)43 2903435

Potential function and parameters for MD simulation

The Lennard-Jones function includes the short-range and Coulombic energy,

$$E_{LJ} = \sum_{i \neq j} \left[D_{0,ij} \left\{ \left(\frac{R_{0,ij}}{r_{ij}} \right)^{12} - 2 \left(\frac{R_{0,ij}}{r_{ij}} \right)^{6} \right\} + \frac{q_i q_j}{4\pi\epsilon_0 r} \right]. \tag{1}$$

Interaction parameters, $R_{0,ji}$ and $D_{0,ji}$, are derived from parameters of each atom shown in Table S1 using mixing,

$$D_{0,ij} = \sqrt{D_{0,i} \cdot D_{0,j}} \tag{2}$$

$$R_{0,ij} = \frac{1}{2} \left(R_{0,i} + R_{0,j} \right) \tag{3}$$

The bond stretch energy of the hydroxyl bond is described by a simple harmonic term using parameters shown in Table S2,

$$E_{\text{bond}} = k_1 \left(r_{ij} - r_0 \right) \tag{4}$$

The angle energy of the hydroxyl group is also described by a simple harmonic term using parameters shown in Table S3,

$$E_{\text{angle}} = k_2 \left(\theta_{ijk} - \theta_0 \right) \tag{5}$$

Table S1: Symbols, charges, and Lennard-Jones potential parameters.

Species	Symbol	Mass (g/mol)	Charge (e)	D_0 (kcal/mol)	R_0 (Å)
Silicon	st	28.0855	2.1000	1.8405E-06	3.7064
Aluminum	at	26.981539	1.5750	1.8405E-06	3.7064
Bridging oxygen	ob	15.9994	-1.0500	0.155400	3.5532
Bridging oxygen with substitution	obts	15.9994	-1.1688	0.155400	3.5532
Hydroxyl oxygen	oh	15.9994	-0.9500	0.155400	3.5532
Hydroxyl oxygen with substitution	ohs	15.9994	-1.0688	0.155400	3.5532
Hydroxyl hydrongen	ho	1.00794	0.4250	0.000000	0.0000
Water oxygen	ow	15.9994	-0.8476	0.155400	3.5532
Water hydrongen	hw	1.00794	0.4238	0.000000	0.0000
Hydronium oxygen	op	15.9994	-0.2480	0.274058	3.2562
Hydronium hydrogen	hp	1.00794	0.4160	0.000000	0.0000
Sodium	Na	22.989768	1.0000	0.130100	2.6378

Table S2: Bond parameters for hydroxyl group.

Ion pair, ij	$k_{1,ij}$ (kcal/mol/Å^2)	r_0 (Å)
oh-ho	554.1349	1.0000
ohs-ho	554.1349	1.0000

Table S3: Angle parameters for hydroxyl group.

Species, ijk	$k_{2,ijk}$ (kcal/mol/rad^2)	θ_0 (deg)
st-oh-ho	30	109.47
at-ohs-ho	30	109.47

Table S4: Water and hydronium geometries.

	Water	Hydronium
$r_{\mathrm{O-H}}$ (Å)	1.000	0.973
H-O-H (deg)	109.47	111.60

Atomic configrations and cell parameters

The atomic configration and the cell parameter of wc05, wc18, wc31 at the last timestep were provided with CIF format (Crystallographic Information File) following the file names.

- wc05_laststep.cif (wc05)
- wc18_laststep.cif (wc18)
- wc31_laststep.cif (wc31)

The rotational correlation function of water

The rotational correlation function of water, $C(t)$ was was defined as follows:

$$C(t) = \frac{1}{2N}\sum_{i}^{N}\left(3[\cos^2\theta(\tau) - 1\right) = \frac{1}{2N}\sum_{i}^{N}\left(3[d_i(\tau)\cdot d_i(0)]^2 - 1\right) \tag{6}$$

where $d_i(\tau)$ is the dipole axis vector of water molecule i. We calculated $C(t)$ for water molecules during simulatin run.

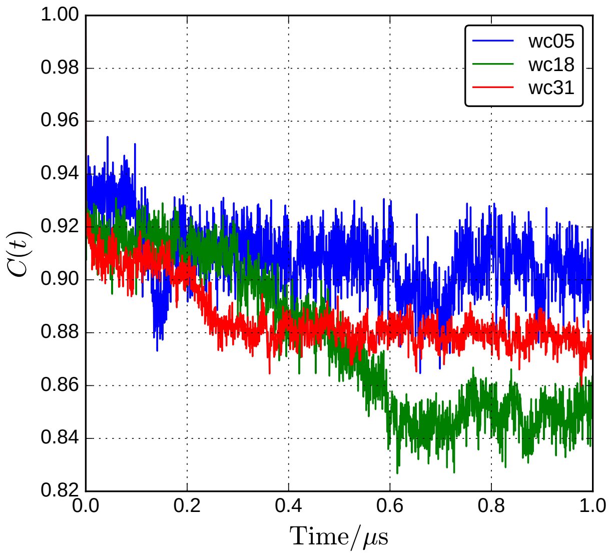

Figure S1: Rotational correlation functions of water in wc05, wc18, and wc31.

```
#======================================================================

# CRYSTAL DATA

#----------------------------------------------------------------------

data_VESTA_phase_1


_chemical_name_common                  'wc05'
_cell_length_a                        34.537130
_cell_length_b                        33.169223
_cell_length_c                        34.112742
_cell_angle_alpha                     90
_cell_angle_beta                      90
_cell_angle_gamma                     90
_space_group_name_H-M_alt             'P 1'
_space_group_IT_number                1

loop_
_space_group_symop_operation_xyz
   'x, y, z'

loop_
   _atom_site_label
   _atom_site_occupancy
   _atom_site_fract_x
   _atom_site_fract_y
   _atom_site_fract_z
   _atom_site_adp_type
   _atom_site_B_iso_or_equiv
   _atom_site_type_symbol
   Si1        1.0     0.108615      0.297780      0.607102     Biso  1.000000 Si
   Al1        1.0     0.370700      0.264532      0.785457     Biso  1.000000 Al
   Si2        1.0     0.391521      0.405840      0.959852     Biso  1.000000 Si
   Al2        1.0     0.140961      0.949676      0.707759     Biso  1.000000 Al
   Al3        1.0     0.818374      0.859438      0.279768     Biso  1.000000 Al
   Si3        1.0     0.195206      0.541586      0.525595     Biso  1.000000 Si
   Si4        1.0     0.389807      0.482239      0.757262     Biso  1.000000 Si
   Si5        1.0     0.371258      0.060406      0.193058     Biso  1.000000 Si
   Si6        1.0     0.171053      0.231583      0.610268     Biso  1.000000 Si
   Al4        1.0     0.323267      0.531019      0.728971     Biso  1.000000 Al
   Si7        1.0     0.066655      0.953185      0.220380     Biso  1.000000 Si
   Si8        1.0     0.122524      0.121330      0.951067     Biso  1.000000 Si
   Si9        1.0     0.137278      0.438623      0.823015     Biso  1.000000 Si
  Si10        1.0     0.947013      0.689540      0.224412     Biso  1.000000 Si
  Si11        1.0     0.064509      0.260765      0.259993     Biso  1.000000 Si
  Si12        1.0     0.166335      0.952618      0.912084     Biso  1.000000 Si
   Al5        1.0     0.091597      0.650323      0.920451     Biso  1.000000 Al
  Si13        1.0     0.804430      0.407779      0.415853     Biso  1.000000 Si
  Si14        1.0     0.877406      0.370805      0.101432     Biso  1.000000 Si
  Si15        1.0     0.756673      0.662678      0.515995     Biso  1.000000 Si
  Si16        1.0     0.574130      0.159578      0.251844     Biso  1.000000 Si
  Si17        1.0     0.302237      0.172615      0.229105     Biso  1.000000 Si
  Si18        1.0     0.279084      0.950881      0.723222     Biso  1.000000 Si
  Si19        1.0     0.227779      0.957143      0.845159     Biso  1.000000 Si
  Si20        1.0     0.927755      0.930984      0.413945     Biso  1.000000 Si
  Si21        1.0     0.543285      0.628583      0.043414     Biso  1.000000 Si
  Si22        1.0     0.460840      0.642544      0.430939     Biso  1.000000 Si
  Si23        1.0     0.943558      0.132714      0.674528     Biso  1.000000 Si
  Si24        1.0     0.268770      0.654185      0.842908     Biso  1.000000 Si
  Si25        1.0     0.468507      0.597361      0.002568     Biso  1.000000 Si
   Al6        1.0     0.419815      0.436127      0.036707     Biso  1.000000 Al
  Si26        1.0     0.408013      0.577198      0.629504     Biso  1.000000 Si
  Si27        1.0     0.889304      0.125002      0.501229     Biso  1.000000 Si
  Si28        1.0     0.864661      0.700529      0.116459     Biso  1.000000 Si
  Si29        1.0     0.322606      0.621507      0.415191     Biso  1.000000 Si
  Si30        1.0     0.234696      0.368504      0.579191     Biso  1.000000 Si
   Al7        1.0     0.154179      0.332139      0.463536     Biso  1.000000 Al
```

Si31	1.0	0.364434	0.087878	0.538969	Biso	1.000000	Si
Si32	1.0	0.237721	0.776575	0.289643	Biso	1.000000	Si
Si33	1.0	0.443407	0.948216	0.026265	Biso	1.000000	Si
Si34	1.0	0.970396	0.958241	0.814335	Biso	1.000000	Si
Si35	1.0	0.951489	0.462420	0.280395	Biso	1.000000	Si
Si36	1.0	0.987286	0.867123	0.810677	Biso	1.000000	Si
Al8	1.0	0.077024	0.094736	0.023918	Biso	1.000000	Al
Si37	1.0	0.569868	0.388677	0.038344	Biso	1.000000	Si
Al9	1.0	0.271830	0.284715	0.266710	Biso	1.000000	Al
Al10	1.0	0.598811	0.405388	0.880246	Biso	1.000000	Al
Si38	1.0	0.437795	0.919003	0.559228	Biso	1.000000	Si
Al11	1.0	0.822914	0.124429	0.211804	Biso	1.000000	Al
Si39	1.0	0.691786	0.447342	0.163206	Biso	1.000000	Si
Si40	1.0	0.677566	0.614531	0.069996	Biso	1.000000	Si
Si41	1.0	0.588294	0.647365	0.320575	Biso	1.000000	Si
Si42	1.0	0.077076	0.353888	0.673367	Biso	1.000000	Si
Si43	1.0	0.485061	0.556136	0.452983	Biso	1.000000	Si
Si44	1.0	0.783070	0.045653	0.596381	Biso	1.000000	Si
Si45	1.0	0.369307	0.960161	0.518542	Biso	1.000000	Si
Si46	1.0	0.204939	0.016552	0.954558	Biso	1.000000	Si
Si47	1.0	0.570858	0.932759	0.300339	Biso	1.000000	Si
Si48	1.0	0.618045	0.886361	0.046437	Biso	1.000000	Si
Al12	1.0	0.675797	0.050722	0.783089	Biso	1.000000	Al
Si49	1.0	0.775696	0.578542	0.479624	Biso	1.000000	Si
Si50	1.0	0.253027	0.030066	0.471434	Biso	1.000000	Si
Si51	1.0	0.163631	0.597011	0.277377	Biso	1.000000	Si
Si52	1.0	0.730382	0.785005	0.309260	Biso	1.000000	Si
Si53	1.0	0.154109	0.398348	0.689678	Biso	1.000000	Si
Si54	1.0	0.268134	0.122890	0.015038	Biso	1.000000	Si
Si55	1.0	0.169483	0.574370	0.997319	Biso	1.000000	Si
Si56	1.0	0.893664	0.997205	0.531432	Biso	1.000000	Si
Si57	1.0	0.186576	0.747600	0.743297	Biso	1.000000	Si
Si58	1.0	0.716994	0.864657	0.144445	Biso	1.000000	Si
Si59	1.0	0.274740	0.558659	0.106143	Biso	1.000000	Si
Si60	1.0	0.904450	0.781824	0.606354	Biso	1.000000	Si
Al13	1.0	0.023212	0.813971	0.390186	Biso	1.000000	Al
Si61	1.0	0.812303	0.306962	0.793445	Biso	1.000000	Si
Si62	1.0	0.870547	0.318042	0.239850	Biso	1.000000	Si
Si63	1.0	0.672219	0.254457	0.525346	Biso	1.000000	Si
Si64	1.0	0.229228	0.116171	0.342201	Biso	1.000000	Si
Si65	1.0	0.474431	0.063386	0.332031	Biso	1.000000	Si
Si66	1.0	0.321660	0.366813	0.692255	Biso	1.000000	Si
Si67	1.0	0.214639	0.683139	0.510493	Biso	1.000000	Si
Si68	1.0	0.987124	0.554559	0.797198	Biso	1.000000	Si
Si69	1.0	0.714281	0.920857	0.608180	Biso	1.000000	Si
Si70	1.0	0.413685	0.857804	0.152404	Biso	1.000000	Si
Si71	1.0	0.509521	0.870310	0.269810	Biso	1.000000	Si
Si72	1.0	0.521004	0.981428	0.644947	Biso	1.000000	Si
Si73	1.0	0.294086	0.404471	0.371542	Biso	1.000000	Si
Si74	1.0	0.707195	0.998579	0.851084	Biso	1.000000	Si
Si75	1.0	0.158586	0.693556	0.884019	Biso	1.000000	Si
Si76	1.0	0.387496	0.456471	0.883904	Biso	1.000000	Si
Si77	1.0	0.026817	0.176894	0.275942	Biso	1.000000	Si
Si78	1.0	0.781799	0.699664	0.593752	Biso	1.000000	Si
Si79	1.0	0.090244	0.219778	0.843758	Biso	1.000000	Si
Al14	1.0	0.480138	0.181548	0.974867	Biso	1.000000	Al
Si80	1.0	0.657576	0.265143	0.391458	Biso	1.000000	Si
Si81	1.0	0.645204	0.658424	0.250720	Biso	1.000000	Si
Si82	1.0	0.825315	0.009646	0.470751	Biso	1.000000	Si
Si83	1.0	0.591022	0.422099	0.665347	Biso	1.000000	Si
Si84	1.0	0.394103	0.194456	0.971851	Biso	1.000000	Si
Si85	1.0	0.847381	0.908692	0.656655	Biso	1.000000	Si
Si86	1.0	0.043810	0.550556	0.010541	Biso	1.000000	Si
Si87	1.0	0.202311	0.164007	0.965522	Biso	1.000000	Si
Si88	1.0	0.164888	0.864157	0.943099	Biso	1.000000	Si
Si89	1.0	0.213561	0.610846	0.709213	Biso	1.000000	Si
Si90	1.0	0.313104	0.498854	0.859849	Biso	1.000000	Si
Si91	1.0	0.560264	0.468000	0.343235	Biso	1.000000	Si
Si92	1.0	0.347406	0.896292	0.396998	Biso	1.000000	Si
Si93	1.0	0.801810	0.022583	0.990613	Biso	1.000000	Si
Al15	1.0	0.002061	0.831846	0.219806	Biso	1.000000	Al
Al16	1.0	0.268130	0.131529	0.633664	Biso	1.000000	Al
Si94	1.0	0.878657	0.787513	0.388427	Biso	1.000000	Si
Si95	1.0	0.263116	0.500075	0.782473	Biso	1.000000	Si
Si96	1.0	0.760109	0.098819	0.954611	Biso	1.000000	Si
Si97	1.0	0.494925	0.844759	0.696438	Biso	1.000000	Si
Si98	1.0	0.684860	0.115192	0.569728	Biso	1.000000	Si
Si99	1.0	0.990932	0.096184	0.869203	Biso	1.000000	Si
Al17	1.0	0.918160	0.827876	0.766579	Biso	1.000000	Al
Al18	1.0	0.274228	0.157395	0.098785	Biso	1.000000	Al
Si100	1.0	0.103953	0.577695	0.211581	Biso	1.000000	Si
Si101	1.0	0.731045	0.408128	0.965894	Biso	1.000000	Si
Si102	1.0	0.773292	0.434412	0.199687	Biso	1.000000	Si

Si103	1.0	0.029392	0.044732	0.437892	Biso	1.000000	Si
Si104	1.0	0.108135	0.753298	0.235456	Biso	1.000000	Si
Si105	1.0	0.899982	0.971705	0.188294	Biso	1.000000	Si
Si106	1.0	0.547900	0.459299	0.557024	Biso	1.000000	Si
Si107	1.0	0.639793	0.063235	0.129934	Biso	1.000000	Si
Si108	1.0	0.793262	0.985287	0.143564	Biso	1.000000	Si
Si109	1.0	0.500097	0.857060	0.179045	Biso	1.000000	Si
Si110	1.0	0.803133	0.888266	0.016901	Biso	1.000000	Si
Si111	1.0	0.281336	0.975449	0.287912	Biso	1.000000	Si
Si112	1.0	0.487252	0.712760	0.647031	Biso	1.000000	Si
Si113	1.0	0.098363	0.626629	0.006361	Biso	1.000000	Si
Si114	1.0	0.630287	0.438409	0.228593	Biso	1.000000	Si
Si115	1.0	0.317878	0.369677	0.993151	Biso	1.000000	Si
Al19	1.0	0.614319	0.170152	0.576046	Biso	1.000000	Al
Al20	1.0	0.030103	0.257575	0.133291	Biso	1.000000	Al
Si116	1.0	0.834962	0.328027	0.626009	Biso	1.000000	Si
Si117	1.0	0.030923	0.157917	0.813412	Biso	1.000000	Si
Al21	1.0	0.249366	0.765559	0.525669	Biso	1.000000	Al
Si118	1.0	0.339108	0.336797	0.831798	Biso	1.000000	Si
Si119	1.0	0.021117	0.728281	0.425879	Biso	1.000000	Si
Si120	1.0	0.470239	0.674785	0.272725	Biso	1.000000	Si
Si121	1.0	0.308294	0.882894	0.775816	Biso	1.000000	Si
Si122	1.0	0.050296	0.346562	0.499151	Biso	1.000000	Si
Si123	1.0	0.149670	0.359743	0.550363	Biso	1.000000	Si
Si124	1.0	0.363927	0.394341	0.765705	Biso	1.000000	Si
Si125	1.0	0.350640	0.254880	0.026126	Biso	1.000000	Si
Si126	1.0	0.292998	0.509026	0.030952	Biso	1.000000	Si
Si127	1.0	0.040843	0.324310	0.322443	Biso	1.000000	Si
Si128	1.0	0.864368	0.137037	0.999950	Biso	1.000000	Si
Al22	1.0	0.123735	0.590472	0.128760	Biso	1.000000	Al
Si129	1.0	0.717115	0.213961	0.795430	Biso	1.000000	Si
Si130	1.0	0.628141	0.320321	0.859896	Biso	1.000000	Si
Si131	1.0	0.387733	0.790950	0.573686	Biso	1.000000	Si
Si132	1.0	0.543172	0.102472	0.377129	Biso	1.000000	Si
Si133	1.0	0.940061	0.837707	0.277804	Biso	1.000000	Si
Si134	1.0	0.310779	0.126953	0.306100	Biso	1.000000	Si
Si135	1.0	0.122241	0.946878	0.476637	Biso	1.000000	Si
Si136	1.0	0.447104	0.621851	0.342711	Biso	1.000000	Si
Si137	1.0	0.615876	0.878631	0.875512	Biso	1.000000	Si
Si138	1.0	0.985191	0.487060	0.201560	Biso	1.000000	Si
Si139	1.0	0.943718	0.074756	0.739844	Biso	1.000000	Si
Si140	1.0	0.181219	0.633437	0.442908	Biso	1.000000	Si
Al23	1.0	0.527985	0.973987	0.097385	Biso	1.000000	Al
Si141	1.0	0.723459	0.831822	0.944679	Biso	1.000000	Si
Si142	1.0	0.587359	0.900314	0.759177	Biso	1.000000	Si
Si143	1.0	0.778122	0.339170	0.007215	Biso	1.000000	Si
Si144	1.0	0.760466	0.324982	0.668744	Biso	1.000000	Si
Si145	1.0	0.350802	0.555126	0.979895	Biso	1.000000	Si
Si146	1.0	0.917528	0.834614	0.930913	Biso	1.000000	Si
Si147	1.0	0.227738	0.869478	0.812564	Biso	1.000000	Si
Si148	1.0	0.467723	0.934631	0.700231	Biso	1.000000	Si
Si149	1.0	0.870756	0.699489	0.578886	Biso	1.000000	Si
Si150	1.0	0.115893	0.244443	0.149964	Biso	1.000000	Si
Si151	1.0	0.683488	0.794417	0.773453	Biso	1.000000	Si
Si152	1.0	0.267887	0.172574	0.803272	Biso	1.000000	Si
Si153	1.0	0.800069	0.921429	0.342435	Biso	1.000000	Si
Si154	1.0	0.809772	0.198421	0.304520	Biso	1.000000	Si
Si155	1.0	0.642992	0.295763	0.232357	Biso	1.000000	Si
Si156	1.0	0.494642	0.141154	0.210664	Biso	1.000000	Si
Si157	1.0	0.016391	0.726568	0.810882	Biso	1.000000	Si
Si158	1.0	0.297196	0.102843	0.166149	Biso	1.000000	Si
Si159	1.0	0.416783	0.462790	0.671887	Biso	1.000000	Si
Si160	1.0	0.854399	0.368718	0.895768	Biso	1.000000	Si
Al24	1.0	0.105772	0.530311	0.526827	Biso	1.000000	Al
Si161	1.0	0.451057	0.771480	0.706856	Biso	1.000000	Si
Si162	1.0	0.689383	0.009809	0.375786	Biso	1.000000	Si
Al25	1.0	0.491005	0.796534	0.842923	Biso	1.000000	Al
Si163	1.0	0.174709	0.727249	0.034995	Biso	1.000000	Si
Si164	1.0	0.636938	0.471763	0.840838	Biso	1.000000	Si
Si165	1.0	0.498116	0.285663	0.184279	Biso	1.000000	Si
Si166	1.0	0.991955	0.214508	0.915277	Biso	1.000000	Si
Si167	1.0	0.111081	0.258214	0.431205	Biso	1.000000	Si
Al26	1.0	0.275440	0.701482	0.260097	Biso	1.000000	Al
Si168	1.0	0.797823	0.591702	0.055876	Biso	1.000000	Si
Si169	1.0	0.192045	0.558105	0.781045	Biso	1.000000	Si
Si170	1.0	0.534109	0.191285	0.702084	Biso	1.000000	Si
Si171	1.0	0.727301	0.456625	0.421602	Biso	1.000000	Si
Al27	1.0	0.569297	0.712272	0.015320	Biso	1.000000	Al

Si172	1.0	0.462983	0.254811	0.440047	Biso	1.000000	Si
Si173	1.0	0.787746	0.652828	0.124626	Biso	1.000000	Si
Si174	1.0	0.592530	0.556124	0.148406	Biso	1.000000	Si
Si175	1.0	0.537028	0.273961	0.103365	Biso	1.000000	Si
Al28	1.0	0.082902	0.886216	0.048461	Biso	1.000000	Al
Si176	1.0	0.547243	0.791559	0.302585	Biso	1.000000	Si
Si177	1.0	0.395383	0.390181	0.229127	Biso	1.000000	Si
Si178	1.0	0.602077	0.894848	0.669735	Biso	1.000000	Si
Si179	1.0	0.910840	0.508779	0.989903	Biso	1.000000	Si
Si180	1.0	0.705994	0.745996	0.382420	Biso	1.000000	Si
Si181	1.0	0.247781	0.997741	0.094372	Biso	1.000000	Si
Al29	1.0	0.116878	0.508170	0.774092	Biso	1.000000	Al
Al30	1.0	0.673070	0.885887	0.765509	Biso	1.000000	Al
Si182	1.0	0.946830	0.703432	0.470974	Biso	1.000000	Si
Si183	1.0	0.431897	0.757136	0.255449	Biso	1.000000	Si
Si184	1.0	0.245886	0.614573	0.972513	Biso	1.000000	Si
Si185	1.0	0.588578	0.602580	0.630272	Biso	1.000000	Si
Si186	1.0	0.039645	0.454403	0.136309	Biso	1.000000	Si
Si187	1.0	0.115475	0.355483	0.857117	Biso	1.000000	Si
Si188	1.0	0.751203	0.424276	0.802633	Biso	1.000000	Si
Si189	1.0	0.988715	0.453625	0.540657	Biso	1.000000	Si
Si190	1.0	0.492140	0.406494	0.768106	Biso	1.000000	Si
Al31	1.0	0.307654	0.795876	0.099589	Biso	1.000000	Al
Si191	1.0	0.645329	0.056471	0.985907	Biso	1.000000	Si
Si192	1.0	0.585993	0.362384	0.202104	Biso	1.000000	Si
Si193	1.0	0.898479	0.387266	0.293100	Biso	1.000000	Si
Si194	1.0	0.897095	0.285702	0.895091	Biso	1.000000	Si
Si195	1.0	0.849862	0.588862	0.698449	Biso	1.000000	Si
Al32	1.0	0.938124	0.600858	0.237898	Biso	1.000000	Al
Al33	1.0	0.531538	0.936356	0.379717	Biso	1.000000	Al
Si196	1.0	0.794936	0.630509	0.340515	Biso	1.000000	Si
Si197	1.0	0.319795	0.952769	0.641930	Biso	1.000000	Si
Si198	1.0	0.421569	0.359571	0.566683	Biso	1.000000	Si
Si199	1.0	0.797620	0.986788	0.844670	Biso	1.000000	Si
Si200	1.0	0.529170	0.637998	0.569007	Biso	1.000000	Si
Si201	1.0	0.104404	0.085039	0.197922	Biso	1.000000	Si
Si202	1.0	0.450098	0.401634	0.848665	Biso	1.000000	Si
Si203	1.0	0.362187	0.830432	0.649250	Biso	1.000000	Si
Si204	1.0	0.636002	0.550170	0.885496	Biso	1.000000	Si
Si205	1.0	0.804743	0.863475	0.485599	Biso	1.000000	Si
Si206	1.0	0.400772	0.372321	0.652894	Biso	1.000000	Si
Si207	1.0	0.326521	0.067846	0.810460	Biso	1.000000	Si
Si208	1.0	0.440352	0.736770	0.072242	Biso	1.000000	Si
Si209	1.0	0.547295	0.324427	0.351036	Biso	1.000000	Si
Al34	1.0	0.601217	0.625263	0.911372	Biso	1.000000	Al
Si210	1.0	0.184419	0.121539	0.215324	Biso	1.000000	Si
Si211	1.0	0.505609	0.382092	0.591541	Biso	1.000000	Si
Si212	1.0	0.378714	0.372276	0.367341	Biso	1.000000	Si
Si213	1.0	0.954929	0.035698	0.974137	Biso	1.000000	Si
Si214	1.0	0.540589	0.823773	0.772878	Biso	1.000000	Si
Si215	1.0	0.346824	0.617367	0.733295	Biso	1.000000	Si
Si216	1.0	0.742456	0.952669	0.995079	Biso	1.000000	Si
Si217	1.0	0.994931	0.003417	0.109207	Biso	1.000000	Si
Si218	1.0	0.608313	0.448434	0.489283	Biso	1.000000	Si
Al35	1.0	0.736926	0.670272	0.665077	Biso	1.000000	Al
Si219	1.0	0.099480	0.391936	0.436693	Biso	1.000000	Si
Si220	1.0	0.570609	0.001646	0.236939	Biso	1.000000	Si
Si221	1.0	0.961832	0.295181	0.688359	Biso	1.000000	Si
Si222	1.0	0.162308	0.611700	0.635924	Biso	1.000000	Si
Si223	1.0	0.202855	0.874395	0.023206	Biso	1.000000	Si
Si224	1.0	0.873133	0.575006	0.184441	Biso	1.000000	Si
Si225	1.0	0.184711	0.805204	0.485053	Biso	1.000000	Si
Si226	1.0	0.139434	0.572706	0.911457	Biso	1.000000	Si
Si227	1.0	0.580656	0.096620	0.613715	Biso	1.000000	Si
Si228	1.0	0.343361	0.753132	0.796380	Biso	1.000000	Si
Si229	1.0	0.725813	0.326079	0.503888	Biso	1.000000	Si
Si230	1.0	0.195143	0.537625	0.142936	Biso	1.000000	Si
Si231	1.0	0.110763	0.183038	0.084953	Biso	1.000000	Si
Si232	1.0	0.410355	0.143255	0.094649	Biso	1.000000	Si
Si233	1.0	0.802971	0.449209	0.541932	Biso	1.000000	Si
Si234	1.0	0.749530	0.257713	0.971798	Biso	1.000000	Si
Si235	1.0	0.068927	0.373072	0.013721	Biso	1.000000	Si
Si236	1.0	0.147948	0.495405	0.380825	Biso	1.000000	Si
Si237	1.0	0.346306	0.209951	0.105197	Biso	1.000000	Si
Si238	1.0	0.989444	0.722320	0.299375	Biso	1.000000	Si
Si239	1.0	0.391367	0.816781	0.074218	Biso	1.000000	Si
Si240	1.0	0.079985	0.524501	0.090341	Biso	1.000000	Si
Si241	1.0	0.008285	0.113061	0.556789	Biso	1.000000	Si
Si242	1.0	0.936971	0.954804	0.731771	Biso	1.000000	Si

Si243	1.0	0.459045	0.369581	0.166817	Biso	1.000000	Si
Si244	1.0	0.901375	0.552196	0.385894	Biso	1.000000	Si
Al36	1.0	0.446302	0.837683	0.523637	Biso	1.000000	Al
Si245	1.0	0.862860	0.164193	0.647148	Biso	1.000000	Si
Si246	1.0	0.452554	0.017363	0.869124	Biso	1.000000	Si
Si247	1.0	0.120887	0.839097	0.602845	Biso	1.000000	Si
Al37	1.0	0.163428	0.293789	0.990991	Biso	1.000000	Al
Si248	1.0	0.414759	0.358157	0.086665	Biso	1.000000	Si
Al38	1.0	0.527360	0.061561	0.765154	Biso	1.000000	Al
Si249	1.0	0.210400	0.428563	0.050726	Biso	1.000000	Si
Si250	1.0	0.836529	0.491413	0.168603	Biso	1.000000	Si
Si251	1.0	0.322465	0.807887	0.724662	Biso	1.000000	Si
Si252	1.0	0.400491	0.915955	0.643598	Biso	1.000000	Si
Si253	1.0	0.325777	0.355263	0.083729	Biso	1.000000	Si
Si254	1.0	0.245961	0.781146	0.681566	Biso	1.000000	Si
Si255	1.0	0.863514	0.474557	0.747120	Biso	1.000000	Si
Si256	1.0	0.758824	0.058227	0.340512	Biso	1.000000	Si
Si257	1.0	0.172077	0.691765	0.588633	Biso	1.000000	Si
Si258	1.0	0.628437	0.209902	0.195757	Biso	1.000000	Si
Si259	1.0	0.677535	0.297996	0.787556	Biso	1.000000	Si
Si260	1.0	0.718340	0.737753	0.837494	Biso	1.000000	Si
Si261	1.0	0.186576	0.339930	0.065719	Biso	1.000000	Si
Si262	1.0	0.401209	0.056400	0.065087	Biso	1.000000	Si
Si263	1.0	0.969085	0.992031	0.654588	Biso	1.000000	Si
Si264	1.0	0.045400	0.844271	0.466471	Biso	1.000000	Si
Si265	1.0	0.534596	0.484374	0.784985	Biso	1.000000	Si
Si266	1.0	0.595559	0.950315	0.531370	Biso	1.000000	Si
Si267	1.0	0.888374	0.264449	0.983665	Biso	1.000000	Si
Al39	1.0	0.085441	0.162289	0.339061	Biso	1.000000	Al
Si268	1.0	0.874245	0.716173	0.858579	Biso	1.000000	Si
Si269	1.0	0.732499	0.313722	0.080731	Biso	1.000000	Si
Si270	1.0	0.820760	0.788580	0.708571	Biso	1.000000	Si
Si271	1.0	0.369709	0.614799	0.206559	Biso	1.000000	Si
Si272	1.0	0.227063	0.795717	0.068794	Biso	1.000000	Si
Si273	1.0	0.673333	0.634902	0.617945	Biso	1.000000	Si
Al40	1.0	0.680401	0.268195	0.024106	Biso	1.000000	Al
Al41	1.0	0.449759	0.192259	0.574290	Biso	1.000000	Al
Si274	1.0	0.735382	0.497938	0.503451	Biso	1.000000	Si
Si275	1.0	0.555087	0.974253	0.941411	Biso	1.000000	Si
Si276	1.0	0.950455	0.628960	0.379131	Biso	1.000000	Si
Si277	1.0	0.600235	0.980958	0.152106	Biso	1.000000	Si
Si278	1.0	0.678707	0.571762	0.256025	Biso	1.000000	Si
Si279	1.0	0.311737	0.956405	0.879507	Biso	1.000000	Si
Al42	1.0	0.972846	0.272700	0.853660	Biso	1.000000	Al
Si280	1.0	0.293218	0.717340	0.145904	Biso	1.000000	Si
Si281	1.0	0.882954	0.545726	0.565255	Biso	1.000000	Si
Si282	1.0	0.277184	0.428653	0.111056	Biso	1.000000	Si
Si283	1.0	0.034943	0.493548	0.937922	Biso	1.000000	Si
Si284	1.0	0.019692	0.038691	0.706114	Biso	1.000000	Si
Si285	1.0	0.361996	0.590855	0.338407	Biso	1.000000	Si
Si286	1.0	0.915858	0.087613	0.824152	Biso	1.000000	Si
Si287	1.0	0.104675	0.747542	0.615585	Biso	1.000000	Si
Si288	1.0	0.654377	0.379409	0.519014	Biso	1.000000	Si
Si289	1.0	0.709998	0.921960	0.353756	Biso	1.000000	Si
Si290	1.0	0.423272	0.496937	0.420350	Biso	1.000000	Si
Si291	1.0	0.307486	0.219888	0.737560	Biso	1.000000	Si
Al43	1.0	0.287555	0.494320	0.275401	Biso	1.000000	Al
Si292	1.0	0.808151	0.077783	0.137922	Biso	1.000000	Si
Si293	1.0	0.387577	0.617114	0.037358	Biso	1.000000	Si
Al44	1.0	0.627351	0.346882	0.653808	Biso	1.000000	Al
Si294	1.0	0.837264	0.928038	0.426638	Biso	1.000000	Si
Al45	1.0	0.275605	0.742125	0.855560	Biso	1.000000	Al
Si295	1.0	0.943127	0.191396	0.307372	Biso	1.000000	Si
Si296	1.0	0.092899	0.390609	0.174908	Biso	1.000000	Si
Si297	1.0	0.240309	0.487624	0.910311	Biso	1.000000	Si
Si298	1.0	0.708921	0.925659	0.697558	Biso	1.000000	Si
Si299	1.0	0.285896	0.383808	0.505623	Biso	1.000000	Si
Si300	1.0	0.431686	0.417441	0.308533	Biso	1.000000	Si
Si301	1.0	0.213687	0.701120	0.956555	Biso	1.000000	Si
Si302	1.0	0.831363	0.472130	0.011077	Biso	1.000000	Si
Si303	1.0	0.723673	0.363029	0.741148	Biso	1.000000	Si
Si304	1.0	0.622683	0.531619	0.450919	Biso	1.000000	Si
Si305	1.0	0.701877	0.655502	0.796840	Biso	1.000000	Si
Si306	1.0	0.431591	0.589100	0.711631	Biso	1.000000	Si
Si307	1.0	0.940252	0.258977	0.246986	Biso	1.000000	Si
Si308	1.0	0.356570	0.062576	0.694682	Biso	1.000000	Si
Si309	1.0	0.540410	0.728428	0.576257	Biso	1.000000	Si
Si310	1.0	0.322317	0.238533	0.589448	Biso	1.000000	Si
Si311	1.0	0.668880	0.525973	0.040483	Biso	1.000000	Si
Si312	1.0	0.003150	0.957499	0.285253	Biso	1.000000	Si
Al46	1.0	0.804801	0.809024	0.054396	Biso	1.000000	Al
Si313	1.0	0.127550	0.364145	0.947848	Biso	1.000000	Si

Si314	1.0	0.590408	0.527911	0.729399	Biso	1.000000	Si
Si315	1.0	0.078712	0.195834	0.927334	Biso	1.000000	Si
Si316	1.0	0.575204	0.118010	0.096734	Biso	1.000000	Si
Si317	1.0	0.848134	0.895625	0.938344	Biso	1.000000	Si
Si318	1.0	0.215545	0.107220	0.832393	Biso	1.000000	Si
Si319	1.0	0.183825	0.024360	0.804693	Biso	1.000000	Si
Si320	1.0	0.629714	0.315217	0.318295	Biso	1.000000	Si
Si321	1.0	0.091623	0.918023	0.638656	Biso	1.000000	Si
Si322	1.0	0.493938	0.655704	0.717673	Biso	1.000000	Si
Si323	1.0	0.116827	0.035665	0.326541	Biso	1.000000	Si
Si324	1.0	0.495898	0.037088	0.500098	Biso	1.000000	Si
Si325	1.0	0.896302	0.096634	0.414344	Biso	1.000000	Si
Al47	1.0	0.551719	0.530841	0.399294	Biso	1.000000	Al
Al48	1.0	0.971997	0.635101	0.698953	Biso	1.000000	Al
Si326	1.0	0.858864	0.739707	0.033066	Biso	1.000000	Si
Si327	1.0	0.670038	0.667179	0.531637	Biso	1.000000	Si
Si328	1.0	0.983102	0.910458	0.484746	Biso	1.000000	Si
Si329	1.0	0.547017	0.635562	0.134803	Biso	1.000000	Si
Si330	1.0	0.223709	0.419555	0.800522	Biso	1.000000	Si
Al49	1.0	0.607818	0.776129	0.237670	Biso	1.000000	Al
Al50	1.0	0.936575	0.070821	0.547350	Biso	1.000000	Al
Si331	1.0	0.080317	0.573631	0.641614	Biso	1.000000	Si
Si332	1.0	0.047906	0.301034	0.826486	Biso	1.000000	Si
Si333	1.0	0.457146	0.531851	0.781315	Biso	1.000000	Si
Si334	1.0	0.300911	0.903304	0.506292	Biso	1.000000	Si
Si335	1.0	0.297060	0.245543	0.899793	Biso	1.000000	Si
Si336	1.0	0.524936	0.767850	0.071509	Biso	1.000000	Si
Si337	1.0	0.646429	0.731268	0.990569	Biso	1.000000	Si
Si338	1.0	0.324497	0.500283	0.144507	Biso	1.000000	Si
Si339	1.0	0.212806	0.055845	0.034445	Biso	1.000000	Si
Si340	1.0	0.376349	0.993412	0.825486	Biso	1.000000	Si
Si341	1.0	0.514247	0.121817	0.461915	Biso	1.000000	Si
Si342	1.0	0.586627	0.170121	0.489503	Biso	1.000000	Si
Si343	1.0	0.839714	0.977304	0.598908	Biso	1.000000	Si
Si344	1.0	0.744581	0.234301	0.647784	Biso	1.000000	Si
Si345	1.0	0.672384	0.379135	0.102573	Biso	1.000000	Si
Si346	1.0	0.407443	0.034013	0.479419	Biso	1.000000	Si
Si347	1.0	0.509029	0.446818	0.156723	Biso	1.000000	Si
Si348	1.0	0.597305	0.791013	0.376663	Biso	1.000000	Si
Si349	1.0	0.141002	0.964337	0.271233	Biso	1.000000	Si
Si350	1.0	0.932663	0.787751	0.009498	Biso	1.000000	Si
Si351	1.0	0.336710	0.178932	0.659475	Biso	1.000000	Si
Si352	1.0	0.472167	0.236772	0.299143	Biso	1.000000	Si
Si353	1.0	0.019699	0.863810	0.107971	Biso	1.000000	Si
Si354	1.0	0.283243	0.904414	0.991094	Biso	1.000000	Si
Si355	1.0	0.152251	0.138405	0.728370	Biso	1.000000	Si
Si356	1.0	0.973245	0.495815	0.655843	Biso	1.000000	Si
Si357	1.0	0.967648	0.764380	0.668776	Biso	1.000000	Si
Si358	1.0	0.400181	0.602492	0.124786	Biso	1.000000	Si
Si359	1.0	0.713658	0.578515	0.849169	Biso	1.000000	Si
Si360	1.0	0.736720	0.515888	0.784220	Biso	1.000000	Si
Si361	1.0	0.715002	0.656126	0.371539	Biso	1.000000	Si
Si362	1.0	0.537920	0.961017	0.464006	Biso	1.000000	Si
Si363	1.0	0.618497	0.077491	0.263142	Biso	1.000000	Si
Si364	1.0	0.054953	0.132588	0.136753	Biso	1.000000	Si
Si365	1.0	0.193401	0.614057	0.853886	Biso	1.000000	Si
Si366	1.0	0.915438	0.412560	0.844740	Biso	1.000000	Si
Si367	1.0	0.972959	0.534773	0.885370	Biso	1.000000	Si
Si368	1.0	0.005286	0.150462	0.036716	Biso	1.000000	Si
Si369	1.0	0.648792	0.121299	0.443242	Biso	1.000000	Si
Si370	1.0	0.474588	0.427634	0.449392	Biso	1.000000	Si
Si371	1.0	0.796441	0.759222	0.875670	Biso	1.000000	Si
Si372	1.0	0.028309	0.274045	0.398268	Biso	1.000000	Si
Si373	1.0	0.244330	0.421204	0.297458	Biso	1.000000	Si
Si374	1.0	0.820410	0.780775	0.317896	Biso	1.000000	Si
Si375	1.0	0.601315	0.755260	0.758819	Biso	1.000000	Si
Si376	1.0	0.456520	0.589712	0.854411	Biso	1.000000	Si
Si377	1.0	0.251637	0.729125	0.608066	Biso	1.000000	Si
Si378	1.0	0.633203	0.896644	0.136825	Biso	1.000000	Si
Al51	1.0	0.846799	0.387543	0.984371	Biso	1.000000	Al
Si379	1.0	0.047055	0.905894	0.969177	Biso	1.000000	Si
Si380	1.0	0.265100	0.295299	0.704772	Biso	1.000000	Si
Si381	1.0	0.148645	0.495212	0.041477	Biso	1.000000	Si
Si382	1.0	0.903546	0.542141	0.294447	Biso	1.000000	Si

Si383	1.0	0.615123	0.322799	0.992840	Biso	1.000000	Si
Si384	1.0	0.098017	0.234098	0.009653	Biso	1.000000	Si
Si385	1.0	0.874056	0.137988	0.282944	Biso	1.000000	Si
Si386	1.0	0.121280	0.864374	0.514031	Biso	1.000000	Si
Si387	1.0	0.858372	0.789500	0.478704	Biso	1.000000	Si
Si388	1.0	0.162064	0.415114	0.336977	Biso	1.000000	Si
Al52	1.0	0.789345	0.206737	0.572950	Biso	1.000000	Al
Si389	1.0	0.894009	0.753955	0.184535	Biso	1.000000	Si
Si390	1.0	0.503325	0.054292	0.590260	Biso	1.000000	Si
Si391	1.0	0.866520	0.319932	0.492842	Biso	1.000000	Si
Si392	1.0	0.400462	0.268439	0.558601	Biso	1.000000	Si
Si393	1.0	0.280049	0.194947	0.522822	Biso	1.000000	Si
Si394	1.0	0.855969	0.154532	0.801797	Biso	1.000000	Si
Si395	1.0	0.335691	0.995510	0.080682	Biso	1.000000	Si
Si396	1.0	0.016539	0.572591	0.208299	Biso	1.000000	Si
Si397	1.0	0.054173	0.885842	0.752220	Biso	1.000000	Si
Si398	1.0	0.054466	0.659045	0.305780	Biso	1.000000	Si
Si399	1.0	0.278380	0.487386	0.411160	Biso	1.000000	Si
Si400	1.0	0.343445	0.117183	0.963312	Biso	1.000000	Si
Si401	1.0	0.449696	0.823167	0.378052	Biso	1.000000	Si
Si402	1.0	0.435216	0.734841	0.984770	Biso	1.000000	Si
Si403	1.0	0.677552	0.014602	0.701641	Biso	1.000000	Si
Si404	1.0	0.360212	0.233863	0.191703	Biso	1.000000	Si
Si405	1.0	0.302527	0.238949	0.396239	Biso	1.000000	Si
Si406	1.0	0.611087	0.983439	0.661295	Biso	1.000000	Si
Si407	1.0	0.024148	0.331615	0.743001	Biso	1.000000	Si
Si408	1.0	0.418054	0.539545	0.233860	Biso	1.000000	Si
Si409	1.0	0.338546	0.730207	0.352859	Biso	1.000000	Si
Al53	1.0	0.744923	0.804381	0.486557	Biso	1.000000	Al
Si410	1.0	0.751296	0.394100	0.277874	Biso	1.000000	Si
Al54	1.0	0.289920	0.988061	0.960213	Biso	1.000000	Al
Si411	1.0	0.573053	0.616535	0.719828	Biso	1.000000	Si
Al55	1.0	0.845939	0.986827	0.920545	Biso	1.000000	Al
Al56	1.0	0.596028	0.528131	0.583691	Biso	1.000000	Al
Si412	1.0	0.924770	0.875755	0.693621	Biso	1.000000	Si
Si413	1.0	0.115375	0.485320	0.900139	Biso	1.000000	Si
Si414	1.0	0.314838	0.663187	0.592072	Biso	1.000000	Si
Si415	1.0	0.624424	0.809844	0.650725	Biso	1.000000	Si
Si416	1.0	0.149413	0.031824	0.570508	Biso	1.000000	Si
Si417	1.0	0.971221	0.440677	0.973293	Biso	1.000000	Si
Si418	1.0	0.913171	0.363687	0.652316	Biso	1.000000	Si
Si419	1.0	0.613163	0.668279	0.779785	Biso	1.000000	Si
Si420	1.0	0.334321	0.150943	0.389508	Biso	1.000000	Si
Al57	1.0	0.017093	0.521076	0.420476	Biso	1.000000	Al
Si421	1.0	0.563536	0.495221	0.212396	Biso	1.000000	Si
Si422	1.0	0.943790	0.851398	0.368425	Biso	1.000000	Si
Si423	1.0	0.781020	0.924004	0.551354	Biso	1.000000	Si
Si424	1.0	0.040545	0.674749	0.737862	Biso	1.000000	Si
Si425	1.0	0.574173	0.107189	0.702981	Biso	1.000000	Si
Si426	1.0	0.587110	0.349110	0.113579	Biso	1.000000	Si
Si427	1.0	0.800240	0.355486	0.545213	Biso	1.000000	Si
Si428	1.0	0.219767	0.280912	0.928287	Biso	1.000000	Si
Si429	1.0	0.201032	0.852583	0.642080	Biso	1.000000	Si
Si430	1.0	0.921188	0.711361	0.354952	Biso	1.000000	Si
Si431	1.0	0.352930	0.308608	0.244138	Biso	1.000000	Si
Si432	1.0	0.799629	0.994705	0.287560	Biso	1.000000	Si
Si433	1.0	0.824585	0.129455	0.719118	Biso	1.000000	Si
Al58	1.0	0.446158	0.652979	0.594235	Biso	1.000000	Al
Si434	1.0	0.377683	0.781586	0.188775	Biso	1.000000	Si
Si435	1.0	0.834621	0.635752	0.843966	Biso	1.000000	Si
Si436	1.0	0.528153	0.301027	0.485006	Biso	1.000000	Si
Si437	1.0	0.214005	0.809160	0.400349	Biso	1.000000	Si
Si438	1.0	0.826817	0.826016	0.625318	Biso	1.000000	Si
Si439	1.0	0.770687	0.621712	0.903633	Biso	1.000000	Si
Si440	1.0	0.126580	0.476538	0.465055	Biso	1.000000	Si
Si441	1.0	0.920331	0.908306	0.543586	Biso	1.000000	Si
Si442	1.0	0.172000	0.931204	0.401431	Biso	1.000000	Si
Si443	1.0	0.998420	0.135130	0.199666	Biso	1.000000	Si
Si444	1.0	0.465056	0.288305	0.050080	Biso	1.000000	Si
Si445	1.0	0.404628	0.783452	0.857070	Biso	1.000000	Si
Si446	1.0	0.202612	0.346797	0.746997	Biso	1.000000	Si
Si447	1.0	0.513187	0.718727	0.437039	Biso	1.000000	Si
Al59	1.0	0.753476	0.002660	0.522602	Biso	1.000000	Al
Si448	1.0	0.918177	0.059953	0.627197	Biso	1.000000	Si
Si449	1.0	0.354199	0.472622	0.226584	Biso	1.000000	Si
Si450	1.0	0.452944	0.175021	0.739565	Biso	1.000000	Si
Si451	1.0	0.835234	0.851033	0.774913	Biso	1.000000	Si
Si452	1.0	0.816918	0.212921	0.008084	Biso	1.000000	Si
Si453	1.0	0.678794	0.514552	0.714088	Biso	1.000000	Si
Si454	1.0	0.796346	0.502713	0.087291	Biso	1.000000	Si
Si455	1.0	0.446528	0.175061	0.486074	Biso	1.000000	Si
Si456	1.0	0.821003	0.421584	0.327417	Biso	1.000000	Si

Si457	1.0	0.119624	0.171890	0.467462	Biso	1.000000	Si
Al60	1.0	0.264638	0.106664	0.421666	Biso	1.000000	Al
Si458	1.0	0.667789	0.838470	0.480032	Biso	1.000000	Si
Si459	1.0	0.716171	0.078354	0.646961	Biso	1.000000	Si
Si460	1.0	0.950172	0.022173	0.398866	Biso	1.000000	Si
Si461	1.0	0.075783	0.703064	0.491549	Biso	1.000000	Si
Si462	1.0	0.308511	0.620663	0.270234	Biso	1.000000	Si
Si463	1.0	0.444527	0.218164	0.216253	Biso	1.000000	Si
Si464	1.0	0.759548	0.789961	0.575275	Biso	1.000000	Si
Si465	1.0	0.697649	0.057706	0.060080	Biso	1.000000	Si
Si466	1.0	0.546186	0.909201	0.597683	Biso	1.000000	Si
Si467	1.0	0.454247	0.977093	0.360505	Biso	1.000000	Si
Al61	1.0	0.871827	0.564671	0.934020	Biso	1.000000	Al
Si468	1.0	0.796948	0.731531	0.469493	Biso	1.000000	Si
Si469	1.0	0.255892	0.648143	0.096702	Biso	1.000000	Si
Si470	1.0	0.168247	0.664143	0.103061	Biso	1.000000	Si
Si471	1.0	0.687023	0.737283	0.248082	Biso	1.000000	Si
Si472	1.0	0.515778	0.415880	0.910985	Biso	1.000000	Si
Si473	1.0	0.723873	0.793766	0.086621	Biso	1.000000	Si
Si474	1.0	0.816562	0.078449	0.408226	Biso	1.000000	Si
Si475	1.0	0.731946	0.316082	0.229347	Biso	1.000000	Si
Si476	1.0	0.078648	0.128828	0.609728	Biso	1.000000	Si
Si477	1.0	0.651340	0.885577	0.553283	Biso	1.000000	Si
Al62	1.0	0.159891	0.891133	0.324011	Biso	1.000000	Al
Si478	1.0	0.860691	0.241990	0.600166	Biso	1.000000	Si
Si479	1.0	0.888704	0.907163	0.253846	Biso	1.000000	Si
Si480	1.0	0.604740	0.257210	0.465075	Biso	1.000000	Si
Si481	1.0	0.258585	0.297713	0.105924	Biso	1.000000	Si
Si482	1.0	0.073546	0.978473	0.751479	Biso	1.000000	Si
Si483	1.0	0.709196	0.196923	0.168270	Biso	1.000000	Si
Al63	1.0	0.918316	0.179956	0.392557	Biso	1.000000	Al
Si484	1.0	0.054361	0.133222	0.418934	Biso	1.000000	Si
Si485	1.0	0.681081	0.498046	0.576222	Biso	1.000000	Si
Si486	1.0	0.666022	0.174204	0.369105	Biso	1.000000	Si
Si487	1.0	0.143421	0.163366	0.555171	Biso	1.000000	Si
Si488	1.0	0.277244	0.765647	0.939631	Biso	1.000000	Si
Si489	1.0	0.412044	0.825654	0.988210	Biso	1.000000	Si
Si490	1.0	0.010861	0.475971	0.347808	Biso	1.000000	Si
Si491	1.0	0.735435	0.146122	0.316802	Biso	1.000000	Si
Si492	1.0	0.867556	0.049982	0.697786	Biso	1.000000	Si
Si493	1.0	0.991859	0.201714	0.570001	Biso	1.000000	Si
Al64	1.0	0.366643	0.400242	0.509590	Biso	1.000000	Al
Si494	1.0	0.614374	0.304885	0.725609	Biso	1.000000	Si
Si495	1.0	0.546282	0.976339	0.793161	Biso	1.000000	Si
Al65	1.0	0.719912	0.513862	0.350203	Biso	1.000000	Al
Si496	1.0	0.615155	0.517667	0.967735	Biso	1.000000	Si
Si497	1.0	0.505798	0.231929	0.624743	Biso	1.000000	Si
Si498	1.0	0.183396	0.806265	0.148188	Biso	1.000000	Si
Si499	1.0	0.561364	0.258683	0.840912	Biso	1.000000	Si
Si500	1.0	0.928922	0.740418	0.790238	Biso	1.000000	Si
Si501	1.0	0.018589	0.726610	0.603695	Biso	1.000000	Si
Si502	1.0	0.712717	0.553537	0.638753	Biso	1.000000	Si
Si503	1.0	0.261183	0.729598	0.399036	Biso	1.000000	Si
Si504	1.0	0.924831	0.015019	0.058326	Biso	1.000000	Si
Si505	1.0	0.849393	0.064898	0.056759	Biso	1.000000	Si
Si506	1.0	0.687211	0.078361	0.204437	Biso	1.000000	Si
Al66	1.0	0.967437	0.376150	0.581041	Biso	1.000000	Al
Si507	1.0	0.619672	0.618748	0.472838	Biso	1.000000	Si
Si508	1.0	0.230119	0.273701	0.188520	Biso	1.000000	Si
Si509	1.0	0.246237	0.283058	0.616095	Biso	1.000000	Si
Si510	1.0	0.827973	0.124693	0.571640	Biso	1.000000	Si
Si511	1.0	0.217129	0.084505	0.692149	Biso	1.000000	Si
Si512	1.0	0.934991	0.849067	0.079383	Biso	1.000000	Si
Si513	1.0	0.061684	0.757751	0.946986	Biso	1.000000	Si
Si514	1.0	0.146051	0.100629	0.075797	Biso	1.000000	Si
Si515	1.0	0.654365	0.906581	0.283172	Biso	1.000000	Si
Si516	1.0	0.665880	0.402183	0.306243	Biso	1.000000	Si
Si517	1.0	0.290183	0.869936	0.152244	Biso	1.000000	Si
Si518	1.0	0.778962	0.853843	0.206361	Biso	1.000000	Si
Si519	1.0	0.280244	0.041291	0.738698	Biso	1.000000	Si
Si520	1.0	0.343627	0.043430	0.278903	Biso	1.000000	Si
Si521	1.0	0.671214	0.818201	0.015272	Biso	1.000000	Si
Si522	1.0	0.542628	0.299268	0.572780	Biso	1.000000	Si
Si523	1.0	0.416922	0.873301	0.447751	Biso	1.000000	Si
Si524	1.0	0.850123	0.693746	0.302063	Biso	1.000000	Si
Si525	1.0	0.022869	0.241348	0.045348	Biso	1.000000	Si
Si526	1.0	0.020272	0.419690	0.768572	Biso	1.000000	Si
Si527	1.0	0.798934	0.269591	0.359520	Biso	1.000000	Si
Si528	1.0	0.761702	0.560013	0.291698	Biso	1.000000	Si
Si529	1.0	0.011934	0.669820	0.883409	Biso	1.000000	Si
Si530	1.0	0.386717	0.700496	0.550281	Biso	1.000000	Si
Al67	1.0	0.584568	0.174704	0.337129	Biso	1.000000	Al

Si531	1.0	0.243913	0.683157	0.759543	Biso	1.000000	Si
Si532	1.0	0.581693	0.822892	0.460370	Biso	1.000000	Si
Si533	1.0	0.001128	0.690435	0.150862	Biso	1.000000	Si
Si534	1.0	0.672331	0.167094	0.860705	Biso	1.000000	Si
Si535	1.0	0.325395	0.811668	0.511680	Biso	1.000000	Si
Si536	1.0	0.927741	0.928457	0.036527	Biso	1.000000	Si
Si537	1.0	0.514475	0.780929	0.505207	Biso	1.000000	Si
O1	1.0	0.531773	0.444123	0.116187	Biso	1.000000	O
O2	1.0	0.270300	0.693525	0.865568	Biso	1.000000	O
O3	1.0	0.208350	0.755628	0.051111	Biso	1.000000	O
O4	1.0	0.240659	0.996942	0.289395	Biso	1.000000	O
O5	1.0	0.006697	0.051251	0.866401	Biso	1.000000	O
O6	1.0	0.882974	0.107187	0.458333	Biso	1.000000	O
O7	1.0	0.139596	0.793835	0.139186	Biso	1.000000	O
O8	1.0	0.628046	0.892562	0.090746	Biso	1.000000	O
O9	1.0	0.761621	0.216251	0.807127	Biso	1.000000	O
O10	1.0	0.610453	0.881166	0.921840	Biso	1.000000	O
O11	1.0	0.092751	0.652843	0.969934	Biso	1.000000	O
O12	1.0	0.254418	0.411601	0.148131	Biso	1.000000	O
O13	1.0	0.243615	0.647851	0.524613	Biso	1.000000	O
O14	1.0	0.391260	0.656768	0.213545	Biso	1.000000	O
O15	1.0	0.087583	0.564816	0.110768	Biso	1.000000	O
O16	1.0	0.051885	0.836818	0.422901	Biso	1.000000	O
O17	1.0	0.548074	0.562244	0.361413	Biso	1.000000	O
O18	1.0	0.243305	0.145590	0.443679	Biso	1.000000	O
O19	1.0	0.076320	0.162587	0.108929	Biso	1.000000	O
O20	1.0	0.169355	0.782120	0.770958	Biso	1.000000	O
O21	1.0	0.348107	0.689073	0.332902	Biso	1.000000	O
O22	1.0	0.822341	0.038131	0.029745	Biso	1.000000	O
O23	1.0	0.058936	0.631498	0.748650	Biso	1.000000	O
O24	1.0	0.650778	0.128864	0.841814	Biso	1.000000	O
O25	1.0	0.117488	0.365788	0.902378	Biso	1.000000	O
O26	1.0	0.445109	0.796392	0.845707	Biso	1.000000	O
O27	1.0	0.085974	0.526494	0.632810	Biso	1.000000	O
O28	1.0	0.148289	0.053698	0.611387	Biso	1.000000	O
O29	1.0	0.765252	0.221896	0.534349	Biso	1.000000	O
O30	1.0	0.167314	0.692805	0.066060	Biso	1.000000	O
O31	1.0	0.688598	0.486098	0.136210	Biso	1.000000	O
O32	1.0	0.002294	0.682184	0.602789	Biso	1.000000	O
O33	1.0	0.569552	0.930378	0.563628	Biso	1.000000	O
O34	1.0	0.447023	0.243802	0.047843	Biso	1.000000	O
O35	1.0	0.271969	0.994678	0.738437	Biso	1.000000	O
O36	1.0	0.113749	0.979743	0.733280	Biso	1.000000	O
O37	1.0	0.090405	0.215397	0.967592	Biso	1.000000	O
O38	1.0	0.597001	0.137284	0.060020	Biso	1.000000	O
O39	1.0	0.248955	0.404465	0.838303	Biso	1.000000	O
O40	1.0	0.100232	0.507293	0.484998	Biso	1.000000	O
O41	1.0	0.519942	0.727874	0.617719	Biso	1.000000	O
O42	1.0	0.845565	0.009561	0.706188	Biso	1.000000	O
O43	1.0	0.980956	0.540655	0.840703	Biso	1.000000	O
O44	1.0	0.645806	0.290043	0.354237	Biso	1.000000	O
O45	1.0	0.954364	0.651411	0.656986	Biso	1.000000	O
O46	1.0	0.664688	0.450014	0.200262	Biso	1.000000	O
O47	1.0	0.646374	0.503627	0.075116	Biso	1.000000	O
O48	1.0	0.180167	0.367552	0.712555	Biso	1.000000	O
O49	1.0	0.238977	0.929850	0.712229	Biso	1.000000	O
O50	1.0	0.435476	0.089679	0.329434	Biso	1.000000	O
O51	1.0	0.226080	0.878775	0.611572	Biso	1.000000	O
O52	1.0	0.024370	0.871612	0.783182	Biso	1.000000	O
O53	1.0	0.086687	0.762270	0.274892	Biso	1.000000	O
O54	1.0	0.040950	0.088443	0.425237	Biso	1.000000	O
O55	1.0	0.402156	0.040421	0.433782	Biso	1.000000	O
O56	1.0	0.616325	0.659660	0.286552	Biso	1.000000	O
O57	1.0	0.465482	0.451865	0.036140	Biso	1.000000	O
O58	1.0	0.395068	0.579537	0.225205	Biso	1.000000	O
O59	1.0	0.528802	0.401236	0.022599	Biso	1.000000	O
O60	1.0	0.079438	0.021438	0.349556	Biso	1.000000	O
O61	1.0	0.215493	0.504754	0.548223	Biso	1.000000	O
O62	1.0	0.924923	0.786000	0.169747	Biso	1.000000	O
O63	1.0	0.025144	0.117437	0.512418	Biso	1.000000	O

O64	1.0	0.700944	0.238850	0.142549	Biso	1.000000	O
O65	1.0	0.809338	0.886400	0.190365	Biso	1.000000	O
O66	1.0	0.518818	0.001800	0.479950	Biso	1.000000	O
O67	1.0	0.355209	0.799720	0.477247	Biso	1.000000	O
O68	1.0	0.998464	0.797079	0.187071	Biso	1.000000	O
O69	1.0	0.007482	0.464458	0.167990	Biso	1.000000	O
O70	1.0	0.816304	0.991681	0.884236	Biso	1.000000	O
O71	1.0	0.153854	0.086737	0.120600	Biso	1.000000	O
O72	1.0	0.580370	0.551909	0.545356	Biso	1.000000	O
O73	1.0	0.593060	0.175362	0.533405	Biso	1.000000	O
O74	1.0	0.405202	0.844871	0.108564	Biso	1.000000	O
O75	1.0	0.977371	0.135814	0.002034	Biso	1.000000	O
O76	1.0	0.871326	0.385442	0.145517	Biso	1.000000	O
O77	1.0	0.893242	0.660817	0.594895	Biso	1.000000	O
O78	1.0	0.326686	0.474045	0.261217	Biso	1.000000	O
O79	1.0	0.892309	0.739019	0.590383	Biso	1.000000	O
O80	1.0	0.951625	0.357401	0.538895	Biso	1.000000	O
O81	1.0	0.308367	0.073456	0.853033	Biso	1.000000	O
O82	1.0	0.545323	0.082465	0.726986	Biso	1.000000	O
O83	1.0	0.674714	0.881625	0.148804	Biso	1.000000	O
O84	1.0	0.195549	0.049563	0.921046	Biso	1.000000	O
O85	1.0	0.570653	0.897799	0.635290	Biso	1.000000	O
O86	1.0	0.161685	0.396452	0.956461	Biso	1.000000	O
O87	1.0	0.837200	0.570288	0.966287	Biso	1.000000	O
O88	1.0	0.861453	0.128032	0.240834	Biso	1.000000	O
O89	1.0	0.045824	0.816570	0.347823	Biso	1.000000	O
O90	1.0	0.277271	0.681156	0.120816	Biso	1.000000	O
O91	1.0	0.424876	0.974102	0.397529	Biso	1.000000	O
O92	1.0	0.058887	0.583432	0.222695	Biso	1.000000	O
O93	1.0	0.709060	0.036819	0.206061	Biso	1.000000	O
O94	1.0	0.404594	0.606472	0.335930	Biso	1.000000	O
O95	1.0	0.189599	0.592890	0.813599	Biso	1.000000	O
O96	1.0	0.426599	0.282154	0.430754	Biso	1.000000	O
O97	1.0	0.450298	0.567716	0.895325	Biso	1.000000	O
O98	1.0	0.231495	0.174590	0.115684	Biso	1.000000	O
O99	1.0	0.495018	0.966936	0.679913	Biso	1.000000	O
O100	1.0	0.852028	0.533030	0.183733	Biso	1.000000	O
O101	1.0	0.977412	0.287846	0.249411	Biso	1.000000	O
O102	1.0	0.158060	0.765562	0.475684	Biso	1.000000	O
O103	1.0	0.820262	0.905541	0.057182	Biso	1.000000	O
O104	1.0	0.719377	0.753768	0.275962	Biso	1.000000	O
O105	1.0	0.843391	0.785891	0.358898	Biso	1.000000	O
O106	1.0	0.683013	0.207563	0.518073	Biso	1.000000	O
O107	1.0	0.279017	0.724880	0.442286	Biso	1.000000	O
O108	1.0	0.517223	0.754718	0.311423	Biso	1.000000	O
O109	1.0	0.701378	0.808650	0.910337	Biso	1.000000	O
O110	1.0	0.441965	0.034682	0.911545	Biso	1.000000	O
O111	1.0	0.525472	0.291970	0.529705	Biso	1.000000	O
O112	1.0	0.032836	0.700568	0.775792	Biso	1.000000	O
O113	1.0	0.527241	0.212641	0.661020	Biso	1.000000	O
O114	1.0	0.869511	0.006769	0.193608	Biso	1.000000	O
O115	1.0	0.429596	0.964460	0.322445	Biso	1.000000	O
O116	1.0	0.487389	0.680043	0.437068	Biso	1.000000	O
O117	1.0	0.260439	0.259538	0.922374	Biso	1.000000	O
O118	1.0	0.978668	0.463360	0.317565	Biso	1.000000	O
O119	1.0	0.743099	0.396976	0.765825	Biso	1.000000	O
O120	1.0	0.214305	0.796280	0.516484	Biso	1.000000	O
O121	1.0	0.640192	0.632194	0.937052	Biso	1.000000	O
O122	1.0	0.684226	0.895758	0.719872	Biso	1.000000	O
O123	1.0	0.625906	0.265155	0.424657	Biso	1.000000	O
O124	1.0	0.570829	0.306203	0.103327	Biso	1.000000	O
O125	1.0	0.830645	0.077285	0.096849	Biso	1.000000	O
O126	1.0	0.472949	0.105924	0.233225	Biso	1.000000	O
O127	1.0	0.182572	0.430860	0.817199	Biso	1.000000	O
O128	1.0	0.798920	0.359852	0.419579	Biso	1.000000	O
O129	1.0	0.066470	0.181346	0.830599	Biso	1.000000	O
O130	1.0	0.220665	0.839085	0.847894	Biso	1.000000	O
O131	1.0	0.498501	0.513045	0.777897	Biso	1.000000	O
O132	1.0	0.107737	0.083429	0.060085	Biso	1.000000	O
O133	1.0	0.694386	0.189100	0.826908	Biso	1.000000	O
O134	1.0	0.826073	0.044875	0.439079	Biso	1.000000	O
O135	1.0	0.779329	0.530661	0.770143	Biso	1.000000	O
O136	1.0	0.440390	0.128813	0.735089	Biso	1.000000	O
O137	1.0	0.172809	0.322115	0.774148	Biso	1.000000	O
O138	1.0	0.319259	0.089546	0.528521	Biso	1.000000	O
O139	1.0	0.319036	0.841307	0.630081	Biso	1.000000	O
O140	1.0	0.567827	0.486587	0.587329	Biso	1.000000	O
O141	1.0	0.569367	0.286545	0.469578	Biso	1.000000	O
O142	1.0	0.505360	0.608395	0.025756	Biso	1.000000	O
O143	1.0	0.824345	0.896370	0.762856	Biso	1.000000	O
O144	1.0	0.508777	0.220018	0.971147	Biso	1.000000	O

O145	1.0	0.025915	0.360316	0.005453	Biso	1.000000	O
O146	1.0	0.794635	0.843315	0.021362	Biso	1.000000	O
O147	1.0	0.526019	0.264725	0.870707	Biso	1.000000	O
O148	1.0	0.068077	0.258204	0.829819	Biso	1.000000	O
O149	1.0	0.760069	0.806642	0.958967	Biso	1.000000	O
O150	1.0	0.388356	0.485504	0.642352	Biso	1.000000	O
O151	1.0	0.813501	0.606451	0.376431	Biso	1.000000	O
O152	1.0	0.760561	0.252537	0.381075	Biso	1.000000	O
O153	1.0	0.510288	0.809841	0.174288	Biso	1.000000	O
O154	1.0	0.834485	0.750376	0.489711	Biso	1.000000	O
O155	1.0	0.720239	0.164682	0.137199	Biso	1.000000	O
O156	1.0	0.310003	0.632499	0.842697	Biso	1.000000	O
O157	1.0	0.588103	0.301542	0.566521	Biso	1.000000	O
O158	1.0	0.976575	0.912475	0.822300	Biso	1.000000	O
O159	1.0	0.345347	0.973695	0.853241	Biso	1.000000	O
O160	1.0	0.488095	0.054024	0.288201	Biso	1.000000	O
O161	1.0	0.145118	0.129984	0.773687	Biso	1.000000	O
O162	1.0	0.174122	0.867129	0.988189	Biso	1.000000	O
O163	1.0	0.407839	0.742052	0.883069	Biso	1.000000	O
O164	1.0	0.560420	0.723363	0.968113	Biso	1.000000	O
O165	1.0	0.614524	0.625812	0.353648	Biso	1.000000	O
O166	1.0	0.976911	0.854244	0.096611	Biso	1.000000	O
O167	1.0	0.684602	0.956685	0.847531	Biso	1.000000	O
O168	1.0	0.615037	0.863912	0.571297	Biso	1.000000	O
O169	1.0	0.821151	0.351347	0.006705	Biso	1.000000	O
O170	1.0	0.279183	0.632635	0.405453	Biso	1.000000	O
O171	1.0	0.950093	0.408680	0.946075	Biso	1.000000	O
O172	1.0	0.943677	0.503596	0.183549	Biso	1.000000	O
O173	1.0	0.785610	0.398906	0.307548	Biso	1.000000	O
O174	1.0	0.119599	0.596393	0.639113	Biso	1.000000	O
O175	1.0	0.503809	0.126411	0.167106	Biso	1.000000	O
O176	1.0	0.671457	0.877759	0.452423	Biso	1.000000	O
O177	1.0	0.711747	0.670486	0.513104	Biso	1.000000	O
O178	1.0	0.415987	0.431813	0.857952	Biso	1.000000	O
O179	1.0	0.585002	0.986972	0.195771	Biso	1.000000	O
O180	1.0	0.936948	0.194549	0.351649	Biso	1.000000	O
O181	1.0	0.437865	0.365155	0.126022	Biso	1.000000	O
O182	1.0	0.141075	0.609164	0.011425	Biso	1.000000	O
O183	1.0	0.858030	0.938409	0.925085	Biso	1.000000	O
O184	1.0	0.187773	0.163326	0.238851	Biso	1.000000	O
O185	1.0	0.757026	0.208465	0.685753	Biso	1.000000	O
O186	1.0	0.130610	0.328286	0.579426	Biso	1.000000	O
O187	1.0	0.468030	0.883135	0.287175	Biso	1.000000	O
O188	1.0	0.142212	0.105077	0.214108	Biso	1.000000	O
O189	1.0	0.152902	0.326743	0.848897	Biso	1.000000	O
O190	1.0	0.694128	0.043365	0.668287	Biso	1.000000	O
O191	1.0	0.103937	0.513202	0.727875	Biso	1.000000	O
O192	1.0	0.786869	0.740810	0.918446	Biso	1.000000	O
O193	1.0	0.855511	0.325193	0.196604	Biso	1.000000	O
O194	1.0	0.232053	0.657830	0.962077	Biso	1.000000	O
O195	1.0	0.724832	0.648007	0.757509	Biso	1.000000	O
O196	1.0	0.329674	0.615929	0.228249	Biso	1.000000	O
O197	1.0	0.635351	0.522720	0.725787	Biso	1.000000	O
O198	1.0	0.710902	0.012736	0.503680	Biso	1.000000	O
O199	1.0	0.855308	0.548111	0.892467	Biso	1.000000	O
O200	1.0	0.386821	0.067940	0.504322	Biso	1.000000	O
O201	1.0	0.447495	0.224943	0.262398	Biso	1.000000	O
O202	1.0	0.474342	0.478947	0.149875	Biso	1.000000	O
O203	1.0	0.937345	0.976815	0.402829	Biso	1.000000	O
O204	1.0	0.737395	0.873928	0.927413	Biso	1.000000	O
O205	1.0	0.807461	0.943037	0.826659	Biso	1.000000	O
O206	1.0	0.765452	0.429971	0.424569	Biso	1.000000	O
O207	1.0	0.299669	0.326260	0.699788	Biso	1.000000	O
O208	1.0	0.421077	0.617871	0.614609	Biso	1.000000	O
O209	1.0	0.937675	0.504260	0.890937	Biso	1.000000	O
O210	1.0	0.338175	0.393054	0.369407	Biso	1.000000	O
O211	1.0	0.947143	0.524335	0.682824	Biso	1.000000	O
O212	1.0	0.424259	0.706637	0.104185	Biso	1.000000	O
O213	1.0	0.754492	0.292493	0.117196	Biso	1.000000	O
O214	1.0	0.038740	0.311198	0.781978	Biso	1.000000	O
O215	1.0	0.405552	0.475908	0.714205	Biso	1.000000	O
O216	1.0	0.043163	0.078649	0.695863	Biso	1.000000	O
O217	1.0	0.749252	0.324255	0.186932	Biso	1.000000	O
O218	1.0	0.390197	0.744045	0.567310	Biso	1.000000	O
O219	1.0	0.337240	0.603578	0.302063	Biso	1.000000	O
O220	1.0	0.938124	0.078868	0.784592	Biso	1.000000	O
O221	1.0	0.885195	0.893141	0.677301	Biso	1.000000	O
O222	1.0	0.131587	0.850846	0.316310	Biso	1.000000	O
O223	1.0	0.312012	0.142320	0.648500	Biso	1.000000	O
O224	1.0	0.299081	0.924125	0.754821	Biso	1.000000	O

O225	1.0	0.936624	0.817656	0.044601	Biso	1.000000	O
O226	1.0	0.068083	0.350530	0.170637	Biso	1.000000	O
O227	1.0	0.379412	0.754890	0.150009	Biso	1.000000	O
O228	1.0	0.567074	0.684083	0.338935	Biso	1.000000	O
O229	1.0	0.990883	0.522716	0.622243	Biso	1.000000	O
O230	1.0	0.018563	0.581274	0.160128	Biso	1.000000	O
O231	1.0	0.879477	0.430028	0.749359	Biso	1.000000	O
O232	1.0	0.212532	0.717418	0.767291	Biso	1.000000	O
O233	1.0	0.291365	0.858799	0.197971	Biso	1.000000	O
O234	1.0	0.980629	0.834056	0.387401	Biso	1.000000	O
O235	1.0	0.316972	0.530851	0.997851	Biso	1.000000	O
O236	1.0	0.613467	0.450445	0.635159	Biso	1.000000	O
O237	1.0	0.230247	0.532307	0.789711	Biso	1.000000	O
O238	1.0	0.726902	0.408074	0.838276	Biso	1.000000	O
O239	1.0	0.991586	0.556320	0.440753	Biso	1.000000	O
O240	1.0	0.686093	0.311644	0.226709	Biso	1.000000	O
O241	1.0	0.752289	0.373379	0.987681	Biso	1.000000	O
O242	1.0	0.431646	0.612034	0.024867	Biso	1.000000	O
O243	1.0	0.085100	0.731163	0.977708	Biso	1.000000	O
O244	1.0	0.112507	0.380024	0.686500	Biso	1.000000	O
O245	1.0	0.571168	0.787682	0.771615	Biso	1.000000	O
O246	1.0	0.478129	0.602604	0.447449	Biso	1.000000	O
O247	1.0	0.986134	0.180481	0.297563	Biso	1.000000	O
O248	1.0	0.889854	0.697680	0.324090	Biso	1.000000	O
O249	1.0	0.211028	0.910567	0.418439	Biso	1.000000	O
O250	1.0	0.676437	0.570432	0.054066	Biso	1.000000	O
O251	1.0	0.335213	0.934004	0.502211	Biso	1.000000	O
O252	1.0	0.051734	0.375098	0.536700	Biso	1.000000	O
O253	1.0	0.794849	0.813224	0.595238	Biso	1.000000	O
O254	1.0	0.034906	0.452926	0.536931	Biso	1.000000	O
O255	1.0	0.270344	0.593234	0.270495	Biso	1.000000	O
O256	1.0	0.018937	0.896943	0.142781	Biso	1.000000	O
O257	1.0	0.640878	0.195623	0.879777	Biso	1.000000	O
O258	1.0	0.046077	0.381435	0.652416	Biso	1.000000	O
O259	1.0	0.911367	0.074056	0.586235	Biso	1.000000	O
O260	1.0	0.007050	0.335826	0.488454	Biso	1.000000	O
O261	1.0	0.553404	0.231982	0.089377	Biso	1.000000	O
O262	1.0	0.871694	0.199087	0.804635	Biso	1.000000	O
O263	1.0	0.319268	0.906732	0.144961	Biso	1.000000	O
O264	1.0	0.521621	0.541396	0.432774	Biso	1.000000	O
O265	1.0	0.110940	0.607400	0.910759	Biso	1.000000	O
O266	1.0	0.740719	0.892439	0.115523	Biso	1.000000	O
O267	1.0	0.048128	0.657999	0.905952	Biso	1.000000	O
O268	1.0	0.243922	0.456450	0.782335	Biso	1.000000	O
O269	1.0	0.394671	0.470593	0.061050	Biso	1.000000	O
O270	1.0	0.331325	0.543703	0.866489	Biso	1.000000	O
O271	1.0	0.644252	0.638182	0.506160	Biso	1.000000	O
O272	1.0	0.815737	0.917100	0.689305	Biso	1.000000	O
O273	1.0	0.744743	0.351856	0.256523	Biso	1.000000	O
O274	1.0	0.930680	0.327514	0.677984	Biso	1.000000	O
O275	1.0	0.958371	0.725968	0.332553	Biso	1.000000	O
O276	1.0	0.960311	0.965009	0.769991	Biso	1.000000	O
O277	1.0	0.548882	0.362960	0.228399	Biso	1.000000	O
O278	1.0	0.345509	0.109657	0.797702	Biso	1.000000	O
O279	1.0	0.756250	0.063001	0.630726	Biso	1.000000	O
O280	1.0	0.475422	0.301810	0.006925	Biso	1.000000	O
O281	1.0	0.458246	0.280084	0.313129	Biso	1.000000	O
O282	1.0	0.697548	0.964014	0.372447	Biso	1.000000	O
O283	1.0	0.465120	0.374636	0.570335	Biso	1.000000	O
O284	1.0	0.402830	0.939332	0.007037	Biso	1.000000	O
O285	1.0	0.805102	0.464874	0.585907	Biso	1.000000	O
O286	1.0	0.705623	0.161018	0.349479	Biso	1.000000	O
O287	1.0	0.567743	0.595561	0.148266	Biso	1.000000	O
O288	1.0	0.387867	0.021376	0.172626	Biso	1.000000	O
O289	1.0	0.303239	0.887341	0.406763	Biso	1.000000	O
O290	1.0	0.429810	0.840873	0.339090	Biso	1.000000	O
O291	1.0	0.708203	0.256884	0.987619	Biso	1.000000	O
O292	1.0	0.014587	0.230948	0.380016	Biso	1.000000	O
O293	1.0	0.241431	0.326339	0.598926	Biso	1.000000	O
O294	1.0	0.538258	0.517434	0.245363	Biso	1.000000	O

O295	1.0	0.645517	0.422964	0.269619	Biso	1.000000	O
O296	1.0	0.654831	0.856258	0.864152	Biso	1.000000	O
O297	1.0	0.983479	0.595070	0.227295	Biso	1.000000	O
O298	1.0	0.618922	0.349716	0.508778	Biso	1.000000	O
O299	1.0	0.520611	0.872616	0.585057	Biso	1.000000	O
O300	1.0	0.824018	0.681237	0.126282	Biso	1.000000	O
O301	1.0	0.414910	0.003930	0.847710	Biso	1.000000	O
O302	1.0	0.119811	0.504398	0.076093	Biso	1.000000	O
O303	1.0	0.578013	0.638372	0.471076	Biso	1.000000	O
O304	1.0	0.314606	0.006946	0.282399	Biso	1.000000	O
O305	1.0	0.850913	0.883762	0.253729	Biso	1.000000	O
O306	1.0	0.652527	0.093520	0.957419	Biso	1.000000	O
O307	1.0	0.812630	0.961669	0.317667	Biso	1.000000	O
O308	1.0	0.102797	0.730566	0.517921	Biso	1.000000	O
O309	1.0	0.975869	0.423531	0.571801	Biso	1.000000	O
O310	1.0	0.665588	0.259710	0.570746	Biso	1.000000	O
O311	1.0	0.956429	0.405198	0.864753	Biso	1.000000	O
O312	1.0	0.233132	0.544836	0.118402	Biso	1.000000	O
O313	1.0	0.513801	0.464434	0.922547	Biso	1.000000	O
O314	1.0	0.956599	0.856038	0.917569	Biso	1.000000	O
O315	1.0	0.057391	0.333668	0.710485	Biso	1.000000	O
O316	1.0	0.128064	0.414402	0.305692	Biso	1.000000	O
O317	1.0	0.377814	0.132338	0.546547	Biso	1.000000	O
O318	1.0	0.254435	0.267357	0.225139	Biso	1.000000	O
O319	1.0	0.394118	0.263579	0.511067	Biso	1.000000	O
O320	1.0	0.980912	0.953613	0.629990	Biso	1.000000	O
O321	1.0	0.070569	0.368697	0.464428	Biso	1.000000	O
O322	1.0	0.595770	0.955009	0.953362	Biso	1.000000	O
O323	1.0	0.195594	0.666708	0.471780	Biso	1.000000	O
O324	1.0	0.657539	0.663085	0.786307	Biso	1.000000	O
O325	1.0	0.209486	0.799597	0.111880	Biso	1.000000	O
O326	1.0	0.321159	0.579902	0.733371	Biso	1.000000	O
O327	1.0	0.402813	0.161264	0.479097	Biso	1.000000	O
O328	1.0	0.057813	0.493557	0.117974	Biso	1.000000	O
O329	1.0	0.489795	0.405110	0.164897	Biso	1.000000	O
O330	1.0	0.862373	0.776560	0.210127	Biso	1.000000	O
O331	1.0	0.075702	0.073952	0.232849	Biso	1.000000	O
O332	1.0	0.804737	0.502231	0.983703	Biso	1.000000	O
O333	1.0	0.182150	0.320523	0.025665	Biso	1.000000	O
O334	1.0	0.279552	0.255462	0.723844	Biso	1.000000	O
O335	1.0	0.603597	0.060794	0.004902	Biso	1.000000	O
O336	1.0	0.824959	0.968859	0.446801	Biso	1.000000	O
O337	1.0	0.241060	0.111872	0.665895	Biso	1.000000	O
O338	1.0	0.989107	0.030974	0.421705	Biso	1.000000	O
O339	1.0	0.214128	0.991395	0.816408	Biso	1.000000	O
O340	1.0	0.693459	0.970412	0.695810	Biso	1.000000	O
O341	1.0	0.600968	0.185714	0.223208	Biso	1.000000	O
O342	1.0	0.594551	0.117267	0.256514	Biso	1.000000	O
O343	1.0	0.408265	0.498299	0.898526	Biso	1.000000	O
O344	1.0	0.656517	0.427930	0.344437	Biso	1.000000	O
O345	1.0	0.632566	0.351573	0.959870	Biso	1.000000	O
O346	1.0	0.971864	0.761794	0.005996	Biso	1.000000	O
O347	1.0	0.586852	0.349924	0.018334	Biso	1.000000	O
O348	1.0	0.852121	0.137139	0.610487	Biso	1.000000	O
O349	1.0	0.709929	0.748287	0.097111	Biso	1.000000	O
O350	1.0	0.504712	0.747006	0.472463	Biso	1.000000	O
O351	1.0	0.047993	0.487877	0.322865	Biso	1.000000	O
O352	1.0	0.479365	0.328105	0.176963	Biso	1.000000	O
O353	1.0	0.256243	0.486611	0.451629	Biso	1.000000	O
O354	1.0	0.643901	0.524926	0.002393	Biso	1.000000	O
O355	1.0	0.144997	0.014658	0.829555	Biso	1.000000	O
O356	1.0	0.332674	0.387612	0.732351	Biso	1.000000	O
O357	1.0	0.319853	0.195713	0.395911	Biso	1.000000	O
O358	1.0	0.104699	0.436519	0.451611	Biso	1.000000	O
O359	1.0	0.921611	0.890597	0.062318	Biso	1.000000	O
O360	1.0	0.244342	0.388261	0.262595	Biso	1.000000	O
O361	1.0	0.797924	0.889071	0.524895	Biso	1.000000	O
O362	1.0	0.073885	0.279154	0.581832	Biso	1.000000	O
O363	1.0	0.492013	0.199482	0.595514	Biso	1.000000	O
O364	1.0	0.538774	0.612010	0.530511	Biso	1.000000	O
O365	1.0	0.796661	0.548098	0.072761	Biso	1.000000	O
O366	1.0	0.333531	0.648719	0.632702	Biso	1.000000	O
O367	1.0	0.579475	0.091644	0.659325	Biso	1.000000	O
O368	1.0	0.181792	0.107898	0.710849	Biso	1.000000	O
O369	1.0	0.647318	0.016277	0.960195	Biso	1.000000	O
O370	1.0	0.842708	0.534821	0.583931	Biso	1.000000	O
O371	1.0	0.197621	0.128450	0.171258	Biso	1.000000	O

O372	1.0	0.133998	0.384462	0.155363	Biso	1.000000	O
O373	1.0	0.405491	0.092452	0.198254	Biso	1.000000	O
O374	1.0	0.383772	0.607967	0.082283	Biso	1.000000	O
O375	1.0	0.173997	0.802889	0.376217	Biso	1.000000	O
O376	1.0	0.073024	0.306230	0.509088	Biso	1.000000	O
O377	1.0	0.494934	0.823607	0.374403	Biso	1.000000	O
O378	1.0	0.072296	0.903187	0.005633	Biso	1.000000	O
O379	1.0	0.365155	0.587322	0.009898	Biso	1.000000	O
O380	1.0	0.412194	0.775330	0.294133	Biso	1.000000	O
O381	1.0	0.780789	0.061651	0.972150	Biso	1.000000	O
O382	1.0	0.658740	0.313966	0.826061	Biso	1.000000	O
O383	1.0	0.564676	0.617844	0.939971	Biso	1.000000	O
O384	1.0	0.105465	0.958639	0.242622	Biso	1.000000	O
O385	1.0	0.925207	0.105818	0.517903	Biso	1.000000	O
O386	1.0	0.241122	0.098143	0.381435	Biso	1.000000	O
O387	1.0	0.271010	0.961666	0.858225	Biso	1.000000	O
O388	1.0	0.297740	0.624624	0.570813	Biso	1.000000	O
O389	1.0	0.294535	0.395294	0.666701	Biso	1.000000	O
O390	1.0	0.474950	0.583668	0.333591	Biso	1.000000	O
O391	1.0	0.437868	0.958895	0.071074	Biso	1.000000	O
O392	1.0	0.634621	0.631209	0.073882	Biso	1.000000	O
O393	1.0	0.656398	0.384058	0.565056	Biso	1.000000	O
O394	1.0	0.600047	0.424249	0.031799	Biso	1.000000	O
O395	1.0	0.699630	0.807643	0.473333	Biso	1.000000	O
O396	1.0	0.705953	0.969185	0.603968	Biso	1.000000	O
O397	1.0	0.576148	0.358136	0.157294	Biso	1.000000	O
O398	1.0	0.036320	0.690351	0.400956	Biso	1.000000	O
O399	1.0	0.058438	0.258404	0.020306	Biso	1.000000	O
O400	1.0	0.409808	0.416341	0.666648	Biso	1.000000	O
O401	1.0	0.244231	0.132349	0.805617	Biso	1.000000	O
O402	1.0	0.199108	0.321729	0.451960	Biso	1.000000	O
O403	1.0	0.156056	0.451425	0.365218	Biso	1.000000	O
O404	1.0	0.701671	0.885939	0.382880	Biso	1.000000	O
O405	1.0	0.270249	0.031378	0.515453	Biso	1.000000	O
O406	1.0	0.911141	0.563263	0.597777	Biso	1.000000	O
O407	1.0	0.579426	0.527688	0.182905	Biso	1.000000	O
O408	1.0	0.947461	0.559862	0.774546	Biso	1.000000	O
O409	1.0	0.001188	0.916343	0.308354	Biso	1.000000	O
O410	1.0	0.376268	0.294352	0.019674	Biso	1.000000	O
O411	1.0	0.761699	0.331392	0.050098	Biso	1.000000	O
O412	1.0	0.875029	0.098659	0.310113	Biso	1.000000	O
O413	1.0	0.340903	0.961862	0.047147	Biso	1.000000	O
O414	1.0	0.401455	0.932364	0.534577	Biso	1.000000	O
O415	1.0	0.830057	0.168130	0.002164	Biso	1.000000	O
O416	1.0	0.760321	0.335772	0.532238	Biso	1.000000	O
O417	1.0	0.819750	0.924550	0.384129	Biso	1.000000	O
O418	1.0	0.543010	0.483885	0.830936	Biso	1.000000	O
O419	1.0	0.150896	0.724129	0.725090	Biso	1.000000	O
O420	1.0	0.585072	0.320918	0.326166	Biso	1.000000	O
O421	1.0	0.935894	0.235007	0.286791	Biso	1.000000	O
O422	1.0	0.920740	0.838343	0.718940	Biso	1.000000	O
O423	1.0	0.111311	0.134056	0.707126	Biso	1.000000	O
O424	1.0	0.258312	0.592296	0.932138	Biso	1.000000	O
O425	1.0	0.673852	0.458524	0.867139	Biso	1.000000	O
O426	1.0	0.862570	0.208870	0.632854	Biso	1.000000	O
O427	1.0	0.982703	0.075954	0.558287	Biso	1.000000	O
O428	1.0	0.324164	0.699501	0.175978	Biso	1.000000	O
O429	1.0	0.252176	0.462953	0.280501	Biso	1.000000	O
O430	1.0	0.369663	0.349463	0.094272	Biso	1.000000	O
O431	1.0	0.015527	0.124629	0.841729	Biso	1.000000	O
O432	1.0	0.109745	0.075579	0.303039	Biso	1.000000	O
O433	1.0	0.063399	0.730490	0.606363	Biso	1.000000	O
O434	1.0	0.613395	0.425729	0.706847	Biso	1.000000	O
O435	1.0	0.210057	0.255965	0.604598	Biso	1.000000	O
O436	1.0	0.799728	0.271146	0.766329	Biso	1.000000	O
O437	1.0	0.633191	0.170967	0.338146	Biso	1.000000	O
O438	1.0	0.810174	0.628981	0.882579	Biso	1.000000	O
O439	1.0	0.338233	0.573272	0.939051	Biso	1.000000	O
O440	1.0	0.928398	0.804038	0.571558	Biso	1.000000	O
O441	1.0	0.130642	0.001302	0.298795	Biso	1.000000	O
O442	1.0	0.018349	0.154197	0.236085	Biso	1.000000	O
O443	1.0	0.947525	0.096733	0.855548	Biso	1.000000	O
O444	1.0	0.604848	0.267335	0.697174	Biso	1.000000	O
O445	1.0	0.441971	0.857337	0.972065	Biso	1.000000	O
O446	1.0	0.577880	0.611154	0.674833	Biso	1.000000	O
O447	1.0	0.769653	0.222165	0.612736	Biso	1.000000	O
O448	1.0	0.372776	0.328853	0.347161	Biso	1.000000	O
O449	1.0	0.380217	0.431647	0.922553	Biso	1.000000	O

O450	1.0	0.140044	0.285362	0.148758	Biso	1.000000	O
O451	1.0	0.008202	0.352354	0.592046	Biso	1.000000	O
O452	1.0	0.796997	0.115781	0.430329	Biso	1.000000	O
O453	1.0	0.647002	0.300979	0.014576	Biso	1.000000	O
O454	1.0	0.160008	0.733391	0.856721	Biso	1.000000	O
O455	1.0	0.960896	0.968138	0.270261	Biso	1.000000	O
O456	1.0	0.721968	0.480433	0.462050	Biso	1.000000	O
O457	1.0	0.191317	0.879134	0.678796	Biso	1.000000	O
O458	1.0	0.396382	0.823227	0.178878	Biso	1.000000	O
O459	1.0	0.020206	0.420302	0.815074	Biso	1.000000	O
O460	1.0	0.980649	0.420378	0.014493	Biso	1.000000	O
O461	1.0	0.285220	0.264160	0.600611	Biso	1.000000	O
O462	1.0	0.979158	0.160932	0.665696	Biso	1.000000	O
O463	1.0	0.943139	0.476451	0.981985	Biso	1.000000	O
O464	1.0	0.966079	0.619291	0.335833	Biso	1.000000	O
O465	1.0	0.834589	0.739749	0.860312	Biso	1.000000	O
O466	1.0	0.386451	0.993089	0.490638	Biso	1.000000	O
O467	1.0	0.444614	0.164023	0.070358	Biso	1.000000	O
O468	1.0	0.256339	0.732164	0.171181	Biso	1.000000	O
O469	1.0	0.232822	0.444261	0.928653	Biso	1.000000	O
O470	1.0	0.715230	0.787010	0.739275	Biso	1.000000	O
O471	1.0	0.691511	0.541119	0.677917	Biso	1.000000	O
O472	1.0	0.383086	0.704400	0.502557	Biso	1.000000	O
O473	1.0	0.457319	0.881148	0.544198	Biso	1.000000	O
O474	1.0	0.004091	0.087205	0.201373	Biso	1.000000	O
O475	1.0	0.199552	0.067345	0.814699	Biso	1.000000	O
O476	1.0	0.863876	0.670781	0.851392	Biso	1.000000	O
O477	1.0	0.509165	0.813887	0.802680	Biso	1.000000	O
O478	1.0	0.263290	0.373403	0.543677	Biso	1.000000	O
O479	1.0	0.465435	0.414556	0.731372	Biso	1.000000	O
O480	1.0	0.493591	0.180768	0.720572	Biso	1.000000	O
O481	1.0	0.131800	0.221019	0.188471	Biso	1.000000	O
O482	1.0	0.473965	0.051070	0.844227	Biso	1.000000	O
O483	1.0	0.114179	0.044579	0.175058	Biso	1.000000	O
O484	1.0	0.357910	0.277800	0.209038	Biso	1.000000	O
O485	1.0	0.269349	0.147237	0.052806	Biso	1.000000	O
O486	1.0	0.192806	0.200412	0.995000	Biso	1.000000	O
O487	1.0	0.134576	0.251449	0.391001	Biso	1.000000	O
O488	1.0	0.872956	0.325485	0.890922	Biso	1.000000	O
O489	1.0	0.710673	0.403214	0.297686	Biso	1.000000	O
O490	1.0	0.192034	0.369689	0.563663	Biso	1.000000	O
O491	1.0	0.591141	0.556709	0.622175	Biso	1.000000	O
O492	1.0	0.544698	0.770654	0.114103	Biso	1.000000	O
O493	1.0	0.274510	0.409946	0.330334	Biso	1.000000	O
O494	1.0	0.146476	0.332998	0.089931	Biso	1.000000	O
O495	1.0	0.093899	0.095430	0.640110	Biso	1.000000	O
O496	1.0	0.300118	0.833040	0.128296	Biso	1.000000	O
O497	1.0	0.670649	0.079630	0.540027	Biso	1.000000	O
O498	1.0	0.691056	0.490213	0.317427	Biso	1.000000	O
O499	1.0	0.370100	0.097588	0.723756	Biso	1.000000	O
O500	1.0	0.420953	0.812841	0.554467	Biso	1.000000	O
O501	1.0	0.352012	0.943875	0.392205	Biso	1.000000	O
O502	1.0	0.379018	0.149123	0.402433	Biso	1.000000	O
O503	1.0	0.503768	0.959540	0.805186	Biso	1.000000	O
O504	1.0	0.685459	0.915330	0.315351	Biso	1.000000	O
O505	1.0	0.036311	0.210908	0.578063	Biso	1.000000	O
O506	1.0	0.164438	0.535695	0.023435	Biso	1.000000	O
O507	1.0	0.550903	0.668457	0.022840	Biso	1.000000	O
O508	1.0	0.195674	0.304737	0.196335	Biso	1.000000	O
O509	1.0	0.706324	0.282685	0.060586	Biso	1.000000	O
O510	1.0	0.625252	0.019749	0.141305	Biso	1.000000	O
O511	1.0	0.807027	0.082742	0.579174	Biso	1.000000	O
O512	1.0	0.273796	0.524643	0.238757	Biso	1.000000	O
O513	1.0	0.888403	0.041337	0.065001	Biso	1.000000	O
O514	1.0	0.756111	0.528005	0.323624	Biso	1.000000	O
O515	1.0	0.188779	0.710559	0.994311	Biso	1.000000	O
O516	1.0	0.083289	0.114293	0.168587	Biso	1.000000	O
O517	1.0	0.138674	0.369547	0.435131	Biso	1.000000	O
O518	1.0	0.608560	0.213242	0.153296	Biso	1.000000	O
O519	1.0	0.238001	0.769714	0.396825	Biso	1.000000	O
O520	1.0	0.006732	0.476942	0.681906	Biso	1.000000	O
O521	1.0	0.287307	0.120781	0.126544	Biso	1.000000	O

O522	1.0	0.809558	0.170487	0.201074	Biso	1.000000	O
O523	1.0	0.340801	0.764453	0.320071	Biso	1.000000	O
O524	1.0	0.568125	0.800766	0.342491	Biso	1.000000	O
O525	1.0	0.886090	0.010873	0.907901	Biso	1.000000	O
O526	1.0	0.536840	0.888254	0.387324	Biso	1.000000	O
O527	1.0	0.606327	0.585015	0.883808	Biso	1.000000	O
O528	1.0	0.735058	0.443019	0.176579	Biso	1.000000	O
O529	1.0	0.707928	0.501947	0.033254	Biso	1.000000	O
O530	1.0	0.466200	0.253132	0.641388	Biso	1.000000	O
O531	1.0	0.418364	0.394025	0.059019	Biso	1.000000	O
O532	1.0	0.982487	0.800118	0.695045	Biso	1.000000	O
O533	1.0	0.733388	0.701976	0.702532	Biso	1.000000	O
O534	1.0	0.928276	0.540447	0.021730	Biso	1.000000	O
O535	1.0	0.298855	0.468063	0.123761	Biso	1.000000	O
O536	1.0	0.885942	0.358495	0.258121	Biso	1.000000	O
O537	1.0	0.118727	0.933631	0.669846	Biso	1.000000	O
O538	1.0	0.247343	0.681743	0.225878	Biso	1.000000	O
O539	1.0	0.985365	0.269324	0.037431	Biso	1.000000	O
O540	1.0	0.118489	0.853499	0.558659	Biso	1.000000	O
O541	1.0	0.425336	0.497648	0.783121	Biso	1.000000	O
O542	1.0	0.657078	0.080010	0.239391	Biso	1.000000	O
O543	1.0	0.873871	0.488781	0.008455	Biso	1.000000	O
O544	1.0	0.757234	0.651658	0.356462	Biso	1.000000	O
O545	1.0	0.787911	0.064194	0.375901	Biso	1.000000	O
O546	1.0	0.467216	0.204448	0.332679	Biso	1.000000	O
O547	1.0	0.071171	0.359731	0.325647	Biso	1.000000	O
O548	1.0	0.480280	0.979541	0.874339	Biso	1.000000	O
O549	1.0	0.501124	0.040410	0.546212	Biso	1.000000	O
O550	1.0	0.070954	0.426591	0.155709	Biso	1.000000	O
O551	1.0	0.099197	0.398770	0.220352	Biso	1.000000	O
O552	1.0	0.742190	0.207450	0.197900	Biso	1.000000	O
O553	1.0	0.630730	0.996158	0.619446	Biso	1.000000	O
O554	1.0	0.316355	0.154970	0.269490	Biso	1.000000	O
O555	1.0	0.816472	0.555271	0.700152	Biso	1.000000	O
O556	1.0	0.530620	0.465905	0.307598	Biso	1.000000	O
O557	1.0	0.452397	0.636023	0.385926	Biso	1.000000	O
O558	1.0	0.986029	0.627835	0.409082	Biso	1.000000	O
O559	1.0	0.226862	0.816965	0.656256	Biso	1.000000	O
O560	1.0	0.828129	0.771821	0.036242	Biso	1.000000	O
O561	1.0	0.816266	0.232522	0.336777	Biso	1.000000	O
O562	1.0	0.575578	0.320041	0.745774	Biso	1.000000	O
O563	1.0	0.481259	0.682681	0.754246	Biso	1.000000	O
O564	1.0	0.548534	0.439428	0.670573	Biso	1.000000	O
O565	1.0	0.393380	0.215040	0.927668	Biso	1.000000	O
O566	1.0	0.601315	0.868441	0.157028	Biso	1.000000	O
O567	1.0	0.569515	0.982025	0.834207	Biso	1.000000	O
O568	1.0	0.327671	0.120914	0.919316	Biso	1.000000	O
O569	1.0	0.269384	0.990988	0.449873	Biso	1.000000	O
O570	1.0	0.061187	0.013384	0.423364	Biso	1.000000	O
O571	1.0	0.192138	0.386946	0.063499	Biso	1.000000	O
O572	1.0	0.161632	0.372083	0.358837	Biso	1.000000	O
O573	1.0	0.747480	0.280585	0.659639	Biso	1.000000	O
O574	1.0	0.503421	0.392918	0.950337	Biso	1.000000	O
O575	1.0	0.087219	0.649828	0.045911	Biso	1.000000	O
O576	1.0	0.993056	0.062839	0.972314	Biso	1.000000	O
O577	1.0	0.461515	0.986426	0.004627	Biso	1.000000	O
O578	1.0	0.291706	0.004792	0.084461	Biso	1.000000	O
O579	1.0	0.805478	0.467096	0.193162	Biso	1.000000	O
O580	1.0	0.067287	0.266946	0.419911	Biso	1.000000	O
O581	1.0	0.259696	0.665092	0.051514	Biso	1.000000	O
O582	1.0	0.833963	0.814282	0.290094	Biso	1.000000	O
O583	1.0	0.148149	0.618634	0.315873	Biso	1.000000	O
O584	1.0	0.466987	0.381993	0.437584	Biso	1.000000	O
O585	1.0	0.691424	0.028897	0.821842	Biso	1.000000	O
O586	1.0	0.467257	0.178220	0.208068	Biso	1.000000	O
O587	1.0	0.898042	0.303465	0.522852	Biso	1.000000	O
O588	1.0	0.364075	0.075386	0.969828	Biso	1.000000	O
O589	1.0	0.186266	0.906657	0.052986	Biso	1.000000	O
O590	1.0	0.903219	0.247390	0.581246	Biso	1.000000	O
O591	1.0	0.182396	0.685910	0.543888	Biso	1.000000	O
O592	1.0	0.457123	0.817894	0.700158	Biso	1.000000	O
O593	1.0	0.416146	0.398351	0.269586	Biso	1.000000	O
O594	1.0	0.779830	0.695072	0.492341	Biso	1.000000	O
O595	1.0	0.553897	0.800010	0.488430	Biso	1.000000	O
O596	1.0	0.785960	0.758824	0.717799	Biso	1.000000	O
O597	1.0	0.618245	0.492086	0.475966	Biso	1.000000	O
O598	1.0	0.988915	0.309712	0.722686	Biso	1.000000	O
O599	1.0	0.703912	0.431831	0.995913	Biso	1.000000	O
O600	1.0	0.557449	0.622752	0.602441	Biso	1.000000	O

0601	1.0	0.476522	0.760150	0.745630	Biso	1.000000	0
0602	1.0	0.632169	0.356327	0.108761	Biso	1.000000	0
0603	1.0	0.697979	0.808958	0.051569	Biso	1.000000	0
0604	1.0	0.017640	0.744760	0.947790	Biso	1.000000	0
0605	1.0	0.208300	0.540908	0.480856	Biso	1.000000	0
0606	1.0	0.312307	0.729438	0.243035	Biso	1.000000	0
0607	1.0	0.457441	0.862420	0.163748	Biso	1.000000	0
0608	1.0	0.928598	0.025465	0.530215	Biso	1.000000	0
0609	1.0	0.233757	0.692723	0.390191	Biso	1.000000	0
0610	1.0	0.528087	0.116699	0.418650	Biso	1.000000	0
0611	1.0	0.703220	0.774374	0.345478	Biso	1.000000	0
0612	1.0	0.976899	0.685889	0.189072	Biso	1.000000	0
0613	1.0	0.430221	0.781767	0.986209	Biso	1.000000	0
0614	1.0	0.875186	0.732866	0.147942	Biso	1.000000	0
0615	1.0	0.713119	0.639677	0.415590	Biso	1.000000	0
0616	1.0	0.241799	0.891504	0.005152	Biso	1.000000	0
0617	1.0	0.705270	0.913280	0.790030	Biso	1.000000	0
0618	1.0	0.601220	0.647926	0.739932	Biso	1.000000	0
0619	1.0	0.484241	0.745869	0.076014	Biso	1.000000	0
0620	1.0	0.297703	0.664031	0.280067	Biso	1.000000	0
0621	1.0	0.126267	0.081183	0.925417	Biso	1.000000	0
0622	1.0	0.194749	0.270355	0.889767	Biso	1.000000	0
0623	1.0	0.819243	0.497832	0.126391	Biso	1.000000	0
0624	1.0	0.968138	0.217476	0.606996	Biso	1.000000	0
0625	1.0	0.303714	0.953923	0.684316	Biso	1.000000	0
0626	1.0	0.322702	0.198090	0.699378	Biso	1.000000	0
0627	1.0	0.643493	0.773573	0.760739	Biso	1.000000	0
0628	1.0	0.505045	0.867605	0.223865	Biso	1.000000	0
0629	1.0	0.662797	0.615019	0.248911	Biso	1.000000	0
0630	1.0	0.363916	0.712362	0.779641	Biso	1.000000	0
0631	1.0	0.120405	0.394275	0.831425	Biso	1.000000	0
0632	1.0	0.056537	0.157576	0.302476	Biso	1.000000	0
0633	1.0	0.849109	0.379825	0.938371	Biso	1.000000	0
0634	1.0	0.530907	0.630898	0.728945	Biso	1.000000	0
0635	1.0	0.636107	0.262348	0.498564	Biso	1.000000	0
0636	1.0	0.820951	0.568048	0.481357	Biso	1.000000	0
0637	1.0	0.188573	0.700665	0.918645	Biso	1.000000	0
0638	1.0	0.367005	0.740632	0.387996	Biso	1.000000	0
0639	1.0	0.564112	0.864509	0.781166	Biso	1.000000	0
0640	1.0	0.096558	0.944547	0.598967	Biso	1.000000	0
0641	1.0	0.296600	0.729499	0.370395	Biso	1.000000	0
0642	1.0	0.906442	0.714780	0.453616	Biso	1.000000	0
0643	1.0	0.060792	0.432645	0.750945	Biso	1.000000	0
0644	1.0	0.704207	0.617377	0.823880	Biso	1.000000	0
0645	1.0	0.184260	0.491193	0.142332	Biso	1.000000	0
0646	1.0	0.433513	0.318229	0.067130	Biso	1.000000	0
0647	1.0	0.027779	0.803317	0.486373	Biso	1.000000	0
0648	1.0	0.502341	0.287164	0.076137	Biso	1.000000	0
0649	1.0	0.236838	0.141178	0.984465	Biso	1.000000	0
0650	1.0	0.642847	0.290220	0.757931	Biso	1.000000	0
0651	1.0	0.126682	0.966426	0.891784	Biso	1.000000	0
0652	1.0	0.337964	0.079896	0.168508	Biso	1.000000	0
0653	1.0	0.582304	0.794568	0.662075	Biso	1.000000	0
0654	1.0	0.132559	0.219052	0.825093	Biso	1.000000	0
0655	1.0	0.928158	0.518520	0.405069	Biso	1.000000	0
0656	1.0	0.580581	0.803986	0.417548	Biso	1.000000	0
0657	1.0	0.381945	0.803085	0.618320	Biso	1.000000	0
0658	1.0	0.973871	0.932720	0.029070	Biso	1.000000	0
0659	1.0	0.351158	0.371727	0.803741	Biso	1.000000	0
0660	1.0	0.905252	0.832464	0.110782	Biso	1.000000	0
0661	1.0	0.468823	0.748703	0.671007	Biso	1.000000	0
0662	1.0	0.817584	0.417818	0.373125	Biso	1.000000	0
0663	1.0	0.252439	0.496514	0.014561	Biso	1.000000	0
0664	1.0	0.835495	0.300765	0.265673	Biso	1.000000	0
0665	1.0	0.980420	0.716197	0.443315	Biso	1.000000	0
0666	1.0	0.064425	0.654354	0.260221	Biso	1.000000	0
0667	1.0	0.348445	0.469637	0.864407	Biso	1.000000	0
0668	1.0	0.923183	0.359285	0.322434	Biso	1.000000	0
0669	1.0	0.152556	0.911077	0.735576	Biso	1.000000	0
0670	1.0	0.215314	0.602420	0.434594	Biso	1.000000	0
0671	1.0	0.058544	0.051792	0.007655	Biso	1.000000	0
0672	1.0	0.612425	0.912671	0.299407	Biso	1.000000	0

O673	1.0	0.271450	0.793431	0.068054	Biso	1.000000	O
O674	1.0	0.349085	0.977008	0.121900	Biso	1.000000	O
O675	1.0	0.980440	0.687140	0.914037	Biso	1.000000	O
O676	1.0	0.987545	0.230325	0.873125	Biso	1.000000	O
O677	1.0	0.165000	0.139172	0.957598	Biso	1.000000	O
O678	1.0	0.005719	0.950776	0.497914	Biso	1.000000	O
O679	1.0	0.998634	0.229939	0.157040	Biso	1.000000	O
O680	1.0	0.882520	0.398595	0.872692	Biso	1.000000	O
O681	1.0	0.596564	0.494591	0.327561	Biso	1.000000	O
O682	1.0	0.722052	0.828783	0.292779	Biso	1.000000	O
O683	1.0	0.457024	0.688970	0.624016	Biso	1.000000	O
O684	1.0	0.308187	0.745149	0.822174	Biso	1.000000	O
O685	1.0	0.720494	0.017253	0.068611	Biso	1.000000	O
O686	1.0	0.669187	0.404167	0.062315	Biso	1.000000	O
O687	1.0	0.011444	0.764205	0.292482	Biso	1.000000	O
O688	1.0	0.516485	0.813046	0.057467	Biso	1.000000	O
O689	1.0	0.428035	0.378194	0.947942	Biso	1.000000	O
O690	1.0	0.650517	0.307433	0.899702	Biso	1.000000	O
O691	1.0	0.418075	0.557417	0.127660	Biso	1.000000	O
O692	1.0	0.539961	0.901902	0.283591	Biso	1.000000	O
O693	1.0	0.042329	0.766690	0.816153	Biso	1.000000	O
O694	1.0	0.183763	0.851687	0.161615	Biso	1.000000	O
O695	1.0	0.994757	0.288699	0.426747	Biso	1.000000	O
O696	1.0	0.281816	0.694457	0.600427	Biso	1.000000	O
O697	1.0	0.381931	0.168627	0.662354	Biso	1.000000	O
O698	1.0	0.384473	0.817230	0.885039	Biso	1.000000	O
O699	1.0	0.044642	0.111251	0.585207	Biso	1.000000	O
O700	1.0	0.559398	0.613044	0.305279	Biso	1.000000	O
O701	1.0	0.765869	0.585959	0.434295	Biso	1.000000	O
O702	1.0	0.003485	0.667537	0.714340	Biso	1.000000	O
O703	1.0	0.317522	0.758945	0.965440	Biso	1.000000	O
O704	1.0	0.481166	0.238741	0.400185	Biso	1.000000	O
O705	1.0	0.860190	0.762623	0.709758	Biso	1.000000	O
O706	1.0	0.913437	0.386786	0.804972	Biso	1.000000	O
O707	1.0	0.865011	0.717053	0.073594	Biso	1.000000	O
O708	1.0	0.454265	0.186674	0.784440	Biso	1.000000	O
O709	1.0	0.179474	0.133023	0.550683	Biso	1.000000	O
O710	1.0	0.139151	0.987907	0.493291	Biso	1.000000	O
O711	1.0	0.647382	0.018247	0.358028	Biso	1.000000	O
O712	1.0	0.811851	0.877651	0.914649	Biso	1.000000	O
O713	1.0	0.705855	0.956558	0.023225	Biso	1.000000	O
O714	1.0	0.331139	0.488604	0.188217	Biso	1.000000	O
O715	1.0	0.350038	0.634416	0.778518	Biso	1.000000	O
O716	1.0	0.793448	0.623053	0.089711	Biso	1.000000	O
O717	1.0	0.513337	0.020377	0.088662	Biso	1.000000	O
O718	1.0	0.317186	0.512324	0.417076	Biso	1.000000	O
O719	1.0	0.749689	0.930094	0.720907	Biso	1.000000	O
O720	1.0	0.062429	0.534076	0.420028	Biso	1.000000	O
O721	1.0	0.746802	0.274346	0.246146	Biso	1.000000	O
O722	1.0	0.862657	0.007402	0.498693	Biso	1.000000	O
O723	1.0	0.156281	0.531550	0.781954	Biso	1.000000	O
O724	1.0	0.212843	0.090998	0.236472	Biso	1.000000	O
O725	1.0	0.960812	0.576688	0.903686	Biso	1.000000	O
O726	1.0	0.572149	0.882628	0.040979	Biso	1.000000	O
O727	1.0	0.203848	0.084598	0.317313	Biso	1.000000	O
O728	1.0	0.106360	0.794001	0.605800	Biso	1.000000	O
O729	1.0	0.602656	0.471464	0.967313	Biso	1.000000	O
O730	1.0	0.176864	0.584171	0.885795	Biso	1.000000	O
O731	1.0	0.751719	0.340258	0.711722	Biso	1.000000	O
O732	1.0	0.751747	0.541864	0.495536	Biso	1.000000	O
O733	1.0	0.288043	0.536892	0.067173	Biso	1.000000	O
O734	1.0	0.146603	0.609047	0.462698	Biso	1.000000	O
O735	1.0	0.897582	0.174000	0.492397	Biso	1.000000	O
O736	1.0	0.874989	0.199076	0.395949	Biso	1.000000	O
O737	1.0	0.241893	0.181180	0.545277	Biso	1.000000	O
O738	1.0	0.726522	0.950607	0.950859	Biso	1.000000	O
O739	1.0	0.522029	0.831132	0.730991	Biso	1.000000	O
O740	1.0	0.127668	0.292926	0.454985	Biso	1.000000	O
O741	1.0	0.994566	0.111479	0.912735	Biso	1.000000	O
O742	1.0	0.841865	0.086609	0.710172	Biso	1.000000	O
O743	1.0	0.463608	0.549350	0.995683	Biso	1.000000	O
O744	1.0	0.869149	0.594533	0.739858	Biso	1.000000	O
O745	1.0	0.161655	0.563550	0.129946	Biso	1.000000	O
O746	1.0	0.016338	0.304924	0.133043	Biso	1.000000	O
O747	1.0	0.802591	0.806235	0.879833	Biso	1.000000	O
O748	1.0	0.099176	0.788017	0.204584	Biso	1.000000	O
O749	1.0	0.319844	0.263495	0.360299	Biso	1.000000	O
O750	1.0	0.057172	0.290712	0.294277	Biso	1.000000	O

O751	1.0	0.720546	0.571506	0.273554	Biso	1.000000	O
O752	1.0	0.943139	0.253213	0.700940	Biso	1.000000	O
O753	1.0	0.669323	0.189692	0.189375	Biso	1.000000	O
O754	1.0	0.408222	0.397516	0.342884	Biso	1.000000	O
O755	1.0	0.915418	0.721123	0.209555	Biso	1.000000	O
O756	1.0	0.954379	0.846643	0.789142	Biso	1.000000	O
O757	1.0	0.367350	0.352423	0.232530	Biso	1.000000	O
O758	1.0	0.834676	0.895568	0.983348	Biso	1.000000	O
O759	1.0	0.112288	0.143837	0.582967	Biso	1.000000	O
O760	1.0	0.663066	0.081126	0.165545	Biso	1.000000	O
O761	1.0	0.348231	0.805084	0.075631	Biso	1.000000	O
O762	1.0	0.274145	0.605531	0.099599	Biso	1.000000	O
O763	1.0	0.180926	0.975691	0.697748	Biso	1.000000	O
O764	1.0	0.649434	0.760597	0.251660	Biso	1.000000	O
O765	1.0	0.305436	0.167115	0.831118	Biso	1.000000	O
O766	1.0	0.782060	0.598627	0.308890	Biso	1.000000	O
O767	1.0	0.418147	0.311596	0.564203	Biso	1.000000	O
O768	1.0	0.318938	0.851485	0.743866	Biso	1.000000	O
O769	1.0	0.301803	0.914963	0.619244	Biso	1.000000	O
O770	1.0	0.526801	0.941870	0.922485	Biso	1.000000	O
O771	1.0	0.322650	0.357979	0.037260	Biso	1.000000	O
O772	1.0	0.311873	0.974660	0.920436	Biso	1.000000	O
O773	1.0	0.148896	0.345513	0.508086	Biso	1.000000	O
O774	1.0	0.364784	0.946380	0.641860	Biso	1.000000	O
O775	1.0	0.650236	0.357961	0.313038	Biso	1.000000	O
O776	1.0	0.206826	0.025484	0.476599	Biso	1.000000	O
O777	1.0	0.073152	0.697858	0.712508	Biso	1.000000	O
O778	1.0	0.272844	0.367546	0.393123	Biso	1.000000	O
O779	1.0	0.560588	0.946811	0.342116	Biso	1.000000	O
O780	1.0	0.902834	0.732140	0.827307	Biso	1.000000	O
O781	1.0	0.045237	0.878139	0.074856	Biso	1.000000	O
O782	1.0	0.011802	0.375854	0.754246	Biso	1.000000	O
O783	1.0	0.625747	0.941138	0.149925	Biso	1.000000	O
O784	1.0	0.180598	0.463764	0.051960	Biso	1.000000	O
O785	1.0	0.450434	0.218721	0.466688	Biso	1.000000	O
O786	1.0	0.814813	0.347421	0.768845	Biso	1.000000	O
O787	1.0	0.577613	0.544909	0.975861	Biso	1.000000	O
O788	1.0	0.118761	0.685708	0.901396	Biso	1.000000	O
O789	1.0	0.878232	0.847126	0.783174	Biso	1.000000	O
O790	1.0	0.125735	0.217848	0.113085	Biso	1.000000	O
O791	1.0	0.947826	0.460864	0.638483	Biso	1.000000	O
O792	1.0	0.295146	0.411957	0.992793	Biso	1.000000	O
O793	1.0	0.971375	0.443055	0.498421	Biso	1.000000	O
O794	1.0	0.512411	0.481398	0.535102	Biso	1.000000	O
O795	1.0	0.700863	0.642557	0.040838	Biso	1.000000	O
O796	1.0	0.186815	0.575711	0.615972	Biso	1.000000	O
O797	1.0	0.633087	0.530169	0.927636	Biso	1.000000	O
O798	1.0	0.898572	0.789328	0.501414	Biso	1.000000	O
O799	1.0	0.700023	0.225299	0.638345	Biso	1.000000	O
O800	1.0	0.087584	0.338495	0.040282	Biso	1.000000	O
O801	1.0	0.126429	0.170010	0.512914	Biso	1.000000	O
O802	1.0	0.389566	0.503165	0.231065	Biso	1.000000	O
O803	1.0	0.212349	0.590970	0.993137	Biso	1.000000	O
O804	1.0	0.041255	0.616897	0.322275	Biso	1.000000	O
O805	1.0	0.696474	0.285881	0.409255	Biso	1.000000	O
O806	1.0	0.399721	0.291953	0.757828	Biso	1.000000	O
O807	1.0	0.540856	0.081371	0.595288	Biso	1.000000	O
O808	1.0	0.941700	0.019146	0.630087	Biso	1.000000	O
O809	1.0	0.891907	0.047589	0.835166	Biso	1.000000	O
O810	1.0	0.308419	0.395891	0.099853	Biso	1.000000	O
O811	1.0	0.416751	0.776699	0.075584	Biso	1.000000	O
O812	1.0	0.322230	0.586275	0.447308	Biso	1.000000	O
O813	1.0	0.285521	0.763723	0.896633	Biso	1.000000	O
O814	1.0	0.019792	0.150322	0.161281	Biso	1.000000	O
O815	1.0	0.140962	0.322100	0.959187	Biso	1.000000	O
O816	1.0	0.625761	0.515671	0.854716	Biso	1.000000	O
O817	1.0	0.701619	0.152549	0.892901	Biso	1.000000	O
O818	1.0	0.517539	0.439881	0.438385	Biso	1.000000	O
O819	1.0	0.636101	0.814011	0.368323	Biso	1.000000	O
O820	1.0	0.560186	0.153927	0.124067	Biso	1.000000	O
O821	1.0	0.032003	0.954499	0.249943	Biso	1.000000	O
O822	1.0	0.779451	0.125236	0.726066	Biso	1.000000	O
O823	1.0	0.769453	0.471030	0.519732	Biso	1.000000	O
O824	1.0	0.924156	0.580101	0.279451	Biso	1.000000	O

O825	1.0	0.008532	0.517567	0.908438	Biso	1.000000	O
O826	1.0	0.345116	0.658951	0.433017	Biso	1.000000	O
O827	1.0	0.199732	0.958880	0.881732	Biso	1.000000	O
O828	1.0	0.924182	0.238735	0.000263	Biso	1.000000	O
O829	1.0	0.986016	0.249795	0.946567	Biso	1.000000	O
O830	1.0	0.032636	0.243372	0.088653	Biso	1.000000	O
O831	1.0	0.686403	0.023007	0.421467	Biso	1.000000	O
O832	1.0	0.571324	0.515903	0.688039	Biso	1.000000	O
O833	1.0	0.082860	0.572365	0.534020	Biso	1.000000	O
O834	1.0	0.310779	0.025190	0.983738	Biso	1.000000	O
O835	1.0	0.706396	0.283703	0.513166	Biso	1.000000	O
O836	1.0	0.823496	0.662228	0.323284	Biso	1.000000	O
O837	1.0	0.625938	0.855820	0.659923	Biso	1.000000	O
O838	1.0	0.692961	0.663166	0.647790	Biso	1.000000	O
O839	1.0	0.794736	0.809678	0.191864	Biso	1.000000	O
O840	1.0	0.167522	0.182837	0.721478	Biso	1.000000	O
O841	1.0	0.752049	0.991320	0.845537	Biso	1.000000	O
O842	1.0	0.198215	0.580406	0.740468	Biso	1.000000	O
O843	1.0	0.815433	0.477123	0.053773	Biso	1.000000	O
O844	1.0	0.617582	0.142581	0.468913	Biso	1.000000	O
O845	1.0	0.622003	0.937649	0.666994	Biso	1.000000	O
O846	1.0	0.155995	0.839845	0.496776	Biso	1.000000	O
O847	1.0	0.376745	0.174262	0.099009	Biso	1.000000	O
O848	1.0	0.233159	0.758652	0.838622	Biso	1.000000	O
O849	1.0	0.926255	0.502366	0.282805	Biso	1.000000	O
O850	1.0	0.494161	0.950372	0.120996	Biso	1.000000	O
O851	1.0	0.012760	0.522382	0.775127	Biso	1.000000	O
O852	1.0	0.877088	0.578928	0.532270	Biso	1.000000	O
O853	1.0	0.382695	0.293752	0.278728	Biso	1.000000	O
O854	1.0	0.279696	0.703963	0.739114	Biso	1.000000	O
O855	1.0	0.508902	0.924215	0.473046	Biso	1.000000	O
O856	1.0	0.371751	0.607382	0.160315	Biso	1.000000	O
O857	1.0	0.818519	0.148906	0.828277	Biso	1.000000	O
O858	1.0	0.354783	0.230594	0.146221	Biso	1.000000	O
O859	1.0	0.774387	0.781945	0.320836	Biso	1.000000	O
O860	1.0	0.871123	0.352523	0.638553	Biso	1.000000	O
O861	1.0	0.155762	0.904595	0.369308	Biso	1.000000	O
O862	1.0	0.373995	0.152216	0.968870	Biso	1.000000	O
O863	1.0	0.510815	0.823001	0.878994	Biso	1.000000	O
O864	1.0	0.318356	0.469930	0.040932	Biso	1.000000	O
O865	1.0	0.050894	0.590116	0.609634	Biso	1.000000	O
O866	1.0	0.343074	0.606083	0.377542	Biso	1.000000	O
O867	1.0	0.900240	0.957113	0.144756	Biso	1.000000	O
O868	1.0	0.165253	0.907612	0.925036	Biso	1.000000	O
O869	1.0	0.283709	0.616991	0.998183	Biso	1.000000	O
O870	1.0	0.898366	0.504006	0.736880	Biso	1.000000	O
O871	1.0	0.999698	0.509602	0.376953	Biso	1.000000	O
O872	1.0	0.760703	0.749680	0.849155	Biso	1.000000	O
O873	1.0	0.222487	0.021818	0.064768	Biso	1.000000	O
O874	1.0	0.212886	0.765855	0.710198	Biso	1.000000	O
O875	1.0	0.796653	0.418542	0.812251	Biso	1.000000	O
O876	1.0	0.251935	0.286254	0.149656	Biso	1.000000	O
O877	1.0	0.318874	0.907911	0.881451	Biso	1.000000	O
O878	1.0	0.130742	0.590970	0.246715	Biso	1.000000	O
O879	1.0	0.474953	0.145373	0.464771	Biso	1.000000	O
O880	1.0	0.221318	0.914209	0.826084	Biso	1.000000	O
O881	1.0	0.546435	0.961657	0.419805	Biso	1.000000	O
O882	1.0	0.945032	0.092564	0.649880	Biso	1.000000	O
O883	1.0	0.387328	0.524124	0.972622	Biso	1.000000	O
O884	1.0	0.854379	0.124812	0.532411	Biso	1.000000	O
O885	1.0	0.260599	0.255361	0.080611	Biso	1.000000	O
O886	1.0	0.689805	0.098580	0.783391	Biso	1.000000	O
O887	1.0	0.281348	0.943130	0.253234	Biso	1.000000	O
O888	1.0	0.426636	0.235122	0.573988	Biso	1.000000	O
O889	1.0	0.577906	0.424213	0.351652	Biso	1.000000	O
O890	1.0	0.679336	0.409702	0.137206	Biso	1.000000	O
O891	1.0	0.752599	0.489680	0.093197	Biso	1.000000	O
O892	1.0	0.425753	0.464859	0.308058	Biso	1.000000	O
O893	1.0	0.093076	0.672081	0.327350	Biso	1.000000	O
O894	1.0	0.304197	0.542521	0.138729	Biso	1.000000	O
O895	1.0	0.423747	0.203651	0.716899	Biso	1.000000	O
O896	1.0	0.203295	0.816392	0.444136	Biso	1.000000	O
O897	1.0	0.915293	0.969191	0.055577	Biso	1.000000	O
O898	1.0	0.975156	0.497856	0.551946	Biso	1.000000	O
O899	1.0	0.460655	0.473095	0.662576	Biso	1.000000	O
O900	1.0	0.667293	0.532693	0.435579	Biso	1.000000	O
O901	1.0	0.451433	0.033373	0.490037	Biso	1.000000	O
O902	1.0	0.359911	0.341347	0.873600	Biso	1.000000	O
O903	1.0	0.845400	0.608980	0.202734	Biso	1.000000	O
O904	1.0	0.963198	0.994735	0.951310	Biso	1.000000	O
O905	1.0	0.725173	0.542717	0.820567	Biso	1.000000	O

O906	1.0	0.586485	0.738386	0.581190	Biso	1.000000	O
O907	1.0	0.539816	0.633509	0.089458	Biso	1.000000	O
O908	1.0	0.906795	0.804083	0.276624	Biso	1.000000	O
O909	1.0	0.648621	0.471145	0.796087	Biso	1.000000	O
O910	1.0	0.592748	0.378815	0.651619	Biso	1.000000	O
O911	1.0	0.534268	0.950049	0.055103	Biso	1.000000	O
O912	1.0	0.351732	0.439314	0.534964	Biso	1.000000	O
O913	1.0	0.361834	0.576462	0.636742	Biso	1.000000	O
O914	1.0	0.251543	0.591473	0.689291	Biso	1.000000	O
O915	1.0	0.398580	0.102235	0.075100	Biso	1.000000	O
O916	1.0	0.565681	0.986625	0.656799	Biso	1.000000	O
O917	1.0	0.767846	0.743120	0.580540	Biso	1.000000	O
O918	1.0	0.721343	0.800025	0.601198	Biso	1.000000	O
O919	1.0	0.954819	0.920769	0.450474	Biso	1.000000	O
O920	1.0	0.081984	0.526793	0.800754	Biso	1.000000	O
O921	1.0	0.495027	0.279938	0.461534	Biso	1.000000	O
O922	1.0	0.903729	0.161489	0.005285	Biso	1.000000	O
O923	1.0	0.924660	0.423899	0.281112	Biso	1.000000	O
O924	1.0	0.047932	0.839917	0.230421	Biso	1.000000	O
O925	1.0	0.510147	0.079751	0.482562	Biso	1.000000	O
O926	1.0	0.008426	0.983122	0.825334	Biso	1.000000	O
O927	1.0	0.630530	0.417685	0.917839	Biso	1.000000	O
O928	1.0	0.489331	0.951180	0.368100	Biso	1.000000	O
O929	1.0	0.766013	0.846269	0.471214	Biso	1.000000	O
O930	1.0	0.308262	0.121541	0.992172	Biso	1.000000	O
O931	1.0	0.895407	0.272247	0.939288	Biso	1.000000	O
O932	1.0	0.742054	0.324674	0.459884	Biso	1.000000	O
O933	1.0	0.536397	0.464937	0.189049	Biso	1.000000	O
O934	1.0	0.448477	0.533232	0.433741	Biso	1.000000	O
O935	1.0	0.573678	0.213770	0.363817	Biso	1.000000	O
O936	1.0	0.208860	0.230639	0.177083	Biso	1.000000	O
O937	1.0	0.211145	0.647911	0.105568	Biso	1.000000	O
O938	1.0	0.220637	0.419434	0.004127	Biso	1.000000	O
O939	1.0	0.466967	0.407167	0.806631	Biso	1.000000	O
O940	1.0	0.070969	0.587822	0.003657	Biso	1.000000	O
O941	1.0	0.907447	0.823495	0.379225	Biso	1.000000	O
O942	1.0	0.811437	0.884796	0.318511	Biso	1.000000	O
O943	1.0	0.044679	0.008318	0.729912	Biso	1.000000	O
O944	1.0	0.753936	0.972284	0.122816	Biso	1.000000	O
O945	1.0	0.395930	0.365275	0.410140	Biso	1.000000	O
O946	1.0	0.259856	0.191049	0.236685	Biso	1.000000	O
O947	1.0	0.930506	0.909650	0.589296	Biso	1.000000	O
O948	1.0	0.704361	0.330758	0.768299	Biso	1.000000	O
O949	1.0	0.072236	0.478293	0.915482	Biso	1.000000	O
O950	1.0	0.021176	0.767748	0.404248	Biso	1.000000	O
O951	1.0	0.808692	0.220160	0.262808	Biso	1.000000	O
O952	1.0	0.092163	0.320610	0.643803	Biso	1.000000	O
O953	1.0	0.784116	0.313113	0.829054	Biso	1.000000	O
O954	1.0	0.891102	0.386057	0.002435	Biso	1.000000	O
O955	1.0	0.660029	0.938340	0.247725	Biso	1.000000	O
O956	1.0	0.687640	0.030654	0.742054	Biso	1.000000	O
O957	1.0	0.656592	0.226498	0.037535	Biso	1.000000	O
O958	1.0	0.776613	0.222800	0.989718	Biso	1.000000	O
O959	1.0	0.497543	0.152469	0.008881	Biso	1.000000	O
O960	1.0	0.716168	0.911426	0.653307	Biso	1.000000	O
O961	1.0	0.105989	0.842591	0.044294	Biso	1.000000	O
O962	1.0	0.160108	0.985814	0.577500	Biso	1.000000	O
O963	1.0	0.534966	0.991271	0.979725	Biso	1.000000	O
O964	1.0	0.426929	0.567900	0.670855	Biso	1.000000	O
O965	1.0	0.831641	0.629095	0.683059	Biso	1.000000	O
O966	1.0	0.132567	0.262206	0.008504	Biso	1.000000	O
O967	1.0	0.107118	0.510042	0.364758	Biso	1.000000	O
O968	1.0	0.550584	0.745550	0.042919	Biso	1.000000	O
O969	1.0	0.270208	0.230527	0.492939	Biso	1.000000	O
O970	1.0	0.352427	0.240824	0.069684	Biso	1.000000	O
O971	1.0	0.202312	0.419066	0.316269	Biso	1.000000	O
O972	1.0	0.846680	0.706001	0.999750	Biso	1.000000	O
O973	1.0	0.129644	0.148117	0.327077	Biso	1.000000	O
O974	1.0	0.883362	0.927860	0.423950	Biso	1.000000	O
O975	1.0	0.982086	0.052541	0.728520	Biso	1.000000	O
O976	1.0	0.520593	0.271871	0.146585	Biso	1.000000	O
O977	1.0	0.839737	0.597566	0.035836	Biso	1.000000	O
O978	1.0	0.833216	0.156651	0.682208	Biso	1.000000	O
O979	1.0	0.267578	0.799156	0.317623	Biso	1.000000	O
O980	1.0	0.707983	0.519898	0.607201	Biso	1.000000	O
O981	1.0	0.381844	0.034385	0.304265	Biso	1.000000	O
O982	1.0	0.077339	0.952657	0.469127	Biso	1.000000	O
O983	1.0	0.612862	0.362773	0.864094	Biso	1.000000	O
O984	1.0	0.307043	0.265319	0.014872	Biso	1.000000	O
O985	1.0	0.578265	0.597418	0.035253	Biso	1.000000	O
O986	1.0	0.895419	0.405876	0.076038	Biso	1.000000	O
O987	1.0	0.101038	0.202042	0.044012	Biso	1.000000	O
O988	1.0	0.700047	0.257422	0.793237	Biso	1.000000	O

O989	1.0	0.768961	0.667960	0.560447	Biso	1.000000	O
O990	1.0	0.631167	0.726888	0.946142	Biso	1.000000	O
O991	1.0	0.999415	0.841976	0.848768	Biso	1.000000	O
O992	1.0	0.516137	0.239457	0.285111	Biso	1.000000	O
O993	1.0	0.456523	0.177369	0.529720	Biso	1.000000	O
O994	1.0	0.941700	0.989589	0.199075	Biso	1.000000	O
O995	1.0	0.885473	0.305850	0.006810	Biso	1.000000	O
O996	1.0	0.040073	0.221671	0.264747	Biso	1.000000	O
O997	1.0	0.994766	0.956754	0.100484	Biso	1.000000	O
O998	1.0	0.834094	0.827282	0.490529	Biso	1.000000	O
O999	1.0	0.163452	0.650401	0.609268	Biso	1.000000	O
O1000	1.0	0.832959	0.832401	0.088098	Biso	1.000000	O
O1001	1.0	0.009009	0.525973	0.211342	Biso	1.000000	O
O1002	1.0	0.827226	0.430125	0.994142	Biso	1.000000	O
O1003	1.0	0.770368	0.298638	0.983776	Biso	1.000000	O
O1004	1.0	0.095242	0.867048	0.759487	Biso	1.000000	O
O1005	1.0	0.654565	0.776596	0.000635	Biso	1.000000	O
O1006	1.0	0.180015	0.525119	0.364154	Biso	1.000000	O
O1007	1.0	0.061885	0.987482	0.188362	Biso	1.000000	O
O1008	1.0	0.636764	0.253299	0.212751	Biso	1.000000	O
O1009	1.0	0.032173	0.022692	0.090166	Biso	1.000000	O
O1010	1.0	0.892445	0.934457	0.292935	Biso	1.000000	O
O1011	1.0	0.476797	0.409214	0.313736	Biso	1.000000	O
O1012	1.0	0.042133	0.138749	0.772298	Biso	1.000000	O
O1013	1.0	0.101781	0.675050	0.464419	Biso	1.000000	O
O1014	1.0	0.294596	0.056233	0.779882	Biso	1.000000	O
O1015	1.0	0.622365	0.303598	0.632447	Biso	1.000000	O
O1016	1.0	0.831696	0.098409	0.171011	Biso	1.000000	O
O1017	1.0	0.832322	0.873035	0.629829	Biso	1.000000	O
O1018	1.0	0.688004	0.627006	0.346422	Biso	1.000000	O
O1019	1.0	0.179633	0.553157	0.290145	Biso	1.000000	O
O1020	1.0	0.602375	0.743071	0.377671	Biso	1.000000	O
O1021	1.0	0.016812	0.992480	0.313894	Biso	1.000000	O
O1022	1.0	0.797466	0.032039	0.145826	Biso	1.000000	O
O1023	1.0	0.308439	0.991443	0.617074	Biso	1.000000	O
O1024	1.0	0.055463	0.531010	0.050698	Biso	1.000000	O
O1025	1.0	0.617498	0.571491	0.476151	Biso	1.000000	O
O1026	1.0	0.329266	0.206447	0.213039	Biso	1.000000	O
O1027	1.0	0.822744	0.892942	0.453810	Biso	1.000000	O
O1028	1.0	0.376181	0.774709	0.822256	Biso	1.000000	O
O1029	1.0	0.857972	0.676781	0.258695	Biso	1.000000	O
O1030	1.0	0.854984	0.297281	0.809434	Biso	1.000000	O
O1031	1.0	0.439388	0.538789	0.274714	Biso	1.000000	O
O1032	1.0	0.958125	0.896014	0.520805	Biso	1.000000	O
O1033	1.0	0.247416	0.733008	0.953696	Biso	1.000000	O
O1034	1.0	0.750418	0.253823	0.925305	Biso	1.000000	O
O1035	1.0	0.265697	0.071088	0.182292	Biso	1.000000	O
O1036	1.0	0.460319	0.625312	0.708978	Biso	1.000000	O
O1037	1.0	0.126290	0.462652	0.861740	Biso	1.000000	O
O1038	1.0	0.355655	0.049474	0.235086	Biso	1.000000	O
O1039	1.0	0.585141	0.533482	0.108076	Biso	1.000000	O
O1040	1.0	0.510488	0.337858	0.327033	Biso	1.000000	O
O1041	1.0	0.757214	0.562289	0.650115	Biso	1.000000	O
O1042	1.0	0.575025	0.953538	0.490902	Biso	1.000000	O
O1043	1.0	0.015202	0.478790	0.446282	Biso	1.000000	O
O1044	1.0	0.702725	0.742948	0.202730	Biso	1.000000	O
O1045	1.0	0.281884	0.491245	0.892353	Biso	1.000000	O
O1046	1.0	0.197648	0.837159	0.923353	Biso	1.000000	O
O1047	1.0	0.613409	0.326354	0.214588	Biso	1.000000	O
O1048	1.0	0.804693	0.327801	0.660187	Biso	1.000000	O
O1049	1.0	0.874685	0.462941	0.164743	Biso	1.000000	O
O1050	1.0	0.240772	0.402834	0.610901	Biso	1.000000	O
O1051	1.0	0.850317	0.281560	0.468476	Biso	1.000000	O
O1052	1.0	0.677262	0.565572	0.873662	Biso	1.000000	O
O1053	1.0	0.668217	0.220470	0.381336	Biso	1.000000	O
O1054	1.0	0.134966	0.633898	0.106401	Biso	1.000000	O
O1055	1.0	0.347568	0.294925	0.815332	Biso	1.000000	O
O1056	1.0	0.471947	0.911577	0.021413	Biso	1.000000	O
O1057	1.0	0.287893	0.443815	0.396374	Biso	1.000000	O
O1058	1.0	0.357957	0.034086	0.810565	Biso	1.000000	O
O1059	1.0	0.615529	0.106767	0.724920	Biso	1.000000	O
O1060	1.0	0.554774	0.222522	0.730982	Biso	1.000000	O
O1061	1.0	0.635076	0.924357	0.529582	Biso	1.000000	O
O1062	1.0	0.375379	0.662454	0.028154	Biso	1.000000	O
O1063	1.0	0.896499	0.664616	0.119466	Biso	1.000000	O
O1064	1.0	0.957963	0.265600	0.808528	Biso	1.000000	O
O1065	1.0	0.243896	0.064309	0.723826	Biso	1.000000	O
O1066	1.0	0.467045	0.956595	0.559301	Biso	1.000000	O
O1067	1.0	0.088481	0.212152	0.345932	Biso	1.000000	O

O1068	1.0	0.045990	0.520615	0.974539	Biso	1.000000	O
O1069	1.0	0.222322	0.320873	0.088879	Biso	1.000000	O
O1070	1.0	0.616861	0.923811	0.860133	Biso	1.000000	O
O1071	1.0	0.347313	0.682078	0.566269	Biso	1.000000	O
O1072	1.0	0.586320	0.140237	0.600494	Biso	1.000000	O
O1073	1.0	0.615749	0.818702	0.214061	Biso	1.000000	O
O1074	1.0	0.693381	0.552340	0.365122	Biso	1.000000	O
O1075	1.0	0.400971	0.759985	0.222004	Biso	1.000000	O
O1076	1.0	0.004528	0.750081	0.564692	Biso	1.000000	O
O1077	1.0	0.463272	0.639925	0.240351	Biso	1.000000	O
O1078	1.0	0.313770	0.883415	0.021071	Biso	1.000000	O
O1079	1.0	0.361663	0.542491	0.339791	Biso	1.000000	O
O1080	1.0	0.270443	0.867575	0.797230	Biso	1.000000	O
O1081	1.0	0.400297	0.717436	0.959134	Biso	1.000000	O
O1082	1.0	0.813018	0.020200	0.813256	Biso	1.000000	O
O1083	1.0	0.251924	0.078953	0.023975	Biso	1.000000	O
O1084	1.0	0.121230	0.459637	0.786530	Biso	1.000000	O
O1085	1.0	0.999945	0.567023	0.012124	Biso	1.000000	O
O1086	1.0	0.856568	0.091912	0.390312	Biso	1.000000	O
O1087	1.0	0.427346	0.380570	0.198063	Biso	1.000000	O
O1088	1.0	0.627163	0.075888	0.309395	Biso	1.000000	O
O1089	1.0	0.575447	0.671785	0.145964	Biso	1.000000	O
O1090	1.0	0.020539	0.733189	0.147664	Biso	1.000000	O
O1091	1.0	0.843570	0.167704	0.302881	Biso	1.000000	O
O1092	1.0	0.290360	0.949235	0.989974	Biso	1.000000	O
O1093	1.0	0.783823	0.627690	0.164244	Biso	1.000000	O
O1094	1.0	0.608157	0.993988	0.545157	Biso	1.000000	O
O1095	1.0	0.033915	0.304174	0.363706	Biso	1.000000	O
O1096	1.0	0.238008	0.096915	0.872023	Biso	1.000000	O
O1097	1.0	0.434147	0.035775	0.091393	Biso	1.000000	O
O1098	1.0	0.291315	0.258449	0.854141	Biso	1.000000	O
O1099	1.0	0.078434	0.332046	0.844300	Biso	1.000000	O
O1100	1.0	0.659392	0.863346	0.264749	Biso	1.000000	O
O1101	1.0	0.367963	0.222032	0.998532	Biso	1.000000	O
O1102	1.0	0.095552	0.711198	0.217637	Biso	1.000000	O
O1103	1.0	0.849943	0.240566	0.990704	Biso	1.000000	O
O1104	1.0	0.696450	0.615981	0.112376	Biso	1.000000	O
O1105	1.0	0.594082	0.288740	0.851652	Biso	1.000000	O
O1106	1.0	0.121533	0.473620	0.008044	Biso	1.000000	O
O1107	1.0	0.236984	0.722510	0.506585	Biso	1.000000	O
O1108	1.0	0.892555	0.959926	0.741740	Biso	1.000000	O
O1109	1.0	0.713855	0.192309	0.753683	Biso	1.000000	O
O1110	1.0	0.649515	0.710716	0.531892	Biso	1.000000	O
O1111	1.0	0.412935	0.498791	0.375332	Biso	1.000000	O
O1112	1.0	0.721554	0.036051	0.356635	Biso	1.000000	O
O1113	1.0	0.142348	0.468045	0.935000	Biso	1.000000	O
O1114	1.0	0.624965	0.822739	0.474512	Biso	1.000000	O
O1115	1.0	0.008851	0.596912	0.797303	Biso	1.000000	O
O1116	1.0	0.262352	0.809078	0.954051	Biso	1.000000	O
O1117	1.0	0.779561	0.031029	0.309210	Biso	1.000000	O
O1118	1.0	0.238181	0.623539	0.861848	Biso	1.000000	O
O1119	1.0	0.295111	0.157774	0.498271	Biso	1.000000	O
O1120	1.0	0.827605	0.966634	0.117467	Biso	1.000000	O
O1121	1.0	0.814109	0.343988	0.587508	Biso	1.000000	O
O1122	1.0	0.209494	0.497253	0.877599	Biso	1.000000	O
O1123	1.0	0.984327	0.873840	0.202351	Biso	1.000000	O
O1124	1.0	0.333638	0.779105	0.758848	Biso	1.000000	O
O1125	1.0	0.525162	0.013642	0.232814	Biso	1.000000	O
O1126	1.0	0.537074	0.087245	0.806857	Biso	1.000000	O
O1127	1.0	0.014435	0.878489	0.473905	Biso	1.000000	O
O1128	1.0	0.428142	0.133113	0.136966	Biso	1.000000	O
O1129	1.0	0.248948	0.285541	0.662298	Biso	1.000000	O
O1130	1.0	0.908582	0.951255	0.528251	Biso	1.000000	O
O1131	1.0	0.594395	0.665783	0.884907	Biso	1.000000	O
O1132	1.0	0.519036	0.944984	0.613964	Biso	1.000000	O
O1133	1.0	0.741915	0.469945	0.795254	Biso	1.000000	O
O1134	1.0	0.811567	0.011804	0.612141	Biso	1.000000	O
O1135	1.0	0.357207	0.377160	0.972241	Biso	1.000000	O
O1136	1.0	0.311694	0.307803	0.259261	Biso	1.000000	O
O1137	1.0	0.943075	0.198831	0.430490	Biso	1.000000	O
O1138	1.0	0.014479	0.196689	0.031928	Biso	1.000000	O
O1139	1.0	0.874659	0.250132	0.870264	Biso	1.000000	O
O1140	1.0	0.539501	0.094138	0.078734	Biso	1.000000	O
O1141	1.0	0.022025	0.438437	0.376455	Biso	1.000000	O
O1142	1.0	0.172057	0.021423	0.760018	Biso	1.000000	O
O1143	1.0	0.823082	0.821584	0.740934	Biso	1.000000	O
O1144	1.0	0.827938	0.700330	0.592016	Biso	1.000000	O
O1145	1.0	0.895155	0.606752	0.929787	Biso	1.000000	O
O1146	1.0	0.199566	0.047512	0.665795	Biso	1.000000	O
O1147	1.0	0.309499	0.210831	0.553553	Biso	1.000000	O
O1148	1.0	0.246032	0.001250	0.951290	Biso	1.000000	O
O1149	1.0	0.112586	0.741962	0.661272	Biso	1.000000	O

O1150	1.0	0.640395	0.515225	0.576198	Biso	1.000000	O
O1151	1.0	0.198152	0.858160	0.778451	Biso	1.000000	O
O1152	1.0	0.074199	0.907423	0.930819	Biso	1.000000	O
O1153	1.0	0.677720	0.691120	0.254071	Biso	1.000000	O
O1154	1.0	0.786438	0.302621	0.329071	Biso	1.000000	O
O1155	1.0	0.041115	0.123219	0.038626	Biso	1.000000	O
O1156	1.0	0.698996	0.770220	0.810430	Biso	1.000000	O
O1157	1.0	0.300123	0.197720	0.900769	Biso	1.000000	O
O1158	1.0	0.879610	0.589043	0.138732	Biso	1.000000	O
O1159	1.0	0.677089	0.451997	0.593684	Biso	1.000000	O
O1160	1.0	0.257586	0.749228	0.649564	Biso	1.000000	O
O1161	1.0	0.486517	0.891115	0.698891	Biso	1.000000	O
O1162	1.0	0.988045	0.154815	0.565818	Biso	1.000000	O
O1163	1.0	0.094564	0.498577	0.562280	Biso	1.000000	O
O1164	1.0	0.063337	0.165384	0.635888	Biso	1.000000	O
O1165	1.0	0.956814	0.727816	0.696839	Biso	1.000000	O
O1166	1.0	0.401559	0.370988	0.607075	Biso	1.000000	O
O1167	1.0	0.335810	0.265444	0.913791	Biso	1.000000	O
O1168	1.0	0.172718	0.655804	0.859131	Biso	1.000000	O
O1169	1.0	0.836925	0.420197	0.447161	Biso	1.000000	O
O1170	1.0	0.402367	0.371528	0.749943	Biso	1.000000	O
O1171	1.0	0.832785	0.227533	0.567849	Biso	1.000000	O
O1172	1.0	0.686125	0.114640	0.469382	Biso	1.000000	O
O1173	1.0	0.557852	0.706908	0.436256	Biso	1.000000	O
O1174	1.0	0.804919	0.648384	0.810855	Biso	1.000000	O
O1175	1.0	0.843918	0.145557	0.757732	Biso	1.000000	O
O1176	1.0	0.507225	0.087240	0.353120	Biso	1.000000	O
O1177	1.0	0.924871	0.811137	0.970297	Biso	1.000000	O
O1178	1.0	0.644249	0.421584	0.501396	Biso	1.000000	O
O1179	1.0	0.136984	0.753307	0.029949	Biso	1.000000	O
O1180	1.0	0.691059	0.383075	0.714422	Biso	1.000000	O
O1181	1.0	0.197601	0.036692	0.995437	Biso	1.000000	O
O1182	1.0	0.437092	0.916814	0.450295	Biso	1.000000	O
O1183	1.0	0.378025	0.413528	0.465545	Biso	1.000000	O
O1184	1.0	0.241775	0.206483	0.824885	Biso	1.000000	O
O1185	1.0	0.358084	0.874525	0.357045	Biso	1.000000	O
O1186	1.0	0.867365	0.806811	0.614222	Biso	1.000000	O
O1187	1.0	0.237173	0.802663	0.249453	Biso	1.000000	O
O1188	1.0	0.576000	0.213419	0.844127	Biso	1.000000	O
O1189	1.0	0.247813	0.434632	0.076223	Biso	1.000000	O
O1190	1.0	0.974635	0.464198	0.240151	Biso	1.000000	O
O1191	1.0	0.738426	0.355347	0.640054	Biso	1.000000	O
O1192	1.0	0.791067	0.392150	0.182774	Biso	1.000000	O
O1193	1.0	0.373230	0.822760	0.962444	Biso	1.000000	O
O1194	1.0	0.700828	0.700541	0.370603	Biso	1.000000	O
O1195	1.0	0.921837	0.594741	0.390540	Biso	1.000000	O
O1196	1.0	0.938295	0.628091	0.731580	Biso	1.000000	O
O1197	1.0	0.498744	0.609833	0.854672	Biso	1.000000	O
O1198	1.0	0.064726	0.803543	0.959128	Biso	1.000000	O
O1199	1.0	0.111429	0.602263	0.174308	Biso	1.000000	O
O1200	1.0	0.299440	0.137731	0.198499	Biso	1.000000	O
O1201	1.0	0.529367	0.265166	0.601708	Biso	1.000000	O
O1202	1.0	0.885143	0.350620	0.463592	Biso	1.000000	O
O1203	1.0	0.571420	0.180238	0.291960	Biso	1.000000	O
O1204	1.0	0.938804	0.370907	0.616799	Biso	1.000000	O
O1205	1.0	0.837901	0.356894	0.084012	Biso	1.000000	O
O1206	1.0	0.083455	0.853770	0.490160	Biso	1.000000	O
O1207	1.0	0.180767	0.053261	0.543835	Biso	1.000000	O
O1208	1.0	0.282400	0.189003	0.763524	Biso	1.000000	O
O1209	1.0	0.373360	0.439935	0.770812	Biso	1.000000	O
O1210	1.0	0.859805	0.103688	0.031414	Biso	1.000000	O
O1211	1.0	0.496199	0.388643	0.637199	Biso	1.000000	O
O1212	1.0	0.555981	0.355935	0.385193	Biso	1.000000	O
O1213	1.0	0.009223	0.457325	0.953869	Biso	1.000000	O
O1214	1.0	0.679139	0.757874	0.417304	Biso	1.000000	O
O1215	1.0	0.291043	0.522656	0.314026	Biso	1.000000	O
O1216	1.0	0.419893	0.671602	0.558870	Biso	1.000000	O
O1217	1.0	0.749084	0.677939	0.119225	Biso	1.000000	O
O1218	1.0	0.279978	0.247497	0.297496	Biso	1.000000	O
O1219	1.0	0.081535	0.393470	0.393349	Biso	1.000000	O
O1220	1.0	0.502908	0.683206	0.681455	Biso	1.000000	O
O1221	1.0	0.353816	0.078701	0.651194	Biso	1.000000	O
O1222	1.0	0.677917	0.839344	0.779480	Biso	1.000000	O
O1223	1.0	0.238947	0.012401	0.137834	Biso	1.000000	O
O1224	1.0	0.630736	0.926072	0.022034	Biso	1.000000	O
O1225	1.0	0.458006	0.655777	0.312801	Biso	1.000000	O
O1226	1.0	0.169993	0.406154	0.646820	Biso	1.000000	O
O1227	1.0	0.202181	0.154855	0.349828	Biso	1.000000	O
O1228	1.0	0.590171	0.741826	0.715155	Biso	1.000000	O
O1229	1.0	0.920722	0.703532	0.759455	Biso	1.000000	O
O1230	1.0	0.702870	0.011425	0.897791	Biso	1.000000	O
O1231	1.0	0.944161	0.910178	0.718652	Biso	1.000000	O

O1232	1.0	0.233420	0.317107	0.730355	Biso	1.000000	O
O1233	1.0	0.458330	0.947496	0.744074	Biso	1.000000	O
O1234	1.0	0.425006	0.623084	0.848513	Biso	1.000000	O
O1235	1.0	0.470334	0.616819	0.959952	Biso	1.000000	O
O1236	1.0	0.515964	0.833954	0.656151	Biso	1.000000	O
O1237	1.0	0.143848	0.937300	0.437312	Biso	1.000000	O
O1238	1.0	0.097790	0.871923	0.627428	Biso	1.000000	O
O1239	1.0	0.180517	0.137417	0.841820	Biso	1.000000	O
O1240	1.0	0.178481	0.976061	0.246092	Biso	1.000000	O
O1241	1.0	0.900686	0.506858	0.545485	Biso	1.000000	O
O1242	1.0	0.927848	0.649053	0.234932	Biso	1.000000	O
O1243	1.0	0.182594	0.619442	0.676474	Biso	1.000000	O
O1244	1.0	0.402483	0.434002	0.993407	Biso	1.000000	O
O1245	1.0	0.766448	0.913968	0.004440	Biso	1.000000	O
O1246	1.0	0.098207	0.153236	0.926745	Biso	1.000000	O
O1247	1.0	0.163624	0.839700	0.619575	Biso	1.000000	O
O1248	1.0	0.566773	0.134731	0.356286	Biso	1.000000	O
O1249	1.0	0.474889	0.721913	0.966876	Biso	1.000000	O
O1250	1.0	0.604225	0.442256	0.849211	Biso	1.000000	O
O1251	1.0	0.028916	0.044144	0.484678	Biso	1.000000	O
O1252	1.0	0.766459	0.618757	0.501751	Biso	1.000000	O
O1253	1.0	0.633064	0.078661	0.429107	Biso	1.000000	O
O1254	1.0	0.504393	0.743837	0.397127	Biso	1.000000	O
O1255	1.0	0.906784	0.333336	0.100462	Biso	1.000000	O
O1256	1.0	0.374412	0.882062	0.430517	Biso	1.000000	O
O1257	1.0	0.893598	0.721964	0.901341	Biso	1.000000	O
O1258	1.0	0.919156	0.131910	0.397332	Biso	1.000000	O
O1259	1.0	0.825541	0.289010	0.391985	Biso	1.000000	O
O1260	1.0	0.769462	0.975522	0.257924	Biso	1.000000	O
O1261	1.0	0.209118	0.710725	0.607761	Biso	1.000000	O
O1262	1.0	0.779998	0.854624	0.250844	Biso	1.000000	O
O1263	1.0	0.953976	0.146302	0.198949	Biso	1.000000	O
O1264	1.0	0.949274	0.980300	0.694629	Biso	1.000000	O
O1265	1.0	0.506993	0.019275	0.621208	Biso	1.000000	O
O1266	1.0	0.067819	0.910458	0.198446	Biso	1.000000	O
O1267	1.0	0.616476	0.212451	0.598747	Biso	1.000000	O
O1268	1.0	0.923838	0.876276	0.255275	Biso	1.000000	O
O1269	1.0	0.094643	0.219053	0.889641	Biso	1.000000	O
O1270	1.0	0.932727	0.672256	0.378932	Biso	1.000000	O
O1271	1.0	0.763943	0.438708	0.950862	Biso	1.000000	O
O1272	1.0	0.590457	0.740376	0.206896	Biso	1.000000	O
O1273	1.0	0.903127	0.745993	0.381075	Biso	1.000000	O
O1274	1.0	0.631402	0.798433	0.606081	Biso	1.000000	O
O1275	1.0	0.973219	0.738251	0.801747	Biso	1.000000	O
O1276	1.0	0.198741	0.621676	0.260263	Biso	1.000000	O
O1277	1.0	0.019527	0.867208	0.964461	Biso	1.000000	O
O1278	1.0	0.141874	0.148650	0.077950	Biso	1.000000	O
O1279	1.0	0.340240	0.237560	0.762228	Biso	1.000000	O
O1280	1.0	0.754576	0.628800	0.685043	Biso	1.000000	O
O1281	1.0	0.017305	0.163081	0.426178	Biso	1.000000	O
O1282	1.0	0.108121	0.034702	0.550856	Biso	1.000000	O
O1283	1.0	0.389630	0.605432	0.722780	Biso	1.000000	O
O1284	1.0	0.749964	0.750449	0.396474	Biso	1.000000	O
O1285	1.0	0.555981	0.405934	0.897410	Biso	1.000000	O
O1286	1.0	0.285136	0.509180	0.744871	Biso	1.000000	O
O1287	1.0	0.590090	0.644305	0.813297	Biso	1.000000	O
O1288	1.0	0.872849	0.694035	0.531960	Biso	1.000000	O
O1289	1.0	0.266427	0.126973	0.317881	Biso	1.000000	O
O1290	1.0	0.658193	0.147846	0.406397	Biso	1.000000	O
O1291	1.0	0.332697	0.520416	0.683308	Biso	1.000000	O
O1292	1.0	0.886961	0.123034	0.815953	Biso	1.000000	O
O1293	1.0	0.281580	0.796549	0.705399	Biso	1.000000	O
O1294	1.0	0.567424	0.976821	0.121849	Biso	1.000000	O
O1295	1.0	0.899116	0.543802	0.340735	Biso	1.000000	O
O1296	1.0	0.957031	0.022148	0.091173	Biso	1.000000	O
O1297	1.0	0.796407	0.158437	0.568617	Biso	1.000000	O
O1298	1.0	0.417797	0.849366	0.485250	Biso	1.000000	O
O1299	1.0	0.449667	0.535373	0.199169	Biso	1.000000	O
O1300	1.0	0.816579	0.808147	0.667261	Biso	1.000000	O
O1301	1.0	0.253182	0.509309	0.378647	Biso	1.000000	O
O1302	1.0	0.694099	0.735408	0.878036	Biso	1.000000	O
O1303	1.0	0.435583	0.777790	0.382532	Biso	1.000000	O
O1304	1.0	0.679773	0.548216	0.215209	Biso	1.000000	O
O1305	1.0	0.835938	0.010131	0.263591	Biso	1.000000	O
O1306	1.0	0.656244	0.786732	0.675563	Biso	1.000000	O
O1307	1.0	0.527754	0.883795	0.153432	Biso	1.000000	O
O1308	1.0	0.297185	0.320812	0.097833	Biso	1.000000	O
O1309	1.0	0.414788	0.049725	0.020592	Biso	1.000000	O
O1310	1.0	0.515037	0.684116	0.271411	Biso	1.000000	O
O1311	1.0	0.545474	0.149056	0.484030	Biso	1.000000	O
O1312	1.0	0.906662	0.923241	0.996027	Biso	1.000000	O
O1313	1.0	0.829713	0.004740	0.960846	Biso	1.000000	O
O1314	1.0	0.814020	0.369291	0.872319	Biso	1.000000	O

O1315	1.0	0.271358	0.912008	0.471299	Biso	1.000000	O
O1316	1.0	0.752978	0.911101	0.586408	Biso	1.000000	O
O1317	1.0	0.329335	0.652524	0.708436	Biso	1.000000	O
O1318	1.0	0.757168	0.025028	0.565196	Biso	1.000000	O
O1319	1.0	0.739604	0.860361	0.184135	Biso	1.000000	O
O1320	1.0	0.996643	0.012273	0.154919	Biso	1.000000	O
O1321	1.0	0.431437	0.719284	0.028861	Biso	1.000000	O
O1322	1.0	0.633003	0.338033	0.701037	Biso	1.000000	O
O1323	1.0	0.906595	0.052549	0.722888	Biso	1.000000	O
O1324	1.0	0.446873	0.713559	0.262806	Biso	1.000000	O
O1325	1.0	0.794362	0.964174	0.185252	Biso	1.000000	O
O1326	1.0	0.467653	0.784948	0.243313	Biso	1.000000	O
O1327	1.0	0.049803	0.729652	0.463750	Biso	1.000000	O
O1328	1.0	0.679854	0.899831	0.585928	Biso	1.000000	O
O1329	1.0	0.843240	0.490274	0.785997	Biso	1.000000	O
O1330	1.0	0.194096	0.778900	0.306179	Biso	1.000000	O
O1331	1.0	0.900298	0.530456	0.952067	Biso	1.000000	O
O1332	1.0	0.616591	0.713279	0.018432	Biso	1.000000	O
O1333	1.0	0.768718	0.102411	0.130412	Biso	1.000000	O
O1334	1.0	0.685983	0.705567	0.991074	Biso	1.000000	O
O1335	1.0	0.437193	0.849360	0.413069	Biso	1.000000	O
O1336	1.0	0.506756	0.749176	0.846669	Biso	1.000000	O
O1337	1.0	0.033545	0.196309	0.922520	Biso	1.000000	O
O1338	1.0	0.467445	0.082477	0.601253	Biso	1.000000	O
O1339	1.0	0.706608	0.392511	0.930171	Biso	1.000000	O
O1340	1.0	0.184331	0.974973	0.386357	Biso	1.000000	O
O1341	1.0	0.006386	0.017315	0.666704	Biso	1.000000	O
O1342	1.0	0.347991	0.805632	0.551328	Biso	1.000000	O
O1343	1.0	0.507845	0.640054	0.158055	Biso	1.000000	O
O1344	1.0	0.579938	0.856451	0.856885	Biso	1.000000	O
O1345	1.0	0.230129	0.328606	0.926510	Biso	1.000000	O
O1346	1.0	0.386216	0.026445	0.696039	Biso	1.000000	O
O1347	1.0	0.848012	0.283441	0.616814	Biso	1.000000	O
O1348	1.0	0.527522	0.287997	0.220788	Biso	1.000000	O
O1349	1.0	0.167663	0.652698	0.402723	Biso	1.000000	O
O1350	1.0	0.891672	0.936425	0.217772	Biso	1.000000	O
O1351	1.0	0.898728	0.757476	0.018492	Biso	1.000000	O
O1352	1.0	0.639179	0.849128	0.028732	Biso	1.000000	O
O1353	1.0	0.944285	0.117711	0.718602	Biso	1.000000	O
O1354	1.0	0.860303	0.403392	0.312751	Biso	1.000000	O
O1355	1.0	0.882644	0.573224	0.669105	Biso	1.000000	O
O1356	1.0	0.589175	0.289035	0.969547	Biso	1.000000	O
O1357	1.0	0.797342	0.402424	0.541079	Biso	1.000000	O
O1358	1.0	0.666558	0.062670	0.092876	Biso	1.000000	O
O1359	1.0	0.136144	0.721639	0.592755	Biso	1.000000	O
O1360	1.0	0.566243	0.868151	0.456017	Biso	1.000000	O
O1361	1.0	0.435207	0.633123	0.130068	Biso	1.000000	O
O1362	1.0	0.991192	0.591419	0.688535	Biso	1.000000	O
O1363	1.0	0.308207	0.120522	0.409674	Biso	1.000000	O
O1364	1.0	0.980142	0.146691	0.076556	Biso	1.000000	O
O1365	1.0	0.690891	0.426621	0.416047	Biso	1.000000	O
O1366	1.0	0.766514	0.795068	0.077477	Biso	1.000000	O
O1367	1.0	0.069814	0.414815	0.036692	Biso	1.000000	O
O1368	1.0	0.102372	0.113348	0.989795	Biso	1.000000	O
O1369	1.0	0.238223	0.313324	0.286979	Biso	1.000000	O
O1370	1.0	0.262663	0.068466	0.449867	Biso	1.000000	O
O1371	1.0	0.815114	0.222236	0.054246	Biso	1.000000	O
O1372	1.0	0.936604	0.975356	0.841636	Biso	1.000000	O
O1373	1.0	0.883273	0.865209	0.934352	Biso	1.000000	O
O1374	1.0	0.528359	0.341645	0.586628	Biso	1.000000	O
O1375	1.0	0.018405	0.701509	0.849782	Biso	1.000000	O
O1376	1.0	0.620382	0.668596	0.212044	Biso	1.000000	O
O1377	1.0	0.315669	0.048559	0.709336	Biso	1.000000	O
O1378	1.0	0.053459	0.283291	0.220011	Biso	1.000000	O
O1379	1.0	0.593842	0.040119	0.250134	Biso	1.000000	O
O1380	1.0	0.422360	0.914812	0.603431	Biso	1.000000	O
O1381	1.0	0.021377	0.690360	0.309831	Biso	1.000000	O
O1382	1.0	0.236035	0.519693	0.944222	Biso	1.000000	O
O1383	1.0	0.854182	0.948403	0.632297	Biso	1.000000	O
O1384	1.0	0.746463	0.591413	0.878554	Biso	1.000000	O

O1385	1.0	0.767018	0.766020	0.465653	Biso	1.000000	O
O1386	1.0	0.079760	0.751251	0.905184	Biso	1.000000	O
O1387	1.0	0.771066	0.175325	0.313915	Biso	1.000000	O
O1388	1.0	0.211073	0.582133	0.544603	Biso	1.000000	O
O1389	1.0	0.588587	0.212588	0.467860	Biso	1.000000	O
O1390	1.0	0.465215	0.022849	0.353352	Biso	1.000000	O
O1391	1.0	0.328183	0.369472	0.505395	Biso	1.000000	O
O1392	1.0	0.598808	0.472821	0.231893	Biso	1.000000	O
O1393	1.0	0.716105	0.694979	0.817800	Biso	1.000000	O
O1394	1.0	0.999550	0.748598	0.639570	Biso	1.000000	O
O1395	1.0	0.085757	0.145902	0.451177	Biso	1.000000	O
O1396	1.0	0.881263	0.055019	0.654412	Biso	1.000000	O
O1397	1.0	0.255995	0.662765	0.799203	Biso	1.000000	O
O1398	1.0	0.249048	0.175415	0.620457	Biso	1.000000	O
O1399	1.0	0.206693	0.548735	0.188213	Biso	1.000000	O
O1400	1.0	0.478806	0.155595	0.933698	Biso	1.000000	O
O1401	1.0	0.401400	0.378887	0.531687	Biso	1.000000	O
O1402	1.0	0.904163	0.153118	0.663605	Biso	1.000000	O
O1403	1.0	0.341574	0.890259	0.807039	Biso	1.000000	O
O1404	1.0	0.614362	0.069477	0.595511	Biso	1.000000	O
O1405	1.0	0.629754	0.622722	0.624617	Biso	1.000000	O
O1406	1.0	0.271181	0.101189	0.595719	Biso	1.000000	O
O1407	1.0	0.631572	0.009840	0.692961	Biso	1.000000	O
O1408	1.0	0.562250	0.008325	0.910243	Biso	1.000000	O
O1409	1.0	0.466854	0.253941	0.195863	Biso	1.000000	O
O1410	1.0	0.728572	0.484440	0.386114	Biso	1.000000	O
O1411	1.0	0.109092	0.247368	0.256733	Biso	1.000000	O
O1412	1.0	0.151585	0.539455	0.530327	Biso	1.000000	O
O1413	1.0	0.010622	0.302184	0.851482	Biso	1.000000	O
O1414	1.0	0.788464	0.101634	0.235081	Biso	1.000000	O
O1415	1.0	0.160671	0.462365	0.493681	Biso	1.000000	O
O1416	1.0	0.760981	0.425168	0.243279	Biso	1.000000	O
O1417	1.0	0.256797	0.236661	0.392202	Biso	1.000000	O
O1418	1.0	0.253359	0.760753	0.574618	Biso	1.000000	O
O1419	1.0	0.707801	0.520694	0.749090	Biso	1.000000	O
O1420	1.0	0.637175	0.566739	0.152086	Biso	1.000000	O
O1421	1.0	0.070092	0.142095	0.378313	Biso	1.000000	O
O1422	1.0	0.864791	0.116955	0.957129	Biso	1.000000	O
O1423	1.0	0.705997	0.348235	0.098701	Biso	1.000000	O
O1424	1.0	0.572508	0.969531	0.270335	Biso	1.000000	O
O1425	1.0	0.947349	0.227263	0.212983	Biso	1.000000	O
O1426	1.0	0.371562	0.429265	0.218835	Biso	1.000000	O
O1427	1.0	0.114098	0.216878	0.454382	Biso	1.000000	O
O1428	1.0	0.292838	0.339945	0.838959	Biso	1.000000	O
O1429	1.0	0.250131	0.733463	0.283611	Biso	1.000000	O
O1430	1.0	0.445784	0.559748	0.745419	Biso	1.000000	O
O1431	1.0	0.384262	0.495215	0.445309	Biso	1.000000	O
O1432	1.0	0.159298	0.155711	0.446874	Biso	1.000000	O
O1433	1.0	0.936992	0.896928	0.382385	Biso	1.000000	O
O1434	1.0	0.247735	0.886750	0.142247	Biso	1.000000	O
O1435	1.0	0.912024	0.574964	0.205095	Biso	1.000000	O
O1436	1.0	0.526289	0.348043	0.475960	Biso	1.000000	O
O1437	1.0	0.398765	0.234447	0.811861	Biso	1.000000	O
O1438	1.0	0.968821	0.708349	0.260765	Biso	1.000000	O
O1439	1.0	0.818785	0.468455	0.315193	Biso	1.000000	O
O1440	1.0	0.538386	0.683604	0.561198	Biso	1.000000	O
O1441	1.0	0.727846	0.093911	0.060514	Biso	1.000000	O
O1442	1.0	0.541811	0.016246	0.772078	Biso	1.000000	O
O1443	1.0	0.401933	0.217313	0.202602	Biso	1.000000	O
O1444	1.0	0.919399	0.055089	0.410929	Biso	1.000000	O
O1445	1.0	0.914251	0.160789	0.287327	Biso	1.000000	O
O1446	1.0	0.694056	0.096778	0.610546	Biso	1.000000	O
O1447	1.0	0.543647	0.486137	0.380377	Biso	1.000000	O
O1448	1.0	0.334029	0.142125	0.343103	Biso	1.000000	O
O1449	1.0	0.757159	0.953887	0.526554	Biso	1.000000	O
O1450	1.0	0.239144	0.950496	0.092695	Biso	1.000000	O
O1451	1.0	0.212577	0.833565	0.044465	Biso	1.000000	O
O1452	1.0	0.383049	0.872719	0.652278	Biso	1.000000	O
O1453	1.0	0.358406	0.264379	0.576925	Biso	1.000000	O
O1454	1.0	0.198019	0.270690	0.965457	Biso	1.000000	O
O1455	1.0	0.872368	0.996312	0.572924	Biso	1.000000	O
O1456	1.0	0.204183	0.876538	0.314572	Biso	1.000000	O
O1457	1.0	0.997155	0.190533	0.807496	Biso	1.000000	O
O1458	1.0	0.287965	0.952051	0.328039	Biso	1.000000	O
O1459	1.0	0.747511	0.101522	0.324489	Biso	1.000000	O

O1460	1.0	0.693040	0.358887	0.505887	Biso	1.000000	O
O1461	1.0	0.436252	0.194839	0.984987	Biso	1.000000	O
O1462	1.0	0.810276	0.716532	0.427040	Biso	1.000000	O
O1463	1.0	0.578508	0.447988	0.524015	Biso	1.000000	O
O1464	1.0	0.478989	0.063069	0.763688	Biso	1.000000	O
O1465	1.0	0.292178	0.336080	0.972502	Biso	1.000000	O
O1466	1.0	0.754521	0.924324	0.346234	Biso	1.000000	O
O1467	1.0	0.609173	0.401966	0.208352	Biso	1.000000	O
O1468	1.0	0.523943	0.757844	0.545025	Biso	1.000000	O
O1469	1.0	0.589041	0.425060	0.452749	Biso	1.000000	O
O1470	1.0	0.352085	0.812132	0.689698	Biso	1.000000	O
O1471	1.0	0.765075	0.687116	0.632681	Biso	1.000000	O
O1472	1.0	0.484536	0.545084	0.498667	Biso	1.000000	O
O1473	1.0	0.112984	0.917031	0.071080	Biso	1.000000	O
O1474	1.0	0.651157	0.548379	0.286050	Biso	1.000000	O
O1475	1.0	0.764982	0.600026	0.024489	Biso	1.000000	O
O1476	1.0	0.584918	0.892285	0.712637	Biso	1.000000	O
O1477	1.0	0.110347	0.530757	0.204383	Biso	1.000000	O
O1478	1.0	0.911338	0.403492	0.679462	Biso	1.000000	O
O1479	1.0	0.520735	0.827731	0.287093	Biso	1.000000	O
O1480	1.0	0.570624	0.501001	0.761979	Biso	1.000000	O
O1481	1.0	0.304487	0.195215	0.103438	Biso	1.000000	O
O1482	1.0	0.951393	0.725820	0.512351	Biso	1.000000	O
O1483	1.0	0.311853	0.261719	0.436447	Biso	1.000000	O
O1484	1.0	0.182079	0.086126	0.051418	Biso	1.000000	O
O1485	1.0	0.486491	0.631935	0.579039	Biso	1.000000	O
O1486	1.0	0.937837	0.290039	0.878824	Biso	1.000000	O
O1487	1.0	0.324462	0.083115	0.295042	Biso	1.000000	O
O1488	1.0	0.332515	0.211833	0.625834	Biso	1.000000	O
O1489	1.0	0.576050	0.780329	0.270990	Biso	1.000000	O
O1490	1.0	0.418552	0.543401	0.597897	Biso	1.000000	O
O1491	1.0	0.920421	0.059082	0.953770	Biso	1.000000	O
O1492	1.0	0.360557	0.359381	0.669556	Biso	1.000000	O
O1493	1.0	0.666410	0.367353	0.636956	Biso	1.000000	O
O1494	1.0	0.137569	0.262678	0.617813	Biso	1.000000	O
O1495	1.0	0.077139	0.995658	0.796072	Biso	1.000000	O
O1496	1.0	0.145412	0.494603	0.426366	Biso	1.000000	O
O1497	1.0	0.596153	0.532590	0.415214	Biso	1.000000	O
O1498	1.0	0.334382	0.789042	0.202015	Biso	1.000000	O
O1499	1.0	0.989386	0.451316	0.753103	Biso	1.000000	O
O1500	1.0	0.702157	0.497778	0.535049	Biso	1.000000	O
O1501	1.0	0.153694	0.439489	0.713282	Biso	1.000000	O
O1502	1.0	0.987594	0.286803	0.650159	Biso	1.000000	O
O1503	1.0	0.092380	0.376913	0.974727	Biso	1.000000	O
O1504	1.0	0.976422	0.222408	0.531807	Biso	1.000000	O
O1505	1.0	0.787150	0.543546	0.255943	Biso	1.000000	O
O1506	1.0	0.710904	0.151041	0.276615	Biso	1.000000	O
O1507	1.0	0.158864	0.203581	0.574627	Biso	1.000000	O
O1508	1.0	0.946813	0.850870	0.321833	Biso	1.000000	O
O1509	1.0	0.020409	0.430046	0.100459	Biso	1.000000	O
O1510	1.0	0.362786	0.032192	0.071584	Biso	1.000000	O
O1511	1.0	0.147803	0.926522	0.295379	Biso	1.000000	O
O1512	1.0	0.725883	0.130155	0.550935	Biso	1.000000	O
O1513	1.0	0.950583	0.868299	0.654717	Biso	1.000000	O
O1514	1.0	0.150975	0.043827	0.357687	Biso	1.000000	O
O1515	1.0	0.467821	0.431174	0.495542	Biso	1.000000	O
O1516	1.0	0.351697	0.985772	0.555734	Biso	1.000000	O
O1517	1.0	0.533261	0.152992	0.231774	Biso	1.000000	O
O1518	1.0	0.198442	0.778833	0.183756	Biso	1.000000	O
O1519	1.0	0.391958	0.898933	0.161407	Biso	1.000000	O
O1520	1.0	0.949436	0.656018	0.478223	Biso	1.000000	O
O1521	1.0	0.717964	0.113608	0.209492	Biso	1.000000	O
O1522	1.0	0.680656	0.468485	0.699349	Biso	1.000000	O
O1523	1.0	0.455243	0.558246	0.820259	Biso	1.000000	O
O1524	1.0	0.903821	0.285862	0.240180	Biso	1.000000	O
O1525	1.0	0.153522	0.753207	0.242964	Biso	1.000000	O
O1526	1.0	0.174533	0.980955	0.948042	Biso	1.000000	O
O1527	1.0	0.400201	0.834837	0.031458	Biso	1.000000	O
O1528	1.0	0.290204	0.883283	0.949267	Biso	1.000000	O
O1529	1.0	0.856032	0.596089	0.830121	Biso	1.000000	O
O1530	1.0	0.809726	0.840957	0.812998	Biso	1.000000	O
O1531	1.0	0.720627	0.113677	0.678729	Biso	1.000000	O
O1532	1.0	0.123258	0.845962	0.935809	Biso	1.000000	O
O1533	1.0	0.789061	0.124361	0.928685	Biso	1.000000	O
O1534	1.0	0.263544	0.364033	0.468414	Biso	1.000000	O
O1535	1.0	0.944155	0.027019	0.017569	Biso	1.000000	O
O1536	1.0	0.369906	0.060115	0.576392	Biso	1.000000	O
O1537	1.0	0.047198	0.922883	0.651932	Biso	1.000000	O
O1538	1.0	0.292711	0.499662	0.819178	Biso	1.000000	O
O1539	1.0	0.603165	0.090389	0.119671	Biso	1.000000	O
O1540	1.0	0.886379	0.877844	0.537010	Biso	1.000000	O
O1541	1.0	0.640036	0.628251	0.431147	Biso	1.000000	O
O1542	1.0	0.038276	0.871594	0.709764	Biso	1.000000	O
O1543	1.0	0.434246	0.342432	0.668381	Biso	1.000000	O

O1544	1.0	0.656618	0.150211	0.568782	Biso	1.000000	O
O1545	1.0	0.747957	0.664514	0.906512	Biso	1.000000	O
O1546	1.0	0.278104	0.914236	0.545881	Biso	1.000000	O
O1547	1.0	0.977183	0.822320	0.258794	Biso	1.000000	O
O1548	1.0	0.122539	0.531562	0.896243	Biso	1.000000	O
O1549	1.0	0.057792	0.933652	0.752077	Biso	1.000000	O
O1550	1.0	0.695729	0.836544	0.980423	Biso	1.000000	O
O1551	1.0	0.216941	0.183174	0.924511	Biso	1.000000	O
O1552	1.0	0.124372	0.400844	0.554901	Biso	1.000000	O
O1553	1.0	0.776550	0.607024	0.947068	Biso	1.000000	O
O1554	1.0	0.567288	0.379858	0.084130	Biso	1.000000	O
O1555	1.0	0.309745	0.751959	0.121668	Biso	1.000000	O
O1556	1.0	0.035017	0.657824	0.149899	Biso	1.000000	O
O1557	1.0	0.510300	0.362785	0.763389	Biso	1.000000	O
O1558	1.0	0.696734	0.593415	0.620885	Biso	1.000000	O
O1559	1.0	0.832353	0.339327	0.515576	Biso	1.000000	O
O1560	1.0	0.049844	0.675433	0.518009	Biso	1.000000	O
O1561	1.0	0.155088	0.564620	0.954409	Biso	1.000000	O
O1562	1.0	0.992337	0.631399	0.863369	Biso	1.000000	O
O1563	1.0	0.866100	0.789982	0.432733	Biso	1.000000	O
O1564	1.0	0.428933	0.930869	0.676293	Biso	1.000000	O
O1565	1.0	0.911376	0.458392	0.834322	Biso	1.000000	O
O1566	1.0	0.636225	0.292500	0.278221	Biso	1.000000	O
O1567	1.0	0.670545	0.856855	0.523244	Biso	1.000000	O
O1568	1.0	0.289527	0.784718	0.510525	Biso	1.000000	O
O1569	1.0	0.960335	0.029717	0.352921	Biso	1.000000	O
O1570	1.0	0.419957	0.649527	0.453567	Biso	1.000000	O
O1571	1.0	0.039265	0.096474	0.110584	Biso	1.000000	O
O1572	1.0	0.483031	0.812464	0.508681	Biso	1.000000	O
O1573	1.0	0.601807	0.714063	0.782133	Biso	1.000000	O
O1574	1.0	0.483236	0.408789	0.879633	Biso	1.000000	O
O1575	1.0	0.433606	0.356698	0.853344	Biso	1.000000	O
O1576	1.0	0.571258	0.064071	0.385648	Biso	1.000000	O
O1577	1.0	0.743831	0.127047	0.988815	Biso	1.000000	O
O1578	1.0	0.627429	0.051561	0.782540	Biso	1.000000	O
O1579	1.0	0.446435	0.456857	0.428315	Biso	1.000000	O
O1580	1.0	0.723896	0.084361	0.928586	Biso	1.000000	O
O1581	1.0	0.768828	0.990677	0.999458	Biso	1.000000	O
O1582	1.0	0.580862	0.573399	0.738431	Biso	1.000000	O
O1583	1.0	0.917265	0.779385	0.770073	Biso	1.000000	O
O1584	1.0	0.220281	0.381709	0.773072	Biso	1.000000	O
O1585	1.0	0.019942	0.944535	0.970250	Biso	1.000000	O
O1586	1.0	0.751886	0.800489	0.532789	Biso	1.000000	O
O1587	1.0	0.426260	0.157534	0.598803	Biso	1.000000	O
O1588	1.0	0.815902	0.948093	0.570133	Biso	1.000000	O
O1589	1.0	0.566923	0.941722	0.769534	Biso	1.000000	O
O1590	1.0	0.860578	0.553368	0.406250	Biso	1.000000	O
O1591	1.0	0.038344	0.823320	0.126734	Biso	1.000000	O
O1592	1.0	0.713942	0.821056	0.124396	Biso	1.000000	O
O1593	1.0	0.359321	0.516331	0.758013	Biso	1.000000	O
O1594	1.0	0.932179	0.776762	0.642303	Biso	1.000000	O
O1595	1.0	0.860112	0.535786	0.278150	Biso	1.000000	O
O1596	1.0	0.906636	0.801825	0.897679	Biso	1.000000	O
O1597	1.0	0.789003	0.016881	0.495689	Biso	1.000000	O
O1598	1.0	0.537798	0.281749	0.370422	Biso	1.000000	O
O1599	1.0	0.385243	0.962269	0.791039	Biso	1.000000	O
O1600	1.0	0.000232	0.338950	0.305481	Biso	1.000000	O
O1601	1.0	0.285013	0.431581	0.499274	Biso	1.000000	O
O1602	1.0	0.832223	0.477406	0.712998	Biso	1.000000	O
O1603	1.0	0.226504	0.650513	0.731217	Biso	1.000000	O
O1604	1.0	0.528970	0.419377	0.575524	Biso	1.000000	O
O1605	1.0	0.629210	0.899436	0.772430	Biso	1.000000	O
O1606	1.0	0.973173	0.683037	0.114041	Biso	1.000000	O
O1607	1.0	0.366466	0.503057	0.124292	Biso	1.000000	O
O1608	1.0	0.676854	0.055906	0.018881	Biso	1.000000	O
O1609	1.0	0.063326	0.578328	0.684659	Biso	1.000000	O
O1610	1.0	0.546620	0.265534	0.797212	Biso	1.000000	O
O1611	1.0	0.129151	0.910944	0.505990	Biso	1.000000	O
O1612	1.0	0.072756	0.252825	0.153805	Biso	1.000000	O
O1613	1.0	0.959863	0.181166	0.923470	Biso	1.000000	O
O1614	1.0	0.558741	0.152248	0.699102	Biso	1.000000	O
O1615	1.0	0.409397	0.754908	0.715003	Biso	1.000000	O
O1616	1.0	0.313787	0.858368	0.507118	Biso	1.000000	O
O1617	1.0	0.843023	0.459516	0.520342	Biso	1.000000	O
O1618	1.0	0.831462	0.736888	0.302072	Biso	1.000000	O
O1619	1.0	0.178912	0.201281	0.646222	Biso	1.000000	O
O1620	1.0	0.523208	0.440677	0.771770	Biso	1.000000	O
O1621	1.0	0.235529	0.847225	0.383344	Biso	1.000000	O
O1622	1.0	0.163194	0.693746	0.142037	Biso	1.000000	O
O1623	1.0	0.675369	0.651863	0.574474	Biso	1.000000	O
H1	1.0	0.072547	0.287950	0.552231	Biso	1.000000	H
H2	1.0	0.384158	0.209184	0.820822	Biso	1.000000	H

H3	1.0	0.428411	0.290571	0.766954	Biso	1.000000	H
H4	1.0	0.423993	0.346963	0.946467	Biso	1.000000	H
H5	1.0	0.166433	0.890931	0.716465	Biso	1.000000	H
H6	1.0	0.184401	0.996128	0.721038	Biso	1.000000	H
H7	1.0	0.205142	0.583538	0.574946	Biso	1.000000	H
H8	1.0	0.244292	0.508731	0.556554	Biso	1.000000	H
H9	1.0	0.225585	0.516792	0.470680	Biso	1.000000	H
H10	1.0	0.371339	0.003980	0.152929	Biso	1.000000	H
H11	1.0	0.413083	0.110171	0.173757	Biso	1.000000	H
H12	1.0	0.203693	0.183618	0.642080	Biso	1.000000	H
H13	1.0	0.307721	0.518369	0.666156	Biso	1.000000	H
H14	1.0	0.034706	0.997554	0.178939	Biso	1.000000	H
H15	1.0	0.048371	0.906280	0.174840	Biso	1.000000	H
H16	1.0	0.154089	0.068973	0.921295	Biso	1.000000	H
H17	1.0	0.060180	0.269619	0.192686	Biso	1.000000	H
H18	1.0	0.118516	0.237606	0.228768	Biso	1.000000	H
H19	1.0	0.130254	0.984814	0.867057	Biso	1.000000	H
H20	1.0	0.863181	0.434062	0.439103	Biso	1.000000	H
H21	1.0	0.775675	0.348030	0.435828	Biso	1.000000	H
H22	1.0	0.895300	0.401809	0.045156	Biso	1.000000	H
H23	1.0	0.831612	0.355604	0.054004	Biso	1.000000	H
H24	1.0	0.894484	0.304412	0.099032	Biso	1.000000	H
H25	1.0	0.875918	0.415985	0.152600	Biso	1.000000	H
H26	1.0	0.257297	0.222590	0.231873	Biso	1.000000	H
H27	1.0	0.214454	0.948678	0.708287	Biso	1.000000	H
H28	1.0	0.603484	0.606140	0.050160	Biso	1.000000	H
H29	1.0	0.414652	0.629373	0.476916	Biso	1.000000	H
H30	1.0	0.974870	0.185550	0.646966	Biso	1.000000	H
H31	1.0	0.326918	0.637024	0.817624	Biso	1.000000	H
H32	1.0	0.463710	0.599001	0.935589	Biso	1.000000	H
H33	1.0	0.436791	0.538798	0.985852	Biso	1.000000	H
H34	1.0	0.365566	0.469094	0.052636	Biso	1.000000	H
H35	1.0	0.484994	0.427984	0.037934	Biso	1.000000	H
H36	1.0	0.350020	0.554056	0.655098	Biso	1.000000	H
H37	1.0	0.407926	0.514890	0.605055	Biso	1.000000	H
H38	1.0	0.916263	0.180784	0.469080	Biso	1.000000	H
H39	1.0	0.926009	0.671755	0.116221	Biso	1.000000	H
H40	1.0	0.256365	0.615863	0.418052	Biso	1.000000	H
H41	1.0	0.329967	0.591711	0.476077	Biso	1.000000	H
H42	1.0	0.374872	0.656286	0.439258	Biso	1.000000	H
H43	1.0	0.263811	0.401158	0.630943	Biso	1.000000	H
H44	1.0	0.204542	0.320791	0.422329	Biso	1.000000	H
H45	1.0	0.309710	0.115504	0.513336	Biso	1.000000	H
H46	1.0	0.365256	0.069979	0.605381	Biso	1.000000	H
H47	1.0	0.396243	0.141127	0.569904	Biso	1.000000	H
H48	1.0	0.295357	0.787040	0.319936	Biso	1.000000	H
H49	1.0	0.224253	0.791279	0.224148	Biso	1.000000	H
H50	1.0	0.174066	0.764790	0.287585	Biso	1.000000	H
H51	1.0	0.498846	0.915219	0.035109	Biso	1.000000	H
H52	1.0	0.377565	0.946507	0.022767	Biso	1.000000	H
H53	1.0	0.459503	0.951023	0.091846	Biso	1.000000	H
H54	1.0	0.446346	0.013675	0.007200	Biso	1.000000	H
H55	1.0	0.005576	0.008806	0.843113	Biso	1.000000	H
H56	1.0	0.918831	0.999149	0.833460	Biso	1.000000	H
H57	1.0	0.985016	0.847548	0.875119	Biso	1.000000	H
H58	1.0	0.073588	0.038130	0.985239	Biso	1.000000	H
H59	1.0	0.599760	0.441904	0.006229	Biso	1.000000	H
H60	1.0	0.519592	0.393316	0.994128	Biso	1.000000	H
H61	1.0	0.269313	0.220098	0.289005	Biso	1.000000	H
H62	1.0	0.241138	0.342821	0.276108	Biso	1.000000	H
H63	1.0	0.630006	0.393931	0.938180	Biso	1.000000	H
H64	1.0	0.489010	0.956426	0.580217	Biso	1.000000	H
H65	1.0	0.795926	0.074903	0.248027	Biso	1.000000	H
H66	1.0	0.824101	0.183932	0.178252	Biso	1.000000	H
H67	1.0	0.714868	0.493693	0.122564	Biso	1.000000	H
H68	1.0	0.682344	0.599887	0.134384	Biso	1.000000	H
H69	1.0	0.631028	0.660914	0.083466	Biso	1.000000	H
H70	1.0	0.726381	0.630072	0.029409	Biso	1.000000	H
H71	1.0	0.644857	0.628429	0.349696	Biso	1.000000	H
H72	1.0	0.582532	0.707137	0.352944	Biso	1.000000	H

H73	1.0	0.553179	0.591129	0.327315	Biso	1.000000	H
H74	1.0	0.030629	0.371263	0.627619	Biso	1.000000	H
H75	1.0	0.500424	0.520365	0.509226	Biso	1.000000	H
H76	1.0	0.364964	0.012817	0.564578	Biso	1.000000	H
H77	1.0	0.217407	0.065198	0.906406	Biso	1.000000	H
H78	1.0	0.616395	0.933160	0.996037	Biso	1.000000	H
H79	1.0	0.555492	0.908200	0.048950	Biso	1.000000	H
H80	1.0	0.614993	0.028914	0.765989	Biso	1.000000	H
H81	1.0	0.718997	0.100489	0.790681	Biso	1.000000	H
H82	1.0	0.835373	0.559880	0.455551	Biso	1.000000	H
H83	1.0	0.743194	0.606207	0.428010	Biso	1.000000	H
H84	1.0	0.290965	0.054536	0.519820	Biso	1.000000	H
H85	1.0	0.268281	0.962344	0.462402	Biso	1.000000	H
H86	1.0	0.196011	0.037664	0.502985	Biso	1.000000	H
H87	1.0	0.179815	0.544704	0.319904	Biso	1.000000	H
H88	1.0	0.227047	0.610672	0.264188	Biso	1.000000	H
H89	1.0	0.160187	0.613457	0.343162	Biso	1.000000	H
H90	1.0	0.745126	0.843194	0.277659	Biso	1.000000	H
H91	1.0	0.199168	0.408882	0.639638	Biso	1.000000	H
H92	1.0	0.138892	0.444252	0.739466	Biso	1.000000	H
H93	1.0	0.180943	0.811433	0.770325	Biso	1.000000	H
H94	1.0	0.139788	0.732984	0.697678	Biso	1.000000	H
H95	1.0	0.742662	0.923796	0.118054	Biso	1.000000	H
H96	1.0	0.913556	0.805524	0.544641	Biso	1.000000	H
H97	1.0	0.036824	0.792795	0.330006	Biso	1.000000	H
H98	1.0	0.792915	0.334554	0.850143	Biso	1.000000	H
H99	1.0	0.858189	0.277125	0.832856	Biso	1.000000	H
H100	1.0	0.785699	0.246646	0.779712	Biso	1.000000	H
H101	1.0	0.804537	0.347720	0.740257	Biso	1.000000	H
H102	1.0	0.864281	0.350310	0.180316	Biso	1.000000	H
H103	1.0	0.823838	0.272310	0.259504	Biso	1.000000	H
H104	1.0	0.672864	0.186338	0.538605	Biso	1.000000	H
H105	1.0	0.642781	0.241977	0.583647	Biso	1.000000	H
H106	1.0	0.172388	0.151041	0.343435	Biso	1.000000	H
H107	1.0	0.207984	0.082591	0.286895	Biso	1.000000	H
H108	1.0	0.480998	0.075064	0.266040	Biso	1.000000	H
H109	1.0	0.411016	0.072368	0.322501	Biso	1.000000	H
H110	1.0	0.282276	0.421843	0.678465	Biso	1.000000	H
H111	1.0	0.230763	0.619749	0.531866	Biso	1.000000	H
H112	1.0	0.031831	0.604208	0.778313	Biso	1.000000	H
H113	1.0	0.943089	0.586860	0.758898	Biso	1.000000	H
H114	1.0	0.042470	0.522023	0.784141	Biso	1.000000	H
H115	1.0	0.724736	0.988332	0.589173	Biso	1.000000	H
H116	1.0	0.408986	0.924384	0.167701	Biso	1.000000	H
H117	1.0	0.457800	0.911022	0.278186	Biso	1.000000	H
H118	1.0	0.270445	0.366867	0.423918	Biso	1.000000	H
H119	1.0	0.712367	0.988986	0.917176	Biso	1.000000	H
H120	1.0	0.692026	0.938228	0.823047	Biso	1.000000	H
H121	1.0	0.187967	0.745085	0.851312	Biso	1.000000	H
H122	1.0	0.401765	0.508297	0.926821	Biso	1.000000	H
H123	1.0	0.136808	0.232445	0.797620	Biso	1.000000	H
H124	1.0	0.480943	0.152802	0.034370	Biso	1.000000	H
H125	1.0	0.495551	0.166737	0.911249	Biso	1.000000	H
H126	1.0	0.531211	0.217339	0.990941	Biso	1.000000	H
H127	1.0	0.722556	0.273648	0.398405	Biso	1.000000	H
H128	1.0	0.608018	0.697876	0.210706	Biso	1.000000	H
H129	1.0	0.595426	0.467056	0.615726	Biso	1.000000	H
H130	1.0	0.621262	0.398806	0.719749	Biso	1.000000	H
H131	1.0	0.547970	0.470246	0.677078	Biso	1.000000	H
H132	1.0	0.413271	0.205069	0.907406	Biso	1.000000	H
H133	1.0	0.822382	0.910820	0.718922	Biso	1.000000	H
H134	1.0	0.995645	0.595603	0.999716	Biso	1.000000	H
H135	1.0	0.246113	0.180002	0.918173	Biso	1.000000	H
H136	1.0	0.193588	0.230107	0.983032	Biso	1.000000	H
H137	1.0	0.204161	0.838567	0.893174	Biso	1.000000	H
H138	1.0	0.102318	0.867491	0.927612	Biso	1.000000	H
H139	1.0	0.276239	0.587074	0.706419	Biso	1.000000	H
H140	1.0	0.333774	0.555964	0.894760	Biso	1.000000	H
H141	1.0	0.509237	0.443212	0.308196	Biso	1.000000	H

H142	1.0	0.602288	0.495688	0.297877	Biso	1.000000	H
H143	1.0	0.608033	0.423543	0.354243	Biso	1.000000	H
H144	1.0	0.385646	0.863448	0.350239	Biso	1.000000	H
H145	1.0	0.285935	0.868938	0.389136	Biso	1.000000	H
H146	1.0	0.379641	0.956911	0.393850	Biso	1.000000	H
H147	1.0	0.057942	0.865688	0.215871	Biso	1.000000	H
H148	1.0	0.958340	0.881986	0.215215	Biso	1.000000	H
H149	1.0	0.969310	0.792310	0.179353	Biso	1.000000	H
H150	1.0	0.258651	0.198935	0.637296	Biso	1.000000	H
H151	1.0	0.289758	0.109676	0.573689	Biso	1.000000	H
H152	1.0	0.818652	0.119684	0.932502	Biso	1.000000	H
H153	1.0	0.746067	0.157865	0.985983	Biso	1.000000	H
H154	1.0	0.722162	0.055343	0.916505	Biso	1.000000	H
H155	1.0	0.508569	0.845256	0.628595	Biso	1.000000	H
H156	1.0	0.749307	0.141537	0.567099	Biso	1.000000	H
H157	1.0	0.645850	0.064839	0.549572	Biso	1.000000	H
H158	1.0	0.032999	0.043182	0.880090	Biso	1.000000	H
H159	1.0	0.997847	0.090983	0.935929	Biso	1.000000	H
H160	1.0	0.219865	0.154976	0.136698	Biso	1.000000	H
H161	1.0	0.103381	0.508890	0.225252	Biso	1.000000	H
H162	1.0	0.791597	0.432726	0.962690	Biso	1.000000	H
H163	1.0	0.677714	0.400612	0.926616	Biso	1.000000	H
H164	1.0	0.692015	0.417221	0.020384	Biso	1.000000	H
H165	1.0	0.817514	0.390965	0.168568	Biso	1.000000	H
H166	1.0	0.030495	0.073322	0.497040	Biso	1.000000	H
H167	1.0	0.069948	0.015702	0.393797	Biso	1.000000	H
H168	1.0	0.083160	0.689139	0.235906	Biso	1.000000	H
H169	1.0	0.172120	0.760404	0.219727	Biso	1.000000	H
H170	1.0	0.056552	0.763114	0.278629	Biso	1.000000	H
H171	1.0	0.077318	0.809075	0.212349	Biso	1.000000	H
H172	1.0	0.950994	0.984078	0.228031	Biso	1.000000	H
H173	1.0	0.853305	0.009045	0.219539	Biso	1.000000	H
H174	1.0	0.874612	0.958768	0.128519	Biso	1.000000	H
H175	1.0	0.491859	0.462323	0.521993	Biso	1.000000	H
H176	1.0	0.740397	0.991874	0.102782	Biso	1.000000	H
H177	1.0	0.821698	0.946516	0.094581	Biso	1.000000	H
H178	1.0	0.800773	0.933193	0.185809	Biso	1.000000	H
H179	1.0	0.557701	0.877518	0.153828	Biso	1.000000	H
H180	1.0	0.499987	0.791589	0.196629	Biso	1.000000	H
H181	1.0	0.829322	0.882451	0.075994	Biso	1.000000	H
H182	1.0	0.288813	0.920438	0.325482	Biso	1.000000	H
H183	1.0	0.233977	0.012162	0.315296	Biso	1.000000	H
H184	1.0	0.300112	0.945982	0.229899	Biso	1.000000	H
H185	1.0	0.103972	0.642915	0.070757	Biso	1.000000	H
H186	1.0	0.294333	0.306860	0.984107	Biso	1.000000	H
H187	1.0	0.264861	0.413220	0.995689	Biso	1.000000	H
H188	1.0	0.606240	0.211541	0.627038	Biso	1.000000	H
H189	1.0	0.006479	0.199602	0.156451	Biso	1.000000	H
H190	1.0	0.035850	0.324144	0.147182	Biso	1.000000	H
H191	1.0	0.990447	0.206854	0.833360	Biso	1.000000	H
H192	1.0	0.353738	0.320463	0.895331	Biso	1.000000	H
H193	1.0	0.278268	0.312573	0.840293	Biso	1.000000	H
H194	1.0	0.020305	0.663513	0.402952	Biso	1.000000	H
H195	1.0	0.438123	0.644549	0.223855	Biso	1.000000	H
H196	1.0	0.524858	0.708609	0.288034	Biso	1.000000	H
H197	1.0	0.334373	0.893428	0.836764	Biso	1.000000	H
H198	1.0	0.035124	0.365426	0.561535	Biso	1.000000	H
H199	1.0	0.984717	0.341672	0.508869	Biso	1.000000	H
H200	1.0	0.097382	0.299733	0.491620	Biso	1.000000	H
H201	1.0	0.094682	0.396949	0.551363	Biso	1.000000	H
H202	1.0	0.402373	0.339827	0.751998	Biso	1.000000	H
H203	1.0	0.285400	0.259583	0.035457	Biso	1.000000	H
H204	1.0	0.394144	0.304207	0.042395	Biso	1.000000	H
H205	1.0	0.242791	0.508058	0.987651	Biso	1.000000	H
H206	1.0	0.310923	0.444322	0.024496	Biso	1.000000	H
H207	1.0	0.989453	0.322555	0.281539	Biso	1.000000	H
H208	1.0	0.075148	0.373415	0.353366	Biso	1.000000	H
H209	1.0	0.931195	0.148163	0.001894	Biso	1.000000	H
H210	1.0	0.884784	0.093559	0.952424	Biso	1.000000	H
H211	1.0	0.769664	0.208731	0.835740	Biso	1.000000	H
H212	1.0	0.731746	0.200369	0.730123	Biso	1.000000	H
H213	1.0	0.680830	0.307294	0.897750	Biso	1.000000	H
H214	1.0	0.595933	0.069770	0.403034	Biso	1.000000	H
H215	1.0	0.877739	0.811587	0.283404	Biso	1.000000	H
H216	1.0	0.069754	0.975703	0.449081	Biso	1.000000	H
H217	1.0	0.165249	0.999258	0.482491	Biso	1.000000	H
H218	1.0	0.463773	0.562253	0.314314	Biso	1.000000	H
H219	1.0	0.608768	0.909678	0.934630	Biso	1.000000	H
H220	1.0	0.644034	0.937010	0.854033	Biso	1.000000	H
H221	1.0	0.573238	0.861148	0.827257	Biso	1.000000	H

H222	1.0	0.662710	0.852513	0.834870	Biso	1.000000	H
H223	1.0	0.933007	0.530995	0.194933	Biso	1.000000	H
H224	1.0	0.188145	0.672177	0.390259	Biso	1.000000	H
H225	1.0	0.215181	0.575157	0.450562	Biso	1.000000	H
H226	1.0	0.144816	0.578108	0.460379	Biso	1.000000	H
H227	1.0	0.539747	0.969522	0.032001	Biso	1.000000	H
H228	1.0	0.483720	0.025818	0.090175	Biso	1.000000	H
H229	1.0	0.505775	0.924242	0.133681	Biso	1.000000	H
H230	1.0	0.683855	0.825711	0.891435	Biso	1.000000	H
H231	1.0	0.775337	0.816920	0.983852	Biso	1.000000	H
H232	1.0	0.767348	0.877108	0.921632	Biso	1.000000	H
H233	1.0	0.707702	0.355426	0.637958	Biso	1.000000	H
H234	1.0	0.310535	0.585718	0.936638	Biso	1.000000	H
H235	1.0	0.386900	0.495471	0.985183	Biso	1.000000	H
H236	1.0	0.903590	0.811976	0.869235	Biso	1.000000	H
H237	1.0	0.979320	0.860982	0.937471	Biso	1.000000	H
H238	1.0	0.181215	0.880862	0.765538	Biso	1.000000	H
H239	1.0	0.226998	0.808102	0.844347	Biso	1.000000	H
H240	1.0	0.430001	0.952283	0.753331	Biso	1.000000	H
H241	1.0	0.859261	0.714753	0.513295	Biso	1.000000	H
H242	1.0	0.895289	0.658119	0.625145	Biso	1.000000	H
H243	1.0	0.140519	0.303706	0.123510	Biso	1.000000	H
H244	1.0	0.161863	0.216660	0.188479	Biso	1.000000	H
H245	1.0	0.718331	0.808382	0.716984	Biso	1.000000	H
H246	1.0	0.256785	0.232702	0.832803	Biso	1.000000	H
H247	1.0	0.326779	0.147592	0.820101	Biso	1.000000	H
H248	1.0	0.809178	0.202236	0.237319	Biso	1.000000	H
H249	1.0	0.445422	0.097931	0.222279	Biso	1.000000	H
H250	1.0	0.509038	0.095013	0.163398	Biso	1.000000	H
H251	1.0	0.028507	0.791631	0.828541	Biso	1.000000	H
H252	1.0	0.255077	0.048321	0.163680	Biso	1.000000	H
H253	1.0	0.481519	0.454261	0.674452	Biso	1.000000	H
H254	1.0	0.363820	0.498007	0.656230	Biso	1.000000	H
H255	1.0	0.807444	0.393476	0.853734	Biso	1.000000	H
H256	1.0	0.070696	0.480994	0.554028	Biso	1.000000	H
H257	1.0	0.101727	0.596173	0.534478	Biso	1.000000	H
H258	1.0	0.396811	0.736553	0.693301	Biso	1.000000	H
H259	1.0	0.479588	0.780823	0.768379	Biso	1.000000	H
H260	1.0	0.643354	0.040782	0.336604	Biso	1.000000	H
H261	1.0	0.709022	0.020608	0.441366	Biso	1.000000	H
H262	1.0	0.530400	0.744292	0.828878	Biso	1.000000	H
H263	1.0	0.538716	0.832881	0.871484	Biso	1.000000	H
H264	1.0	0.115613	0.744856	0.009548	Biso	1.000000	H
H265	1.0	0.629224	0.461416	0.775197	Biso	1.000000	H
H266	1.0	0.664653	0.442142	0.891769	Biso	1.000000	H
H267	1.0	0.525707	0.268366	0.244716	Biso	1.000000	H
H268	1.0	0.970538	0.151495	0.925651	Biso	1.000000	H
H269	1.0	0.963939	0.246769	0.966967	Biso	1.000000	H
H270	1.0	0.118708	0.235549	0.369237	Biso	1.000000	H
H271	1.0	0.223987	0.666175	0.237775	Biso	1.000000	H
H272	1.0	0.329043	0.740017	0.265957	Biso	1.000000	H
H273	1.0	0.840953	0.588464	0.005781	Biso	1.000000	H
H274	1.0	0.770032	0.600894	0.994111	Biso	1.000000	H
H275	1.0	0.575549	0.241036	0.717125	Biso	1.000000	H
H276	1.0	0.675890	0.428150	0.389482	Biso	1.000000	H
H277	1.0	0.546218	0.750506	0.962831	Biso	1.000000	H
H278	1.0	0.427621	0.311304	0.420432	Biso	1.000000	H
H279	1.0	0.504486	0.255617	0.388638	Biso	1.000000	H
H280	1.0	0.808840	0.620105	0.180546	Biso	1.000000	H
H281	1.0	0.723885	0.660552	0.119344	Biso	1.000000	H
H282	1.0	0.608519	0.519268	0.093657	Biso	1.000000	H
H283	1.0	0.653372	0.556208	0.176146	Biso	1.000000	H
H284	1.0	0.549032	0.204735	0.103542	Biso	1.000000	H
H285	1.0	0.120373	0.838524	0.018218	Biso	1.000000	H
H286	1.0	0.116104	0.911058	0.100958	Biso	1.000000	H
H287	1.0	0.515654	0.743469	0.339718	Biso	1.000000	H
H288	1.0	0.957468	0.549157	0.017778	Biso	1.000000	H
H289	1.0	0.688586	0.776759	0.440255	Biso	1.000000	H
H290	1.0	0.754744	0.759967	0.425935	Biso	1.000000	H
H291	1.0	0.215562	0.998407	0.152029	Biso	1.000000	H
H292	1.0	0.216017	0.938478	0.076391	Biso	1.000000	H
H293	1.0	0.090819	0.545599	0.822918	Biso	1.000000	H
H294	1.0	0.088746	0.539548	0.722651	Biso	1.000000	H
H295	1.0	0.727791	0.921671	0.771718	Biso	1.000000	H

H296	1.0	0.930011	0.747295	0.517839	Biso	1.000000	H
H297	1.0	0.967197	0.639264	0.459652	Biso	1.000000	H
H298	1.0	0.902880	0.725227	0.425076	Biso	1.000000	H
H299	1.0	0.486876	0.795273	0.264433	Biso	1.000000	H
H300	1.0	0.420846	0.802259	0.307310	Biso	1.000000	H
H301	1.0	0.251814	0.605673	0.904940	Biso	1.000000	H
H302	1.0	0.279970	0.634398	0.023521	Biso	1.000000	H
H303	1.0	0.993171	0.439926	0.091080	Biso	1.000000	H
H304	1.0	0.161304	0.323499	0.819374	Biso	1.000000	H
H305	1.0	0.705736	0.426199	0.851438	Biso	1.000000	H
H306	1.0	0.811286	0.398894	0.793589	Biso	1.000000	H
H307	1.0	0.945495	0.503596	0.547529	Biso	1.000000	H
H308	1.0	0.046641	0.423818	0.536890	Biso	1.000000	H
H309	1.0	0.986492	0.455718	0.474289	Biso	1.000000	H
H310	1.0	0.538722	0.356680	0.755161	Biso	1.000000	H
H311	1.0	0.439623	0.398038	0.730970	Biso	1.000000	H
H312	1.0	0.679631	0.095987	0.944181	Biso	1.000000	H
H313	1.0	0.597328	0.038692	0.025987	Biso	1.000000	H
H314	1.0	0.665064	0.015555	0.935445	Biso	1.000000	H
H315	1.0	0.535887	0.335115	0.233697	Biso	1.000000	H
H316	1.0	0.953304	0.357964	0.319517	Biso	1.000000	H
H317	1.0	0.873518	0.222220	0.884798	Biso	1.000000	H
H318	1.0	0.801329	0.633002	0.683419	Biso	1.000000	H
H319	1.0	0.905669	0.556124	0.679928	Biso	1.000000	H
H320	1.0	0.794771	0.555485	0.678570	Biso	1.000000	H
H321	1.0	0.896655	0.608612	0.740037	Biso	1.000000	H
H322	1.0	0.558263	0.875399	0.370495	Biso	1.000000	H
H323	1.0	0.794611	0.598531	0.399352	Biso	1.000000	H
H324	1.0	0.325568	0.993270	0.591301	Biso	1.000000	H
H325	1.0	0.290655	0.917845	0.590641	Biso	1.000000	H
H326	1.0	0.803842	0.015446	0.784443	Biso	1.000000	H
H327	1.0	0.788612	0.919817	0.834890	Biso	1.000000	H
H328	1.0	0.555240	0.585130	0.535650	Biso	1.000000	H
H329	1.0	0.045957	0.074974	0.227550	Biso	1.000000	H
H330	1.0	0.095564	0.019749	0.177914	Biso	1.000000	H
H331	1.0	0.405960	0.352661	0.865257	Biso	1.000000	H
H332	1.0	0.311725	0.872269	0.628753	Biso	1.000000	H
H333	1.0	0.462195	0.353934	0.664989	Biso	1.000000	H
H334	1.0	0.358038	0.110577	0.769548	Biso	1.000000	H
H335	1.0	0.322282	0.088453	0.875972	Biso	1.000000	H
H336	1.0	0.406774	0.720608	0.125462	Biso	1.000000	H
H337	1.0	0.488967	0.316281	0.321217	Biso	1.000000	H
H338	1.0	0.562264	0.385433	0.375654	Biso	1.000000	H
H339	1.0	0.555298	0.255999	0.364594	Biso	1.000000	H
H340	1.0	0.606440	0.691551	0.896800	Biso	1.000000	H
H341	1.0	0.636506	0.628167	0.966598	Biso	1.000000	H
H342	1.0	0.564398	0.589417	0.952682	Biso	1.000000	H
H343	1.0	0.215439	0.176823	0.236745	Biso	1.000000	H
H344	1.0	0.183196	0.111577	0.149574	Biso	1.000000	H
H345	1.0	0.234682	0.078560	0.218716	Biso	1.000000	H
H346	1.0	0.515353	0.406491	0.653331	Biso	1.000000	H
H347	1.0	0.387053	0.385306	0.433140	Biso	1.000000	H
H348	1.0	0.351387	0.308675	0.356360	Biso	1.000000	H
H349	1.0	0.988541	0.978051	0.956813	Biso	1.000000	H
H350	1.0	0.903998	0.040438	0.934073	Biso	1.000000	H
H351	1.0	0.018462	0.055578	0.988690	Biso	1.000000	H
H352	1.0	0.329538	0.651517	0.677524	Biso	1.000000	H
H353	1.0	0.356306	0.665732	0.780573	Biso	1.000000	H
H354	1.0	0.679347	0.942476	0.018088	Biso	1.000000	H
H355	1.0	0.728451	0.922476	0.937110	Biso	1.000000	H
H356	1.0	0.004902	0.935660	0.121061	Biso	1.000000	H
H357	1.0	0.037701	0.052866	0.098473	Biso	1.000000	H
H358	1.0	0.973399	0.003156	0.173383	Biso	1.000000	H
H359	1.0	0.559960	0.431928	0.445830	Biso	1.000000	H
H360	1.0	0.746515	0.627606	0.714443	Biso	1.000000	H
H361	1.0	0.756786	0.721895	0.705566	Biso	1.000000	H
H362	1.0	0.057740	0.412382	0.387046	Biso	1.000000	H
H363	1.0	0.511681	0.030425	0.255151	Biso	1.000000	H
H364	1.0	0.982620	0.260517	0.634352	Biso	1.000000	H
H365	1.0	0.915134	0.251439	0.712848	Biso	1.000000	H
H366	1.0	0.187247	0.549130	0.632212	Biso	1.000000	H
H367	1.0	0.156246	0.909753	0.058486	Biso	1.000000	H
H368	1.0	0.854327	0.625800	0.226739	Biso	1.000000	H
H369	1.0	0.886599	0.619743	0.134807	Biso	1.000000	H
H370	1.0	0.139082	0.754067	0.496738	Biso	1.000000	H
H371	1.0	0.622469	0.042111	0.608245	Biso	1.000000	H
H372	1.0	0.381384	0.718166	0.755536	Biso	1.000000	H
H373	1.0	0.725275	0.309341	0.439082	Biso	1.000000	H
H374	1.0	0.224618	0.574053	0.191408	Biso	1.000000	H
H375	1.0	0.157581	0.482179	0.132374	Biso	1.000000	H

H376	1.0	0.458095	0.131845	0.140165	Biso	1.000000	H
H377	1.0	0.444163	0.194950	0.064751	Biso	1.000000	H
H378	1.0	0.846391	0.448220	0.491977	Biso	1.000000	H
H379	1.0	0.819286	0.492429	0.590603	Biso	1.000000	H
H380	1.0	0.741999	0.279291	0.909191	Biso	1.000000	H
H381	1.0	0.050258	0.419726	0.059612	Biso	1.000000	H
H382	1.0	0.004153	0.381999	0.010600	Biso	1.000000	H
H383	1.0	0.078908	0.308946	0.035065	Biso	1.000000	H
H384	1.0	0.088053	0.522572	0.386029	Biso	1.000000	H
H385	1.0	0.209324	0.520666	0.371621	Biso	1.000000	H
H386	1.0	0.996627	0.788005	0.277787	Biso	1.000000	H
H387	1.0	0.016035	0.140948	0.494824	Biso	1.000000	H
H388	1.0	0.872846	0.977273	0.725518	Biso	1.000000	H
H389	1.0	0.840545	0.573875	0.394064	Biso	1.000000	H
H390	1.0	0.955511	0.513738	0.392235	Biso	1.000000	H
H391	1.0	0.419844	0.018015	0.925809	Biso	1.000000	H
H392	1.0	0.469561	0.054507	0.813960	Biso	1.000000	H
H393	1.0	0.489745	0.966770	0.847548	Biso	1.000000	H
H394	1.0	0.515306	0.079993	0.826873	Biso	1.000000	H
H395	1.0	0.466704	0.039920	0.747993	Biso	1.000000	H
H396	1.0	0.198287	0.405900	0.987979	Biso	1.000000	H
H397	1.0	0.901300	0.475513	0.172721	Biso	1.000000	H
H398	1.0	0.818363	0.508312	0.781517	Biso	1.000000	H
H399	1.0	0.823673	0.506255	0.703951	Biso	1.000000	H
H400	1.0	0.890520	0.417688	0.723231	Biso	1.000000	H
H401	1.0	0.910406	0.523416	0.757720	Biso	1.000000	H
H402	1.0	0.589609	0.190497	0.144055	Biso	1.000000	H
H403	1.0	0.691905	0.762837	0.893402	Biso	1.000000	H
H404	1.0	0.121528	0.334225	0.072131	Biso	1.000000	H
H405	1.0	0.434309	0.004174	0.091376	Biso	1.000000	H
H406	1.0	0.395264	0.058218	0.998254	Biso	1.000000	H
H407	1.0	0.007490	0.939621	0.636888	Biso	1.000000	H
H408	1.0	0.040417	0.775511	0.479598	Biso	1.000000	H
H409	1.0	0.565635	0.464874	0.840753	Biso	1.000000	H
H410	1.0	0.617034	0.998356	0.574055	Biso	1.000000	H
H411	1.0	0.858734	0.320879	0.008303	Biso	1.000000	H
H412	1.0	0.919364	0.207674	0.004324	Biso	1.000000	H
H413	1.0	0.126512	0.118901	0.315351	Biso	1.000000	H
H414	1.0	0.061214	0.222637	0.352384	Biso	1.000000	H
H415	1.0	0.903344	0.751655	0.905500	Biso	1.000000	H
H416	1.0	0.752570	0.307309	0.144599	Biso	1.000000	H
H417	1.0	0.759606	0.769979	0.728798	Biso	1.000000	H
H418	1.0	0.882815	0.772059	0.727995	Biso	1.000000	H
H419	1.0	0.377527	0.682708	0.203920	Biso	1.000000	H
H420	1.0	0.643284	0.212981	0.013934	Biso	1.000000	H
H421	1.0	0.441400	0.130454	0.601705	Biso	1.000000	H
H422	1.0	0.505352	0.986499	0.985602	Biso	1.000000	H
H423	1.0	0.564949	0.001392	0.880395	Biso	1.000000	H
H424	1.0	0.618537	0.976131	0.954875	Biso	1.000000	H
H425	1.0	0.507222	0.954466	0.901930	Biso	1.000000	H
H426	1.0	0.948683	0.605875	0.314120	Biso	1.000000	H
H427	1.0	0.992400	0.599076	0.421303	Biso	1.000000	H
H428	1.0	0.706657	0.546299	0.200828	Biso	1.000000	H
H429	1.0	0.664525	0.523567	0.300137	Biso	1.000000	H
H430	1.0	0.309872	0.894594	0.907863	Biso	1.000000	H
H431	1.0	0.963418	0.236870	0.798200	Biso	1.000000	H
H432	1.0	0.320794	0.709604	0.205373	Biso	1.000000	H
H433	1.0	0.250424	0.712199	0.195093	Biso	1.000000	H
H434	1.0	0.881214	0.485546	0.533683	Biso	1.000000	H
H435	1.0	0.821759	0.558056	0.585409	Biso	1.000000	H
H436	1.0	0.899012	0.568889	0.625567	Biso	1.000000	H
H437	1.0	0.854350	0.575277	0.511847	Biso	1.000000	H
H438	1.0	0.270986	0.396796	0.169773	Biso	1.000000	H
H439	1.0	0.335804	0.528951	0.329859	Biso	1.000000	H
H440	1.0	0.863665	0.042805	0.825413	Biso	1.000000	H
H441	1.0	0.092242	0.726149	0.678403	Biso	1.000000	H
H442	1.0	0.608258	0.331141	0.531262	Biso	1.000000	H
H443	1.0	0.665414	0.412458	0.575928	Biso	1.000000	H
H444	1.0	0.723534	0.863921	0.385486	Biso	1.000000	H
H445	1.0	0.390357	0.516862	0.363750	Biso	1.000000	H
H446	1.0	0.357404	0.501414	0.431710	Biso	1.000000	H
H447	1.0	0.280946	0.551493	0.308462	Biso	1.000000	H
H448	1.0	0.287697	0.517977	0.212910	Biso	1.000000	H
H449	1.0	0.755095	0.100256	0.103120	Biso	1.000000	H
H450	1.0	0.397149	0.684204	0.031452	Biso	1.000000	H
H451	1.0	0.646081	0.293142	0.616770	Biso	1.000000	H
H452	1.0	0.663118	0.372737	0.606524	Biso	1.000000	H
H453	1.0	0.226779	0.746364	0.811530	Biso	1.000000	H
H454	1.0	0.065089	0.330252	0.193788	Biso	1.000000	H
H455	1.0	0.137716	0.366638	0.130499	Biso	1.000000	H
H456	1.0	0.094140	0.427221	0.232302	Biso	1.000000	H

H457	1.0	0.196103	0.524938	0.874767	Biso	1.000000	H
H458	1.0	0.244342	0.549479	0.937711	Biso	1.000000	H
H459	1.0	0.231304	0.441415	0.959243	Biso	1.000000	H
H460	1.0	0.775809	0.923612	0.706179	Biso	1.000000	H
H461	1.0	0.239369	0.344802	0.470616	Biso	1.000000	H
H462	1.0	0.308335	0.445802	0.513576	Biso	1.000000	H
H463	1.0	0.488399	0.380117	0.317333	Biso	1.000000	H
H464	1.0	0.419818	0.479086	0.335071	Biso	1.000000	H
H465	1.0	0.782147	0.489077	0.968345	Biso	1.000000	H
H466	1.0	0.666850	0.364775	0.707114	Biso	1.000000	H
H467	1.0	0.687408	0.512900	0.447721	Biso	1.000000	H
H468	1.0	0.731077	0.672286	0.738557	Biso	1.000000	H
H469	1.0	0.998703	0.287306	0.227814	Biso	1.000000	H
H470	1.0	0.967790	0.232222	0.190172	Biso	1.000000	H
H471	1.0	0.335760	0.104454	0.647696	Biso	1.000000	H
H472	1.0	0.387768	0.004336	0.674947	Biso	1.000000	H
H473	1.0	0.398507	0.108748	0.724043	Biso	1.000000	H
H474	1.0	0.606003	0.726779	0.560629	Biso	1.000000	H
H475	1.0	0.662119	0.495872	0.100501	Biso	1.000000	H
H476	1.0	0.706744	0.475598	0.015931	Biso	1.000000	H
H477	1.0	0.995104	0.008750	0.328748	Biso	1.000000	H
H478	1.0	0.975735	0.901375	0.314956	Biso	1.000000	H
H479	1.0	0.935735	0.960550	0.285781	Biso	1.000000	H
H480	1.0	0.819480	0.834117	0.114781	Biso	1.000000	H
H481	1.0	0.155648	0.426639	0.948775	Biso	1.000000	H
H482	1.0	0.578980	0.531122	0.661961	Biso	1.000000	H
H483	1.0	0.532372	0.064026	0.086250	Biso	1.000000	H
H484	1.0	0.625446	0.147359	0.065072	Biso	1.000000	H
H485	1.0	0.535945	0.146115	0.141360	Biso	1.000000	H
H486	1.0	0.813467	0.849040	0.901335	Biso	1.000000	H
H487	1.0	0.266426	0.085832	0.869344	Biso	1.000000	H
H488	1.0	0.119012	0.007006	0.815458	Biso	1.000000	H
H489	1.0	0.038805	0.904082	0.675311	Biso	1.000000	H
H490	1.0	0.073168	0.962591	0.590979	Biso	1.000000	H
H491	1.0	0.481878	0.714421	0.751913	Biso	1.000000	H
H492	1.0	0.174195	0.059389	0.344880	Biso	1.000000	H
H493	1.0	0.093394	0.075878	0.276967	Biso	1.000000	H
H494	1.0	0.055125	0.010611	0.334072	Biso	1.000000	H
H495	1.0	0.519267	0.568512	0.354375	Biso	1.000000	H
H496	1.0	0.931345	0.654598	0.746671	Biso	1.000000	H
H497	1.0	0.973413	0.566950	0.694990	Biso	1.000000	H
H498	1.0	0.930089	0.669283	0.661782	Biso	1.000000	H
H499	1.0	0.819816	0.710303	0.987100	Biso	1.000000	H
H500	1.0	0.658642	0.732459	0.512439	Biso	1.000000	H
H501	1.0	0.034549	0.952516	0.488257	Biso	1.000000	H
H502	1.0	0.594019	0.667079	0.169909	Biso	1.000000	H
H503	1.0	0.480298	0.637501	0.145465	Biso	1.000000	H
H504	1.0	0.269050	0.380699	0.835019	Biso	1.000000	H
H505	1.0	0.632134	0.837436	0.232404	Biso	1.000000	H
H506	1.0	0.562676	0.733122	0.214890	Biso	1.000000	H
H507	1.0	0.032862	0.582851	0.687523	Biso	1.000000	H
H508	1.0	0.088563	0.516596	0.603267	Biso	1.000000	H
H509	1.0	0.057270	0.584527	0.580147	Biso	1.000000	H
H510	1.0	0.280050	0.900567	0.443720	Biso	1.000000	H
H511	1.0	0.248923	0.922702	0.542747	Biso	1.000000	H
H512	1.0	0.315654	0.266855	0.837312	Biso	1.000000	H
H513	1.0	0.303844	0.183513	0.873158	Biso	1.000000	H
H514	1.0	0.359682	0.246514	0.918982	Biso	1.000000	H
H515	1.0	0.530646	0.786262	0.136803	Biso	1.000000	H
H516	1.0	0.537760	0.834894	0.055752	Biso	1.000000	H
H517	1.0	0.600536	0.726083	0.946690	Biso	1.000000	H
H518	1.0	0.691511	0.684137	0.012885	Biso	1.000000	H
H519	1.0	0.374035	0.485366	0.099735	Biso	1.000000	H
H520	1.0	0.373158	0.933570	0.793343	Biso	1.000000	H
H521	1.0	0.781139	0.190145	0.683492	Biso	1.000000	H
H522	1.0	0.687182	0.236821	0.613181	Biso	1.000000	H
H523	1.0	0.642847	0.416482	0.052778	Biso	1.000000	H
H524	1.0	0.411505	0.016452	0.416044	Biso	1.000000	H
H525	1.0	0.544686	0.417625	0.106259	Biso	1.000000	H
H526	1.0	0.467010	0.498706	0.172604	Biso	1.000000	H
H527	1.0	0.592800	0.728362	0.403017	Biso	1.000000	H
H528	1.0	0.660376	0.800534	0.356178	Biso	1.000000	H
H529	1.0	0.202939	0.986873	0.260976	Biso	1.000000	H
H530	1.0	0.981792	0.744642	0.029107	Biso	1.000000	H
H531	1.0	0.398568	0.163797	0.636841	Biso	1.000000	H
H532	1.0	0.536924	0.217670	0.292812	Biso	1.000000	H

H533	1.0	0.431139	0.289751	0.303643	Biso	1.000000	H
H534	1.0	0.472871	0.214595	0.361539	Biso	1.000000	H
H535	1.0	0.023583	0.810465	0.150818	Biso	1.000000	H
H536	1.0	0.001558	0.888685	0.167810	Biso	1.000000	H
H537	1.0	0.330323	0.902994	0.037699	Biso	1.000000	H
H538	1.0	0.279640	0.853843	0.946124	Biso	1.000000	H
H539	1.0	0.123527	0.109644	0.781981	Biso	1.000000	H
H540	1.0	0.108695	0.116010	0.682337	Biso	1.000000	H
H541	1.0	0.929536	0.512427	0.705461	Biso	1.000000	H
H542	1.0	0.986377	0.514929	0.592541	Biso	1.000000	H
H543	1.0	0.998593	0.465525	0.709500	Biso	1.000000	H
H544	1.0	0.956915	0.444316	0.613815	Biso	1.000000	H
H545	1.0	0.977261	0.704729	0.701829	Biso	1.000000	H
H546	1.0	0.960007	0.815283	0.709937	Biso	1.000000	H
H547	1.0	0.431735	0.663470	0.121246	Biso	1.000000	H
H548	1.0	0.397242	0.533923	0.127293	Biso	1.000000	H
H549	1.0	0.783496	0.546320	0.744206	Biso	1.000000	H
H550	1.0	0.691569	0.595516	0.351672	Biso	1.000000	H
H551	1.0	0.684918	0.635047	0.426536	Biso	1.000000	H
H552	1.0	0.522215	0.896189	0.475960	Biso	1.000000	H
H553	1.0	0.615801	0.098469	0.326796	Biso	1.000000	H
H554	1.0	0.038894	0.103942	0.080485	Biso	1.000000	H
H555	1.0	0.921350	0.479622	0.854994	Biso	1.000000	H
H556	1.0	0.957040	0.405243	0.895445	Biso	1.000000	H
H557	1.0	0.901024	0.402632	0.781174	Biso	1.000000	H
H558	1.0	0.918632	0.512671	0.913966	Biso	1.000000	H
H559	1.0	0.971864	0.601904	0.888208	Biso	1.000000	H
H560	1.0	0.964993	0.172444	0.086002	Biso	1.000000	H
H561	1.0	0.985415	0.109498	0.987347	Biso	1.000000	H
H562	1.0	0.683543	0.096709	0.494452	Biso	1.000000	H
H563	1.0	0.652941	0.054541	0.428816	Biso	1.000000	H
H564	1.0	0.446919	0.413413	0.509012	Biso	1.000000	H
H565	1.0	0.439429	0.374682	0.426726	Biso	1.000000	H
H566	1.0	0.523466	0.460400	0.415475	Biso	1.000000	H
H567	1.0	0.777925	0.762279	0.939071	Biso	1.000000	H
H568	1.0	0.807016	0.821765	0.853001	Biso	1.000000	H
H569	1.0	0.986548	0.225525	0.370513	Biso	1.000000	H
H570	1.0	0.000801	0.307912	0.450741	Biso	1.000000	H
H571	1.0	0.225130	0.393283	0.239057	Biso	1.000000	H
H572	1.0	0.581687	0.763817	0.694456	Biso	1.000000	H
H573	1.0	0.435514	0.540130	0.895223	Biso	1.000000	H
H574	1.0	0.502943	0.638866	0.866480	Biso	1.000000	H
H575	1.0	0.432804	0.652629	0.840917	Biso	1.000000	H
H576	1.0	0.607526	0.848479	0.180628	Biso	1.000000	H
H577	1.0	0.900052	0.356192	0.004776	Biso	1.000000	H
H578	1.0	0.080043	0.934062	0.916241	Biso	1.000000	H
H579	1.0	0.033930	0.839290	0.964294	Biso	1.000000	H
H580	1.0	0.000159	0.942630	0.993890	Biso	1.000000	H
H581	1.0	0.100378	0.453463	0.017978	Biso	1.000000	H
H582	1.0	0.845568	0.510350	0.288652	Biso	1.000000	H
H583	1.0	0.605792	0.263992	0.961142	Biso	1.000000	H
H584	1.0	0.642537	0.336254	0.934747	Biso	1.000000	H
H585	1.0	0.865926	0.100242	0.339210	Biso	1.000000	H
H586	1.0	0.922335	0.798430	0.483936	Biso	1.000000	H
H587	1.0	0.105030	0.394130	0.308888	Biso	1.000000	H
H588	1.0	0.153317	0.370226	0.388465	Biso	1.000000	H
H589	1.0	0.752405	0.249710	0.536911	Biso	1.000000	H
H590	1.0	0.870648	0.785424	0.238169	Biso	1.000000	H
H591	1.0	0.916848	0.806241	0.147369	Biso	1.000000	H
H592	1.0	0.465652	0.087133	0.631667	Biso	1.000000	H
H593	1.0	0.842456	0.285073	0.438924	Biso	1.000000	H
H594	1.0	0.872484	0.378495	0.458512	Biso	1.000000	H
H595	1.0	0.920873	0.323387	0.530872	Biso	1.000000	H
H596	1.0	0.412041	0.244194	0.495683	Biso	1.000000	H
H597	1.0	0.241312	0.234909	0.484183	Biso	1.000000	H
H598	1.0	0.277625	0.151150	0.473632	Biso	1.000000	H
H599	1.0	0.243503	0.179312	0.576313	Biso	1.000000	H
H600	1.0	0.875096	0.214406	0.831038	Biso	1.000000	H
H601	1.0	0.809989	0.169577	0.849861	Biso	1.000000	H
H602	1.0	0.320930	0.962902	0.023616	Biso	1.000000	H
H603	1.0	0.336875	0.949549	0.131037	Biso	1.000000	H
H604	1.0	0.043254	0.573197	0.144217	Biso	1.000000	H

H605	1.0	0.119905	0.884070	0.751531	Biso	1.000000	H
H606	1.0	0.020437	0.846402	0.708313	Biso	1.000000	H
H607	1.0	0.117559	0.653751	0.322317	Biso	1.000000	H
H608	1.0	0.059557	0.626044	0.247411	Biso	1.000000	H
H609	1.0	0.011359	0.612471	0.326321	Biso	1.000000	H
H610	1.0	0.267519	0.515062	0.351655	Biso	1.000000	H
H611	1.0	0.264629	0.464850	0.472492	Biso	1.000000	H
H612	1.0	0.316708	0.540787	0.430722	Biso	1.000000	H
H613	1.0	0.315452	0.148382	0.911343	Biso	1.000000	H
H614	1.0	0.344395	0.050778	0.974155	Biso	1.000000	H
H615	1.0	0.457693	0.756515	0.386477	Biso	1.000000	H
H616	1.0	0.445596	0.859725	0.319854	Biso	1.000000	H
H617	1.0	0.510731	0.850092	0.381004	Biso	1.000000	H
H618	1.0	0.401122	0.724394	0.928970	Biso	1.000000	H
H619	1.0	0.493318	0.745378	0.957756	Biso	1.000000	H
H620	1.0	0.243560	0.262697	0.380351	Biso	1.000000	H
H621	1.0	0.295485	0.251433	0.460728	Biso	1.000000	H
H622	1.0	0.304950	0.259596	0.333192	Biso	1.000000	H
H623	1.0	0.659861	0.988440	0.614641	Biso	1.000000	H
H624	1.0	0.436110	0.511601	0.290388	Biso	1.000000	H
H625	1.0	0.442553	0.547197	0.171744	Biso	1.000000	H
H626	1.0	0.328192	0.677194	0.312479	Biso	1.000000	H
H627	1.0	0.368490	0.772150	0.309632	Biso	1.000000	H
H628	1.0	0.394069	0.754513	0.383646	Biso	1.000000	H
H629	1.0	0.292083	0.041759	0.001013	Biso	1.000000	H
H630	1.0	0.885427	0.024089	0.880296	Biso	1.000000	H
H631	1.0	0.599532	0.553564	0.522127	Biso	1.000000	H
H632	1.0	0.941407	0.883114	0.629328	Biso	1.000000	H
H633	1.0	0.133318	0.473315	0.964223	Biso	1.000000	H
H634	1.0	0.343349	0.618495	0.633268	Biso	1.000000	H
H635	1.0	0.276700	0.631733	0.549346	Biso	1.000000	H
H636	1.0	0.614779	0.774510	0.594540	Biso	1.000000	H
H637	1.0	0.682932	0.801659	0.677776	Biso	1.000000	H
H638	1.0	0.556526	0.810354	0.655617	Biso	1.000000	H
H639	1.0	0.136940	0.966408	0.584688	Biso	1.000000	H
H640	1.0	0.182848	0.085041	0.546010	Biso	1.000000	H
H641	1.0	0.106687	0.024602	0.522060	Biso	1.000000	H
H642	1.0	0.170868	0.050448	0.632248	Biso	1.000000	H
H643	1.0	0.925401	0.395837	0.958680	Biso	1.000000	H
H644	1.0	0.970136	0.435271	0.039372	Biso	1.000000	H
H645	1.0	0.923235	0.428786	0.665520	Biso	1.000000	H
H646	1.0	0.592322	0.655469	0.842670	Biso	1.000000	H
H647	1.0	0.386129	0.150853	0.432299	Biso	1.000000	H
H648	1.0	0.038012	0.476200	0.465756	Biso	1.000000	H
H649	1.0	0.963815	0.546754	0.446950	Biso	1.000000	H
H650	1.0	0.076128	0.529201	0.447097	Biso	1.000000	H
H651	1.0	0.530881	0.500048	0.270030	Biso	1.000000	H
H652	1.0	0.065902	0.611519	0.726145	Biso	1.000000	H
H653	1.0	0.101001	0.700086	0.724008	Biso	1.000000	H
H654	1.0	0.618867	0.086114	0.747961	Biso	1.000000	H
H655	1.0	0.254423	0.335914	0.943817	Biso	1.000000	H
H656	1.0	0.177965	0.292336	0.875679	Biso	1.000000	H
H657	1.0	0.212883	0.897082	0.692161	Biso	1.000000	H
H658	1.0	0.225656	0.910142	0.615790	Biso	1.000000	H
H659	1.0	0.378103	0.306953	0.306331	Biso	1.000000	H
H660	1.0	0.777453	0.971349	0.228371	Biso	1.000000	H
H661	1.0	0.862208	0.014834	0.278853	Biso	1.000000	H
H662	1.0	0.770319	0.114937	0.753513	Biso	1.000000	H
H663	1.0	0.321309	0.766310	0.219329	Biso	1.000000	H
H664	1.0	0.352951	0.752173	0.134612	Biso	1.000000	H
H665	1.0	0.815265	0.666413	0.787864	Biso	1.000000	H
H666	1.0	0.861484	0.592037	0.800457	Biso	1.000000	H
H667	1.0	0.503015	0.359399	0.459579	Biso	1.000000	H
H668	1.0	0.228516	0.874802	0.397321	Biso	1.000000	H
H669	1.0	0.178697	0.793914	0.346876	Biso	1.000000	H
H670	1.0	0.763196	0.690673	0.913005	Biso	1.000000	H
H671	1.0	0.802968	0.591675	0.953521	Biso	1.000000	H
H672	1.0	0.182735	0.445705	0.480167	Biso	1.000000	H
H673	1.0	0.950806	0.929784	0.601344	Biso	1.000000	H
H674	1.0	0.857862	0.884057	0.543565	Biso	1.000000	H
H675	1.0	0.164743	0.997012	0.377686	Biso	1.000000	H
H676	1.0	0.224547	0.918770	0.444429	Biso	1.000000	H

H677	1.0	0.946022	0.176519	0.203350	Biso	1.000000	H
H678	1.0	0.995349	0.067425	0.179666	Biso	1.000000	H
H679	1.0	0.454908	0.302552	0.984524	Biso	1.000000	H
H680	1.0	0.445929	0.228094	0.021046	Biso	1.000000	H
H681	1.0	0.420695	0.718320	0.868048	Biso	1.000000	H
H682	1.0	0.367607	0.838717	0.871490	Biso	1.000000	H
H683	1.0	0.161591	0.294452	0.764304	Biso	1.000000	H
H684	1.0	0.566121	0.679901	0.449935	Biso	1.000000	H
H685	1.0	0.512489	0.774010	0.394697	Biso	1.000000	H
H686	1.0	0.696760	0.036325	0.517798	Biso	1.000000	H
H687	1.0	0.405656	0.189946	0.695854	Biso	1.000000	H
H688	1.0	0.455319	0.105752	0.750333	Biso	1.000000	H
H689	1.0	0.433632	0.207207	0.795720	Biso	1.000000	H
H690	1.0	0.820914	0.918406	0.784437	Biso	1.000000	H
H691	1.0	0.786055	0.859176	0.820714	Biso	1.000000	H
H692	1.0	0.789930	0.218775	0.070031	Biso	1.000000	H
H693	1.0	0.656876	0.450047	0.705839	Biso	1.000000	H
H694	1.0	0.735044	0.492028	0.067961	Biso	1.000000	H
H695	1.0	0.387971	0.152665	0.504474	Biso	1.000000	H
H696	1.0	0.795495	0.486771	0.323114	Biso	1.000000	H
H697	1.0	0.159382	0.125020	0.438933	Biso	1.000000	H
H698	1.0	0.217223	0.138475	0.456615	Biso	1.000000	H
H699	1.0	0.681142	0.876445	0.423129	Biso	1.000000	H
H700	1.0	0.746009	0.117498	0.695541	Biso	1.000000	H
H701	1.0	0.967973	0.059842	0.346510	Biso	1.000000	H
H702	1.0	0.112220	0.646515	0.472111	Biso	1.000000	H
H703	1.0	0.039908	0.647272	0.508411	Biso	1.000000	H
H704	1.0	0.113565	0.720801	0.544706	Biso	1.000000	H
H705	1.0	0.270495	0.565404	0.254646	Biso	1.000000	H
H706	1.0	0.694241	0.795641	0.589258	Biso	1.000000	H
H707	1.0	0.733495	0.109533	0.034480	Biso	1.000000	H
H708	1.0	0.713762	0.991482	0.051885	Biso	1.000000	H
H709	1.0	0.497531	0.874172	0.564637	Biso	1.000000	H
H710	1.0	0.432019	0.953025	0.419717	Biso	1.000000	H
H711	1.0	0.443178	0.963610	0.295042	Biso	1.000000	H
H712	1.0	0.922862	0.599520	0.919082	Biso	1.000000	H
H713	1.0	0.859209	0.567623	0.868634	Biso	1.000000	H
H714	1.0	0.822506	0.542931	0.971599	Biso	1.000000	H
H715	1.0	0.792008	0.724759	0.404098	Biso	1.000000	H
H716	1.0	0.255954	0.695666	0.047434	Biso	1.000000	H
H717	1.0	0.186978	0.699230	0.159389	Biso	1.000000	H
H718	1.0	0.681606	0.753789	0.183343	Biso	1.000000	H
H719	1.0	0.521673	0.483466	0.899916	Biso	1.000000	H
H720	1.0	0.474229	0.384082	0.951891	Biso	1.000000	H
H721	1.0	0.729702	0.724922	0.101349	Biso	1.000000	H
H722	1.0	0.785598	0.109155	0.458266	Biso	1.000000	H
H723	1.0	0.767394	0.349526	0.183604	Biso	1.000000	H
H724	1.0	0.752500	0.249596	0.227898	Biso	1.000000	H
H725	1.0	0.054887	0.190734	0.619865	Biso	1.000000	H
H726	1.0	0.618899	0.834373	0.581765	Biso	1.000000	H
H727	1.0	0.219663	0.866321	0.338791	Biso	1.000000	H
H728	1.0	0.147988	0.827707	0.304672	Biso	1.000000	H
H729	1.0	0.906010	0.268889	0.558955	Biso	1.000000	H
H730	1.0	0.867831	0.944894	0.306941	Biso	1.000000	H
H731	1.0	0.277084	0.231103	0.092127	Biso	1.000000	H
H732	1.0	0.052177	0.990370	0.813148	Biso	1.000000	H
H733	1.0	0.739231	0.140254	0.141936	Biso	1.000000	H
H734	1.0	0.768254	0.190617	0.199120	Biso	1.000000	H
H735	1.0	0.724832	0.255951	0.133556	Biso	1.000000	H
H736	1.0	0.969296	0.183994	0.434864	Biso	1.000000	H
H737	1.0	0.864099	0.210173	0.370003	Biso	1.000000	H
H738	1.0	0.016321	0.188012	0.407554	Biso	1.000000	H
H739	1.0	0.651704	0.449640	0.610965	Biso	1.000000	H
H740	1.0	0.206615	0.147146	0.548385	Biso	1.000000	H
H741	1.0	0.236001	0.819115	0.941730	Biso	1.000000	H
H742	1.0	0.341149	0.775351	0.955511	Biso	1.000000	H
H743	1.0	0.376192	0.823836	0.931649	Biso	1.000000	H
H744	1.0	0.455171	0.876448	0.992987	Biso	1.000000	H
H745	1.0	0.016166	0.444997	0.405992	Biso	1.000000	H
H746	1.0	0.072632	0.498543	0.337868	Biso	1.000000	H
H747	1.0	0.718818	0.136023	0.250553	Biso	1.000000	H
H748	1.0	0.816614	0.009091	0.714572	Biso	1.000000	H

H749	1.0	0.951501	0.212683	0.518264	Biso	1.000000	H
H750	1.0	0.048129	0.240259	0.577934	Biso	1.000000	H
H751	1.0	0.940892	0.230289	0.600148	Biso	1.000000	H
H752	1.0	0.360366	0.439905	0.563854	Biso	1.000000	H
H753	1.0	0.378315	0.444581	0.461238	Biso	1.000000	H
H754	1.0	0.609260	0.275160	0.667422	Biso	1.000000	H
H755	1.0	0.561665	0.301578	0.766159	Biso	1.000000	H
H756	1.0	0.484536	0.952868	0.782195	Biso	1.000000	H
H757	1.0	0.589719	0.959808	0.842181	Biso	1.000000	H
H758	1.0	0.686820	0.548521	0.394905	Biso	1.000000	H
H759	1.0	0.706776	0.477024	0.295303	Biso	1.000000	H
H760	1.0	0.614017	0.451464	0.945931	Biso	1.000000	H
H761	1.0	0.577613	0.563595	0.000830	Biso	1.000000	H
H762	1.0	0.444365	0.251590	0.620322	Biso	1.000000	H
H763	1.0	0.223039	0.760368	0.177917	Biso	1.000000	H
H764	1.0	0.208565	0.869113	0.154484	Biso	1.000000	H
H765	1.0	0.120981	0.792554	0.163753	Biso	1.000000	H
H766	1.0	0.555929	0.190769	0.836230	Biso	1.000000	H
H767	1.0	0.497696	0.268601	0.859456	Biso	1.000000	H
H768	1.0	0.550975	0.243762	0.775171	Biso	1.000000	H
H769	1.0	0.930897	0.710728	0.731249	Biso	1.000000	H
H770	1.0	0.985689	0.735513	0.544964	Biso	1.000000	H
H771	1.0	0.983475	0.672412	0.625379	Biso	1.000000	H
H772	1.0	0.758789	0.590635	0.665270	Biso	1.000000	H
H773	1.0	0.261512	0.725193	0.467652	Biso	1.000000	H
H774	1.0	0.248823	0.665068	0.391135	Biso	1.000000	H
H775	1.0	0.707433	0.020096	0.231834	Biso	1.000000	H
H776	1.0	0.747039	0.106295	0.216707	Biso	1.000000	H
H777	1.0	0.003182	0.326034	0.607656	Biso	1.000000	H
H778	1.0	0.943605	0.379861	0.519659	Biso	1.000000	H
H779	1.0	0.623208	0.630814	0.405951	Biso	1.000000	H
H780	1.0	0.557832	0.628354	0.492359	Biso	1.000000	H
H781	1.0	0.220882	0.210993	0.156583	Biso	1.000000	H
H782	1.0	0.171551	0.301647	0.177763	Biso	1.000000	H
H783	1.0	0.875684	0.832504	0.102367	Biso	1.000000	H
H784	1.0	0.089673	0.819232	0.949780	Biso	1.000000	H
H785	1.0	0.094610	0.723701	0.900658	Biso	1.000000	H
H786	1.0	0.087942	0.699649	0.973973	Biso	1.000000	H
H787	1.0	0.999895	0.753768	0.971405	Biso	1.000000	H
H788	1.0	0.139855	0.061761	0.132589	Biso	1.000000	H
H789	1.0	0.663660	0.928177	0.219501	Biso	1.000000	H
H790	1.0	0.683036	0.846499	0.275348	Biso	1.000000	H
H791	1.0	0.666274	0.457958	0.339949	Biso	1.000000	H
H792	1.0	0.245215	0.911661	0.123397	Biso	1.000000	H
H793	1.0	0.305984	0.831985	0.204574	Biso	1.000000	H
H794	1.0	0.348735	0.903416	0.153101	Biso	1.000000	H
H795	1.0	0.831800	0.890229	0.210809	Biso	1.000000	H
H796	1.0	0.822526	0.800338	0.201016	Biso	1.000000	H
H797	1.0	0.391877	0.005153	0.308421	Biso	1.000000	H
H798	1.0	0.603828	0.301249	0.593347	Biso	1.000000	H
H799	1.0	0.465059	0.918987	0.462704	Biso	1.000000	H
H800	1.0	0.885922	0.666603	0.251332	Biso	1.000000	H
H801	1.0	0.961206	0.255881	0.024008	Biso	1.000000	H
H802	1.0	0.994974	0.481416	0.761613	Biso	1.000000	H
H803	1.0	0.994759	0.416106	0.830997	Biso	1.000000	H
H804	1.0	0.084078	0.443737	0.767930	Biso	1.000000	H
H805	1.0	0.802279	0.306890	0.303262	Biso	1.000000	H
H806	1.0	0.818302	0.318428	0.400932	Biso	1.000000	H
H807	1.0	0.767012	0.227660	0.398863	Biso	1.000000	H
H808	1.0	0.817248	0.542907	0.259519	Biso	1.000000	H
H809	1.0	0.001528	0.621618	0.835515	Biso	1.000000	H
H810	1.0	0.990299	0.713236	0.928333	Biso	1.000000	H
H811	1.0	0.402092	0.686603	0.486343	Biso	1.000000	H
H812	1.0	0.565273	0.206556	0.391830	Biso	1.000000	H
H813	1.0	0.300850	0.686935	0.724509	Biso	1.000000	H
H814	1.0	0.554525	0.876924	0.428743	Biso	1.000000	H
H815	1.0	0.012103	0.757675	0.165592	Biso	1.000000	H
H816	1.0	0.027476	0.627090	0.150702	Biso	1.000000	H
H817	1.0	0.986413	0.683278	0.086258	Biso	1.000000	H
H818	1.0	0.665539	0.116179	0.817269	Biso	1.000000	H
H819	1.0	0.614568	0.201496	0.865084	Biso	1.000000	H
H820	1.0	0.708012	0.122580	0.899145	Biso	1.000000	H
H821	1.0	0.381922	0.814644	0.479261	Biso	1.000000	H
H822	1.0	0.877285	0.915677	0.995800	Biso	1.000000	H
H823	1.0	0.990162	0.936676	0.054743	Biso	1.000000	H
O1624	1.0	0.753554	0.887232	0.843025	Biso	1.000000	O
H824	1.0	0.741628	0.883174	0.869446	Biso	1.000000	H
H825	1.0	0.733037	0.895215	0.823853	Biso	1.000000	H
O1625	1.0	0.294257	0.873961	0.313082	Biso	1.000000	O
H826	1.0	0.281454	0.847071	0.315861	Biso	1.000000	H
H827	1.0	0.321631	0.872122	0.322466	Biso	1.000000	H

O1626	1.0	0.129865	0.907100	0.149394	Biso	1.000000	O
H828	1.0	0.149532	0.885812	0.155266	Biso	1.000000	H
H829	1.0	0.107833	0.904772	0.168280	Biso	1.000000	H
O1627	1.0	0.881790	0.689859	0.669911	Biso	1.000000	O
H830	1.0	0.860312	0.670546	0.675727	Biso	1.000000	H
H831	1.0	0.876822	0.715941	0.683727	Biso	1.000000	H
O1628	1.0	0.256134	0.613044	0.184848	Biso	1.000000	O
H832	1.0	0.283932	0.605498	0.188509	Biso	1.000000	H
H833	1.0	0.250917	0.639267	0.198317	Biso	1.000000	H
O1629	1.0	0.639639	0.227168	0.952752	Biso	1.000000	O
H834	1.0	0.640890	0.218129	0.924816	Biso	1.000000	H
H835	1.0	0.665519	0.237726	0.960964	Biso	1.000000	H
O1630	1.0	0.073051	0.249772	0.716413	Biso	1.000000	O
H836	1.0	0.069252	0.277630	0.705886	Biso	1.000000	H
H837	1.0	0.047275	0.237419	0.722246	Biso	1.000000	H
O1631	1.0	0.141593	0.251095	0.753021	Biso	1.000000	O
H838	1.0	0.115885	0.252830	0.739639	Biso	1.000000	H
H839	1.0	0.156414	0.227630	0.742359	Biso	1.000000	H
O1632	1.0	0.752211	0.554140	0.186397	Biso	1.000000	O
H840	1.0	0.767777	0.546932	0.210100	Biso	1.000000	H
H841	1.0	0.760862	0.580927	0.176188	Biso	1.000000	H
O1633	1.0	0.001861	0.642068	0.977588	Biso	1.000000	O
H842	1.0	0.028992	0.641525	0.967363	Biso	1.000000	H
H843	1.0	0.984660	0.655762	0.958126	Biso	1.000000	H
O1634	1.0	0.454175	0.277120	0.847537	Biso	1.000000	O
H844	1.0	0.433250	0.260911	0.834808	Biso	1.000000	H
H845	1.0	0.446244	0.306097	0.848519	Biso	1.000000	H
O1635	1.0	0.094010	0.471262	0.260533	Biso	1.000000	O
H846	1.0	0.110386	0.451147	0.274742	Biso	1.000000	H
H847	1.0	0.073148	0.481250	0.278390	Biso	1.000000	H
O1636	1.0	0.080259	0.606360	0.390713	Biso	1.000000	O
H848	1.0	0.074868	0.580963	0.405535	Biso	1.000000	H
H849	1.0	0.066668	0.605447	0.364843	Biso	1.000000	H
O1637	1.0	0.727096	0.320966	0.888266	Biso	1.000000	O
H850	1.0	0.742705	0.320303	0.863583	Biso	1.000000	H
H851	1.0	0.723584	0.349553	0.896867	Biso	1.000000	H
O1638	1.0	0.943741	0.094394	0.125915	Biso	1.000000	O
H852	1.0	0.946529	0.067887	0.112238	Biso	1.000000	H
H853	1.0	0.956513	0.115955	0.110020	Biso	1.000000	H
O1639	1.0	0.794174	0.594452	0.587405	Biso	1.000000	O
H854	1.0	0.783467	0.620726	0.577957	Biso	1.000000	H
H855	1.0	0.779483	0.585440	0.611097	Biso	1.000000	H
O1640	1.0	0.874876	0.246625	0.738566	Biso	1.000000	O
H856	1.0	0.846862	0.252747	0.734145	Biso	1.000000	H
H857	1.0	0.877684	0.229208	0.762325	Biso	1.000000	H
O1641	1.0	0.013033	0.682566	0.046804	Biso	1.000000	O
H858	1.0	0.004406	0.670055	0.021603	Biso	1.000000	H
H859	1.0	0.041089	0.676588	0.051130	Biso	1.000000	H
O1642	1.0	0.204646	0.935563	0.533490	Biso	1.000000	O
H860	1.0	0.190038	0.955992	0.549176	Biso	1.000000	H
H861	1.0	0.186039	0.915979	0.521582	Biso	1.000000	H
O1643	1.0	0.024286	0.602118	0.495483	Biso	1.000000	O
H862	1.0	0.045945	0.587867	0.509138	Biso	1.000000	H
H863	1.0	0.012967	0.584008	0.475040	Biso	1.000000	H
O1644	1.0	0.320600	0.667218	0.918114	Biso	1.000000	O
H864	1.0	0.335097	0.647817	0.901141	Biso	1.000000	H
H865	1.0	0.301316	0.682256	0.901857	Biso	1.000000	H
O1645	1.0	0.234921	0.306353	0.364169	Biso	1.000000	O
H866	1.0	0.248379	0.329821	0.376540	Biso	1.000000	H
H867	1.0	0.241050	0.305603	0.335529	Biso	1.000000	H
O1646	1.0	0.111194	0.976981	0.000711	Biso	1.000000	O
H868	1.0	0.103029	0.948461	0.005390	Biso	1.000000	H
H869	1.0	0.135796	0.977285	0.985256	Biso	1.000000	H
O1647	1.0	0.042006	0.403591	0.888656	Biso	1.000000	O
H870	1.0	0.054146	0.427378	0.901824	Biso	1.000000	H
H871	1.0	0.037178	0.410049	0.860444	Biso	1.000000	H
O1648	1.0	0.481682	0.091709	0.677509	Biso	1.000000	O
H872	1.0	0.504304	0.082701	0.693571	Biso	1.000000	H
H873	1.0	0.461886	0.104399	0.694987	Biso	1.000000	H
O1649	1.0	0.403708	0.604690	0.516728	Biso	1.000000	O
H874	1.0	0.410622	0.626442	0.535782	Biso	1.000000	H

H875	1.0	0.391993	0.581443	0.531145	Biso	1.000000	H
O1650	1.0	0.956814	0.807815	0.457600	Biso	1.000000	O
H876	1.0	0.962631	0.816103	0.430036	Biso	1.000000	H
H877	1.0	0.981616	0.803543	0.472143	Biso	1.000000	H
O1651	1.0	0.296203	0.378245	0.203243	Biso	1.000000	O
H878	1.0	0.323003	0.370407	0.211301	Biso	1.000000	H
H879	1.0	0.278882	0.378233	0.226734	Biso	1.000000	H
O1652	1.0	0.389549	0.702199	0.659862	Biso	1.000000	O
H880	1.0	0.369148	0.682630	0.651455	Biso	1.000000	H
H881	1.0	0.413891	0.697011	0.644806	Biso	1.000000	H
O1653	1.0	0.388231	0.984823	0.940347	Biso	1.000000	O
H882	1.0	0.392543	0.965693	0.962582	Biso	1.000000	H
H883	1.0	0.360120	0.985178	0.933340	Biso	1.000000	H
O1654	1.0	0.795506	0.693474	0.214211	Biso	1.000000	O
H884	1.0	0.819871	0.690806	0.229835	Biso	1.000000	H
H885	1.0	0.784214	0.666136	0.209510	Biso	1.000000	H
O1655	1.0	0.161640	0.077931	0.430798	Biso	1.000000	O
H886	1.0	0.177481	0.057844	0.445652	Biso	1.000000	H
H887	1.0	0.154669	0.066551	0.404585	Biso	1.000000	H
O1656	1.0	0.612911	0.705811	0.647277	Biso	1.000000	O
H888	1.0	0.595432	0.713604	0.669386	Biso	1.000000	H
H889	1.0	0.601839	0.715841	0.622008	Biso	1.000000	H
O1657	1.0	0.770429	0.094835	0.498468	Biso	1.000000	O
H890	1.0	0.778261	0.065813	0.498066	Biso	1.000000	H
H891	1.0	0.755514	0.100553	0.522972	Biso	1.000000	H
O1658	1.0	0.773602	0.447912	0.658821	Biso	1.000000	O
H892	1.0	0.784831	0.453267	0.632309	Biso	1.000000	H
H893	1.0	0.793407	0.453969	0.679380	Biso	1.000000	H
O1659	1.0	0.502671	0.526325	0.615767	Biso	1.000000	O
H894	1.0	0.528724	0.513841	0.611733	Biso	1.000000	H
H895	1.0	0.487189	0.509273	0.634174	Biso	1.000000	H
O1660	1.0	0.639636	0.711678	0.091405	Biso	1.000000	O
H896	1.0	0.629937	0.713918	0.063869	Biso	1.000000	H
H897	1.0	0.665394	0.725269	0.093552	Biso	1.000000	H
O1661	1.0	0.650969	0.781450	0.156204	Biso	1.000000	O
H898	1.0	0.638110	0.797815	0.177100	Biso	1.000000	H
H899	1.0	0.671773	0.797836	0.143483	Biso	1.000000	H
O1662	1.0	0.267257	0.527920	0.638972	Biso	1.000000	O
H900	1.0	0.274914	0.534426	0.611417	Biso	1.000000	H
H901	1.0	0.259140	0.553188	0.652692	Biso	1.000000	H
O1663	1.0	0.801778	0.194720	0.419603	Biso	1.000000	O
H902	1.0	0.829603	0.198141	0.412210	Biso	1.000000	H
H903	1.0	0.796120	0.165456	0.423713	Biso	1.000000	H
O1664	1.0	0.771240	0.005794	0.745264	Biso	1.000000	O
H904	1.0	0.745728	0.019811	0.747803	Biso	1.000000	H
H905	1.0	0.766850	0.976821	0.738478	Biso	1.000000	H
O1665	1.0	0.479603	0.288938	0.752115	Biso	1.000000	O
H906	1.0	0.488648	0.317541	0.753534	Biso	1.000000	H
H907	1.0	0.496356	0.271707	0.769173	Biso	1.000000	H
O1666	1.0	0.922135	0.285078	0.355445	Biso	1.000000	O
H908	1.0	0.920569	0.313396	0.345513	Biso	1.000000	H
H909	1.0	0.923762	0.266061	0.332755	Biso	1.000000	H
O1667	1.0	0.820780	0.265147	0.155517	Biso	1.000000	O
H910	1.0	0.830631	0.288324	0.171392	Biso	1.000000	H
H911	1.0	0.797880	0.274021	0.139790	Biso	1.000000	H
O1668	1.0	0.759238	0.835748	0.394735	Biso	1.000000	O
H912	1.0	0.762458	0.840047	0.423566	Biso	1.000000	H
H913	1.0	0.769966	0.808789	0.387374	Biso	1.000000	H
O1669	1.0	0.649417	0.194958	0.690636	Biso	1.000000	O
H914	1.0	0.632667	0.218610	0.697188	Biso	1.000000	H
H915	1.0	0.668087	0.202670	0.669524	Biso	1.000000	H
O1670	1.0	0.177674	0.972142	0.166608	Biso	1.000000	O
H916	1.0	0.178581	0.976438	0.195608	Biso	1.000000	H
H917	1.0	0.161907	0.947601	0.160675	Biso	1.000000	H
O1671	1.0	0.739118	0.927360	0.456618	Biso	1.000000	O
H918	1.0	0.744486	0.897796	0.454768	Biso	1.000000	H
H919	1.0	0.750190	0.938135	0.481594	Biso	1.000000	H
O1672	1.0	0.977800	0.106462	0.349998	Biso	1.000000	O
H920	1.0	0.958221	0.119698	0.367341	Biso	1.000000	H
H921	1.0	0.997394	0.126842	0.341453	Biso	1.000000	H
O1673	1.0	0.335236	0.046700	0.395474	Biso	1.000000	O
H922	1.0	0.361541	0.045364	0.407654	Biso	1.000000	H
H923	1.0	0.323084	0.073326	0.401615	Biso	1.000000	H
O1674	1.0	0.170594	0.310209	0.312880	Biso	1.000000	O
H924	1.0	0.168716	0.330529	0.333362	Biso	1.000000	H
H925	1.0	0.197563	0.309799	0.304640	Biso	1.000000	H
H926	1.0	0.158007	0.321660	0.289919	Biso	1.000000	H
O1675	1.0	0.901494	0.087405	0.186330	Biso	1.000000	O
H927	1.0	0.916521	0.092814	0.162784	Biso	1.000000	H
H928	1.0	0.874187	0.088682	0.179424	Biso	1.000000	H

H929	1.0	0.905411	0.110192	0.203850	Biso	1.000000	H
O1676	1.0	0.905214	0.593604	0.067671	Biso	1.000000	O
H930	1.0	0.916582	0.570930	0.053705	Biso	1.000000	H
H931	1.0	0.880073	0.597545	0.055380	Biso	1.000000	H
H932	1.0	0.899991	0.584898	0.094390	Biso	1.000000	H
O1677	1.0	0.369953	0.568476	0.810140	Biso	1.000000	O
H933	1.0	0.353802	0.562362	0.832742	Biso	1.000000	H
H934	1.0	0.363420	0.549289	0.789602	Biso	1.000000	H
H935	1.0	0.362601	0.595359	0.801489	Biso	1.000000	H
O1678	1.0	0.064634	0.864820	0.296259	Biso	1.000000	O
H936	1.0	0.092140	0.860379	0.300662	Biso	1.000000	H
H937	1.0	0.058069	0.853252	0.270906	Biso	1.000000	H
H938	1.0	0.052653	0.847680	0.315976	Biso	1.000000	H
O1679	1.0	0.836902	0.484491	0.861051	Biso	1.000000	O
H939	1.0	0.809219	0.482535	0.865984	Biso	1.000000	H
H940	1.0	0.845088	0.509741	0.872973	Biso	1.000000	H
H941	1.0	0.840901	0.486743	0.832903	Biso	1.000000	H
O1680	1.0	0.672748	0.957493	0.473160	Biso	1.000000	O
H942	1.0	0.686742	0.979354	0.485851	Biso	1.000000	H
H943	1.0	0.660321	0.941677	0.493625	Biso	1.000000	H
H944	1.0	0.692756	0.940330	0.461995	Biso	1.000000	H
O1681	1.0	0.252520	0.030859	0.588358	Biso	1.000000	O
H945	1.0	0.257915	0.028483	0.560459	Biso	1.000000	H
H946	1.0	0.257805	0.059141	0.593714	Biso	1.000000	H
H947	1.0	0.272880	0.016120	0.601895	Biso	1.000000	H
O1682	1.0	0.995712	0.825383	0.557153	Biso	1.000000	O
H948	1.0	0.968112	0.821475	0.561427	Biso	1.000000	H
H949	1.0	0.004419	0.797724	0.553603	Biso	1.000000	H
H950	1.0	0.005565	0.832941	0.582844	Biso	1.000000	H
O1683	1.0	0.157061	0.464816	0.578605	Biso	1.000000	O
H951	1.0	0.134334	0.481208	0.573126	Biso	1.000000	H
H952	1.0	0.177952	0.474645	0.562028	Biso	1.000000	H
H953	1.0	0.148732	0.438780	0.568524	Biso	1.000000	H
O1684	1.0	0.233758	0.881805	0.249282	Biso	1.000000	O
H954	1.0	0.222560	0.880449	0.275422	Biso	1.000000	H
H955	1.0	0.255811	0.900030	0.250327	Biso	1.000000	H
H956	1.0	0.243503	0.854817	0.244038	Biso	1.000000	H
O1685	1.0	0.079983	0.466752	0.676709	Biso	1.000000	O
H957	1.0	0.090174	0.479707	0.700128	Biso	1.000000	H
H958	1.0	0.083492	0.487961	0.657326	Biso	1.000000	H
H959	1.0	0.052131	0.465881	0.680913	Biso	1.000000	H
O1686	1.0	0.008459	0.340650	0.913878	Biso	1.000000	O
H960	1.0	0.027913	0.325479	0.928298	Biso	1.000000	H
H961	1.0	0.020994	0.365664	0.906069	Biso	1.000000	H
H962	1.0	0.005761	0.325829	0.889415	Biso	1.000000	H
O1687	1.0	0.836781	0.213618	0.496697	Biso	1.000000	O
H963	1.0	0.861180	0.199124	0.494516	Biso	1.000000	H
H964	1.0	0.834201	0.219778	0.524461	Biso	1.000000	H
H965	1.0	0.840684	0.239462	0.483793	Biso	1.000000	H
O1688	1.0	0.640893	0.256059	0.101638	Biso	1.000000	O
H966	1.0	0.644188	0.244031	0.075838	Biso	1.000000	H
H967	1.0	0.666323	0.253452	0.113646	Biso	1.000000	H
H968	1.0	0.624328	0.238130	0.116751	Biso	1.000000	H
O1689	1.0	0.158280	0.205793	0.292471	Biso	1.000000	O
H969	1.0	0.137733	0.222235	0.281281	Biso	1.000000	H
H970	1.0	0.169181	0.190283	0.270923	Biso	1.000000	H
H971	1.0	0.145451	0.185319	0.308237	Biso	1.000000	H
O1690	1.0	0.977140	0.331102	0.079352	Biso	1.000000	O
H972	1.0	0.979511	0.309088	0.060655	Biso	1.000000	H
H973	1.0	0.950476	0.330767	0.088550	Biso	1.000000	H
H974	1.0	0.992673	0.322338	0.101569	Biso	1.000000	H
O1691	1.0	0.481236	0.879975	0.913031	Biso	1.000000	O
H975	1.0	0.468284	0.869689	0.936304	Biso	1.000000	H
H976	1.0	0.492511	0.856921	0.899591	Biso	1.000000	H
H977	1.0	0.502656	0.896955	0.921439	Biso	1.000000	H
O1692	1.0	0.917508	0.316070	0.776581	Biso	1.000000	O
H978	1.0	0.892706	0.306700	0.786583	Biso	1.000000	H
H979	1.0	0.920635	0.342513	0.788512	Biso	1.000000	H
H980	1.0	0.935599	0.297266	0.788568	Biso	1.000000	H
O1693	1.0	0.525788	0.595953	0.241081	Biso	1.000000	O
H981	1.0	0.500904	0.609297	0.237843	Biso	1.000000	H
H982	1.0	0.537199	0.605194	0.265563	Biso	1.000000	H
H983	1.0	0.522768	0.566905	0.243610	Biso	1.000000	H
O1694	1.0	0.510755	0.489758	0.993066	Biso	1.000000	O
H984	1.0	0.496254	0.514905	0.992577	Biso	1.000000	H
H985	1.0	0.494601	0.472504	0.009332	Biso	1.000000	H
H986	1.0	0.511308	0.479001	0.966536	Biso	1.000000	H
O1695	1.0	0.357456	0.312600	0.467564	Biso	1.000000	O
H987	1.0	0.373349	0.295338	0.484083	Biso	1.000000	H
H988	1.0	0.338030	0.295055	0.455912	Biso	1.000000	H
H989	1.0	0.345269	0.333173	0.483725	Biso	1.000000	H
O1696	1.0	0.198593	0.498118	0.242983	Biso	1.000000	O
H990	1.0	0.190530	0.517841	0.262456	Biso	1.000000	H
H991	1.0	0.206199	0.514745	0.220782	Biso	1.000000	H
H992	1.0	0.222077	0.486128	0.253584	Biso	1.000000	H

O1697	1.0	0.559876	0.579016	0.829804	Biso	1.000000	O
H993	1.0	0.576794	0.577520	0.852564	Biso	1.000000	H
H994	1.0	0.535508	0.589369	0.839985	Biso	1.000000	H
H995	1.0	0.571026	0.602655	0.817243	Biso	1.000000	H
O1698	1.0	0.198165	0.216948	0.066918	Biso	1.000000	O
H996	1.0	0.220872	0.234311	0.066898	Biso	1.000000	H
H997	1.0	0.206775	0.195232	0.083996	Biso	1.000000	H
H998	1.0	0.195464	0.206812	0.040291	Biso	1.000000	H
O1699	1.0	0.521691	0.285997	0.941156	Biso	1.000000	O
H999	1.0	0.512958	0.261458	0.954042	Biso	1.000000	H
H1000	1.0	0.523836	0.278631	0.913629	Biso	1.000000	H
H1001	1.0	0.547773	0.289636	0.951340	Biso	1.000000	H
O1700	1.0	0.928204	0.942847	0.908622	Biso	1.000000	O
H1002	1.0	0.900839	0.941454	0.915271	Biso	1.000000	H
H1003	1.0	0.928236	0.954532	0.882459	Biso	1.000000	H
H1004	1.0	0.938668	0.963954	0.925358	Biso	1.000000	H
O1701	1.0	0.901441	0.674179	0.957677	Biso	1.000000	O
H1005	1.0	0.878550	0.682108	0.972405	Biso	1.000000	H
H1006	1.0	0.901870	0.691876	0.934929	Biso	1.000000	H
H1007	1.0	0.897347	0.647314	0.947001	Biso	1.000000	H
O1702	1.0	0.271856	0.832428	0.576603	Biso	1.000000	O
H1008	1.0	0.262281	0.804842	0.576872	Biso	1.000000	H
H1009	1.0	0.292534	0.832968	0.595968	Biso	1.000000	H
H1010	1.0	0.252421	0.850731	0.587074	Biso	1.000000	H
O1703	1.0	0.766755	0.745115	0.169693	Biso	1.000000	O
H1011	1.0	0.780360	0.725808	0.186168	Biso	1.000000	H
H1012	1.0	0.777294	0.771251	0.177040	Biso	1.000000	H
H1013	1.0	0.740678	0.744615	0.180477	Biso	1.000000	H
O1704	1.0	0.660443	0.203871	0.279470	Biso	1.000000	O
H1014	1.0	0.645615	0.192367	0.300990	Biso	1.000000	H
H1015	1.0	0.677228	0.223893	0.291543	Biso	1.000000	H
H1016	1.0	0.678548	0.182385	0.273059	Biso	1.000000	H
O1705	1.0	0.455203	0.090920	0.959794	Biso	1.000000	O
H1017	1.0	0.451074	0.070174	0.940068	Biso	1.000000	H
H1018	1.0	0.464645	0.115237	0.947019	Biso	1.000000	H
H1019	1.0	0.475674	0.081744	0.977242	Biso	1.000000	H
O1706	1.0	0.924553	0.270258	0.428045	Biso	1.000000	O
H1020	1.0	0.918915	0.275030	0.400487	Biso	1.000000	H
H1021	1.0	0.928989	0.241370	0.430135	Biso	1.000000	H
H1022	1.0	0.950623	0.280935	0.431068	Biso	1.000000	H
O1707	1.0	0.203828	0.387462	0.891341	Biso	1.000000	O
H1023	1.0	0.213318	0.363762	0.905134	Biso	1.000000	H
H1024	1.0	0.209461	0.409346	0.909461	Biso	1.000000	H
H1025	1.0	0.222470	0.392421	0.870508	Biso	1.000000	H
O1708	1.0	0.115407	0.785834	0.825744	Biso	1.000000	O
H1026	1.0	0.129672	0.786784	0.801164	Biso	1.000000	H
H1027	1.0	0.128684	0.765460	0.841249	Biso	1.000000	H
H1028	1.0	0.088874	0.777139	0.821224	Biso	1.000000	H
O1709	1.0	0.087921	0.786443	0.087165	Biso	1.000000	O
H1029	1.0	0.109914	0.786597	0.104990	Biso	1.000000	H
H1030	1.0	0.067224	0.799081	0.102112	Biso	1.000000	H
H1031	1.0	0.095125	0.806489	0.067661	Biso	1.000000	H
O1710	1.0	0.741608	0.173610	0.485722	Biso	1.000000	O
H1032	1.0	0.714944	0.179992	0.492532	Biso	1.000000	H
H1033	1.0	0.754782	0.191356	0.504102	Biso	1.000000	H
H1034	1.0	0.745241	0.146402	0.495727	Biso	1.000000	H
O1711	1.0	0.597710	0.074982	0.842949	Biso	1.000000	O
H1035	1.0	0.609834	0.060877	0.821159	Biso	1.000000	H
H1036	1.0	0.613786	0.098999	0.844784	Biso	1.000000	H
H1037	1.0	0.573331	0.082378	0.830593	Biso	1.000000	H
O1712	1.0	0.665047	0.715290	0.700471	Biso	1.000000	O
H1038	1.0	0.661940	0.743319	0.692665	Biso	1.000000	H
H1039	1.0	0.692875	0.710882	0.701635	Biso	1.000000	H
H1040	1.0	0.656696	0.700137	0.677556	Biso	1.000000	H
Na1	1.0	0.532204	0.157760	0.562165	Biso	1.000000	Na
Na2	1.0	0.345249	0.732269	0.453898	Biso	1.000000	Na
Na3	1.0	0.089187	0.013973	0.055093	Biso	1.000000	Na
Na4	1.0	0.622009	0.553184	0.354680	Biso	1.000000	Na
Na5	1.0	0.598272	0.738453	0.140140	Biso	1.000000	Na
Na6	1.0	0.683658	0.682986	0.922951	Biso	1.000000	Na
Na7	1.0	0.993862	0.010578	0.533308	Biso	1.000000	Na
Na8	1.0	0.041685	0.154391	0.702573	Biso	1.000000	Na
Na9	1.0	0.586873	0.997141	0.378636	Biso	1.000000	Na
Na10	1.0	0.676365	0.139424	0.726822	Biso	1.000000	Na
Na11	1.0	0.100848	0.669500	0.162007	Biso	1.000000	Na
Na12	1.0	0.588419	0.356529	0.445948	Biso	1.000000	Na
Na13	1.0	0.106337	0.832057	0.377961	Biso	1.000000	Na
Na14	1.0	0.299973	0.987723	0.387832	Biso	1.000000	Na
Na15	1.0	0.434136	0.276108	0.699703	Biso	1.000000	Na
Na16	1.0	0.102758	0.674746	0.395061	Biso	1.000000	Na
Na17	1.0	0.415234	0.157854	0.343798	Biso	1.000000	Na

Na18	1.0	0.185208	0.204502	0.865219	Biso	1.000000	Na
Na19	1.0	0.709231	0.422054	0.655623	Biso	1.000000	Na
Na20	1.0	0.521792	0.668755	0.954807	Biso	1.000000	Na
Na21	1.0	0.556795	0.119360	0.006771	Biso	1.000000	Na
Na22	1.0	0.339681	0.691430	0.977174	Biso	1.000000	Na
Na23	1.0	0.475462	0.563329	0.564006	Biso	1.000000	Na
Na24	1.0	0.450897	0.498311	0.087984	Biso	1.000000	Na
Na25	1.0	0.205717	0.193878	0.410545	Biso	1.000000	Na
Na26	1.0	0.977548	0.593562	0.619959	Biso	1.000000	Na
Na27	1.0	0.914561	0.636008	0.533425	Biso	1.000000	Na
Na28	1.0	0.313300	0.814047	0.017820	Biso	1.000000	Na
H1041	1.0	0.059476	0.113058	0.773860	Biso	1.000000	H
H1042	1.0	0.064186	0.078876	0.673789	Biso	1.000000	H
H1043	1.0	0.161111	0.140020	0.818219	Biso	1.000000	H
H1044	1.0	0.146415	0.006503	0.750922	Biso	1.000000	H
H1045	1.0	0.172108	0.192445	0.692515	Biso	1.000000	H
H1046	1.0	0.117188	0.077140	0.630140	Biso	1.000000	H
H1047	1.0	0.192789	0.019651	0.678635	Biso	1.000000	H
O1713	1.0	0.198383	0.406473	0.201525	Biso	1.000000	O
H1048	1.0	0.173584	0.398849	0.188335	Biso	1.000000	H
H1049	1.0	0.218611	0.411981	0.181245	Biso	1.000000	H
O1714	1.0	0.712789	0.246017	0.310330	Biso	1.000000	O
H1050	1.0	0.729386	0.257765	0.289199	Biso	1.000000	H
H1051	1.0	0.726812	0.247465	0.335936	Biso	1.000000	H
O1715	1.0	0.089219	0.079173	0.802164	Biso	1.000000	O
H1052	1.0	0.081424	0.050524	0.797579	Biso	1.000000	H
H1053	1.0	0.095887	0.082992	0.830449	Biso	1.000000	H
O1716	1.0	0.258914	0.462429	0.687154	Biso	1.000000	O
H1054	1.0	0.257089	0.481054	0.664177	Biso	1.000000	H
H1055	1.0	0.268140	0.477750	0.710611	Biso	1.000000	H
O1717	1.0	0.907684	0.452106	0.445584	Biso	1.000000	O
H1056	1.0	0.915131	0.476604	0.430256	Biso	1.000000	H
H1057	1.0	0.928470	0.445763	0.465040	Biso	1.000000	H
O1718	1.0	0.201507	0.413787	0.446549	Biso	1.000000	O
H1058	1.0	0.178881	0.395997	0.440601	Biso	1.000000	H
H1059	1.0	0.224958	0.396796	0.451312	Biso	1.000000	H
O1719	1.0	0.516942	0.512333	0.075916	Biso	1.000000	O
H1060	1.0	0.523214	0.484588	0.085471	Biso	1.000000	H
H1061	1.0	0.539680	0.530434	0.080337	Biso	1.000000	H
O1720	1.0	0.136150	0.989815	0.096885	Biso	1.000000	O
H1062	1.0	0.150186	0.988398	0.122489	Biso	1.000000	H
H1063	1.0	0.130886	0.961895	0.087194	Biso	1.000000	H
O1721	1.0	0.464570	0.960119	0.251875	Biso	1.000000	O
H1064	1.0	0.451233	0.956272	0.226125	Biso	1.000000	H
H1065	1.0	0.487597	0.978009	0.248247	Biso	1.000000	H
O1722	1.0	0.519363	0.681159	0.886094	Biso	1.000000	O
H1066	1.0	0.547949	0.676636	0.884514	Biso	1.000000	H
H1067	1.0	0.512831	0.707819	0.874116	Biso	1.000000	H
O1723	1.0	0.545231	0.862140	0.967120	Biso	1.000000	O
H1068	1.0	0.551259	0.867726	0.995273	Biso	1.000000	H
H1069	1.0	0.569329	0.865019	0.951114	Biso	1.000000	H
O1724	1.0	0.452027	0.696194	0.828371	Biso	1.000000	O
H1070	1.0	0.457041	0.689841	0.800167	Biso	1.000000	H
H1071	1.0	0.471061	0.716836	0.837599	Biso	1.000000	H
O1725	1.0	0.605713	0.999004	0.053676	Biso	1.000000	O
H1072	1.0	0.591442	0.992342	0.078346	Biso	1.000000	H
H1073	1.0	0.618688	0.974198	0.043425	Biso	1.000000	H
O1726	1.0	0.073896	0.030839	0.897096	Biso	1.000000	O
H1074	1.0	0.086764	0.003837	0.896556	Biso	1.000000	H
H1075	1.0	0.092364	0.051380	0.907623	Biso	1.000000	H
O1727	1.0	0.352655	0.517848	0.517408	Biso	1.000000	O
H1076	1.0	0.365690	0.518330	0.491236	Biso	1.000000	H
H1077	1.0	0.352516	0.489653	0.527791	Biso	1.000000	H
O1728	1.0	0.783493	0.195093	0.882072	Biso	1.000000	O
H1078	1.0	0.781764	0.169923	0.898113	Biso	1.000000	H
H1079	1.0	0.771960	0.218073	0.897028	Biso	1.000000	H
O1729	1.0	0.518569	0.050916	0.161924	Biso	1.000000	O
H1080	1.0	0.516748	0.035557	0.136766	Biso	1.000000	H
H1081	1.0	0.517009	0.031744	0.184492	Biso	1.000000	H
O1730	1.0	0.062300	0.298590	0.944544	Biso	1.000000	O
H1082	1.0	0.091120	0.297097	0.946960	Biso	1.000000	H
H1083	1.0	0.050078	0.282610	0.966105	Biso	1.000000	H
O1731	1.0	0.520837	0.164275	0.821971	Biso	1.000000	O

H1084	1.0	0.495093	0.172330	0.811078	Biso	1.000000	H
H1085	1.0	0.526700	0.135855	0.814194	Biso	1.000000	H
O1732	1.0	0.168518	0.518755	0.674361	Biso	1.000000	O
H1086	1.0	0.148043	0.521918	0.694861	Biso	1.000000	H
H1087	1.0	0.183607	0.493575	0.679511	Biso	1.000000	H
O1733	1.0	0.875110	0.257630	0.099582	Biso	1.000000	O
H1088	1.0	0.859130	0.245292	0.078283	Biso	1.000000	H
H1089	1.0	0.859510	0.259078	0.124237	Biso	1.000000	H
O1734	1.0	0.841312	0.693224	0.759889	Biso	1.000000	O
H1090	1.0	0.828778	0.712389	0.741154	Biso	1.000000	H
H1091	1.0	0.870081	0.694752	0.756928	Biso	1.000000	H
O1735	1.0	0.977823	0.622707	0.556540	Biso	1.000000	O
H1092	1.0	0.990401	0.646419	0.569409	Biso	1.000000	H
H1093	1.0	0.994058	0.613557	0.533956	Biso	1.000000	H
O1736	1.0	0.766488	0.478459	0.881735	Biso	1.000000	O
H1094	1.0	0.739691	0.484105	0.872085	Biso	1.000000	H
H1095	1.0	0.765087	0.462670	0.906667	Biso	1.000000	H
O1737	1.0	0.944934	0.212998	0.101610	Biso	1.000000	O
H1096	1.0	0.962150	0.221962	0.123507	Biso	1.000000	H
H1097	1.0	0.920120	0.228508	0.102525	Biso	1.000000	H
O1738	1.0	0.516421	0.787438	0.948150	Biso	1.000000	O
H1098	1.0	0.514756	0.794722	0.919753	Biso	1.000000	H
H1099	1.0	0.525707	0.811264	0.963455	Biso	1.000000	H
O1739	1.0	0.653010	0.356360	0.424003	Biso	1.000000	O
H1100	1.0	0.665162	0.383726	0.423994	Biso	1.000000	H
H1101	1.0	0.673278	0.335531	0.418715	Biso	1.000000	H
O1740	1.0	0.768318	0.095266	0.799373	Biso	1.000000	O
H1102	1.0	0.783282	0.116121	0.814159	Biso	1.000000	H
H1103	1.0	0.778194	0.067842	0.806326	Biso	1.000000	H
O1741	1.0	0.042580	0.006181	0.583269	Biso	1.000000	O
H1104	1.0	0.064830	0.021286	0.571599	Biso	1.000000	H
H1105	1.0	0.034615	0.019357	0.608374	Biso	1.000000	H
O1742	1.0	0.883727	0.895870	0.846777	Biso	1.000000	O
H1106	1.0	0.860882	0.914046	0.843315	Biso	1.000000	H
H1107	1.0	0.886681	0.878441	0.823044	Biso	1.000000	H
O1743	1.0	0.017604	0.842115	0.624171	Biso	1.000000	O
H1108	1.0	0.994571	0.850427	0.639990	Biso	1.000000	H
H1109	1.0	0.041794	0.848121	0.639186	Biso	1.000000	H
O1744	1.0	0.953383	0.461283	0.076556	Biso	1.000000	O
H1110	1.0	0.949025	0.490162	0.083723	Biso	1.000000	H
H1111	1.0	0.928479	0.446097	0.078929	Biso	1.000000	H
O1745	1.0	0.898859	0.024889	0.303971	Biso	1.000000	O
H1112	1.0	0.923623	0.021577	0.318816	Biso	1.000000	H
H1113	1.0	0.890141	0.053588	0.305637	Biso	1.000000	H
O1746	1.0	0.953441	0.540106	0.112753	Biso	1.000000	O
H1114	1.0	0.946596	0.525535	0.137463	Biso	1.000000	H
H1115	1.0	0.976190	0.558069	0.117635	Biso	1.000000	H
O1747	1.0	0.212632	0.956818	0.628032	Biso	1.000000	O
H1116	1.0	0.202166	0.962148	0.654867	Biso	1.000000	H
H1117	1.0	0.193979	0.967110	0.607969	Biso	1.000000	H
O1748	1.0	0.698819	0.000292	0.275412	Biso	1.000000	O
H1118	1.0	0.679993	0.979703	0.265651	Biso	1.000000	H
H1119	1.0	0.725825	0.990135	0.271630	Biso	1.000000	H
O1749	1.0	0.148525	0.342010	0.250727	Biso	1.000000	O
H1120	1.0	0.166147	0.334005	0.228807	Biso	1.000000	H
H1121	1.0	0.131982	0.365137	0.242176	Biso	1.000000	H
O1750	1.0	0.884619	0.177824	0.904008	Biso	1.000000	O
H1122	1.0	0.873315	0.153917	0.917719	Biso	1.000000	H
H1123	1.0	0.912867	0.179754	0.910170	Biso	1.000000	H
O1751	1.0	0.510758	0.063923	0.003152	Biso	1.000000	O
H1124	1.0	0.523639	0.038190	0.995201	Biso	1.000000	H
H1125	1.0	0.509087	0.065234	0.032390	Biso	1.000000	H
O1752	1.0	0.290351	0.542645	0.562740	Biso	1.000000	O
H1126	1.0	0.312756	0.532982	0.546722	Biso	1.000000	H
H1127	1.0	0.292477	0.572437	0.566677	Biso	1.000000	H
O1753	1.0	0.129262	0.740711	0.405925	Biso	1.000000	O
H1128	1.0	0.138596	0.745115	0.433342	Biso	1.000000	H
H1129	1.0	0.145487	0.756819	0.387371	Biso	1.000000	H
O1754	1.0	0.721615	0.829025	0.672705	Biso	1.000000	O
H1130	1.0	0.725723	0.816986	0.646152	Biso	1.000000	H
H1131	1.0	0.718855	0.858929	0.670236	Biso	1.000000	H
O1755	1.0	0.198632	0.242588	0.466738	Biso	1.000000	O
H1132	1.0	0.175167	0.238409	0.483426	Biso	1.000000	H
H1133	1.0	0.201255	0.271869	0.460288	Biso	1.000000	H
O1756	1.0	0.417831	0.298226	0.952893	Biso	1.000000	O
H1134	1.0	0.398400	0.298582	0.974621	Biso	1.000000	H
H1135	1.0	0.411786	0.275594	0.934519	Biso	1.000000	H
O1757	1.0	0.665142	0.162272	0.081711	Biso	1.000000	O

H1136	1.0	0.668342	0.185497	0.063302	Biso	1.000000	H
H1137	1.0	0.689400	0.158696	0.097330	Biso	1.000000	H
O1758	1.0	0.599621	0.153651	0.964285	Biso	1.000000	O
H1138	1.0	0.619837	0.132744	0.959070	Biso	1.000000	H
H1139	1.0	0.610691	0.181045	0.959357	Biso	1.000000	H
O1759	1.0	0.381705	0.218715	0.323750	Biso	1.000000	O
H1140	1.0	0.385663	0.233510	0.298522	Biso	1.000000	H
H1141	1.0	0.360571	0.231997	0.339070	Biso	1.000000	H
O1760	1.0	0.244197	0.020579	0.366277	Biso	1.000000	O
H1142	1.0	0.221501	0.004558	0.375690	Biso	1.000000	H
H1143	1.0	0.241611	0.049114	0.375367	Biso	1.000000	H
O1761	1.0	0.439701	0.960303	0.176390	Biso	1.000000	O
H1144	1.0	0.424196	0.985561	0.173263	Biso	1.000000	H
H1145	1.0	0.460438	0.959435	0.155951	Biso	1.000000	H
O1762	1.0	0.005619	0.211865	0.731524	Biso	1.000000	O
H1146	1.0	0.981332	0.222341	0.719242	Biso	1.000000	H
H1147	1.0	0.000620	0.205311	0.759687	Biso	1.000000	H

```
#======================================================================

# CRYSTAL DATA

#----------------------------------------------------------------------

data_VESTA_phase_1


_chemical_name_common                  'wc18'
_cell_length_a                         36.389170
_cell_length_b                         35.214709
_cell_length_c                         36.687110
_cell_angle_alpha                      90
_cell_angle_beta                       90
_cell_angle_gamma                      90
_space_group_name_H-M_alt              'P 1'
_space_group_IT_number                 1

loop_
_space_group_symop_operation_xyz
   'x, y, z'

loop_
   _atom_site_label
   _atom_site_occupancy
   _atom_site_fract_x
   _atom_site_fract_y
   _atom_site_fract_z
   _atom_site_adp_type
   _atom_site_B_iso_or_equiv
   _atom_site_type_symbol
   Si1        1.0     0.037671      0.977215      0.463018     Biso  1.000000 Si
   Al1        1.0     0.284582      0.973163      0.636534     Biso  1.000000 Al
   Si2        1.0     0.339302      0.115484      0.788416     Biso  1.000000 Si
   Al2        1.0     0.028873      0.645531      0.540871     Biso  1.000000 Al
   Al3        1.0     0.745752      0.576509      0.150205     Biso  1.000000 Al
   Si3        1.0     0.103484      0.238848      0.346389     Biso  1.000000 Si
   Si4        1.0     0.324654      0.167247      0.557771     Biso  1.000000 Si
   Si5        1.0     0.230644      0.753864      0.045702     Biso  1.000000 Si
   Si6        1.0     0.099329      0.915933      0.467862     Biso  1.000000 Si
   Al4        1.0     0.256261      0.217350      0.567131     Biso  1.000000 Al
   Si7        1.0     0.037214      0.682130      0.064715     Biso  1.000000 Si
   Si8        1.0     0.080168      0.861501      0.788958     Biso  1.000000 Si
   Si9        1.0     0.078453      0.151991      0.662257     Biso  1.000000 Si
  Si10        1.0     0.896484      0.436911      0.064708     Biso  1.000000 Si
  Si11        1.0     0.981817      0.943396      0.120458     Biso  1.000000 Si
  Si12        1.0     0.140320      0.679716      0.759861     Biso  1.000000 Si
   Al5        1.0     0.049033      0.354707      0.745889     Biso  1.000000 Al
  Si13        1.0     0.771183      0.124400      0.218482     Biso  1.000000 Si
  Si14        1.0     0.889971      0.136119      0.927293     Biso  1.000000 Si
  Si15        1.0     0.723199      0.344157      0.357213     Biso  1.000000 Si
  Si16        1.0     0.520342      0.865655      0.112945     Biso  1.000000 Si
  Si17        1.0     0.252247      0.888390      0.083143     Biso  1.000000 Si
  Si18        1.0     0.261594      0.671055      0.546216     Biso  1.000000 Si
  Si19        1.0     0.203207      0.681786      0.702089     Biso  1.000000 Si
  Si20        1.0     0.859363      0.630606      0.270472     Biso  1.000000 Si
  Si21        1.0     0.494276      0.332338      0.907924     Biso  1.000000 Si
  Si22        1.0     0.409226      0.347125      0.260435     Biso  1.000000 Si
  Si23        1.0     0.877813      0.833416      0.535594     Biso  1.000000 Si
  Si24        1.0     0.219143      0.360943      0.659098     Biso  1.000000 Si
  Si25        1.0     0.421774      0.332696      0.865792     Biso  1.000000 Si
   Al6        1.0     0.360929      0.152417      0.862823     Biso  1.000000 Al
  Si26        1.0     0.339244      0.290708      0.445006     Biso  1.000000 Si
  Si27        1.0     0.821945      0.809858      0.374409     Biso  1.000000 Si
  Si28        1.0     0.821948      0.442114      0.948848     Biso  1.000000 Si
```

Si29	1.0	0.268978	0.310314	0.245916	Biso	1.000000	Si
Si30	1.0	0.150378	0.037676	0.415064	Biso	1.000000	Si
Al7	1.0	0.060090	0.015411	0.322091	Biso	1.000000	Al
Si31	1.0	0.330079	0.778609	0.360745	Biso	1.000000	Si
Si32	1.0	0.209175	0.479751	0.147319	Biso	1.000000	Si
Si33	1.0	0.407201	0.694832	0.776259	Biso	1.000000	Si
Si34	1.0	0.858302	0.659088	0.632519	Biso	1.000000	Si
Si35	1.0	0.896761	0.195720	0.081398	Biso	1.000000	Si
Si36	1.0	0.896385	0.586831	0.664997	Biso	1.000000	Si
Al8	1.0	0.027511	0.826680	0.846085	Biso	1.000000	Al
Si37	1.0	0.521106	0.089242	0.895415	Biso	1.000000	Si
Al9	1.0	0.208610	0.021414	0.073109	Biso	1.000000	Al
Al10	1.0	0.530773	0.114297	0.690728	Biso	1.000000	Al
Si38	1.0	0.379822	0.604469	0.394662	Biso	1.000000	Si
Al11	1.0	0.759240	0.902856	0.002615	Biso	1.000000	Al
Si39	1.0	0.620415	0.142475	0.982975	Biso	1.000000	Si
Si40	1.0	0.681752	0.355868	0.836874	Biso	1.000000	Si
Si41	1.0	0.535734	0.353375	0.094833	Biso	1.000000	Si
Si42	1.0	0.008210	0.032421	0.519114	Biso	1.000000	Si
Si43	1.0	0.438941	0.269183	0.288532	Biso	1.000000	Si
Si44	1.0	0.738453	0.718578	0.453941	Biso	1.000000	Si
Si45	1.0	0.322324	0.653167	0.354263	Biso	1.000000	Si
Si46	1.0	0.174519	0.746702	0.802325	Biso	1.000000	Si
Si47	1.0	0.514700	0.652085	0.137749	Biso	1.000000	Si
Si48	1.0	0.534461	0.539448	0.980355	Biso	1.000000	Si
Al12	1.0	0.614960	0.711782	0.607467	Biso	1.000000	Al
Al13	1.0	0.765596	0.271147	0.369699	Biso	1.000000	Al
Si49	1.0	0.236571	0.732827	0.301523	Biso	1.000000	Si
Si50	1.0	0.088170	0.315226	0.085001	Biso	1.000000	Si
Si51	1.0	0.667726	0.478701	0.195141	Biso	1.000000	Si
Si52	1.0	0.067571	0.096329	0.527976	Biso	1.000000	Si
Si53	1.0	0.205174	0.850789	0.877030	Biso	1.000000	Si
Si54	1.0	0.119601	0.282758	0.817339	Biso	1.000000	Si
Si55	1.0	0.829860	0.695462	0.384511	Biso	1.000000	Si
Si56	1.0	0.143233	0.449150	0.572471	Biso	1.000000	Si
Si57	1.0	0.656709	0.579332	0.025205	Biso	1.000000	Si
Si58	1.0	0.214436	0.263292	0.946711	Biso	1.000000	Si
Si59	1.0	0.840723	0.458754	0.452418	Biso	1.000000	Si
Al14	1.0	0.938944	0.514893	0.260748	Biso	1.000000	Al
Si60	1.0	0.790983	0.048301	0.657539	Biso	1.000000	Si
Si61	1.0	0.837018	0.084383	0.026057	Biso	1.000000	Si
Si62	1.0	0.632850	0.954133	0.385792	Biso	1.000000	Si
Si63	1.0	0.193974	0.835855	0.203913	Biso	1.000000	Si
Si64	1.0	0.461319	0.778896	0.179863	Biso	1.000000	Si
Si65	1.0	0.253504	0.063656	0.523950	Biso	1.000000	Si
Si66	1.0	0.165330	0.395085	0.366908	Biso	1.000000	Si
Si67	1.0	0.951371	0.241186	0.639110	Biso	1.000000	Si
Si68	1.0	0.654291	0.595635	0.435186	Biso	1.000000	Si
Si69	1.0	0.353133	0.557560	0.021987	Biso	1.000000	Si
Si70	1.0	0.462825	0.583410	0.120601	Biso	1.000000	Si
Si71	1.0	0.470679	0.660732	0.479217	Biso	1.000000	Si
Si72	1.0	0.246130	0.121363	0.223431	Biso	1.000000	Si
Si73	1.0	0.626293	0.696172	0.687045	Biso	1.000000	Si
Si74	1.0	0.113844	0.394753	0.713635	Biso	1.000000	Si
Si75	1.0	0.324410	0.166143	0.719114	Biso	1.000000	Si
Si76	1.0	0.922170	0.915274	0.065771	Biso	1.000000	Si
Si77	1.0	0.726964	0.384581	0.434365	Biso	1.000000	Si
Si78	1.0	0.028134	0.948382	0.684535	Biso	1.000000	Si
Al15	1.0	0.382625	0.807893	0.816115	Biso	1.000000	Al
Si79	1.0	0.606120	0.941368	0.261175	Biso	1.000000	Si
Si80	1.0	0.620503	0.367045	0.094436	Biso	1.000000	Si
Si81	1.0	0.762131	0.703902	0.330511	Biso	1.000000	Si
Si82	1.0	0.531669	0.060508	0.516762	Biso	1.000000	Si
Si83	1.0	0.350159	0.879596	0.842140	Biso	1.000000	Si
Si84	1.0	0.797320	0.588359	0.500208	Biso	1.000000	Si
Si85	1.0	0.000190	0.268497	0.838474	Biso	1.000000	Si
Si86	1.0	0.154975	0.894231	0.820659	Biso	1.000000	Si
Si87	1.0	0.119277	0.602640	0.795694	Biso	1.000000	Si
Si88	1.0	0.163877	0.305940	0.545401	Biso	1.000000	Si
Si89	1.0	0.240270	0.179578	0.704340	Biso	1.000000	Si
Si90	1.0	0.542150	0.232759	0.192429	Biso	1.000000	Si
Si91	1.0	0.290155	0.561606	0.226392	Biso	1.000000	Si
Si92	1.0	0.715652	0.756295	0.839567	Biso	1.000000	Si
Al16	1.0	0.948612	0.570299	0.093548	Biso	1.000000	Al
Al17	1.0	0.185190	0.854200	0.515437	Biso	1.000000	Al

Si93	1.0	0.815770	0.494753	0.235388	Biso	1.000000	Si
Si94	1.0	0.200790	0.200220	0.629690	Biso	1.000000	Si
Si95	1.0	0.663524	0.790258	0.783422	Biso	1.000000	Si
Si96	1.0	0.446152	0.525011	0.519261	Biso	1.000000	Si
Si97	1.0	0.637071	0.779402	0.429985	Biso	1.000000	Si
Si98	1.0	0.909251	0.869568	0.711705	Biso	1.000000	Si
Al18	1.0	0.864307	0.526709	0.615186	Biso	1.000000	Al
Al19	1.0	0.226029	0.878380	0.955026	Biso	1.000000	Al
Si99	1.0	0.041936	0.307681	0.013593	Biso	1.000000	Si
Si100	1.0	0.674566	0.148934	0.870180	Biso	1.000000	Si
Si101	1.0	0.700250	0.129462	0.012140	Biso	1.000000	Si
Si102	1.0	0.956994	0.746035	0.266592	Biso	1.000000	Si
Si103	1.0	0.107164	0.461850	0.065769	Biso	1.000000	Si
Si104	1.0	0.832448	0.686957	0.087901	Biso	1.000000	Si
Si105	1.0	0.456853	0.131336	0.372992	Biso	1.000000	Si
Si106	1.0	0.574825	0.754457	0.982240	Biso	1.000000	Si
Si107	1.0	0.752430	0.785896	0.022583	Biso	1.000000	Si
Si108	1.0	0.437251	0.560808	0.042888	Biso	1.000000	Si
Si109	1.0	0.733762	0.626324	0.892477	Biso	1.000000	Si
Si110	1.0	0.208821	0.702712	0.183744	Biso	1.000000	Si
Si111	1.0	0.409023	0.409176	0.478023	Biso	1.000000	Si
Si112	1.0	0.053697	0.336553	0.827492	Biso	1.000000	Si
Si113	1.0	0.570403	0.139099	0.050773	Biso	1.000000	Si
Si114	1.0	0.284346	0.057504	0.820637	Biso	1.000000	Si
Al20	1.0	0.573355	0.835387	0.433174	Biso	1.000000	Al
Al21	1.0	0.011972	0.011532	0.939733	Biso	1.000000	Al
Si115	1.0	0.771966	0.004425	0.464986	Biso	1.000000	Si
Si116	1.0	0.981480	0.876651	0.666103	Biso	1.000000	Si
Al22	1.0	0.185239	0.480251	0.371381	Biso	1.000000	Al
Si117	1.0	0.289383	0.056872	0.654688	Biso	1.000000	Si
Si118	1.0	0.936177	0.433137	0.272755	Biso	1.000000	Si
Si119	1.0	0.418281	0.390323	0.124410	Biso	1.000000	Si
Si120	1.0	0.269726	0.600945	0.595623	Biso	1.000000	Si
Si121	1.0	0.938502	0.093526	0.259988	Biso	1.000000	Si
Si122	1.0	0.065560	0.033964	0.402828	Biso	1.000000	Si
Si123	1.0	0.317751	0.080816	0.578279	Biso	1.000000	Si
Si124	1.0	0.369517	0.905889	0.920958	Biso	1.000000	Si
Si125	1.0	0.244868	0.234345	0.872140	Biso	1.000000	Si
Si126	1.0	0.964975	0.988227	0.191236	Biso	1.000000	Si
Si127	1.0	0.764750	0.876262	0.822869	Biso	1.000000	Si
Al23	1.0	0.080354	0.317328	0.944923	Biso	1.000000	Al
Si128	1.0	0.665690	0.012788	0.682305	Biso	1.000000	Si
Si129	1.0	0.543931	0.043328	0.651109	Biso	1.000000	Si
Si130	1.0	0.317001	0.495043	0.408111	Biso	1.000000	Si
Si131	1.0	0.500267	0.807410	0.246051	Biso	1.000000	Si
Si132	1.0	0.884381	0.562049	0.144515	Biso	1.000000	Si
Si133	1.0	0.259503	0.835938	0.150708	Biso	1.000000	Si
Si134	1.0	0.063693	0.645069	0.339291	Biso	1.000000	Si
Si135	1.0	0.392584	0.327590	0.178958	Biso	1.000000	Si
Si136	1.0	0.566710	0.568078	0.679050	Biso	1.000000	Si
Si137	1.0	0.932308	0.227279	0.011021	Biso	1.000000	Si
Si138	1.0	0.875873	0.778885	0.603029	Biso	1.000000	Si
Si139	1.0	0.127556	0.369334	0.294847	Biso	1.000000	Si
Al24	1.0	0.467345	0.671884	0.972430	Biso	1.000000	Al
Si140	1.0	0.617909	0.587240	0.879178	Biso	1.000000	Si
Si141	1.0	0.539573	0.576785	0.576374	Biso	1.000000	Si
Si142	1.0	0.718494	0.073640	0.876861	Biso	1.000000	Si
Si143	1.0	0.696963	0.999772	0.500056	Biso	1.000000	Si
Si144	1.0	0.311964	0.283305	0.849713	Biso	1.000000	Si
Si145	1.0	0.849632	0.606062	0.821725	Biso	1.000000	Si
Si146	1.0	0.207621	0.608004	0.654238	Biso	1.000000	Si
Si147	1.0	0.420801	0.609018	0.524457	Biso	1.000000	Si
Si148	1.0	0.807328	0.377589	0.464992	Biso	1.000000	Si
Si149	1.0	0.088981	0.976371	0.948753	Biso	1.000000	Si
Si150	1.0	0.618272	0.471008	0.594789	Biso	1.000000	Si
Si151	1.0	0.201426	0.879243	0.630527	Biso	1.000000	Si
Si152	1.0	0.726799	0.629351	0.212728	Biso	1.000000	Si
Si153	1.0	0.728085	0.910631	0.147070	Biso	1.000000	Si
Si154	1.0	0.599263	0.999195	0.081726	Biso	1.000000	Si
Si155	1.0	0.440222	0.870156	0.080549	Biso	1.000000	Si
Si156	1.0	0.945955	0.414080	0.657713	Biso	1.000000	Si
Si157	1.0	0.211220	0.838192	0.025798	Biso	1.000000	Si
Si158	1.0	0.336823	0.153834	0.474270	Biso	1.000000	Si
Si159	1.0	0.802827	0.084945	0.776957	Biso	1.000000	Si
Al25	1.0	0.038746	0.229439	0.292615	Biso	1.000000	Al
Si160	1.0	0.387181	0.466725	0.536954	Biso	1.000000	Si
Si161	1.0	0.636118	0.732231	0.211013	Biso	1.000000	Si
Al26	1.0	0.424206	0.520757	0.633315	Biso	1.000000	Al

Si162	1.0	0.143190	0.435216	0.852978	Biso	1.000000	Si
Si163	1.0	0.585949	0.177275	0.669009	Biso	1.000000	Si
Si164	1.0	0.422340	0.009760	0.016072	Biso	1.000000	Si
Si165	1.0	0.956326	0.961743	0.767490	Biso	1.000000	Si
Si166	1.0	0.035162	0.937614	0.292381	Biso	1.000000	Si
Al27	1.0	0.233597	0.416462	0.097249	Biso	1.000000	Al
Si167	1.0	0.804918	0.303839	0.926298	Biso	1.000000	Si
Si168	1.0	0.140770	0.262800	0.616797	Biso	1.000000	Si
Si169	1.0	0.448150	0.898795	0.524010	Biso	1.000000	Si
Si170	1.0	0.719349	0.172872	0.264734	Biso	1.000000	Si
Al28	1.0	0.497354	0.415667	0.896623	Biso	1.000000	Al
Si171	1.0	0.445575	0.936467	0.286757	Biso	1.000000	Si
Si172	1.0	0.762488	0.377871	0.943225	Biso	1.000000	Si
Si173	1.0	0.506239	0.240862	0.996004	Biso	1.000000	Si
Si174	1.0	0.466122	0.993561	0.944299	Biso	1.000000	Si
Al29	1.0	0.001030	0.661076	0.948172	Biso	1.000000	Al
Si175	1.0	0.496189	0.506535	0.149301	Biso	1.000000	Si
Si176	1.0	0.323772	0.096291	0.060637	Biso	1.000000	Si
Si177	1.0	0.547166	0.578023	0.491058	Biso	1.000000	Si
Si178	1.0	0.876481	0.214068	0.823578	Biso	1.000000	Si
Si179	1.0	0.637816	0.421335	0.252716	Biso	1.000000	Si
Si180	1.0	0.172452	0.725276	0.941941	Biso	1.000000	Si
Al30	1.0	0.075528	0.209345	0.601075	Biso	1.000000	Al
Al31	1.0	0.618885	0.557196	0.583987	Biso	1.000000	Al
Si181	1.0	0.873235	0.385012	0.304491	Biso	1.000000	Si
Al32	1.0	0.386178	0.464837	0.101439	Biso	1.000000	Al
Si182	1.0	0.194270	0.316595	0.796808	Biso	1.000000	Si
Si183	1.0	0.522914	0.307017	0.444693	Biso	1.000000	Si
Si184	1.0	0.000320	0.196647	0.966506	Biso	1.000000	Si
Si185	1.0	0.050685	0.074009	0.684867	Biso	1.000000	Si
Si186	1.0	0.686113	0.124026	0.588899	Biso	1.000000	Si
Si187	1.0	0.948593	0.043741	0.373973	Biso	1.000000	Si
Si188	1.0	0.432906	0.116422	0.612929	Biso	1.000000	Si
Al33	1.0	0.253654	0.494535	0.942364	Biso	1.000000	Al
Si189	1.0	0.568557	0.746677	0.845826	Biso	1.000000	Si
Si190	1.0	0.545432	0.053942	0.043845	Biso	1.000000	Si
Si191	1.0	0.837966	0.131214	0.097411	Biso	1.000000	Si
Si192	1.0	0.852218	0.016944	0.754595	Biso	1.000000	Si
Si193	1.0	0.792829	0.241980	0.527834	Biso	1.000000	Si
Si194	1.0	0.751149	0.349828	0.189789	Biso	1.000000	Si
Si195	1.0	0.298182	0.668272	0.469748	Biso	1.000000	Si
Si196	1.0	0.324761	0.044694	0.386201	Biso	1.000000	Si
Si197	1.0	0.698073	0.669658	0.726879	Biso	1.000000	Si
Si198	1.0	0.465746	0.355777	0.400165	Biso	1.000000	Si
Si199	1.0	0.076841	0.870347	0.020695	Biso	1.000000	Si
Si200	1.0	0.389481	0.125972	0.683237	Biso	1.000000	Si
Si201	1.0	0.297289	0.535217	0.480250	Biso	1.000000	Si
Si202	1.0	0.614081	0.251797	0.699417	Biso	1.000000	Si
Si203	1.0	0.747981	0.559502	0.334586	Biso	1.000000	Si
Si204	1.0	0.308647	0.070741	0.463288	Biso	1.000000	Si
Si205	1.0	0.297212	0.786629	0.637796	Biso	1.000000	Si
Si206	1.0	0.402614	0.480191	0.919279	Biso	1.000000	Si
Al34	1.0	0.555426	0.063594	0.204251	Biso	1.000000	Al
Al35	1.0	0.559309	0.318367	0.710776	Biso	1.000000	Al
Si207	1.0	0.112175	0.919232	0.081776	Biso	1.000000	Si
Si208	1.0	0.407088	0.065574	0.403319	Biso	1.000000	Si
Si209	1.0	0.315440	0.075063	0.202566	Biso	1.000000	Si
Si210	1.0	0.883469	0.799672	0.829417	Biso	1.000000	Si
Si211	1.0	0.492075	0.504823	0.587809	Biso	1.000000	Si
Si212	1.0	0.288476	0.297819	0.561549	Biso	1.000000	Si
Si213	1.0	0.682673	0.697743	0.894478	Biso	1.000000	Si
Si214	1.0	0.870396	0.817562	0.974548	Biso	1.000000	Si
Si215	1.0	0.540724	0.118228	0.356793	Biso	1.000000	Si
Al36	1.0	0.658399	0.355604	0.475829	Biso	1.000000	Al
Si216	1.0	0.020511	0.074310	0.276871	Biso	1.000000	Si
Si217	1.0	0.512545	0.712691	0.075521	Biso	1.000000	Si
Si218	1.0	0.883444	0.008871	0.556961	Biso	1.000000	Si
Si219	1.0	0.108341	0.328695	0.484380	Biso	1.000000	Si
Si220	1.0	0.177187	0.576464	0.850331	Biso	1.000000	Si
Si221	1.0	0.720148	0.266496	0.021847	Biso	1.000000	Si
Si222	1.0	0.117799	0.504471	0.332681	Biso	1.000000	Si

Si223	1.0	0.097687	0.283752	0.735037	Biso	1.000000	Si
Si224	1.0	0.519196	0.784300	0.471495	Biso	1.000000	Si
Si225	1.0	0.279602	0.462619	0.612272	Biso	1.000000	Si
Si226	1.0	0.658567	0.038835	0.377893	Biso	1.000000	Si
Si227	1.0	0.134188	0.257590	0.974026	Biso	1.000000	Si
Si228	1.0	0.070392	0.910358	0.901973	Biso	1.000000	Si
Si229	1.0	0.332764	0.815364	0.008328	Biso	1.000000	Si
Si230	1.0	0.775582	0.129111	0.391846	Biso	1.000000	Si
Si231	1.0	0.700324	0.013685	0.818846	Biso	1.000000	Si
Si232	1.0	0.025526	0.094240	0.829965	Biso	1.000000	Si
Si233	1.0	0.070291	0.137661	0.188726	Biso	1.000000	Si
Si234	1.0	0.306698	0.892332	0.977790	Biso	1.000000	Si
Si235	1.0	0.921870	0.451573	0.143810	Biso	1.000000	Si
Si236	1.0	0.334113	0.526135	0.943290	Biso	1.000000	Si
Si237	1.0	0.041873	0.249071	0.909355	Biso	1.000000	Si
Si238	1.0	0.949263	0.789395	0.409531	Biso	1.000000	Si
Si239	1.0	0.882908	0.650160	0.552974	Biso	1.000000	Si
Si240	1.0	0.382795	0.083599	0.000006	Biso	1.000000	Si
Si241	1.0	0.854688	0.274193	0.193261	Biso	1.000000	Si
Al37	1.0	0.374438	0.529412	0.356428	Biso	1.000000	Al
Si242	1.0	0.800851	0.851726	0.501271	Biso	1.000000	Si
Al38	1.0	0.403109	0.770953	0.691109	Biso	1.000000	Al
Si243	1.0	0.063367	0.539431	0.442390	Biso	1.000000	Si
Al39	1.0	0.111765	0.021845	0.811495	Biso	1.000000	Al
Si244	1.0	0.357117	0.083878	0.917968	Biso	1.000000	Si
Si245	1.0	0.167223	0.155495	0.868346	Biso	1.000000	Si
Si246	1.0	0.756788	0.193007	0.992470	Biso	1.000000	Si
Si247	1.0	0.257784	0.523946	0.553887	Biso	1.000000	Si
Si248	1.0	0.353174	0.600113	0.473867	Biso	1.000000	Si
Si249	1.0	0.274001	0.076972	0.902216	Biso	1.000000	Si
Si250	1.0	0.182998	0.503562	0.517855	Biso	1.000000	Si
Si251	1.0	0.826925	0.131619	0.557234	Biso	1.000000	Si
Si252	1.0	0.711976	0.771263	0.219181	Biso	1.000000	Si
Si253	1.0	0.127437	0.403340	0.441498	Biso	1.000000	Si
Si254	1.0	0.559381	0.924611	0.063876	Biso	1.000000	Si
Si255	1.0	0.616233	0.020300	0.613393	Biso	1.000000	Si
Si256	1.0	0.648473	0.415491	0.652513	Biso	1.000000	Si
Si257	1.0	0.148162	0.069006	0.870543	Biso	1.000000	Si
Si258	1.0	0.311593	0.780787	0.933121	Biso	1.000000	Si
Si259	1.0	0.933066	0.707091	0.515263	Biso	1.000000	Si
Si260	1.0	0.972593	0.546695	0.329007	Biso	1.000000	Si
Si261	1.0	0.465339	0.192823	0.590014	Biso	1.000000	Si
Si262	1.0	0.548232	0.634346	0.374063	Biso	1.000000	Si
Si263	1.0	0.845856	0.981606	0.831916	Biso	1.000000	Si
Si264	1.0	0.799048	0.404402	0.660534	Biso	1.000000	Si
Si265	1.0	0.678636	0.017528	0.927874	Biso	1.000000	Si
Si266	1.0	0.745299	0.498292	0.559853	Biso	1.000000	Si
Si267	1.0	0.329840	0.311600	0.053829	Biso	1.000000	Si
Si268	1.0	0.187716	0.499289	0.889304	Biso	1.000000	Si
Si269	1.0	0.605944	0.315005	0.425275	Biso	1.000000	Si
Al40	1.0	0.635648	0.988776	0.861581	Biso	1.000000	Al
Al41	1.0	0.362649	0.895575	0.398971	Biso	1.000000	Al
Si270	1.0	0.734705	0.193383	0.346560	Biso	1.000000	Si
Si271	1.0	0.477051	0.581995	0.759463	Biso	1.000000	Si
Si272	1.0	0.888954	0.354832	0.192619	Biso	1.000000	Si
Si273	1.0	0.544321	0.679648	0.002359	Biso	1.000000	Si
Si274	1.0	0.647684	0.287491	0.118853	Biso	1.000000	Si
Si275	1.0	0.279410	0.690615	0.740257	Biso	1.000000	Si
Al42	1.0	0.917408	0.000354	0.707660	Biso	1.000000	Al
Si276	1.0	0.233663	0.416721	0.970513	Biso	1.000000	Si
Si277	1.0	0.922791	0.203090	0.500525	Biso	1.000000	Si
Si278	1.0	0.225955	0.144720	0.929558	Biso	1.000000	Si
Si279	1.0	0.995545	0.214469	0.772165	Biso	1.000000	Si
Si280	1.0	0.952393	0.741781	0.587896	Biso	1.000000	Si
Si281	1.0	0.309466	0.305284	0.171631	Biso	1.000000	Si
Si282	1.0	0.850855	0.818624	0.673316	Biso	1.000000	Si
Si283	1.0	0.056719	0.451334	0.454481	Biso	1.000000	Si
Si284	1.0	0.595075	0.083628	0.414295	Biso	1.000000	Si
Si285	1.0	0.641908	0.644617	0.220926	Biso	1.000000	Si
Si286	1.0	0.402614	0.207952	0.238588	Biso	1.000000	Si
Si287	1.0	0.225400	0.944194	0.583491	Biso	1.000000	Si
Al43	1.0	0.218724	0.203268	0.101608	Biso	1.000000	Al
Si288	1.0	0.727134	0.835787	0.959318	Biso	1.000000	Si
Si289	1.0	0.345581	0.343303	0.902025	Biso	1.000000	Si
Al44	1.0	0.569013	0.986696	0.503539	Biso	1.000000	Al
Si290	1.0	0.773535	0.628354	0.286049	Biso	1.000000	Si
Al45	1.0	0.225106	0.443144	0.674995	Biso	1.000000	Al

Si291	1.0	0.844793	0.948004	0.084908	Biso	1.000000	Si
Al46	1.0	0.057954	0.154370	0.013927	Biso	1.000000	Al
Al47	1.0	0.207719	0.109026	0.741100	Biso	1.000000	Al
Si292	1.0	0.651677	0.588476	0.518547	Biso	1.000000	Si
Si293	1.0	0.198546	0.019728	0.347514	Biso	1.000000	Si
Si294	1.0	0.355680	0.115413	0.138036	Biso	1.000000	Si
Si295	1.0	0.170671	0.399864	0.778971	Biso	1.000000	Si
Si296	1.0	0.804410	0.177238	0.854862	Biso	1.000000	Si
Si297	1.0	0.654324	0.057614	0.546086	Biso	1.000000	Si
Si298	1.0	0.565223	0.186607	0.308844	Biso	1.000000	Si
Si299	1.0	0.633158	0.345378	0.602713	Biso	1.000000	Si
Si300	1.0	0.365873	0.289819	0.525413	Biso	1.000000	Si
Si301	1.0	0.808317	0.001777	0.029318	Biso	1.000000	Si
Si302	1.0	0.339040	0.786407	0.520793	Biso	1.000000	Si
Si303	1.0	0.463404	0.438936	0.418834	Biso	1.000000	Si
Si304	1.0	0.244795	0.930441	0.443892	Biso	1.000000	Si
Si305	1.0	0.714229	0.284174	0.799226	Biso	1.000000	Si
Si306	1.0	0.967028	0.730039	0.081306	Biso	1.000000	Si
Al48	1.0	0.730448	0.554788	0.934260	Biso	1.000000	Al
Si307	1.0	0.074303	0.084636	0.765708	Biso	1.000000	Si
Si308	1.0	0.516088	0.221831	0.529638	Biso	1.000000	Si
Si309	1.0	0.034658	0.932150	0.765154	Biso	1.000000	Si
Si310	1.0	0.496271	0.791229	0.987512	Biso	1.000000	Si
Si311	1.0	0.766146	0.627746	0.813081	Biso	1.000000	Si
Si312	1.0	0.154368	0.807515	0.618945	Biso	1.000000	Si
Si313	1.0	0.175697	0.724036	0.636229	Biso	1.000000	Si
Si314	1.0	0.003180	0.579065	0.490894	Biso	1.000000	Si
Si315	1.0	0.430903	0.348726	0.534035	Biso	1.000000	Si
Si316	1.0	0.074848	0.761534	0.163622	Biso	1.000000	Si
Si317	1.0	0.453272	0.731870	0.353508	Biso	1.000000	Si
Si318	1.0	0.836389	0.783051	0.295360	Biso	1.000000	Si
Al49	1.0	0.518924	0.245943	0.270753	Biso	1.000000	Al
Al50	1.0	0.907242	0.315408	0.553175	Biso	1.000000	Al
Si319	1.0	0.800062	0.514390	0.904121	Biso	1.000000	Si
Si320	1.0	0.639866	0.319807	0.347604	Biso	1.000000	Si
Si321	1.0	0.930645	0.623038	0.313854	Biso	1.000000	Si
Si322	1.0	0.505433	0.327093	0.991702	Biso	1.000000	Si
Si323	1.0	0.155525	0.124214	0.633724	Biso	1.000000	Si
Al51	1.0	0.566811	0.493317	0.104050	Biso	1.000000	Al
Al52	1.0	0.869693	0.766981	0.415503	Biso	1.000000	Al
Si324	1.0	0.024193	0.339662	0.492388	Biso	1.000000	Si
Si325	1.0	0.982900	0.022976	0.667259	Biso	1.000000	Si
Si326	1.0	0.386535	0.224335	0.576576	Biso	1.000000	Si
Si327	1.0	0.255351	0.604642	0.332566	Biso	1.000000	Si
Si328	1.0	0.223612	0.965846	0.736379	Biso	1.000000	Si
Si329	1.0	0.454946	0.432725	0.966201	Biso	1.000000	Si
Si330	1.0	0.567015	0.457428	0.870496	Biso	1.000000	Si
Si331	1.0	0.265976	0.206648	0.976355	Biso	1.000000	Si
Si332	1.0	0.157032	0.780557	0.877717	Biso	1.000000	Si
Si333	1.0	0.335548	0.723107	0.683791	Biso	1.000000	Si
Si334	1.0	0.474551	0.815091	0.327060	Biso	1.000000	Si
Si335	1.0	0.551222	0.848478	0.351440	Biso	1.000000	Si
Si336	1.0	0.789100	0.651679	0.445797	Biso	1.000000	Si
Si337	1.0	0.692343	0.911114	0.503468	Biso	1.000000	Si
Si338	1.0	0.612097	0.065613	0.940513	Biso	1.000000	Si
Si339	1.0	0.373749	0.715173	0.322786	Biso	1.000000	Si
Al53	1.0	0.442904	0.143497	0.005754	Biso	1.000000	Al
Si340	1.0	0.524939	0.520740	0.226800	Biso	1.000000	Si
Si341	1.0	0.088477	0.680119	0.133142	Biso	1.000000	Si
Si342	1.0	0.865944	0.571415	0.898604	Biso	1.000000	Si
Si343	1.0	0.258008	0.897313	0.519717	Biso	1.000000	Si
Si344	1.0	0.395739	0.980428	0.141896	Biso	1.000000	Si
Si345	1.0	0.939156	0.638696	0.998577	Biso	1.000000	Si
Si346	1.0	0.247658	0.623106	0.830488	Biso	1.000000	Si
Si347	1.0	0.135135	0.709891	0.526348	Biso	1.000000	Si
Si348	1.0	0.044150	0.222098	0.485478	Biso	1.000000	Si
Si349	1.0	0.920848	0.453342	0.480591	Biso	1.000000	Si
Si350	1.0	0.360236	0.309763	0.977456	Biso	1.000000	Si
Si351	1.0	0.662428	0.266831	0.630243	Biso	1.000000	Si
Si352	1.0	0.663491	0.207019	0.567545	Biso	1.000000	Si
Si353	1.0	0.679254	0.346415	0.233915	Biso	1.000000	Si
Si354	1.0	0.503609	0.672841	0.313999	Biso	1.000000	Si
Si355	1.0	0.554256	0.784507	0.103068	Biso	1.000000	Si
Si356	1.0	0.017584	0.888950	0.964130	Biso	1.000000	Si
Si357	1.0	0.145978	0.322425	0.679138	Biso	1.000000	Si
Si358	1.0	0.811632	0.160701	0.734198	Biso	1.000000	Si
Si359	1.0	0.933745	0.232853	0.720828	Biso	1.000000	Si
Si360	1.0	0.967297	0.884744	0.867106	Biso	1.000000	Si
Si361	1.0	0.610646	0.816468	0.297952	Biso	1.000000	Si
Si362	1.0	0.464721	0.147316	0.239224	Biso	1.000000	Si

Si363	1.0	0.725271	0.444965	0.674986	Biso	1.000000	Si
Si364	1.0	0.953776	0.952136	0.268459	Biso	1.000000	Si
Si365	1.0	0.207650	0.139143	0.149442	Biso	1.000000	Si
Si366	1.0	0.750429	0.494364	0.179340	Biso	1.000000	Si
Si367	1.0	0.541900	0.435548	0.572659	Biso	1.000000	Si
Si368	1.0	0.376942	0.275466	0.645916	Biso	1.000000	Si
Si369	1.0	0.196382	0.454370	0.449937	Biso	1.000000	Si
Si370	1.0	0.573360	0.601189	0.027551	Biso	1.000000	Si
Al54	1.0	0.800021	0.092800	0.861529	Biso	1.000000	Al
Si371	1.0	0.981711	0.703175	0.876605	Biso	1.000000	Si
Si372	1.0	0.188998	0.008210	0.540119	Biso	1.000000	Si
Si373	1.0	0.100823	0.209744	0.862469	Biso	1.000000	Si
Si374	1.0	0.855392	0.267657	0.109109	Biso	1.000000	Si
Si375	1.0	0.557311	0.019961	0.854232	Biso	1.000000	Si
Si376	1.0	0.054414	0.969203	0.839755	Biso	1.000000	Si
Si377	1.0	0.780150	0.888504	0.082255	Biso	1.000000	Si
Si378	1.0	0.053982	0.558960	0.358761	Biso	1.000000	Si
Si379	1.0	0.790221	0.484488	0.316555	Biso	1.000000	Si
Si380	1.0	0.140316	0.090745	0.169134	Biso	1.000000	Si
Al55	1.0	0.724768	0.874387	0.433594	Biso	1.000000	Al
Si381	1.0	0.822984	0.467259	0.030791	Biso	1.000000	Si
Si382	1.0	0.456116	0.736860	0.438781	Biso	1.000000	Si
Si383	1.0	0.782804	0.005904	0.328521	Biso	1.000000	Si
Si384	1.0	0.303665	0.958954	0.388681	Biso	1.000000	Si
Si385	1.0	0.233409	0.859212	0.394700	Biso	1.000000	Si
Si386	1.0	0.783392	0.859598	0.637570	Biso	1.000000	Si
Si387	1.0	0.257374	0.712404	0.938970	Biso	1.000000	Si
Si388	1.0	0.960587	0.306261	0.039582	Biso	1.000000	Si
Si389	1.0	0.977915	0.614357	0.674079	Biso	1.000000	Si
Si390	1.0	0.987665	0.395741	0.137296	Biso	1.000000	Si
Si391	1.0	0.255716	0.190603	0.275173	Biso	1.000000	Si
Si392	1.0	0.273922	0.841745	0.827225	Biso	1.000000	Si
Si393	1.0	0.401427	0.518911	0.224604	Biso	1.000000	Si
Si394	1.0	0.434374	0.551582	0.880985	Biso	1.000000	Si
Si395	1.0	0.623081	0.671552	0.536695	Biso	1.000000	Si
Si396	1.0	0.311829	0.938290	0.046381	Biso	1.000000	Si
Si397	1.0	0.312862	0.909327	0.258050	Biso	1.000000	Si
Si398	1.0	0.556149	0.661630	0.491085	Biso	1.000000	Si
Si399	1.0	0.960474	0.036492	0.585535	Biso	1.000000	Si
Si400	1.0	0.348134	0.227743	0.075971	Biso	1.000000	Si
Si401	1.0	0.293760	0.445021	0.236290	Biso	1.000000	Si
Si402	1.0	0.695737	0.105661	0.093687	Biso	1.000000	Si
Al56	1.0	0.250088	0.707994	0.812743	Biso	1.000000	Al
Si403	1.0	0.507868	0.309871	0.527730	Biso	1.000000	Si
Al57	1.0	0.747459	0.706353	0.779350	Biso	1.000000	Al
Si404	1.0	0.871001	0.566187	0.540470	Biso	1.000000	Si
Si405	1.0	0.070282	0.202436	0.732366	Biso	1.000000	Si
Si406	1.0	0.254618	0.388758	0.449245	Biso	1.000000	Si
Si407	1.0	0.589604	0.503860	0.477579	Biso	1.000000	Si
Si408	1.0	0.144063	0.766350	0.341477	Biso	1.000000	Si
Si409	1.0	0.938925	0.158544	0.804441	Biso	1.000000	Si
Si410	1.0	0.851424	0.034898	0.481139	Biso	1.000000	Si
Si411	1.0	0.548053	0.351410	0.591047	Biso	1.000000	Si
Si412	1.0	0.308814	0.832939	0.214839	Biso	1.000000	Si
Al58	1.0	0.981820	0.214199	0.202119	Biso	1.000000	Al
Si413	1.0	0.494301	0.180817	0.055980	Biso	1.000000	Si
Si414	1.0	0.875513	0.558076	0.228737	Biso	1.000000	Si
Si415	1.0	0.728887	0.608825	0.400555	Biso	1.000000	Si
Si416	1.0	0.964746	0.364961	0.589068	Biso	1.000000	Si
Si417	1.0	0.471748	0.816028	0.535981	Biso	1.000000	Si
Si418	1.0	0.533664	0.043189	0.960328	Biso	1.000000	Si
Si419	1.0	0.743199	0.046383	0.393999	Biso	1.000000	Si
Si420	1.0	0.141190	0.988575	0.744283	Biso	1.000000	Si
Si421	1.0	0.137634	0.567098	0.477399	Biso	1.000000	Si
Si422	1.0	0.854881	0.430405	0.192793	Biso	1.000000	Si
Si423	1.0	0.291180	0.016381	0.079800	Biso	1.000000	Si
Si424	1.0	0.756082	0.706813	0.180681	Biso	1.000000	Si
Si425	1.0	0.762139	0.817340	0.565607	Biso	1.000000	Si
Al59	1.0	0.380795	0.361082	0.420090	Biso	1.000000	Al
Si426	1.0	0.322291	0.512317	0.087950	Biso	1.000000	Si
Si427	1.0	0.792832	0.328150	0.616802	Biso	1.000000	Si
Si428	1.0	0.493191	0.982575	0.341818	Biso	1.000000	Si
Si429	1.0	0.165972	0.513234	0.263607	Biso	1.000000	Si
Si430	1.0	0.776860	0.508089	0.480578	Biso	1.000000	Si
Si431	1.0	0.739267	0.280592	0.663557	Biso	1.000000	Si
Si432	1.0	0.061864	0.150141	0.272761	Biso	1.000000	Si

Si433	1.0	0.905297	0.684640	0.364703	Biso	1.000000	Si
Si434	1.0	0.071497	0.664401	0.256455	Biso	1.000000	Si
Si435	1.0	0.943599	0.927919	0.984240	Biso	1.000000	Si
Si436	1.0	0.414327	0.026863	0.883667	Biso	1.000000	Si
Si437	1.0	0.354493	0.482225	0.649555	Biso	1.000000	Si
Si438	1.0	0.130810	0.063489	0.575883	Biso	1.000000	Si
Si439	1.0	0.458623	0.414230	0.286387	Biso	1.000000	Si
Al60	1.0	0.704141	0.687031	0.382025	Biso	1.000000	Al
Si440	1.0	0.871196	0.761898	0.494367	Biso	1.000000	Si
Si441	1.0	0.282661	0.173900	0.054663	Biso	1.000000	Si
Si442	1.0	0.391361	0.928910	0.579653	Biso	1.000000	Si
Si443	1.0	0.785261	0.550191	0.616988	Biso	1.000000	Si
Si444	1.0	0.764200	0.961431	0.846949	Biso	1.000000	Si
Si445	1.0	0.596768	0.196252	0.515009	Biso	1.000000	Si
Si446	1.0	0.764821	0.226371	0.914193	Biso	1.000000	Si
Si447	1.0	0.408108	0.870034	0.330440	Biso	1.000000	Si
Si448	1.0	0.761317	0.141462	0.134942	Biso	1.000000	Si
Si449	1.0	0.041781	0.860266	0.334188	Biso	1.000000	Si
Al61	1.0	0.245887	0.808247	0.261106	Biso	1.000000	Al
Si450	1.0	0.589271	0.507183	0.322887	Biso	1.000000	Si
Si451	1.0	0.668933	0.734338	0.496861	Biso	1.000000	Si
Si452	1.0	0.875123	0.718109	0.257378	Biso	1.000000	Si
Si453	1.0	0.014982	0.431814	0.298004	Biso	1.000000	Si
Si454	1.0	0.269236	0.340420	0.104134	Biso	1.000000	Si
Si455	1.0	0.394686	0.943009	0.063919	Biso	1.000000	Si
Si456	1.0	0.716892	0.473498	0.428963	Biso	1.000000	Si
Si457	1.0	0.606823	0.787583	0.910535	Biso	1.000000	Si
Si458	1.0	0.494477	0.596450	0.430146	Biso	1.000000	Si
Si459	1.0	0.387978	0.734741	0.167932	Biso	1.000000	Si
Al62	1.0	0.834493	0.277805	0.796492	Biso	1.000000	Al
Si460	1.0	0.730838	0.421134	0.316782	Biso	1.000000	Si
Si461	1.0	0.209844	0.347480	0.924976	Biso	1.000000	Si
Si462	1.0	0.129622	0.379784	0.918666	Biso	1.000000	Si
Si463	1.0	0.635849	0.446115	0.121629	Biso	1.000000	Si
Si464	1.0	0.461723	0.156637	0.713670	Biso	1.000000	Si
Si465	1.0	0.668765	0.511948	0.970209	Biso	1.000000	Si
Si466	1.0	0.760452	0.780782	0.287641	Biso	1.000000	Si
Si467	1.0	0.681541	0.021322	0.069475	Biso	1.000000	Si
Si468	1.0	0.009891	0.810542	0.463103	Biso	1.000000	Si
Si469	1.0	0.596884	0.566502	0.379463	Biso	1.000000	Si
Al63	1.0	0.076000	0.616728	0.189226	Biso	1.000000	Al
Si470	1.0	0.790210	0.918713	0.448122	Biso	1.000000	Si
Si471	1.0	0.818216	0.618139	0.140260	Biso	1.000000	Si
Si472	1.0	0.565119	0.934939	0.336137	Biso	1.000000	Si
Si473	1.0	0.207117	0.028377	0.919448	Biso	1.000000	Si
Si474	1.0	0.996246	0.671432	0.611585	Biso	1.000000	Si
Si475	1.0	0.590769	0.892482	0.991230	Biso	1.000000	Si
Si476	1.0	0.969605	0.824996	0.303431	Biso	1.000000	Si
Si477	1.0	0.667352	0.177937	0.396690	Biso	1.000000	Si
Si478	1.0	0.612484	0.861015	0.225257	Biso	1.000000	Si
Si479	1.0	0.068921	0.852928	0.415454	Biso	1.000000	Si
Si480	1.0	0.219107	0.466104	0.752659	Biso	1.000000	Si
Si481	1.0	0.360624	0.591190	0.890929	Biso	1.000000	Si
Si482	1.0	0.948348	0.165476	0.142005	Biso	1.000000	Si
Si483	1.0	0.684830	0.842702	0.180597	Biso	1.000000	Si
Si484	1.0	0.814402	0.747335	0.557509	Biso	1.000000	Si
Si485	1.0	0.921898	0.872743	0.420377	Biso	1.000000	Si
Al64	1.0	0.266752	0.070534	0.333281	Biso	1.000000	Al
Si486	1.0	0.592506	0.945889	0.574003	Biso	1.000000	Si
Si487	1.0	0.495584	0.639494	0.617214	Biso	1.000000	Si
Al65	1.0	0.688405	0.221866	0.204540	Biso	1.000000	Al
Si488	1.0	0.644216	0.238975	0.776703	Biso	1.000000	Si
Si489	1.0	0.409410	0.935464	0.457657	Biso	1.000000	Si
Si490	1.0	0.122332	0.520751	0.942056	Biso	1.000000	Si
Si491	1.0	0.485815	0.990193	0.614330	Biso	1.000000	Si
Si492	1.0	0.870913	0.440322	0.628886	Biso	1.000000	Si
Si493	1.0	0.979787	0.430076	0.423116	Biso	1.000000	Si
Si494	1.0	0.638190	0.237346	0.452911	Biso	1.000000	Si
Si495	1.0	0.212629	0.438482	0.262328	Biso	1.000000	Si

Si496	1.0	0.841173	0.784681	0.901229	Biso	1.000000	Si
Si497	1.0	0.762359	0.815370	0.884057	Biso	1.000000	Si
Si498	1.0	0.620542	0.777022	0.050570	Biso	1.000000	Si
Al66	1.0	0.908333	0.996765	0.432956	Biso	1.000000	Al
Si499	1.0	0.626851	0.243007	0.308324	Biso	1.000000	Si
Si500	1.0	0.180553	0.017570	0.998580	Biso	1.000000	Si
Si501	1.0	0.169453	0.968882	0.465390	Biso	1.000000	Si
Si502	1.0	0.768690	0.801710	0.439999	Biso	1.000000	Si
Si503	1.0	0.198296	0.769514	0.521919	Biso	1.000000	Si
Si504	1.0	0.864329	0.636765	0.956922	Biso	1.000000	Si
Si505	1.0	0.023071	0.453504	0.786173	Biso	1.000000	Si
Si506	1.0	0.086555	0.826163	0.897699	Biso	1.000000	Si
Si507	1.0	0.591113	0.616805	0.156458	Biso	1.000000	Si
Si508	1.0	0.624323	0.148016	0.116904	Biso	1.000000	Si
Si509	1.0	0.216587	0.527902	0.011438	Biso	1.000000	Si
Si510	1.0	0.684921	0.555947	0.102435	Biso	1.000000	Si
Si511	1.0	0.271251	0.754571	0.563847	Biso	1.000000	Si
Si512	1.0	0.253929	0.750329	0.127225	Biso	1.000000	Si
Si513	1.0	0.596985	0.516296	0.926383	Biso	1.000000	Si
Si514	1.0	0.457499	0.996080	0.417174	Biso	1.000000	Si
Si515	1.0	0.353732	0.557557	0.283462	Biso	1.000000	Si
Si516	1.0	0.782612	0.419844	0.148649	Biso	1.000000	Si
Si517	1.0	0.980054	0.972060	0.877117	Biso	1.000000	Si
Si518	1.0	0.956287	0.125355	0.587945	Biso	1.000000	Si
Si519	1.0	0.779999	0.961876	0.192880	Biso	1.000000	Si
Si520	1.0	0.725394	0.275852	0.153914	Biso	1.000000	Si
Si521	1.0	0.966664	0.361627	0.723581	Biso	1.000000	Si
Si522	1.0	0.317397	0.407296	0.394310	Biso	1.000000	Si
Al67	1.0	0.537753	0.869179	0.197147	Biso	1.000000	Al
Si523	1.0	0.197206	0.380273	0.578252	Biso	1.000000	Si
Si524	1.0	0.506184	0.522171	0.308239	Biso	1.000000	Si
Si525	1.0	0.960841	0.473484	0.021379	Biso	1.000000	Si
Si526	1.0	0.616362	0.972786	0.740317	Biso	1.000000	Si
Si527	1.0	0.259244	0.516758	0.350079	Biso	1.000000	Si
Si528	1.0	0.850385	0.696896	0.898275	Biso	1.000000	Si
Si529	1.0	0.441016	0.475412	0.343576	Biso	1.000000	Si
O1	1.0	0.473509	0.139088	0.972797	Biso	1.000000	O
O2	1.0	0.225618	0.397516	0.680092	Biso	1.000000	O
O3	1.0	0.174711	0.461827	0.868793	Biso	1.000000	O
O4	1.0	0.188501	0.666764	0.167185	Biso	1.000000	O
O5	1.0	0.914599	0.854933	0.752305	Biso	1.000000	O
O6	1.0	0.825386	0.787961	0.336372	Biso	1.000000	O
O7	1.0	0.127402	0.510116	0.983660	Biso	1.000000	O
O8	1.0	0.551183	0.569518	0.007113	Biso	1.000000	O
O9	1.0	0.672895	0.054576	0.698248	Biso	1.000000	O
O10	1.0	0.524043	0.563304	0.672547	Biso	1.000000	O
O11	1.0	0.050712	0.356999	0.790815	Biso	1.000000	O
O12	1.0	0.201614	0.122552	0.960278	Biso	1.000000	O
O13	1.0	0.201316	0.369391	0.373840	Biso	1.000000	O
O14	1.0	0.360736	0.338955	0.071008	Biso	1.000000	O
O15	1.0	0.054642	0.288246	0.922188	Biso	1.000000	O
O16	1.0	0.969323	0.527549	0.291536	Biso	1.000000	O
O17	1.0	0.548795	0.280298	0.279632	Biso	1.000000	O
O18	1.0	0.257212	0.836764	0.295303	Biso	1.000000	O
O19	1.0	0.035595	0.902146	0.927067	Biso	1.000000	O
O20	1.0	0.138752	0.471411	0.609994	Biso	1.000000	O
O21	1.0	0.295211	0.458726	0.195459	Biso	1.000000	O
O22	1.0	0.737599	0.785623	0.863772	Biso	1.000000	O
O23	1.0	0.004942	0.346923	0.592393	Biso	1.000000	O
O24	1.0	0.612171	0.000506	0.774613	Biso	1.000000	O
O25	1.0	0.056528	0.076168	0.727503	Biso	1.000000	O
O26	1.0	0.389874	0.490693	0.629057	Biso	1.000000	O
O27	1.0	0.001676	0.310072	0.515753	Biso	1.000000	O
O28	1.0	0.146441	0.722337	0.333212	Biso	1.000000	O
O29	1.0	0.696031	0.877088	0.400195	Biso	1.000000	O
O30	1.0	0.133625	0.403451	0.882067	Biso	1.000000	O
O31	1.0	0.620863	0.176704	0.954228	Biso	1.000000	O
O32	1.0	0.972831	0.385345	0.417305	Biso	1.000000	O
O33	1.0	0.523354	0.616890	0.404774	Biso	1.000000	O
O34	1.0	0.398011	0.990730	0.863737	Biso	1.000000	O
O35	1.0	0.272982	0.709502	0.565531	Biso	1.000000	O
O36	1.0	0.002686	0.648823	0.576573	Biso	1.000000	O
O37	1.0	0.048303	0.955249	0.799134	Biso	1.000000	O
O38	1.0	0.471701	0.778967	0.953732	Biso	1.000000	O
O39	1.0	0.180441	0.098454	0.660030	Biso	1.000000	O
O40	1.0	0.039179	0.185951	0.278460	Biso	1.000000	O
O41	1.0	0.434660	0.430536	0.449703	Biso	1.000000	O
O42	1.0	0.795778	0.707179	0.558488	Biso	1.000000	O
O43	1.0	0.944517	0.243010	0.681051	Biso	1.000000	O
O44	1.0	0.584806	0.967019	0.232414	Biso	1.000000	O
O45	1.0	0.871086	0.342312	0.550087	Biso	1.000000	O
O46	1.0	0.592264	0.149097	0.014540	Biso	1.000000	O
O47	1.0	0.738593	0.261595	0.828574	Biso	1.000000	O
O48	1.0	0.101868	0.079183	0.547800	Biso	1.000000	O
O49	1.0	0.217473	0.670635	0.543463	Biso	1.000000	O

O50	1.0	0.462739	0.817701	0.157331	Biso	1.000000	O
O51	1.0	0.164735	0.581064	0.445996	Biso	1.000000	O
O52	1.0	0.938461	0.597574	0.670323	Biso	1.000000	O
O53	1.0	0.149907	0.454637	0.071208	Biso	1.000000	O
O54	1.0	0.964254	0.783008	0.289491	Biso	1.000000	O
O55	1.0	0.378299	0.716996	0.279395	Biso	1.000000	O
O56	1.0	0.577543	0.362556	0.094712	Biso	1.000000	O
O57	1.0	0.401974	0.171490	0.861144	Biso	1.000000	O
O58	1.0	0.336540	0.269179	0.065083	Biso	1.000000	O
O59	1.0	0.480833	0.097247	0.880890	Biso	1.000000	O
O60	1.0	0.037068	0.774702	0.146293	Biso	1.000000	O
O61	1.0	0.091645	0.244575	0.387608	Biso	1.000000	O
O62	1.0	0.822396	0.512473	0.034530	Biso	1.000000	O
O63	1.0	0.966405	0.777826	0.370476	Biso	1.000000	O
O64	1.0	0.633785	0.884687	0.991596	Biso	1.000000	O
O65	1.0	0.707079	0.515140	0.096411	Biso	1.000000	O
O66	1.0	0.481151	0.702547	0.336486	Biso	1.000000	O
O67	1.0	0.285865	0.501009	0.318564	Biso	1.000000	O
O68	1.0	0.925990	0.559300	0.056565	Biso	1.000000	O
O69	1.0	0.965570	0.211142	0.988256	Biso	1.000000	O
O70	1.0	0.711118	0.698802	0.754837	Biso	1.000000	O
O71	1.0	0.085630	0.815778	0.941677	Biso	1.000000	O
O72	1.0	0.562942	0.842159	0.390947	Biso	1.000000	O
O73	1.0	0.344759	0.540652	0.983207	Biso	1.000000	O
O74	1.0	0.938079	0.881640	0.833791	Biso	1.000000	O
O75	1.0	0.918881	0.165331	0.910505	Biso	1.000000	O
O76	1.0	0.811129	0.368108	0.507004	Biso	1.000000	O
O77	1.0	0.250833	0.178936	0.081441	Biso	1.000000	O
O78	1.0	0.826576	0.416968	0.456994	Biso	1.000000	O
O79	1.0	0.892070	0.960590	0.409460	Biso	1.000000	O
O80	1.0	0.269378	0.808040	0.664397	Biso	1.000000	O
O81	1.0	0.437834	0.788903	0.544633	Biso	1.000000	O
O82	1.0	0.615535	0.591969	0.025606	Biso	1.000000	O
O83	1.0	0.173234	0.780858	0.773757	Biso	1.000000	O
O84	1.0	0.513573	0.577600	0.463814	Biso	1.000000	O
O85	1.0	0.105623	0.116092	0.760373	Biso	1.000000	O
O86	1.0	0.810793	0.277638	0.833563	Biso	1.000000	O
O87	1.0	0.764552	0.888657	0.043943	Biso	1.000000	O
O88	1.0	0.961913	0.510315	0.221539	Biso	1.000000	O
O89	1.0	0.229020	0.381483	0.944593	Biso	1.000000	O
O90	1.0	0.362924	0.740142	0.202627	Biso	1.000000	O
O91	1.0	0.999634	0.313298	0.023030	Biso	1.000000	O
O92	1.0	0.642447	0.738941	0.060009	Biso	1.000000	O
O93	1.0	0.351060	0.316033	0.175425	Biso	1.000000	O
O94	1.0	0.137586	0.298739	0.643550	Biso	1.000000	O
O95	1.0	0.416008	0.958988	0.262720	Biso	1.000000	O
O96	1.0	0.347815	0.257582	0.673220	Biso	1.000000	O
O97	1.0	0.206627	0.920652	0.964521	Biso	1.000000	O
O98	1.0	0.446034	0.641780	0.509023	Biso	1.000000	O
O99	1.0	0.742674	0.229892	0.013060	Biso	1.000000	O
O100	1.0	0.812082	0.982980	0.989801	Biso	1.000000	O
O101	1.0	0.099377	0.462897	0.328731	Biso	1.000000	O
O102	1.0	0.769333	0.639383	0.916717	Biso	1.000000	O
O103	1.0	0.655192	0.459447	0.158067	Biso	1.000000	O
O104	1.0	0.781128	0.497954	0.209704	Biso	1.000000	O
O105	1.0	0.665242	0.931068	0.366856	Biso	1.000000	O
O106	1.0	0.213660	0.417661	0.301179	Biso	1.000000	O
O107	1.0	0.464270	0.475861	0.143461	Biso	1.000000	O
O108	1.0	0.611382	0.624407	0.903257	Biso	1.000000	O
O109	1.0	0.430274	0.754943	0.659373	Biso	1.000000	O
O110	1.0	0.476378	0.980212	0.381502	Biso	1.000000	O
O111	1.0	0.957578	0.389229	0.624233	Biso	1.000000	O
O112	1.0	0.432175	0.913536	0.487395	Biso	1.000000	O
O113	1.0	0.796509	0.710839	0.078294	Biso	1.000000	O
O114	1.0	0.365260	0.749377	0.133798	Biso	1.000000	O
O115	1.0	0.432912	0.383405	0.269414	Biso	1.000000	O
O116	1.0	0.183208	0.978818	0.740061	Biso	1.000000	O
O117	1.0	0.927713	0.182700	0.108463	Biso	1.000000	O
O118	1.0	0.675066	0.094777	0.558755	Biso	1.000000	O
O119	1.0	0.148128	0.504596	0.361626	Biso	1.000000	O
O120	1.0	0.564759	0.353443	0.682109	Biso	1.000000	O
O121	1.0	0.630278	0.559167	0.541027	Biso	1.000000	O
O122	1.0	0.582646	0.937267	0.296562	Biso	1.000000	O
O123	1.0	0.506417	0.010309	0.948603	Biso	1.000000	O
O124	1.0	0.743853	0.828994	0.920353	Biso	1.000000	O
O125	1.0	0.415536	0.854135	0.114121	Biso	1.000000	O

O126	1.0	0.119752	0.136330	0.654396	Biso	1.000000	O
O127	1.0	0.749053	0.086500	0.228397	Biso	1.000000	O
O128	1.0	0.004881	0.911233	0.679590	Biso	1.000000	O
O129	1.0	0.216674	0.573607	0.681539	Biso	1.000000	O
O130	1.0	0.427058	0.209073	0.576947	Biso	1.000000	O
O131	1.0	0.050096	0.812623	0.881814	Biso	1.000000	O
O132	1.0	0.640339	0.991837	0.710582	Biso	1.000000	O
O133	1.0	0.766528	0.740727	0.305663	Biso	1.000000	O
O134	1.0	0.695135	0.225205	0.542103	Biso	1.000000	O
O135	1.0	0.350066	0.917713	0.571405	Biso	1.000000	O
O136	1.0	0.108312	0.035025	0.602841	Biso	1.000000	O
O137	1.0	0.295003	0.757950	0.378046	Biso	1.000000	O
O138	1.0	0.260942	0.558034	0.463623	Biso	1.000000	O
O139	1.0	0.453129	0.163904	0.402793	Biso	1.000000	O
O140	1.0	0.531021	0.961942	0.339732	Biso	1.000000	O
O141	1.0	0.454649	0.324506	0.891496	Biso	1.000000	O
O142	1.0	0.788509	0.592196	0.599366	Biso	1.000000	O
O143	1.0	0.350981	0.782787	0.837509	Biso	1.000000	O
O144	1.0	0.988232	0.072196	0.824208	Biso	1.000000	O
O145	1.0	0.718510	0.588927	0.907060	Biso	1.000000	O
O146	1.0	0.482693	0.951898	0.636232	Biso	1.000000	O
O147	1.0	0.003566	0.983710	0.675853	Biso	1.000000	O
O148	1.0	0.590602	0.588873	0.845455	Biso	1.000000	O
O149	1.0	0.319427	0.182865	0.445838	Biso	1.000000	O
O150	1.0	0.784929	0.339446	0.216073	Biso	1.000000	O
O151	1.0	0.772562	0.002399	0.176530	Biso	1.000000	O
O152	1.0	0.452121	0.518687	0.042964	Biso	1.000000	O
O153	1.0	0.764139	0.449292	0.322547	Biso	1.000000	O
O154	1.0	0.582951	0.926601	0.963830	Biso	1.000000	O
O155	1.0	0.256022	0.335099	0.659409	Biso	1.000000	O
O156	1.0	0.487865	0.012848	0.443922	Biso	1.000000	O
O157	1.0	0.874953	0.622828	0.651152	Biso	1.000000	O
O158	1.0	0.309064	0.709905	0.715123	Biso	1.000000	O
O159	1.0	0.494573	0.752333	0.166635	Biso	1.000000	O
O160	1.0	0.141981	0.680023	0.494389	Biso	1.000000	O
O161	1.0	0.149334	0.580882	0.817598	Biso	1.000000	O
O162	1.0	0.360690	0.445070	0.676033	Biso	1.000000	O
O163	1.0	0.460975	0.423491	0.870006	Biso	1.000000	O
O164	1.0	0.518077	0.357322	0.134309	Biso	1.000000	O
O165	1.0	0.900122	0.639895	0.979933	Biso	1.000000	O
O166	1.0	0.593798	0.666747	0.695130	Biso	1.000000	O
O167	1.0	0.565168	0.544133	0.401206	Biso	1.000000	O
O168	1.0	0.759388	0.083131	0.876624	Biso	1.000000	O
O169	1.0	0.228930	0.292068	0.241927	Biso	1.000000	O
O170	1.0	0.930788	0.119772	0.784295	Biso	1.000000	O
O171	1.0	0.898938	0.235293	0.982946	Biso	1.000000	O
O172	1.0	0.731575	0.115035	0.116095	Biso	1.000000	O
O173	1.0	0.066603	0.333571	0.494708	Biso	1.000000	O
O174	1.0	0.438227	0.839325	0.049071	Biso	1.000000	O
O175	1.0	0.605243	0.508856	0.282734	Biso	1.000000	O
O176	1.0	0.681052	0.333006	0.352710	Biso	1.000000	O
O177	1.0	0.352443	0.146978	0.691990	Biso	1.000000	O
O178	1.0	0.531273	0.697547	0.040037	Biso	1.000000	O
O179	1.0	0.849879	0.967789	0.123883	Biso	1.000000	O
O180	1.0	0.368288	0.088559	0.959384	Biso	1.000000	O
O181	1.0	0.091011	0.313238	0.831257	Biso	1.000000	O
O182	1.0	0.761735	0.666983	0.796391	Biso	1.000000	O
O183	1.0	0.090274	0.920803	0.118711	Biso	1.000000	O
O184	1.0	0.712305	0.894757	0.539625	Biso	1.000000	O
O185	1.0	0.050930	0.007813	0.434818	Biso	1.000000	O
O186	1.0	0.429405	0.592028	0.148856	Biso	1.000000	O
O187	1.0	0.090936	0.897176	0.051509	Biso	1.000000	O
O188	1.0	0.084356	0.048247	0.669674	Biso	1.000000	O
O189	1.0	0.644788	0.701008	0.511752	Biso	1.000000	O
O190	1.0	0.091723	0.188777	0.563566	Biso	1.000000	O
O191	1.0	0.723366	0.438618	0.718339	Biso	1.000000	O
O192	1.0	0.879852	0.079556	0.018152	Biso	1.000000	O
O193	1.0	0.184237	0.358376	0.786898	Biso	1.000000	O
O194	1.0	0.654186	0.356087	0.565714	Biso	1.000000	O
O195	1.0	0.292582	0.326298	0.069930	Biso	1.000000	O
O196	1.0	0.557586	0.211862	0.525757	Biso	1.000000	O
O197	1.0	0.658869	0.690041	0.381210	Biso	1.000000	O
O198	1.0	0.807960	0.270338	0.761360	Biso	1.000000	O
O199	1.0	0.350140	0.750062	0.335660	Biso	1.000000	O
O200	1.0	0.394389	0.963617	0.102452	Biso	1.000000	O
O201	1.0	0.415294	0.179594	0.997599	Biso	1.000000	O
O202	1.0	0.868981	0.674056	0.262019	Biso	1.000000	O
O203	1.0	0.658778	0.584386	0.865375	Biso	1.000000	O
O204	1.0	0.691250	0.629257	0.746221	Biso	1.000000	O

O205	1.0	0.753840	0.157766	0.241320	Biso	1.000000	O
O206	1.0	0.225574	0.030630	0.529805	Biso	1.000000	O
O207	1.0	0.363160	0.319315	0.425386	Biso	1.000000	O
O208	1.0	0.921491	0.188881	0.721635	Biso	1.000000	O
O209	1.0	0.283639	0.102319	0.214080	Biso	1.000000	O
O210	1.0	0.047158	0.255898	0.457291	Biso	1.000000	O
O211	1.0	0.396376	0.450329	0.887246	Biso	1.000000	O
O212	1.0	0.699417	0.993682	0.958106	Biso	1.000000	O
O213	1.0	0.970305	0.024366	0.625797	Biso	1.000000	O
O214	1.0	0.331464	0.166376	0.514380	Biso	1.000000	O
O215	1.0	0.982681	0.774176	0.595146	Biso	1.000000	O
O216	1.0	0.687457	0.024246	0.026129	Biso	1.000000	O
O217	1.0	0.320826	0.450809	0.403038	Biso	1.000000	O
O218	1.0	0.290908	0.326900	0.139283	Biso	1.000000	O
O219	1.0	0.868135	0.794603	0.641877	Biso	1.000000	O
O220	1.0	0.831849	0.575103	0.522614	Biso	1.000000	O
O221	1.0	0.046400	0.582788	0.177156	Biso	1.000000	O
O222	1.0	0.226523	0.868947	0.518471	Biso	1.000000	O
O223	1.0	0.272767	0.636160	0.569622	Biso	1.000000	O
O224	1.0	0.866794	0.601996	0.929637	Biso	1.000000	O
O225	1.0	0.066770	0.114801	0.991460	Biso	1.000000	O
O226	1.0	0.278803	0.505956	0.087835	Biso	1.000000	O
O227	1.0	0.513881	0.383314	0.069917	Biso	1.000000	O
O228	1.0	0.024969	0.186405	0.467107	Biso	1.000000	O
O229	1.0	0.933454	0.339162	0.026478	Biso	1.000000	O
O230	1.0	0.826914	0.169007	0.581479	Biso	1.000000	O
O231	1.0	0.168856	0.414216	0.578064	Biso	1.000000	O
O232	1.0	0.243644	0.548211	0.038825	Biso	1.000000	O
O233	1.0	0.905910	0.544557	0.254546	Biso	1.000000	O
O234	1.0	0.278588	0.260159	0.863925	Biso	1.000000	O
O235	1.0	0.494922	0.045478	0.535084	Biso	1.000000	O
O236	1.0	0.172693	0.234859	0.631511	Biso	1.000000	O
O237	1.0	0.665802	0.114182	0.626454	Biso	1.000000	O
O238	1.0	0.955501	0.250344	0.216415	Biso	1.000000	O
O239	1.0	0.641369	0.007357	0.077389	Biso	1.000000	O
O240	1.0	0.695177	0.111702	0.878925	Biso	1.000000	O
O241	1.0	0.386203	0.343044	0.886813	Biso	1.000000	O
O242	1.0	0.022872	0.483332	0.818467	Biso	1.000000	O
O243	1.0	0.037171	0.065199	0.522410	Biso	1.000000	O
O244	1.0	0.513653	0.465777	0.586405	Biso	1.000000	O
O245	1.0	0.427930	0.310930	0.277384	Biso	1.000000	O
O246	1.0	0.882065	0.929640	0.073219	Biso	1.000000	O
O247	1.0	0.822217	0.423340	0.165246	Biso	1.000000	O
O248	1.0	0.112829	0.680369	0.253592	Biso	1.000000	O
O249	1.0	0.701739	0.322683	0.816867	Biso	1.000000	O
O250	1.0	0.292299	0.628138	0.333815	Biso	1.000000	O
O251	1.0	0.940805	0.125715	0.229778	Biso	1.000000	O
O252	1.0	0.748640	0.492769	0.451758	Biso	1.000000	O
O253	1.0	0.916231	0.063107	0.350649	Biso	1.000000	O
O254	1.0	0.230779	0.318935	0.103739	Biso	1.000000	O
O255	1.0	0.936683	0.652693	0.039969	Biso	1.000000	O
O256	1.0	0.633219	0.933539	0.752401	Biso	1.000000	O
O257	1.0	0.973900	0.045263	0.494364	Biso	1.000000	O
O258	1.0	0.857618	0.768540	0.456577	Biso	1.000000	O
O259	1.0	0.917284	0.058346	0.243230	Biso	1.000000	O
O260	1.0	0.468282	0.948936	0.936634	Biso	1.000000	O
O261	1.0	0.790111	0.902850	0.626929	Biso	1.000000	O
O262	1.0	0.180616	0.554692	0.006955	Biso	1.000000	O
O263	1.0	0.479066	0.259032	0.283563	Biso	1.000000	O
O264	1.0	0.067695	0.314488	0.733833	Biso	1.000000	O
O265	1.0	0.680823	0.613511	0.008351	Biso	1.000000	O
O266	1.0	0.007368	0.360991	0.732230	Biso	1.000000	O
O267	1.0	0.177501	0.161502	0.623573	Biso	1.000000	O
O268	1.0	0.332896	0.187567	0.875482	Biso	1.000000	O
O269	1.0	0.233332	0.213756	0.733587	Biso	1.000000	O
O270	1.0	0.636057	0.278327	0.332119	Biso	1.000000	O
O271	1.0	0.765585	0.600309	0.528924	Biso	1.000000	O
O272	1.0	0.689103	0.062085	0.085628	Biso	1.000000	O
O273	1.0	0.870056	0.023114	0.518694	Biso	1.000000	O
O274	1.0	0.890158	0.445050	0.171669	Biso	1.000000	O
O275	1.0	0.875697	0.662056	0.593530	Biso	1.000000	O
O276	1.0	0.512031	0.046276	0.070643	Biso	1.000000	O
O277	1.0	0.328414	0.816145	0.624650	Biso	1.000000	O
O278	1.0	0.708293	0.721752	0.485274	Biso	1.000000	O
O279	1.0	0.434802	0.051576	0.853155	Biso	1.000000	O
O280	1.0	0.365892	0.958432	0.165605	Biso	1.000000	O
O281	1.0	0.639726	0.689240	0.219797	Biso	1.000000	O
O282	1.0	0.367645	0.057036	0.387403	Biso	1.000000	O
O283	1.0	0.417088	0.657240	0.797285	Biso	1.000000	O
O284	1.0	0.775014	0.149387	0.431010	Biso	1.000000	O
O285	1.0	0.650971	0.853768	0.204929	Biso	1.000000	O
O286	1.0	0.504298	0.284444	0.002523	Biso	1.000000	O
O287	1.0	0.198689	0.725964	0.033406	Biso	1.000000	O
O288	1.0	0.254307	0.537233	0.234136	Biso	1.000000	O
O289	1.0	0.417281	0.478453	0.234396	Biso	1.000000	O

O290	1.0	0.661463	0.999176	0.826514	Biso	1.000000	O
O291	1.0	0.930777	0.913647	0.267037	Biso	1.000000	O
O292	1.0	0.158269	0.001743	0.439089	Biso	1.000000	O
O293	1.0	0.485139	0.196270	0.096437	Biso	1.000000	O
O294	1.0	0.598049	0.144461	0.083437	Biso	1.000000	O
O295	1.0	0.573338	0.572707	0.721613	Biso	1.000000	O
O296	1.0	0.964111	0.307712	0.083123	Biso	1.000000	O
O297	1.0	0.573500	0.046968	0.429527	Biso	1.000000	O
O298	1.0	0.470909	0.568183	0.406560	Biso	1.000000	O
O299	1.0	0.793115	0.408943	0.945692	Biso	1.000000	O
O300	1.0	0.372111	0.738714	0.698095	Biso	1.000000	O
O301	1.0	0.077143	0.224118	0.896083	Biso	1.000000	O
O302	1.0	0.614427	0.257986	0.268640	Biso	1.000000	O
O303	1.0	0.228764	0.726486	0.153670	Biso	1.000000	O
O304	1.0	0.783412	0.595160	0.134057	Biso	1.000000	O
O305	1.0	0.567092	0.768088	0.807736	Biso	1.000000	O
O306	1.0	0.737821	0.669277	0.194034	Biso	1.000000	O
O307	1.0	0.027007	0.454375	0.333256	Biso	1.000000	O
O308	1.0	0.932890	0.022030	0.405704	Biso	1.000000	O
O309	1.0	0.627228	0.939480	0.426392	Biso	1.000000	O
O310	1.0	0.851542	0.169472	0.718988	Biso	1.000000	O
O311	1.0	0.174723	0.253602	0.959599	Biso	1.000000	O
O312	1.0	0.464941	0.196096	0.691608	Biso	1.000000	O
O313	1.0	0.874400	0.642981	0.815382	Biso	1.000000	O
O314	1.0	0.992824	0.023718	0.558518	Biso	1.000000	O
O315	1.0	0.134064	0.058059	0.139834	Biso	1.000000	O
O316	1.0	0.316759	0.814515	0.337674	Biso	1.000000	O
O317	1.0	0.208432	0.024426	0.028844	Biso	1.000000	O
O318	1.0	0.280155	0.954882	0.350406	Biso	1.000000	O
O319	1.0	0.964165	0.694312	0.488256	Biso	1.000000	O
O320	1.0	0.978083	0.082000	0.271963	Biso	1.000000	O
O321	1.0	0.461352	0.596347	0.797746	Biso	1.000000	O
O322	1.0	0.144232	0.378578	0.333049	Biso	1.000000	O
O323	1.0	0.590689	0.346469	0.595013	Biso	1.000000	O
O324	1.0	0.156165	0.507944	0.919146	Biso	1.000000	O
O325	1.0	0.275483	0.258628	0.573155	Biso	1.000000	O
O326	1.0	0.376920	0.850429	0.303436	Biso	1.000000	O
O327	1.0	0.020503	0.228301	0.941944	Biso	1.000000	O
O328	1.0	0.417935	0.106206	0.006391	Biso	1.000000	O
O329	1.0	0.785693	0.451459	0.047337	Biso	1.000000	O
O330	1.0	0.059617	0.833041	0.038421	Biso	1.000000	O
O331	1.0	0.774277	0.177116	0.822373	Biso	1.000000	O
O332	1.0	0.141154	0.045236	0.836779	Biso	1.000000	O
O333	1.0	0.200108	0.976379	0.568202	Biso	1.000000	O
O334	1.0	0.527797	0.739804	0.859550	Biso	1.000000	O
O335	1.0	0.764365	0.666528	0.306503	Biso	1.000000	O
O336	1.0	0.182124	0.809366	0.524468	Biso	1.000000	O
O337	1.0	0.916297	0.731169	0.262861	Biso	1.000000	O
O338	1.0	0.190576	0.716152	0.676039	Biso	1.000000	O
O339	1.0	0.638951	0.630228	0.528573	Biso	1.000000	O
O340	1.0	0.547976	0.895492	0.094678	Biso	1.000000	O
O341	1.0	0.534483	0.824002	0.102528	Biso	1.000000	O
O342	1.0	0.340711	0.207802	0.728752	Biso	1.000000	O
O343	1.0	0.638283	0.190898	0.120797	Biso	1.000000	O
O344	1.0	0.551829	0.026733	0.811255	Biso	1.000000	O
O345	1.0	0.905253	0.551236	0.897427	Biso	1.000000	O
O346	1.0	0.539100	0.054911	0.874866	Biso	1.000000	O
O347	1.0	0.793874	0.820223	0.471490	Biso	1.000000	O
O348	1.0	0.678966	0.473674	0.991704	Biso	1.000000	O
O349	1.0	0.438328	0.439765	0.315490	Biso	1.000000	O
O350	1.0	0.979516	0.136608	0.129257	Biso	1.000000	O
O351	1.0	0.391633	0.039353	0.006315	Biso	1.000000	O
O352	1.0	0.224443	0.220778	0.264525	Biso	1.000000	O
O353	1.0	0.679906	0.259807	0.789037	Biso	1.000000	O
O354	1.0	0.136504	0.703729	0.631969	Biso	1.000000	O
O355	1.0	0.284670	0.067103	0.553649	Biso	1.000000	O
O356	1.0	0.312887	0.872039	0.235476	Biso	1.000000	O
O357	1.0	0.040743	0.113048	0.284958	Biso	1.000000	O
O358	1.0	0.856708	0.673074	0.933461	Biso	1.000000	O
O359	1.0	0.230515	0.116122	0.118860	Biso	1.000000	O
O360	1.0	0.744694	0.576197	0.374636	Biso	1.000000	O
O361	1.0	0.002056	0.956742	0.447007	Biso	1.000000	O
O362	1.0	0.389858	0.908600	0.431506	Biso	1.000000	O
O363	1.0	0.474625	0.345932	0.358349	Biso	1.000000	O
O364	1.0	0.782565	0.266278	0.921574	Biso	1.000000	O
O365	1.0	0.267728	0.382922	0.490606	Biso	1.000000	O
O366	1.0	0.496736	0.798039	0.506112	Biso	1.000000	O
O367	1.0	0.168054	0.737913	0.530077	Biso	1.000000	O
O368	1.0	0.587686	0.706750	0.840006	Biso	1.000000	O
O369	1.0	0.887239	0.187047	0.481532	Biso	1.000000	O
O370	1.0	0.121071	0.961462	0.069385	Biso	1.000000	O
O371	1.0	0.096151	0.177961	0.017989	Biso	1.000000	O
O372	1.0	0.267267	0.741315	0.025156	Biso	1.000000	O
O373	1.0	0.346749	0.331461	0.943091	Biso	1.000000	O
O374	1.0	0.145954	0.514771	0.224763	Biso	1.000000	O

O375	1.0	0.916883	0.109424	0.294588	Biso	1.000000	O
O376	1.0	0.371671	0.512837	0.193237	Biso	1.000000	O
O377	1.0	0.989842	0.682576	0.911612	Biso	1.000000	O
O378	1.0	0.324492	0.312736	0.878870	Biso	1.000000	O
O379	1.0	0.399490	0.499899	0.128547	Biso	1.000000	O
O380	1.0	0.683404	0.779640	0.819631	Biso	1.000000	O
O381	1.0	0.580851	0.040487	0.629221	Biso	1.000000	O
O382	1.0	0.558031	0.335172	0.751553	Biso	1.000000	O
O383	1.0	0.064757	0.680037	0.097101	Biso	1.000000	O
O384	1.0	0.858745	0.805976	0.394171	Biso	1.000000	O
O385	1.0	0.210643	0.827117	0.240867	Biso	1.000000	O
O386	1.0	0.240900	0.690456	0.720010	Biso	1.000000	O
O387	1.0	0.231331	0.351935	0.438031	Biso	1.000000	O
O388	1.0	0.230503	0.102705	0.526376	Biso	1.000000	O
O389	1.0	0.417022	0.290293	0.169476	Biso	1.000000	O
O390	1.0	0.437394	0.727074	0.782188	Biso	1.000000	O
O391	1.0	0.641174	0.360284	0.822469	Biso	1.000000	O
O392	1.0	0.605746	0.107485	0.449771	Biso	1.000000	O
O393	1.0	0.544459	0.126502	0.889729	Biso	1.000000	O
O394	1.0	0.598541	0.467251	0.340948	Biso	1.000000	O
O395	1.0	0.642133	0.640431	0.433019	Biso	1.000000	O
O396	1.0	0.533084	0.048290	0.002801	Biso	1.000000	O
O397	1.0	0.949148	0.422934	0.231218	Biso	1.000000	O
O398	1.0	0.015376	0.983644	0.854480	Biso	1.000000	O
O399	1.0	0.319848	0.113848	0.468317	Biso	1.000000	O
O400	1.0	0.183197	0.840413	0.621196	Biso	1.000000	O
O401	1.0	0.101324	0.032628	0.311845	Biso	1.000000	O
O402	1.0	0.103424	0.111744	0.177516	Biso	1.000000	O
O403	1.0	0.625312	0.628223	0.258218	Biso	1.000000	O
O404	1.0	0.231605	0.745745	0.343003	Biso	1.000000	O
O405	1.0	0.915635	0.205973	0.542681	Biso	1.000000	O
O406	1.0	0.495367	0.215953	0.028956	Biso	1.000000	O
O407	1.0	0.980749	0.209355	0.629968	Biso	1.000000	O
O408	1.0	0.972942	0.761293	0.111622	Biso	1.000000	O
O409	1.0	0.408072	0.888515	0.932499	Biso	1.000000	O
O410	1.0	0.705691	0.046993	0.909409	Biso	1.000000	O
O411	1.0	0.797015	0.846516	0.091371	Biso	1.000000	O
O412	1.0	0.262462	0.685608	0.904451	Biso	1.000000	O
O413	1.0	0.344616	0.625900	0.379561	Biso	1.000000	O
O414	1.0	0.763749	0.918900	0.833876	Biso	1.000000	O
O415	1.0	0.699870	0.043430	0.390344	Biso	1.000000	O
O416	1.0	0.750264	0.627312	0.249598	Biso	1.000000	O
O417	1.0	0.477898	0.217091	0.625091	Biso	1.000000	O
O418	1.0	0.103172	0.435542	0.560085	Biso	1.000000	O
O419	1.0	0.520713	0.034592	0.207414	Biso	1.000000	O
O420	1.0	0.833245	0.979221	0.056656	Biso	1.000000	O
O421	1.0	0.868868	0.538746	0.572310	Biso	1.000000	O
O422	1.0	0.098818	0.733097	0.516511	Biso	1.000000	O
O423	1.0	0.201016	0.291174	0.761363	Biso	1.000000	O
O424	1.0	0.626890	0.158968	0.671375	Biso	1.000000	O
O425	1.0	0.797108	0.891639	0.482333	Biso	1.000000	O
O426	1.0	0.915783	0.764433	0.415816	Biso	1.000000	O
O427	1.0	0.251904	0.401869	0.007365	Biso	1.000000	O
O428	1.0	0.203739	0.179870	0.136215	Biso	1.000000	O
O429	1.0	0.314294	0.071957	0.916968	Biso	1.000000	O
O430	1.0	0.947331	0.870361	0.691480	Biso	1.000000	O
O431	1.0	0.105527	0.792203	0.152989	Biso	1.000000	O
O432	1.0	0.019481	0.436420	0.438481	Biso	1.000000	O
O433	1.0	0.521020	0.070530	0.474927	Biso	1.000000	O
O434	1.0	0.138560	0.936029	0.465169	Biso	1.000000	O
O435	1.0	0.822789	0.018791	0.650746	Biso	1.000000	O
O436	1.0	0.581712	0.858178	0.196216	Biso	1.000000	O
O437	1.0	0.770960	0.299244	0.640868	Biso	1.000000	O
O438	1.0	0.301400	0.304194	0.813128	Biso	1.000000	O
O439	1.0	0.837417	0.471735	0.410725	Biso	1.000000	O
O440	1.0	0.086003	0.721761	0.149657	Biso	1.000000	O
O441	1.0	0.934641	0.924932	0.025594	Biso	1.000000	O
O442	1.0	0.880877	0.844502	0.690946	Biso	1.000000	O
O443	1.0	0.627244	0.917176	0.571070	Biso	1.000000	O
O444	1.0	0.354358	0.631713	0.909614	Biso	1.000000	O
O445	1.0	0.519993	0.315377	0.486877	Biso	1.000000	O
O446	1.0	0.710118	0.893536	0.470364	Biso	1.000000	O
O447	1.0	0.297523	0.034912	0.193478	Biso	1.000000	O

O448	1.0	0.325830	0.142373	0.756206	Biso	1.000000	O
O449	1.0	0.120646	0.004051	0.935124	Biso	1.000000	O
O450	1.0	0.933728	0.980875	0.467101	Biso	1.000000	O
O451	1.0	0.748041	0.811252	0.316640	Biso	1.000000	O
O452	1.0	0.598346	0.014971	0.862011	Biso	1.000000	O
O453	1.0	0.117677	0.434390	0.691739	Biso	1.000000	O
O454	1.0	0.932527	0.704464	0.092285	Biso	1.000000	O
O455	1.0	0.731687	0.183092	0.304480	Biso	1.000000	O
O456	1.0	0.128678	0.601547	0.504237	Biso	1.000000	O
O457	1.0	0.332761	0.533814	0.051832	Biso	1.000000	O
O458	1.0	0.980433	0.145046	0.557019	Biso	1.000000	O
O459	1.0	0.948527	0.149239	0.846548	Biso	1.000000	O
O460	1.0	0.207945	0.952023	0.453124	Biso	1.000000	O
O461	1.0	0.906374	0.867129	0.535771	Biso	1.000000	O
O462	1.0	0.904442	0.185130	0.805580	Biso	1.000000	O
O463	1.0	0.893090	0.361403	0.149515	Biso	1.000000	O
O464	1.0	0.762252	0.427759	0.661150	Biso	1.000000	O
O465	1.0	0.351451	0.678160	0.332686	Biso	1.000000	O
O466	1.0	0.375760	0.814654	0.015329	Biso	1.000000	O
O467	1.0	0.192184	0.430726	0.980765	Biso	1.000000	O
O468	1.0	0.236133	0.073770	0.732115	Biso	1.000000	O
O469	1.0	0.650284	0.463360	0.565427	Biso	1.000000	O
O470	1.0	0.610487	0.214890	0.478190	Biso	1.000000	O
O471	1.0	0.299457	0.403388	0.353225	Biso	1.000000	O
O472	1.0	0.390487	0.571537	0.369994	Biso	1.000000	O
O473	1.0	0.915511	0.901663	0.961703	Biso	1.000000	O
O474	1.0	0.171339	0.767213	0.628248	Biso	1.000000	O
O475	1.0	0.794060	0.367452	0.637096	Biso	1.000000	O
O476	1.0	0.462715	0.506022	0.617538	Biso	1.000000	O
O477	1.0	0.173330	0.034619	0.379019	Biso	1.000000	O
O478	1.0	0.412485	0.100468	0.578214	Biso	1.000000	O
O479	1.0	0.419650	0.908075	0.555078	Biso	1.000000	O
O480	1.0	0.094346	0.968289	0.990928	Biso	1.000000	O
O481	1.0	0.426300	0.779964	0.728092	Biso	1.000000	O
O482	1.0	0.111319	0.856560	0.996845	Biso	1.000000	O
O483	1.0	0.301395	0.981190	0.054662	Biso	1.000000	O
O484	1.0	0.212764	0.868248	0.914166	Biso	1.000000	O
O485	1.0	0.152816	0.938139	0.830188	Biso	1.000000	O
O486	1.0	0.052779	0.925863	0.253144	Biso	1.000000	O
O487	1.0	0.829571	0.054505	0.759616	Biso	1.000000	O
O488	1.0	0.659765	0.122896	0.111372	Biso	1.000000	O
O489	1.0	0.108216	0.039893	0.407814	Biso	1.000000	O
O490	1.0	0.515233	0.261783	0.440239	Biso	1.000000	O
O491	1.0	0.432145	0.395181	0.978343	Biso	1.000000	O
O492	1.0	0.227102	0.134888	0.187593	Biso	1.000000	O
O493	1.0	0.111427	0.067929	0.896805	Biso	1.000000	O
O494	1.0	0.033123	0.782290	0.487722	Biso	1.000000	O
O495	1.0	0.233613	0.519854	0.974698	Biso	1.000000	O
O496	1.0	0.623337	0.751413	0.398710	Biso	1.000000	O
O497	1.0	0.683717	0.186683	0.175309	Biso	1.000000	O
O498	1.0	0.365095	0.800515	0.553151	Biso	1.000000	O
O499	1.0	0.350178	0.514756	0.391026	Biso	1.000000	O
O500	1.0	0.277536	0.604083	0.218286	Biso	1.000000	O
O501	1.0	0.347856	0.812754	0.212682	Biso	1.000000	O
O502	1.0	0.475216	0.615484	0.648345	Biso	1.000000	O
O503	1.0	0.617112	0.630075	0.188121	Biso	1.000000	O
O504	1.0	0.932489	0.904625	0.392075	Biso	1.000000	O
O505	1.0	0.113556	0.244545	0.838720	Biso	1.000000	O
O506	1.0	0.507313	0.370785	0.894003	Biso	1.000000	O
O507	1.0	0.143922	0.042544	0.007884	Biso	1.000000	O
O508	1.0	0.659468	0.994668	0.898021	Biso	1.000000	O
O509	1.0	0.572335	0.709760	0.985605	Biso	1.000000	O
O510	1.0	0.756340	0.759663	0.449518	Biso	1.000000	O
O511	1.0	0.184843	0.208123	0.071928	Biso	1.000000	O
O512	1.0	0.800749	0.797150	0.892919	Biso	1.000000	O
O513	1.0	0.717854	0.251358	0.187575	Biso	1.000000	O
O514	1.0	0.153939	0.417115	0.815513	Biso	1.000000	O
O515	1.0	0.048165	0.889092	0.994522	Biso	1.000000	O
O516	1.0	0.029066	0.042978	0.303807	Biso	1.000000	O
O517	1.0	0.521608	0.944739	0.051711	Biso	1.000000	O
O518	1.0	0.189653	0.475898	0.265129	Biso	1.000000	O
O519	1.0	0.084138	0.211034	0.497802	Biso	1.000000	O
O520	1.0	0.214961	0.848844	0.986094	Biso	1.000000	O
O521	1.0	0.801332	0.910563	0.985396	Biso	1.000000	O
O522	1.0	0.317507	0.471246	0.261864	Biso	1.000000	O

O523	1.0	0.502935	0.510241	0.191975	Biso	1.000000	O
O524	1.0	0.780853	0.724050	0.753240	Biso	1.000000	O
O525	1.0	0.592088	0.288218	0.705479	Biso	1.000000	O
O526	1.0	0.660479	0.139524	0.999098	Biso	1.000000	O
O527	1.0	0.738962	0.291273	0.764546	Biso	1.000000	O
O528	1.0	0.378217	0.960016	0.479337	Biso	1.000000	O
O529	1.0	0.361011	0.120765	0.895658	Biso	1.000000	O
O530	1.0	0.934583	0.487663	0.505232	Biso	1.000000	O
O531	1.0	0.642702	0.391379	0.501348	Biso	1.000000	O
O532	1.0	0.899103	0.232391	0.857980	Biso	1.000000	O
O533	1.0	0.246245	0.177374	0.950377	Biso	1.000000	O
O534	1.0	0.832423	0.105585	0.063190	Biso	1.000000	O
O535	1.0	0.018490	0.612366	0.512275	Biso	1.000000	O
O536	1.0	0.195954	0.395778	0.082963	Biso	1.000000	O
O537	1.0	0.945658	0.991090	0.856162	Biso	1.000000	O
O538	1.0	0.057254	0.551378	0.401386	Biso	1.000000	O
O539	1.0	0.357714	0.191559	0.573913	Biso	1.000000	O
O540	1.0	0.591431	0.785856	0.081081	Biso	1.000000	O
O541	1.0	0.841676	0.193774	0.839586	Biso	1.000000	O
O542	1.0	0.715969	0.356007	0.213886	Biso	1.000000	O
O543	1.0	0.731415	0.777701	0.256687	Biso	1.000000	O
O544	1.0	0.435696	0.973386	0.157282	Biso	1.000000	O
O545	1.0	0.003715	0.009867	0.195364	Biso	1.000000	O
O546	1.0	0.383589	0.810502	0.677887	Biso	1.000000	O
O547	1.0	0.454025	0.729272	0.396417	Biso	1.000000	O
O548	1.0	0.028307	0.177255	0.990799	Biso	1.000000	O
O549	1.0	0.041971	0.143719	0.054244	Biso	1.000000	O
O550	1.0	0.570587	0.856014	0.975731	Biso	1.000000	O
O551	1.0	0.569543	0.686665	0.457141	Biso	1.000000	O
O552	1.0	0.260529	0.861501	0.115992	Biso	1.000000	O
O553	1.0	0.762661	0.258583	0.554887	Biso	1.000000	O
O554	1.0	0.519949	0.206920	0.164389	Biso	1.000000	O
O555	1.0	0.402051	0.339509	0.219019	Biso	1.000000	O
O556	1.0	0.929513	0.356198	0.209498	Biso	1.000000	O
O557	1.0	0.158087	0.535572	0.500263	Biso	1.000000	O
O558	1.0	0.766918	0.533822	0.920688	Biso	1.000000	O
O559	1.0	0.756016	0.931676	0.172807	Biso	1.000000	O
O560	1.0	0.561417	0.922049	0.594871	Biso	1.000000	O
O561	1.0	0.427924	0.368982	0.573294	Biso	1.000000	O
O562	1.0	0.544330	0.098801	0.535668	Biso	1.000000	O
O563	1.0	0.351198	0.919505	0.820046	Biso	1.000000	O
O564	1.0	0.562656	0.601260	0.069767	Biso	1.000000	O
O565	1.0	0.519597	0.670407	0.636899	Biso	1.000000	O
O566	1.0	0.260928	0.852328	0.785935	Biso	1.000000	O
O567	1.0	0.274766	0.710240	0.296401	Biso	1.000000	O
O568	1.0	0.969176	0.756746	0.224698	Biso	1.000000	O
O569	1.0	0.155217	0.112823	0.864167	Biso	1.000000	O
O570	1.0	0.156567	0.072382	0.205348	Biso	1.000000	O
O571	1.0	0.693256	0.955802	0.506210	Biso	1.000000	O
O572	1.0	0.468356	0.167030	0.755538	Biso	1.000000	O
O573	1.0	0.052285	0.364811	0.861676	Biso	1.000000	O
O574	1.0	0.864963	0.833041	0.806392	Biso	1.000000	O
O575	1.0	0.368574	0.710226	0.789048	Biso	1.000000	O
O576	1.0	0.215570	0.722465	0.943465	Biso	1.000000	O
O577	1.0	0.728566	0.160099	0.999110	Biso	1.000000	O
O578	1.0	0.992925	0.945048	0.284626	Biso	1.000000	O
O579	1.0	0.224187	0.346085	0.883836	Biso	1.000000	O
O580	1.0	0.750366	0.529713	0.154534	Biso	1.000000	O
O581	1.0	0.122036	0.343277	0.079734	Biso	1.000000	O
O582	1.0	0.494543	0.148307	0.207488	Biso	1.000000	O
O583	1.0	0.631894	0.701179	0.646276	Biso	1.000000	O
O584	1.0	0.421167	0.907692	0.067722	Biso	1.000000	O
O585	1.0	0.819387	0.028023	0.318804	Biso	1.000000	O
O586	1.0	0.283777	0.797806	0.827277	Biso	1.000000	O
O587	1.0	0.157170	0.597106	0.884880	Biso	1.000000	O
O588	1.0	0.829148	0.923094	0.427513	Biso	1.000000	O
O589	1.0	0.138787	0.391956	0.401174	Biso	1.000000	O
O590	1.0	0.409017	0.503724	0.527545	Biso	1.000000	O
O591	1.0	0.342901	0.104344	0.098648	Biso	1.000000	O
O592	1.0	0.734540	0.379535	0.331473	Biso	1.000000	O
O593	1.0	0.475894	0.498775	0.327976	Biso	1.000000	O
O594	1.0	0.704429	0.514234	0.557057	Biso	1.000000	O
O595	1.0	0.552609	0.156776	0.338623	Biso	1.000000	O
O596	1.0	0.923107	0.020948	0.569701	Biso	1.000000	O
O597	1.0	0.632883	0.139363	0.860826	Biso	1.000000	O
O598	1.0	0.491399	0.328749	0.423849	Biso	1.000000	O
O599	1.0	0.405005	0.446816	0.571746	Biso	1.000000	O
O600	1.0	0.575682	0.042482	0.948191	Biso	1.000000	O
O601	1.0	0.631056	0.504874	0.949985	Biso	1.000000	O
O602	1.0	0.063176	0.447503	0.771066	Biso	1.000000	O
O603	1.0	0.128911	0.202259	0.342932	Biso	1.000000	O
O604	1.0	0.248539	0.445129	0.065153	Biso	1.000000	O
O605	1.0	0.395068	0.560070	0.032477	Biso	1.000000	O
O606	1.0	0.848291	0.732347	0.396068	Biso	1.000000	O
O607	1.0	0.192882	0.410476	0.234291	Biso	1.000000	O

O608	1.0	0.482940	0.815798	0.284438	Biso	1.000000	O
O609	1.0	0.653376	0.452780	0.226873	Biso	1.000000	O
O610	1.0	0.927699	0.450823	0.038111	Biso	1.000000	O
O611	1.0	0.402389	0.581300	0.885635	Biso	1.000000	O
O612	1.0	0.824726	0.455704	0.989672	Biso	1.000000	O
O613	1.0	0.686528	0.321547	0.269014	Biso	1.000000	O
O614	1.0	0.212516	0.599531	0.841396	Biso	1.000000	O
O615	1.0	0.651175	0.578849	0.607249	Biso	1.000000	O
O616	1.0	0.533697	0.331688	0.555225	Biso	1.000000	O
O617	1.0	0.425879	0.460918	0.949794	Biso	1.000000	O
O618	1.0	0.264141	0.383681	0.103640	Biso	1.000000	O
O619	1.0	0.095600	0.835120	0.756004	Biso	1.000000	O
O620	1.0	0.118099	0.955220	0.724208	Biso	1.000000	O
O621	1.0	0.760284	0.204432	0.951154	Biso	1.000000	O
O622	1.0	0.937065	0.884213	0.459480	Biso	1.000000	O
O623	1.0	0.278369	0.670762	0.507304	Biso	1.000000	O
O624	1.0	0.249100	0.928663	0.549542	Biso	1.000000	O
O625	1.0	0.581684	0.452254	0.579749	Biso	1.000000	O
O626	1.0	0.446163	0.578432	0.081463	Biso	1.000000	O
O627	1.0	0.638396	0.328400	0.105640	Biso	1.000000	O
O628	1.0	0.284758	0.422627	0.590608	Biso	1.000000	O
O629	1.0	0.055028	0.114441	0.668098	Biso	1.000000	O
O630	1.0	0.922629	0.869969	0.069503	Biso	1.000000	O
O631	1.0	0.805437	0.085014	0.818361	Biso	1.000000	O
O632	1.0	0.467439	0.324981	0.532577	Biso	1.000000	O
O633	1.0	0.596463	0.946349	0.363836	Biso	1.000000	O
O634	1.0	0.756547	0.273208	0.414263	Biso	1.000000	O
O635	1.0	0.141915	0.397246	0.746745	Biso	1.000000	O
O636	1.0	0.309364	0.402880	0.238567	Biso	1.000000	O
O637	1.0	0.520880	0.539019	0.591080	Biso	1.000000	O
O638	1.0	0.970672	0.591761	0.463953	Biso	1.000000	O
O639	1.0	0.252948	0.445240	0.249743	Biso	1.000000	O
O640	1.0	0.881150	0.381318	0.347313	Biso	1.000000	O
O641	1.0	0.915061	0.139266	0.581845	Biso	1.000000	O
O642	1.0	0.646107	0.305301	0.615053	Biso	1.000000	O
O643	1.0	0.111287	0.221086	0.961218	Biso	1.000000	O
O644	1.0	0.383045	0.051619	0.901090	Biso	1.000000	O
O645	1.0	0.948815	0.523571	0.358333	Biso	1.000000	O
O646	1.0	0.443934	0.013739	0.912631	Biso	1.000000	O
O647	1.0	0.173500	0.872973	0.853338	Biso	1.000000	O
O648	1.0	0.604117	0.979728	0.599180	Biso	1.000000	O
O649	1.0	0.103373	0.690362	0.738254	Biso	1.000000	O
O650	1.0	0.220921	0.795602	0.034964	Biso	1.000000	O
O651	1.0	0.556083	0.474910	0.480365	Biso	1.000000	O
O652	1.0	0.061678	0.948714	0.657035	Biso	1.000000	O
O653	1.0	0.880968	0.243550	0.211754	Biso	1.000000	O
O654	1.0	0.505417	0.519962	0.264899	Biso	1.000000	O
O655	1.0	0.309161	0.506090	0.450125	Biso	1.000000	O
O656	1.0	0.884706	0.691276	0.871208	Biso	1.000000	O
O657	1.0	0.308364	0.072752	0.619188	Biso	1.000000	O
O658	1.0	0.829544	0.628635	0.982669	Biso	1.000000	O
O659	1.0	0.390429	0.438902	0.504090	Biso	1.000000	O
O660	1.0	0.767404	0.130983	0.176339	Biso	1.000000	O
O661	1.0	0.215740	0.235269	0.840573	Biso	1.000000	O
O662	1.0	0.820052	0.109902	0.994696	Biso	1.000000	O
O663	1.0	0.906715	0.404090	0.285776	Biso	1.000000	O
O664	1.0	0.989796	0.362175	0.165990	Biso	1.000000	O
O665	1.0	0.283975	0.172010	0.705043	Biso	1.000000	O
O666	1.0	0.860349	0.107174	0.128202	Biso	1.000000	O
O667	1.0	0.071821	0.638852	0.553851	Biso	1.000000	O
O668	1.0	0.159981	0.355414	0.269105	Biso	1.000000	O
O669	1.0	0.001174	0.792163	0.828474	Biso	1.000000	O
O670	1.0	0.553673	0.639244	0.151455	Biso	1.000000	O
O671	1.0	0.225825	0.497011	0.906517	Biso	1.000000	O
O672	1.0	0.270837	0.689865	0.974254	Biso	1.000000	O
O673	1.0	0.942162	0.372012	0.759081	Biso	1.000000	O
O674	1.0	0.935614	0.965588	0.731895	Biso	1.000000	O
O675	1.0	0.116443	0.879837	0.806668	Biso	1.000000	O
O676	1.0	0.953454	0.642445	0.281074	Biso	1.000000	O
O677	1.0	0.996352	0.019002	0.980829	Biso	1.000000	O
O678	1.0	0.811233	0.124971	0.759526	Biso	1.000000	O

O679	1.0	0.552780	0.272682	0.174533	Biso	1.000000	O
O680	1.0	0.649089	0.519814	0.198039	Biso	1.000000	O
O681	1.0	0.381685	0.384152	0.457695	Biso	1.000000	O
O682	1.0	0.252567	0.456772	0.643531	Biso	1.000000	O
O683	1.0	0.650347	0.785226	0.912258	Biso	1.000000	O
O684	1.0	0.614087	0.076065	0.897997	Biso	1.000000	O
O685	1.0	0.941511	0.491468	0.152513	Biso	1.000000	O
O686	1.0	0.472987	0.449312	0.002584	Biso	1.000000	O
O687	1.0	0.374012	0.091507	0.774057	Biso	1.000000	O
O688	1.0	0.551782	0.025998	0.691420	Biso	1.000000	O
O689	1.0	0.363559	0.265343	0.967127	Biso	1.000000	O
O690	1.0	0.490291	0.617691	0.125215	Biso	1.000000	O
O691	1.0	0.970260	0.452606	0.658523	Biso	1.000000	O
O692	1.0	0.085455	0.502889	0.927304	Biso	1.000000	O
O693	1.0	0.932151	0.980638	0.294384	Biso	1.000000	O
O694	1.0	0.229013	0.424561	0.447623	Biso	1.000000	O
O695	1.0	0.297047	0.878497	0.529777	Biso	1.000000	O
O696	1.0	0.344600	0.516014	0.677510	Biso	1.000000	O
O697	1.0	0.981426	0.788773	0.438574	Biso	1.000000	O
O698	1.0	0.528688	0.311745	0.080443	Biso	1.000000	O
O699	1.0	0.811112	0.273770	0.368135	Biso	1.000000	O
O700	1.0	0.935138	0.335087	0.582777	Biso	1.000000	O
O701	1.0	0.248934	0.482392	0.780498	Biso	1.000000	O
O702	1.0	0.474410	0.918539	0.259027	Biso	1.000000	O
O703	1.0	0.744367	0.455633	0.573199	Biso	1.000000	O
O704	1.0	0.786468	0.150697	0.699766	Biso	1.000000	O
O705	1.0	0.810626	0.476227	0.924366	Biso	1.000000	O
O706	1.0	0.399284	0.918948	0.621303	Biso	1.000000	O
O707	1.0	0.100181	0.820475	0.409444	Biso	1.000000	O
O708	1.0	0.098483	0.665438	0.357848	Biso	1.000000	O
O709	1.0	0.628937	0.737487	0.168132	Biso	1.000000	O
O710	1.0	0.746101	0.596773	0.786298	Biso	1.000000	O
O711	1.0	0.683492	0.719611	0.932578	Biso	1.000000	O
O712	1.0	0.271232	0.188181	0.014811	Biso	1.000000	O
O713	1.0	0.285983	0.327522	0.594457	Biso	1.000000	O
O714	1.0	0.779746	0.339554	0.930095	Biso	1.000000	O
O715	1.0	0.458090	0.642501	0.939485	Biso	1.000000	O
O716	1.0	0.252635	0.181828	0.317425	Biso	1.000000	O
O717	1.0	0.694641	0.582907	0.528646	Biso	1.000000	O
O718	1.0	0.022813	0.230157	0.192628	Biso	1.000000	O
O719	1.0	0.710547	0.992330	0.085639	Biso	1.000000	O
O720	1.0	0.795314	0.701139	0.358826	Biso	1.000000	O
O721	1.0	0.105022	0.240695	0.613542	Biso	1.000000	O
O722	1.0	0.150505	0.897764	0.087982	Biso	1.000000	O
O723	1.0	0.899320	0.256894	0.734694	Biso	1.000000	O
O724	1.0	0.523365	0.502091	0.001909	Biso	1.000000	O
O725	1.0	0.172164	0.799578	0.187876	Biso	1.000000	O
O726	1.0	0.061397	0.494438	0.445682	Biso	1.000000	O
O727	1.0	0.647902	0.195299	0.788143	Biso	1.000000	O
O728	1.0	0.132324	0.296487	0.711819	Biso	1.000000	O
O729	1.0	0.681112	0.025815	0.531170	Biso	1.000000	O
O730	1.0	0.752933	0.231339	0.353500	Biso	1.000000	O
O731	1.0	0.227207	0.244712	0.909461	Biso	1.000000	O
O732	1.0	0.098335	0.336138	0.297922	Biso	1.000000	O
O733	1.0	0.816812	0.854578	0.362146	Biso	1.000000	O
O734	1.0	0.274113	0.853249	0.379376	Biso	1.000000	O
O735	1.0	0.640875	0.688874	0.884662	Biso	1.000000	O
O736	1.0	0.473732	0.511863	0.549133	Biso	1.000000	O
O737	1.0	0.055235	0.972591	0.306508	Biso	1.000000	O
O738	1.0	0.893046	0.911775	0.712746	Biso	1.000000	O
O739	1.0	0.784973	0.780109	0.559575	Biso	1.000000	O
O740	1.0	0.413931	0.294985	0.841778	Biso	1.000000	O
O741	1.0	0.778729	0.202478	0.513126	Biso	1.000000	O
O742	1.0	0.115365	0.294025	0.961767	Biso	1.000000	O
O743	1.0	0.014458	0.052562	0.918333	Biso	1.000000	O
O744	1.0	0.723303	0.489298	0.667098	Biso	1.000000	O
O745	1.0	0.085074	0.433026	0.090767	Biso	1.000000	O
O746	1.0	0.285604	0.904253	0.291822	Biso	1.000000	O
O747	1.0	0.968761	0.960408	0.158081	Biso	1.000000	O
O748	1.0	0.687740	0.283569	0.133276	Biso	1.000000	O
O749	1.0	0.855403	0.025131	0.586653	Biso	1.000000	O
O750	1.0	0.576386	0.903588	0.029964	Biso	1.000000	O
O751	1.0	0.335636	0.091446	0.168081	Biso	1.000000	O
O752	1.0	0.857824	0.451223	0.051136	Biso	1.000000	O
O753	1.0	0.893189	0.553081	0.638933	Biso	1.000000	O
O754	1.0	0.305025	0.055802	0.064249	Biso	1.000000	O
O755	1.0	0.747766	0.623964	0.851888	Biso	1.000000	O
O756	1.0	0.037535	0.834134	0.438814	Biso	1.000000	O
O757	1.0	0.599549	0.770138	0.013982	Biso	1.000000	O
O758	1.0	0.295170	0.511383	0.938877	Biso	1.000000	O
O759	1.0	0.217670	0.307840	0.943127	Biso	1.000000	O
O760	1.0	0.027260	0.687170	0.519839	Biso	1.000000	O
O761	1.0	0.597681	0.463786	0.117856	Biso	1.000000	O
O762	1.0	0.243754	0.872914	0.642253	Biso	1.000000	O

O763	1.0	0.743526	0.314533	0.164873	Biso	1.000000	O
O764	1.0	0.319672	0.000357	0.389434	Biso	1.000000	O
O765	1.0	0.262687	0.563952	0.572621	Biso	1.000000	O
O766	1.0	0.268234	0.660730	0.438903	Biso	1.000000	O
O767	1.0	0.480585	0.617165	0.733457	Biso	1.000000	O
O768	1.0	0.276404	0.074757	0.859310	Biso	1.000000	O
O769	1.0	0.276613	0.710609	0.776886	Biso	1.000000	O
O770	1.0	0.057343	0.016270	0.365793	Biso	1.000000	O
O771	1.0	0.327246	0.635933	0.470522	Biso	1.000000	O
O772	1.0	0.602223	0.135824	0.152286	Biso	1.000000	O
O773	1.0	0.204949	0.703121	0.292097	Biso	1.000000	O
O774	1.0	0.966092	0.394540	0.555386	Biso	1.000000	O
O775	1.0	0.219328	0.090029	0.242441	Biso	1.000000	O
O776	1.0	0.494051	0.671938	0.172095	Biso	1.000000	O
O777	1.0	0.833580	0.427304	0.647167	Biso	1.000000	O
O778	1.0	0.967236	0.660954	0.976517	Biso	1.000000	O
O779	1.0	0.958721	0.081265	0.583973	Biso	1.000000	O
O780	1.0	0.564335	0.641170	0.011081	Biso	1.000000	O
O781	1.0	0.134035	0.183367	0.873519	Biso	1.000000	O
O782	1.0	0.424651	0.904261	0.307713	Biso	1.000000	O
O783	1.0	0.768602	0.054778	0.621006	Biso	1.000000	O
O784	1.0	0.609168	0.256239	0.797010	Biso	1.000000	O
O785	1.0	0.074956	0.388994	0.728823	Biso	1.000000	O
O786	1.0	0.823006	0.535918	0.627760	Biso	1.000000	O
O787	1.0	0.096256	0.937026	0.927113	Biso	1.000000	O
O788	1.0	0.020480	0.235762	0.519152	Biso	1.000000	O
O789	1.0	0.245964	0.051059	0.801102	Biso	1.000000	O
O790	1.0	0.976120	0.076082	0.387201	Biso	1.000000	O
O791	1.0	0.442544	0.147212	0.335033	Biso	1.000000	O
O792	1.0	0.702225	0.395519	0.830248	Biso	1.000000	O
O793	1.0	0.110492	0.295592	0.454353	Biso	1.000000	O
O794	1.0	0.636560	0.241379	0.735010	Biso	1.000000	O
O795	1.0	0.827733	0.476341	0.338727	Biso	1.000000	O
O796	1.0	0.649386	0.898627	0.505338	Biso	1.000000	O
O797	1.0	0.051221	0.068197	0.855505	Biso	1.000000	O
O798	1.0	0.053182	0.861648	0.375783	Biso	1.000000	O
O799	1.0	0.316998	0.198572	0.066188	Biso	1.000000	O
O800	1.0	0.160425	0.298994	0.818895	Biso	1.000000	O
O801	1.0	0.023971	0.420807	0.139589	Biso	1.000000	O
O802	1.0	0.645395	0.958895	0.270497	Biso	1.000000	O
O803	1.0	0.322134	0.973230	0.611449	Biso	1.000000	O
O804	1.0	0.490891	0.761151	0.447432	Biso	1.000000	O
O805	1.0	0.901087	0.729635	0.496970	Biso	1.000000	O
O806	1.0	0.834397	0.790968	0.703198	Biso	1.000000	O
O807	1.0	0.254369	0.115237	0.913920	Biso	1.000000	O
O808	1.0	0.363935	0.493626	0.933606	Biso	1.000000	O
O809	1.0	0.290597	0.282700	0.273393	Biso	1.000000	O
O810	1.0	0.235553	0.462883	0.714417	Biso	1.000000	O
O811	1.0	0.983338	0.913011	0.974970	Biso	1.000000	O
O812	1.0	0.089262	0.050240	0.785794	Biso	1.000000	O
O813	1.0	0.588516	0.216093	0.690251	Biso	1.000000	O
O814	1.0	0.576072	0.965386	0.724080	Biso	1.000000	O
O815	1.0	0.485224	0.152619	0.277304	Biso	1.000000	O
O816	1.0	0.538622	0.564321	0.220786	Biso	1.000000	O
O817	1.0	0.502743	0.835693	0.985886	Biso	1.000000	O
O818	1.0	0.001886	0.705415	0.075754	Biso	1.000000	O
O819	1.0	0.719406	0.809060	0.566935	Biso	1.000000	O
O820	1.0	0.759278	0.157612	0.363553	Biso	1.000000	O
O821	1.0	0.871732	0.308357	0.099346	Biso	1.000000	O
O822	1.0	0.967087	0.236278	0.747350	Biso	1.000000	O
O823	1.0	0.266871	0.350462	0.264939	Biso	1.000000	O
O824	1.0	0.172269	0.675953	0.731393	Biso	1.000000	O
O825	1.0	0.863353	0.939610	0.834402	Biso	1.000000	O
O826	1.0	0.971110	0.003908	0.779263	Biso	1.000000	O
O827	1.0	0.982792	0.985780	0.916412	Biso	1.000000	O
O828	1.0	0.600478	0.748684	0.230530	Biso	1.000000	O
O829	1.0	0.496711	0.211554	0.490742	Biso	1.000000	O
O830	1.0	0.044280	0.259121	0.258706	Biso	1.000000	O
O831	1.0	0.274040	0.724405	0.847398	Biso	1.000000	O
O832	1.0	0.643557	0.997415	0.384566	Biso	1.000000	O
O833	1.0	0.762122	0.385362	0.166877	Biso	1.000000	O
O834	1.0	0.573346	0.544437	0.482423	Biso	1.000000	O

O835	1.0	0.625183	0.344243	0.449428	Biso	1.000000	O
O836	1.0	0.651323	0.544724	0.127975	Biso	1.000000	O
O837	1.0	0.126439	0.687213	0.562775	Biso	1.000000	O
O838	1.0	0.661996	0.682541	0.707139	Biso	1.000000	O
O839	1.0	0.152574	0.279896	0.577947	Biso	1.000000	O
O840	1.0	0.787622	0.203718	0.885657	Biso	1.000000	O
O841	1.0	0.578161	0.829301	0.322718	Biso	1.000000	O
O842	1.0	0.565611	0.618313	0.483377	Biso	1.000000	O
O843	1.0	0.084852	0.533022	0.340436	Biso	1.000000	O
O844	1.0	0.317935	0.856574	0.001900	Biso	1.000000	O
O845	1.0	0.181904	0.456079	0.666000	Biso	1.000000	O
O846	1.0	0.880594	0.235167	0.094530	Biso	1.000000	O
O847	1.0	0.448139	0.712276	0.962750	Biso	1.000000	O
O848	1.0	0.965964	0.279956	0.622960	Biso	1.000000	O
O849	1.0	0.932143	0.243642	0.484012	Biso	1.000000	O
O850	1.0	0.313459	0.008571	0.117757	Biso	1.000000	O
O851	1.0	0.231550	0.393242	0.554094	Biso	1.000000	O
O852	1.0	0.475271	0.649746	0.288935	Biso	1.000000	O
O853	1.0	0.334553	0.314002	0.011133	Biso	1.000000	O
O854	1.0	0.748813	0.858582	0.664239	Biso	1.000000	O
O855	1.0	0.304470	0.927323	0.005266	Biso	1.000000	O
O856	1.0	0.710566	0.485632	0.194769	Biso	1.000000	O
O857	1.0	0.809675	0.020882	0.478969	Biso	1.000000	O
O858	1.0	0.065887	0.632284	0.229381	Biso	1.000000	O
O859	1.0	0.309820	0.863719	0.836498	Biso	1.000000	O
O860	1.0	0.412422	0.560913	0.614060	Biso	1.000000	O
O861	1.0	0.261776	0.191370	0.871587	Biso	1.000000	O
O862	1.0	0.011698	0.334366	0.450829	Biso	1.000000	O
O863	1.0	0.288753	0.311819	0.208650	Biso	1.000000	O
O864	1.0	0.851674	0.672125	0.052150	Biso	1.000000	O
O865	1.0	0.133214	0.641644	0.781059	Biso	1.000000	O
O866	1.0	0.230487	0.316564	0.820340	Biso	1.000000	O
O867	1.0	0.844845	0.140181	0.518992	Biso	1.000000	O
O868	1.0	0.963513	0.196102	0.166841	Biso	1.000000	O
O869	1.0	0.689878	0.425640	0.658994	Biso	1.000000	O
O870	1.0	0.160398	0.750221	0.909044	Biso	1.000000	O
O871	1.0	0.159379	0.476772	0.542962	Biso	1.000000	O
O872	1.0	0.729299	0.119925	0.597033	Biso	1.000000	O
O873	1.0	0.194368	0.029974	0.960120	Biso	1.000000	O
O874	1.0	0.291150	0.646659	0.745382	Biso	1.000000	O
O875	1.0	0.065064	0.313378	0.049524	Biso	1.000000	O
O876	1.0	0.439623	0.840129	0.335099	Biso	1.000000	O
O877	1.0	0.206330	0.645497	0.677042	Biso	1.000000	O
O878	1.0	0.531334	0.694738	0.288194	Biso	1.000000	O
O879	1.0	0.890086	0.799970	0.509999	Biso	1.000000	O
O880	1.0	0.346001	0.254883	0.842127	Biso	1.000000	O
O881	1.0	0.789969	0.801199	0.401880	Biso	1.000000	O
O882	1.0	0.205961	0.985115	0.906321	Biso	1.000000	O
O883	1.0	0.629355	0.754506	0.594132	Biso	1.000000	O
O884	1.0	0.238994	0.689431	0.212524	Biso	1.000000	O
O885	1.0	0.334506	0.929768	0.388706	Biso	1.000000	O
O886	1.0	0.580054	0.210972	0.201295	Biso	1.000000	O
O887	1.0	0.612102	0.103702	0.962661	Biso	1.000000	O
O888	1.0	0.723982	0.233345	0.898051	Biso	1.000000	O
O889	1.0	0.345869	0.158944	0.144822	Biso	1.000000	O
O890	1.0	0.985350	0.378441	0.097474	Biso	1.000000	O
O891	1.0	0.241738	0.244279	0.976554	Biso	1.000000	O
O892	1.0	0.396373	0.973384	0.573013	Biso	1.000000	O
O893	1.0	0.136362	0.513822	0.294417	Biso	1.000000	O
O894	1.0	0.844608	0.740131	0.904934	Biso	1.000000	O
O895	1.0	0.970951	0.016801	0.345838	Biso	1.000000	O
O896	1.0	0.380347	0.151806	0.466082	Biso	1.000000	O
O897	1.0	0.587875	0.161096	0.278553	Biso	1.000000	O
O898	1.0	0.413346	0.720122	0.339844	Biso	1.000000	O
O899	1.0	0.317320	0.058875	0.688640	Biso	1.000000	O
O900	1.0	0.731830	0.284304	0.059395	Biso	1.000000	O
O901	1.0	0.926091	0.808608	0.830894	Biso	1.000000	O
O902	1.0	0.658924	0.234089	0.601067	Biso	1.000000	O
O903	1.0	0.495810	0.464153	0.436167	Biso	1.000000	O
O904	1.0	0.494056	0.330589	0.950623	Biso	1.000000	O
O905	1.0	0.870124	0.522728	0.126610	Biso	1.000000	O
O906	1.0	0.578444	0.187125	0.627144	Biso	1.000000	O

O907	1.0	0.561692	0.030467	0.517179	Biso	1.000000	O
O908	1.0	0.450049	0.657447	0.011417	Biso	1.000000	O
O909	1.0	0.255513	0.113883	0.346751	Biso	1.000000	O
O910	1.0	0.297083	0.302802	0.448389	Biso	1.000000	O
O911	1.0	0.196046	0.285787	0.522039	Biso	1.000000	O
O912	1.0	0.323063	0.792365	0.972835	Biso	1.000000	O
O913	1.0	0.512567	0.663873	0.491955	Biso	1.000000	O
O914	1.0	0.723004	0.429301	0.425866	Biso	1.000000	O
O915	1.0	0.679065	0.480941	0.449719	Biso	1.000000	O
O916	1.0	0.889523	0.617975	0.299931	Biso	1.000000	O
O917	1.0	0.036762	0.231921	0.591131	Biso	1.000000	O
O918	1.0	0.465639	0.963271	0.314146	Biso	1.000000	O
O919	1.0	0.798378	0.867865	0.795778	Biso	1.000000	O
O920	1.0	0.863985	0.165414	0.085771	Biso	1.000000	O
O921	1.0	0.977763	0.535915	0.103178	Biso	1.000000	O
O922	1.0	0.465133	0.773069	0.340329	Biso	1.000000	O
O923	1.0	0.865736	0.695607	0.656476	Biso	1.000000	O
O924	1.0	0.546630	0.089400	0.726023	Biso	1.000000	O
O925	1.0	0.398547	0.691614	0.161303	Biso	1.000000	O
O926	1.0	0.706488	0.548066	0.322634	Biso	1.000000	O
O927	1.0	0.240750	0.851014	0.852204	Biso	1.000000	O
O928	1.0	0.855411	0.998168	0.793666	Biso	1.000000	O
O929	1.0	0.657069	0.047482	0.334649	Biso	1.000000	O
O930	1.0	0.465523	0.152310	0.042896	Biso	1.000000	O
O931	1.0	0.414104	0.241017	0.264496	Biso	1.000000	O
O932	1.0	0.534280	0.913127	0.211368	Biso	1.000000	O
O933	1.0	0.167056	0.974189	0.997674	Biso	1.000000	O
O934	1.0	0.167051	0.356598	0.923507	Biso	1.000000	O
O935	1.0	0.186876	0.167670	0.830134	Biso	1.000000	O
O936	1.0	0.404071	0.128740	0.642926	Biso	1.000000	O
O937	1.0	0.021055	0.306787	0.831360	Biso	1.000000	O
O938	1.0	0.839997	0.532133	0.233701	Biso	1.000000	O
O939	1.0	0.735727	0.596501	0.187660	Biso	1.000000	O
O940	1.0	0.967322	0.704748	0.606638	Biso	1.000000	O
O941	1.0	0.791197	0.802420	0.034023	Biso	1.000000	O
O942	1.0	0.344171	0.069372	0.234894	Biso	1.000000	O
O943	1.0	0.220764	0.918023	0.097194	Biso	1.000000	O
O944	1.0	0.912923	0.722286	0.342638	Biso	1.000000	O
O945	1.0	0.633147	0.041992	0.580223	Biso	1.000000	O
O946	1.0	0.030434	0.203050	0.749607	Biso	1.000000	O
O947	1.0	0.924000	0.474018	0.272501	Biso	1.000000	O
O948	1.0	0.697092	0.939934	0.134906	Biso	1.000000	O
O949	1.0	0.026292	0.997832	0.499508	Biso	1.000000	O
O950	1.0	0.763755	0.034357	0.688566	Biso	1.000000	O
O951	1.0	0.828848	0.066184	0.885431	Biso	1.000000	O
O952	1.0	0.612347	0.621769	0.118639	Biso	1.000000	O
O953	1.0	0.628165	0.681624	0.576889	Biso	1.000000	O
O954	1.0	0.623518	0.943787	0.858239	Biso	1.000000	O
O955	1.0	0.731668	0.984734	0.830929	Biso	1.000000	O
O956	1.0	0.372644	0.802960	0.772405	Biso	1.000000	O
O957	1.0	0.648215	0.583515	0.475747	Biso	1.000000	O
O958	1.0	0.011720	0.616913	0.937386	Biso	1.000000	O
O959	1.0	0.158430	0.790465	0.308149	Biso	1.000000	O
O960	1.0	0.515813	0.562765	0.767008	Biso	1.000000	O
O961	1.0	0.354930	0.283214	0.484571	Biso	1.000000	O
O962	1.0	0.830610	0.237637	0.548329	Biso	1.000000	O
O963	1.0	0.082238	0.001444	0.839565	Biso	1.000000	O
O964	1.0	0.032828	0.116559	0.177935	Biso	1.000000	O
O965	1.0	0.484886	0.424277	0.938439	Biso	1.000000	O
O966	1.0	0.221726	0.821748	0.415808	Biso	1.000000	O
O967	1.0	0.339395	0.898971	0.950345	Biso	1.000000	O
O968	1.0	0.168943	0.119117	0.153519	Biso	1.000000	O
O969	1.0	0.793371	0.504426	0.861913	Biso	1.000000	O
O970	1.0	0.817295	0.627936	0.280739	Biso	1.000000	O
O971	1.0	0.913865	0.757624	0.600050	Biso	1.000000	O
O972	1.0	0.445045	0.000175	0.981060	Biso	1.000000	O
O973	1.0	0.828777	0.300536	0.963217	Biso	1.000000	O
O974	1.0	0.770408	0.845295	0.532495	Biso	1.000000	O
O975	1.0	0.212449	0.512371	0.117294	Biso	1.000000	O

O976	1.0	0.654560	0.211191	0.422565	Biso	1.000000	0
O977	1.0	0.293978	0.731464	0.127337	Biso	1.000000	0
O978	1.0	0.028225	0.666060	0.355621	Biso	1.000000	0
O979	1.0	0.530125	0.084224	0.656233	Biso	1.000000	0
O980	1.0	0.374807	0.950770	0.917273	Biso	1.000000	0
O981	1.0	0.521389	0.298989	0.894017	Biso	1.000000	0
O982	1.0	0.904332	0.124168	0.966322	Biso	1.000000	0
O983	1.0	0.063747	0.934300	0.866471	Biso	1.000000	0
O984	1.0	0.645140	0.016254	0.645044	Biso	1.000000	0
O985	1.0	0.728228	0.361596	0.397159	Biso	1.000000	0
O986	1.0	0.562780	0.482974	0.834309	Biso	1.000000	0
O987	1.0	0.880438	0.576242	0.704683	Biso	1.000000	0
O988	1.0	0.386280	0.024219	0.140855	Biso	1.000000	0
O989	1.0	0.388261	0.882773	0.364763	Biso	1.000000	0
O990	1.0	0.861089	0.714099	0.108099	Biso	1.000000	0
O991	1.0	0.863119	0.006367	0.863584	Biso	1.000000	0
O992	1.0	0.949269	0.932471	0.094568	Biso	1.000000	0
O993	1.0	0.857412	0.792010	0.007486	Biso	1.000000	0
O994	1.0	0.771749	0.522532	0.330105	Biso	1.000000	0
O995	1.0	0.122198	0.366935	0.466085	Biso	1.000000	0
O996	1.0	0.741039	0.575632	0.974796	Biso	1.000000	0
O997	1.0	0.944968	0.266683	0.027679	Biso	1.000000	0
O998	1.0	0.809590	0.137120	0.869679	Biso	1.000000	0
O999	1.0	0.707419	0.051527	0.840808	Biso	1.000000	0
O1000	1.0	0.982700	0.633218	0.713539	Biso	1.000000	0
O1001	1.0	0.584210	0.483883	0.900502	Biso	1.000000	0
O1002	1.0	0.072965	0.176956	0.167872	Biso	1.000000	0
O1003	1.0	0.056067	0.703093	0.031190	Biso	1.000000	0
O1004	1.0	0.586010	0.956981	0.077015	Biso	1.000000	0
O1005	1.0	0.913989	0.814393	0.970751	Biso	1.000000	0
O1006	1.0	0.819637	0.635595	0.181655	Biso	1.000000	0
O1007	1.0	0.399001	0.110829	0.141235	Biso	1.000000	0
O1008	1.0	0.007095	0.840251	0.664326	Biso	1.000000	0
O1009	1.0	0.027836	0.455131	0.263167	Biso	1.000000	0
O1010	1.0	0.277635	0.771257	0.602953	Biso	1.000000	0
O1011	1.0	0.528652	0.972401	0.487365	Biso	1.000000	0
O1012	1.0	0.738379	0.873902	0.975834	Biso	1.000000	0
O1013	1.0	0.782947	0.552576	0.478749	Biso	1.000000	0
O1014	1.0	0.655025	0.321417	0.206084	Biso	1.000000	0
O1015	1.0	0.063516	0.328272	0.118238	Biso	1.000000	0
O1016	1.0	0.560587	0.494785	0.228175	Biso	1.000000	0
O1017	1.0	0.956686	0.750294	0.043568	Biso	1.000000	0
O1018	1.0	0.740179	0.802460	0.985341	Biso	1.000000	0
O1019	1.0	0.318556	0.707199	0.460873	Biso	1.000000	0
O1020	1.0	0.016229	0.251750	0.874972	Biso	1.000000	0
O1021	1.0	0.594320	0.217072	0.322285	Biso	1.000000	0
O1022	1.0	0.286052	0.912159	0.070527	Biso	1.000000	0
O1023	1.0	0.762452	0.592471	0.309607	Biso	1.000000	0
O1024	1.0	0.319092	0.473847	0.626839	Biso	1.000000	0
O1025	1.0	0.785566	0.411973	0.106072	Biso	1.000000	0
O1026	1.0	0.808587	0.087259	0.670312	Biso	1.000000	0
O1027	1.0	0.354781	0.226954	0.118633	Biso	1.000000	0
O1028	1.0	0.928298	0.651642	0.346868	Biso	1.000000	0
O1029	1.0	0.202505	0.427833	0.766787	Biso	1.000000	0
O1030	1.0	0.706848	0.021865	0.776387	Biso	1.000000	0
O1031	1.0	0.168607	0.844306	0.039543	Biso	1.000000	0
O1032	1.0	0.396720	0.321178	0.529189	Biso	1.000000	0
O1033	1.0	0.075926	0.174798	0.699943	Biso	1.000000	0
O1034	1.0	0.237866	0.749854	0.087581	Biso	1.000000	0
O1035	1.0	0.480956	0.231881	0.961077	Biso	1.000000	0
O1036	1.0	0.563135	0.073605	0.161489	Biso	1.000000	0
O1037	1.0	0.672101	0.250724	0.478653	Biso	1.000000	0
O1038	1.0	0.524183	0.645270	0.340237	Biso	1.000000	0
O1039	1.0	0.983130	0.183729	0.235135	Biso	1.000000	0
O1040	1.0	0.662590	0.458720	0.088923	Biso	1.000000	0
O1041	1.0	0.219830	0.145161	0.718462	Biso	1.000000	0
O1042	1.0	0.103133	0.578488	0.762690	Biso	1.000000	0
O1043	1.0	0.576729	0.025414	0.055250	Biso	1.000000	0
O1044	1.0	0.739052	0.008727	0.493296	Biso	1.000000	0
O1045	1.0	0.796086	0.181499	0.007816	Biso	1.000000	0
O1046	1.0	0.163851	0.075299	0.435793	Biso	1.000000	0
O1047	1.0	0.794769	0.963955	0.340218	Biso	1.000000	0
O1048	1.0	0.640823	0.256031	0.665850	Biso	1.000000	0
O1049	1.0	0.612869	0.901635	0.243013	Biso	1.000000	0
O1050	1.0	0.096468	0.352907	0.921095	Biso	1.000000	0
O1051	1.0	0.276915	0.015922	0.650144	Biso	1.000000	0
O1052	1.0	0.405481	0.685915	0.733884	Biso	1.000000	0
O1053	1.0	0.250574	0.154379	0.251808	Biso	1.000000	0
O1054	1.0	0.315877	0.754176	0.659744	Biso	1.000000	0

O1055	1.0	0.495708	0.821978	0.571958	Biso	1.000000	O
O1056	1.0	0.486301	0.920754	0.532678	Biso	1.000000	O
O1057	1.0	0.579403	0.604875	0.365968	Biso	1.000000	O
O1058	1.0	0.326910	0.383263	0.897187	Biso	1.000000	O
O1059	1.0	0.862419	0.427551	0.937547	Biso	1.000000	O
O1060	1.0	0.895008	0.984353	0.672740	Biso	1.000000	O
O1061	1.0	0.232845	0.764879	0.547457	Biso	1.000000	O
O1062	1.0	0.412048	0.636561	0.396515	Biso	1.000000	O
O1063	1.0	0.006451	0.240584	0.805585	Biso	1.000000	O
O1064	1.0	0.180684	0.052921	0.894641	Biso	1.000000	O
O1065	1.0	0.581989	0.602947	0.657163	Biso	1.000000	O
O1066	1.0	0.287679	0.392055	0.422514	Biso	1.000000	O
O1067	1.0	0.535909	0.817868	0.451483	Biso	1.000000	O
O1068	1.0	0.585067	0.535682	0.102561	Biso	1.000000	O
O1069	1.0	0.648495	0.244570	0.207056	Biso	1.000000	O
O1070	1.0	0.343190	0.475148	0.092266	Biso	1.000000	O
O1071	1.0	0.976334	0.449588	0.384476	Biso	1.000000	O
O1072	1.0	0.425423	0.371081	0.086013	Biso	1.000000	O
O1073	1.0	0.275296	0.622027	0.865001	Biso	1.000000	O
O1074	1.0	0.306693	0.260429	0.162270	Biso	1.000000	O
O1075	1.0	0.236584	0.607496	0.623039	Biso	1.000000	O
O1076	1.0	0.438067	0.540408	0.839134	Biso	1.000000	O
O1077	1.0	0.729178	0.662354	0.696531	Biso	1.000000	O
O1078	1.0	0.190022	0.809017	0.880028	Biso	1.000000	O
O1079	1.0	0.068077	0.177683	0.630914	Biso	1.000000	O
O1080	1.0	0.956848	0.274707	0.842457	Biso	1.000000	O
O1081	1.0	0.799974	0.792975	0.272752	Biso	1.000000	O
O1082	1.0	0.351877	0.094988	0.028550	Biso	1.000000	O
O1083	1.0	0.563574	0.773711	0.144638	Biso	1.000000	O
O1084	1.0	0.546028	0.343660	0.995918	Biso	1.000000	O
O1085	1.0	0.989264	0.483812	0.052173	Biso	1.000000	O
O1086	1.0	0.749767	0.896339	0.112436	Biso	1.000000	O
O1087	1.0	0.239914	0.663845	0.820302	Biso	1.000000	O
O1088	1.0	0.743867	0.370297	0.981822	Biso	1.000000	O
O1089	1.0	0.566462	0.673071	0.387433	Biso	1.000000	O
O1090	1.0	0.957076	0.968166	0.228711	Biso	1.000000	O
O1091	1.0	0.122659	0.814975	0.648078	Biso	1.000000	O
O1092	1.0	0.348131	0.767177	0.912340	Biso	1.000000	O
O1093	1.0	0.228325	0.939471	0.701312	Biso	1.000000	O
O1094	1.0	0.013512	0.054516	0.674842	Biso	1.000000	O
O1095	1.0	0.579983	0.573718	0.163194	Biso	1.000000	O
O1096	1.0	0.357535	0.889614	0.883207	Biso	1.000000	O
O1097	1.0	0.097469	0.454875	0.024419	Biso	1.000000	O
O1098	1.0	0.803266	0.978494	0.837760	Biso	1.000000	O
O1099	1.0	0.683327	0.348431	0.879609	Biso	1.000000	O
O1100	1.0	0.514202	0.017325	0.632429	Biso	1.000000	O
O1101	1.0	0.074263	0.184266	0.837071	Biso	1.000000	O
O1102	1.0	0.177886	0.436110	0.360841	Biso	1.000000	O
O1103	1.0	0.846013	0.660610	0.530462	Biso	1.000000	O
O1104	1.0	0.703822	0.991630	0.678824	Biso	1.000000	O
O1105	1.0	0.620778	0.348550	0.319251	Biso	1.000000	O
O1106	1.0	0.394035	0.223560	0.198963	Biso	1.000000	O
O1107	1.0	0.671626	0.754210	0.221992	Biso	1.000000	O
O1108	1.0	0.097216	0.188185	0.765432	Biso	1.000000	O
O1109	1.0	0.546344	0.511281	0.320284	Biso	1.000000	O
O1110	1.0	0.913236	0.230762	0.620597	Biso	1.000000	O
O1111	1.0	0.185609	0.496656	0.752270	Biso	1.000000	O
O1112	1.0	0.735917	0.742113	0.196818	Biso	1.000000	O
O1113	1.0	0.187935	0.334704	0.676619	Biso	1.000000	O
O1114	1.0	0.205173	0.866490	0.362642	Biso	1.000000	O
O1115	1.0	0.722278	0.797133	0.052192	Biso	1.000000	O
O1116	1.0	0.758761	0.025584	0.429047	Biso	1.000000	O
O1117	1.0	0.166756	0.094407	0.732006	Biso	1.000000	O
O1118	1.0	0.970600	0.610083	0.087192	Biso	1.000000	O
O1119	1.0	0.266313	0.492334	0.582545	Biso	1.000000	O
O1120	1.0	0.469926	0.718791	0.066665	Biso	1.000000	O
O1121	1.0	0.952385	0.587062	0.325727	Biso	1.000000	O
O1122	1.0	0.313409	0.796534	0.042615	Biso	1.000000	O
O1123	1.0	0.173789	0.987208	0.504842	Biso	1.000000	O
O1124	1.0	0.863490	0.672926	0.363997	Biso	1.000000	O
O1125	1.0	0.520218	0.296373	0.702099	Biso	1.000000	O
O1126	1.0	0.469972	0.629825	0.446214	Biso	1.000000	O
O1127	1.0	0.676655	0.165830	0.578429	Biso	1.000000	O
O1128	1.0	0.768146	0.686452	0.463280	Biso	1.000000	O
O1129	1.0	0.308111	0.085755	0.798000	Biso	1.000000	O
O1130	1.0	0.250087	0.018301	0.087357	Biso	1.000000	O
O1131	1.0	0.975186	0.927854	0.873296	Biso	1.000000	O
O1132	1.0	0.826565	0.988116	0.730878	Biso	1.000000	O
O1133	1.0	0.475463	0.780944	0.024050	Biso	1.000000	O
O1134	1.0	0.917410	0.140766	0.164146	Biso	1.000000	O

O1135	1.0	0.203601	0.704260	0.609108	Biso	1.000000	O
O1136	1.0	0.767061	0.523946	0.587043	Biso	1.000000	O
O1137	1.0	0.766255	0.380012	0.453053	Biso	1.000000	O
O1138	1.0	0.851949	0.320582	0.791804	Biso	1.000000	O
O1139	1.0	0.213138	0.761097	0.480902	Biso	1.000000	O
O1140	1.0	0.234629	0.893760	0.420963	Biso	1.000000	O
O1141	1.0	0.213293	0.732089	0.807289	Biso	1.000000	O
O1142	1.0	0.054434	0.446183	0.498502	Biso	1.000000	O
O1143	1.0	0.631790	0.161183	0.377427	Biso	1.000000	O
O1144	1.0	0.167551	0.600945	0.638221	Biso	1.000000	O
O1145	1.0	0.945287	0.728820	0.881002	Biso	1.000000	O
O1146	1.0	0.632007	0.400969	0.119689	Biso	1.000000	O
O1147	1.0	0.822621	0.951597	0.188091	Biso	1.000000	O
O1148	1.0	0.000353	0.860532	0.858326	Biso	1.000000	O
O1149	1.0	0.629767	0.448852	0.630617	Biso	1.000000	O
O1150	1.0	0.234164	0.939846	0.770854	Biso	1.000000	O
O1151	1.0	0.677740	0.256225	0.022520	Biso	1.000000	O
O1152	1.0	0.684781	0.146065	0.422514	Biso	1.000000	O
O1153	1.0	0.199480	0.479672	0.485244	Biso	1.000000	O
O1154	1.0	0.440010	0.569245	0.520543	Biso	1.000000	O
O1155	1.0	0.938123	0.833612	0.407470	Biso	1.000000	O
O1156	1.0	0.998432	0.237432	0.312039	Biso	1.000000	O
O1157	1.0	0.987468	0.839382	0.488242	Biso	1.000000	O
O1158	1.0	0.918026	0.415395	0.505158	Biso	1.000000	O
O1159	1.0	0.306712	0.062027	0.421889	Biso	1.000000	O
O1160	1.0	0.251029	0.000314	0.733860	Biso	1.000000	O
O1161	1.0	0.125144	0.361837	0.686489	Biso	1.000000	O
O1162	1.0	0.813203	0.119955	0.228915	Biso	1.000000	O
O1163	1.0	0.354006	0.058397	0.566796	Biso	1.000000	O
O1164	1.0	0.762628	0.898627	0.422808	Biso	1.000000	O
O1165	1.0	0.649020	0.830167	0.314203	Biso	1.000000	O
O1166	1.0	0.492210	0.392225	0.305663	Biso	1.000000	O
O1167	1.0	0.772070	0.330845	0.578737	Biso	1.000000	O
O1168	1.0	0.774469	0.836682	0.602113	Biso	1.000000	O
O1169	1.0	0.468483	0.789020	0.220897	Biso	1.000000	O
O1170	1.0	0.857986	0.590861	0.860965	Biso	1.000000	O
O1171	1.0	0.568029	0.104955	0.387820	Biso	1.000000	O
O1172	1.0	0.107661	0.461568	0.848254	Biso	1.000000	O
O1173	1.0	0.625260	0.066902	0.514693	Biso	1.000000	O
O1174	1.0	0.157310	0.760245	0.839600	Biso	1.000000	O
O1175	1.0	0.366999	0.600706	0.290647	Biso	1.000000	O
O1176	1.0	0.274359	0.069999	0.289502	Biso	1.000000	O
O1177	1.0	0.181493	0.896904	0.665484	Biso	1.000000	O
O1178	1.0	0.308586	0.545590	0.190403	Biso	1.000000	O
O1179	1.0	0.814784	0.486192	0.474943	Biso	1.000000	O
O1180	1.0	0.167515	0.478329	0.161856	Biso	1.000000	O
O1181	1.0	0.495845	0.982517	0.573160	Biso	1.000000	O
O1182	1.0	0.197059	0.157138	0.899512	Biso	1.000000	O
O1183	1.0	0.914888	0.202270	0.042498	Biso	1.000000	O
O1184	1.0	0.672648	0.009763	0.464654	Biso	1.000000	O
O1185	1.0	0.711261	0.088613	0.996124	Biso	1.000000	O
O1186	1.0	0.340728	0.592253	0.852703	Biso	1.000000	O
O1187	1.0	0.656509	0.382158	0.244429	Biso	1.000000	O
O1188	1.0	0.871391	0.315016	0.198136	Biso	1.000000	O
O1189	1.0	0.895918	0.272766	0.565556	Biso	1.000000	O
O1190	1.0	0.415203	0.280556	0.666278	Biso	1.000000	O
O1191	1.0	0.007842	0.414588	0.801262	Biso	1.000000	O
O1192	1.0	0.056472	0.330657	0.981404	Biso	1.000000	O
O1193	1.0	0.234953	0.865019	0.051354	Biso	1.000000	O
O1194	1.0	0.435297	0.964091	0.436971	Biso	1.000000	O
O1195	1.0	0.757310	0.004110	0.293223	Biso	1.000000	O
O1196	1.0	0.519828	0.868254	0.155113	Biso	1.000000	O
O1197	1.0	0.873331	0.019717	0.448771	Biso	1.000000	O
O1198	1.0	0.889515	0.099358	0.902142	Biso	1.000000	O
O1199	1.0	0.013866	0.550185	0.343243	Biso	1.000000	O
O1200	1.0	0.168630	0.774725	0.375805	Biso	1.000000	O
O1201	1.0	0.201940	0.908359	0.598270	Biso	1.000000	O
O1202	1.0	0.325380	0.124358	0.571206	Biso	1.000000	O
O1203	1.0	0.768215	0.849898	0.856805	Biso	1.000000	O
O1204	1.0	0.399652	0.080418	0.445309	Biso	1.000000	O
O1205	1.0	0.591272	0.042576	0.222355	Biso	1.000000	O
O1206	1.0	0.973095	0.179402	0.788070	Biso	1.000000	O
O1207	1.0	0.594493	0.415599	0.246040	Biso	1.000000	O
O1208	1.0	0.233088	0.245044	0.114038	Biso	1.000000	O
O1209	1.0	0.353174	0.384853	0.393506	Biso	1.000000	O
O1210	1.0	0.730649	0.391518	0.916333	Biso	1.000000	O
O1211	1.0	0.186053	0.982980	0.084297	Biso	1.000000	O
O1212	1.0	0.034885	0.062122	0.235674	Biso	1.000000	O
O1213	1.0	0.431573	0.380835	0.504452	Biso	1.000000	O
O1214	1.0	0.327619	0.820805	0.495392	Biso	1.000000	O
O1215	1.0	0.614400	0.514018	0.599903	Biso	1.000000	O
O1216	1.0	0.155927	0.741145	0.978645	Biso	1.000000	O

O1217	1.0	0.498885	0.556676	0.961171	Biso	1.000000	O
O1218	1.0	0.402760	0.359554	0.151332	Biso	1.000000	O
O1219	1.0	0.079226	0.113275	0.489532	Biso	1.000000	O
O1220	1.0	0.164434	0.869631	0.206860	Biso	1.000000	O
O1221	1.0	0.535808	0.428361	0.529930	Biso	1.000000	O
O1222	1.0	0.872768	0.418419	0.589493	Biso	1.000000	O
O1223	1.0	0.614257	0.736207	0.704713	Biso	1.000000	O
O1224	1.0	0.887423	0.606082	0.550676	Biso	1.000000	O
O1225	1.0	0.160857	0.037928	0.556700	Biso	1.000000	O
O1226	1.0	0.413258	0.616617	0.566725	Biso	1.000000	O
O1227	1.0	0.363710	0.316285	0.633250	Biso	1.000000	O
O1228	1.0	0.431804	0.366375	0.838771	Biso	1.000000	O
O1229	1.0	0.460431	0.512130	0.480324	Biso	1.000000	O
O1230	1.0	0.065041	0.652381	0.297287	Biso	1.000000	O
O1231	1.0	0.032579	0.557804	0.467104	Biso	1.000000	O
O1232	1.0	0.136446	0.805388	0.579471	Biso	1.000000	O
O1233	1.0	0.130534	0.671873	0.121998	Biso	1.000000	O
O1234	1.0	0.956853	0.174920	0.494817	Biso	1.000000	O
O1235	1.0	0.896066	0.391606	0.064396	Biso	1.000000	O
O1236	1.0	0.132199	0.316237	0.517656	Biso	1.000000	O
O1237	1.0	0.349769	0.136981	0.823112	Biso	1.000000	O
O1238	1.0	0.705028	0.659881	0.895980	Biso	1.000000	O
O1239	1.0	0.053731	0.892252	0.769347	Biso	1.000000	O
O1240	1.0	0.101788	0.551982	0.458889	Biso	1.000000	O
O1241	1.0	0.516926	0.839927	0.223999	Biso	1.000000	O
O1242	1.0	0.472891	0.567504	0.893728	Biso	1.000000	O
O1243	1.0	0.557759	0.149857	0.684409	Biso	1.000000	O
O1244	1.0	0.982082	0.712072	0.279709	Biso	1.000000	O
O1245	1.0	0.748311	0.309615	0.352235	Biso	1.000000	O
O1246	1.0	0.611454	0.770934	0.297200	Biso	1.000000	O
O1247	1.0	0.476021	0.440029	0.255531	Biso	1.000000	O
O1248	1.0	0.850951	0.154464	0.927026	Biso	1.000000	O
O1249	1.0	0.317361	0.560323	0.259203	Biso	1.000000	O
O1250	1.0	0.807117	0.391348	0.702072	Biso	1.000000	O
O1251	1.0	0.868366	0.812538	0.285801	Biso	1.000000	O
O1252	1.0	0.770784	0.961178	0.235298	Biso	1.000000	O
O1253	1.0	0.752642	0.708857	0.136973	Biso	1.000000	O
O1254	1.0	0.159594	0.429894	0.454628	Biso	1.000000	O
O1255	1.0	0.712421	0.582242	0.120533	Biso	1.000000	O
O1256	1.0	0.939420	0.969870	0.969238	Biso	1.000000	O
O1257	1.0	0.915407	0.672313	0.535951	Biso	1.000000	O
O1258	1.0	0.457620	0.699054	0.461792	Biso	1.000000	O
O1259	1.0	0.025810	0.640423	0.052599	Biso	1.000000	O
O1260	1.0	0.582723	0.876656	0.452489	Biso	1.000000	O
O1261	1.0	0.853573	0.592678	0.137446	Biso	1.000000	O
O1262	1.0	0.043592	0.949044	0.725615	Biso	1.000000	O
O1263	1.0	0.866722	0.389650	0.207895	Biso	1.000000	O
O1264	1.0	0.674863	0.177185	0.904053	Biso	1.000000	O
O1265	1.0	0.553148	0.480061	0.062651	Biso	1.000000	O
O1266	1.0	0.841028	0.460842	0.220866	Biso	1.000000	O
O1267	1.0	0.608604	0.498792	0.439410	Biso	1.000000	O
O1268	1.0	0.904868	0.425615	0.652240	Biso	1.000000	O
O1269	1.0	0.105365	0.274640	0.094789	Biso	1.000000	O
O1270	1.0	0.014492	0.730558	0.865061	Biso	1.000000	O
O1271	1.0	0.089333	0.870985	0.894974	Biso	1.000000	O
O1272	1.0	0.251100	0.956637	0.613635	Biso	1.000000	O
O1273	1.0	0.667102	0.319665	0.503207	Biso	1.000000	O
O1274	1.0	0.934676	0.837326	0.326897	Biso	1.000000	O
O1275	1.0	0.102366	0.778362	0.348637	Biso	1.000000	O
O1276	1.0	0.329450	0.298023	0.547397	Biso	1.000000	O
O1277	1.0	0.642691	0.432038	0.294409	Biso	1.000000	O
O1278	1.0	0.489646	0.127682	0.700499	Biso	1.000000	O
O1279	1.0	0.227558	0.207127	0.598605	Biso	1.000000	O
O1280	1.0	0.528138	0.332213	0.625179	Biso	1.000000	O
O1281	1.0	0.827667	0.345299	0.441964	Biso	1.000000	O
O1282	1.0	0.224708	0.845928	0.175111	Biso	1.000000	O
O1283	1.0	0.607642	0.831865	0.257829	Biso	1.000000	O
O1284	1.0	0.232865	0.218869	0.528008	Biso	1.000000	O
O1285	1.0	0.818059	0.842722	0.656953	Biso	1.000000	O
O1286	1.0	0.216305	0.520490	0.539999	Biso	1.000000	O
O1287	1.0	0.511383	0.674173	0.977122	Biso	1.000000	O

O1288	1.0	0.850635	0.265488	0.151435	Biso	1.000000	O
O1289	1.0	0.853345	0.803511	0.938071	Biso	1.000000	O
O1290	1.0	0.735216	0.829000	0.437791	Biso	1.000000	O
O1291	1.0	0.348807	0.537153	0.319802	Biso	1.000000	O
O1292	1.0	0.384562	0.216831	0.054715	Biso	1.000000	O
O1293	1.0	0.763340	0.499871	0.520916	Biso	1.000000	O
O1294	1.0	0.294579	0.209182	0.266051	Biso	1.000000	O
O1295	1.0	0.627832	0.411959	0.691000	Biso	1.000000	O
O1296	1.0	0.432032	0.546124	0.208101	Biso	1.000000	O
O1297	1.0	0.642408	0.257267	0.085890	Biso	1.000000	O
O1298	1.0	0.798136	0.705285	0.191850	Biso	1.000000	O
O1299	1.0	0.618321	0.495878	0.508685	Biso	1.000000	O
O1300	1.0	0.457916	0.584739	0.012267	Biso	1.000000	O
O1301	1.0	0.247331	0.044146	0.915093	Biso	1.000000	O
O1302	1.0	0.295533	0.817303	0.913446	Biso	1.000000	O
O1303	1.0	0.458004	0.403510	0.140001	Biso	1.000000	O
O1304	1.0	0.510190	0.831900	0.346173	Biso	1.000000	O
O1305	1.0	0.815539	0.679662	0.877763	Biso	1.000000	O
O1306	1.0	0.739481	0.738044	0.810195	Biso	1.000000	O
O1307	1.0	0.761490	0.073739	0.762952	Biso	1.000000	O
O1308	1.0	0.240096	0.606675	0.291459	Biso	1.000000	O
O1309	1.0	0.695693	0.592900	0.424157	Biso	1.000000	O
O1310	1.0	0.262403	0.313414	0.529197	Biso	1.000000	O
O1311	1.0	0.719571	0.705467	0.418651	Biso	1.000000	O
O1312	1.0	0.670634	0.569830	0.064521	Biso	1.000000	O
O1313	1.0	0.859715	0.860416	0.981838	Biso	1.000000	O
O1314	1.0	0.423505	0.515569	0.903462	Biso	1.000000	O
O1315	1.0	0.580390	0.958616	0.536286	Biso	1.000000	O
O1316	1.0	0.842784	0.753143	0.590188	Biso	1.000000	O
O1317	1.0	0.392405	0.424428	0.120772	Biso	1.000000	O
O1318	1.0	0.756346	0.740744	0.022420	Biso	1.000000	O
O1319	1.0	0.408545	0.465925	0.063218	Biso	1.000000	O
O1320	1.0	0.972333	0.427290	0.296706	Biso	1.000000	O
O1321	1.0	0.629036	0.575044	0.406513	Biso	1.000000	O
O1322	1.0	0.786432	0.116696	0.553099	Biso	1.000000	O
O1323	1.0	0.233892	0.492635	0.181021	Biso	1.000000	O
O1324	1.0	0.866340	0.246170	0.797531	Biso	1.000000	O
O1325	1.0	0.531100	0.440259	0.882642	Biso	1.000000	O
O1326	1.0	0.682706	0.834421	0.956006	Biso	1.000000	O
O1327	1.0	0.598027	0.425683	0.862368	Biso	1.000000	O
O1328	1.0	0.383741	0.538232	0.258369	Biso	1.000000	O
O1329	1.0	0.428540	0.528558	0.677415	Biso	1.000000	O
O1330	1.0	0.990791	0.934513	0.766730	Biso	1.000000	O
O1331	1.0	0.420326	0.760736	0.449752	Biso	1.000000	O
O1332	1.0	0.693852	0.169120	0.836411	Biso	1.000000	O
O1333	1.0	0.045402	0.700361	0.247627	Biso	1.000000	O
O1334	1.0	0.950398	0.733222	0.545960	Biso	1.000000	O
O1335	1.0	0.280625	0.508645	0.386994	Biso	1.000000	O
O1336	1.0	0.478923	0.352109	0.015925	Biso	1.000000	O
O1337	1.0	0.587675	0.530642	0.666054	Biso	1.000000	O
O1338	1.0	0.132804	0.027093	0.722815	Biso	1.000000	O
O1339	1.0	0.359929	0.755315	0.498009	Biso	1.000000	O
O1340	1.0	0.777528	0.960224	0.458136	Biso	1.000000	O
O1341	1.0	0.448375	0.031009	0.045221	Biso	1.000000	O
O1342	1.0	0.110559	0.407344	0.279210	Biso	1.000000	O
O1343	1.0	0.821945	0.653210	0.113438	Biso	1.000000	O
O1344	1.0	0.835295	0.541163	0.905792	Biso	1.000000	O
O1345	1.0	0.564289	0.528569	0.951653	Biso	1.000000	O
O1346	1.0	0.875752	0.814253	0.574938	Biso	1.000000	O
O1347	1.0	0.799507	0.141579	0.114496	Biso	1.000000	O
O1348	1.0	0.796553	0.270095	0.494239	Biso	1.000000	O
O1349	1.0	0.534662	0.982764	0.864628	Biso	1.000000	O
O1350	1.0	0.755664	0.089727	0.393740	Biso	1.000000	O
O1351	1.0	0.590692	0.766938	0.944566	Biso	1.000000	O
O1352	1.0	0.090316	0.426614	0.441736	Biso	1.000000	O
O1353	1.0	0.501003	0.566238	0.318989	Biso	1.000000	O
O1354	1.0	0.400724	0.324032	0.987209	Biso	1.000000	O
O1355	1.0	0.929384	0.312449	0.514202	Biso	1.000000	O
O1356	1.0	0.280402	0.807552	0.233441	Biso	1.000000	O

O1357	1.0	0.945278	0.869154	0.902287	Biso	1.000000	O
O1358	1.0	0.692283	0.136224	0.268061	Biso	1.000000	O
O1359	1.0	0.698700	0.524281	0.943661	Biso	1.000000	O
O1360	1.0	0.019153	0.133217	0.850746	Biso	1.000000	O
O1361	1.0	0.057260	0.839825	0.816254	Biso	1.000000	O
O1362	1.0	0.187668	0.058027	0.091287	Biso	1.000000	O
O1363	1.0	0.237086	0.767321	0.276909	Biso	1.000000	O
O1364	1.0	0.760116	0.961550	0.890637	Biso	1.000000	O
O1365	1.0	0.814825	0.654550	0.628692	Biso	1.000000	O
O1366	1.0	0.808037	0.615107	0.816379	Biso	1.000000	O
O1367	1.0	0.430395	0.028821	0.406333	Biso	1.000000	O
O1368	1.0	0.954990	0.392137	0.693666	Biso	1.000000	O
O1369	1.0	0.633425	0.377658	0.053572	Biso	1.000000	O
O1370	1.0	0.303357	0.769318	0.538584	Biso	1.000000	O
O1371	1.0	0.006753	0.974093	0.100513	Biso	1.000000	O
O1372	1.0	0.530237	0.751382	0.087275	Biso	1.000000	O
O1373	1.0	0.371457	0.593638	0.435455	Biso	1.000000	O
O1374	1.0	0.953234	0.420850	0.145490	Biso	1.000000	O
O1375	1.0	0.213756	0.117873	0.785780	Biso	1.000000	O
O1376	1.0	0.805949	0.623969	0.475805	Biso	1.000000	O
O1377	1.0	0.703954	0.273869	0.640263	Biso	1.000000	O
O1378	1.0	0.696738	0.439918	0.338378	Biso	1.000000	O
O1379	1.0	0.997509	0.468196	0.754194	Biso	1.000000	O
O1380	1.0	0.708711	0.876591	0.167330	Biso	1.000000	O
O1381	1.0	0.128322	0.275274	0.334518	Biso	1.000000	O
O1382	1.0	0.552079	0.892763	0.342968	Biso	1.000000	O
O1383	1.0	0.424200	0.758291	0.173201	Biso	1.000000	O
O1384	1.0	0.233562	0.042408	0.342502	Biso	1.000000	O
O1385	1.0	0.534464	0.162935	0.056169	Biso	1.000000	O
O1386	1.0	0.642958	0.377268	0.631184	Biso	1.000000	O
O1387	1.0	0.950104	0.444811	0.450289	Biso	1.000000	O
O1388	1.0	0.005681	0.837213	0.324504	Biso	1.000000	O
O1389	1.0	0.839176	0.752302	0.522609	Biso	1.000000	O
O1390	1.0	0.208948	0.370155	0.618231	Biso	1.000000	O
O1391	1.0	0.158916	0.879459	0.542902	Biso	1.000000	O
O1392	1.0	0.136632	0.256711	0.017800	Biso	1.000000	O
O1393	1.0	0.422997	0.789091	0.823564	Biso	1.000000	O
O1394	1.0	0.306226	0.059849	0.351728	Biso	1.000000	O
O1395	1.0	0.839071	0.846748	0.521246	Biso	1.000000	O
O1396	1.0	0.306330	0.595734	0.618577	Biso	1.000000	O
O1397	1.0	0.549686	0.754298	0.485004	Biso	1.000000	O
O1398	1.0	0.562338	0.315439	0.429876	Biso	1.000000	O
O1399	1.0	0.169272	0.861330	0.473894	Biso	1.000000	O
O1400	1.0	0.580942	0.672844	0.524061	Biso	1.000000	O
O1401	1.0	0.449862	0.551926	0.742789	Biso	1.000000	O
O1402	1.0	0.407456	0.972872	0.034694	Biso	1.000000	O
O1403	1.0	0.699780	0.204049	0.243276	Biso	1.000000	O
O1404	1.0	0.005252	0.906175	0.128814	Biso	1.000000	O
O1405	1.0	0.070545	0.235734	0.321661	Biso	1.000000	O
O1406	1.0	0.950934	0.027233	0.694252	Biso	1.000000	O
O1407	1.0	0.737379	0.943606	0.003102	Biso	1.000000	O
O1408	1.0	0.100360	0.152751	0.293964	Biso	1.000000	O
O1409	1.0	0.700263	0.125535	0.054757	Biso	1.000000	O
O1410	1.0	0.353064	0.916844	0.273303	Biso	1.000000	O
O1411	1.0	0.194061	0.480498	0.416285	Biso	1.000000	O
O1412	1.0	0.626917	0.203551	0.544856	Biso	1.000000	O
O1413	1.0	0.547759	0.230439	0.985171	Biso	1.000000	O
O1414	1.0	0.968819	0.850622	0.266594	Biso	1.000000	O
O1415	1.0	0.727455	0.866115	0.801854	Biso	1.000000	O
O1416	1.0	0.646736	0.041017	0.948854	Biso	1.000000	O
O1417	1.0	0.516959	0.682828	0.107066	Biso	1.000000	O
O1418	1.0	0.766890	0.995773	0.041795	Biso	1.000000	O
O1419	1.0	0.295033	0.129832	0.053580	Biso	1.000000	O
O1420	1.0	0.039438	0.901055	0.317550	Biso	1.000000	O
O1421	1.0	0.255583	0.084648	0.664231	Biso	1.000000	O
O1422	1.0	0.222222	0.441955	0.132088	Biso	1.000000	O
O1423	1.0	0.382358	0.251308	0.541959	Biso	1.000000	O
O1424	1.0	0.367313	0.187329	0.255522	Biso	1.000000	O
O1425	1.0	0.075333	0.838278	0.313006	Biso	1.000000	O
O1426	1.0	0.864823	0.601763	0.237576	Biso	1.000000	O
O1427	1.0	0.202492	0.488904	0.030344	Biso	1.000000	O
O1428	1.0	0.726711	0.297137	0.990233	Biso	1.000000	O

O1429	1.0	0.497662	0.026044	0.330628	Biso	1.000000	O
O1430	1.0	0.292191	0.942669	0.670862	Biso	1.000000	O
O1431	1.0	0.905693	0.452379	0.103867	Biso	1.000000	O
O1432	1.0	0.743306	0.183089	0.134698	Biso	1.000000	O
O1433	1.0	0.479461	0.397916	0.408830	Biso	1.000000	O
O1434	1.0	0.595108	0.831232	0.912380	Biso	1.000000	O
O1435	1.0	0.464322	0.660139	0.593838	Biso	1.000000	O
O1436	1.0	0.353619	0.931497	0.055324	Biso	1.000000	O
O1437	1.0	0.850030	0.742917	0.282761	Biso	1.000000	O
O1438	1.0	0.812843	0.917841	0.086970	Biso	1.000000	O
O1439	1.0	0.650630	0.754969	0.463430	Biso	1.000000	O
O1440	1.0	0.520006	0.239330	0.226964	Biso	1.000000	O
O1441	1.0	0.294942	0.841912	0.174625	Biso	1.000000	O
O1442	1.0	0.713281	0.641689	0.377669	Biso	1.000000	O
O1443	1.0	0.157055	0.682502	0.936043	Biso	1.000000	O
O1444	1.0	0.185037	0.534720	0.861758	Biso	1.000000	O
O1445	1.0	0.328312	0.565434	0.484459	Biso	1.000000	O
O1446	1.0	0.274507	0.952426	0.420442	Biso	1.000000	O
O1447	1.0	0.130615	0.990815	0.784551	Biso	1.000000	O
O1448	1.0	0.816949	0.668709	0.417345	Biso	1.000000	O
O1449	1.0	0.116384	0.596381	0.187812	Biso	1.000000	O
O1450	1.0	0.967025	0.885161	0.625841	Biso	1.000000	O
O1451	1.0	0.179247	0.728732	0.203049	Biso	1.000000	O
O1452	1.0	0.707529	0.810516	0.199018	Biso	1.000000	O
O1453	1.0	0.631218	0.068133	0.396025	Biso	1.000000	O
O1454	1.0	0.378657	0.851559	0.827457	Biso	1.000000	O
O1455	1.0	0.721759	0.420580	0.274985	Biso	1.000000	O
O1456	1.0	0.498827	0.120787	0.369421	Biso	1.000000	O
O1457	1.0	0.305300	0.018400	0.824377	Biso	1.000000	O
O1458	1.0	0.683360	0.632375	0.219274	Biso	1.000000	O
O1459	1.0	0.559691	0.095692	0.048170	Biso	1.000000	O
O1460	1.0	0.450527	0.457729	0.382554	Biso	1.000000	O
O1461	1.0	0.542010	0.085396	0.326016	Biso	1.000000	O
O1462	1.0	0.282812	0.520859	0.518209	Biso	1.000000	O
O1463	1.0	0.694696	0.370212	0.456708	Biso	1.000000	O
O1464	1.0	0.426058	0.263174	0.330138	Biso	1.000000	O
O1465	1.0	0.037603	0.681062	0.966397	Biso	1.000000	O
O1466	1.0	0.619855	0.274468	0.149554	Biso	1.000000	O
O1467	1.0	0.832182	0.308911	0.893276	Biso	1.000000	O
O1468	1.0	0.536618	0.577429	0.532898	Biso	1.000000	O
O1469	1.0	0.045166	0.262400	0.004019	Biso	1.000000	O
O1470	1.0	0.851358	0.080570	0.479130	Biso	1.000000	O
O1471	1.0	0.481237	0.545823	0.134080	Biso	1.000000	O
O1472	1.0	0.494639	0.199417	0.559703	Biso	1.000000	O
O1473	1.0	0.270809	0.885443	0.957045	Biso	1.000000	O
O1474	1.0	0.864378	0.344387	0.288911	Biso	1.000000	O
O1475	1.0	0.300433	0.944798	0.234608	Biso	1.000000	O
O1476	1.0	0.120543	0.804451	0.882449	Biso	1.000000	O
O1477	1.0	0.424363	0.352955	0.406731	Biso	1.000000	O
O1478	1.0	0.888547	0.022178	0.734438	Biso	1.000000	O
O1479	1.0	0.256485	0.792663	0.140333	Biso	1.000000	O
O1480	1.0	0.261974	0.917679	0.481461	Biso	1.000000	O
O1481	1.0	0.531023	0.494577	0.129464	Biso	1.000000	O
O1482	1.0	0.339686	0.251240	0.422042	Biso	1.000000	O
O1483	1.0	0.876258	0.760208	0.809977	Biso	1.000000	O
O1484	1.0	0.273432	0.062067	0.485865	Biso	1.000000	O
O1485	1.0	0.599555	0.989024	0.471002	Biso	1.000000	O
O1486	1.0	0.068919	0.947536	0.469298	Biso	1.000000	O
O1487	1.0	0.035303	0.691220	0.622826	Biso	1.000000	O
O1488	1.0	0.070678	0.145112	0.230505	Biso	1.000000	O
O1489	1.0	0.532631	0.206534	0.290767	Biso	1.000000	O
O1490	1.0	0.332368	0.540865	0.120922	Biso	1.000000	O
O1491	1.0	0.969539	0.137246	0.627139	Biso	1.000000	O
O1492	1.0	0.697210	0.189376	0.368282	Biso	1.000000	O
O1493	1.0	0.050936	0.129646	0.552093	Biso	1.000000	O
O1494	1.0	0.882504	0.963293	0.556370	Biso	1.000000	O
O1495	1.0	0.043824	0.101800	0.792668	Biso	1.000000	O
O1496	1.0	0.878630	0.868699	0.422884	Biso	1.000000	O
O1497	1.0	0.752037	0.254594	0.125067	Biso	1.000000	O
O1498	1.0	0.666468	0.823801	0.143754	Biso	1.000000	O
O1499	1.0	0.087586	0.886385	0.437028	Biso	1.000000	O
O1500	1.0	0.886563	0.556816	0.187263	Biso	1.000000	O
O1501	1.0	0.985770	0.165414	0.936849	Biso	1.000000	O
O1502	1.0	0.282474	0.748227	0.934282	Biso	1.000000	O

O1503	1.0	0.074079	0.650586	0.159922	Biso	1.000000	O
O1504	1.0	0.672343	0.801253	0.413072	Biso	1.000000	O
O1505	1.0	0.894865	0.548294	0.507560	Biso	1.000000	O
O1506	1.0	0.071087	0.760600	0.207057	Biso	1.000000	O
O1507	1.0	0.446221	0.106228	0.238341	Biso	1.000000	O
O1508	1.0	0.299023	0.683129	0.378422	Biso	1.000000	O
O1509	1.0	0.480632	0.873237	0.095689	Biso	1.000000	O
O1510	1.0	0.119283	0.566190	0.938507	Biso	1.000000	O
O1511	1.0	0.337161	0.600462	0.021480	Biso	1.000000	O
O1512	1.0	0.838370	0.411703	0.300708	Biso	1.000000	O
O1513	1.0	0.649757	0.810249	0.048097	Biso	1.000000	O
O1514	1.0	0.591910	0.151566	0.508083	Biso	1.000000	O
O1515	1.0	0.380740	0.249016	0.612011	Biso	1.000000	O
O1516	1.0	0.818876	0.044382	0.027428	Biso	1.000000	O
O1517	1.0	0.098489	0.504159	0.077279	Biso	1.000000	O
O1518	1.0	0.149520	0.712955	0.786631	Biso	1.000000	O
O1519	1.0	0.342934	0.560351	0.916049	Biso	1.000000	O
O1520	1.0	0.267366	0.601541	0.797245	Biso	1.000000	O
O1521	1.0	0.834015	0.315550	0.609576	Biso	1.000000	O
O1522	1.0	0.758734	0.552374	0.651357	Biso	1.000000	O
O1523	1.0	0.671274	0.765720	0.529055	Biso	1.000000	O
O1524	1.0	0.085412	0.609859	0.823518	Biso	1.000000	O
O1525	1.0	0.624815	0.809875	0.791591	Biso	1.000000	O
O1526	1.0	0.208562	0.975917	0.356460	Biso	1.000000	O
O1527	1.0	0.866813	0.797530	0.868605	Biso	1.000000	O
O1528	1.0	0.355455	0.792609	0.392571	Biso	1.000000	O
O1529	1.0	0.986067	0.546931	0.518125	Biso	1.000000	O
O1530	1.0	0.224575	0.194206	0.666569	Biso	1.000000	O
O1531	1.0	0.534491	0.770936	0.986245	Biso	1.000000	O
O1532	1.0	0.918828	0.690640	0.405371	Biso	1.000000	O
O1533	1.0	0.661650	0.217518	0.301814	Biso	1.000000	O
O1534	1.0	0.007802	0.581481	0.670557	Biso	1.000000	O
O1535	1.0	0.342847	0.046631	0.479318	Biso	1.000000	O
O1536	1.0	0.607565	0.807753	0.440446	Biso	1.000000	O
O1537	1.0	0.729593	0.308709	0.696168	Biso	1.000000	O
O1538	1.0	0.225175	0.623918	0.357924	Biso	1.000000	O
O1539	1.0	0.921082	0.572727	0.127607	Biso	1.000000	O
O1540	1.0	0.082446	0.243846	0.722701	Biso	1.000000	O
O1541	1.0	0.985060	0.644736	0.643981	Biso	1.000000	O
O1542	1.0	0.608428	0.551883	0.902652	Biso	1.000000	O
O1543	1.0	0.183385	0.889588	0.786519	Biso	1.000000	O
O1544	1.0	0.045356	0.074201	0.406870	Biso	1.000000	O
O1545	1.0	0.752161	0.241234	0.680160	Biso	1.000000	O
O1546	1.0	0.518421	0.079068	0.937326	Biso	1.000000	O
O1547	1.0	0.254646	0.450127	0.954818	Biso	1.000000	O
O1548	1.0	0.979771	0.450795	0.989538	Biso	1.000000	O
O1549	1.0	0.457381	0.083247	0.629867	Biso	1.000000	O
O1550	1.0	0.619448	0.273477	0.437186	Biso	1.000000	O
O1551	1.0	0.761485	0.026012	0.359712	Biso	1.000000	O
O1552	1.0	0.033058	0.391004	0.298462	Biso	1.000000	O
O1553	1.0	0.110943	0.277581	0.775779	Biso	1.000000	O
O1554	1.0	0.952572	0.321780	0.707554	Biso	1.000000	O
O1555	1.0	0.801920	0.488654	0.275623	Biso	1.000000	O
O1556	1.0	0.383982	0.607860	0.502678	Biso	1.000000	O
O1557	1.0	0.796303	0.195736	0.756170	Biso	1.000000	O
O1558	1.0	0.588422	0.011167	0.123255	Biso	1.000000	O
O1559	1.0	0.606029	0.539720	0.346593	Biso	1.000000	O
O1560	1.0	0.220974	0.499078	0.351837	Biso	1.000000	O
O1561	1.0	0.863905	0.728011	0.215602	Biso	1.000000	O
O1562	1.0	0.370239	0.353651	0.280622	Biso	1.000000	O
O1563	1.0	0.004296	0.845224	0.961310	Biso	1.000000	O
O1564	1.0	0.407121	0.500362	0.345560	Biso	1.000000	O
O1565	1.0	0.539125	0.395065	0.590447	Biso	1.000000	O
O1566	1.0	0.421101	0.142081	0.708175	Biso	1.000000	O
O1567	1.0	0.384458	0.081559	0.691641	Biso	1.000000	O
O1568	1.0	0.530889	0.774847	0.252856	Biso	1.000000	O
O1569	1.0	0.657924	0.753126	0.759937	Biso	1.000000	O
O1570	1.0	0.570623	0.714275	0.611959	Biso	1.000000	O
O1571	1.0	0.434789	0.178322	0.235694	Biso	1.000000	O
O1572	1.0	0.687966	0.819521	0.761251	Biso	1.000000	O
O1573	1.0	0.698617	0.725120	0.865064	Biso	1.000000	O
O1574	1.0	0.510042	0.265700	0.535335	Biso	1.000000	O
O1575	1.0	0.873785	0.482739	0.621823	Biso	1.000000	O
O1576	1.0	0.145898	0.097415	0.599963	Biso	1.000000	O
O1577	1.0	0.973431	0.674849	0.843353	Biso	1.000000	O
O1578	1.0	0.714600	0.490397	0.388938	Biso	1.000000	O
O1579	1.0	0.337147	0.859922	0.414560	Biso	1.000000	O
O1580	1.0	0.760320	0.625369	0.425128	Biso	1.000000	O
O1581	1.0	0.517838	0.611977	0.592810	Biso	1.000000	O
O1582	1.0	0.815674	0.272646	0.212042	Biso	1.000000	O

O1583	1.0	0.951703	0.594729	0.000990	Biso	1.000000	O
O1584	1.0	0.661755	0.544148	0.999663	Biso	1.000000	O
O1585	1.0	0.287577	0.185014	0.565853	Biso	1.000000	O
O1586	1.0	0.881878	0.461259	0.464387	Biso	1.000000	O
O1587	1.0	0.816504	0.264226	0.089183	Biso	1.000000	O
O1588	1.0	0.862273	0.574558	0.792954	Biso	1.000000	O
O1589	1.0	0.723605	0.707665	0.347779	Biso	1.000000	O
O1590	1.0	0.547399	0.103063	0.226266	Biso	1.000000	O
O1591	1.0	0.342998	0.686614	0.658237	Biso	1.000000	O
O1592	1.0	0.933723	0.018502	0.183648	Biso	1.000000	O
O1593	1.0	0.173281	0.017784	0.311123	Biso	1.000000	O
O1594	1.0	0.851789	0.100889	0.577413	Biso	1.000000	O
O1595	1.0	0.179780	0.344191	0.560573	Biso	1.000000	O
O1596	1.0	0.430647	0.097444	0.384492	Biso	1.000000	O
O1597	1.0	0.579782	0.578167	0.588790	Biso	1.000000	O
O1598	1.0	0.944174	0.511820	0.005071	Biso	1.000000	O
O1599	1.0	0.305385	0.217850	0.960469	Biso	1.000000	O
O1600	1.0	0.590640	0.769906	0.874738	Biso	1.000000	O
O1601	1.0	0.014826	0.381855	0.504371	Biso	1.000000	O
O1602	1.0	0.446177	0.010677	0.615311	Biso	1.000000	O
O1603	1.0	0.061882	0.602214	0.349725	Biso	1.000000	O
O1604	1.0	0.051280	0.991721	0.940164	Biso	1.000000	O
O1605	1.0	0.930186	0.948371	0.800240	Biso	1.000000	O
O1606	1.0	0.457232	0.855730	0.523770	Biso	1.000000	O
O1607	1.0	0.345787	0.476818	0.545895	Biso	1.000000	O
O1608	1.0	0.258255	0.562032	0.343949	Biso	1.000000	O
O1609	1.0	0.818002	0.121025	0.381616	Biso	1.000000	O
O1610	1.0	0.761543	0.457721	0.155886	Biso	1.000000	O
O1611	1.0	0.098127	0.893624	0.506491	Biso	1.000000	O
O1612	1.0	0.457488	0.151059	0.602372	Biso	1.000000	O
O1613	1.0	0.192593	0.548969	0.266535	Biso	1.000000	O
O1614	1.0	0.127420	0.410047	0.951449	Biso	1.000000	O
O1615	1.0	0.617975	0.321241	0.384454	Biso	1.000000	O
H1	1.0	0.000287	0.951707	0.418804	Biso	1.000000	H
H2	1.0	0.312260	0.922543	0.664135	Biso	1.000000	H
H3	1.0	0.342893	0.990775	0.621251	Biso	1.000000	H
H4	1.0	0.400432	0.098576	0.782983	Biso	1.000000	H
H5	1.0	0.086230	0.620508	0.537161	Biso	1.000000	H
H6	1.0	0.033451	0.709363	0.537505	Biso	1.000000	H
H7	1.0	0.152943	0.278608	0.348937	Biso	1.000000	H
H8	1.0	0.104268	0.228051	0.408026	Biso	1.000000	H
H9	1.0	0.119407	0.181521	0.324752	Biso	1.000000	H
H10	1.0	0.202461	0.696595	0.036384	Biso	1.000000	H
H11	1.0	0.289820	0.759024	0.030696	Biso	1.000000	H
H12	1.0	0.123290	0.889268	0.520491	Biso	1.000000	H
H13	1.0	0.216212	0.242883	0.525730	Biso	1.000000	H
H14	1.0	0.049360	0.693719	0.004579	Biso	1.000000	H
H15	1.0	0.003530	0.627252	0.066234	Biso	1.000000	H
H16	1.0	0.123258	0.837553	0.748748	Biso	1.000000	H
H17	1.0	0.014261	0.970512	0.073120	Biso	1.000000	H
H18	1.0	0.013918	0.900195	0.155353	Biso	1.000000	H
H19	1.0	0.094547	0.718708	0.739148	Biso	1.000000	H
H20	1.0	0.832017	0.140548	0.219432	Biso	1.000000	H
H21	1.0	0.735895	0.072449	0.206406	Biso	1.000000	H
H22	1.0	0.931436	0.115294	0.970540	Biso	1.000000	H
H23	1.0	0.863919	0.085480	0.897236	Biso	1.000000	H
H24	1.0	0.831742	0.145570	0.907349	Biso	1.000000	H
H25	1.0	0.928895	0.160412	0.883904	Biso	1.000000	H
H26	1.0	0.193134	0.909290	0.095853	Biso	1.000000	H
H27	1.0	0.203727	0.683337	0.565228	Biso	1.000000	H
H28	1.0	0.548999	0.301792	0.901581	Biso	1.000000	H
H29	1.0	0.362806	0.381556	0.287253	Biso	1.000000	H
H30	1.0	0.932756	0.860833	0.545227	Biso	1.000000	H
H31	1.0	0.268763	0.330388	0.633975	Biso	1.000000	H
H32	1.0	0.443365	0.391206	0.850961	Biso	1.000000	H
H33	1.0	0.388289	0.281820	0.841317	Biso	1.000000	H
H34	1.0	0.338315	0.195622	0.901949	Biso	1.000000	H
H35	1.0	0.420787	0.162640	0.880835	Biso	1.000000	H
H36	1.0	0.280471	0.280689	0.458262	Biso	1.000000	H
H37	1.0	0.332747	0.225615	0.434591	Biso	1.000000	H

H38	1.0	0.841234	0.867916	0.353819	Biso	1.000000	H
H39	1.0	0.882886	0.448483	0.938368	Biso	1.000000	H
H40	1.0	0.226042	0.262992	0.248067	Biso	1.000000	H
H41	1.0	0.294060	0.292730	0.300010	Biso	1.000000	H
H42	1.0	0.284678	0.371660	0.254625	Biso	1.000000	H
H43	1.0	0.187404	0.089302	0.426616	Biso	1.000000	H
H44	1.0	0.106843	0.058666	0.324471	Biso	1.000000	H
H45	1.0	0.296528	0.728079	0.380420	Biso	1.000000	H
H46	1.0	0.351102	0.820257	0.402823	Biso	1.000000	H
H47	1.0	0.299702	0.833703	0.352083	Biso	1.000000	H
H48	1.0	0.238319	0.514140	0.104967	Biso	1.000000	H
H49	1.0	0.151451	0.453904	0.158465	Biso	1.000000	H
H50	1.0	0.259799	0.479487	0.183767	Biso	1.000000	H
H51	1.0	0.392029	0.706591	0.717702	Biso	1.000000	H
H52	1.0	0.398660	0.634150	0.794470	Biso	1.000000	H
H53	1.0	0.432112	0.749928	0.799995	Biso	1.000000	H
H54	1.0	0.357318	0.700855	0.813899	Biso	1.000000	H
H55	1.0	0.842328	0.709499	0.666395	Biso	1.000000	H
H56	1.0	0.803643	0.629615	0.617312	Biso	1.000000	H
H57	1.0	0.899224	0.569708	0.725505	Biso	1.000000	H
H58	1.0	0.972880	0.796014	0.832369	Biso	1.000000	H
H59	1.0	0.546608	0.144427	0.912743	Biso	1.000000	H
H60	1.0	0.469445	0.123551	0.888413	Biso	1.000000	H
H61	1.0	0.202973	0.959150	0.087566	Biso	1.000000	H
H62	1.0	0.206232	0.078295	0.101105	Biso	1.000000	H
H63	1.0	0.531139	0.091889	0.749724	Biso	1.000000	H
H64	1.0	0.433747	0.633536	0.414650	Biso	1.000000	H
H65	1.0	0.727062	0.955038	0.979033	Biso	1.000000	H
H66	1.0	0.820285	0.889077	0.991243	Biso	1.000000	H
H67	1.0	0.643189	0.178204	0.936130	Biso	1.000000	H
H68	1.0	0.701492	0.365538	0.894625	Biso	1.000000	H
H69	1.0	0.633554	0.382096	0.804640	Biso	1.000000	H
H70	1.0	0.687328	0.420796	0.833494	Biso	1.000000	H
H71	1.0	0.494059	0.373600	0.136439	Biso	1.000000	H
H72	1.0	0.496700	0.371061	0.049837	Biso	1.000000	H
H73	1.0	0.505758	0.296811	0.090087	Biso	1.000000	H
H74	1.0	0.955831	0.024362	0.484617	Biso	1.000000	H
H75	1.0	0.401982	0.247664	0.334924	Biso	1.000000	H
H76	1.0	0.283178	0.672483	0.400061	Biso	1.000000	H
H77	1.0	0.191803	0.782400	0.752371	Biso	1.000000	H
H78	1.0	0.479819	0.568470	0.979747	Biso	1.000000	H
H79	1.0	0.536418	0.496111	0.026959	Biso	1.000000	H
H80	1.0	0.562228	0.732856	0.632252	Biso	1.000000	H
H81	1.0	0.645747	0.754333	0.570794	Biso	1.000000	H
H82	1.0	0.739893	0.295413	0.421941	Biso	1.000000	H
H83	1.0	0.822550	0.254148	0.350695	Biso	1.000000	H
H84	1.0	0.255019	0.750385	0.359088	Biso	1.000000	H
H85	1.0	0.291743	0.720571	0.275737	Biso	1.000000	H
H86	1.0	0.179377	0.706872	0.304554	Biso	1.000000	H
H87	1.0	0.043116	0.309746	0.126894	Biso	1.000000	H
H88	1.0	0.129458	0.274630	0.110672	Biso	1.000000	H
H89	1.0	0.127292	0.351311	0.052675	Biso	1.000000	H
H90	1.0	0.645167	0.534402	0.173483	Biso	1.000000	H
H91	1.0	0.106533	0.123202	0.487812	Biso	1.000000	H
H92	1.0	0.067547	0.153532	0.558450	Biso	1.000000	H
H93	1.0	0.156926	0.464374	0.631464	Biso	1.000000	H
H94	1.0	0.091702	0.442156	0.534992	Biso	1.000000	H
H95	1.0	0.666119	0.633287	0.992476	Biso	1.000000	H
H96	1.0	0.826097	0.499218	0.406911	Biso	1.000000	H
H97	1.0	0.963425	0.535949	0.207249	Biso	1.000000	H
H98	1.0	0.765307	0.045246	0.714881	Biso	1.000000	H
H99	1.0	0.837120	0.087075	0.676142	Biso	1.000000	H
H100	1.0	0.835243	0.018265	0.624811	Biso	1.000000	H
H101	1.0	0.754994	0.080229	0.615737	Biso	1.000000	H
H102	1.0	0.891100	0.097002	0.997736	Biso	1.000000	H
H103	1.0	0.813717	0.138524	0.999665	Biso	1.000000	H
H104	1.0	0.678361	0.908745	0.381905	Biso	1.000000	H
H105	1.0	0.615254	0.958205	0.445628	Biso	1.000000	H
H106	1.0	0.173919	0.897801	0.205683	Biso	1.000000	H
H107	1.0	0.147422	0.801892	0.173745	Biso	1.000000	H
H108	1.0	0.491748	0.722962	0.168854	Biso	1.000000	H
H109	1.0	0.483993	0.837062	0.162514	Biso	1.000000	H
H110	1.0	0.230375	0.115105	0.552360	Biso	1.000000	H
H111	1.0	0.208101	0.359120	0.399819	Biso	1.000000	H
H112	1.0	0.903780	0.248337	0.599091	Biso	1.000000	H
H113	1.0	0.004368	0.216733	0.614728	Biso	1.000000	H
H114	1.0	0.950912	0.296138	0.604316	Biso	1.000000	H
H115	1.0	0.620753	0.650819	0.448523	Biso	1.000000	H
H116	1.0	0.324654	0.609938	0.997239	Biso	1.000000	H
H117	1.0	0.427020	0.572576	0.170410	Biso	1.000000	H
H118	1.0	0.196313	0.080159	0.228118	Biso	1.000000	H
H119	1.0	0.629616	0.744518	0.727577	Biso	1.000000	H
H120	1.0	0.589329	0.643825	0.677399	Biso	1.000000	H
H121	1.0	0.144121	0.440651	0.681392	Biso	1.000000	H

H122	1.0	0.343534	0.228225	0.707984	Biso	1.000000	H
H123	1.0	0.058621	0.962549	0.631963	Biso	1.000000	H
H124	1.0	0.394576	0.792305	0.756906	Biso	1.000000	H
H125	1.0	0.445273	0.806200	0.816352	Biso	1.000000	H
H126	1.0	0.355001	0.776854	0.864963	Biso	1.000000	H
H127	1.0	0.664668	0.940224	0.281469	Biso	1.000000	H
H128	1.0	0.628742	0.357172	0.033162	Biso	1.000000	H
H129	1.0	0.495774	0.019186	0.548670	Biso	1.000000	H
H130	1.0	0.504598	0.050044	0.461680	Biso	1.000000	H
H131	1.0	0.564621	0.116422	0.524296	Biso	1.000000	H
H132	1.0	0.325525	0.932514	0.818129	Biso	1.000000	H
H133	1.0	0.737816	0.593059	0.524470	Biso	1.000000	H
H134	1.0	0.944262	0.300079	0.833873	Biso	1.000000	H
H135	1.0	0.174894	0.874203	0.763432	Biso	1.000000	H
H136	1.0	0.142190	0.958230	0.811590	Biso	1.000000	H
H137	1.0	0.105975	0.548592	0.764214	Biso	1.000000	H
H138	1.0	0.066686	0.631750	0.817611	Biso	1.000000	H
H139	1.0	0.222678	0.297640	0.524004	Biso	1.000000	H
H140	1.0	0.207059	0.214929	0.745259	Biso	1.000000	H
H141	1.0	0.504994	0.185033	0.176586	Biso	1.000000	H
H142	1.0	0.578873	0.275304	0.162174	Biso	1.000000	H
H143	1.0	0.605015	0.225619	0.201997	Biso	1.000000	H
H144	1.0	0.333209	0.529713	0.192431	Biso	1.000000	H
H145	1.0	0.245144	0.519209	0.213078	Biso	1.000000	H
H146	1.0	0.267591	0.609669	0.191733	Biso	1.000000	H
H147	1.0	0.966397	0.518011	0.123026	Biso	1.000000	H
H148	1.0	0.978645	0.624432	0.110947	Biso	1.000000	H
H149	1.0	0.899039	0.569915	0.055780	Biso	1.000000	H
H150	1.0	0.174224	0.893385	0.562756	Biso	1.000000	H
H151	1.0	0.167953	0.889381	0.465850	Biso	1.000000	H
H152	1.0	0.601292	0.792612	0.795020	Biso	1.000000	H
H153	1.0	0.678655	0.732251	0.759013	Biso	1.000000	H
H154	1.0	0.704880	0.838505	0.775768	Biso	1.000000	H
H155	1.0	0.441093	0.514384	0.458842	Biso	1.000000	H
H156	1.0	0.695674	0.809926	0.428090	Biso	1.000000	H
H157	1.0	0.638827	0.726972	0.392160	Biso	1.000000	H
H158	1.0	0.940192	0.844139	0.759877	Biso	1.000000	H
H159	1.0	0.910991	0.934570	0.719433	Biso	1.000000	H
H160	1.0	0.226721	0.940826	0.971020	Biso	1.000000	H
H161	1.0	0.040272	0.253269	0.977565	Biso	1.000000	H
H162	1.0	0.692687	0.200574	0.902470	Biso	1.000000	H
H163	1.0	0.675946	0.180916	0.816761	Biso	1.000000	H
H164	1.0	0.622138	0.114720	0.873100	Biso	1.000000	H
H165	1.0	0.732166	0.087648	0.976167	Biso	1.000000	H
H166	1.0	0.008036	0.708224	0.267368	Biso	1.000000	H
H167	1.0	0.958031	0.739887	0.204044	Biso	1.000000	H
H168	1.0	0.107075	0.474396	0.005032	Biso	1.000000	H
H169	1.0	0.105703	0.527893	0.061689	Biso	1.000000	H
H170	1.0	0.169569	0.469821	0.056218	Biso	1.000000	H
H171	1.0	0.096912	0.426254	0.115952	Biso	1.000000	H
H172	1.0	0.889105	0.711370	0.101897	Biso	1.000000	H
H173	1.0	0.777627	0.712949	0.099830	Biso	1.000000	H
H174	1.0	0.867964	0.690581	0.036627	Biso	1.000000	H
H175	1.0	0.460862	0.148796	0.312900	Biso	1.000000	H
H176	1.0	0.793989	0.819640	0.057394	Biso	1.000000	H
H177	1.0	0.725298	0.789616	0.079785	Biso	1.000000	H
H178	1.0	0.772683	0.727886	0.042604	Biso	1.000000	H
H179	1.0	0.454737	0.614820	0.012514	Biso	1.000000	H
H180	1.0	0.434206	0.496173	0.050915	Biso	1.000000	H
H181	1.0	0.765648	0.650933	0.942669	Biso	1.000000	H
H182	1.0	0.177723	0.757698	0.195305	Biso	1.000000	H
H183	1.0	0.167073	0.670197	0.147998	Biso	1.000000	H
H184	1.0	0.261273	0.708014	0.216703	Biso	1.000000	H
H185	1.0	0.068661	0.358089	0.884392	Biso	1.000000	H
H186	1.0	0.329887	0.014594	0.810017	Biso	1.000000	H
H187	1.0	0.228619	0.028796	0.810034	Biso	1.000000	H
H188	1.0	0.600390	0.894430	0.438377	Biso	1.000000	H
H189	1.0	0.970551	0.007154	0.985325	Biso	1.000000	H
H190	1.0	0.028770	0.051007	0.893777	Biso	1.000000	H
H191	1.0	0.947032	0.906508	0.622565	Biso	1.000000	H
H192	1.0	0.320049	0.033591	0.703329	Biso	1.000000	H
H193	1.0	0.247674	0.083049	0.692126	Biso	1.000000	H
H194	1.0	0.959494	0.445558	0.216552	Biso	1.000000	H
H195	1.0	0.404678	0.353523	0.075733	Biso	1.000000	H
H196	1.0	0.461228	0.433450	0.141587	Biso	1.000000	H

H197	1.0	0.325685	0.576969	0.606908	Biso	1.000000	H
H198	1.0	0.958580	0.149651	0.233162	Biso	1.000000	H
H199	1.0	0.888993	0.055679	0.248780	Biso	1.000000	H
H200	1.0	0.915434	0.091526	0.317692	Biso	1.000000	H
H201	1.0	0.019020	0.076793	0.395330	Biso	1.000000	H
H202	1.0	0.378956	0.073116	0.570129	Biso	1.000000	H
H203	1.0	0.353443	0.968291	0.927029	Biso	1.000000	H
H204	1.0	0.430691	0.906485	0.933797	Biso	1.000000	H
H205	1.0	0.201659	0.210483	0.834023	Biso	1.000000	H
H206	1.0	0.290771	0.189222	0.875253	Biso	1.000000	H
H207	1.0	0.924473	0.034451	0.206070	Biso	1.000000	H
H208	1.0	0.026423	0.998753	0.181342	Biso	1.000000	H
H209	1.0	0.823946	0.858286	0.805239	Biso	1.000000	H
H210	1.0	0.705710	0.885758	0.803664	Biso	1.000000	H
H211	1.0	0.653664	0.064615	0.717018	Biso	1.000000	H
H212	1.0	0.728033	0.007244	0.683436	Biso	1.000000	H
H213	1.0	0.550397	0.047781	0.711152	Biso	1.000000	H
H214	1.0	0.555404	0.774100	0.238538	Biso	1.000000	H
H215	1.0	0.888273	0.505536	0.112767	Biso	1.000000	H
H216	1.0	0.010533	0.649649	0.371984	Biso	1.000000	H
H217	1.0	0.116495	0.681655	0.342251	Biso	1.000000	H
H218	1.0	0.406181	0.263984	0.177539	Biso	1.000000	H
H219	1.0	0.513147	0.535626	0.673310	Biso	1.000000	H
H220	1.0	0.584982	0.599491	0.628845	Biso	1.000000	H
H221	1.0	0.594210	0.525939	0.638385	Biso	1.000000	H
H222	1.0	0.600217	0.573476	0.731851	Biso	1.000000	H
H223	1.0	0.873004	0.237814	0.995186	Biso	1.000000	H
H224	1.0	0.106656	0.429942	0.297540	Biso	1.000000	H
H225	1.0	0.181160	0.340315	0.281922	Biso	1.000000	H
H226	1.0	0.105417	0.313593	0.315037	Biso	1.000000	H
H227	1.0	0.454957	0.676050	0.032686	Biso	1.000000	H
H228	1.0	0.470406	0.616407	0.942110	Biso	1.000000	H
H229	1.0	0.422730	0.708303	0.949557	Biso	1.000000	H
H230	1.0	0.583894	0.632628	0.908439	Biso	1.000000	H
H231	1.0	0.567499	0.571026	0.844899	Biso	1.000000	H
H232	1.0	0.681293	0.583464	0.883512	Biso	1.000000	H
H233	1.0	0.644994	0.001386	0.465131	Biso	1.000000	H
H234	1.0	0.273399	0.311816	0.811470	Biso	1.000000	H
H235	1.0	0.343294	0.227555	0.853769	Biso	1.000000	H
H236	1.0	0.842764	0.555271	0.782932	Biso	1.000000	H
H237	1.0	0.894016	0.642234	0.794295	Biso	1.000000	H
H238	1.0	0.145407	0.595970	0.655710	Biso	1.000000	H
H239	1.0	0.215444	0.577824	0.710078	Biso	1.000000	H
H240	1.0	0.411870	0.592806	0.584415	Biso	1.000000	H
H241	1.0	0.825221	0.346219	0.413456	Biso	1.000000	H
H242	1.0	0.833608	0.353690	0.518217	Biso	1.000000	H
H243	1.0	0.113796	0.028683	0.920757	Biso	1.000000	H
H244	1.0	0.121605	0.966093	0.999889	Biso	1.000000	H
H245	1.0	0.642488	0.467748	0.538320	Biso	1.000000	H
H246	1.0	0.197979	0.915609	0.681084	Biso	1.000000	H
H247	1.0	0.260513	0.896489	0.645960	Biso	1.000000	H
H248	1.0	0.703135	0.960900	0.115256	Biso	1.000000	H
H249	1.0	0.430999	0.839470	0.133866	Biso	1.000000	H
H250	1.0	0.413340	0.833703	0.035508	Biso	1.000000	H
H251	1.0	0.960158	0.473918	0.675984	Biso	1.000000	H
H252	1.0	0.148837	0.847924	0.018904	Biso	1.000000	H
H253	1.0	0.395846	0.176863	0.468203	Biso	1.000000	H
H254	1.0	0.315690	0.172244	0.419196	Biso	1.000000	H
H255	1.0	0.741690	0.095459	0.766313	Biso	1.000000	H
H256	1.0	0.000489	0.233324	0.340283	Biso	1.000000	H
H257	1.0	0.035964	0.248832	0.233318	Biso	1.000000	H
H258	1.0	0.324484	0.461835	0.534318	Biso	1.000000	H
H259	1.0	0.402518	0.461478	0.596654	Biso	1.000000	H
H260	1.0	0.605551	0.752436	0.159843	Biso	1.000000	H
H261	1.0	0.603171	0.756190	0.258191	Biso	1.000000	H
H262	1.0	0.428853	0.502886	0.691690	Biso	1.000000	H
H263	1.0	0.412205	0.582484	0.633312	Biso	1.000000	H
H264	1.0	0.082772	0.452160	0.859476	Biso	1.000000	H
H265	1.0	0.562920	0.166726	0.612722	Biso	1.000000	H
H266	1.0	0.636321	0.140158	0.651278	Biso	1.000000	H
H267	1.0	0.440642	0.056896	0.056868	Biso	1.000000	H
H268	1.0	0.931030	0.966082	0.823488	Biso	1.000000	H
H269	1.0	0.975758	0.010234	0.806490	Biso	1.000000	H
H270	1.0	0.075469	0.940916	0.243236	Biso	1.000000	H
H271	1.0	0.175182	0.415625	0.078149	Biso	1.000000	H

H272	1.0	0.254681	0.429420	0.041430	Biso	1.000000	H
H273	1.0	0.845790	0.323342	0.970516	Biso	1.000000	H
H274	1.0	0.824886	0.296101	0.868013	Biso	1.000000	H
H275	1.0	0.510902	0.906266	0.527480	Biso	1.000000	H
H276	1.0	0.672689	0.134847	0.288935	Biso	1.000000	H
H277	1.0	0.466103	0.443107	0.849304	Biso	1.000000	H
H278	1.0	0.390001	0.946888	0.261489	Biso	1.000000	H
H279	1.0	0.466664	0.914144	0.231879	Biso	1.000000	H
H280	1.0	0.738167	0.341806	0.988256	Biso	1.000000	H
H281	1.0	0.729080	0.420446	0.908744	Biso	1.000000	H
H282	1.0	0.494232	0.226957	0.936247	Biso	1.000000	H
H283	1.0	0.566790	0.238488	0.005203	Biso	1.000000	H
H284	1.0	0.492561	0.938060	0.925284	Biso	1.000000	H
H285	1.0	0.035252	0.616055	0.920645	Biso	1.000000	H
H286	1.0	0.060300	0.663129	0.967799	Biso	1.000000	H
H287	1.0	0.437570	0.486700	0.137746	Biso	1.000000	H
H288	1.0	0.922299	0.249263	0.851675	Biso	1.000000	H
H289	1.0	0.583396	0.423712	0.221045	Biso	1.000000	H
H290	1.0	0.620646	0.441912	0.309945	Biso	1.000000	H
H291	1.0	0.170886	0.736113	0.002839	Biso	1.000000	H
H292	1.0	0.150335	0.675394	0.909137	Biso	1.000000	H
H293	1.0	0.029063	0.229758	0.563702	Biso	1.000000	H
H294	1.0	0.120105	0.187865	0.561753	Biso	1.000000	H
H295	1.0	0.664580	0.560899	0.625034	Biso	1.000000	H
H296	1.0	0.836735	0.335224	0.289072	Biso	1.000000	H
H297	1.0	0.837870	0.436758	0.316043	Biso	1.000000	H
H298	1.0	0.861482	0.366043	0.362067	Biso	1.000000	H
H299	1.0	0.398638	0.448367	0.042901	Biso	1.000000	H
H300	1.0	0.384243	0.524454	0.125422	Biso	1.000000	H
H301	1.0	0.187293	0.265317	0.759978	Biso	1.000000	H
H302	1.0	0.228579	0.327482	0.847129	Biso	1.000000	H
H303	1.0	0.958893	0.168073	0.926533	Biso	1.000000	H
H304	1.0	0.103814	0.038494	0.688721	Biso	1.000000	H
H305	1.0	0.682764	0.109405	0.649045	Biso	1.000000	H
H306	1.0	0.749171	0.118280	0.576216	Biso	1.000000	H
H307	1.0	0.994682	0.027566	0.333095	Biso	1.000000	H
H308	1.0	0.889894	0.058820	0.361339	Biso	1.000000	H
H309	1.0	0.971421	0.088840	0.412488	Biso	1.000000	H
H310	1.0	0.483553	0.088773	0.640473	Biso	1.000000	H
H311	1.0	0.421395	0.110154	0.552458	Biso	1.000000	H
H312	1.0	0.546935	0.758501	0.789520	Biso	1.000000	H
H313	1.0	0.510635	0.762394	0.867228	Biso	1.000000	H
H314	1.0	0.610294	0.699824	0.856843	Biso	1.000000	H
H315	1.0	0.485985	0.040027	0.059388	Biso	1.000000	H
H316	1.0	0.882142	0.119951	0.142216	Biso	1.000000	H
H317	1.0	0.799584	0.983160	0.740116	Biso	1.000000	H
H318	1.0	0.834424	0.212701	0.563530	Biso	1.000000	H
H319	1.0	0.775503	0.290717	0.492830	Biso	1.000000	H
H320	1.0	0.736315	0.246588	0.553630	Biso	1.000000	H
H321	1.0	0.764095	0.202087	0.488676	Biso	1.000000	H
H322	1.0	0.795580	0.311634	0.215621	Biso	1.000000	H
H323	1.0	0.338439	0.707023	0.439969	Biso	1.000000	H
H324	1.0	0.241078	0.669652	0.443049	Biso	1.000000	H
H325	1.0	0.745818	0.638250	0.699466	Biso	1.000000	H
H326	1.0	0.711753	0.617719	0.762908	Biso	1.000000	H
H327	1.0	0.495326	0.325946	0.353018	Biso	1.000000	H
H328	1.0	0.070750	0.806703	0.031220	Biso	1.000000	H
H329	1.0	0.105336	0.839669	0.974020	Biso	1.000000	H
H330	1.0	0.356875	0.072545	0.694366	Biso	1.000000	H
H331	1.0	0.247293	0.577236	0.480932	Biso	1.000000	H
H332	1.0	0.368579	0.055775	0.470383	Biso	1.000000	H
H333	1.0	0.340912	0.813260	0.599230	Biso	1.000000	H
H334	1.0	0.259885	0.835196	0.656380	Biso	1.000000	H
H335	1.0	0.420565	0.436911	0.877848	Biso	1.000000	H
H336	1.0	0.571541	0.050780	0.145473	Biso	1.000000	H
H337	1.0	0.587628	0.013166	0.225474	Biso	1.000000	H
H338	1.0	0.546143	0.100116	0.254204	Biso	1.000000	H
H339	1.0	0.496901	0.310013	0.711839	Biso	1.000000	H
H340	1.0	0.590318	0.366083	0.683897	Biso	1.000000	H
H341	1.0	0.552978	0.364160	0.753494	Biso	1.000000	H
H342	1.0	0.093817	0.899349	0.138205	Biso	1.000000	H
H343	1.0	0.148868	0.970237	0.074018	Biso	1.000000	H
H344	1.0	0.157150	0.875728	0.069852	Biso	1.000000	H
H345	1.0	0.396365	0.109437	0.450221	Biso	1.000000	H
H346	1.0	0.366939	0.051484	0.230038	Biso	1.000000	H
H347	1.0	0.271138	0.034802	0.181553	Biso	1.000000	H
H348	1.0	0.932747	0.837593	0.828978	Biso	1.000000	H
H349	1.0	0.850201	0.748150	0.814621	Biso	1.000000	H
H350	1.0	0.880921	0.844556	0.785262	Biso	1.000000	H
H351	1.0	0.269724	0.337470	0.514412	Biso	1.000000	H

H352	1.0	0.306621	0.348210	0.595514	Biso	1.000000	H
H353	1.0	0.674077	0.747551	0.930842	Biso	1.000000	H
H354	1.0	0.629437	0.662933	0.893346	Biso	1.000000	H
H355	1.0	0.831376	0.796670	0.019304	Biso	1.000000	H
H356	1.0	0.931582	0.819476	0.992820	Biso	1.000000	H
H357	1.0	0.880223	0.880405	0.975087	Biso	1.000000	H
H358	1.0	0.523722	0.062337	0.328652	Biso	1.000000	H
H359	1.0	0.666088	0.329195	0.530143	Biso	1.000000	H
H360	1.0	0.642056	0.384001	0.528829	Biso	1.000000	H
H361	1.0	0.021345	0.040169	0.221484	Biso	1.000000	H
H362	1.0	0.464201	0.742687	0.050559	Biso	1.000000	H
H363	1.0	0.856189	0.951125	0.560145	Biso	1.000000	H
H364	1.0	0.850764	0.054615	0.585350	Biso	1.000000	H
H365	1.0	0.136772	0.285457	0.448057	Biso	1.000000	H
H366	1.0	0.150481	0.625665	0.880598	Biso	1.000000	H
H367	1.0	0.740075	0.268185	0.081911	Biso	1.000000	H
H368	1.0	0.663222	0.256452	0.047353	Biso	1.000000	H
H369	1.0	0.071239	0.460648	0.334657	Biso	1.000000	H
H370	1.0	0.549622	0.748261	0.512984	Biso	1.000000	H
H371	1.0	0.309850	0.408213	0.591646	Biso	1.000000	H
H372	1.0	0.633779	0.039555	0.319554	Biso	1.000000	H
H373	1.0	0.161950	0.263596	0.029306	Biso	1.000000	H
H374	1.0	0.101170	0.219881	0.934658	Biso	1.000000	H
H375	1.0	0.325523	0.802903	0.067956	Biso	1.000000	H
H376	1.0	0.388129	0.787705	0.013354	Biso	1.000000	H
H377	1.0	0.828268	0.129479	0.356174	Biso	1.000000	H
H378	1.0	0.756794	0.171688	0.436254	Biso	1.000000	H
H379	1.0	0.724902	0.043845	0.769565	Biso	1.000000	H
H380	1.0	0.038438	0.154949	0.846142	Biso	1.000000	H
H381	1.0	0.969810	0.069231	0.846068	Biso	1.000000	H
H382	1.0	0.061840	0.042206	0.845785	Biso	1.000000	H
H383	1.0	0.025911	0.092900	0.193379	Biso	1.000000	H
H384	1.0	0.098842	0.190042	0.166785	Biso	1.000000	H
H385	1.0	0.948216	0.498303	0.179612	Biso	1.000000	H
H386	1.0	0.992856	0.766117	0.368312	Biso	1.000000	H
H387	1.0	0.841940	0.651288	0.503978	Biso	1.000000	H
H388	1.0	0.809301	0.251363	0.231083	Biso	1.000000	H
H389	1.0	0.909507	0.246585	0.210785	Biso	1.000000	H
H390	1.0	0.416709	0.755213	0.634133	Biso	1.000000	H
H391	1.0	0.434654	0.757167	0.744090	Biso	1.000000	H
H392	1.0	0.367725	0.809185	0.654352	Biso	1.000000	H
H393	1.0	0.192439	0.145759	0.810958	Biso	1.000000	H
H394	1.0	0.814602	0.203507	0.013221	Biso	1.000000	H
H395	1.0	0.776044	0.110911	0.526695	Biso	1.000000	H
H396	1.0	0.878322	0.111197	0.583300	Biso	1.000000	H
H397	1.0	0.804797	0.172864	0.599821	Biso	1.000000	H
H398	1.0	0.847090	0.116845	0.500920	Biso	1.000000	H
H399	1.0	0.506123	0.932343	0.030605	Biso	1.000000	H
H400	1.0	0.611237	0.434841	0.699022	Biso	1.000000	H
H401	1.0	0.086334	0.068533	0.882831	Biso	1.000000	H
H402	1.0	0.372427	0.782457	0.918181	Biso	1.000000	H
H403	1.0	0.282408	0.839090	0.927189	Biso	1.000000	H
H404	1.0	0.990401	0.689894	0.500187	Biso	1.000000	H
H405	1.0	0.921761	0.518309	0.350984	Biso	1.000000	H
H406	1.0	0.470209	0.207004	0.651207	Biso	1.000000	H
H407	1.0	0.569073	0.679554	0.415405	Biso	1.000000	H
H408	1.0	0.850324	0.032255	0.871121	Biso	1.000000	H
H409	1.0	0.885840	0.935055	0.817361	Biso	1.000000	H
H410	1.0	0.833001	0.382289	0.710977	Biso	1.000000	H
H411	1.0	0.696696	0.002525	0.985191	Biso	1.000000	H
H412	1.0	0.683063	0.494484	0.560507	Biso	1.000000	H
H413	1.0	0.768646	0.439288	0.571092	Biso	1.000000	H
H414	1.0	0.353072	0.367542	0.072581	Biso	1.000000	H
H415	1.0	0.600115	0.938736	0.842617	Biso	1.000000	H
H416	1.0	0.339104	0.855904	0.442324	Biso	1.000000	H
H417	1.0	0.539009	0.569535	0.751106	Biso	1.000000	H
H418	1.0	0.447268	0.546155	0.714676	Biso	1.000000	H
H419	1.0	0.446504	0.621916	0.798877	Biso	1.000000	H
H420	1.0	0.503254	0.635947	0.735155	Biso	1.000000	H
H421	1.0	0.886992	0.340696	0.130086	Biso	1.000000	H
H422	1.0	0.938287	0.383249	0.218721	Biso	1.000000	H

H423	1.0	0.639091	0.229168	0.095426	Biso	1.000000	H
H424	1.0	0.631523	0.262100	0.173285	Biso	1.000000	H
H425	1.0	0.305371	0.632074	0.724960	Biso	1.000000	H
H426	1.0	0.873661	0.001366	0.663737	Biso	1.000000	H
H427	1.0	0.240959	0.376593	0.017468	Biso	1.000000	H
H428	1.0	0.190209	0.452635	0.999640	Biso	1.000000	H
H429	1.0	0.968100	0.163755	0.519163	Biso	1.000000	H
H430	1.0	0.870069	0.168338	0.496166	Biso	1.000000	H
H431	1.0	0.907787	0.232560	0.553679	Biso	1.000000	H
H432	1.0	0.930403	0.268824	0.499723	Biso	1.000000	H
H433	1.0	0.216876	0.108489	0.980841	Biso	1.000000	H
H434	1.0	0.328021	0.248330	0.146734	Biso	1.000000	H
H435	1.0	0.824372	0.764379	0.695340	Biso	1.000000	H
H436	1.0	0.039076	0.422417	0.506655	Biso	1.000000	H
H437	1.0	0.550488	0.052775	0.445568	Biso	1.000000	H
H438	1.0	0.616730	0.092981	0.472130	Biso	1.000000	H
H439	1.0	0.636541	0.602004	0.265897	Biso	1.000000	H
H440	1.0	0.405307	0.208277	0.176944	Biso	1.000000	H
H441	1.0	0.341742	0.200252	0.257992	Biso	1.000000	H
H442	1.0	0.255162	0.244355	0.131610	Biso	1.000000	H
H443	1.0	0.184934	0.187177	0.051744	Biso	1.000000	H
H444	1.0	0.666495	0.851846	0.972513	Biso	1.000000	H
H445	1.0	0.326583	0.402426	0.919129	Biso	1.000000	H
H446	1.0	0.512147	0.958770	0.506164	Biso	1.000000	H
H447	1.0	0.590835	0.008999	0.451685	Biso	1.000000	H
H448	1.0	0.175589	0.483892	0.673583	Biso	1.000000	H
H449	1.0	0.094283	0.111347	0.983821	Biso	1.000000	H
H450	1.0	0.100254	0.195916	0.995444	Biso	1.000000	H
H451	1.0	0.015658	0.131650	0.053488	Biso	1.000000	H
H452	1.0	0.164129	0.094731	0.703702	Biso	1.000000	H
H453	1.0	0.218558	0.091383	0.797487	Biso	1.000000	H
H454	1.0	0.245318	0.062463	0.756669	Biso	1.000000	H
H455	1.0	0.699183	0.556960	0.542169	Biso	1.000000	H
H456	1.0	0.235916	0.967079	0.353797	Biso	1.000000	H
H457	1.0	0.145264	0.025532	0.312390	Biso	1.000000	H
H458	1.0	0.414775	0.103581	0.118289	Biso	1.000000	H
H459	1.0	0.327551	0.167180	0.165533	Biso	1.000000	H
H460	1.0	0.746508	0.178784	0.828732	Biso	1.000000	H
H461	1.0	0.600525	0.051338	0.515290	Biso	1.000000	H
H462	1.0	0.608192	0.143825	0.290015	Biso	1.000000	H
H463	1.0	0.680601	0.368505	0.568619	Biso	1.000000	H
H464	1.0	0.807108	0.953391	0.988614	Biso	1.000000	H
H465	1.0	0.751954	0.975387	0.026275	Biso	1.000000	H
H466	1.0	0.316495	0.845249	0.507879	Biso	1.000000	H
H467	1.0	0.344204	0.735250	0.483589	Biso	1.000000	H
H468	1.0	0.393730	0.800203	0.548817	Biso	1.000000	H
H469	1.0	0.486120	0.485118	0.454329	Biso	1.000000	H
H470	1.0	0.727046	0.249108	0.851582	Biso	1.000000	H
H471	1.0	0.766849	0.282716	0.764127	Biso	1.000000	H
H472	1.0	0.931263	0.742692	0.032297	Biso	1.000000	H
H473	1.0	0.998215	0.764422	0.125005	Biso	1.000000	H
H474	1.0	0.928438	0.681289	0.074496	Biso	1.000000	H
H475	1.0	0.721292	0.594919	0.983306	Biso	1.000000	H
H476	1.0	0.131533	0.107531	0.749767	Biso	1.000000	H
H477	1.0	0.502650	0.229771	0.468712	Biso	1.000000	H
H478	1.0	0.460272	0.803091	0.036272	Biso	1.000000	H
H479	1.0	0.461536	0.750394	0.955524	Biso	1.000000	H
H480	1.0	0.529987	0.844843	0.981461	Biso	1.000000	H
H481	1.0	0.742619	0.569441	0.796841	Biso	1.000000	H
H482	1.0	0.104981	0.792317	0.654314	Biso	1.000000	H
H483	1.0	0.130502	0.694022	0.605390	Biso	1.000000	H
H484	1.0	0.966946	0.527447	0.508132	Biso	1.000000	H
H485	1.0	0.957431	0.569413	0.450068	Biso	1.000000	H
H486	1.0	0.420545	0.397706	0.574771	Biso	1.000000	H
H487	1.0	0.059644	0.784422	0.220496	Biso	1.000000	H
H488	1.0	0.115041	0.792694	0.125759	Biso	1.000000	H
H489	1.0	0.036397	0.800867	0.132089	Biso	1.000000	H
H490	1.0	0.543197	0.306906	0.268625	Biso	1.000000	H
H491	1.0	0.868503	0.265242	0.560513	Biso	1.000000	H
H492	1.0	0.917556	0.328604	0.493579	Biso	1.000000	H
H493	1.0	0.873460	0.368755	0.562116	Biso	1.000000	H
H494	1.0	0.816597	0.496733	0.846810	Biso	1.000000	H
H495	1.0	0.634279	0.353244	0.294951	Biso	1.000000	H
H496	1.0	0.965829	0.669078	0.285285	Biso	1.000000	H
H497	1.0	0.550746	0.371402	0.986344	Biso	1.000000	H

H498	1.0	0.457455	0.366969	0.003280	Biso	1.000000	H
H499	1.0	0.209018	0.100485	0.659425	Biso	1.000000	H
H500	1.0	0.570760	0.555839	0.087673	Biso	1.000000	H
H501	1.0	0.574198	0.466126	0.048523	Biso	1.000000	H
H502	1.0	0.995349	0.387338	0.524939	Biso	1.000000	H
H503	1.0	0.972518	0.310546	0.515767	Biso	1.000000	H
H504	1.0	0.995942	0.355760	0.438072	Biso	1.000000	H
H505	1.0	0.219674	0.587607	0.283500	Biso	1.000000	H
H506	1.0	0.201255	0.607902	0.362670	Biso	1.000000	H
H507	1.0	0.253889	0.941746	0.687479	Biso	1.000000	H
H508	1.0	0.214623	0.919502	0.778753	Biso	1.000000	H
H509	1.0	0.241406	0.027987	0.728485	Biso	1.000000	H
H510	1.0	0.407401	0.401338	0.992065	Biso	1.000000	H
H511	1.0	0.490880	0.472805	0.999877	Biso	1.000000	H
H512	1.0	0.547124	0.507811	0.836501	Biso	1.000000	H
H513	1.0	0.604029	0.407131	0.884011	Biso	1.000000	H
H514	1.0	0.326402	0.197620	0.957658	Biso	1.000000	H
H515	1.0	0.370613	0.679966	0.653139	Biso	1.000000	H
H516	1.0	0.734136	0.876128	0.535003	Biso	1.000000	H
H517	1.0	0.633150	0.903571	0.482488	Biso	1.000000	H
H518	1.0	0.609819	0.052869	0.880102	Biso	1.000000	H
H519	1.0	0.385725	0.744053	0.269397	Biso	1.000000	H
H520	1.0	0.482394	0.111718	0.968360	Biso	1.000000	H
H521	1.0	0.404194	0.191117	0.021566	Biso	1.000000	H
H522	1.0	0.581222	0.499666	0.247719	Biso	1.000000	H
H523	1.0	0.556429	0.567981	0.198255	Biso	1.000000	H
H524	1.0	0.134820	0.663260	0.095081	Biso	1.000000	H
H525	1.0	0.928301	0.567649	0.891193	Biso	1.000000	H
H526	1.0	0.316072	0.893607	0.545641	Biso	1.000000	H
H527	1.0	0.403535	0.043526	0.153658	Biso	1.000000	H
H528	1.0	0.363570	0.928978	0.162270	Biso	1.000000	H
H529	1.0	0.457977	0.979133	0.139984	Biso	1.000000	H
H530	1.0	0.941346	0.579718	0.023896	Biso	1.000000	H
H531	1.0	0.949703	0.635203	0.059762	Biso	1.000000	H
H532	1.0	0.271912	0.644611	0.883457	Biso	1.000000	H
H533	1.0	0.278899	0.618795	0.776602	Biso	1.000000	H
H534	1.0	0.135791	0.651211	0.497954	Biso	1.000000	H
H535	1.0	0.080914	0.740764	0.537753	Biso	1.000000	H
H536	1.0	0.072732	0.268995	0.453028	Biso	1.000000	H
H537	1.0	0.997784	0.180903	0.474823	Biso	1.000000	H
H538	1.0	0.088279	0.201870	0.525168	Biso	1.000000	H
H539	1.0	0.011788	0.264121	0.520649	Biso	1.000000	H
H540	1.0	0.909468	0.390822	0.491527	Biso	1.000000	H
H541	1.0	0.917309	0.511849	0.506202	Biso	1.000000	H
H542	1.0	0.410619	0.350470	0.979348	Biso	1.000000	H
H543	1.0	0.339714	0.248788	0.964760	Biso	1.000000	H
H544	1.0	0.687306	0.236298	0.516661	Biso	1.000000	H
H545	1.0	0.652988	0.292040	0.209584	Biso	1.000000	H
H546	1.0	0.708571	0.302342	0.268630	Biso	1.000000	H
H547	1.0	0.457232	0.630560	0.301877	Biso	1.000000	H
H548	1.0	0.540985	0.763655	0.159291	Biso	1.000000	H
H549	1.0	0.989741	0.834512	0.983673	Biso	1.000000	H
H550	1.0	0.784940	0.189735	0.781964	Biso	1.000000	H
H551	1.0	0.856068	0.170124	0.690741	Biso	1.000000	H
H552	1.0	0.794552	0.126321	0.685083	Biso	1.000000	H
H553	1.0	0.893560	0.181455	0.723352	Biso	1.000000	H
H554	1.0	0.888325	0.251333	0.760897	Biso	1.000000	H
H555	1.0	0.950492	0.840646	0.908911	Biso	1.000000	H
H556	1.0	0.933690	0.905414	0.817393	Biso	1.000000	H
H557	1.0	0.665654	0.810539	0.327815	Biso	1.000000	H
H558	1.0	0.594704	0.757198	0.316637	Biso	1.000000	H
H559	1.0	0.444470	0.090944	0.214060	Biso	1.000000	H
H560	1.0	0.517572	0.130629	0.212764	Biso	1.000000	H
H561	1.0	0.503381	0.175798	0.281491	Biso	1.000000	H
H562	1.0	0.712118	0.412714	0.727070	Biso	1.000000	H
H563	1.0	0.698848	0.503883	0.673419	Biso	1.000000	H
H564	1.0	0.902950	0.916316	0.261022	Biso	1.000000	H
H565	1.0	0.947323	0.995847	0.313813	Biso	1.000000	H
H566	1.0	0.243136	0.134090	0.100014	Biso	1.000000	H
H567	1.0	0.543582	0.449159	0.510727	Biso	1.000000	H
H568	1.0	0.321002	0.253206	0.663227	Biso	1.000000	H
H569	1.0	0.432461	0.302413	0.657155	Biso	1.000000	H
H570	1.0	0.354814	0.336666	0.652036	Biso	1.000000	H
H571	1.0	0.583190	0.610009	0.087990	Biso	1.000000	H
H572	1.0	0.814819	0.047877	0.903047	Biso	1.000000	H
H573	1.0	0.920189	0.715411	0.874277	Biso	1.000000	H

H574	1.0	0.008810	0.756002	0.850737	Biso	1.000000	H
H575	1.0	0.957523	0.686208	0.822177	Biso	1.000000	H
H576	1.0	0.080208	0.184495	0.809137	Biso	1.000000	H
H577	1.0	0.792324	0.261293	0.104578	Biso	1.000000	H
H578	1.0	0.518129	0.970242	0.844591	Biso	1.000000	H
H579	1.0	0.572794	0.016466	0.794429	Biso	1.000000	H
H580	1.0	0.791035	0.834183	0.116747	Biso	1.000000	H
H581	1.0	0.828082	0.473013	0.367102	Biso	1.000000	H
H582	1.0	0.154541	0.055694	0.119408	Biso	1.000000	H
H583	1.0	0.138485	0.057001	0.222099	Biso	1.000000	H
H584	1.0	0.681412	0.852195	0.396949	Biso	1.000000	H
H585	1.0	0.786583	0.435960	0.072071	Biso	1.000000	H
H586	1.0	0.814751	0.521245	0.060820	Biso	1.000000	H
H587	1.0	0.402667	0.753776	0.471043	Biso	1.000000	H
H588	1.0	0.823283	0.959335	0.340531	Biso	1.000000	H
H589	1.0	0.764076	0.986845	0.270695	Biso	1.000000	H
H590	1.0	0.835383	0.036834	0.341019	Biso	1.000000	H
H591	1.0	0.284898	0.931608	0.333360	Biso	1.000000	H
H592	1.0	0.242530	0.807200	0.429947	Biso	1.000000	H
H593	1.0	0.185368	0.887933	0.367094	Biso	1.000000	H
H594	1.0	0.297528	0.858573	0.395817	Biso	1.000000	H
H595	1.0	0.766827	0.918676	0.619261	Biso	1.000000	H
H596	1.0	0.745365	0.836131	0.682578	Biso	1.000000	H
H597	1.0	0.266868	0.700710	0.879628	Biso	1.000000	H
H598	1.0	0.272670	0.706480	0.998040	Biso	1.000000	H
H599	1.0	0.921521	0.357947	0.045439	Biso	1.000000	H
H600	1.0	0.999438	0.618551	0.732050	Biso	1.000000	H
H601	1.0	0.027805	0.584611	0.650103	Biso	1.000000	H
H602	1.0	0.975059	0.350879	0.094421	Biso	1.000000	H
H603	1.0	0.966655	0.356167	0.181851	Biso	1.000000	H
H604	1.0	0.043365	0.420040	0.118859	Biso	1.000000	H
H605	1.0	0.296836	0.238816	0.269835	Biso	1.000000	H
H606	1.0	0.198218	0.210321	0.258317	Biso	1.000000	H
H607	1.0	0.256061	0.153628	0.327101	Biso	1.000000	H
H608	1.0	0.234518	0.862850	0.783082	Biso	1.000000	H
H609	1.0	0.311365	0.791468	0.835173	Biso	1.000000	H
H610	1.0	0.445154	0.566993	0.223444	Biso	1.000000	H
H611	1.0	0.444929	0.472439	0.239814	Biso	1.000000	H
H612	1.0	0.381671	0.504400	0.167523	Biso	1.000000	H
H613	1.0	0.444267	0.562745	0.821242	Biso	1.000000	H
H614	1.0	0.483432	0.561427	0.919811	Biso	1.000000	H
H615	1.0	0.367829	0.892207	0.279829	Biso	1.000000	H
H616	1.0	0.292406	0.968450	0.250271	Biso	1.000000	H
H617	1.0	0.273932	0.876665	0.295169	Biso	1.000000	H
H618	1.0	0.567394	0.716113	0.461424	Biso	1.000000	H
H619	1.0	0.355202	0.199895	0.131125	Biso	1.000000	H
H620	1.0	0.403543	0.238894	0.049696	Biso	1.000000	H
H621	1.0	0.307721	0.440802	0.175969	Biso	1.000000	H
H622	1.0	0.303632	0.484113	0.283983	Biso	1.000000	H
H623	1.0	0.336444	0.400236	0.228979	Biso	1.000000	H
H624	1.0	0.274944	0.753989	0.845619	Biso	1.000000	H
H625	1.0	0.783725	0.753185	0.758255	Biso	1.000000	H
H626	1.0	0.887929	0.556796	0.481098	Biso	1.000000	H
H627	1.0	0.103485	0.158751	0.764089	Biso	1.000000	H
H628	1.0	0.250807	0.390303	0.512739	Biso	1.000000	H
H629	1.0	0.245829	0.326579	0.438516	Biso	1.000000	H
H630	1.0	0.636832	0.492172	0.439408	Biso	1.000000	H
H631	1.0	0.625197	0.520507	0.524091	Biso	1.000000	H
H632	1.0	0.535698	0.473155	0.460301	Biso	1.000000	H
H633	1.0	0.151156	0.780293	0.282246	Biso	1.000000	H
H634	1.0	0.196374	0.767455	0.373109	Biso	1.000000	H
H635	1.0	0.091181	0.799328	0.331435	Biso	1.000000	H
H636	1.0	0.161131	0.705833	0.352666	Biso	1.000000	H
H637	1.0	0.908938	0.104444	0.796255	Biso	1.000000	H
H638	1.0	0.976364	0.145001	0.852828	Biso	1.000000	H
H639	1.0	0.864713	0.093646	0.456967	Biso	1.000000	H
H640	1.0	0.540477	0.339889	0.650411	Biso	1.000000	H
H641	1.0	0.371399	0.829545	0.216044	Biso	1.000000	H
H642	1.0	0.006295	0.185370	0.252323	Biso	1.000000	H
H643	1.0	0.949538	0.268002	0.194271	Biso	1.000000	H
H644	1.0	0.040171	0.209797	0.181089	Biso	1.000000	H
H645	1.0	0.504941	0.197763	0.117023	Biso	1.000000	H
H646	1.0	0.026634	0.366815	0.595206	Biso	1.000000	H
H647	1.0	0.943149	0.399836	0.538731	Biso	1.000000	H
H648	1.0	0.492311	0.803156	0.594225	Biso	1.000000	H

H649	1.0	0.148209	0.051681	0.728607	Biso	1.000000	H
H650	1.0	0.089509	0.955950	0.725039	Biso	1.000000	H
H651	1.0	0.136746	0.597202	0.531609	Biso	1.000000	H
H652	1.0	0.152844	0.603117	0.430418	Biso	1.000000	H
H653	1.0	0.337268	0.023627	0.123835	Biso	1.000000	H
H654	1.0	0.736909	0.686954	0.125004	Biso	1.000000	H
H655	1.0	0.809845	0.677870	0.189586	Biso	1.000000	H
H656	1.0	0.706364	0.788145	0.551453	Biso	1.000000	H
H657	1.0	0.318284	0.540002	0.145858	Biso	1.000000	H
H658	1.0	0.268805	0.478868	0.079137	Biso	1.000000	H
H659	1.0	0.764659	0.304975	0.566719	Biso	1.000000	H
H660	1.0	0.846323	0.323149	0.584682	Biso	1.000000	H
H661	1.0	0.488181	0.033440	0.304371	Biso	1.000000	H
H662	1.0	0.219067	0.544585	0.255152	Biso	1.000000	H
H663	1.0	0.155177	0.498351	0.202938	Biso	1.000000	H
H664	1.0	0.733303	0.299687	0.723382	Biso	1.000000	H
H665	1.0	0.733018	0.218861	0.680157	Biso	1.000000	H
H666	1.0	0.107191	0.129958	0.311300	Biso	1.000000	H
H667	1.0	0.930862	0.743067	0.352056	Biso	1.000000	H
H668	1.0	0.918336	0.718881	0.415184	Biso	1.000000	H
H669	1.0	0.054795	0.722292	0.230874	Biso	1.000000	H
H670	1.0	0.118359	0.709346	0.256382	Biso	1.000000	H
H671	1.0	0.956216	0.977452	0.946684	Biso	1.000000	H
H672	1.0	0.926105	0.888058	0.938433	Biso	1.000000	H
H673	1.0	0.454924	0.070646	0.863450	Biso	1.000000	H
H674	1.0	0.386813	0.970924	0.882285	Biso	1.000000	H
H675	1.0	0.386730	0.445402	0.688345	Biso	1.000000	H
H676	1.0	0.316630	0.520902	0.682744	Biso	1.000000	H
H677	1.0	0.100058	0.043296	0.629188	Biso	1.000000	H
H678	1.0	0.484894	0.372768	0.326284	Biso	1.000000	H
H679	1.0	0.488788	0.426646	0.233302	Biso	1.000000	H
H680	1.0	0.649105	0.673102	0.402755	Biso	1.000000	H
H681	1.0	0.414810	0.989331	0.588490	Biso	1.000000	H
H682	1.0	0.331843	0.935964	0.585334	Biso	1.000000	H
H683	1.0	0.414604	0.894038	0.627076	Biso	1.000000	H
H684	1.0	0.778770	0.596606	0.572926	Biso	1.000000	H
H685	1.0	0.744406	0.527532	0.658490	Biso	1.000000	H
H686	1.0	0.769946	0.984927	0.905607	Biso	1.000000	H
H687	1.0	0.599307	0.139441	0.483020	Biso	1.000000	H
H688	1.0	0.708752	0.256253	0.908586	Biso	1.000000	H
H689	1.0	0.353605	0.838374	0.315334	Biso	1.000000	H
H690	1.0	0.719016	0.185759	0.150421	Biso	1.000000	H
H691	1.0	0.097161	0.854035	0.301953	Biso	1.000000	H
H692	1.0	0.278454	0.825712	0.310992	Biso	1.000000	H
H693	1.0	0.625521	0.528944	0.275863	Biso	1.000000	H
H694	1.0	0.656509	0.790820	0.523912	Biso	1.000000	H
H695	1.0	0.837398	0.720483	0.206634	Biso	1.000000	H
H696	1.0	0.044412	0.441705	0.243655	Biso	1.000000	H
H697	1.0	0.046999	0.383842	0.322724	Biso	1.000000	H
H698	1.0	0.007776	0.456292	0.354721	Biso	1.000000	H
H699	1.0	0.231241	0.288879	0.104364	Biso	1.000000	H
H700	1.0	0.678925	0.470193	0.476271	Biso	1.000000	H
H701	1.0	0.588241	0.843909	0.887246	Biso	1.000000	H
H702	1.0	0.663945	0.805959	0.927874	Biso	1.000000	H
H703	1.0	0.480434	0.559102	0.381578	Biso	1.000000	H
H704	1.0	0.358736	0.768500	0.210848	Biso	1.000000	H
H705	1.0	0.337699	0.740991	0.130417	Biso	1.000000	H
H706	1.0	0.878517	0.323070	0.802394	Biso	1.000000	H
H707	1.0	0.801607	0.241400	0.757639	Biso	1.000000	H
H708	1.0	0.783527	0.268593	0.831518	Biso	1.000000	H
H709	1.0	0.743892	0.415789	0.257267	Biso	1.000000	H
H710	1.0	0.241859	0.367415	0.873996	Biso	1.000000	H
H711	1.0	0.151907	0.418956	0.963989	Biso	1.000000	H
H712	1.0	0.679688	0.482080	0.094112	Biso	1.000000	H
H713	1.0	0.457960	0.220560	0.706452	Biso	1.000000	H
H714	1.0	0.496098	0.169956	0.762862	Biso	1.000000	H
H715	1.0	0.674415	0.447878	0.977762	Biso	1.000000	H
H716	1.0	0.725779	0.804385	0.333267	Biso	1.000000	H
H717	1.0	0.697848	0.049392	0.014495	Biso	1.000000	H
H718	1.0	0.735834	0.992049	0.071555	Biso	1.000000	H
H719	1.0	0.986263	0.837426	0.516587	Biso	1.000000	H
H720	1.0	0.575303	0.520217	0.414546	Biso	1.000000	H
H721	1.0	0.116777	0.573246	0.169876	Biso	1.000000	H
H722	1.0	0.019346	0.585855	0.185588	Biso	1.000000	H
H723	1.0	0.847123	0.899820	0.426289	Biso	1.000000	H
H724	1.0	0.835161	0.621113	0.200898	Biso	1.000000	H
H725	1.0	0.231198	0.970543	0.904138	Biso	1.000000	H
H726	1.0	0.052081	0.677387	0.641806	Biso	1.000000	H
H727	1.0	0.595169	0.952645	0.970837	Biso	1.000000	H
H728	1.0	0.579156	0.846425	0.949802	Biso	1.000000	H
H729	1.0	0.650666	0.903682	0.005998	Biso	1.000000	H
H730	1.0	0.936432	0.834015	0.354972	Biso	1.000000	H
H731	1.0	0.685536	0.152340	0.450507	Biso	1.000000	H
H732	1.0	0.097759	0.802247	0.387019	Biso	1.000000	H
H733	1.0	0.175224	0.505973	0.727277	Biso	1.000000	H

H734	1.0	0.251037	0.474137	0.807867	Biso	1.000000	H
H735	1.0	0.313635	0.602265	0.853153	Biso	1.000000	H
H736	1.0	0.335592	0.631625	0.931341	Biso	1.000000	H
H737	1.0	0.925465	0.131815	0.190392	Biso	1.000000	H
H738	1.0	0.999852	0.128441	0.147939	Biso	1.000000	H
H739	1.0	0.683052	0.804289	0.130216	Biso	1.000000	H
H740	1.0	0.810760	0.685060	0.546543	Biso	1.000000	H
H741	1.0	0.868500	0.842168	0.413058	Biso	1.000000	H
H742	1.0	0.917119	0.930026	0.396518	Biso	1.000000	H
H743	1.0	0.957331	0.867493	0.472259	Biso	1.000000	H
H744	1.0	0.243762	0.113727	0.372681	Biso	1.000000	H
H745	1.0	0.251466	0.073788	0.272872	Biso	1.000000	H
H746	1.0	0.634840	0.909327	0.544445	Biso	1.000000	H
H747	1.0	0.562780	0.921933	0.623475	Biso	1.000000	H
H748	1.0	0.489582	0.592270	0.659147	Biso	1.000000	H
H749	1.0	0.541450	0.685219	0.624094	Biso	1.000000	H
H750	1.0	0.636981	0.248487	0.233114	Biso	1.000000	H
H751	1.0	0.673593	0.161704	0.186661	Biso	1.000000	H
H752	1.0	0.645709	0.174230	0.767915	Biso	1.000000	H
H753	1.0	0.583503	0.254259	0.784194	Biso	1.000000	H
H754	1.0	0.351074	0.955234	0.472771	Biso	1.000000	H
H755	1.0	0.136813	0.579602	0.919516	Biso	1.000000	H
H756	1.0	0.076865	0.511352	0.901396	Biso	1.000000	H
H757	1.0	0.146632	0.526928	0.997302	Biso	1.000000	H
H758	1.0	0.493490	0.955586	0.561124	Biso	1.000000	H
H759	1.0	0.476930	0.955081	0.664023	Biso	1.000000	H
H760	1.0	0.448169	0.040116	0.620128	Biso	1.000000	H
H761	1.0	0.872174	0.436704	0.566916	Biso	1.000000	H
H762	1.0	0.949851	0.457834	0.376394	Biso	1.000000	H
H763	1.0	0.969462	0.376206	0.390252	Biso	1.000000	H
H764	1.0	0.671821	0.279383	0.487735	Biso	1.000000	H
H765	1.0	0.201980	0.431104	0.324005	Biso	1.000000	H
H766	1.0	0.179033	0.386881	0.245110	Biso	1.000000	H
H767	1.0	0.659438	0.727554	0.039350	Biso	1.000000	H
H768	1.0	0.678009	0.803701	0.047823	Biso	1.000000	H
H769	1.0	0.959010	0.969984	0.458523	Biso	1.000000	H
H770	1.0	0.866219	0.950969	0.417836	Biso	1.000000	H
H771	1.0	0.675509	0.218248	0.276710	Biso	1.000000	H
H772	1.0	0.587439	0.269582	0.269302	Biso	1.000000	H
H773	1.0	0.184029	0.952182	0.987978	Biso	1.000000	H
H774	1.0	0.137824	0.064827	0.989916	Biso	1.000000	H
H775	1.0	0.808966	0.649405	0.984522	Biso	1.000000	H
H776	1.0	0.023568	0.389812	0.796179	Biso	1.000000	H
H777	1.0	0.969523	0.461520	0.755966	Biso	1.000000	H
H778	1.0	0.028278	0.472797	0.844501	Biso	1.000000	H
H779	1.0	0.068476	0.423093	0.755189	Biso	1.000000	H
H780	1.0	0.086804	0.786907	0.947733	Biso	1.000000	H
H781	1.0	0.626813	0.647173	0.114509	Biso	1.000000	H
H782	1.0	0.581896	0.555203	0.140497	Biso	1.000000	H
H783	1.0	0.656987	0.194301	0.142675	Biso	1.000000	H
H784	1.0	0.221577	0.472271	0.045361	Biso	1.000000	H
H785	1.0	0.258416	0.532048	0.057753	Biso	1.000000	H
H786	1.0	0.183759	0.580434	0.992793	Biso	1.000000	H
H787	1.0	0.729148	0.513391	0.114531	Biso	1.000000	H
H788	1.0	0.624867	0.541109	0.116222	Biso	1.000000	H
H789	1.0	0.296327	0.702218	0.121689	Biso	1.000000	H
H790	1.0	0.504180	0.994469	0.460301	Biso	1.000000	H
H791	1.0	0.393334	0.603714	0.301599	Biso	1.000000	H
H792	1.0	0.775989	0.386140	0.095316	Biso	1.000000	H
H793	1.0	0.937699	0.018095	0.865050	Biso	1.000000	H
H794	1.0	0.978306	0.165568	0.630859	Biso	1.000000	H
H795	1.0	0.009232	0.141505	0.557539	Biso	1.000000	H
H796	1.0	0.911499	0.166883	0.571874	Biso	1.000000	H
H797	1.0	0.835279	0.959193	0.163142	Biso	1.000000	H
H798	1.0	0.758885	0.936154	0.245390	Biso	1.000000	H
H799	1.0	0.793802	0.022725	0.175173	Biso	1.000000	H
H800	1.0	0.751564	0.224655	0.127575	Biso	1.000000	H
H801	1.0	0.932148	0.321014	0.687795	Biso	1.000000	H
H802	1.0	0.941602	0.400401	0.767711	Biso	1.000000	H

H803	1.0	0.317570	0.411575	0.332684	Biso	1.000000	H
H804	1.0	0.516571	0.915961	0.233517	Biso	1.000000	H
H805	1.0	0.254577	0.404323	0.568213	Biso	1.000000	H
H806	1.0	0.521380	0.584923	0.309804	Biso	1.000000	H
H807	1.0	0.984440	0.505025	0.071722	Biso	1.000000	H
H808	1.0	0.969202	0.424450	0.981033	Biso	1.000000	H
H809	1.0	0.934462	0.531610	0.024590	Biso	1.000000	H
H810	1.0	0.632471	0.999829	0.795454	Biso	1.000000	H
H811	1.0	0.616909	0.908847	0.753158	Biso	1.000000	H
H812	1.0	0.563610	0.988943	0.710972	Biso	1.000000	H
H813	1.0	0.312263	0.513865	0.319390	Biso	1.000000	H
H814	1.0	0.797336	0.662496	0.892788	Biso	1.000000	H
H815	1.0	0.881040	0.673807	0.848320	Biso	1.000000	H
O1616	1.0	0.558584	0.645432	0.810007	Biso	1.000000	O
H816	1.0	0.571541	0.622470	0.819598	Biso	1.000000	H
H817	1.0	0.571382	0.668834	0.818792	Biso	1.000000	H
O1617	1.0	0.351896	0.814946	0.101575	Biso	1.000000	O
H818	1.0	0.355919	0.789545	0.113090	Biso	1.000000	H
H819	1.0	0.375051	0.830162	0.103031	Biso	1.000000	H
O1618	1.0	0.134767	0.647275	0.051373	Biso	1.000000	O
H820	1.0	0.121378	0.652338	0.028071	Biso	1.000000	H
H821	1.0	0.161838	0.649288	0.047101	Biso	1.000000	H
O1619	1.0	0.325121	0.432027	0.951768	Biso	1.000000	O
H822	1.0	0.298627	0.439120	0.954242	Biso	1.000000	H
H823	1.0	0.339530	0.454682	0.943650	Biso	1.000000	H
O1620	1.0	0.131715	0.352517	0.008422	Biso	1.000000	O
H824	1.0	0.159043	0.354531	0.010544	Biso	1.000000	H
H825	1.0	0.125300	0.331035	0.991770	Biso	1.000000	H
O1621	1.0	0.667275	0.923455	0.635329	Biso	1.000000	O
H826	1.0	0.692657	0.931980	0.628828	Biso	1.000000	H
H827	1.0	0.651631	0.923128	0.612921	Biso	1.000000	H
O1622	1.0	0.924377	0.552809	0.757882	Biso	1.000000	O
H828	1.0	0.946883	0.551917	0.773501	Biso	1.000000	H
H829	1.0	0.902766	0.560382	0.773067	Biso	1.000000	H
O1623	1.0	0.145619	0.978677	0.639184	Biso	1.000000	O
H830	1.0	0.168163	0.991783	0.648388	Biso	1.000000	H
H831	1.0	0.131361	0.996455	0.623317	Biso	1.000000	H
O1624	1.0	0.718271	0.463667	0.899000	Biso	1.000000	O
H832	1.0	0.715938	0.486683	0.914798	Biso	1.000000	H
H833	1.0	0.698260	0.463741	0.880320	Biso	1.000000	H
O1625	1.0	0.833809	0.350569	0.051565	Biso	1.000000	O
H834	1.0	0.838829	0.353934	0.024961	Biso	1.000000	H
H835	1.0	0.806735	0.352003	0.056014	Biso	1.000000	H
O1626	1.0	0.532282	0.923901	0.910102	Biso	1.000000	O
H836	1.0	0.536110	0.945469	0.892780	Biso	1.000000	H
H837	1.0	0.551824	0.924435	0.929261	Biso	1.000000	H
O1627	1.0	0.036445	0.220431	0.066800	Biso	1.000000	O
H838	1.0	0.035332	0.192133	0.064804	Biso	1.000000	H
H839	1.0	0.040593	0.231646	0.042099	Biso	1.000000	H
O1628	1.0	0.935215	0.275493	0.149842	Biso	1.000000	O
H840	1.0	0.943753	0.249046	0.144661	Biso	1.000000	H
H841	1.0	0.939593	0.291816	0.127965	Biso	1.000000	H
O1629	1.0	0.537822	0.662161	0.742288	Biso	1.000000	O
H842	1.0	0.548457	0.655184	0.766512	Biso	1.000000	H
H843	1.0	0.558059	0.664782	0.724020	Biso	1.000000	H
O1630	1.0	0.867648	0.886857	0.888574	Biso	1.000000	O
H844	1.0	0.892279	0.874564	0.891209	Biso	1.000000	H
H845	1.0	0.867088	0.902183	0.865636	Biso	1.000000	H
O1631	1.0	0.938199	0.159051	0.398456	Biso	1.000000	O
H846	1.0	0.955515	0.146610	0.415931	Biso	1.000000	H
H847	1.0	0.949618	0.159845	0.373676	Biso	1.000000	H
O1632	1.0	0.988706	0.337714	0.915747	Biso	1.000000	O
H848	1.0	0.012945	0.324702	0.918745	Biso	1.000000	H
H849	1.0	0.990643	0.357317	0.896119	Biso	1.000000	H
O1633	1.0	0.454627	0.337978	0.635512	Biso	1.000000	O
H850	1.0	0.481558	0.333971	0.631691	Biso	1.000000	H
H851	1.0	0.443676	0.349618	0.613147	Biso	1.000000	H
O1634	1.0	0.219456	0.599031	0.510454	Biso	1.000000	O
H852	1.0	0.209329	0.581243	0.529178	Biso	1.000000	H
H853	1.0	0.212195	0.625645	0.516661	Biso	1.000000	H
O1635	1.0	0.971173	0.110612	0.453432	Biso	1.000000	O
H854	1.0	0.963489	0.132400	0.469162	Biso	1.000000	H
H855	1.0	0.970330	0.086588	0.467941	Biso	1.000000	H
O1636	1.0	0.439488	0.107398	0.803661	Biso	1.000000	O
H856	1.0	0.465531	0.104481	0.795421	Biso	1.000000	H
H857	1.0	0.434201	0.088593	0.823401	Biso	1.000000	H
O1637	1.0	0.770449	0.674238	0.983816	Biso	1.000000	O
H858	1.0	0.759017	0.660264	0.004661	Biso	1.000000	H
H859	1.0	0.769047	0.702187	0.988441	Biso	1.000000	H
O1638	1.0	0.076849	0.533695	0.860441	Biso	1.000000	O
H860	1.0	0.077802	0.556461	0.844174	Biso	1.000000	H
H861	1.0	0.087637	0.511346	0.847472	Biso	1.000000	H
O1639	1.0	0.646852	0.140329	0.739219	Biso	1.000000	O
H862	1.0	0.642430	0.147819	0.713294	Biso	1.000000	H

H863	1.0	0.635008	0.115210	0.744071	Biso	1.000000	H
O1640	1.0	0.460145	0.902126	0.386673	Biso	1.000000	O
H864	1.0	0.432788	0.899800	0.387954	Biso	1.000000	H
H865	1.0	0.467213	0.929546	0.387757	Biso	1.000000	H
O1641	1.0	0.261275	0.241594	0.368996	Biso	1.000000	O
H866	1.0	0.261422	0.218849	0.352674	Biso	1.000000	H
H867	1.0	0.275549	0.235880	0.391631	Biso	1.000000	H
O1642	1.0	0.902345	0.447830	0.376620	Biso	1.000000	O
H868	1.0	0.879850	0.459848	0.387207	Biso	1.000000	H
H869	1.0	0.896151	0.421878	0.367418	Biso	1.000000	H
O1643	1.0	0.108269	0.963384	0.220078	Biso	1.000000	O
H870	1.0	0.133638	0.952554	0.218770	Biso	1.000000	H
H871	1.0	0.109446	0.989865	0.229850	Biso	1.000000	H
O1644	1.0	0.766888	0.598721	0.710121	Biso	1.000000	O
H872	1.0	0.762944	0.580152	0.689874	Biso	1.000000	H
H873	1.0	0.757981	0.587496	0.733546	Biso	1.000000	H
O1645	1.0	0.207872	0.787867	0.706120	Biso	1.000000	O
H874	1.0	0.230114	0.796937	0.692685	Biso	1.000000	H
H875	1.0	0.200686	0.762201	0.696882	Biso	1.000000	H
O1646	1.0	0.552788	0.412904	0.969361	Biso	1.000000	O
H876	1.0	0.529567	0.422309	0.957912	Biso	1.000000	H
H877	1.0	0.563239	0.433132	0.985443	Biso	1.000000	H
O1647	1.0	0.280710	0.995697	0.283339	Biso	1.000000	O
H878	1.0	0.280561	0.024088	0.283887	Biso	1.000000	H
H879	1.0	0.282166	0.985703	0.308811	Biso	1.000000	H
O1648	1.0	0.369305	0.965034	0.331201	Biso	1.000000	O
H880	1.0	0.363391	0.948084	0.310133	Biso	1.000000	H
H881	1.0	0.362473	0.952045	0.354473	Biso	1.000000	H
O1649	1.0	0.691434	0.776871	0.348253	Biso	1.000000	O
H882	1.0	0.684737	0.786242	0.373112	Biso	1.000000	H
H883	1.0	0.702967	0.751180	0.350270	Biso	1.000000	H
O1650	1.0	0.693418	0.083004	0.463983	Biso	1.000000	O
H884	1.0	0.682637	0.057585	0.458213	Biso	1.000000	H
H885	1.0	0.688018	0.101041	0.443624	Biso	1.000000	H
O1651	1.0	0.270927	0.237301	0.469868	Biso	1.000000	O
H886	1.0	0.284315	0.213792	0.462290	Biso	1.000000	H
H887	1.0	0.256457	0.232024	0.492481	Biso	1.000000	H
O1652	1.0	0.666437	0.411180	0.954091	Biso	1.000000	O
H888	1.0	0.643117	0.400520	0.943934	Biso	1.000000	H
H889	1.0	0.687804	0.401159	0.939902	Biso	1.000000	H
O1653	1.0	0.590689	0.305614	0.919315	Biso	1.000000	O
H890	1.0	0.597719	0.291145	0.941709	Biso	1.000000	H
H891	1.0	0.603693	0.294667	0.897724	Biso	1.000000	H
O1654	1.0	0.171210	0.177432	0.547876	Biso	1.000000	O
H892	1.0	0.176319	0.166155	0.572373	Biso	1.000000	H
H893	1.0	0.192425	0.193664	0.540299	Biso	1.000000	H
O1655	1.0	0.654890	0.121755	0.208296	Biso	1.000000	O
H894	1.0	0.658693	0.096957	0.195560	Biso	1.000000	H
H895	1.0	0.669452	0.122093	0.231410	Biso	1.000000	H
O1656	1.0	0.752760	0.698978	0.638292	Biso	1.000000	O
H896	1.0	0.741366	0.684225	0.658656	Biso	1.000000	H
H897	1.0	0.773002	0.683726	0.627092	Biso	1.000000	H
O1657	1.0	0.423895	0.039707	0.507827	Biso	1.000000	O
H898	1.0	0.422071	0.057866	0.486948	Biso	1.000000	H
H899	1.0	0.448622	0.042117	0.519493	Biso	1.000000	H
O1658	1.0	0.634587	0.871906	0.678903	Biso	1.000000	O
H900	1.0	0.645887	0.870809	0.703727	Biso	1.000000	H
H901	1.0	0.649094	0.889583	0.663151	Biso	1.000000	H
O1659	1.0	0.050330	0.577898	0.019266	Biso	1.000000	O
H902	1.0	0.041769	0.601808	0.031273	Biso	1.000000	H
H903	1.0	0.075997	0.572110	0.027264	Biso	1.000000	H
O1660	1.0	0.656514	0.558065	0.262419	Biso	1.000000	O
H904	1.0	0.656789	0.546732	0.237427	Biso	1.000000	H
H905	1.0	0.680246	0.552298	0.274993	Biso	1.000000	H
O1661	1.0	0.622894	0.412481	0.770859	Biso	1.000000	O
H906	1.0	0.628791	0.405032	0.745215	Biso	1.000000	H
H907	1.0	0.622990	0.440793	0.772964	Biso	1.000000	H
O1662	1.0	0.127986	0.795151	0.083611	Biso	1.000000	O
H908	1.0	0.114637	0.780330	0.064499	Biso	1.000000	H
H909	1.0	0.145809	0.812836	0.071685	Biso	1.000000	H
O1663	1.0	0.667897	0.712875	0.298129	Biso	1.000000	O
H910	1.0	0.689933	0.711075	0.314320	Biso	1.000000	H
H911	1.0	0.658789	0.739665	0.297772	Biso	1.000000	H
O1664	1.0	0.969188	0.576586	0.184252	Biso	1.000000	O
H912	1.0	0.952786	0.573136	0.162634	Biso	1.000000	H
H913	1.0	0.964594	0.602067	0.195389	Biso	1.000000	H
O1665	1.0	0.295151	0.733759	0.228155	Biso	1.000000	O
H914	1.0	0.288099	0.761088	0.230603	Biso	1.000000	H
H915	1.0	0.320738	0.731927	0.218370	Biso	1.000000	H

O1666	1.0	0.072812	0.107381	0.102288	Biso	1.000000	O
H916	1.0	0.058701	0.121496	0.084290	Biso	1.000000	H
H917	1.0	0.061604	0.082497	0.105342	Biso	1.000000	H
H918	1.0	0.096699	0.104154	0.090781	Biso	1.000000	H
O1667	1.0	0.824399	0.928356	0.924641	Biso	1.000000	O
H919	1.0	0.815166	0.920204	0.948268	Biso	1.000000	H
H920	1.0	0.841940	0.948598	0.929455	Biso	1.000000	H
H921	1.0	0.839230	0.908081	0.914231	Biso	1.000000	H
O1668	1.0	0.878921	0.252621	0.919666	Biso	1.000000	O
H922	1.0	0.890163	0.277648	0.918254	Biso	1.000000	H
H923	1.0	0.887924	0.239744	0.897967	Biso	1.000000	H
H924	1.0	0.888993	0.241356	0.941726	Biso	1.000000	H
O1669	1.0	0.253860	0.287664	0.711394	Biso	1.000000	O
H925	1.0	0.234501	0.294601	0.728433	Biso	1.000000	H
H926	1.0	0.254254	0.306412	0.691913	Biso	1.000000	H
H927	1.0	0.277019	0.291168	0.724216	Biso	1.000000	H
O1670	1.0	0.042375	0.549708	0.117621	Biso	1.000000	O
H928	1.0	0.042894	0.564906	0.139765	Biso	1.000000	H
H929	1.0	0.048772	0.524727	0.127011	Biso	1.000000	H
H930	1.0	0.016678	0.546479	0.110979	Biso	1.000000	H
O1671	1.0	0.845403	0.293724	0.709914	Biso	1.000000	O
H931	1.0	0.868915	0.283838	0.718247	Biso	1.000000	H
H932	1.0	0.828966	0.288039	0.730109	Biso	1.000000	H
H933	1.0	0.837925	0.274794	0.692074	Biso	1.000000	H
O1672	1.0	0.621987	0.669237	0.328058	Biso	1.000000	O
H934	1.0	0.633510	0.644376	0.326243	Biso	1.000000	H
H935	1.0	0.635587	0.684546	0.310580	Biso	1.000000	H
H936	1.0	0.631955	0.678753	0.350911	Biso	1.000000	H
O1673	1.0	0.157332	0.801515	0.443278	Biso	1.000000	O
H937	1.0	0.162315	0.824817	0.456645	Biso	1.000000	H
H938	1.0	0.177681	0.800109	0.426128	Biso	1.000000	H
H939	1.0	0.135609	0.807381	0.428878	Biso	1.000000	H
O1674	1.0	0.953042	0.524213	0.623156	Biso	1.000000	O
H940	1.0	0.962903	0.545315	0.609102	Biso	1.000000	H
H941	1.0	0.950426	0.503256	0.606071	Biso	1.000000	H
H942	1.0	0.928545	0.532601	0.630091	Biso	1.000000	H
O1675	1.0	0.939541	0.231272	0.280079	Biso	1.000000	O
H943	1.0	0.945298	0.237873	0.254969	Biso	1.000000	H
H944	1.0	0.924302	0.251555	0.289876	Biso	1.000000	H
H945	1.0	0.962782	0.233219	0.293059	Biso	1.000000	H
O1676	1.0	0.182337	0.602867	0.198599	Biso	1.000000	O
H946	1.0	0.188050	0.623305	0.181674	Biso	1.000000	H
H947	1.0	0.156308	0.598372	0.194329	Biso	1.000000	H
H948	1.0	0.182177	0.615632	0.222121	Biso	1.000000	H
O1677	1.0	0.042784	0.294448	0.620689	Biso	1.000000	O
H949	1.0	0.040536	0.269813	0.608887	Biso	1.000000	H
H950	1.0	0.032967	0.291046	0.645142	Biso	1.000000	H
H951	1.0	0.025551	0.311225	0.608366	Biso	1.000000	H
O1678	1.0	0.967860	0.070037	0.747813	Biso	1.000000	O
H952	1.0	0.957442	0.061030	0.724966	Biso	1.000000	H
H953	1.0	0.951431	0.088871	0.758351	Biso	1.000000	H
H954	1.0	0.968524	0.047995	0.763791	Biso	1.000000	H
O1679	1.0	0.764101	0.899962	0.355700	Biso	1.000000	O
H955	1.0	0.783354	0.880839	0.354370	Biso	1.000000	H
H956	1.0	0.759589	0.900482	0.381837	Biso	1.000000	H
H957	1.0	0.776066	0.923952	0.350019	Biso	1.000000	H
O1680	1.0	0.643741	0.902583	0.908679	Biso	1.000000	O
H958	1.0	0.667850	0.911917	0.915834	Biso	1.000000	H
H959	1.0	0.634672	0.920436	0.890547	Biso	1.000000	H
H960	1.0	0.649685	0.880155	0.894352	Biso	1.000000	H
O1681	1.0	0.100458	0.027877	0.057907	Biso	1.000000	O
H961	1.0	0.104645	0.000814	0.061275	Biso	1.000000	H
H962	1.0	0.076676	0.031538	0.046305	Biso	1.000000	H
H963	1.0	0.117977	0.035603	0.039293	Biso	1.000000	H
O1682	1.0	0.035409	0.116621	0.932131	Biso	1.000000	O
H964	1.0	0.025635	0.091251	0.928083	Biso	1.000000	H
H965	1.0	0.014297	0.133496	0.933718	Biso	1.000000	H
H966	1.0	0.046446	0.115832	0.956277	Biso	1.000000	H
O1683	1.0	0.676229	0.643913	0.610176	Biso	1.000000	O
H967	1.0	0.697210	0.647181	0.594037	Biso	1.000000	H
H968	1.0	0.667460	0.617904	0.608066	Biso	1.000000	H
H969	1.0	0.656715	0.659528	0.599976	Biso	1.000000	H
O1684	1.0	0.835350	0.937918	0.683273	Biso	1.000000	O
H970	1.0	0.820967	0.919982	0.697537	Biso	1.000000	H
H971	1.0	0.860602	0.931809	0.689727	Biso	1.000000	H
H972	1.0	0.831547	0.961840	0.695994	Biso	1.000000	H
O1685	1.0	0.490833	0.450800	0.069590	Biso	1.000000	O
H973	1.0	0.513939	0.464584	0.067855	Biso	1.000000	H
H974	1.0	0.479000	0.453671	0.045967	Biso	1.000000	H
H975	1.0	0.498250	0.424277	0.070655	Biso	1.000000	H
O1686	1.0	0.539133	0.304691	0.808368	Biso	1.000000	O
H976	1.0	0.512721	0.302118	0.805040	Biso	1.000000	H
H977	1.0	0.543964	0.326494	0.823938	Biso	1.000000	H
H978	1.0	0.547677	0.314156	0.784938	Biso	1.000000	H

O1687	1.0	0.349915	0.082192	0.302599	Biso	1.000000	O
H979	1.0	0.330016	0.073622	0.318286	Biso	1.000000	H
H980	1.0	0.342319	0.078311	0.277446	Biso	1.000000	H
H981	1.0	0.369885	0.064223	0.306287	Biso	1.000000	H
O1688	1.0	0.116976	0.206369	0.076755	Biso	1.000000	O
H982	1.0	0.109092	0.232587	0.079737	Biso	1.000000	H
H983	1.0	0.108587	0.197966	0.052899	Biso	1.000000	H
H984	1.0	0.143605	0.208013	0.074939	Biso	1.000000	H
O1689	1.0	0.530911	0.252765	0.650907	Biso	1.000000	O
H985	1.0	0.544739	0.264013	0.630938	Biso	1.000000	H
H986	1.0	0.524837	0.271719	0.669241	Biso	1.000000	H
H987	1.0	0.508660	0.241609	0.640830	Biso	1.000000	H
O1690	1.0	0.164392	0.928907	0.912072	Biso	1.000000	O
H988	1.0	0.139387	0.933559	0.920337	Biso	1.000000	H
H989	1.0	0.179121	0.921249	0.932952	Biso	1.000000	H
H990	1.0	0.176000	0.952951	0.905882	Biso	1.000000	H
O1691	1.0	0.363998	0.854998	0.726833	Biso	1.000000	O
H991	1.0	0.370052	0.840092	0.705324	Biso	1.000000	H
H992	1.0	0.364652	0.836571	0.746584	Biso	1.000000	H
H993	1.0	0.339043	0.864647	0.724620	Biso	1.000000	H
O1692	1.0	0.839863	0.691612	0.763650	Biso	1.000000	O
H994	1.0	0.857601	0.705001	0.748527	Biso	1.000000	H
H995	1.0	0.816424	0.704603	0.760913	Biso	1.000000	H
H996	1.0	0.836403	0.667122	0.751861	Biso	1.000000	H
O1693	1.0	0.804050	0.360361	0.821686	Biso	1.000000	O
H997	1.0	0.822462	0.343365	0.811506	Biso	1.000000	H
H998	1.0	0.818320	0.382718	0.828212	Biso	1.000000	H
H999	1.0	0.789216	0.368085	0.800903	Biso	1.000000	H
O1694	1.0	0.215697	0.547853	0.414096	Biso	1.000000	O
H1000	1.0	0.195178	0.562424	0.423767	Biso	1.000000	H
H1001	1.0	0.208210	0.521362	0.415369	Biso	1.000000	H
H1002	1.0	0.235418	0.551074	0.431737	Biso	1.000000	H
O1695	1.0	0.806886	0.561390	0.985231	Biso	1.000000	O
H1003	1.0	0.816891	0.586678	0.989204	Biso	1.000000	H
H1004	1.0	0.780735	0.565727	0.981591	Biso	1.000000	H
H1005	1.0	0.810433	0.546417	0.007242	Biso	1.000000	H
O1696	1.0	0.606244	0.857514	0.133131	Biso	1.000000	O
H1006	1.0	0.595095	0.854521	0.157065	Biso	1.000000	H
H1007	1.0	0.613880	0.883991	0.133548	Biso	1.000000	H
H1008	1.0	0.628926	0.842912	0.134040	Biso	1.000000	H
O1697	1.0	0.436995	0.659824	0.878420	Biso	1.000000	O
H1009	1.0	0.461033	0.662059	0.867002	Biso	1.000000	H
H1010	1.0	0.426621	0.685273	0.877504	Biso	1.000000	H
H1011	1.0	0.442478	0.653772	0.903718	Biso	1.000000	H
O1698	1.0	0.975199	0.823806	0.201334	Biso	1.000000	O
H1012	1.0	0.975427	0.797306	0.208834	Biso	1.000000	H
H1013	1.0	0.956111	0.825033	0.182799	Biso	1.000000	H
H1014	1.0	0.966269	0.837488	0.222605	Biso	1.000000	H
O1699	1.0	0.266967	0.153837	0.813002	Biso	1.000000	O
H1015	1.0	0.274453	0.176318	0.799488	Biso	1.000000	H
H1016	1.0	0.261030	0.163514	0.837136	Biso	1.000000	H
H1017	1.0	0.245234	0.142836	0.801723	Biso	1.000000	H
O1700	1.0	0.082706	0.490997	0.667681	Biso	1.000000	O
H1018	1.0	0.093571	0.467543	0.676655	Biso	1.000000	H
H1019	1.0	0.066425	0.500538	0.686623	Biso	1.000000	H
H1020	1.0	0.065541	0.484360	0.648369	Biso	1.000000	H
O1701	1.0	0.003576	0.558573	0.972350	Biso	1.000000	O
H1021	1.0	0.980879	0.566786	0.983945	Biso	1.000000	H
H1022	1.0	0.007659	0.579687	0.955731	Biso	1.000000	H
H1023	1.0	0.022129	0.562918	0.990987	Biso	1.000000	H
O1702	1.0	0.715114	0.059408	0.282380	Biso	1.000000	O
H1024	1.0	0.728115	0.073186	0.263347	Biso	1.000000	H
H1025	1.0	0.692687	0.049825	0.271244	Biso	1.000000	H
H1026	1.0	0.730146	0.037044	0.286877	Biso	1.000000	H
O1703	1.0	0.654678	0.796079	0.641149	Biso	1.000000	O
H1027	1.0	0.679029	0.785186	0.644442	Biso	1.000000	H
H1028	1.0	0.642870	0.778513	0.624361	Biso	1.000000	H
H1029	1.0	0.657745	0.818453	0.625887	Biso	1.000000	H
O1704	1.0	0.605653	0.419126	0.451930	Biso	1.000000	O
H1030	1.0	0.620388	0.407546	0.471067	Biso	1.000000	H
H1031	1.0	0.582275	0.405720	0.452298	Biso	1.000000	H
H1032	1.0	0.617643	0.414335	0.428676	Biso	1.000000	H

Na1	1.0	0.496898	0.857843	0.417250	Biso	1.000000	Na
Na2	1.0	0.249326	0.367026	0.327837	Biso	1.000000	Na
Na3	1.0	0.018084	0.787324	0.930798	Biso	1.000000	Na
Na4	1.0	0.532964	0.335959	0.193266	Biso	1.000000	Na
Na5	1.0	0.718046	0.466010	0.044812	Biso	1.000000	Na
Na6	1.0	0.346828	0.489758	0.778614	Biso	1.000000	Na
Na7	1.0	0.054816	0.719804	0.471168	Biso	1.000000	Na
Na8	1.0	0.921192	0.928933	0.506472	Biso	1.000000	Na
Na9	1.0	0.433373	0.645000	0.190898	Biso	1.000000	Na
Na10	1.0	0.546300	0.856551	0.591167	Biso	1.000000	Na
Na11	1.0	0.075796	0.396488	0.990690	Biso	1.000000	Na
Na12	1.0	0.593853	0.147898	0.215062	Biso	1.000000	Na
Na13	1.0	0.018183	0.520430	0.251596	Biso	1.000000	Na
Na14	1.0	0.271167	0.654286	0.257967	Biso	1.000000	Na
Na15	1.0	0.368459	0.008449	0.525716	Biso	1.000000	Na
Na16	1.0	0.174424	0.346617	0.119345	Biso	1.000000	Na
Na17	1.0	0.303920	0.972984	0.175213	Biso	1.000000	Na
Na18	1.0	0.120785	0.924287	0.663244	Biso	1.000000	Na
Na19	1.0	0.817735	0.271496	0.433100	Biso	1.000000	Na
Na20	1.0	0.439389	0.646531	0.693988	Biso	1.000000	Na
Na21	1.0	0.400773	0.960227	0.805795	Biso	1.000000	Na
Na22	1.0	0.430842	0.175363	0.806430	Biso	1.000000	Na
Na23	1.0	0.402040	0.291534	0.382445	Biso	1.000000	Na
Na24	1.0	0.416860	0.228269	0.957506	Biso	1.000000	Na
Na25	1.0	0.193319	0.849730	0.300882	Biso	1.000000	Na
Na26	1.0	0.052044	0.132745	0.434447	Biso	1.000000	Na
Na27	1.0	0.037661	0.325884	0.271357	Biso	1.000000	Na
Na28	1.0	0.322318	0.758890	0.781743	Biso	1.000000	Na
H1033	1.0	0.027235	0.840816	0.644000	Biso	1.000000	H
H1034	1.0	0.994964	0.773325	0.621106	Biso	1.000000	H
H1035	1.0	0.153601	0.810144	0.556691	Biso	1.000000	H
H1036	1.0	0.228715	0.694962	0.620133	Biso	1.000000	H
H1037	1.0	0.104434	0.667352	0.562034	Biso	1.000000	H
H1038	1.0	0.050968	0.793822	0.507353	Biso	1.000000	H
H1039	1.0	0.238373	0.771990	0.472229	Biso	1.000000	H
O1705	1.0	0.046607	0.051821	0.022621	Biso	1.000000	O
H1040	1.0	0.052821	0.076846	0.011307	Biso	1.000000	H
H1041	1.0	0.026033	0.039737	0.008765	Biso	1.000000	H
O1706	1.0	0.670708	0.935609	0.030413	Biso	1.000000	O
H1042	1.0	0.696389	0.940462	0.021904	Biso	1.000000	H
H1043	1.0	0.671277	0.918764	0.052351	Biso	1.000000	H
O1707	1.0	0.807856	0.538093	0.401544	Biso	1.000000	O
H1044	1.0	0.781749	0.546942	0.401045	Biso	1.000000	H
H1045	1.0	0.823893	0.557511	0.389608	Biso	1.000000	H
O1708	1.0	0.794313	0.323723	0.294916	Biso	1.000000	O
H1046	1.0	0.781323	0.321363	0.271001	Biso	1.000000	H
H1047	1.0	0.777363	0.315962	0.315034	Biso	1.000000	H
O1709	1.0	0.638663	0.121526	0.316146	Biso	1.000000	O
H1048	1.0	0.644390	0.094354	0.321663	Biso	1.000000	H
H1049	1.0	0.635313	0.135804	0.339473	Biso	1.000000	H
O1710	1.0	0.148243	0.134625	0.490993	Biso	1.000000	O
H1050	1.0	0.166967	0.114863	0.484811	Biso	1.000000	H
H1051	1.0	0.157641	0.150821	0.511351	Biso	1.000000	H
O1711	1.0	0.087513	0.720262	0.860531	Biso	1.000000	O
H1052	1.0	0.098985	0.739157	0.843659	Biso	1.000000	H
H1053	1.0	0.060179	0.723104	0.859852	Biso	1.000000	H
O1712	1.0	0.836296	0.516753	0.697032	Biso	1.000000	O
H1054	1.0	0.852990	0.538746	0.701846	Biso	1.000000	H
H1055	1.0	0.826754	0.518326	0.671514	Biso	1.000000	H
O1713	1.0	0.335880	0.658251	0.255434	Biso	1.000000	O
H1056	1.0	0.346757	0.637654	0.270787	Biso	1.000000	H
H1057	1.0	0.350178	0.682221	0.258968	Biso	1.000000	H
O1714	1.0	0.254751	0.359344	0.738197	Biso	1.000000	O
H1058	1.0	0.243694	0.379035	0.721905	Biso	1.000000	H
H1059	1.0	0.270464	0.371987	0.756982	Biso	1.000000	H
O1715	1.0	0.542098	0.170860	0.757735	Biso	1.000000	O
H1060	1.0	0.552708	0.160052	0.734833	Biso	1.000000	H
H1061	1.0	0.554824	0.159494	0.779290	Biso	1.000000	H
O1716	1.0	0.472157	0.280606	0.796212	Biso	1.000000	O
H1062	1.0	0.449411	0.286182	0.810541	Biso	1.000000	H
H1063	1.0	0.465323	0.274326	0.770508	Biso	1.000000	H
O1717	1.0	0.818535	0.518678	0.767640	Biso	1.000000	O
H1064	1.0	0.797234	0.501299	0.771917	Biso	1.000000	H

H1065	1.0	0.824215	0.519593	0.740985	Biso	1.000000	H
O1718	1.0	0.900339	0.384677	0.884785	Biso	1.000000	O
H1066	1.0	0.888635	0.398950	0.905293	Biso	1.000000	H
H1067	1.0	0.926578	0.392708	0.882294	Biso	1.000000	H
O1719	1.0	0.197065	0.206093	0.435087	Biso	1.000000	O
H1068	1.0	0.199661	0.196442	0.409583	Biso	1.000000	H
H1069	1.0	0.211983	0.189936	0.451924	Biso	1.000000	H
O1720	1.0	0.798842	0.895688	0.725522	Biso	1.000000	O
H1070	1.0	0.772408	0.901535	0.720615	Biso	1.000000	H
H1071	1.0	0.801532	0.885863	0.750956	Biso	1.000000	H
O1721	1.0	0.246911	0.582827	0.933838	Biso	1.000000	O
H1072	1.0	0.255812	0.578068	0.908458	Biso	1.000000	H
H1073	1.0	0.243919	0.558059	0.946839	Biso	1.000000	H
O1722	1.0	0.949216	0.061379	0.883727	Biso	1.000000	O
H1074	1.0	0.927062	0.074709	0.893545	Biso	1.000000	H
H1075	1.0	0.968688	0.060410	0.902938	Biso	1.000000	H
O1723	1.0	0.499965	0.785419	0.718031	Biso	1.000000	O
H1076	1.0	0.472550	0.786813	0.716707	Biso	1.000000	H
H1077	1.0	0.507313	0.766021	0.736556	Biso	1.000000	H
O1724	1.0	0.364007	0.233898	0.354427	Biso	1.000000	O
H1078	1.0	0.352830	0.236574	0.379196	Biso	1.000000	H
H1079	1.0	0.362611	0.206763	0.346511	Biso	1.000000	H
O1725	1.0	0.314267	0.153754	0.378111	Biso	1.000000	O
H1080	1.0	0.293854	0.137902	0.368034	Biso	1.000000	H
H1081	1.0	0.334819	0.154906	0.360050	Biso	1.000000	H
O1726	1.0	0.857629	0.472391	0.540214	Biso	1.000000	O
H1082	1.0	0.837356	0.477712	0.522535	Biso	1.000000	H
H1083	1.0	0.862836	0.495750	0.554824	Biso	1.000000	H
O1727	1.0	0.830090	0.204132	0.438724	Biso	1.000000	O
H1084	1.0	0.811305	0.183467	0.440239	Biso	1.000000	H
H1085	1.0	0.851091	0.198073	0.455315	Biso	1.000000	H
O1728	1.0	0.824927	0.239194	0.672838	Biso	1.000000	O
H1086	1.0	0.797737	0.235155	0.673645	Biso	1.000000	H
H1087	1.0	0.836787	0.217626	0.659572	Biso	1.000000	H
O1729	1.0	0.026911	0.598034	0.761611	Biso	1.000000	O
H1088	1.0	0.051526	0.586414	0.756873	Biso	1.000000	H
H1089	1.0	0.029988	0.621414	0.776777	Biso	1.000000	H
O1730	1.0	0.510495	0.484423	0.754837	Biso	1.000000	O
H1090	1.0	0.517283	0.510929	0.761930	Biso	1.000000	H
H1091	1.0	0.503974	0.483752	0.728365	Biso	1.000000	H
O1731	1.0	0.053648	0.312571	0.392811	Biso	1.000000	O
H1092	1.0	0.038788	0.317316	0.415285	Biso	1.000000	H
H1093	1.0	0.070835	0.290887	0.397191	Biso	1.000000	H
O1732	1.0	0.962990	0.600783	0.883994	Biso	1.000000	O
H1094	1.0	0.979361	0.608865	0.904465	Biso	1.000000	H
H1095	1.0	0.961859	0.621399	0.865285	Biso	1.000000	H
O1733	1.0	0.219617	0.121875	0.413619	Biso	1.000000	O
H1096	1.0	0.208040	0.141537	0.397652	Biso	1.000000	H
H1097	1.0	0.229176	0.134187	0.436279	Biso	1.000000	H
O1734	1.0	0.730154	0.657932	0.567551	Biso	1.000000	O
H1098	1.0	0.746766	0.680508	0.568895	Biso	1.000000	H
H1099	1.0	0.741284	0.638333	0.551204	Biso	1.000000	H
O1735	1.0	0.004962	0.534246	0.889318	Biso	1.000000	O
H1100	1.0	0.990770	0.557866	0.883771	Biso	1.000000	H
H1101	1.0	0.029833	0.535895	0.877831	Biso	1.000000	H
O1736	1.0	0.387783	0.222115	0.783174	Biso	1.000000	O
H1102	1.0	0.370165	0.216778	0.762895	Biso	1.000000	H
H1103	1.0	0.374282	0.233843	0.804078	Biso	1.000000	H
O1737	1.0	0.726903	0.780333	0.121818	Biso	1.000000	O
H1104	1.0	0.746544	0.794651	0.135031	Biso	1.000000	H
H1105	1.0	0.730803	0.752447	0.125214	Biso	1.000000	H
O1738	1.0	0.539820	0.745646	0.555890	Biso	1.000000	O
H1106	1.0	0.550153	0.729661	0.575954	Biso	1.000000	H
H1107	1.0	0.548498	0.772416	0.558798	Biso	1.000000	H
O1739	1.0	0.007951	0.700841	0.430999	Biso	1.000000	O
H1108	1.0	0.986524	0.698523	0.447920	Biso	1.000000	H
H1109	1.0	0.999317	0.698063	0.405260	Biso	1.000000	H
O1740	1.0	0.742735	0.524625	0.026152	Biso	1.000000	O
H1110	1.0	0.737970	0.542702	0.005669	Biso	1.000000	H
H1111	1.0	0.755247	0.538346	0.046535	Biso	1.000000	H
O1741	1.0	0.022415	0.157878	0.383707	Biso	1.000000	O
H1112	1.0	0.018124	0.185906	0.384740	Biso	1.000000	H
H1113	1.0	0.007382	0.146636	0.363602	Biso	1.000000	H
O1742	1.0	0.373397	0.599113	0.787287	Biso	1.000000	O
H1114	1.0	0.370140	0.577423	0.769995	Biso	1.000000	H
H1115	1.0	0.358634	0.594371	0.809824	Biso	1.000000	H
O1743	1.0	0.414750	0.677146	0.093740	Biso	1.000000	O
H1116	1.0	0.397376	0.671697	0.073278	Biso	1.000000	H
H1117	1.0	0.434835	0.694193	0.084884	Biso	1.000000	H
O1744	1.0	0.182126	0.277686	0.448975	Biso	1.000000	O
H1118	1.0	0.189334	0.250863	0.443589	Biso	1.000000	H
H1119	1.0	0.189833	0.284393	0.474333	Biso	1.000000	H
O1745	1.0	0.142236	0.202476	0.174925	Biso	1.000000	O

H1120	1.0	0.150029	0.200498	0.200995	Biso	1.000000	H
H1121	1.0	0.163969	0.198261	0.158741	Biso	1.000000	H
O1746	1.0	0.908421	0.489713	0.942508	Biso	1.000000	O
H1122	1.0	0.906017	0.511034	0.924663	Biso	1.000000	H
H1123	1.0	0.919452	0.499462	0.965653	Biso	1.000000	H
O1747	1.0	0.249700	0.940434	0.161974	Biso	1.000000	O
H1124	1.0	0.238277	0.935226	0.137692	Biso	1.000000	H
H1125	1.0	0.230732	0.937608	0.181510	Biso	1.000000	H
O1748	1.0	0.768055	0.429076	0.835909	Biso	1.000000	O
H1126	1.0	0.743496	0.416346	0.835416	Biso	1.000000	H
H1127	1.0	0.767088	0.451482	0.852629	Biso	1.000000	H
O1749	1.0	0.554976	0.766359	0.672274	Biso	1.000000	O
H1128	1.0	0.576441	0.755727	0.685895	Biso	1.000000	H
H1129	1.0	0.535200	0.773523	0.689907	Biso	1.000000	H
O1750	1.0	0.301340	0.886885	0.727912	Biso	1.000000	O
H1130	1.0	0.294181	0.907672	0.710751	Biso	1.000000	H
H1131	1.0	0.282612	0.884852	0.747764	Biso	1.000000	H
O1751	1.0	0.358601	0.884457	0.153338	Biso	1.000000	O
H1132	1.0	0.377898	0.873081	0.137295	Biso	1.000000	H
H1133	1.0	0.335636	0.868955	0.151727	Biso	1.000000	H
O1752	1.0	0.096347	0.766061	0.020812	Biso	1.000000	O
H1134	1.0	0.117234	0.759634	0.004206	Biso	1.000000	H
H1135	1.0	0.080553	0.743118	0.024360	Biso	1.000000	H
O1753	1.0	0.356507	0.611838	0.131221	Biso	1.000000	O
H1136	1.0	0.345707	0.586085	0.127083	Biso	1.000000	H
H1137	1.0	0.383927	0.609950	0.131467	Biso	1.000000	H
O1754	1.0	0.999786	0.712327	0.717328	Biso	1.000000	O
H1138	1.0	0.994592	0.684563	0.714823	Biso	1.000000	H
H1139	1.0	0.012786	0.717104	0.740901	Biso	1.000000	H
O1755	1.0	0.058841	0.594064	0.617307	Biso	1.000000	O
H1140	1.0	0.072300	0.570367	0.610427	Biso	1.000000	H
H1141	1.0	0.062897	0.613886	0.598205	Biso	1.000000	H
O1756	1.0	0.929488	0.446262	0.773702	Biso	1.000000	O
H1142	1.0	0.927974	0.462719	0.795865	Biso	1.000000	H
H1143	1.0	0.907308	0.450513	0.758130	Biso	1.000000	H
O1757	1.0	0.039878	0.393620	0.045265	Biso	1.000000	O
H1144	1.0	0.056071	0.408673	0.061885	Biso	1.000000	H
H1145	1.0	0.016648	0.386838	0.058292	Biso	1.000000	H
O1758	1.0	0.054260	0.398308	0.602822	Biso	1.000000	O
H1146	1.0	0.053049	0.409590	0.627806	Biso	1.000000	H
H1147	1.0	0.072675	0.412515	0.587877	Biso	1.000000	H
O1759	1.0	0.439337	0.852800	0.746903	Biso	1.000000	O
H1148	1.0	0.434519	0.826936	0.736717	Biso	1.000000	H
H1149	1.0	0.454176	0.850480	0.769736	Biso	1.000000	H
O1760	1.0	0.789108	0.846155	0.220307	Biso	1.000000	O
H1150	1.0	0.796341	0.826930	0.239040	Biso	1.000000	H
H1151	1.0	0.770732	0.864079	0.231016	Biso	1.000000	H
O1761	1.0	0.467425	0.274750	0.105814	Biso	1.000000	O
H1152	1.0	0.471956	0.246782	0.104380	Biso	1.000000	H
H1153	1.0	0.451055	0.280346	0.127038	Biso	1.000000	H
O1762	1.0	0.165291	0.851386	0.726274	Biso	1.000000	O
H1154	1.0	0.166674	0.867961	0.704185	Biso	1.000000	H
H1155	1.0	0.177805	0.826669	0.721172	Biso	1.000000	H
O1763	1.0	0.364163	0.012491	0.779590	Biso	1.000000	O
H1156	1.0	0.351486	0.002301	0.757470	Biso	1.000000	H
H1157	1.0	0.371731	0.039387	0.775101	Biso	1.000000	H
O1764	1.0	0.924261	0.647866	0.762099	Biso	1.000000	O
H1158	1.0	0.943630	0.640925	0.743945	Biso	1.000000	H
H1159	1.0	0.928337	0.674502	0.770639	Biso	1.000000	H
O1765	1.0	0.557809	0.361230	0.844373	Biso	1.000000	O
H1160	1.0	0.540015	0.368741	0.863854	Biso	1.000000	H
H1161	1.0	0.579035	0.379262	0.844327	Biso	1.000000	H
O1766	1.0	0.208432	0.651472	0.034927	Biso	1.000000	O
H1162	1.0	0.231527	0.641235	0.045957	Biso	1.000000	H
H1163	1.0	0.205283	0.640817	0.009854	Biso	1.000000	H
O1767	1.0	0.716771	0.113113	0.682657	Biso	1.000000	O
H1164	1.0	0.737810	0.119853	0.698954	Biso	1.000000	H
H1165	1.0	0.704097	0.090032	0.692358	Biso	1.000000	H
O1768	1.0	0.664885	0.613218	0.332550	Biso	1.000000	O
H1166	1.0	0.683877	0.625298	0.348479	Biso	1.000000	H
H1167	1.0	0.673827	0.587786	0.324286	Biso	1.000000	H
O1769	1.0	0.410284	0.924810	0.752624	Biso	1.000000	O
H1168	1.0	0.421829	0.899173	0.750116	Biso	1.000000	H
H1169	1.0	0.401303	0.933562	0.728269	Biso	1.000000	H
O1770	1.0	0.318636	0.600136	0.691684	Biso	1.000000	O
H1170	1.0	0.310820	0.574777	0.701192	Biso	1.000000	H
H1171	1.0	0.315558	0.600726	0.664604	Biso	1.000000	H
O1771	1.0	0.928293	0.712509	0.177773	Biso	1.000000	O
H1172	1.0	0.928548	0.710544	0.150582	Biso	1.000000	H
H1173	1.0	0.902972	0.719202	0.186201	Biso	1.000000	H
O1772	1.0	0.374383	0.619423	0.203734	Biso	1.000000	O
H1174	1.0	0.362690	0.617361	0.179147	Biso	1.000000	H

H1175	1.0	0.357889	0.634079	0.220391	Biso	1.000000	H
O1773	1.0	0.820640	0.347108	0.370026	Biso	1.000000	O
H1176	1.0	0.819233	0.318816	0.368124	Biso	1.000000	H
H1177	1.0	0.801516	0.358796	0.353988	Biso	1.000000	H
O1774	1.0	0.493611	0.950799	0.812850	Biso	1.000000	O
H1178	1.0	0.481643	0.972344	0.799643	Biso	1.000000	H
H1179	1.0	0.475040	0.930455	0.817579	Biso	1.000000	H
O1775	1.0	0.411142	0.724484	0.859722	Biso	1.000000	O
H1180	1.0	0.383955	0.720545	0.860962	Biso	1.000000	H
H1181	1.0	0.416443	0.749957	0.848881	Biso	1.000000	H
O1776	1.0	0.573269	0.258258	0.595340	Biso	1.000000	O
H1182	1.0	0.580807	0.231600	0.601023	Biso	1.000000	H
H1183	1.0	0.585787	0.266841	0.572517	Biso	1.000000	H
O1777	1.0	0.065787	0.380741	0.362441	Biso	1.000000	O
H1184	1.0	0.088236	0.392515	0.373371	Biso	1.000000	H
H1185	1.0	0.061392	0.355357	0.373856	Biso	1.000000	H
O1778	1.0	0.845480	0.770877	0.154868	Biso	1.000000	O
H1186	1.0	0.851580	0.751987	0.135437	Biso	1.000000	H
H1187	1.0	0.854894	0.761562	0.178864	Biso	1.000000	H
O1779	1.0	0.383449	0.329996	0.731775	Biso	1.000000	O
H1188	1.0	0.410677	0.329016	0.728204	Biso	1.000000	H
H1189	1.0	0.372100	0.345671	0.712029	Biso	1.000000	H
O1780	1.0	0.514013	0.223220	0.894668	Biso	1.000000	O
H1190	1.0	0.539053	0.211541	0.893979	Biso	1.000000	H
H1191	1.0	0.516255	0.251470	0.892998	Biso	1.000000	H
O1781	1.0	0.545393	0.694943	0.217615	Biso	1.000000	O
H1192	1.0	0.562662	0.716967	0.216003	Biso	1.000000	H
H1193	1.0	0.539468	0.689777	0.243765	Biso	1.000000	H
O1782	1.0	0.572497	0.739171	0.349741	Biso	1.000000	O
H1194	1.0	0.589931	0.750502	0.367789	Biso	1.000000	H
H1195	1.0	0.563036	0.714446	0.359314	Biso	1.000000	H
O1783	1.0	0.850357	0.048358	0.382363	Biso	1.000000	O
H1196	1.0	0.858695	0.036765	0.405832	Biso	1.000000	H
H1197	1.0	0.839313	0.073852	0.387275	Biso	1.000000	H
O1784	1.0	0.870803	0.848884	0.117744	Biso	1.000000	O
H1198	1.0	0.864980	0.857963	0.142917	Biso	1.000000	H
H1199	1.0	0.847477	0.842361	0.104765	Biso	1.000000	H
O1785	1.0	0.376073	0.376113	0.790986	Biso	1.000000	O
H1200	1.0	0.378511	0.357430	0.770603	Biso	1.000000	H
H1201	1.0	0.397000	0.372833	0.808368	Biso	1.000000	H
O1786	1.0	0.162234	0.291708	0.205597	Biso	1.000000	O
H1202	1.0	0.186914	0.296564	0.216643	Biso	1.000000	H
H1203	1.0	0.144980	0.282442	0.224858	Biso	1.000000	H
O1787	1.0	0.192865	0.276475	0.373469	Biso	1.000000	O
H1204	1.0	0.187328	0.282489	0.399535	Biso	1.000000	H
H1205	1.0	0.216495	0.262062	0.371970	Biso	1.000000	H
O1788	1.0	0.827581	0.052247	0.178247	Biso	1.000000	O
H1206	1.0	0.837518	0.073327	0.162872	Biso	1.000000	H
H1207	1.0	0.836771	0.055090	0.203790	Biso	1.000000	H
O1789	1.0	0.593847	0.871460	0.759825	Biso	1.000000	O
H1208	1.0	0.568708	0.863349	0.752046	Biso	1.000000	H
H1209	1.0	0.607348	0.849179	0.770137	Biso	1.000000	H
O1790	1.0	0.567702	0.138100	0.820675	Biso	1.000000	O
H1210	1.0	0.595092	0.138948	0.822741	Biso	1.000000	H
H1211	1.0	0.556627	0.137226	0.845608	Biso	1.000000	H
O1791	1.0	0.419619	0.214350	0.484252	Biso	1.000000	O
H1212	1.0	0.446608	0.211286	0.488447	Biso	1.000000	H
H1213	1.0	0.408440	0.228207	0.505300	Biso	1.000000	H
O1792	1.0	0.553162	0.443607	0.390797	Biso	1.000000	O
H1214	1.0	0.532002	0.450130	0.407023	Biso	1.000000	H
H1215	1.0	0.549158	0.417902	0.379918	Biso	1.000000	H
O1793	1.0	0.085886	0.946701	0.378065	Biso	1.000000	O
H1216	1.0	0.066552	0.926985	0.373930	Biso	1.000000	H
H1217	1.0	0.076066	0.972207	0.371084	Biso	1.000000	H
O1794	1.0	0.916295	0.313085	0.915951	Biso	1.000000	O
H1218	1.0	0.903456	0.334994	0.904181	Biso	1.000000	H
H1219	1.0	0.943338	0.318097	0.916564	Biso	1.000000	H
O1795	1.0	0.273835	0.526002	0.692001	Biso	1.000000	O
H1220	1.0	0.254596	0.545295	0.686009	Biso	1.000000	H
H1221	1.0	0.261788	0.501239	0.697932	Biso	1.000000	H
O1796	1.0	0.303065	0.396417	0.783995	Biso	1.000000	O
H1222	1.0	0.305143	0.418496	0.766981	Biso	1.000000	H
H1223	1.0	0.328158	0.386429	0.789623	Biso	1.000000	H
O1797	1.0	0.683467	0.898786	0.303957	Biso	1.000000	O
H1224	1.0	0.679427	0.912340	0.327573	Biso	1.000000	H

H1225	1.0	0.673384	0.872434	0.305710	Biso	1.000000	H
O1798	1.0	0.668435	0.908612	0.811397	Biso	1.000000	O
H1226	1.0	0.660139	0.916765	0.786617	Biso	1.000000	H
H1227	1.0	0.654599	0.923160	0.830360	Biso	1.000000	H
O1799	1.0	0.183936	0.942109	0.204225	Biso	1.000000	O
H1228	1.0	0.194326	0.957872	0.224420	Biso	1.000000	H
H1229	1.0	0.176583	0.958980	0.183550	Biso	1.000000	H
O1800	1.0	0.552923	0.889270	0.519248	Biso	1.000000	O
H1230	1.0	0.562217	0.878843	0.495629	Biso	1.000000	H
H1231	1.0	0.562582	0.915600	0.522764	Biso	1.000000	H
O1801	1.0	0.267400	0.943557	0.901374	Biso	1.000000	O
H1232	1.0	0.268284	0.918767	0.914640	Biso	1.000000	H
H1233	1.0	0.273233	0.939335	0.875048	Biso	1.000000	H
O1802	1.0	0.313019	0.452092	0.733735	Biso	1.000000	O
H1234	1.0	0.287657	0.457411	0.724565	Biso	1.000000	H
H1235	1.0	0.329900	0.447966	0.712594	Biso	1.000000	H
O1803	1.0	0.424810	0.457499	0.708082	Biso	1.000000	O
H1236	1.0	0.418306	0.454574	0.734416	Biso	1.000000	H
H1237	1.0	0.441379	0.436250	0.700532	Biso	1.000000	H
O1804	1.0	0.925729	0.303018	0.374693	Biso	1.000000	O
H1238	1.0	0.943959	0.291659	0.391928	Biso	1.000000	H
H1239	1.0	0.935487	0.327616	0.365106	Biso	1.000000	H
H1240	1.0	0.444355	0.759898	0.546939	Biso	1.000000	H
H1241	1.0	0.443352	0.643342	0.582275	Biso	1.000000	H
O1805	1.0	0.211343	0.911673	0.284356	Biso	1.000000	O
H1242	1.0	0.238189	0.910497	0.290072	Biso	1.000000	H
H1243	1.0	0.204652	0.938114	0.276956	Biso	1.000000	H
O1806	1.0	0.414895	0.681772	0.645120	Biso	1.000000	O
H1244	1.0	0.428707	0.674934	0.622488	Biso	1.000000	H
H1245	1.0	0.419647	0.708979	0.651343	Biso	1.000000	H
O1807	1.0	0.908056	0.639105	0.459687	Biso	1.000000	O
H1246	1.0	0.932651	0.629072	0.467110	Biso	1.000000	H
H1247	1.0	0.911417	0.661315	0.443030	Biso	1.000000	H
O1808	1.0	0.878319	0.081952	0.689092	Biso	1.000000	O
H1248	1.0	0.882568	0.060968	0.706968	Biso	1.000000	H
H1249	1.0	0.900908	0.098017	0.687331	Biso	1.000000	H
O1809	1.0	0.919906	0.336729	0.821065	Biso	1.000000	O
H1250	1.0	0.929812	0.350084	0.799109	Biso	1.000000	H
H1251	1.0	0.915347	0.355621	0.840906	Biso	1.000000	H
O1810	1.0	0.489967	0.363516	0.791619	Biso	1.000000	O
H1252	1.0	0.508223	0.384473	0.788380	Biso	1.000000	H
H1253	1.0	0.470813	0.371521	0.809590	Biso	1.000000	H
O1811	1.0	0.480802	0.794307	0.882092	Biso	1.000000	O
H1254	1.0	0.476221	0.788225	0.908327	Biso	1.000000	H
H1255	1.0	0.457306	0.792021	0.868128	Biso	1.000000	H
O1812	1.0	0.459714	0.338685	0.729286	Biso	1.000000	O
H1256	1.0	0.471055	0.345336	0.753281	Biso	1.000000	H
H1257	1.0	0.461085	0.361099	0.712605	Biso	1.000000	H
O1813	1.0	0.131617	0.098943	0.960839	Biso	1.000000	O
H1258	1.0	0.123148	0.088065	0.937103	Biso	1.000000	H
H1259	1.0	0.157574	0.107906	0.958376	Biso	1.000000	H
O1814	1.0	0.880339	0.531837	0.325084	Biso	1.000000	O
H1260	1.0	0.864425	0.508813	0.327420	Biso	1.000000	H
H1261	1.0	0.888421	0.534691	0.299176	Biso	1.000000	H
O1815	1.0	0.741822	0.317313	0.499846	Biso	1.000000	O
H1262	1.0	0.714372	0.318148	0.498903	Biso	1.000000	H
H1263	1.0	0.751210	0.340500	0.512534	Biso	1.000000	H
O1816	1.0	0.071806	0.612602	0.897495	Biso	1.000000	O
H1264	1.0	0.077852	0.612923	0.870908	Biso	1.000000	H
H1265	1.0	0.088026	0.593908	0.910230	Biso	1.000000	H
O1817	1.0	0.079264	0.277418	0.183603	Biso	1.000000	O
H1266	1.0	0.057475	0.260521	0.187194	Biso	1.000000	H
H1267	1.0	0.101887	0.261547	0.180885	Biso	1.000000	H
O1818	1.0	0.050572	0.489753	0.153660	Biso	1.000000	O
H1268	1.0	0.040342	0.463551	0.150949	Biso	1.000000	H
H1269	1.0	0.059590	0.493388	0.179171	Biso	1.000000	H
O1819	1.0	0.039982	0.423121	0.673054	Biso	1.000000	O
H1270	1.0	0.046862	0.407949	0.695062	Biso	1.000000	H
H1271	1.0	0.014415	0.433075	0.675987	Biso	1.000000	H

O1820	1.0	0.522263	0.913953	0.442354	Biso	1.000000	O
H1272	1.0	0.546223	0.900419	0.445415	Biso	1.000000	H
H1273	1.0	0.522664	0.938091	0.456708	Biso	1.000000	H
O1821	1.0	0.293986	0.801284	0.738265	Biso	1.000000	O
H1274	1.0	0.280553	0.819936	0.753913	Biso	1.000000	H
H1275	1.0	0.284739	0.803085	0.712654	Biso	1.000000	H
O1822	1.0	0.356952	0.425362	0.306783	Biso	1.000000	O
H1276	1.0	0.381597	0.433904	0.315620	Biso	1.000000	H
H1277	1.0	0.346339	0.445126	0.290285	Biso	1.000000	H
O1823	1.0	0.188766	0.584222	0.120229	Biso	1.000000	O
H1278	1.0	0.161306	0.583827	0.119258	Biso	1.000000	H
H1279	1.0	0.198385	0.557767	0.117545	Biso	1.000000	H
O1824	1.0	0.205835	0.315760	0.310943	Biso	1.000000	O
H1280	1.0	0.201241	0.303535	0.335118	Biso	1.000000	H
H1281	1.0	0.210608	0.295695	0.292244	Biso	1.000000	H
O1825	1.0	0.048099	0.342831	0.208754	Biso	1.000000	O
H1282	1.0	0.026748	0.348442	0.192461	Biso	1.000000	H
H1283	1.0	0.060997	0.319506	0.199922	Biso	1.000000	H
O1826	1.0	0.146160	0.583222	0.570898	Biso	1.000000	O
H1284	1.0	0.155942	0.593641	0.594326	Biso	1.000000	H
H1285	1.0	0.127463	0.563162	0.576227	Biso	1.000000	H
O1827	1.0	0.954331	0.472328	0.574512	Biso	1.000000	O
H1286	1.0	0.945383	0.480728	0.550035	Biso	1.000000	H
H1287	1.0	0.961893	0.445056	0.573286	Biso	1.000000	H
O1828	1.0	0.511768	0.925474	0.728256	Biso	1.000000	O
H1288	1.0	0.534838	0.940834	0.729670	Biso	1.000000	H
H1289	1.0	0.490657	0.942453	0.722022	Biso	1.000000	H
O1829	1.0	0.321799	0.991061	0.722469	Biso	1.000000	O
H1290	1.0	0.321565	0.970651	0.703517	Biso	1.000000	H
H1291	1.0	0.296080	0.996314	0.730635	Biso	1.000000	H
O1830	1.0	0.492204	0.776746	0.628030	Biso	1.000000	O
H1292	1.0	0.469010	0.769079	0.640656	Biso	1.000000	H
H1293	1.0	0.513735	0.770672	0.643932	Biso	1.000000	H
O1831	1.0	0.433439	0.686622	0.239987	Biso	1.000000	O
H1294	1.0	0.411928	0.699392	0.251709	Biso	1.000000	H
H1295	1.0	0.449213	0.674800	0.259207	Biso	1.000000	H
O1832	1.0	0.543126	0.356485	0.256372	Biso	1.000000	O
H1296	1.0	0.523595	0.367153	0.272586	Biso	1.000000	H
H1297	1.0	0.563217	0.375599	0.253322	Biso	1.000000	H
O1833	1.0	0.095231	0.530954	0.590207	Biso	1.000000	O
H1298	1.0	0.075054	0.519266	0.575488	Biso	1.000000	H
H1299	1.0	0.111091	0.510466	0.600633	Biso	1.000000	H
O1834	1.0	0.903225	0.357530	0.458970	Biso	1.000000	O
H1300	1.0	0.879819	0.345478	0.450589	Biso	1.000000	H
H1301	1.0	0.920617	0.359625	0.437960	Biso	1.000000	H
O1835	1.0	0.899042	0.280975	0.308765	Biso	1.000000	O
H1302	1.0	0.906674	0.286911	0.334322	Biso	1.000000	H
H1303	1.0	0.886214	0.303461	0.298031	Biso	1.000000	H
O1836	1.0	0.731479	0.922930	0.702203	Biso	1.000000	O
H1304	1.0	0.731105	0.900584	0.685388	Biso	1.000000	H
H1305	1.0	0.718379	0.944730	0.690529	Biso	1.000000	H
O1837	1.0	0.435149	0.597123	0.320587	Biso	1.000000	O
H1306	1.0	0.419493	0.586868	0.340708	Biso	1.000000	H
H1307	1.0	0.459439	0.583838	0.320636	Biso	1.000000	H
O1838	1.0	0.502880	0.491079	0.677524	Biso	1.000000	O
H1308	1.0	0.483594	0.495029	0.658482	Biso	1.000000	H
H1309	1.0	0.513634	0.465095	0.674847	Biso	1.000000	H
O1839	1.0	0.378522	0.016938	0.648359	Biso	1.000000	O
H1310	1.0	0.379410	0.040878	0.662993	Biso	1.000000	H
H1311	1.0	0.401861	0.014167	0.634220	Biso	1.000000	H
O1840	1.0	0.063957	0.979082	0.162921	Biso	1.000000	O
H1312	1.0	0.068337	0.960039	0.143174	Biso	1.000000	H
H1313	1.0	0.080430	0.973452	0.184059	Biso	1.000000	H
O1841	1.0	0.001604	0.749278	0.980151	Biso	1.000000	O
H1314	1.0	0.984716	0.749266	0.001654	Biso	1.000000	H
H1315	1.0	0.013528	0.723805	0.977836	Biso	1.000000	H
O1842	1.0	0.048344	0.741835	0.569753	Biso	1.000000	O
H1316	1.0	0.028887	0.761494	0.573556	Biso	1.000000	H
H1317	1.0	0.048565	0.723950	0.590921	Biso	1.000000	H
O1843	1.0	0.158701	0.922109	0.376358	Biso	1.000000	O
H1318	1.0	0.175818	0.942998	0.369097	Biso	1.000000	H
H1319	1.0	0.132883	0.931838	0.376372	Biso	1.000000	H
O1844	1.0	0.183807	0.682675	0.380207	Biso	1.000000	O
H1320	1.0	0.200386	0.662314	0.370689	Biso	1.000000	H
H1321	1.0	0.189441	0.687437	0.406492	Biso	1.000000	H
O1845	1.0	0.134484	0.104267	0.069558	Biso	1.000000	O
H1322	1.0	0.154532	0.086621	0.077346	Biso	1.000000	H
H1323	1.0	0.144417	0.122903	0.051505	Biso	1.000000	H
O1846	1.0	0.284881	0.215849	0.783649	Biso	1.000000	O
H1324	1.0	0.263941	0.221170	0.766754	Biso	1.000000	H

H1325	1.0	0.308174	0.225808	0.772792	Biso	1.000000	H
O1847	1.0	0.872034	0.961405	0.340035	Biso	1.000000	O
H1326	1.0	0.879699	0.963518	0.366134	Biso	1.000000	H
H1327	1.0	0.891677	0.972237	0.324062	Biso	1.000000	H
O1848	1.0	0.540941	0.393205	0.460870	Biso	1.000000	O
H1328	1.0	0.533997	0.400696	0.486242	Biso	1.000000	H
H1329	1.0	0.518429	0.392435	0.445254	Biso	1.000000	H
O1849	1.0	0.478870	0.211784	0.829676	Biso	1.000000	O
H1330	1.0	0.481899	0.235795	0.815436	Biso	1.000000	H
H1331	1.0	0.493273	0.213551	0.852825	Biso	1.000000	H
O1850	1.0	0.565160	0.922453	0.810871	Biso	1.000000	O
H1332	1.0	0.539325	0.931932	0.808960	Biso	1.000000	H
H1333	1.0	0.571349	0.907254	0.788678	Biso	1.000000	H
O1851	1.0	0.234235	0.029877	0.159435	Biso	1.000000	O
H1334	1.0	0.210024	0.017283	0.163927	Biso	1.000000	H
H1335	1.0	0.240547	0.028016	0.132966	Biso	1.000000	H
O1852	1.0	0.101072	0.867325	0.169111	Biso	1.000000	O
H1336	1.0	0.100504	0.840481	0.160237	Biso	1.000000	H
H1337	1.0	0.123542	0.871375	0.184313	Biso	1.000000	H
O1853	1.0	0.015636	0.587195	0.258220	Biso	1.000000	O
H1338	1.0	0.035253	0.602941	0.246564	Biso	1.000000	H
H1339	1.0	0.994527	0.603841	0.265238	Biso	1.000000	H
O1854	1.0	0.799345	0.220597	0.261593	Biso	1.000000	O
H1340	1.0	0.788419	0.195167	0.256144	Biso	1.000000	H
H1341	1.0	0.815100	0.218761	0.283857	Biso	1.000000	H
O1855	1.0	0.626777	0.924168	0.148942	Biso	1.000000	O
H1342	1.0	0.652161	0.932687	0.142446	Biso	1.000000	H
H1343	1.0	0.614158	0.944563	0.163190	Biso	1.000000	H
O1856	1.0	0.930821	0.496142	0.829875	Biso	1.000000	O
H1344	1.0	0.946378	0.517880	0.821537	Biso	1.000000	H
H1345	1.0	0.940266	0.486493	0.853736	Biso	1.000000	H
O1857	1.0	0.165689	0.154767	0.020829	Biso	1.000000	O
H1346	1.0	0.177626	0.144380	0.998393	Biso	1.000000	H
H1347	1.0	0.140199	0.163366	0.014856	Biso	1.000000	H
O1858	1.0	0.130288	0.754262	0.250908	Biso	1.000000	O
H1348	1.0	0.108289	0.760191	0.235596	Biso	1.000000	H
H1349	1.0	0.150542	0.744581	0.235000	Biso	1.000000	H
O1859	1.0	0.166368	0.990505	0.152483	Biso	1.000000	O
H1350	1.0	0.148337	0.011930	0.152879	Biso	1.000000	H
H1351	1.0	0.173883	0.985229	0.126758	Biso	1.000000	H
O1860	1.0	0.210550	0.985060	0.257406	Biso	1.000000	O
H1352	1.0	0.194315	0.000549	0.273611	Biso	1.000000	H
H1353	1.0	0.236861	0.991013	0.262816	Biso	1.000000	H
O1861	1.0	0.959091	0.648808	0.207563	Biso	1.000000	O
H1354	1.0	0.946051	0.671691	0.197910	Biso	1.000000	H
H1355	1.0	0.956120	0.647650	0.234637	Biso	1.000000	H
O1862	1.0	0.455767	0.056663	0.181193	Biso	1.000000	O
H1356	1.0	0.478629	0.045206	0.191580	Biso	1.000000	H
H1357	1.0	0.462437	0.074528	0.161064	Biso	1.000000	H
O1863	1.0	0.731682	0.937512	0.598196	Biso	1.000000	O
H1358	1.0	0.722836	0.921476	0.577483	Biso	1.000000	H
H1359	1.0	0.739541	0.962882	0.588754	Biso	1.000000	H
O1864	1.0	0.015092	0.266193	0.120964	Biso	1.000000	O
H1360	1.0	0.024218	0.247553	0.102500	Biso	1.000000	H
H1361	1.0	0.016043	0.254448	0.145763	Biso	1.000000	H
O1865	1.0	0.789070	0.018415	0.929585	Biso	1.000000	O
H1362	1.0	0.772491	0.039665	0.937103	Biso	1.000000	H
H1363	1.0	0.799584	0.006007	0.951776	Biso	1.000000	H
O1866	1.0	0.360533	0.675107	0.582360	Biso	1.000000	O
H1364	1.0	0.376123	0.652883	0.575374	Biso	1.000000	H
H1365	1.0	0.348373	0.669925	0.606292	Biso	1.000000	H
O1867	1.0	0.921005	0.942024	0.617975	Biso	1.000000	O
H1366	1.0	0.912238	0.958219	0.638606	Biso	1.000000	H
H1367	1.0	0.905058	0.946360	0.596169	Biso	1.000000	H
O1868	1.0	0.535516	0.421963	0.681820	Biso	1.000000	O
H1368	1.0	0.553357	0.438436	0.695233	Biso	1.000000	H
H1369	1.0	0.545041	0.395340	0.681016	Biso	1.000000	H
O1869	1.0	0.965883	0.364081	0.348354	Biso	1.000000	O
H1370	1.0	0.961998	0.385046	0.330378	Biso	1.000000	H
H1371	1.0	0.984079	0.345535	0.338334	Biso	1.000000	H
O1870	1.0	0.438045	0.836224	0.435938	Biso	1.000000	O
H1372	1.0	0.426814	0.810627	0.439833	Biso	1.000000	H
H1373	1.0	0.418276	0.855949	0.435706	Biso	1.000000	H
O1871	1.0	0.383087	0.747026	0.603479	Biso	1.000000	O
H1374	1.0	0.376252	0.721534	0.593565	Biso	1.000000	H
H1375	1.0	0.379948	0.766671	0.584044	Biso	1.000000	H
O1872	1.0	0.372704	0.706310	0.412870	Biso	1.000000	O
H1376	1.0	0.390732	0.686818	0.404308	Biso	1.000000	H
H1377	1.0	0.385134	0.731578	0.414535	Biso	1.000000	H

O1873	1.0	0.400386	0.865564	0.211701	Biso	1.000000	O
H1378	1.0	0.383265	0.874998	0.192396	Biso	1.000000	H
H1379	1.0	0.425286	0.876906	0.207907	Biso	1.000000	H
O1874	1.0	0.763175	0.468262	0.772140	Biso	1.000000	O
H1380	1.0	0.767209	0.448878	0.791654	Biso	1.000000	H
H1381	1.0	0.748486	0.456769	0.751918	Biso	1.000000	H
O1875	1.0	0.943042	0.120746	0.694181	Biso	1.000000	O
H1382	1.0	0.935916	0.145566	0.705379	Biso	1.000000	H
H1383	1.0	0.955831	0.125444	0.670481	Biso	1.000000	H
O1876	1.0	0.038152	0.500592	0.549122	Biso	1.000000	O
H1384	1.0	0.018428	0.517392	0.539110	Biso	1.000000	H
H1385	1.0	0.043742	0.480001	0.531189	Biso	1.000000	H
O1877	1.0	0.477867	0.702652	0.690109	Biso	1.000000	O
H1386	1.0	0.464679	0.724842	0.679233	Biso	1.000000	H
H1387	1.0	0.498519	0.694931	0.673724	Biso	1.000000	H
O1878	1.0	0.116967	0.421071	0.156647	Biso	1.000000	O
H1388	1.0	0.099777	0.427384	0.177031	Biso	1.000000	H
H1389	1.0	0.123934	0.393645	0.158104	Biso	1.000000	H
O1879	1.0	0.462412	0.978054	0.702429	Biso	1.000000	O
H1390	1.0	0.461492	0.005013	0.710945	Biso	1.000000	H
H1391	1.0	0.436863	0.967760	0.700660	Biso	1.000000	H
O1880	1.0	0.498407	0.106370	0.141108	Biso	1.000000	O
H1392	1.0	0.522708	0.093404	0.143776	Biso	1.000000	H
H1393	1.0	0.496214	0.126891	0.159823	Biso	1.000000	H
O1881	1.0	0.535723	0.304705	0.346282	Biso	1.000000	O
H1394	1.0	0.550452	0.328036	0.351568	Biso	1.000000	H
H1395	1.0	0.541821	0.295215	0.321314	Biso	1.000000	H
O1882	1.0	0.886519	0.721548	0.722109	Biso	1.000000	O
H1396	1.0	0.885272	0.713631	0.695964	Biso	1.000000	H
H1397	1.0	0.906539	0.740716	0.725296	Biso	1.000000	H
O1883	1.0	0.874895	0.976101	0.929877	Biso	1.000000	O
H1398	1.0	0.876107	0.991788	0.907188	Biso	1.000000	H
H1399	1.0	0.897407	0.980670	0.944882	Biso	1.000000	H
O1884	1.0	0.206453	0.271400	0.037444	Biso	1.000000	O
H1400	1.0	0.224569	0.262888	0.018647	Biso	1.000000	H
H1401	1.0	0.201583	0.250150	0.054867	Biso	1.000000	H
O1885	1.0	0.935479	0.716772	0.793723	Biso	1.000000	O
H1402	1.0	0.912376	0.728392	0.803386	Biso	1.000000	H
H1403	1.0	0.945671	0.733171	0.773899	Biso	1.000000	H
O1886	1.0	0.480758	0.300525	0.206463	Biso	1.000000	O
H1404	1.0	0.457815	0.295039	0.192414	Biso	1.000000	H
H1405	1.0	0.490616	0.276373	0.216945	Biso	1.000000	H
O1887	1.0	0.047930	0.040473	0.114191	Biso	1.000000	O
H1406	1.0	0.027252	0.024621	0.104664	Biso	1.000000	H
H1407	1.0	0.060822	0.026452	0.134150	Biso	1.000000	H
O1888	1.0	0.540084	0.131882	0.600535	Biso	1.000000	O
H1408	1.0	0.540436	0.118850	0.576320	Biso	1.000000	H
H1409	1.0	0.536039	0.112691	0.620221	Biso	1.000000	H
O1889	1.0	0.427465	0.239450	0.414107	Biso	1.000000	O
H1410	1.0	0.438122	0.216234	0.402501	Biso	1.000000	H
H1411	1.0	0.422574	0.234323	0.440476	Biso	1.000000	H
O1890	1.0	0.124087	0.202403	0.437783	Biso	1.000000	O
H1412	1.0	0.151484	0.203893	0.439370	Biso	1.000000	H
H1413	1.0	0.113337	0.204149	0.462814	Biso	1.000000	H
O1891	1.0	0.933171	0.499181	0.703212	Biso	1.000000	O
H1414	1.0	0.934119	0.519121	0.722594	Biso	1.000000	H
H1415	1.0	0.910815	0.483085	0.706769	Biso	1.000000	H
O1892	1.0	0.876964	0.574473	0.440871	Biso	1.000000	O
H1416	1.0	0.885986	0.599979	0.448844	Biso	1.000000	H
H1417	1.0	0.864444	0.576861	0.416713	Biso	1.000000	H
O1893	1.0	0.049176	0.822384	0.240699	Biso	1.000000	O
H1418	1.0	0.059135	0.827552	0.265615	Biso	1.000000	H
H1419	1.0	0.041593	0.846839	0.229067	Biso	1.000000	H
O1894	1.0	0.709692	0.916927	0.923144	Biso	1.000000	O
H1420	1.0	0.721588	0.903671	0.944163	Biso	1.000000	H
H1421	1.0	0.727472	0.935291	0.912132	Biso	1.000000	H
O1895	1.0	0.645052	0.570125	0.744395	Biso	1.000000	O
H1422	1.0	0.659880	0.594002	0.743207	Biso	1.000000	H
H1423	1.0	0.649919	0.556918	0.768035	Biso	1.000000	H
O1896	1.0	0.621866	0.980839	0.994989	Biso	1.000000	O
H1424	1.0	0.641400	0.967837	0.009545	Biso	1.000000	H
H1425	1.0	0.631614	0.005407	0.985325	Biso	1.000000	H
O1897	1.0	0.469505	0.721233	0.549155	Biso	1.000000	O
H1426	1.0	0.465259	0.698299	0.564670	Biso	1.000000	H
H1427	1.0	0.495738	0.729374	0.551341	Biso	1.000000	H

O1898	1.0	0.127810	0.950572	0.299222	Biso	1.000000	O
H1428	1.0	0.102701	0.960641	0.304630	Biso	1.000000	H
H1429	1.0	0.146482	0.970802	0.304014	Biso	1.000000	H
O1899	1.0	0.603267	0.383098	0.922787	Biso	1.000000	O
H1430	1.0	0.584597	0.393645	0.940036	Biso	1.000000	H
H1431	1.0	0.599788	0.355028	0.920544	Biso	1.000000	H
O1900	1.0	0.251182	0.977615	0.985352	Biso	1.000000	O
H1432	1.0	0.275909	0.965750	0.988778	Biso	1.000000	H
H1433	1.0	0.246619	0.996371	0.005311	Biso	1.000000	H
O1901	1.0	0.666814	0.852718	0.864383	Biso	1.000000	O
H1434	1.0	0.672964	0.825419	0.860000	Biso	1.000000	H
H1435	1.0	0.668754	0.867112	0.840966	Biso	1.000000	H
O1902	1.0	0.934481	0.815909	0.146297	Biso	1.000000	O
H1436	1.0	0.946718	0.796789	0.130209	Biso	1.000000	H
H1437	1.0	0.910485	0.823636	0.135273	Biso	1.000000	H
O1903	1.0	0.377186	0.534041	0.742645	Biso	1.000000	O
H1438	1.0	0.368557	0.530000	0.717058	Biso	1.000000	H
H1439	1.0	0.404241	0.539013	0.742623	Biso	1.000000	H
O1904	1.0	0.036750	0.838127	0.109291	Biso	1.000000	O
H1440	1.0	0.042779	0.835855	0.082787	Biso	1.000000	H
H1441	1.0	0.024984	0.863313	0.114013	Biso	1.000000	H
H1442	1.0	0.512158	0.679847	0.193245	Biso	1.000000	H
H1443	1.0	0.532760	0.724243	0.287818	Biso	1.000000	H
H1444	1.0	0.402796	0.683476	0.133898	Biso	1.000000	H
O1905	1.0	0.573506	0.916606	0.666370	Biso	1.000000	O
H1445	1.0	0.594993	0.899596	0.671078	Biso	1.000000	H
H1446	1.0	0.570692	0.934834	0.687083	Biso	1.000000	H
O1906	1.0	0.956092	0.795321	0.913890	Biso	1.000000	O
H1447	1.0	0.939686	0.797672	0.935642	Biso	1.000000	H
H1448	1.0	0.947587	0.773711	0.898348	Biso	1.000000	H
O1907	1.0	0.661980	0.526627	0.683150	Biso	1.000000	O
H1449	1.0	0.636816	0.522742	0.672852	Biso	1.000000	H
H1450	1.0	0.660295	0.542353	0.705785	Biso	1.000000	H
O1908	1.0	0.445899	0.096842	0.087246	Biso	1.000000	O
H1451	1.0	0.467609	0.096037	0.103940	Biso	1.000000	H
H1452	1.0	0.449771	0.116911	0.068348	Biso	1.000000	H
O1909	1.0	0.736887	0.213486	0.451712	Biso	1.000000	O
H1453	1.0	0.745887	0.234557	0.435769	Biso	1.000000	H
H1454	1.0	0.712973	0.220961	0.463065	Biso	1.000000	H
O1910	1.0	0.644324	0.689590	0.114412	Biso	1.000000	O
H1455	1.0	0.643988	0.705191	0.091639	Biso	1.000000	H
H1456	1.0	0.639259	0.706270	0.135891	Biso	1.000000	H
O1911	1.0	0.300730	0.191150	0.192665	Biso	1.000000	O
H1457	1.0	0.300523	0.216752	0.180876	Biso	1.000000	H
H1458	1.0	0.302447	0.194184	0.219713	Biso	1.000000	H
O1912	1.0	0.119931	0.632352	0.420434	Biso	1.000000	O
H1459	1.0	0.096226	0.635811	0.433817	Biso	1.000000	H
H1460	1.0	0.117437	0.642612	0.395139	Biso	1.000000	H
O1913	1.0	0.044305	0.442071	0.952945	Biso	1.000000	O
H1461	1.0	0.020844	0.448236	0.965847	Biso	1.000000	H
H1462	1.0	0.057721	0.466112	0.947166	Biso	1.000000	H
O1914	1.0	0.404758	0.785922	0.266836	Biso	1.000000	O
H1463	1.0	0.395205	0.810710	0.276168	Biso	1.000000	H
H1464	1.0	0.427586	0.790414	0.252290	Biso	1.000000	H
O1915	1.0	0.042463	0.916520	0.557615	Biso	1.000000	O
H1465	1.0	0.049176	0.940985	0.569751	Biso	1.000000	H
H1466	1.0	0.061276	0.910211	0.538693	Biso	1.000000	H
O1916	1.0	0.686031	0.717851	0.006483	Biso	1.000000	O
H1467	1.0	0.683319	0.714278	0.979576	Biso	1.000000	H
H1468	1.0	0.712657	0.720523	0.012719	Biso	1.000000	H
O1917	1.0	0.527803	0.549552	0.842416	Biso	1.000000	O
H1469	1.0	0.508445	0.557915	0.860021	Biso	1.000000	H
H1470	1.0	0.520177	0.556455	0.817083	Biso	1.000000	H
O1918	1.0	0.558504	0.599054	0.280949	Biso	1.000000	O
H1471	1.0	0.551299	0.587618	0.257045	Biso	1.000000	H
H1472	1.0	0.581475	0.614292	0.277779	Biso	1.000000	H
O1919	1.0	0.426119	0.275860	0.040516	Biso	1.000000	O
H1473	1.0	0.443901	0.281283	0.060634	Biso	1.000000	H
H1474	1.0	0.423676	0.298801	0.024633	Biso	1.000000	H
O1920	1.0	0.490651	0.983025	0.108440	Biso	1.000000	O
H1475	1.0	0.496343	0.965838	0.087489	Biso	1.000000	H
H1476	1.0	0.496164	0.009873	0.101444	Biso	1.000000	H
O1921	1.0	0.983544	0.626457	0.397581	Biso	1.000000	O
H1477	1.0	0.978393	0.614536	0.421786	Biso	1.000000	H
H1478	1.0	0.959850	0.631231	0.384555	Biso	1.000000	H
O1922	1.0	0.069468	0.740401	0.931859	Biso	1.000000	O
H1479	1.0	0.058785	0.718367	0.945400	Biso	1.000000	H
H1480	1.0	0.078312	0.731725	0.907430	Biso	1.000000	H
O1923	1.0	0.240991	0.091007	0.010939	Biso	1.000000	O
H1481	1.0	0.258979	0.102133	0.028562	Biso	1.000000	H
H1482	1.0	0.232354	0.065835	0.020202	Biso	1.000000	H
O1924	1.0	0.879704	0.116771	0.422549	Biso	1.000000	O
H1483	1.0	0.857733	0.123421	0.407470	Biso	1.000000	H
H1484	1.0	0.901172	0.132425	0.414560	Biso	1.000000	H
O1925	1.0	0.132721	0.876142	0.281121	Biso	1.000000	O

H1485	1.0	0.132075	0.903168	0.289470	Biso	1.000000	H
H1486	1.0	0.139069	0.875248	0.254615	Biso	1.000000	H
O1926	1.0	0.501259	0.669473	0.853769	Biso	1.000000	O
H1487	1.0	0.517319	0.656669	0.835381	Biso	1.000000	H
H1488	1.0	0.508844	0.696638	0.856328	Biso	1.000000	H
O1927	1.0	0.638369	0.420529	0.389717	Biso	1.000000	O
H1489	1.0	0.623639	0.437002	0.373000	Biso	1.000000	H
H1490	1.0	0.663283	0.416187	0.378994	Biso	1.000000	H
O1928	1.0	0.881826	0.276357	0.432334	Biso	1.000000	O
H1491	1.0	0.899847	0.264166	0.449261	Biso	1.000000	H
H1492	1.0	0.894610	0.283896	0.409315	Biso	1.000000	H
O1929	1.0	0.583954	0.808088	0.564523	Biso	1.000000	O
H1493	1.0	0.598989	0.789483	0.578724	Biso	1.000000	H
H1494	1.0	0.598167	0.816733	0.542719	Biso	1.000000	H
O1930	1.0	0.378984	0.665824	0.029344	Biso	1.000000	O
H1495	1.0	0.363185	0.642726	0.026950	Biso	1.000000	H
H1496	1.0	0.403760	0.660562	0.018688	Biso	1.000000	H
O1931	1.0	0.742501	0.275935	0.252665	Biso	1.000000	O
H1497	1.0	0.760614	0.258670	0.264731	Biso	1.000000	H
H1498	1.0	0.735024	0.264920	0.228661	Biso	1.000000	H
O1932	1.0	0.029294	0.969706	0.030903	Biso	1.000000	O
H1499	1.0	0.052793	0.962018	0.018848	Biso	1.000000	H
H1500	1.0	0.015421	0.987291	0.014512	Biso	1.000000	H
O1933	1.0	0.358725	0.327724	0.346828	Biso	1.000000	O
H1501	1.0	0.356543	0.350927	0.362394	Biso	1.000000	H
H1502	1.0	0.362539	0.335428	0.320865	Biso	1.000000	H
O1934	1.0	0.314959	0.295311	0.743452	Biso	1.000000	O
H1503	1.0	0.311098	0.300587	0.769960	Biso	1.000000	H
H1504	1.0	0.340313	0.303316	0.736275	Biso	1.000000	H
O1935	1.0	0.384417	0.699248	0.930234	Biso	1.000000	O
H1505	1.0	0.371264	0.675164	0.924047	Biso	1.000000	H
H1506	1.0	0.369357	0.721451	0.922125	Biso	1.000000	H
O1936	1.0	0.032960	0.517644	0.707565	Biso	1.000000	O
H1507	1.0	0.020131	0.540195	0.696956	Biso	1.000000	H
H1508	1.0	0.015556	0.503321	0.723562	Biso	1.000000	H
O1937	1.0	0.175380	0.644912	0.253955	Biso	1.000000	O
H1509	1.0	0.192428	0.662010	0.267655	Biso	1.000000	H
H1510	1.0	0.151053	0.657737	0.250916	Biso	1.000000	H
O1938	1.0	0.793934	0.798808	0.761494	Biso	1.000000	O
H1511	1.0	0.813308	0.797025	0.742239	Biso	1.000000	H
H1512	1.0	0.796721	0.823289	0.775024	Biso	1.000000	H
O1939	1.0	0.853927	0.588649	0.370446	Biso	1.000000	O
H1513	1.0	0.855499	0.615879	0.362869	Biso	1.000000	H
H1514	1.0	0.864917	0.572244	0.351050	Biso	1.000000	H
O1940	1.0	0.092232	0.870173	0.687579	Biso	1.000000	O
H1515	1.0	0.087489	0.859025	0.712204	Biso	1.000000	H
H1516	1.0	0.102126	0.850074	0.671012	Biso	1.000000	H
O1941	1.0	0.336169	0.375610	0.513139	Biso	1.000000	O
H1517	1.0	0.352245	0.377149	0.491083	Biso	1.000000	H
H1518	1.0	0.309993	0.379782	0.505867	Biso	1.000000	H
O1942	1.0	0.393774	0.952858	0.686347	Biso	1.000000	O
H1519	1.0	0.397555	0.936668	0.664269	Biso	1.000000	H
H1520	1.0	0.382397	0.977537	0.678974	Biso	1.000000	H
O1943	1.0	0.715930	0.765473	0.651316	Biso	1.000000	O
H1521	1.0	0.727810	0.775142	0.674076	Biso	1.000000	H
H1522	1.0	0.728535	0.741667	0.643280	Biso	1.000000	H
O1944	1.0	0.125095	0.345336	0.163239	Biso	1.000000	O
H1523	1.0	0.135710	0.328482	0.182486	Biso	1.000000	H
H1524	1.0	0.099659	0.336646	0.157164	Biso	1.000000	H
O1945	1.0	0.199808	0.001264	0.826050	Biso	1.000000	O
H1525	1.0	0.178030	0.018509	0.827590	Biso	1.000000	H
H1526	1.0	0.191794	0.974461	0.830281	Biso	1.000000	H
O1946	1.0	0.628706	0.255745	0.968531	Biso	1.000000	O
H1527	1.0	0.618080	0.230213	0.962944	Biso	1.000000	H
H1528	1.0	0.646201	0.253530	0.989444	Biso	1.000000	H
O1947	1.0	0.954751	0.382922	0.968977	Biso	1.000000	O
H1529	1.0	0.948593	0.366852	0.990603	Biso	1.000000	H
H1530	1.0	0.968456	0.367335	0.950691	Biso	1.000000	H
O1948	1.0	0.967607	0.932820	0.550496	Biso	1.000000	O
H1531	1.0	0.994162	0.925514	0.550608	Biso	1.000000	H
H1532	1.0	0.959102	0.937026	0.576099	Biso	1.000000	H
O1949	1.0	0.113270	0.587533	0.688212	Biso	1.000000	O
H1533	1.0	0.096223	0.606604	0.677170	Biso	1.000000	H
H1534	1.0	0.107932	0.585079	0.714845	Biso	1.000000	H
O1950	1.0	0.460901	0.472195	0.811549	Biso	1.000000	O
H1535	1.0	0.452698	0.497630	0.820542	Biso	1.000000	H
H1536	1.0	0.479569	0.475625	0.791820	Biso	1.000000	H
O1951	1.0	0.571673	0.196187	0.407963	Biso	1.000000	O
H1537	1.0	0.570291	0.207170	0.433062	Biso	1.000000	H

H1538	1.0	0.553159	0.208928	0.391955	Biso	1.000000	H
O1952	1.0	0.156667	0.284640	0.891250	Biso	1.000000	O
H1539	1.0	0.164997	0.291395	0.866100	Biso	1.000000	H
H1540	1.0	0.148410	0.308116	0.904217	Biso	1.000000	H
O1953	1.0	0.868929	0.360628	0.983687	Biso	1.000000	O
H1541	1.0	0.872740	0.383053	0.967399	Biso	1.000000	H
H1542	1.0	0.892735	0.353679	0.995559	Biso	1.000000	H
O1954	1.0	0.264615	0.468046	0.851632	Biso	1.000000	O
H1543	1.0	0.252732	0.480424	0.873146	Biso	1.000000	H
H1544	1.0	0.263684	0.439830	0.854573	Biso	1.000000	H
O1955	1.0	0.848626	0.876012	0.185613	Biso	1.000000	O
H1545	1.0	0.827653	0.863105	0.198131	Biso	1.000000	H
H1546	1.0	0.844471	0.904082	0.185648	Biso	1.000000	H
O1956	1.0	0.105745	0.641550	0.976040	Biso	1.000000	O
H1547	1.0	0.107575	0.614868	0.966883	Biso	1.000000	H
H1548	1.0	0.122780	0.658177	0.961799	Biso	1.000000	H
O1957	1.0	0.586955	0.470272	0.711149	Biso	1.000000	O
H1549	1.0	0.586570	0.492030	0.693636	Biso	1.000000	H
H1550	1.0	0.593509	0.479757	0.736005	Biso	1.000000	H
O1958	1.0	0.276516	0.787364	0.450131	Biso	1.000000	O
H1551	1.0	0.296228	0.801532	0.463392	Biso	1.000000	H
H1552	1.0	0.287838	0.771527	0.430489	Biso	1.000000	H
O1959	1.0	0.872751	0.456315	0.727234	Biso	1.000000	O
H1553	1.0	0.860792	0.430777	0.728406	Biso	1.000000	H
H1554	1.0	0.855455	0.474924	0.715851	Biso	1.000000	H
O1960	1.0	0.326913	0.407259	0.068254	Biso	1.000000	O
H1555	1.0	0.300823	0.400833	0.074197	Biso	1.000000	H
H1556	1.0	0.332552	0.433489	0.077072	Biso	1.000000	H
O1961	1.0	0.738547	0.642308	0.041629	Biso	1.000000	O
H1557	1.0	0.731352	0.650458	0.066746	Biso	1.000000	H
H1558	1.0	0.716320	0.633093	0.028260	Biso	1.000000	H
O1962	1.0	0.993524	0.946369	0.375982	Biso	1.000000	O
H1559	1.0	0.970904	0.930421	0.378288	Biso	1.000000	H
H1560	1.0	0.987317	0.970623	0.363215	Biso	1.000000	H
O1963	1.0	0.802159	0.516071	0.103655	Biso	1.000000	O
H1561	1.0	0.783893	0.523128	0.122860	Biso	1.000000	H
H1562	1.0	0.827535	0.517463	0.114032	Biso	1.000000	H
O1964	1.0	0.774118	0.171422	0.629183	Biso	1.000000	O
H1563	1.0	0.775066	0.170204	0.656397	Biso	1.000000	H
H1564	1.0	0.754249	0.154260	0.620057	Biso	1.000000	H
O1965	1.0	0.474825	0.606556	0.228128	Biso	1.000000	O
H1565	1.0	0.499330	0.594834	0.223067	Biso	1.000000	H
H1566	1.0	0.476340	0.622243	0.250800	Biso	1.000000	H
O1966	1.0	0.984373	0.546783	0.801153	Biso	1.000000	O
H1567	1.0	0.999818	0.565647	0.787724	Biso	1.000000	H
H1568	1.0	0.000214	0.525437	0.809889	Biso	1.000000	H
O1967	1.0	0.971495	0.841867	0.563522	Biso	1.000000	O
H1569	1.0	0.971864	0.861203	0.583483	Biso	1.000000	H
H1570	1.0	0.976652	0.816176	0.573951	Biso	1.000000	H
O1968	1.0	0.338837	0.886385	0.653197	Biso	1.000000	O
H1571	1.0	0.334083	0.861282	0.641359	Biso	1.000000	H
H1572	1.0	0.360989	0.898389	0.641915	Biso	1.000000	H
O1969	1.0	0.673915	0.876321	0.077439	Biso	1.000000	O
H1573	1.0	0.701305	0.874050	0.077154	Biso	1.000000	H
H1574	1.0	0.662829	0.853575	0.065378	Biso	1.000000	H
O1970	1.0	0.169098	0.215152	0.769385	Biso	1.000000	O
H1575	1.0	0.142612	0.210263	0.763835	Biso	1.000000	H
H1576	1.0	0.176690	0.199651	0.790945	Biso	1.000000	H
O1971	1.0	0.940043	0.534385	0.430061	Biso	1.000000	O
H1577	1.0	0.945803	0.527010	0.404365	Biso	1.000000	H
H1578	1.0	0.914234	0.544026	0.431457	Biso	1.000000	H
O1972	1.0	0.902549	0.833995	0.222843	Biso	1.000000	O
H1579	1.0	0.889817	0.826873	0.246011	Biso	1.000000	H
H1580	1.0	0.885480	0.849191	0.207233	Biso	1.000000	H
O1973	1.0	0.369761	0.161747	0.326425	Biso	1.000000	O
H1581	1.0	0.396173	0.155162	0.330514	Biso	1.000000	H
H1582	1.0	0.365818	0.167936	0.300114	Biso	1.000000	H
O1974	1.0	0.758825	0.972349	0.751823	Biso	1.000000	O
H1583	1.0	0.739072	0.989842	0.760608	Biso	1.000000	H
H1584	1.0	0.747989	0.953184	0.734822	Biso	1.000000	H
O1975	1.0	0.802033	0.412612	0.565269	Biso	1.000000	O
H1585	1.0	0.827263	0.413427	0.576044	Biso	1.000000	H
H1586	1.0	0.801590	0.394117	0.544589	Biso	1.000000	H
O1976	1.0	0.815506	0.898216	0.288096	Biso	1.000000	O
H1587	1.0	0.834045	0.884628	0.303414	Biso	1.000000	H
H1588	1.0	0.828249	0.917299	0.272354	Biso	1.000000	H

O1977	1.0	0.788070	0.406117	0.248051	Biso	1.000000	O
H1589	1.0	0.807276	0.410882	0.267002	Biso	1.000000	H
H1590	1.0	0.791422	0.380088	0.237671	Biso	1.000000	H
O1978	1.0	0.891785	0.032315	0.123733	Biso	1.000000	O
H1591	1.0	0.879924	0.057861	0.125564	Biso	1.000000	H
H1592	1.0	0.907792	0.028035	0.145505	Biso	1.000000	H
O1979	1.0	0.639690	0.328888	0.750424	Biso	1.000000	O
H1593	1.0	0.621028	0.316058	0.734653	Biso	1.000000	H
H1594	1.0	0.628528	0.334823	0.774670	Biso	1.000000	H
O1980	1.0	0.298484	0.657237	0.113277	Biso	1.000000	O
H1595	1.0	0.321816	0.644058	0.120157	Biso	1.000000	H
H1596	1.0	0.288663	0.646000	0.090217	Biso	1.000000	H
O1981	1.0	0.776585	0.107025	0.306631	Biso	1.000000	O
H1597	1.0	0.803137	0.113684	0.303692	Biso	1.000000	H
H1598	1.0	0.763702	0.127903	0.319973	Biso	1.000000	H
O1982	1.0	0.201724	0.692043	0.451900	Biso	1.000000	O
H1599	1.0	0.181134	0.681670	0.466957	Biso	1.000000	H
H1600	1.0	0.207389	0.718552	0.459900	Biso	1.000000	H
O1983	1.0	0.765417	0.091769	0.949303	Biso	1.000000	O
H1601	1.0	0.767819	0.094735	0.922299	Biso	1.000000	H
H1602	1.0	0.788671	0.100210	0.961362	Biso	1.000000	H
O1984	1.0	0.000321	0.648033	0.144465	Biso	1.000000	O
H1603	1.0	0.986267	0.649496	0.167846	Biso	1.000000	H
H1604	1.0	0.027256	0.647394	0.149839	Biso	1.000000	H
O1985	1.0	0.014287	0.309246	0.329906	Biso	1.000000	O
H1605	1.0	0.006016	0.283064	0.323269	Biso	1.000000	H
H1606	1.0	0.029537	0.308135	0.352557	Biso	1.000000	H
O1986	1.0	0.282793	0.245569	0.644736	Biso	1.000000	O
H1607	1.0	0.264318	0.225596	0.651030	Biso	1.000000	H
H1608	1.0	0.280660	0.252084	0.618288	Biso	1.000000	H
O1987	1.0	0.762427	0.001141	0.567627	Biso	1.000000	O
H1609	1.0	0.764618	0.022274	0.585704	Biso	1.000000	H
H1610	1.0	0.757448	0.011928	0.542902	Biso	1.000000	H
O1988	1.0	0.578952	0.833478	0.643016	Biso	1.000000	O
H1611	1.0	0.599250	0.844675	0.657926	Biso	1.000000	H
H1612	1.0	0.570700	0.809094	0.654333	Biso	1.000000	H
O1989	1.0	0.413755	0.519601	0.428693	Biso	1.000000	O
H1613	1.0	0.389000	0.516795	0.417174	Biso	1.000000	H
H1614	1.0	0.428715	0.538187	0.414391	Biso	1.000000	H
O1990	1.0	0.082914	0.763312	0.735482	Biso	1.000000	O
H1615	1.0	0.088870	0.789565	0.744027	Biso	1.000000	H
H1616	1.0	0.083320	0.762599	0.708235	Biso	1.000000	H
O1991	1.0	0.464644	0.398314	0.683458	Biso	1.000000	O
H1617	1.0	0.490624	0.407310	0.681367	Biso	1.000000	H
H1618	1.0	0.459186	0.380168	0.663203	Biso	1.000000	H
O1992	1.0	0.009832	0.764255	0.661641	Biso	1.000000	O
H1619	1.0	0.004613	0.746756	0.682474	Biso	1.000000	H
H1620	1.0	0.007412	0.791172	0.669987	Biso	1.000000	H
O1993	1.0	0.569601	0.439592	0.180941	Biso	1.000000	O
H1621	1.0	0.566240	0.462077	0.197253	Biso	1.000000	H
H1622	1.0	0.575210	0.448449	0.155648	Biso	1.000000	H
O1994	1.0	0.586905	0.198797	0.891471	Biso	1.000000	O
H1623	1.0	0.594707	0.177825	0.874803	Biso	1.000000	H
H1624	1.0	0.593446	0.192064	0.917145	Biso	1.000000	H
O1995	1.0	0.400399	0.023159	0.222875	Biso	1.000000	O
H1625	1.0	0.422532	0.032533	0.209457	Biso	1.000000	H
H1626	1.0	0.406841	0.999533	0.236580	Biso	1.000000	H
O1996	1.0	0.931604	0.046999	0.060772	Biso	1.000000	O
H1627	1.0	0.913033	0.056131	0.042694	Biso	1.000000	H
H1628	1.0	0.918732	0.037503	0.083062	Biso	1.000000	H
O1997	1.0	0.398591	0.032097	0.309373	Biso	1.000000	O
H1629	1.0	0.420919	0.028299	0.293904	Biso	1.000000	H
H1630	1.0	0.390204	0.007041	0.319137	Biso	1.000000	H
O1998	1.0	0.317331	0.991391	0.935217	Biso	1.000000	O
H1631	1.0	0.295577	0.977580	0.925131	Biso	1.000000	H
H1632	1.0	0.312288	0.019306	0.935252	Biso	1.000000	H
O1999	1.0	0.850393	0.220416	0.317289	Biso	1.000000	O
H1633	1.0	0.863595	0.244049	0.309744	Biso	1.000000	H
H1634	1.0	0.859786	0.211846	0.341548	Biso	1.000000	H
O2000	1.0	0.538828	0.163569	0.951860	Biso	1.000000	O
H1635	1.0	0.514807	0.155039	0.962262	Biso	1.000000	H
H1636	1.0	0.547374	0.186813	0.965028	Biso	1.000000	H
O2001	1.0	0.398511	0.448310	0.776801	Biso	1.000000	O
H1637	1.0	0.389547	0.422389	0.783496	Biso	1.000000	H
H1638	1.0	0.420078	0.455085	0.792393	Biso	1.000000	H
O2002	1.0	0.118730	0.261378	0.258849	Biso	1.000000	O
H1639	1.0	0.124720	0.271594	0.283579	Biso	1.000000	H

H1640	1.0	0.091482	0.261422	0.255312	Biso	1.000000	H
O2003	1.0	0.615290	0.072349	0.744133	Biso	1.000000	O
H1641	1.0	0.614356	0.049503	0.760294	Biso	1.000000	H
H1642	1.0	0.589744	0.079885	0.737169	Biso	1.000000	H
O2004	1.0	0.193004	0.620176	0.968879	Biso	1.000000	O
H1643	1.0	0.212822	0.609527	0.953002	Biso	1.000000	H
H1644	1.0	0.179483	0.640471	0.955330	Biso	1.000000	H
O2005	1.0	0.308966	0.984268	0.540860	Biso	1.000000	O
H1645	1.0	0.285093	0.971497	0.535207	Biso	1.000000	H
H1646	1.0	0.313786	0.982816	0.567660	Biso	1.000000	H
O2006	1.0	0.123253	0.538193	0.139680	Biso	1.000000	O
H1647	1.0	0.138970	0.518244	0.151222	Biso	1.000000	H
H1648	1.0	0.112502	0.528095	0.116543	Biso	1.000000	H
O2007	1.0	0.203856	0.348144	0.028099	Biso	1.000000	O
H1649	1.0	0.200559	0.364288	0.050284	Biso	1.000000	H
H1650	1.0	0.207417	0.321090	0.035595	Biso	1.000000	H
O2008	1.0	0.459645	0.163609	0.907553	Biso	1.000000	O
H1651	1.0	0.463231	0.155871	0.933538	Biso	1.000000	H
H1652	1.0	0.476202	0.185459	0.901769	Biso	1.000000	H
O2009	1.0	0.281771	0.947104	0.826672	Biso	1.000000	O
H1653	1.0	0.263713	0.943654	0.806395	Biso	1.000000	H
H1654	1.0	0.289575	0.974307	0.827787	Biso	1.000000	H
O2010	1.0	0.660985	0.030326	0.250631	Biso	1.000000	O
H1655	1.0	0.637541	0.039685	0.239603	Biso	1.000000	H
H1656	1.0	0.657921	0.003274	0.258342	Biso	1.000000	H
O2011	1.0	0.474828	0.041277	0.264758	Biso	1.000000	O
H1657	1.0	0.493185	0.035518	0.245242	Biso	1.000000	H
H1658	1.0	0.461973	0.065629	0.258929	Biso	1.000000	H
O2012	1.0	0.195219	0.177943	0.367767	Biso	1.000000	O
H1659	1.0	0.170362	0.185234	0.358486	Biso	1.000000	H
H1660	1.0	0.213536	0.179797	0.347523	Biso	1.000000	H
O2013	1.0	0.189594	0.110715	0.310477	Biso	1.000000	O
H1661	1.0	0.212445	0.112531	0.325520	Biso	1.000000	H
H1662	1.0	0.195893	0.100836	0.285697	Biso	1.000000	H
O2014	1.0	0.977318	0.106776	0.060782	Biso	1.000000	O
H1663	1.0	0.977563	0.117786	0.085907	Biso	1.000000	H
H1664	1.0	0.962492	0.082866	0.060722	Biso	1.000000	H
O2015	1.0	0.270400	0.685787	0.635441	Biso	1.000000	O
H1665	1.0	0.277577	0.695948	0.611005	Biso	1.000000	H
H1666	1.0	0.292785	0.676447	0.648465	Biso	1.000000	H
O2016	1.0	0.972240	0.093574	0.984466	Biso	1.000000	O
H1667	1.0	0.974246	0.101641	0.010525	Biso	1.000000	H
H1668	1.0	0.979452	0.066274	0.982203	Biso	1.000000	H
O2017	1.0	0.713646	0.128451	0.771772	Biso	1.000000	O
H1669	1.0	0.712259	0.143048	0.795113	Biso	1.000000	H
H1670	1.0	0.690824	0.132969	0.757225	Biso	1.000000	H
O2018	1.0	0.030899	0.484039	0.621504	Biso	1.000000	O
H1671	1.0	0.008074	0.471104	0.630235	Biso	1.000000	H
H1672	1.0	0.029668	0.487319	0.594457	Biso	1.000000	H
O2019	1.0	0.603328	0.289797	0.530775	Biso	1.000000	O
H1673	1.0	0.626851	0.298446	0.519390	Biso	1.000000	H
H1674	1.0	0.585770	0.311597	0.532094	Biso	1.000000	H
O2020	1.0	0.685748	0.290106	0.928841	Biso	1.000000	O
H1675	1.0	0.682005	0.311106	0.910876	Biso	1.000000	H
H1676	1.0	0.661329	0.280372	0.937149	Biso	1.000000	H
O2021	1.0	0.621567	0.262508	0.868152	Biso	1.000000	O
H1677	1.0	0.609610	0.238827	0.877406	Biso	1.000000	H
H1678	1.0	0.617214	0.264830	0.841331	Biso	1.000000	H
O2022	1.0	0.116491	0.890676	0.605886	Biso	1.000000	O
H1679	1.0	0.128357	0.886931	0.581564	Biso	1.000000	H
H1680	1.0	0.093193	0.875662	0.607014	Biso	1.000000	H
O2023	1.0	0.525090	0.852047	0.746101	Biso	1.000000	O
H1681	1.0	0.514634	0.830803	0.731281	Biso	1.000000	H
H1682	1.0	0.515238	0.876793	0.736967	Biso	1.000000	H
O2024	1.0	0.342888	0.377033	0.586784	Biso	1.000000	O
H1683	1.0	0.344974	0.375641	0.559638	Biso	1.000000	H
H1684	1.0	0.360654	0.358861	0.598109	Biso	1.000000	H
O2025	1.0	0.302978	0.624381	0.961990	Biso	1.000000	O
H1685	1.0	0.283246	0.607823	0.951631	Biso	1.000000	H
H1686	1.0	0.292351	0.649649	0.968596	Biso	1.000000	H
O2026	1.0	0.605818	0.443647	0.022513	Biso	1.000000	O
H1687	1.0	0.614120	0.418655	0.032494	Biso	1.000000	H
H1688	1.0	0.627673	0.458638	0.014392	Biso	1.000000	H
O2027	1.0	0.034912	0.661846	0.802898	Biso	1.000000	O
H1689	1.0	0.039276	0.688346	0.794113	Biso	1.000000	H
H1690	1.0	0.012392	0.661283	0.818511	Biso	1.000000	H
O2028	1.0	0.969225	0.275733	0.427984	Biso	1.000000	O
H1691	1.0	0.957142	0.262657	0.449002	Biso	1.000000	H
H1692	1.0	0.987232	0.294843	0.437342	Biso	1.000000	H

O2029	1.0	0.120939	0.570699	0.047314	Biso	1.000000	O
H1693	1.0	0.121017	0.597867	0.055242	Biso	1.000000	H
H1694	1.0	0.144119	0.565042	0.033718	Biso	1.000000	H
O2030	1.0	0.054938	0.645034	0.453903	Biso	1.000000	O
H1695	1.0	0.044396	0.635538	0.477367	Biso	1.000000	H
H1696	1.0	0.035368	0.658358	0.439667	Biso	1.000000	H
O2031	1.0	0.103600	0.713497	0.428050	Biso	1.000000	O
H1697	1.0	0.100211	0.701485	0.403580	Biso	1.000000	H
H1698	1.0	0.122081	0.698680	0.442354	Biso	1.000000	H
O2032	1.0	0.686306	0.158423	0.494026	Biso	1.000000	O
H1699	1.0	0.705468	0.170356	0.509852	Biso	1.000000	H
H1700	1.0	0.684536	0.130715	0.499734	Biso	1.000000	H
O2033	1.0	0.926127	0.719393	0.953429	Biso	1.000000	O
H1701	1.0	0.931338	0.726384	0.927519	Biso	1.000000	H
H1702	1.0	0.943608	0.699120	0.961406	Biso	1.000000	H
O2034	1.0	0.831489	0.630007	0.730175	Biso	1.000000	O
H1703	1.0	0.850954	0.611267	0.723347	Biso	1.000000	H
H1704	1.0	0.807070	0.620741	0.721387	Biso	1.000000	H
O2035	1.0	0.665396	0.532846	0.810898	Biso	1.000000	O
H1705	1.0	0.661219	0.553729	0.828899	Biso	1.000000	H
H1706	1.0	0.692360	0.527950	0.808529	Biso	1.000000	H
O2036	1.0	0.323187	0.410970	0.148528	Biso	1.000000	O
H1707	1.0	0.303112	0.400852	0.132650	Biso	1.000000	H
H1708	1.0	0.345916	0.414739	0.133641	Biso	1.000000	H
O2037	1.0	0.434566	0.904293	0.833306	Biso	1.000000	O
H1709	1.0	0.416989	0.883685	0.826399	Biso	1.000000	H
H1710	1.0	0.453418	0.893928	0.850462	Biso	1.000000	H
O2038	1.0	0.032435	0.744885	0.361953	Biso	1.000000	O
H1711	1.0	0.030105	0.717024	0.357218	Biso	1.000000	H
H1712	1.0	0.058333	0.753072	0.357330	Biso	1.000000	H
O2039	1.0	0.341118	0.468665	0.838074	Biso	1.000000	O
H1713	1.0	0.358189	0.457542	0.856576	Biso	1.000000	H
H1714	1.0	0.315613	0.469321	0.848200	Biso	1.000000	H
O2040	1.0	0.814960	0.944296	0.569761	Biso	1.000000	O
H1715	1.0	0.796289	0.964969	0.567278	Biso	1.000000	H
H1716	1.0	0.808779	0.928149	0.591330	Biso	1.000000	H
O2041	1.0	0.706532	0.400131	0.038594	Biso	1.000000	O
H1717	1.0	0.716054	0.390453	0.014774	Biso	1.000000	H
H1718	1.0	0.680123	0.392816	0.041343	Biso	1.000000	H
O2042	1.0	0.539735	0.969566	0.162600	Biso	1.000000	O
H1719	1.0	0.535918	0.946937	0.178627	Biso	1.000000	H
H1720	1.0	0.519240	0.971233	0.144513	Biso	1.000000	H
O2043	1.0	0.719046	0.383930	0.573975	Biso	1.000000	O
H1721	1.0	0.727612	0.410385	0.579078	Biso	1.000000	H
H1722	1.0	0.740481	0.366179	0.574834	Biso	1.000000	H
O2044	1.0	0.397503	0.742037	0.010341	Biso	1.000000	O
H1723	1.0	0.384510	0.718941	0.019589	Biso	1.000000	H
H1724	1.0	0.417987	0.734182	0.993809	Biso	1.000000	H
O2045	1.0	0.515871	0.236379	0.371411	Biso	1.000000	O
H1725	1.0	0.498800	0.224199	0.353533	Biso	1.000000	H
H1726	1.0	0.524953	0.261011	0.361271	Biso	1.000000	H
O2046	1.0	0.615881	0.325145	0.006186	Biso	1.000000	O
H1727	1.0	0.621100	0.299196	0.996401	Biso	1.000000	H
H1728	1.0	0.589293	0.331271	0.002576	Biso	1.000000	H
O2047	1.0	0.250757	0.612738	0.152727	Biso	1.000000	O
H1729	1.0	0.229180	0.604509	0.137809	Biso	1.000000	H
H1730	1.0	0.266720	0.629916	0.137881	Biso	1.000000	H
O2048	1.0	0.234542	0.160973	0.481237	Biso	1.000000	O
H1731	1.0	0.232725	0.137095	0.495880	Biso	1.000000	H
H1732	1.0	0.234829	0.183363	0.498001	Biso	1.000000	H
O2049	1.0	0.711300	0.655794	0.109085	Biso	1.000000	O
H1733	1.0	0.711855	0.628067	0.114951	Biso	1.000000	H
H1734	1.0	0.685514	0.665395	0.111077	Biso	1.000000	H
O2050	1.0	0.104246	0.036864	0.241347	Biso	1.000000	O
H1735	1.0	0.079122	0.046333	0.235071	Biso	1.000000	H
H1736	1.0	0.106525	0.034404	0.268408	Biso	1.000000	H
O2051	1.0	0.628915	0.825132	0.508317	Biso	1.000000	O
H1737	1.0	0.617799	0.819019	0.484088	Biso	1.000000	H
H1738	1.0	0.635884	0.852590	0.509010	Biso	1.000000	H
O2052	1.0	0.340431	0.692114	0.854218	Biso	1.000000	O
H1739	1.0	0.338617	0.670234	0.871500	Biso	1.000000	H
H1740	1.0	0.316061	0.705126	0.852550	Biso	1.000000	H
O2053	1.0	0.664115	0.458414	0.845954	Biso	1.000000	O
H1741	1.0	0.660812	0.483860	0.834307	Biso	1.000000	H
H1742	1.0	0.639495	0.446893	0.850893	Biso	1.000000	H
O2054	1.0	0.545467	0.246518	0.758656	Biso	1.000000	O
H1743	1.0	0.542230	0.218400	0.756595	Biso	1.000000	H
H1744	1.0	0.533942	0.259209	0.737120	Biso	1.000000	H
O2055	1.0	0.121916	0.100861	0.341365	Biso	1.000000	O
H1745	1.0	0.118836	0.111143	0.366589	Biso	1.000000	H
H1746	1.0	0.148114	0.103738	0.333616	Biso	1.000000	H

O2056	1.0	0.485925	0.908978	0.999431	Biso	1.000000	O
H1747	1.0	0.488409	0.881044	0.995188	Biso	1.000000	H
H1748	1.0	0.476098	0.921376	0.976929	Biso	1.000000	H
O2057	1.0	0.363825	0.175439	0.947690	Biso	1.000000	O
H1749	1.0	0.365463	0.153428	0.930545	Biso	1.000000	H
H1750	1.0	0.381855	0.171927	0.967982	Biso	1.000000	H
O2058	1.0	0.859313	0.593309	0.050729	Biso	1.000000	O
H1751	1.0	0.860770	0.621416	0.047122	Biso	1.000000	H
H1752	1.0	0.833608	0.586107	0.057454	Biso	1.000000	H
O2059	1.0	0.155030	0.195090	0.251188	Biso	1.000000	O
H1753	1.0	0.142247	0.174449	0.264959	Biso	1.000000	H
H1754	1.0	0.140912	0.219306	0.253753	Biso	1.000000	H
O2060	1.0	0.833960	0.248168	0.017148	Biso	1.000000	O
H1755	1.0	0.824339	0.255241	0.041762	Biso	1.000000	H
H1756	1.0	0.829184	0.269366	0.999639	Biso	1.000000	H
O2061	1.0	0.112501	0.506013	0.770058	Biso	1.000000	O
H1757	1.0	0.138935	0.498550	0.768005	Biso	1.000000	H
H1758	1.0	0.097007	0.482696	0.772487	Biso	1.000000	H
O2062	1.0	0.077641	0.822614	0.534218	Biso	1.000000	O
H1759	1.0	0.084367	0.847847	0.523644	Biso	1.000000	H
H1760	1.0	0.098585	0.812783	0.549130	Biso	1.000000	H
O2063	1.0	0.368931	0.414307	0.008456	Biso	1.000000	O
H1761	1.0	0.352374	0.420364	0.987494	Biso	1.000000	H
H1762	1.0	0.353836	0.409985	0.030851	Biso	1.000000	H
O2064	1.0	0.868146	0.371561	0.740325	Biso	1.000000	O
H1763	1.0	0.858874	0.352995	0.758787	Biso	1.000000	H
H1764	1.0	0.895261	0.374889	0.743395	Biso	1.000000	H
O2065	1.0	0.879902	0.088912	0.825151	Biso	1.000000	O
H1765	1.0	0.852625	0.085965	0.823423	Biso	1.000000	H
H1766	1.0	0.887443	0.088903	0.851362	Biso	1.000000	H
O2066	1.0	0.506970	0.878817	0.641242	Biso	1.000000	O
H1767	1.0	0.495702	0.904375	0.637210	Biso	1.000000	H
H1768	1.0	0.527866	0.881143	0.658801	Biso	1.000000	H
O2067	1.0	0.877025	0.199295	0.380556	Biso	1.000000	O
H1769	1.0	0.900353	0.186959	0.388758	Biso	1.000000	H
H1770	1.0	0.860418	0.203012	0.401978	Biso	1.000000	H
O2068	1.0	0.430488	0.181911	0.148720	Biso	1.000000	O
H1771	1.0	0.448213	0.187428	0.128574	Biso	1.000000	H
H1772	1.0	0.421422	0.155210	0.146428	Biso	1.000000	H
O2069	1.0	0.396854	0.597395	0.684856	Biso	1.000000	O
H1773	1.0	0.369690	0.597631	0.688961	Biso	1.000000	H
H1774	1.0	0.405728	0.570546	0.683714	Biso	1.000000	H
O2070	1.0	0.967610	0.156748	0.329206	Biso	1.000000	O
H1775	1.0	0.947735	0.142206	0.316577	Biso	1.000000	H
H1776	1.0	0.983931	0.168912	0.310643	Biso	1.000000	H
O2071	1.0	0.976744	0.314565	0.249965	Biso	1.000000	O
H1777	1.0	0.975190	0.289075	0.238053	Biso	1.000000	H
H1778	1.0	0.958626	0.332171	0.238370	Biso	1.000000	H
O2072	1.0	0.859679	0.170265	0.202692	Biso	1.000000	O
H1779	1.0	0.866379	0.196861	0.209553	Biso	1.000000	H
H1780	1.0	0.880210	0.158630	0.188428	Biso	1.000000	H
O2073	1.0	0.610415	0.488620	0.779290	Biso	1.000000	O
H1781	1.0	0.629954	0.506411	0.787996	Biso	1.000000	H
H1782	1.0	0.590492	0.486941	0.797994	Biso	1.000000	H
O2074	1.0	0.449123	0.052380	0.724293	Biso	1.000000	O
H1783	1.0	0.467279	0.072833	0.718525	Biso	1.000000	H
H1784	1.0	0.424145	0.060337	0.715873	Biso	1.000000	H
O2075	1.0	0.851314	0.410465	0.834816	Biso	1.000000	O
H1785	1.0	0.852138	0.438669	0.831747	Biso	1.000000	H
H1786	1.0	0.870240	0.402494	0.853038	Biso	1.000000	H
O2076	1.0	0.431925	0.855461	0.637442	Biso	1.000000	O
H1787	1.0	0.419672	0.836605	0.653802	Biso	1.000000	H
H1788	1.0	0.459172	0.853771	0.640590	Biso	1.000000	H
O2077	1.0	0.513323	0.731484	0.769077	Biso	1.000000	O
H1789	1.0	0.520823	0.707724	0.756132	Biso	1.000000	H
H1790	1.0	0.487115	0.729280	0.776992	Biso	1.000000	H
O2078	1.0	0.864598	0.180135	0.646410	Biso	1.000000	O
H1791	1.0	0.884686	0.197507	0.638170	Biso	1.000000	H
H1792	1.0	0.851979	0.169047	0.624658	Biso	1.000000	H
O2079	1.0	0.415959	0.236909	0.894140	Biso	1.000000	O
H1793	1.0	0.409594	0.212588	0.881563	Biso	1.000000	H
H1794	1.0	0.424036	0.256065	0.875681	Biso	1.000000	H
O2080	1.0	0.583929	0.856724	0.846423	Biso	1.000000	O
H1795	1.0	0.596911	0.842134	0.826904	Biso	1.000000	H
H1796	1.0	0.575383	0.881603	0.836381	Biso	1.000000	H
O2081	1.0	0.505205	0.404340	0.198940	Biso	1.000000	O
H1797	1.0	0.528509	0.416201	0.190053	Biso	1.000000	H
H1798	1.0	0.486557	0.404524	0.178919	Biso	1.000000	H
O2082	1.0	0.745975	0.895169	0.261175	Biso	1.000000	O
H1799	1.0	0.723685	0.894038	0.277078	Biso	1.000000	H
H1800	1.0	0.768550	0.893110	0.276596	Biso	1.000000	H
O2083	1.0	0.115346	0.131443	0.411769	Biso	1.000000	O
H1801	1.0	0.131140	0.113630	0.426095	Biso	1.000000	H
H1802	1.0	0.120730	0.158172	0.419264	Biso	1.000000	H

O2084	1.0	0.406280	0.810071	0.928631	Biso	1.000000	O
H1803	1.0	0.408358	0.838326	0.930403	Biso	1.000000	H
H1804	1.0	0.428603	0.798059	0.939578	Biso	1.000000	H
O2085	1.0	0.984219	0.826402	0.764312	Biso	1.000000	O
H1805	1.0	0.990357	0.812978	0.787546	Biso	1.000000	H
H1806	1.0	0.004291	0.844718	0.758187	Biso	1.000000	H
O2086	1.0	0.846669	0.139549	0.315062	Biso	1.000000	O
H1807	1.0	0.870649	0.128782	0.306664	Biso	1.000000	H
H1808	1.0	0.847266	0.167842	0.312802	Biso	1.000000	H
O2087	1.0	0.212459	0.573354	0.753126	Biso	1.000000	O
H1809	1.0	0.202386	0.548328	0.761254	Biso	1.000000	H
H1810	1.0	0.233439	0.580754	0.769232	Biso	1.000000	H
O2088	1.0	0.890581	0.726458	0.016870	Biso	1.000000	O
H1811	1.0	0.875653	0.750297	0.016491	Biso	1.000000	H
H1812	1.0	0.901070	0.721769	0.992081	Biso	1.000000	H
O2089	1.0	0.301167	0.534853	0.784256	Biso	1.000000	O
H1813	1.0	0.291551	0.560791	0.789918	Biso	1.000000	H
H1814	1.0	0.280204	0.516563	0.782695	Biso	1.000000	H
O2090	1.0	0.739401	0.802449	0.711833	Biso	1.000000	O
H1815	1.0	0.760938	0.798252	0.728280	Biso	1.000000	H
H1816	1.0	0.717337	0.809116	0.726768	Biso	1.000000	H
O2091	1.0	0.286115	0.310370	0.342409	Biso	1.000000	O
H1817	1.0	0.312626	0.315158	0.347912	Biso	1.000000	H
H1818	1.0	0.277888	0.286826	0.355280	Biso	1.000000	H
O2092	1.0	0.033996	0.886965	0.194785	Biso	1.000000	O
H1819	1.0	0.038524	0.903949	0.216165	Biso	1.000000	H
H1820	1.0	0.058001	0.879851	0.183407	Biso	1.000000	H
O2093	1.0	0.963123	0.471570	0.894058	Biso	1.000000	O
H1821	1.0	0.981032	0.493109	0.893935	Biso	1.000000	H
H1822	1.0	0.946122	0.474316	0.915311	Biso	1.000000	H
O2094	1.0	0.635395	0.654624	0.967459	Biso	1.000000	O
H1823	1.0	0.617975	0.675110	0.975065	Biso	1.000000	H
H1824	1.0	0.626189	0.642337	0.944645	Biso	1.000000	H
O2095	1.0	0.593784	0.040871	0.297339	Biso	1.000000	O
H1825	1.0	0.574165	0.057464	0.307857	Biso	1.000000	H
H1826	1.0	0.592432	0.041692	0.270124	Biso	1.000000	H
O2096	1.0	0.161164	0.525315	0.688937	Biso	1.000000	O
H1827	1.0	0.139249	0.542381	0.687462	Biso	1.000000	H
H1828	1.0	0.183933	0.540311	0.683864	Biso	1.000000	H
O2097	1.0	0.051365	0.985353	0.593195	Biso	1.000000	O
H1829	1.0	0.072392	0.003571	0.594691	Biso	1.000000	H
H1830	1.0	0.029259	0.998724	0.583322	Biso	1.000000	H
O2098	1.0	0.018846	0.290904	0.684788	Biso	1.000000	O
H1831	1.0	0.992882	0.295300	0.692657	Biso	1.000000	H
H1832	1.0	0.035962	0.299471	0.704463	Biso	1.000000	H
O2099	1.0	0.975020	0.577676	0.583352	Biso	1.000000	O
H1833	1.0	0.977748	0.566062	0.558627	Biso	1.000000	H
H1834	1.0	0.984289	0.604404	0.582807	Biso	1.000000	H
O2100	1.0	0.341662	0.369434	0.681414	Biso	1.000000	O
H1835	1.0	0.348752	0.396371	0.676431	Biso	1.000000	H
H1836	1.0	0.314445	0.367792	0.684832	Biso	1.000000	H
O2101	1.0	0.513625	0.090657	0.792900	Biso	1.000000	O
H1837	1.0	0.523900	0.064721	0.797296	Biso	1.000000	H
H1838	1.0	0.531251	0.110184	0.802173	Biso	1.000000	H
O2102	1.0	0.167114	0.578338	0.361247	Biso	1.000000	O
H1839	1.0	0.161332	0.550699	0.363738	Biso	1.000000	H
H1840	1.0	0.152533	0.589308	0.340684	Biso	1.000000	H
O2103	1.0	0.542854	0.646341	0.915057	Biso	1.000000	O
H1841	1.0	0.529891	0.655274	0.937509	Biso	1.000000	H
H1842	1.0	0.527212	0.651589	0.893218	Biso	1.000000	H
O2104	1.0	0.491413	0.871735	0.870685	Biso	1.000000	O
H1843	1.0	0.508258	0.887993	0.885526	Biso	1.000000	H
H1844	1.0	0.491242	0.845365	0.880797	Biso	1.000000	H
H1845	1.0	0.870872	0.377484	0.064663	Biso	1.000000	H
H1846	1.0	0.711052	0.295530	0.966304	Biso	1.000000	H
H1847	1.0	0.865227	0.992938	0.124979	Biso	1.000000	H
H1848	1.0	0.886626	0.821890	0.305614	Biso	1.000000	H
H1849	1.0	0.857255	0.324861	0.080366	Biso	1.000000	H
H1850	1.0	0.984276	0.291302	0.096267	Biso	1.000000	H
O2105	1.0	0.875637	0.884755	0.332667	Biso	1.000000	O
H1851	1.0	0.877698	0.912849	0.336088	Biso	1.000000	H
H1852	1.0	0.900743	0.873598	0.329816	Biso	1.000000	H
O2106	1.0	0.030357	0.738030	0.781631	Biso	1.000000	O
H1853	1.0	0.019727	0.756999	0.798962	Biso	1.000000	H
H1854	1.0	0.050503	0.750195	0.767231	Biso	1.000000	H
O2107	1.0	0.770405	0.375993	0.764050	Biso	1.000000	O
H1855	1.0	0.783772	0.382928	0.741184	Biso	1.000000	H

H1856	1.0	0.743938	0.371223	0.758326	Biso	1.000000	H
O2108	1.0	0.862781	0.947462	0.250592	Biso	1.000000	O
H1857	1.0	0.867033	0.973186	0.261340	Biso	1.000000	H
H1858	1.0	0.850558	0.950177	0.226319	Biso	1.000000	H
O2109	1.0	0.244450	0.125594	0.595523	Biso	1.000000	O
H1859	1.0	0.256557	0.150571	0.590622	Biso	1.000000	H
H1860	1.0	0.251814	0.116279	0.620215	Biso	1.000000	H
O2110	1.0	0.272665	0.616703	0.055695	Biso	1.000000	O
H1861	1.0	0.297701	0.615024	0.044572	Biso	1.000000	H
H1862	1.0	0.259478	0.592059	0.052180	Biso	1.000000	H
O2111	1.0	0.072063	0.525099	0.217670	Biso	1.000000	O
H1863	1.0	0.099328	0.522745	0.220226	Biso	1.000000	H
H1864	1.0	0.066136	0.547496	0.201979	Biso	1.000000	H
O2112	1.0	0.759715	0.353810	0.068122	Biso	1.000000	O
H1865	1.0	0.749893	0.327295	0.067514	Biso	1.000000	H
H1866	1.0	0.740981	0.371615	0.057843	Biso	1.000000	H
O2113	1.0	0.699087	0.438893	0.507620	Biso	1.000000	O
H1867	1.0	0.678801	0.420123	0.503956	Biso	1.000000	H
H1868	1.0	0.710967	0.434375	0.531813	Biso	1.000000	H
O2114	1.0	0.347384	0.547038	0.582687	Biso	1.000000	O
H1869	1.0	0.345339	0.522353	0.569366	Biso	1.000000	H
H1870	1.0	0.372638	0.549560	0.593159	Biso	1.000000	H
O2115	1.0	0.897957	0.323694	0.648855	Biso	1.000000	O
H1871	1.0	0.914547	0.329448	0.627836	Biso	1.000000	H
H1872	1.0	0.872804	0.317595	0.639568	Biso	1.000000	H
O2116	1.0	0.167156	0.277135	0.132899	Biso	1.000000	O
H1873	1.0	0.190750	0.264637	0.125729	Biso	1.000000	H
H1874	1.0	0.165749	0.278887	0.160068	Biso	1.000000	H
O2117	1.0	0.783434	0.584971	0.060737	Biso	1.000000	O
H1875	1.0	0.782101	0.584321	0.087955	Biso	1.000000	H
H1876	1.0	0.768352	0.606619	0.051388	Biso	1.000000	H
O2118	1.0	0.144542	0.669487	0.865151	Biso	1.000000	O
H1877	1.0	0.162644	0.677541	0.846156	Biso	1.000000	H
H1878	1.0	0.121740	0.685171	0.862935	Biso	1.000000	H
O2119	1.0	0.640834	0.041945	0.168800	Biso	1.000000	O
H1879	1.0	0.623878	0.043659	0.190188	Biso	1.000000	H
H1880	1.0	0.627228	0.031400	0.147390	Biso	1.000000	H
O2120	1.0	0.948631	0.764362	0.734860	Biso	1.000000	O
H1881	1.0	0.960935	0.788193	0.743277	Biso	1.000000	H
H1882	1.0	0.967461	0.746603	0.724682	Biso	1.000000	H
O2121	1.0	0.593001	0.356019	0.180868	Biso	1.000000	O
H1883	1.0	0.615337	0.341585	0.188625	Biso	1.000000	H
H1884	1.0	0.600572	0.381057	0.170423	Biso	1.000000	H
O2122	1.0	0.784659	0.813189	0.154458	Biso	1.000000	O
H1885	1.0	0.782362	0.825882	0.178733	Biso	1.000000	H
H1886	1.0	0.807078	0.796766	0.154257	Biso	1.000000	H
O2123	1.0	0.432766	0.129571	0.515012	Biso	1.000000	O
H1887	1.0	0.410880	0.137195	0.500241	Biso	1.000000	H
H1888	1.0	0.455317	0.130770	0.499478	Biso	1.000000	H
O2124	1.0	0.271555	0.392739	0.851910	Biso	1.000000	O
H1889	1.0	0.280037	0.392149	0.825990	Biso	1.000000	H
H1890	1.0	0.293244	0.390399	0.868496	Biso	1.000000	H
O2125	1.0	0.481838	0.835520	0.805253	Biso	1.000000	O
H1891	1.0	0.488013	0.849389	0.828236	Biso	1.000000	H
H1892	1.0	0.502127	0.839010	0.787176	Biso	1.000000	H
O2126	1.0	0.759275	0.104206	0.488785	Biso	1.000000	O
H1893	1.0	0.735395	0.092775	0.480948	Biso	1.000000	H
H1894	1.0	0.768556	0.121548	0.469263	Biso	1.000000	H
O2127	1.0	0.085683	0.662717	0.671271	Biso	1.000000	O
H1895	1.0	0.088220	0.672282	0.696814	Biso	1.000000	H
H1896	1.0	0.106159	0.673026	0.656021	Biso	1.000000	H
O2128	1.0	0.083665	0.753725	0.661202	Biso	1.000000	O
H1897	1.0	0.056554	0.753100	0.656781	Biso	1.000000	H
H1898	1.0	0.095411	0.731257	0.649280	Biso	1.000000	H
O2129	1.0	0.202881	0.018669	0.662347	Biso	1.000000	O
H1899	1.0	0.229525	0.015365	0.656476	Biso	1.000000	H
H1900	1.0	0.197261	0.046318	0.665095	Biso	1.000000	H
O2130	1.0	0.048749	0.852959	0.601399	Biso	1.000000	O
H1901	1.0	0.055226	0.833669	0.582455	Biso	1.000000	H
H1902	1.0	0.040759	0.876991	0.589234	Biso	1.000000	H
O2131	1.0	0.457812	0.907763	0.188144	Biso	1.000000	O

H1903	1.0	0.481264	0.899732	0.176208	Biso	1.000000	H
H1904	1.0	0.450241	0.933150	0.178518	Biso	1.000000	H
O2132	1.0	0.803646	0.725265	0.685513	Biso	1.000000	O
H1905	1.0	0.792087	0.723391	0.710179	Biso	1.000000	H
H1906	1.0	0.785566	0.717038	0.666564	Biso	1.000000	H
O2133	1.0	0.845768	0.057736	0.255315	Biso	1.000000	O
H1907	1.0	0.832484	0.081758	0.249173	Biso	1.000000	H
H1908	1.0	0.835630	0.046981	0.278452	Biso	1.000000	H
O2134	1.0	0.713863	0.052295	0.176575	Biso	1.000000	O
H1909	1.0	0.687971	0.044300	0.171620	Biso	1.000000	H
H1910	1.0	0.730965	0.031106	0.170131	Biso	1.000000	H
O2135	1.0	0.448224	0.009068	0.790913	Biso	1.000000	O
H1911	1.0	0.445264	0.026753	0.812037	Biso	1.000000	H
H1912	1.0	0.449268	0.024046	0.767779	Biso	1.000000	H
O2136	1.0	0.375142	0.419759	0.211851	Biso	1.000000	O
H1913	1.0	0.372572	0.422091	0.184806	Biso	1.000000	H
H1914	1.0	0.391990	0.440046	0.221044	Biso	1.000000	H
O2137	1.0	0.072695	0.416465	0.218071	Biso	1.000000	O
H1915	1.0	0.089784	0.414702	0.239350	Biso	1.000000	H
H1916	1.0	0.063516	0.390533	0.211705	Biso	1.000000	H
O2138	1.0	0.132687	0.602890	0.301070	Biso	1.000000	O
H1917	1.0	0.150460	0.615950	0.284487	Biso	1.000000	H
H1918	1.0	0.117742	0.584301	0.286757	Biso	1.000000	H
O2139	1.0	0.739627	0.525797	0.813474	Biso	1.000000	O
H1919	1.0	0.745447	0.505544	0.795260	Biso	1.000000	H
H1920	1.0	0.755409	0.522367	0.835544	Biso	1.000000	H
O2140	1.0	0.576287	0.258671	0.047233	Biso	1.000000	O
H1921	1.0	0.599681	0.257735	0.061505	Biso	1.000000	H
H1922	1.0	0.558974	0.277064	0.058914	Biso	1.000000	H
O2141	1.0	0.699395	0.188082	0.675921	Biso	1.000000	O
H1923	1.0	0.709151	0.161700	0.678772	Biso	1.000000	H
H1924	1.0	0.672054	0.187172	0.673337	Biso	1.000000	H
O2142	1.0	0.486136	0.139736	0.463528	Biso	1.000000	O
H1925	1.0	0.497049	0.163378	0.474058	Biso	1.000000	H
H1926	1.0	0.503960	0.118300	0.466183	Biso	1.000000	H
O2143	1.0	0.012625	0.233447	0.384238	Biso	1.000000	O
H1927	1.0	0.038651	0.239361	0.390895	Biso	1.000000	H
H1928	1.0	0.995642	0.246945	0.401307	Biso	1.000000	H
O2144	1.0	0.557306	0.381128	0.346443	Biso	1.000000	O
H1929	1.0	0.538875	0.387491	0.327169	Biso	1.000000	H
H1930	1.0	0.580945	0.373387	0.334695	Biso	1.000000	H
O2145	1.0	0.700571	0.367628	0.731505	Biso	1.000000	O
H1931	1.0	0.676567	0.357220	0.740235	Biso	1.000000	H
H1932	1.0	0.712740	0.348653	0.715213	Biso	1.000000	H
O2146	1.0	0.962359	0.826405	0.023522	Biso	1.000000	O
H1933	1.0	0.950514	0.842540	0.042630	Biso	1.000000	H
H1934	1.0	0.964766	0.799794	0.032732	Biso	1.000000	H
O2147	1.0	0.810722	0.734832	0.827934	Biso	1.000000	O
H1935	1.0	0.809268	0.711566	0.843495	Biso	1.000000	H
H1936	1.0	0.785577	0.741642	0.819092	Biso	1.000000	H
O2148	1.0	0.044608	0.438646	0.877354	Biso	1.000000	O
H1937	1.0	0.043798	0.411308	0.870019	Biso	1.000000	H
H1938	1.0	0.041653	0.440699	0.904383	Biso	1.000000	H
O2149	1.0	0.970568	0.400531	0.866064	Biso	1.000000	O
H1939	1.0	0.971300	0.426248	0.877603	Biso	1.000000	H
H1940	1.0	0.983168	0.401486	0.841857	Biso	1.000000	H
O2150	1.0	0.438413	0.252694	0.732628	Biso	1.000000	O
H1941	1.0	0.417308	0.246113	0.748903	Biso	1.000000	H
H1942	1.0	0.429111	0.266587	0.710718	Biso	1.000000	H
O2151	1.0	0.856073	0.487504	0.825731	Biso	1.000000	O
H1943	1.0	0.882741	0.493814	0.828335	Biso	1.000000	H
H1944	1.0	0.846653	0.497965	0.802175	Biso	1.000000	H
O2152	1.0	0.203130	0.380137	0.167716	Biso	1.000000	O
H1945	1.0	0.198226	0.387012	0.193711	Biso	1.000000	H
H1946	1.0	0.212993	0.402786	0.154502	Biso	1.000000	H
O2153	1.0	0.661356	0.847759	0.594868	Biso	1.000000	O
H1947	1.0	0.651383	0.872397	0.585606	Biso	1.000000	H
H1948	1.0	0.683352	0.840353	0.580160	Biso	1.000000	H
O2154	1.0	0.544162	0.413137	0.770625	Biso	1.000000	O
H1949	1.0	0.534390	0.439271	0.766168	Biso	1.000000	H
H1950	1.0	0.571159	0.414543	0.775534	Biso	1.000000	H
H1951	1.0	0.465290	0.158242	0.428020	Biso	1.000000	H
H1952	1.0	0.512540	0.250740	0.413756	Biso	1.000000	H
H1953	1.0	0.606444	0.172314	0.385877	Biso	1.000000	H
H1954	1.0	0.694003	0.527226	0.338190	Biso	1.000000	H
H1955	1.0	0.579791	0.457564	0.360895	Biso	1.000000	H
H1956	1.0	0.706787	0.471499	0.368266	Biso	1.000000	H
H1957	1.0	0.672557	0.438925	0.322271	Biso	1.000000	H
H1958	1.0	0.903489	0.858448	0.087614	Biso	1.000000	H
H1959	1.0	0.562879	0.952610	0.220203	Biso	1.000000	H
H1960	1.0	0.570807	0.993714	0.138475	Biso	1.000000	H
H1961	1.0	0.525579	0.008417	0.195553	Biso	1.000000	H
H1962	1.0	0.951761	0.874822	0.266523	Biso	1.000000	H
H1963	1.0	0.586870	0.110187	0.153048	Biso	1.000000	H

```
#======================================================================

# CRYSTAL DATA

#----------------------------------------------------------------------

data_VESTA_phase_1


_chemical_name_common                  'wc31'
_cell_length_a                         40.546120
_cell_length_b                         36.997780
_cell_length_c                         42.379050
_cell_angle_alpha                      90
_cell_angle_beta                       90
_cell_angle_gamma                      90
_space_group_name_H-M_alt              'P 1'
_space_group_IT_number                 1

loop_
_space_group_symop_operation_xyz
   'x, y, z'

loop_
   _atom_site_label
   _atom_site_occupancy
   _atom_site_fract_x
   _atom_site_fract_y
   _atom_site_fract_z
   _atom_site_adp_type
   _atom_site_B_iso_or_equiv
   _atom_site_type_symbol
   Si1        1.0     0.534377     0.124234     0.622978    Biso  1.000000 Si
   Al1        1.0     0.765166     0.085466     0.773344    Biso  1.000000 Al
   Si2        1.0     0.796429     0.307413     0.899304    Biso  1.000000 Si
   Al2        1.0     0.456441     0.707142     0.771115    Biso  1.000000 Al
   Al3        1.0     0.132581     0.694452     0.337653    Biso  1.000000 Al
   Si3        1.0     0.696587     0.294829     0.449071    Biso  1.000000 Si
   Si4        1.0     0.797973     0.289844     0.722633    Biso  1.000000 Si
   Si5        1.0     0.637617     0.916655     0.109349    Biso  1.000000 Si
   Si6        1.0     0.591820     0.069696     0.636593    Biso  1.000000 Si
   Al4        1.0     0.733878     0.327809     0.706790    Biso  1.000000 Al
   Si7        1.0     0.477466     0.836056     0.496030    Biso  1.000000 Si
   Si8        1.0     0.559203     0.984799     0.914523    Biso  1.000000 Si
   Si9        1.0     0.589497     0.263833     0.814690    Biso  1.000000 Si
  Si10        1.0     0.221168     0.524437     0.237544    Biso  1.000000 Si
  Si11        1.0     0.347927     0.110868     0.462653    Biso  1.000000 Si
  Si12        1.0     0.608273     0.796913     0.884907    Biso  1.000000 Si
  Si13        1.0     0.119220     0.253986     0.384272    Biso  1.000000 Si
  Si14        1.0     0.385119     0.247973     0.471011    Biso  1.000000 Si
  Si15        1.0     0.145814     0.452895     0.518980    Biso  1.000000 Si
  Si16        1.0     0.900550     0.002938     0.240373    Biso  1.000000 Si
  Si17        1.0     0.691509     0.026050     0.161407    Biso  1.000000 Si
  Si18        1.0     0.702812     0.772538     0.702951    Biso  1.000000 Si
  Si19        1.0     0.651781     0.803729     0.823869    Biso  1.000000 Si
  Si20        1.0     0.223209     0.708537     0.455298    Biso  1.000000 Si
  Si21        1.0     0.915989     0.507903     0.153428    Biso  1.000000 Si
  Si22        1.0     0.831431     0.476937     0.447896    Biso  1.000000 Si
  Si23        1.0     0.363334     0.913858     0.685487    Biso  1.000000 Si
  Si24        1.0     0.703653     0.466144     0.798241    Biso  1.000000 Si
  Si25        1.0     0.870695     0.495854     0.094818    Biso  1.000000 Si
  Si26        1.0     0.823095     0.385020     0.604301    Biso  1.000000 Si
  Si27        1.0     0.226443     0.876523     0.556211    Biso  1.000000 Si
  Si28        1.0     0.188637     0.547041     0.116474    Biso  1.000000 Si
```

Si29	1.0	0.739681	0.429566	0.403027	Biso	1.000000	Si
Si30	1.0	0.638256	0.180694	0.586360	Biso	1.000000	Si
Al5	1.0	0.554605	0.152805	0.509124	Biso	1.000000	Al
Si31	1.0	0.766436	0.879142	0.521888	Biso	1.000000	Si
Si32	1.0	0.708258	0.535616	0.314939	Biso	1.000000	Si
Si33	1.0	0.924346	0.852494	0.040114	Biso	1.000000	Si
Si34	1.0	0.255351	0.766668	0.789758	Biso	1.000000	Si
Si35	1.0	0.270344	0.684128	0.793855	Biso	1.000000	Si
Al6	1.0	0.517492	0.956910	0.969386	Biso	1.000000	Al
Si36	1.0	0.029838	0.147168	0.974972	Biso	1.000000	Si
Al7	1.0	0.667881	0.177403	0.113918	Biso	1.000000	Al
Al8	1.0	0.990922	0.228745	0.848688	Biso	1.000000	Al
Si37	1.0	0.812953	0.705796	0.574793	Biso	1.000000	Si
Si38	1.0	0.022963	0.280476	0.163944	Biso	1.000000	Si
Si39	1.0	0.077529	0.422447	0.957492	Biso	1.000000	Si
Si40	1.0	0.921301	0.549336	0.261681	Biso	1.000000	Si
Al9	1.0	0.513164	0.167368	0.680936	Biso	1.000000	Al
Si41	1.0	0.856708	0.405565	0.479048	Biso	1.000000	Si
Si42	1.0	0.134287	0.781460	0.615552	Biso	1.000000	Si
Si43	1.0	0.776037	0.769730	0.543091	Biso	1.000000	Si
Si44	1.0	0.645865	0.856603	0.920557	Biso	1.000000	Si
Si45	1.0	0.945645	0.816165	0.325031	Biso	1.000000	Si
Si46	1.0	0.979483	0.704999	0.172513	Biso	1.000000	Si
Al10	1.0	0.063531	0.788764	0.763061	Biso	1.000000	Al
Si47	1.0	0.667968	0.852711	0.308308	Biso	1.000000	Si
Si48	1.0	0.534848	0.547068	0.067879	Biso	1.000000	Si
Si49	1.0	0.042283	0.625905	0.376948	Biso	1.000000	Si
Si50	1.0	0.572679	0.208893	0.702529	Biso	1.000000	Si
Si51	1.0	0.676047	0.972861	0.976713	Biso	1.000000	Si
Si52	1.0	0.615255	0.401557	0.943239	Biso	1.000000	Si
Si53	1.0	0.219962	0.761711	0.549073	Biso	1.000000	Si
Si54	1.0	0.621551	0.546660	0.742681	Biso	1.000000	Si
Si55	1.0	0.088705	0.735755	0.217097	Biso	1.000000	Si
Si56	1.0	0.703665	0.383718	0.061499	Biso	1.000000	Si
Si57	1.0	0.290795	0.539351	0.608945	Biso	1.000000	Si
Si58	1.0	0.279610	0.225705	0.427313	Biso	1.000000	Si
Si59	1.0	0.215699	0.211298	0.330000	Biso	1.000000	Si
Si60	1.0	0.001551	0.087506	0.605025	Biso	1.000000	Si
Si61	1.0	0.633259	0.981900	0.264489	Biso	1.000000	Si
Si62	1.0	0.935825	0.968055	0.426307	Biso	1.000000	Si
Si63	1.0	0.733471	0.200790	0.679027	Biso	1.000000	Si
Si64	1.0	0.540288	0.470569	0.573564	Biso	1.000000	Si
Si65	1.0	0.481980	0.265120	0.977288	Biso	1.000000	Si
Si66	1.0	0.059611	0.689644	0.602378	Biso	1.000000	Si
Si67	1.0	0.791309	0.704115	0.268574	Biso	1.000000	Si
Si68	1.0	0.889215	0.759994	0.336216	Biso	1.000000	Si
Si69	1.0	0.902407	0.761581	0.655841	Biso	1.000000	Si
Si70	1.0	0.701034	0.235823	0.307761	Biso	1.000000	Si
Si71	1.0	0.078785	0.734912	0.817442	Biso	1.000000	Si
Si72	1.0	0.607370	0.488789	0.854033	Biso	1.000000	Si
Si73	1.0	0.808262	0.296599	0.826939	Biso	1.000000	Si
Si74	1.0	0.287941	0.076220	0.427735	Biso	1.000000	Si
Si75	1.0	0.177223	0.477266	0.581856	Biso	1.000000	Si
Si76	1.0	0.533765	0.072612	0.829745	Biso	1.000000	Si
Al11	1.0	0.831959	0.894316	0.002093	Biso	1.000000	Al
Si77	1.0	0.988682	0.529170	0.291809	Biso	1.000000	Si
Si78	1.0	0.158783	0.784128	0.508825	Biso	1.000000	Si
Si79	1.0	0.108265	0.055803	0.667382	Biso	1.000000	Si
Si80	1.0	0.804703	0.963999	0.976966	Biso	1.000000	Si
Si81	1.0	0.205383	0.659653	0.641482	Biso	1.000000	Si
Si82	1.0	0.500095	0.404678	0.972380	Biso	1.000000	Si
Si83	1.0	0.628790	0.015065	0.931807	Biso	1.000000	Si
Si84	1.0	0.615810	0.713616	0.895315	Biso	1.000000	Si
Si85	1.0	0.626950	0.423139	0.707088	Biso	1.000000	Si
Si86	1.0	0.734342	0.306529	0.819051	Biso	1.000000	Si
Si87	1.0	0.930496	0.366200	0.389138	Biso	1.000000	Si
Si88	1.0	0.712048	0.581375	0.475525	Biso	1.000000	Si
Si89	1.0	0.077813	0.821601	0.986693	Biso	1.000000	Si
Al12	1.0	0.312353	0.753445	0.355631	Biso	1.000000	Al
Al13	1.0	0.702541	0.966147	0.660836	Biso	1.000000	Al
Si90	1.0	0.179454	0.605642	0.401368	Biso	1.000000	Si
Si91	1.0	0.686830	0.318097	0.761374	Biso	1.000000	Si
Si92	1.0	0.023059	0.867414	0.952853	Biso	1.000000	Si
Si93	1.0	0.881811	0.626097	0.667224	Biso	1.000000	Si
Si94	1.0	0.062897	0.873566	0.617454	Biso	1.000000	Si
Si95	1.0	0.475932	0.965728	0.756893	Biso	1.000000	Si
Al14	1.0	0.232419	0.636741	0.751879	Biso	1.000000	Al

Al15	1.0	0.685486	0.016197	0.037437	Biso	1.000000	Al
Si96	1.0	0.558327	0.479375	0.106320	Biso	1.000000	Si
Si97	1.0	0.127068	0.213435	0.016534	Biso	1.000000	Si
Si98	1.0	0.084933	0.256353	0.202604	Biso	1.000000	Si
Si99	1.0	0.545712	0.704369	0.334486	Biso	1.000000	Si
Si100	1.0	0.198387	0.676230	0.241180	Biso	1.000000	Si
Si101	1.0	0.890522	0.242402	0.503006	Biso	1.000000	Si
Si102	1.0	0.036390	0.901673	0.205531	Biso	1.000000	Si
Si103	1.0	0.120947	0.795442	0.129175	Biso	1.000000	Si
Si104	1.0	0.867025	0.715475	0.277363	Biso	1.000000	Si
Si105	1.0	0.103993	0.716219	0.045485	Biso	1.000000	Si
Si106	1.0	0.592974	0.873396	0.220751	Biso	1.000000	Si
Si107	1.0	0.858294	0.511420	0.626114	Biso	1.000000	Si
Si108	1.0	0.557831	0.456527	0.954571	Biso	1.000000	Si
Si109	1.0	0.960272	0.251530	0.199308	Biso	1.000000	Si
Si110	1.0	0.793279	0.237749	0.940893	Biso	1.000000	Si
Al16	1.0	0.002096	0.920644	0.634250	Biso	1.000000	Al
Al17	1.0	0.535240	0.118804	0.064466	Biso	1.000000	Al
Si111	1.0	0.275506	0.182926	0.738870	Biso	1.000000	Si
Si112	1.0	0.531410	0.995025	0.801752	Biso	1.000000	Si
Si113	1.0	0.753729	0.166545	0.789744	Biso	1.000000	Si
Al18	1.0	0.607387	0.580346	0.141361	Biso	1.000000	Al
Si114	1.0	0.824449	0.517928	0.330102	Biso	1.000000	Si
Si115	1.0	0.690801	0.705507	0.744196	Biso	1.000000	Si
Si116	1.0	0.571011	0.277338	0.514823	Biso	1.000000	Si
Si117	1.0	0.561425	0.183518	0.576660	Biso	1.000000	Si
Si118	1.0	0.783325	0.211067	0.734712	Biso	1.000000	Si
Si119	1.0	0.814818	0.026273	0.026372	Biso	1.000000	Si
Si120	1.0	0.759000	0.391699	0.014238	Biso	1.000000	Si
Si121	1.0	0.393436	0.044994	0.478578	Biso	1.000000	Si
Si122	1.0	0.122145	0.954751	0.005691	Biso	1.000000	Si
Al19	1.0	0.585647	0.431693	0.052309	Biso	1.000000	Al
Si123	1.0	0.145006	0.197347	0.855958	Biso	1.000000	Si
Si124	1.0	0.030634	0.191904	0.794680	Biso	1.000000	Si
Si125	1.0	0.743336	0.620605	0.586679	Biso	1.000000	Si
Si126	1.0	0.928011	0.898251	0.467132	Biso	1.000000	Si
Si127	1.0	0.266003	0.689355	0.340012	Biso	1.000000	Si
Si128	1.0	0.684112	0.963989	0.210754	Biso	1.000000	Si
Si129	1.0	0.452268	0.713875	0.575284	Biso	1.000000	Si
Si130	1.0	0.847830	0.460460	0.377000	Biso	1.000000	Si
Si131	1.0	0.011633	0.526542	0.937716	Biso	1.000000	Si
Si132	1.0	0.484865	0.371584	0.139497	Biso	1.000000	Si
Si133	1.0	0.376685	0.876482	0.750029	Biso	1.000000	Si
Si134	1.0	0.519786	0.391121	0.588991	Biso	1.000000	Si
Al20	1.0	0.949821	0.827693	0.144131	Biso	1.000000	Al
Si135	1.0	0.032209	0.632849	0.076989	Biso	1.000000	Si
Si136	1.0	0.960525	0.667494	0.725516	Biso	1.000000	Si
Si137	1.0	0.126279	0.158973	0.073136	Biso	1.000000	Si
Si138	1.0	0.223082	0.233368	0.771615	Biso	1.000000	Si
Si139	1.0	0.812726	0.400308	0.066924	Biso	1.000000	Si
Si140	1.0	0.176608	0.655780	0.962797	Biso	1.000000	Si
Si141	1.0	0.630699	0.737898	0.781490	Biso	1.000000	Si
Si142	1.0	0.857562	0.703315	0.684966	Biso	1.000000	Si
Si143	1.0	0.251526	0.465393	0.601915	Biso	1.000000	Si
Si144	1.0	0.603962	0.092425	0.045003	Biso	1.000000	Si
Si145	1.0	0.038014	0.570459	0.732695	Biso	1.000000	Si
Si146	1.0	0.666661	0.020251	0.761520	Biso	1.000000	Si
Si147	1.0	0.113972	0.742688	0.393916	Biso	1.000000	Si
Si148	1.0	0.157541	0.997685	0.398803	Biso	1.000000	Si
Si149	1.0	0.973159	0.132969	0.255412	Biso	1.000000	Si
Si150	1.0	0.866053	0.003867	0.173750	Biso	1.000000	Si
Si151	1.0	0.298174	0.564880	0.830861	Biso	1.000000	Si
Si152	1.0	0.651865	0.999520	0.101868	Biso	1.000000	Si
Si153	1.0	0.807690	0.275652	0.649741	Biso	1.000000	Si
Si154	1.0	0.229126	0.168913	0.037419	Biso	1.000000	Si
Al21	1.0	0.638983	0.292672	0.401578	Biso	1.000000	Al
Si155	1.0	0.821299	0.576186	0.657783	Biso	1.000000	Si
Si156	1.0	0.041775	0.835093	0.439958	Biso	1.000000	Si
Al22	1.0	0.858797	0.571483	0.762341	Biso	1.000000	Al
Si157	1.0	0.629582	0.539868	0.971343	Biso	1.000000	Si
Si158	1.0	0.008410	0.304902	0.874430	Biso	1.000000	Si
Si159	1.0	0.861436	0.135062	0.133987	Biso	1.000000	Si
Si160	1.0	0.443214	0.050041	0.905041	Biso	1.000000	Si
Si161	1.0	0.512880	0.083378	0.503716	Biso	1.000000	Si
Al23	1.0	0.749315	0.495211	0.267415	Biso	1.000000	Al
Si162	1.0	0.190407	0.406308	0.119384	Biso	1.000000	Si
Si163	1.0	0.627922	0.373176	0.767006	Biso	1.000000	Si
Si164	1.0	0.859942	0.014886	0.665308	Biso	1.000000	Si
Si165	1.0	0.075600	0.302748	0.427740	Biso	1.000000	Si
Al24	1.0	0.924106	0.584813	0.170940	Biso	1.000000	Al
Si166	1.0	0.856462	0.024743	0.501054	Biso	1.000000	Si
Si167	1.0	0.147412	0.476458	0.121845	Biso	1.000000	Si

Si168	1.0	0.945183	0.373017	0.173954	Biso	1.000000	Si
Si169	1.0	0.891599	0.107730	0.070402	Biso	1.000000	Si
Al25	1.0	0.323562	0.787301	0.008909	Biso	1.000000	Al
Si170	1.0	0.898621	0.688358	0.369584	Biso	1.000000	Si
Si171	1.0	0.761202	0.210493	0.162862	Biso	1.000000	Si
Si172	1.0	0.968965	0.678422	0.652693	Biso	1.000000	Si
Si173	1.0	0.261169	0.295099	0.104570	Biso	1.000000	Si
Si174	1.0	0.002820	0.566845	0.410040	Biso	1.000000	Si
Si175	1.0	0.665289	0.842718	0.018140	Biso	1.000000	Si
Al26	1.0	0.562582	0.333571	0.785027	Biso	1.000000	Al
Al27	1.0	0.034863	0.652953	0.730689	Biso	1.000000	Al
Si176	1.0	0.645906	0.634919	0.182577	Biso	1.000000	Si
Al28	1.0	0.838162	0.599447	0.337426	Biso	1.000000	Al
Si177	1.0	0.682729	0.437307	0.943607	Biso	1.000000	Si
Si178	1.0	0.992288	0.435172	0.614610	Biso	1.000000	Si
Si179	1.0	0.514335	0.330709	0.081790	Biso	1.000000	Si
Si180	1.0	0.559703	0.189206	0.826772	Biso	1.000000	Si
Si181	1.0	0.141889	0.296529	0.705877	Biso	1.000000	Si
Si182	1.0	0.403146	0.304880	0.675737	Biso	1.000000	Si
Si183	1.0	0.893661	0.234367	0.745227	Biso	1.000000	Si
Si184	1.0	0.137635	0.889889	0.287534	Biso	1.000000	Si
Si185	1.0	0.972668	0.170056	0.188862	Biso	1.000000	Si
Si186	1.0	0.165121	0.241449	0.281240	Biso	1.000000	Si
Si187	1.0	0.256678	0.093710	0.059300	Biso	1.000000	Si
Si188	1.0	0.351970	0.105046	0.841774	Biso	1.000000	Si
Si189	1.0	0.094194	0.481769	0.380407	Biso	1.000000	Si
Al29	1.0	0.748718	0.776403	0.644866	Biso	1.000000	Al
Si190	1.0	0.787984	0.179299	0.560664	Biso	1.000000	Si
Si191	1.0	0.062198	0.767416	0.884017	Biso	1.000000	Si
Al30	1.0	0.943821	0.472045	0.574444	Biso	1.000000	Al
Si192	1.0	0.350433	0.140798	0.105328	Biso	1.000000	Si
Si193	1.0	0.858094	0.234671	0.810615	Biso	1.000000	Si
Si194	1.0	0.731091	0.661802	0.650069	Biso	1.000000	Si
Si195	1.0	0.060464	0.359378	0.847301	Biso	1.000000	Si
Si196	1.0	0.126955	0.653264	0.495526	Biso	1.000000	Si
Si197	1.0	0.790860	0.196502	0.633270	Biso	1.000000	Si
Si198	1.0	0.745682	0.873639	0.793713	Biso	1.000000	Si
Si199	1.0	0.812586	0.633581	0.163680	Biso	1.000000	Si
Al31	1.0	0.871908	0.195482	0.285967	Biso	1.000000	Al
Al32	1.0	0.042276	0.437199	0.867591	Biso	1.000000	Al
Al33	1.0	0.411443	0.127108	0.063750	Biso	1.000000	Al
Si200	1.0	0.860625	0.196458	0.557643	Biso	1.000000	Si
Si201	1.0	0.753870	0.180588	0.285766	Biso	1.000000	Si
Si202	1.0	0.225740	0.906933	0.980015	Biso	1.000000	Si
Si203	1.0	0.918574	0.597701	0.727690	Biso	1.000000	Si
Si204	1.0	0.755426	0.407000	0.702387	Biso	1.000000	Si
Si205	1.0	0.045852	0.769168	0.036702	Biso	1.000000	Si
Si206	1.0	0.220842	0.858927	0.096581	Biso	1.000000	Si
Si207	1.0	0.965208	0.259943	0.513867	Biso	1.000000	Si
Al34	1.0	0.134056	0.442329	0.632905	Biso	1.000000	Al
Si208	1.0	0.549919	0.218342	0.466353	Biso	1.000000	Si
Si209	1.0	0.935904	0.855935	0.260350	Biso	1.000000	Si
Si210	1.0	0.396716	0.134577	0.740614	Biso	1.000000	Si
Si211	1.0	0.556502	0.450068	0.687233	Biso	1.000000	Si
Si212	1.0	0.640066	0.690050	0.962304	Biso	1.000000	Si
Si213	1.0	0.140815	0.361830	0.255327	Biso	1.000000	Si
Si214	1.0	0.572504	0.687747	0.568840	Biso	1.000000	Si
Si215	1.0	0.617973	0.374641	0.873167	Biso	1.000000	Si
Si216	1.0	0.936316	0.897405	0.658170	Biso	1.000000	Si
Si217	1.0	0.733700	0.581367	0.755104	Biso	1.000000	Si
Si218	1.0	0.028195	0.157307	0.571716	Biso	1.000000	Si
Si219	1.0	0.631049	0.366544	0.069859	Biso	1.000000	Si
Si220	1.0	0.572104	0.032125	0.008293	Biso	1.000000	Si
Si221	1.0	0.767417	0.957713	0.110782	Biso	1.000000	Si
Si222	1.0	0.180931	0.283458	0.492415	Biso	1.000000	Si
Si223	1.0	0.106393	0.083856	0.049076	Biso	1.000000	Si
Si224	1.0	0.475182	0.170715	0.911016	Biso	1.000000	Si
Si225	1.0	0.554665	0.240630	0.345079	Biso	1.000000	Si
Si226	1.0	0.752072	0.025379	0.068982	Biso	1.000000	Si
Si227	1.0	0.239651	0.565693	0.299391	Biso	1.000000	Si
Si228	1.0	0.764372	0.679300	0.203659	Biso	1.000000	Si
Si229	1.0	0.543352	0.374177	0.024527	Biso	1.000000	Si
Si230	1.0	0.456189	0.946246	0.641841	Biso	1.000000	Si
Si231	1.0	0.242511	0.763170	0.718475	Biso	1.000000	Si
Si232	1.0	0.824237	0.207787	0.120125	Biso	1.000000	Si

Si233	1.0	0.198013	0.412541	0.421083	Biso	1.000000	Si
Al35	1.0	0.807404	0.628213	0.551088	Biso	1.000000	Al
Al36	1.0	0.829359	0.852043	0.863002	Biso	1.000000	Al
Si234	1.0	0.503437	0.636073	0.665152	Biso	1.000000	Si
Al37	1.0	0.600274	0.152294	0.942436	Biso	1.000000	Al
Si235	1.0	0.821435	0.219487	0.047410	Biso	1.000000	Si
Si236	1.0	0.650711	0.282544	0.985734	Biso	1.000000	Si
Si237	1.0	0.142995	0.311986	0.197023	Biso	1.000000	Si
Si238	1.0	0.694091	0.631995	0.708577	Biso	1.000000	Si
Si239	1.0	0.792229	0.710318	0.645569	Biso	1.000000	Si
Si240	1.0	0.758595	0.231595	0.005245	Biso	1.000000	Si
Si241	1.0	0.626082	0.599909	0.687163	Biso	1.000000	Si
Si242	1.0	0.361206	0.417839	0.622572	Biso	1.000000	Si
Si243	1.0	0.110807	0.869333	0.424030	Biso	1.000000	Si
Si244	1.0	0.548230	0.512174	0.637747	Biso	1.000000	Si
Si245	1.0	0.959762	0.050808	0.260136	Biso	1.000000	Si
Si246	1.0	0.107193	0.192680	0.792720	Biso	1.000000	Si
Si247	1.0	0.061398	0.519466	0.786629	Biso	1.000000	Si
Si248	1.0	0.640123	0.199092	0.988882	Biso	1.000000	Si
Si249	1.0	0.764569	0.878061	0.085422	Biso	1.000000	Si
Si250	1.0	0.302399	0.808430	0.693298	Biso	1.000000	Si
Si251	1.0	0.507578	0.583454	0.556813	Biso	1.000000	Si
Si252	1.0	0.921566	0.310592	0.731937	Biso	1.000000	Si
Si253	1.0	0.975630	0.740025	0.559319	Biso	1.000000	Si
Si254	1.0	0.220790	0.019926	0.053618	Biso	1.000000	Si
Si255	1.0	0.189633	0.511071	0.798463	Biso	1.000000	Si
Si256	1.0	0.056471	0.163731	0.104504	Biso	1.000000	Si
Si257	1.0	0.196004	0.551387	0.686054	Biso	1.000000	Si
Si258	1.0	0.794458	0.394894	0.228162	Biso	1.000000	Si
Si259	1.0	0.666574	0.613767	0.979860	Biso	1.000000	Si
Si260	1.0	0.066827	0.426015	0.607645	Biso	1.000000	Si
Al38	1.0	0.035506	0.093786	0.073485	Biso	1.000000	Al
Si261	1.0	0.113580	0.324830	0.490091	Biso	1.000000	Si
Si262	1.0	0.930058	0.927872	0.870218	Biso	1.000000	Si
Si263	1.0	0.231898	0.472747	0.380778	Biso	1.000000	Si
Si264	1.0	0.987198	0.836420	0.207355	Biso	1.000000	Si
Si265	1.0	0.986912	0.464871	0.339559	Biso	1.000000	Si
Si266	1.0	0.714234	0.781766	0.862117	Biso	1.000000	Si
Si267	1.0	0.673759	0.476802	0.140790	Biso	1.000000	Si
Si268	1.0	0.144288	0.990041	0.525243	Biso	1.000000	Si
Si269	1.0	0.701643	0.271630	0.037678	Biso	1.000000	Si
Si270	1.0	0.371523	0.798048	0.725611	Biso	1.000000	Si
Si271	1.0	0.794619	0.403670	0.356756	Biso	1.000000	Si
Si272	1.0	0.426233	0.916331	0.793416	Biso	1.000000	Si
Si273	1.0	0.485992	0.553312	0.663113	Biso	1.000000	Si
Si274	1.0	0.982497	0.224977	0.579100	Biso	1.000000	Si
Si275	1.0	0.039178	0.763676	0.399815	Biso	1.000000	Si
Si276	1.0	0.813089	0.340826	0.456527	Biso	1.000000	Si
Si277	1.0	0.715999	0.066765	0.721045	Biso	1.000000	Si
Al39	1.0	0.655888	0.286598	0.195699	Biso	1.000000	Al
Si278	1.0	0.091887	0.860371	0.090673	Biso	1.000000	Si
Si279	1.0	0.802249	0.465006	0.112745	Biso	1.000000	Si
Al40	1.0	0.148248	0.079439	0.726203	Biso	1.000000	Al
Si280	1.0	0.148319	0.723108	0.458191	Biso	1.000000	Si
Al41	1.0	0.702435	0.546465	0.815202	Biso	1.000000	Al
Si281	1.0	0.223279	0.117017	0.411486	Biso	1.000000	Si
Al42	1.0	0.570654	0.287688	0.113868	Biso	1.000000	Al
Al43	1.0	0.716036	0.251264	0.869781	Biso	1.000000	Al
Si282	1.0	0.066117	0.687730	0.675572	Biso	1.000000	Si
Si283	1.0	0.667166	0.188203	0.518905	Biso	1.000000	Si
Si284	1.0	0.795953	0.206576	0.228663	Biso	1.000000	Si
Si285	1.0	0.657185	0.505609	0.909016	Biso	1.000000	Si
Si286	1.0	0.186932	0.276449	0.089893	Biso	1.000000	Si
Si287	1.0	0.152986	0.223316	0.740583	Biso	1.000000	Si
Si288	1.0	0.979869	0.335688	0.487290	Biso	1.000000	Si
Si289	1.0	0.048923	0.446932	0.752426	Biso	1.000000	Si
Si290	1.0	0.826925	0.397643	0.676959	Biso	1.000000	Si
Si291	1.0	0.214165	0.127620	0.338717	Biso	1.000000	Si
Si292	1.0	0.801889	0.812424	0.711186	Biso	1.000000	Si
Si293	1.0	0.911929	0.544681	0.580896	Biso	1.000000	Si
Si294	1.0	0.714742	0.066933	0.598890	Biso	1.000000	Si
Si295	1.0	0.130287	0.363235	0.945655	Biso	1.000000	Si
Si296	1.0	0.532031	0.808900	0.541881	Biso	1.000000	Si
Al44	1.0	0.132362	0.682254	0.101653	Biso	1.000000	Al

Si297	1.0	0.549098	0.191727	0.900057	Biso	1.000000	Si
Si298	1.0	0.956585	0.337918	0.672257	Biso	1.000000	Si
Si299	1.0	0.520114	0.055591	0.900658	Biso	1.000000	Si
Si300	1.0	0.019537	0.926937	0.138141	Biso	1.000000	Si
Si301	1.0	0.107408	0.690506	0.975966	Biso	1.000000	Si
Al45	1.0	0.662749	0.953732	0.796356	Biso	1.000000	Al
Si302	1.0	0.641393	0.875474	0.786431	Biso	1.000000	Si
Si303	1.0	0.943362	0.187985	0.302881	Biso	1.000000	Si
Si304	1.0	0.449338	0.667689	0.710344	Biso	1.000000	Si
Si305	1.0	0.881589	0.457965	0.678595	Biso	1.000000	Si
Si306	1.0	0.354453	0.869255	0.486238	Biso	1.000000	Si
Si307	1.0	0.887582	0.839183	0.558734	Biso	1.000000	Si
Si308	1.0	0.234414	0.855473	0.485771	Biso	1.000000	Si
Al46	1.0	0.926419	0.381696	0.458342	Biso	1.000000	Al
Al47	1.0	0.333603	0.468704	0.756659	Biso	1.000000	Al
Si309	1.0	0.156706	0.610072	0.076994	Biso	1.000000	Si
Si310	1.0	0.070897	0.445243	0.535935	Biso	1.000000	Si
Si311	1.0	0.905532	0.440010	0.196724	Biso	1.000000	Si
Si312	1.0	0.654625	0.244481	0.778038	Biso	1.000000	Si
Al48	1.0	0.955135	0.664334	0.329016	Biso	1.000000	Al
Al49	1.0	0.497036	0.456908	0.732133	Biso	1.000000	Al
Si313	1.0	0.500886	0.142951	0.804102	Biso	1.000000	Si
Si314	1.0	0.851904	0.345159	0.728442	Biso	1.000000	Si
Si315	1.0	0.708487	0.746310	0.520937	Biso	1.000000	Si
Si316	1.0	0.701423	0.082725	0.876612	Biso	1.000000	Si
Si317	1.0	0.876192	0.635798	0.204842	Biso	1.000000	Si
Si318	1.0	0.999413	0.577818	0.181307	Biso	1.000000	Si
Si319	1.0	0.730548	0.317073	0.093231	Biso	1.000000	Si
Si320	1.0	0.634256	0.903703	0.979183	Biso	1.000000	Si
Si321	1.0	0.778163	0.808873	0.829200	Biso	1.000000	Si
Si322	1.0	0.917963	0.914725	0.538924	Biso	1.000000	Si
Si323	1.0	0.980459	0.950553	0.568991	Biso	1.000000	Si
Si324	1.0	0.177412	0.719711	0.597859	Biso	1.000000	Si
Si325	1.0	0.258750	0.306586	0.761544	Biso	1.000000	Si
Si326	1.0	0.018950	0.238018	0.100365	Biso	1.000000	Si
Al50	1.0	0.824671	0.827377	0.519367	Biso	1.000000	Al
Al51	1.0	0.852148	0.281347	0.130822	Biso	1.000000	Al
Si327	1.0	0.924498	0.655810	0.428450	Biso	1.000000	Si
Si328	1.0	0.405304	0.812854	0.513324	Biso	1.000000	Si
Si329	1.0	0.208843	0.638252	0.027921	Biso	1.000000	Si
Si330	1.0	0.744044	0.030437	0.659229	Biso	1.000000	Si
Si331	1.0	0.793748	0.086997	0.231224	Biso	1.000000	Si
Si332	1.0	0.313179	0.716324	0.044604	Biso	1.000000	Si
Si333	1.0	0.703680	0.738263	0.965583	Biso	1.000000	Si
Si334	1.0	0.670769	0.848232	0.624308	Biso	1.000000	Si
Si335	1.0	0.509607	0.655218	0.142984	Biso	1.000000	Si
Si336	1.0	0.350833	0.532654	0.655362	Biso	1.000000	Si
Si337	1.0	0.789762	0.462301	0.184612	Biso	1.000000	Si
Si338	1.0	0.077977	0.373709	0.777193	Biso	1.000000	Si
Si339	1.0	0.076295	0.339169	0.714768	Biso	1.000000	Si
Si340	1.0	0.038666	0.495641	0.429734	Biso	1.000000	Si
Si341	1.0	0.926041	0.782620	0.518060	Biso	1.000000	Si
Si342	1.0	0.937640	0.939735	0.270584	Biso	1.000000	Si
Si343	1.0	0.644962	0.423863	0.824532	Biso	1.000000	Si
Si344	1.0	0.295535	0.205639	0.018939	Biso	1.000000	Si
Si345	1.0	0.419125	0.310200	0.960945	Biso	1.000000	Si
Si346	1.0	0.475792	0.016641	0.999738	Biso	1.000000	Si
Si347	1.0	0.040041	0.928496	0.525821	Biso	1.000000	Si
Si348	1.0	0.857631	0.291199	0.416140	Biso	1.000000	Si
Si349	1.0	0.124180	0.546708	0.821217	Biso	1.000000	Si
Si350	1.0	0.443611	0.065326	0.532018	Biso	1.000000	Si
Si351	1.0	0.644797	0.242661	0.256943	Biso	1.000000	Si
Si352	1.0	0.116233	0.620521	0.361905	Biso	1.000000	Si
Si353	1.0	0.969439	0.535686	0.715580	Biso	1.000000	Si
Si354	1.0	0.860733	0.406778	0.778009	Biso	1.000000	Si
Si355	1.0	0.605459	0.567075	0.622836	Biso	1.000000	Si
Si356	1.0	0.015316	0.758897	0.217724	Biso	1.000000	Si
Al52	1.0	0.187656	0.194129	0.094023	Biso	1.000000	Al
Si357	1.0	0.319412	0.866152	0.030162	Biso	1.000000	Si
Si358	1.0	0.684995	0.137808	0.693675	Biso	1.000000	Si
Si359	1.0	0.590434	0.333636	0.980531	Biso	1.000000	Si
Si360	1.0	0.226728	0.337112	0.402470	Biso	1.000000	Si
Si361	1.0	0.994992	0.086776	0.012615	Biso	1.000000	Si
Si362	1.0	0.552428	0.090727	0.959671	Biso	1.000000	Si
Si363	1.0	0.161770	0.072411	0.433013	Biso	1.000000	Si
Si364	1.0	0.505215	0.656037	0.593513	Biso	1.000000	Si
Si365	1.0	0.175316	0.592944	0.474525	Biso	1.000000	Si
Si366	1.0	0.595517	0.288134	0.297892	Biso	1.000000	Si
Si367	1.0	0.193228	0.575518	0.185733	Biso	1.000000	Si
Si368	1.0	0.887106	0.838907	0.632471	Biso	1.000000	Si
Al53	1.0	0.204994	0.987029	0.678161	Biso	1.000000	Al

Si369	1.0	0.763799	0.100104	0.550583	Biso	1.000000	Si
Si370	1.0	0.676334	0.008737	0.559720	Biso	1.000000	Si
Si371	1.0	0.383193	0.974455	0.827657	Biso	1.000000	Si
Si372	1.0	0.739467	0.864730	0.017075	Biso	1.000000	Si
Si373	1.0	0.497123	0.454746	0.146127	Biso	1.000000	Si
Si374	1.0	0.335384	0.669035	0.755966	Biso	1.000000	Si
Si375	1.0	0.314107	0.548038	0.310363	Biso	1.000000	Si
Si376	1.0	0.728785	0.312573	0.321756	Biso	1.000000	Si
Si377	1.0	0.741755	0.983453	0.938961	Biso	1.000000	Si
Si378	1.0	0.797317	0.657596	0.429951	Biso	1.000000	Si
Si379	1.0	0.816727	0.694655	0.114248	Biso	1.000000	Si
Si380	1.0	0.041566	0.764900	0.698267	Biso	1.000000	Si
Si381	1.0	0.750802	0.066661	0.132734	Biso	1.000000	Si
Si382	1.0	0.792073	0.967896	0.291807	Biso	1.000000	Si
Si383	1.0	0.978503	0.758913	0.660982	Biso	1.000000	Si
Si384	1.0	0.470205	0.161439	0.738278	Biso	1.000000	Si
Si385	1.0	0.774027	0.317446	0.210181	Biso	1.000000	Si
Si386	1.0	0.453740	0.710451	0.461412	Biso	1.000000	Si
Si387	1.0	0.053595	0.229456	0.265940	Biso	1.000000	Si
Al54	1.0	0.705500	0.809024	0.930778	Biso	1.000000	Al
Si388	1.0	0.951778	0.422793	0.676237	Biso	1.000000	Si
Al55	1.0	0.108061	0.764213	0.941286	Biso	1.000000	Al
Si389	1.0	0.251025	0.682754	0.696165	Biso	1.000000	Si
Si390	1.0	0.613141	0.292769	0.880454	Biso	1.000000	Si
Al56	1.0	0.647993	0.527405	0.573559	Biso	1.000000	Al
Si391	1.0	0.005903	0.608123	0.636225	Biso	1.000000	Si
Si392	1.0	0.880474	0.125314	0.368581	Biso	1.000000	Si
Si393	1.0	0.286363	0.356473	0.060167	Biso	1.000000	Si
Si394	1.0	0.350036	0.201214	0.753075	Biso	1.000000	Si
Si395	1.0	0.973417	0.457511	0.740102	Biso	1.000000	Si
Si396	1.0	0.729488	0.934772	0.261113	Biso	1.000000	Si
Al57	1.0	0.309909	0.399835	0.143253	Biso	1.000000	Al
Si397	1.0	0.902289	0.302975	0.182421	Biso	1.000000	Si
Si398	1.0	0.241542	0.654028	0.402513	Biso	1.000000	Si
Si399	1.0	0.117037	0.693147	0.557849	Biso	1.000000	Si
Si400	1.0	0.356690	0.513877	0.814428	Biso	1.000000	Si
Si401	1.0	0.925335	0.979323	0.646461	Biso	1.000000	Si
Si402	1.0	0.633908	0.120289	0.889128	Biso	1.000000	Si
Si403	1.0	0.570824	0.658264	0.695252	Biso	1.000000	Si
Si404	1.0	0.195068	0.541170	0.356001	Biso	1.000000	Si
Si405	1.0	0.718281	0.139949	0.154612	Biso	1.000000	Si
Si406	1.0	0.129175	0.819749	0.367580	Biso	1.000000	Si
Si407	1.0	0.308434	0.962349	0.812876	Biso	1.000000	Si
Si408	1.0	0.776740	0.639628	0.313332	Biso	1.000000	Si
Si409	1.0	0.170492	0.454906	0.746284	Biso	1.000000	Si
Si410	1.0	0.912914	0.072737	0.528997	Biso	1.000000	Si
Si411	1.0	0.577851	0.687560	0.495247	Biso	1.000000	Si
Si412	1.0	0.230786	0.585019	0.626538	Biso	1.000000	Si
Al58	1.0	0.154057	0.383423	0.779581	Biso	1.000000	Al
Si413	1.0	0.595606	0.225540	0.406677	Biso	1.000000	Si
Si414	1.0	0.276668	0.708094	0.537936	Biso	1.000000	Si
Si415	1.0	0.385359	0.686730	0.546968	Biso	1.000000	Si
Si416	1.0	0.310816	0.044953	0.362757	Biso	1.000000	Si
Si417	1.0	0.860223	0.155060	0.017005	Biso	1.000000	Si
Si418	1.0	0.799581	0.623878	0.768040	Biso	1.000000	Si
Si419	1.0	0.635000	0.179071	0.736933	Biso	1.000000	Si
Si420	1.0	0.837580	0.555374	0.472028	Biso	1.000000	Si
Al59	1.0	0.101905	0.769111	0.552709	Biso	1.000000	Al
Si421	1.0	0.292107	0.892294	0.698999	Biso	1.000000	Si
Si422	1.0	0.724267	0.285677	0.161710	Biso	1.000000	Si
Si423	1.0	0.814339	0.951884	0.642983	Biso	1.000000	Si
Si424	1.0	0.167020	0.611796	0.730467	Biso	1.000000	Si
Si425	1.0	0.144401	0.010461	0.057990	Biso	1.000000	Si
Si426	1.0	0.028653	0.311032	0.663764	Biso	1.000000	Si
Si427	1.0	0.147391	0.336272	0.126960	Biso	1.000000	Si
Si428	1.0	0.857801	0.965671	0.550666	Biso	1.000000	Si
Si429	1.0	0.099840	0.263963	0.314655	Biso	1.000000	Si
Si430	1.0	0.571004	0.035646	0.525205	Biso	1.000000	Si
Al60	1.0	0.670910	0.934248	0.308480	Biso	1.000000	Al
Si431	1.0	0.992583	0.626662	0.500318	Biso	1.000000	Si
Si432	1.0	0.091537	0.818182	0.666497	Biso	1.000000	Si
Si433	1.0	0.255322	0.784774	0.452797	Biso	1.000000	Si
Si434	1.0	0.567332	0.546560	0.192869	Biso	1.000000	Si
Si435	1.0	0.763198	0.422087	0.291609	Biso	1.000000	Si
Si436	1.0	0.820589	0.071714	0.163769	Biso	1.000000	Si
Si437	1.0	0.191049	0.557777	0.567559	Biso	1.000000	Si
Si438	1.0	0.104800	0.871423	0.223980	Biso	1.000000	Si
Si439	1.0	0.923444	0.708767	0.604695	Biso	1.000000	Si
Si440	1.0	0.881174	0.977415	0.375013	Biso	1.000000	Si
Al61	1.0	0.267224	0.297721	0.176868	Biso	1.000000	Al

Si441	1.0	0.143821	0.518404	0.473406	Biso	1.000000	Si
Si442	1.0	0.691218	0.466287	0.070078	Biso	1.000000	Si
Si443	1.0	0.630541	0.491981	0.032080	Biso	1.000000	Si
Si444	1.0	0.002898	0.606977	0.316284	Biso	1.000000	Si
Si445	1.0	0.918237	0.246381	0.856308	Biso	1.000000	Si
Si446	1.0	0.100645	0.676051	0.165593	Biso	1.000000	Si
Si447	1.0	0.162506	0.861500	0.478338	Biso	1.000000	Si
Al62	1.0	0.047068	0.146771	0.255178	Biso	1.000000	Al
Al63	1.0	0.503634	0.008917	0.626427	Biso	1.000000	Al
Si448	1.0	0.001808	0.665559	0.559916	Biso	1.000000	Si
Al64	1.0	0.368918	0.744007	0.497156	Biso	1.000000	Al
Si449	1.0	0.258506	0.103121	0.755760	Biso	1.000000	Si
Si450	1.0	0.199407	0.712575	0.307758	Biso	1.000000	Si
Si451	1.0	0.976631	0.033475	0.552955	Biso	1.000000	Si
Si452	1.0	0.710492	0.166255	0.994367	Biso	1.000000	Si
Si453	1.0	0.383309	0.733158	0.771603	Biso	1.000000	Si
Si454	1.0	0.965630	0.034762	0.332818	Biso	1.000000	Si
Si455	1.0	0.593864	0.980700	0.473189	Biso	1.000000	Si
Si456	1.0	0.079575	0.296875	0.551256	Biso	1.000000	Si
Si457	1.0	0.051861	0.981366	0.469591	Biso	1.000000	Si
Si458	1.0	0.569956	0.005494	0.594360	Biso	1.000000	Si
Si459	1.0	0.709545	0.561696	0.884647	Biso	1.000000	Si
Si460	1.0	0.764453	0.738725	0.151718	Biso	1.000000	Si
Si461	1.0	0.298815	0.472674	0.109115	Biso	1.000000	Si
Si462	1.0	0.095608	0.951511	0.415151	Biso	1.000000	Si
Si463	1.0	0.304628	0.894397	0.769703	Biso	1.000000	Si
Si464	1.0	0.454307	0.887407	0.588902	Biso	1.000000	Si
Al65	1.0	0.742702	0.196652	0.509365	Biso	1.000000	Al
Si465	1.0	0.107927	0.108347	0.782361	Biso	1.000000	Si
Si466	1.0	0.927374	0.736128	0.756551	Biso	1.000000	Si
Al66	1.0	0.036373	0.355786	0.387394	Biso	1.000000	Al
Si467	1.0	0.125557	0.329836	0.878243	Biso	1.000000	Si
Si468	1.0	0.844105	0.076202	0.617819	Biso	1.000000	Si
Si469	1.0	0.683035	0.612658	0.051620	Biso	1.000000	Si
Si470	1.0	0.005190	0.216811	0.728898	Biso	1.000000	Si
Si471	1.0	0.248793	0.558858	0.774239	Biso	1.000000	Si
Si472	1.0	0.422191	0.531837	0.626284	Biso	1.000000	Si
Si473	1.0	0.077061	0.342207	0.613577	Biso	1.000000	Si
Si474	1.0	0.528107	0.727119	0.450829	Biso	1.000000	Si
Si475	1.0	0.199778	0.849000	0.026435	Biso	1.000000	Si
Si476	1.0	0.125230	0.872306	0.024759	Biso	1.000000	Si
Si477	1.0	0.013996	0.947219	0.263576	Biso	1.000000	Si
Al67	1.0	0.361956	0.277492	0.727999	Biso	1.000000	Al
Si478	1.0	0.020380	0.403257	0.495903	Biso	1.000000	Si
Si479	1.0	0.698999	0.137666	0.062537	Biso	1.000000	Si
Si480	1.0	0.657293	0.113420	0.627534	Biso	1.000000	Si
Si481	1.0	0.178094	0.849305	0.606137	Biso	1.000000	Si
Si482	1.0	0.716784	0.888253	0.672469	Biso	1.000000	Si
Si483	1.0	0.237390	0.711045	0.054518	Biso	1.000000	Si
Si484	1.0	0.439939	0.158697	0.243821	Biso	1.000000	Si
Si485	1.0	0.576489	0.949141	0.007160	Biso	1.000000	Si
Si486	1.0	0.015405	0.780052	0.330982	Biso	1.000000	Si
Si487	1.0	0.979498	0.253186	0.270433	Biso	1.000000	Si
Si488	1.0	0.265490	0.514798	0.997674	Biso	1.000000	Si
Si489	1.0	0.091812	0.693561	0.280917	Biso	1.000000	Si
Si490	1.0	0.731093	0.839067	0.730394	Biso	1.000000	Si
Si491	1.0	0.653305	0.894805	0.178902	Biso	1.000000	Si
Si492	1.0	0.030140	0.646598	0.149765	Biso	1.000000	Si
Si493	1.0	0.893841	0.126082	0.584090	Biso	1.000000	Si
Si494	1.0	0.763479	0.638203	0.493475	Biso	1.000000	Si
Si495	1.0	0.121515	0.541168	0.336468	Biso	1.000000	Si
Si496	1.0	0.495530	0.098642	0.006162	Biso	1.000000	Si
Si497	1.0	0.472233	0.245871	0.736312	Biso	1.000000	Si
Si498	1.0	0.154319	0.017980	0.327079	Biso	1.000000	Si
Si499	1.0	0.053735	0.422001	0.346468	Biso	1.000000	Si
Si500	1.0	0.257147	0.541149	0.889593	Biso	1.000000	Si
Si501	1.0	0.722661	0.539792	0.580143	Biso	1.000000	Si
Si502	1.0	0.672262	0.484391	0.732527	Biso	1.000000	Si
Si503	1.0	0.916909	0.646277	0.499648	Biso	1.000000	Si
Si504	1.0	0.286027	0.481367	0.224794	Biso	1.000000	Si
Si505	1.0	0.197899	0.137341	0.843893	Biso	1.000000	Si
Si506	1.0	0.693869	0.672554	0.554413	Biso	1.000000	Si
Si507	1.0	0.210888	0.768243	0.006618	Biso	1.000000	Si
Si508	1.0	0.870362	0.588449	0.531383	Biso	1.000000	Si
O1	1.0	0.863355	0.294410	0.094642	Biso	1.000000	O
O2	1.0	0.709765	0.503165	0.812781	Biso	1.000000	O
O3	1.0	0.646725	0.577575	0.975969	Biso	1.000000	O
O4	1.0	0.579057	0.908652	0.237648	Biso	1.000000	O
O5	1.0	0.453101	0.997541	0.743702	Biso	1.000000	O
O6	1.0	0.231626	0.860160	0.522584	Biso	1.000000	O
O7	1.0	0.662522	0.579586	0.065100	Biso	1.000000	O
O8	1.0	0.996078	0.733622	0.194321	Biso	1.000000	O
O9	1.0	0.122172	0.187796	0.885430	Biso	1.000000	O
O10	1.0	0.024014	0.506422	0.968532	Biso	1.000000	O
O11	1.0	0.552164	0.482402	0.924836	Biso	1.000000	O

O12	1.0	0.684470	0.235155	0.050726	Biso	1.000000	O
O13	1.0	0.571744	0.476061	0.552190	Biso	1.000000	O
O14	1.0	0.832906	0.387612	0.232697	Biso	1.000000	O
O15	1.0	0.556073	0.411822	0.032846	Biso	1.000000	O
O16	1.0	0.499229	0.590581	0.520948	Biso	1.000000	O
O17	1.0	0.949889	0.418071	0.451461	Biso	1.000000	O
O18	1.0	0.679367	0.953270	0.343047	Biso	1.000000	O
O19	1.0	0.541850	0.031114	0.032698	Biso	1.000000	O
O20	1.0	0.629569	0.575472	0.769772	Biso	1.000000	O
O21	1.0	0.430349	0.728260	0.435694	Biso	1.000000	O
O22	1.0	0.103155	0.849329	0.001880	Biso	1.000000	O
O23	1.0	0.358927	0.484656	0.843237	Biso	1.000000	O
O24	1.0	0.185262	0.110254	0.817612	Biso	1.000000	O
O25	1.0	0.556122	0.186693	0.863516	Biso	1.000000	O
O26	1.0	0.829192	0.599228	0.766079	Biso	1.000000	O
O27	1.0	0.498654	0.483388	0.763070	Biso	1.000000	O
O28	1.0	0.861103	0.115977	0.337254	Biso	1.000000	O
O29	1.0	0.635728	0.512577	0.999485	Biso	1.000000	O
O30	1.0	0.022108	0.323568	0.158311	Biso	1.000000	O
O31	1.0	0.427558	0.489308	0.621836	Biso	1.000000	O
O32	1.0	0.952702	0.727354	0.587061	Biso	1.000000	O
O33	1.0	0.834159	0.132681	0.997021	Biso	1.000000	O
O34	1.0	0.709370	0.804110	0.727230	Biso	1.000000	O
O35	1.0	0.419500	0.722733	0.770428	Biso	1.000000	O
O36	1.0	0.536022	0.079833	0.927223	Biso	1.000000	O
O37	1.0	0.986253	0.949111	0.138985	Biso	1.000000	O
O38	1.0	0.680383	0.232033	0.805459	Biso	1.000000	O
O39	1.0	0.618792	0.257426	0.410210	Biso	1.000000	O
O40	1.0	0.885190	0.529470	0.604964	Biso	1.000000	O
O41	1.0	0.281970	0.863490	0.784430	Biso	1.000000	O
O42	1.0	0.451910	0.289471	0.968077	Biso	1.000000	O
O43	1.0	0.957551	0.178726	0.337832	Biso	1.000000	O
O44	1.0	0.364200	0.443383	0.742603	Biso	1.000000	O
O45	1.0	0.991551	0.269083	0.183011	Biso	1.000000	O
O46	1.0	0.132167	0.333750	0.972704	Biso	1.000000	O
O47	1.0	0.604748	0.193686	0.718072	Biso	1.000000	O
O48	1.0	0.666143	0.778039	0.687651	Biso	1.000000	O
O49	1.0	0.932303	0.001316	0.449708	Biso	1.000000	O
O50	1.0	0.581553	0.695028	0.679095	Biso	1.000000	O
O51	1.0	0.301997	0.676219	0.773104	Biso	1.000000	O
O52	1.0	0.518543	0.697409	0.360296	Biso	1.000000	O
O53	1.0	0.601606	0.941973	0.487868	Biso	1.000000	O
O54	1.0	0.845400	0.821047	0.486169	Biso	1.000000	O
O55	1.0	0.952241	0.535494	0.280183	Biso	1.000000	O
O56	1.0	0.778210	0.358708	0.216390	Biso	1.000000	O
O57	1.0	0.065071	0.146337	0.991694	Biso	1.000000	O
O58	1.0	0.318620	0.853227	0.491948	Biso	1.000000	O
O59	1.0	0.732566	0.296045	0.433872	Biso	1.000000	O
O60	1.0	0.213555	0.612618	0.185365	Biso	1.000000	O
O61	1.0	0.460333	0.927112	0.675820	Biso	1.000000	O
O62	1.0	0.961393	0.993018	0.341886	Biso	1.000000	O
O63	1.0	0.109050	0.655199	0.271559	Biso	1.000000	O
O64	1.0	0.909377	0.808965	0.541803	Biso	1.000000	O
O65	1.0	0.672454	0.652872	0.528204	Biso	1.000000	O
O66	1.0	0.328572	0.772633	0.324370	Biso	1.000000	O
O67	1.0	0.497184	0.355708	0.107419	Biso	1.000000	O
O68	1.0	0.091800	0.765249	0.906213	Biso	1.000000	O
O69	1.0	0.580899	0.941651	0.044812	Biso	1.000000	O
O70	1.0	0.989952	0.942254	0.603100	Biso	1.000000	O
O71	1.0	0.783313	0.691055	0.234383	Biso	1.000000	O
O72	1.0	0.439295	0.013147	0.984134	Biso	1.000000	O
O73	1.0	0.404152	0.263363	0.441081	Biso	1.000000	O
O74	1.0	0.251363	0.448865	0.636557	Biso	1.000000	O
O75	1.0	0.690239	0.283814	0.176842	Biso	1.000000	O
O76	1.0	0.270233	0.503311	0.603728	Biso	1.000000	O
O77	1.0	0.361852	0.299237	0.762438	Biso	1.000000	O
O78	1.0	0.718601	0.889653	0.816889	Biso	1.000000	O
O79	1.0	0.959361	0.994766	0.658814	Biso	1.000000	O
O80	1.0	0.053331	0.752418	0.213773	Biso	1.000000	O
O81	1.0	0.650901	0.882612	0.890287	Biso	1.000000	O
O82	1.0	0.937455	0.681608	0.630413	Biso	1.000000	O
O83	1.0	0.548346	0.234962	0.907390	Biso	1.000000	O
O84	1.0	0.254873	0.255835	0.183807	Biso	1.000000	O
O85	1.0	0.159065	0.069616	0.470334	Biso	1.000000	O
O86	1.0	0.679249	0.475969	0.104537	Biso	1.000000	O
O87	1.0	0.887663	0.014276	0.357044	Biso	1.000000	O
O88	1.0	0.528741	0.466244	0.127849	Biso	1.000000	O

O89	1.0	0.027905	0.923364	0.292496	Biso	1.000000	O
O90	1.0	0.823662	0.429907	0.365702	Biso	1.000000	O
O91	1.0	0.631377	0.404038	0.794027	Biso	1.000000	O
O92	1.0	0.824118	0.047732	0.494192	Biso	1.000000	O
O93	1.0	0.837678	0.404240	0.808119	Biso	1.000000	O
O94	1.0	0.666887	0.052089	0.020958	Biso	1.000000	O
O95	1.0	0.879593	0.737901	0.677564	Biso	1.000000	O
O96	1.0	0.140728	0.342321	0.222671	Biso	1.000000	O
O97	1.0	0.238277	0.105606	0.316704	Biso	1.000000	O
O98	1.0	0.603473	0.665978	0.581252	Biso	1.000000	O
O99	1.0	0.136854	0.741453	0.046083	Biso	1.000000	O
O100	1.0	0.028162	0.614742	0.343535	Biso	1.000000	O
O101	1.0	0.144986	0.616791	0.387272	Biso	1.000000	O
O102	1.0	0.030714	0.068219	0.623426	Biso	1.000000	O
O103	1.0	0.531989	0.770025	0.453238	Biso	1.000000	O
O104	1.0	0.860906	0.678114	0.362995	Biso	1.000000	O
O105	1.0	0.070017	0.631919	0.067889	Biso	1.000000	O
O106	1.0	0.811338	0.889529	0.853509	Biso	1.000000	O
O107	1.0	0.903127	0.098897	0.556796	Biso	1.000000	O
O108	1.0	0.330855	0.543081	0.824674	Biso	1.000000	O
O109	1.0	0.857638	0.048570	0.642539	Biso	1.000000	O
O110	1.0	0.161858	0.667032	0.230183	Biso	1.000000	O
O111	1.0	0.880370	0.945381	0.350315	Biso	1.000000	O
O112	1.0	0.841605	0.515261	0.460721	Biso	1.000000	O
O113	1.0	0.666920	0.098141	0.884671	Biso	1.000000	O
O114	1.0	0.284321	0.465368	0.074614	Biso	1.000000	O
O115	1.0	0.143831	0.258812	0.722345	Biso	1.000000	O
O116	1.0	0.575838	0.729246	0.580317	Biso	1.000000	O
O117	1.0	0.074732	0.462668	0.875857	Biso	1.000000	O
O118	1.0	0.047253	0.659067	0.694227	Biso	1.000000	O
O119	1.0	0.003274	0.033900	0.525458	Biso	1.000000	O
O120	1.0	0.930795	0.111669	0.067939	Biso	1.000000	O
O121	1.0	0.111090	0.867279	0.059393	Biso	1.000000	O
O122	1.0	0.842221	0.969745	0.173062	Biso	1.000000	O
O123	1.0	0.620627	0.252839	0.794010	Biso	1.000000	O
O124	1.0	0.120634	0.213129	0.397234	Biso	1.000000	O
O125	1.0	0.527523	0.035144	0.812734	Biso	1.000000	O
O126	1.0	0.619364	0.707629	0.806465	Biso	1.000000	O
O127	1.0	0.887680	0.330217	0.724485	Biso	1.000000	O
O128	1.0	0.540713	0.940067	0.998143	Biso	1.000000	O
O129	1.0	0.170565	0.165934	0.851152	Biso	1.000000	O
O130	1.0	0.161593	0.821976	0.491793	Biso	1.000000	O
O131	1.0	0.084009	0.378861	0.700068	Biso	1.000000	O
O132	1.0	0.783821	0.965726	0.623114	Biso	1.000000	O
O133	1.0	0.622614	0.150419	0.763339	Biso	1.000000	O
O134	1.0	0.771573	0.911995	0.546050	Biso	1.000000	O
O135	1.0	0.702410	0.690612	0.638934	Biso	1.000000	O
O136	1.0	0.867138	0.277338	0.503447	Biso	1.000000	O
O137	1.0	0.942937	0.048608	0.539314	Biso	1.000000	O
O138	1.0	0.893232	0.504863	0.123577	Biso	1.000000	O
O139	1.0	0.142712	0.633238	0.707224	Biso	1.000000	O
O140	1.0	0.868941	0.902819	0.989344	Biso	1.000000	O
O141	1.0	0.467825	0.163229	0.947164	Biso	1.000000	O
O142	1.0	0.106566	0.689558	0.072757	Biso	1.000000	O
O143	1.0	0.034539	0.219512	0.704471	Biso	1.000000	O
O144	1.0	0.515425	0.104522	0.812833	Biso	1.000000	O
O145	1.0	0.013500	0.661261	0.055774	Biso	1.000000	O
O146	1.0	0.776286	0.294791	0.634774	Biso	1.000000	O
O147	1.0	0.129148	0.469425	0.395051	Biso	1.000000	O
O148	1.0	0.139868	0.057582	0.323634	Biso	1.000000	O
O149	1.0	0.882400	0.675946	0.283173	Biso	1.000000	O
O150	1.0	0.159753	0.555193	0.482222	Biso	1.000000	O
O151	1.0	0.938133	0.057132	0.350831	Biso	1.000000	O
O152	1.0	0.737604	0.443432	0.794352	Biso	1.000000	O
O153	1.0	0.926897	0.138232	0.601823	Biso	1.000000	O
O154	1.0	0.264992	0.726384	0.795398	Biso	1.000000	O
O155	1.0	0.748402	0.793812	0.848488	Biso	1.000000	O
O156	1.0	0.971613	0.970234	0.410104	Biso	1.000000	O
O157	1.0	0.672683	0.805494	0.629934	Biso	1.000000	O
O158	1.0	0.627550	0.698223	0.927759	Biso	1.000000	O
O159	1.0	0.797938	0.641863	0.803281	Biso	1.000000	O
O160	1.0	0.919685	0.600131	0.135003	Biso	1.000000	O
O161	1.0	0.914361	0.523177	0.232840	Biso	1.000000	O
O162	1.0	0.275851	0.710478	0.052932	Biso	1.000000	O
O163	1.0	0.118737	0.728117	0.817541	Biso	1.000000	O

O164	1.0	0.969044	0.650255	0.576068	Biso	1.000000	0
O165	1.0	0.155043	0.168968	0.094839	Biso	1.000000	0
O166	1.0	0.748952	0.470577	0.409110	Biso	1.000000	0
O167	1.0	0.270258	0.395140	0.064219	Biso	1.000000	0
O168	1.0	0.445924	0.363911	0.142965	Biso	1.000000	0
O169	1.0	0.073221	0.241480	0.296802	Biso	1.000000	0
O170	1.0	0.530892	0.454557	0.713334	Biso	1.000000	0
O171	1.0	0.893634	0.997751	0.147273	Biso	1.000000	0
O172	1.0	0.006379	0.648296	0.470780	Biso	1.000000	0
O173	1.0	0.107966	0.445824	0.524696	Biso	1.000000	0
O174	1.0	0.831500	0.264628	0.817293	Biso	1.000000	0
O175	1.0	0.963861	0.845586	0.236657	Biso	1.000000	0
O176	1.0	0.217947	0.155781	0.426956	Biso	1.000000	0
O177	1.0	0.815358	0.212019	0.083411	Biso	1.000000	0
O178	1.0	0.588406	0.431672	0.948731	Biso	1.000000	0
O179	1.0	0.100360	0.725327	0.957924	Biso	1.000000	0
O180	1.0	0.406044	0.137837	0.026189	Biso	1.000000	0
O181	1.0	0.291695	0.304118	0.782365	Biso	1.000000	0
O182	1.0	0.546102	0.155769	0.601014	Biso	1.000000	0
O183	1.0	0.858447	0.778958	0.353087	Biso	1.000000	0
O184	1.0	0.378251	0.131168	0.083238	Biso	1.000000	0
O185	1.0	0.591741	0.166437	0.816002	Biso	1.000000	0
O186	1.0	0.062371	0.795264	0.680289	Biso	1.000000	0
O187	1.0	0.535921	0.326509	0.756612	Biso	1.000000	0
O188	1.0	0.120613	0.539757	0.858181	Biso	1.000000	0
O189	1.0	0.207016	0.227709	0.363758	Biso	1.000000	0
O190	1.0	0.673507	0.469171	0.920676	Biso	1.000000	0
O191	1.0	0.062306	0.446192	0.716288	Biso	1.000000	0
O192	1.0	0.779399	0.406608	0.260775	Biso	1.000000	0
O193	1.0	0.993315	0.328422	0.664946	Biso	1.000000	0
O194	1.0	0.067872	0.790996	0.546647	Biso	1.000000	0
O195	1.0	0.242542	0.325201	0.195818	Biso	1.000000	0
O196	1.0	0.798947	0.860314	0.515403	Biso	1.000000	0
O197	1.0	0.810329	0.082457	0.198428	Biso	1.000000	0
O198	1.0	0.815822	0.299177	0.138639	Biso	1.000000	0
O199	1.0	0.240516	0.746264	0.458460	Biso	1.000000	0
O200	1.0	0.016139	0.593773	0.070173	Biso	1.000000	0
O201	1.0	0.052977	0.808989	0.878040	Biso	1.000000	0
O202	1.0	0.098567	0.277622	0.407340	Biso	1.000000	0
O203	1.0	0.713869	0.165341	0.685957	Biso	1.000000	0
O204	1.0	0.844268	0.417114	0.590001	Biso	1.000000	0
O205	1.0	0.399271	0.289666	0.934263	Biso	1.000000	0
O206	1.0	0.727271	0.206515	0.299370	Biso	1.000000	0
O207	1.0	0.480843	0.658437	0.167835	Biso	1.000000	0
O208	1.0	0.805044	0.593114	0.154453	Biso	1.000000	0
O209	1.0	0.060309	0.153911	0.141824	Biso	1.000000	0
O210	1.0	0.487161	0.146451	0.769786	Biso	1.000000	0
O211	1.0	0.806538	0.281414	0.686172	Biso	1.000000	0
O212	1.0	0.395789	0.791029	0.696519	Biso	1.000000	0
O213	1.0	0.052753	0.136844	0.217478	Biso	1.000000	0
O214	1.0	0.727335	0.581862	0.583895	Biso	1.000000	0
O215	1.0	0.782391	0.410049	0.322162	Biso	1.000000	0
O216	1.0	0.404534	0.891883	0.771414	Biso	1.000000	0
O217	1.0	0.223398	0.676335	0.670511	Biso	1.000000	0
O218	1.0	0.331632	0.761759	0.500200	Biso	1.000000	0
O219	1.0	0.735804	0.990073	0.656452	Biso	1.000000	0
O220	1.0	0.702523	0.735314	0.720578	Biso	1.000000	0
O221	1.0	0.223588	0.674527	0.040801	Biso	1.000000	0
O222	1.0	0.559447	0.253745	0.136178	Biso	1.000000	0
O223	1.0	0.755078	0.611437	0.293739	Biso	1.000000	0
O224	1.0	0.889831	0.550314	0.283713	Biso	1.000000	0
O225	1.0	0.539008	0.632241	0.157611	Biso	1.000000	0
O226	1.0	0.466168	0.476475	0.132424	Biso	1.000000	0
O227	1.0	0.378014	0.448808	0.602619	Biso	1.000000	0
O228	1.0	0.648679	0.517082	0.740201	Biso	1.000000	0
O229	1.0	0.290553	0.524759	0.970211	Biso	1.000000	0
O230	1.0	0.265648	0.623597	0.416031	Biso	1.000000	0
O231	1.0	0.785767	0.396002	0.040822	Biso	1.000000	0
O232	1.0	0.069375	0.061941	0.673550	Biso	1.000000	0
O233	1.0	0.659575	0.348245	0.768408	Biso	1.000000	0
O234	1.0	0.172482	0.319965	0.717022	Biso	1.000000	0
O235	1.0	0.324581	0.406833	0.179673	Biso	1.000000	0
O236	1.0	0.010128	0.133899	0.264140	Biso	1.000000	0
O237	1.0	0.127830	0.183619	0.042126	Biso	1.000000	0
O238	1.0	0.835185	0.485851	0.105928	Biso	1.000000	0
O239	1.0	0.469833	0.181642	0.257330	Biso	1.000000	0
O240	1.0	0.547643	0.179048	0.697005	Biso	1.000000	0
O241	1.0	0.941145	0.563277	0.722109	Biso	1.000000	0
O242	1.0	0.847669	0.443897	0.464942	Biso	1.000000	0
O243	1.0	0.257497	0.101208	0.421477	Biso	1.000000	0

O244	1.0	0.159309	0.533816	0.342655	Biso	1.000000	O
O245	1.0	0.357704	0.685917	0.574189	Biso	1.000000	O
O246	1.0	0.100600	0.389534	0.950842	Biso	1.000000	O
O247	1.0	0.745457	0.758767	0.521083	Biso	1.000000	O
O248	1.0	0.567337	0.314913	0.497357	Biso	1.000000	O
O249	1.0	0.210279	0.571667	0.597040	Biso	1.000000	O
O250	1.0	0.386091	0.281703	0.648032	Biso	1.000000	O
O251	1.0	0.726486	0.404246	0.293862	Biso	1.000000	O
O252	1.0	0.335973	0.706969	0.074304	Biso	1.000000	O
O253	1.0	0.205779	0.115600	0.875178	Biso	1.000000	O
O254	1.0	0.498358	0.204127	0.663800	Biso	1.000000	O
O255	1.0	0.261354	0.906208	0.680414	Biso	1.000000	O
O256	1.0	0.545418	0.275462	0.543155	Biso	1.000000	O
O257	1.0	0.883591	0.065818	0.067060	Biso	1.000000	O
O258	1.0	0.397074	0.012796	0.817437	Biso	1.000000	O
O259	1.0	0.236121	0.542841	0.997944	Biso	1.000000	O
O260	1.0	0.891780	0.393870	0.471941	Biso	1.000000	O
O261	1.0	0.581974	0.380485	0.857749	Biso	1.000000	O
O262	1.0	0.115137	0.767778	0.213933	Biso	1.000000	O
O263	1.0	0.222438	0.526694	0.878078	Biso	1.000000	O
O264	1.0	0.668910	0.279590	0.763193	Biso	1.000000	O
O265	1.0	0.737173	0.343169	0.840956	Biso	1.000000	O
O266	1.0	0.049382	0.416355	0.517133	Biso	1.000000	O
O267	1.0	0.170928	0.641179	0.652787	Biso	1.000000	O
O268	1.0	0.052209	0.188702	0.261957	Biso	1.000000	O
O269	1.0	0.371954	0.167014	0.743964	Biso	1.000000	O
O270	1.0	0.217379	0.555323	0.328183	Biso	1.000000	O
O271	1.0	0.242078	0.769719	0.755210	Biso	1.000000	O
O272	1.0	0.953987	0.149224	0.161632	Biso	1.000000	O
O273	1.0	0.768673	0.906722	0.783236	Biso	1.000000	O
O274	1.0	0.115757	0.793834	0.646562	Biso	1.000000	O
O275	1.0	0.889197	0.168676	0.995000	Biso	1.000000	O
O276	1.0	0.816697	0.111822	0.252698	Biso	1.000000	O
O277	1.0	0.038998	0.798072	0.422069	Biso	1.000000	O
O278	1.0	0.824266	0.186926	0.548785	Biso	1.000000	O
O279	1.0	0.944307	0.815511	0.040048	Biso	1.000000	O
O280	1.0	0.211336	0.309246	0.499306	Biso	1.000000	O
O281	1.0	0.076199	0.964049	0.445005	Biso	1.000000	O
O282	1.0	0.928178	0.407249	0.187924	Biso	1.000000	O
O283	1.0	0.600287	0.909330	0.098230	Biso	1.000000	O
O284	1.0	0.735414	0.558550	0.451997	Biso	1.000000	O
O285	1.0	0.792167	0.633352	0.399414	Biso	1.000000	O
O286	1.0	0.072015	0.081853	0.063349	Biso	1.000000	O
O287	1.0	0.448835	0.030833	0.554149	Biso	1.000000	O
O288	1.0	0.647544	0.147194	0.606960	Biso	1.000000	O
O289	1.0	0.881465	0.309802	0.214466	Biso	1.000000	O
O290	1.0	0.964937	0.254458	0.236209	Biso	1.000000	O
O291	1.0	0.041660	0.540635	0.917612	Biso	1.000000	O
O292	1.0	0.501246	0.464860	0.182525	Biso	1.000000	O
O293	1.0	0.947864	0.212456	0.594763	Biso	1.000000	O
O294	1.0	0.900340	0.689939	0.580140	Biso	1.000000	O
O295	1.0	0.167628	0.511490	0.115101	Biso	1.000000	O
O296	1.0	0.807596	0.817965	0.850142	Biso	1.000000	O
O297	1.0	0.572235	0.348891	0.010632	Biso	1.000000	O
O298	1.0	0.989284	0.428974	0.499893	Biso	1.000000	O
O299	1.0	0.623206	0.883302	0.199702	Biso	1.000000	O
O300	1.0	0.171281	0.701102	0.329335	Biso	1.000000	O
O301	1.0	0.162943	0.857660	0.292083	Biso	1.000000	O
O302	1.0	0.127789	0.781393	0.382458	Biso	1.000000	O
O303	1.0	0.538239	0.575662	0.195932	Biso	1.000000	O
O304	1.0	0.394430	0.288601	0.707711	Biso	1.000000	O
O305	1.0	0.972488	0.097541	0.628761	Biso	1.000000	O
O306	1.0	0.306409	0.241998	0.036201	Biso	1.000000	O
O307	1.0	0.667785	0.368684	0.058481	Biso	1.000000	O
O308	1.0	0.918510	0.289915	0.859521	Biso	1.000000	O
O309	1.0	0.199701	0.682692	0.943158	Biso	1.000000	O
O310	1.0	0.488710	0.152507	0.707975	Biso	1.000000	O
O311	1.0	0.618698	0.315157	0.316805	Biso	1.000000	O
O312	1.0	0.739876	0.852051	0.536874	Biso	1.000000	O
O313	1.0	0.674883	0.163627	0.078477	Biso	1.000000	O
O314	1.0	0.742821	0.104765	0.518657	Biso	1.000000	O

O315	1.0	0.308355	0.800762	0.656242	Biso	1.000000	O
O316	1.0	0.564199	0.246252	0.491155	Biso	1.000000	O
O317	1.0	0.910496	0.946116	0.898736	Biso	1.000000	O
O318	1.0	0.532880	0.429739	0.580077	Biso	1.000000	O
O319	1.0	0.010188	0.449938	0.750154	Biso	1.000000	O
O320	1.0	0.676003	0.617975	0.015712	Biso	1.000000	O
O321	1.0	0.740909	0.369744	0.698095	Biso	1.000000	O
O322	1.0	0.829359	0.938732	0.560385	Biso	1.000000	O
O323	1.0	0.528122	0.354337	0.053802	Biso	1.000000	O
O324	1.0	0.848116	0.238026	0.129365	Biso	1.000000	O
O325	1.0	0.156421	0.585551	0.197399	Biso	1.000000	O
O326	1.0	0.327735	0.172318	0.090031	Biso	1.000000	O
O327	1.0	0.180568	0.286031	0.052806	Biso	1.000000	O
O328	1.0	0.614394	0.178709	0.970114	Biso	1.000000	O
O329	1.0	0.698200	0.103262	0.711370	Biso	1.000000	O
O330	1.0	0.110180	0.886367	0.314464	Biso	1.000000	O
O331	1.0	0.149903	0.754529	0.483173	Biso	1.000000	O
O332	1.0	0.708822	0.927818	0.678807	Biso	1.000000	O
O333	1.0	0.294719	0.782493	0.453958	Biso	1.000000	O
O334	1.0	0.651002	0.841215	0.806420	Biso	1.000000	O
O335	1.0	0.053512	0.726665	0.685697	Biso	1.000000	O
O336	1.0	0.928553	0.032063	0.245678	Biso	1.000000	O
O337	1.0	0.916547	0.965044	0.248451	Biso	1.000000	O
O338	1.0	0.825497	0.333496	0.814910	Biso	1.000000	O
O339	1.0	0.973856	0.291417	0.287114	Biso	1.000000	O
O340	1.0	0.004792	0.048593	0.996209	Biso	1.000000	O
O341	1.0	0.238591	0.609018	0.026395	Biso	1.000000	O
O342	1.0	0.007633	0.117506	0.989888	Biso	1.000000	O
O343	1.0	0.204778	0.842842	0.633716	Biso	1.000000	O
O344	1.0	0.125727	0.646955	0.180947	Biso	1.000000	O
O345	1.0	0.857673	0.563407	0.502647	Biso	1.000000	O
O346	1.0	0.273321	0.498625	0.127298	Biso	1.000000	O
O347	1.0	0.840613	0.169548	0.126175	Biso	1.000000	O
O348	1.0	0.701480	0.330155	0.343429	Biso	1.000000	O
O349	1.0	0.128052	0.344275	0.912833	Biso	1.000000	O
O350	1.0	0.603227	0.884383	0.795046	Biso	1.000000	O
O351	1.0	0.758203	0.209066	0.706460	Biso	1.000000	O
O352	1.0	0.762586	0.953570	0.271733	Biso	1.000000	O
O353	1.0	0.571537	0.220649	0.435411	Biso	1.000000	O
O354	1.0	0.223511	0.743493	0.034417	Biso	1.000000	O
O355	1.0	0.630312	0.202217	0.251589	Biso	1.000000	O
O356	1.0	0.124523	0.664705	0.531078	Biso	1.000000	O
O357	1.0	0.506714	0.101597	0.603633	Biso	1.000000	O
O358	1.0	0.834018	0.053193	0.587083	Biso	1.000000	O
O359	1.0	0.940581	0.451281	0.540189	Biso	1.000000	O
O360	1.0	0.168593	0.371901	0.124018	Biso	1.000000	O
O361	1.0	0.634068	0.484991	0.570853	Biso	1.000000	O
O362	1.0	0.923976	0.937397	0.651416	Biso	1.000000	O
O363	1.0	0.690621	0.868763	0.650347	Biso	1.000000	O
O364	1.0	0.156017	0.927196	0.292007	Biso	1.000000	O
O365	1.0	0.126086	0.952241	0.524342	Biso	1.000000	O
O366	1.0	0.425498	0.085780	0.064568	Biso	1.000000	O
O367	1.0	0.599026	0.272324	0.089482	Biso	1.000000	O
O368	1.0	0.661533	0.895851	0.086088	Biso	1.000000	O
O369	1.0	0.798481	0.458090	0.149008	Biso	1.000000	O
O370	1.0	0.613154	0.705304	0.488163	Biso	1.000000	O
O371	1.0	0.607429	0.273979	0.528653	Biso	1.000000	O
O372	1.0	0.782884	0.696742	0.422286	Biso	1.000000	O
O373	1.0	0.317791	0.825763	0.025647	Biso	1.000000	O
O374	1.0	0.802841	0.430029	0.091481	Biso	1.000000	O
O375	1.0	0.846283	0.611904	0.374565	Biso	1.000000	O
O376	1.0	0.048740	0.844648	0.971545	Biso	1.000000	O
O377	1.0	0.069069	0.200371	0.793751	Biso	1.000000	O
O378	1.0	0.017378	0.438772	0.898223	Biso	1.000000	O
O379	1.0	0.441554	0.823439	0.503789	Biso	1.000000	O
O380	1.0	0.258725	0.875528	0.577724	Biso	1.000000	O
O381	1.0	0.640764	0.955542	0.291080	Biso	1.000000	O
O382	1.0	0.686290	0.795380	0.838789	Biso	1.000000	O
O383	1.0	0.639898	0.548309	0.540387	Biso	1.000000	O
O384	1.0	0.709096	0.234121	0.674992	Biso	1.000000	O
O385	1.0	0.884716	0.445521	0.377963	Biso	1.000000	O
O386	1.0	0.888314	0.844245	0.052654	Biso	1.000000	O
O387	1.0	0.040164	0.412665	0.950169	Biso	1.000000	O
O388	1.0	0.998704	0.252782	0.603131	Biso	1.000000	O
O389	1.0	0.013828	0.186055	0.979194	Biso	1.000000	O
O390	1.0	0.999365	0.584746	0.494799	Biso	1.000000	O
O391	1.0	0.054970	0.733001	0.598657	Biso	1.000000	O
O392	1.0	0.011281	0.171428	0.180908	Biso	1.000000	O
O393	1.0	0.581779	0.603985	0.118950	Biso	1.000000	O
O394	1.0	0.524154	0.107358	0.982087	Biso	1.000000	O

O395	1.0	0.806136	0.234439	0.641522	Biso	1.000000	O
O396	1.0	0.671717	0.979161	0.765201	Biso	1.000000	O
O397	1.0	0.592034	0.135362	0.504063	Biso	1.000000	O
O398	1.0	0.575902	0.263738	0.321583	Biso	1.000000	O
O399	1.0	0.015790	0.732506	0.413325	Biso	1.000000	O
O400	1.0	0.685563	0.833485	0.337759	Biso	1.000000	O
O401	1.0	0.180999	0.984951	0.514037	Biso	1.000000	O
O402	1.0	0.922814	0.339012	0.176122	Biso	1.000000	O
O403	1.0	0.469009	0.224399	0.979905	Biso	1.000000	O
O404	1.0	0.530643	0.767535	0.550942	Biso	1.000000	O
O405	1.0	0.847857	0.013765	0.044389	Biso	1.000000	O
O406	1.0	0.091792	0.166182	0.089583	Biso	1.000000	O
O407	1.0	0.131588	0.096082	0.420281	Biso	1.000000	O
O408	1.0	0.746269	0.897805	0.994095	Biso	1.000000	O
O409	1.0	0.788537	0.733406	0.558130	Biso	1.000000	O
O410	1.0	0.132295	0.981821	0.032765	Biso	1.000000	O
O411	1.0	0.063011	0.167796	0.585792	Biso	1.000000	O
O412	1.0	0.128782	0.734003	0.427782	Biso	1.000000	O
O413	1.0	0.936187	0.327774	0.763347	Biso	1.000000	O
O414	1.0	0.586623	0.528713	0.751147	Biso	1.000000	O
O415	1.0	0.906104	0.194203	0.304698	Biso	1.000000	O
O416	1.0	0.221054	0.119986	0.374449	Biso	1.000000	O
O417	1.0	0.250695	0.652742	0.720797	Biso	1.000000	O
O418	1.0	0.686327	0.857224	0.591030	Biso	1.000000	O
O419	1.0	0.708107	0.410544	0.927335	Biso	1.000000	O
O420	1.0	0.972744	0.323438	0.874609	Biso	1.000000	O
O421	1.0	0.220486	0.103348	0.764996	Biso	1.000000	O
O422	1.0	0.421086	0.967176	0.642525	Biso	1.000000	O
O423	1.0	0.639894	0.458395	0.150424	Biso	1.000000	O
O424	1.0	0.654174	0.260563	0.225929	Biso	1.000000	O
O425	1.0	0.788112	0.224177	0.028796	Biso	1.000000	O
O426	1.0	0.502174	0.981681	0.780341	Biso	1.000000	O
O427	1.0	0.359798	0.874896	0.449500	Biso	1.000000	O
O428	1.0	0.451094	0.546114	0.647940	Biso	1.000000	O
O429	1.0	0.113207	0.012948	0.661268	Biso	1.000000	O
O430	1.0	0.626472	0.087584	0.630852	Biso	1.000000	O
O431	1.0	0.267375	0.262948	0.441235	Biso	1.000000	O
O432	1.0	0.017055	0.987812	0.452684	Biso	1.000000	O
O433	1.0	0.166618	0.416101	0.757131	Biso	1.000000	O
O434	1.0	0.847104	0.410165	0.051480	Biso	1.000000	O
O435	1.0	0.304170	0.553349	0.575341	Biso	1.000000	O
O436	1.0	0.380572	0.842059	0.499140	Biso	1.000000	O
O437	1.0	0.300236	0.059363	0.395828	Biso	1.000000	O
O438	1.0	0.453188	0.937794	0.774333	Biso	1.000000	O
O439	1.0	0.123659	0.089831	0.814051	Biso	1.000000	O
O440	1.0	0.778585	0.776276	0.164316	Biso	1.000000	O
O441	1.0	0.973760	0.435399	0.647577	Biso	1.000000	O
O442	1.0	0.241012	0.343704	0.769972	Biso	1.000000	O
O443	1.0	0.736102	0.144419	0.274213	Biso	1.000000	O
O444	1.0	0.805747	0.298856	0.864224	Biso	1.000000	O
O445	1.0	0.625764	0.119386	0.023706	Biso	1.000000	O
O446	1.0	0.329195	0.290709	0.708864	Biso	1.000000	O
O447	1.0	0.151250	0.888945	0.505453	Biso	1.000000	O
O448	1.0	0.010324	0.089808	0.044940	Biso	1.000000	O
O449	1.0	0.605959	0.523596	0.831866	Biso	1.000000	O
O450	1.0	0.530694	0.832442	0.573099	Biso	1.000000	O
O451	1.0	0.093767	0.313181	0.459687	Biso	1.000000	O
O452	1.0	0.563132	0.666067	0.731904	Biso	1.000000	O
O453	1.0	0.780723	0.675095	0.293395	Biso	1.000000	O
O454	1.0	0.439889	0.266494	0.748653	Biso	1.000000	O
O455	1.0	0.282103	0.342275	0.025099	Biso	1.000000	O
O456	1.0	0.686406	0.092849	0.610700	Biso	1.000000	O
O457	1.0	0.380964	0.891883	0.658285	Biso	1.000000	O
O458	1.0	0.270785	0.328644	0.083267	Biso	1.000000	O
O459	1.0	0.246874	0.453649	0.350447	Biso	1.000000	O
O460	1.0	0.158198	0.531562	0.810242	Biso	1.000000	O
O461	1.0	0.803638	0.790458	0.526800	Biso	1.000000	O
O462	1.0	0.804634	0.966306	0.120317	Biso	1.000000	O
O463	1.0	0.673438	0.517782	0.152075	Biso	1.000000	O
O464	1.0	0.747354	0.224137	0.870550	Biso	1.000000	O
O465	1.0	0.062990	0.562742	0.703180	Biso	1.000000	O
O466	1.0	0.049895	0.324825	0.635170	Biso	1.000000	O
O467	1.0	0.741620	0.528637	0.547596	Biso	1.000000	O
O468	1.0	0.815681	0.670954	0.555961	Biso	1.000000	O
O469	1.0	0.292494	0.066521	0.335475	Biso	1.000000	O
O470	1.0	0.661888	0.909660	0.791651	Biso	1.000000	O
O471	1.0	0.179560	0.482113	0.773436	Biso	1.000000	O
O472	1.0	0.893348	0.590684	0.753726	Biso	1.000000	O
O473	1.0	0.655557	0.179938	0.553302	Biso	1.000000	O
O474	1.0	0.869203	0.227237	0.716392	Biso	1.000000	O

O475	1.0	0.836021	0.983724	0.654451	Biso	1.000000	O
O476	1.0	0.624395	0.084166	0.076627	Biso	1.000000	O
O477	1.0	0.865636	0.847967	0.847570	Biso	1.000000	O
O478	1.0	0.364087	0.156605	0.138431	Biso	1.000000	O
O479	1.0	0.738263	0.107659	0.136550	Biso	1.000000	O
O480	1.0	0.682161	0.984645	0.010787	Biso	1.000000	O
O481	1.0	0.627143	0.057632	0.938061	Biso	1.000000	O
O482	1.0	0.507484	0.076542	0.465659	Biso	1.000000	O
O483	1.0	0.241267	0.129453	0.046468	Biso	1.000000	O
O484	1.0	0.017380	0.245488	0.268085	Biso	1.000000	O
O485	1.0	0.600223	0.183951	0.581917	Biso	1.000000	O
O486	1.0	0.984742	0.395067	0.599610	Biso	1.000000	O
O487	1.0	0.862884	0.627689	0.239953	Biso	1.000000	O
O488	1.0	0.674775	0.238669	0.280681	Biso	1.000000	O
O489	1.0	0.632773	0.192331	0.026872	Biso	1.000000	O
O490	1.0	0.515430	0.030868	0.658087	Biso	1.000000	O
O491	1.0	0.252389	0.475142	0.991392	Biso	1.000000	O
O492	1.0	0.051235	0.847189	0.588885	Biso	1.000000	O
O493	1.0	0.017484	0.327482	0.364246	Biso	1.000000	O
O494	1.0	0.831613	0.824652	0.732947	Biso	1.000000	O
O495	1.0	0.780045	0.618302	0.578180	Biso	1.000000	O
O496	1.0	0.699061	0.553766	0.501370	Biso	1.000000	O
O497	1.0	0.737239	0.892475	0.253944	Biso	1.000000	O
O498	1.0	0.889316	0.731652	0.764710	Biso	1.000000	O
O499	1.0	0.025781	0.774325	0.366197	Biso	1.000000	O
O500	1.0	0.474179	0.852338	0.599549	Biso	1.000000	O
O501	1.0	0.604985	0.366819	0.962053	Biso	1.000000	O
O502	1.0	0.909794	0.542454	0.172339	Biso	1.000000	O
O503	1.0	0.686576	0.097058	0.066701	Biso	1.000000	O
O504	1.0	0.036117	0.134079	0.088344	Biso	1.000000	O
O505	1.0	0.014413	0.866901	0.205728	Biso	1.000000	O
O506	1.0	0.156228	0.814816	0.604546	Biso	1.000000	O
O507	1.0	0.627436	0.273619	0.169521	Biso	1.000000	O
O508	1.0	0.161882	0.858473	0.023655	Biso	1.000000	O
O509	1.0	0.051853	0.388364	0.367078	Biso	1.000000	O
O510	1.0	0.640456	0.523175	0.938962	Biso	1.000000	O
O511	1.0	0.326196	0.108282	0.113338	Biso	1.000000	O
O512	1.0	0.548272	0.180294	0.479676	Biso	1.000000	O
O513	1.0	0.992553	0.035102	0.243753	Biso	1.000000	O
O514	1.0	0.550837	0.708651	0.476299	Biso	1.000000	O
O515	1.0	0.496380	0.635333	0.112609	Biso	1.000000	O
O516	1.0	0.666300	0.007522	0.069723	Biso	1.000000	O
O517	1.0	0.445330	0.668535	0.462136	Biso	1.000000	O
O518	1.0	0.905404	0.681852	0.406092	Biso	1.000000	O
O519	1.0	0.148748	0.767851	0.939353	Biso	1.000000	O
O520	1.0	0.052623	0.396872	0.859986	Biso	1.000000	O
O521	1.0	0.055076	0.271187	0.182767	Biso	1.000000	O
O522	1.0	0.163702	0.386377	0.946752	Biso	1.000000	O
O523	1.0	0.811575	0.094324	0.631413	Biso	1.000000	O
O524	1.0	0.842517	0.256008	0.044587	Biso	1.000000	O
O525	1.0	0.344751	0.564193	0.680704	Biso	1.000000	O
O526	1.0	0.141207	0.458214	0.668776	Biso	1.000000	O
O527	1.0	0.283845	0.261470	0.093433	Biso	1.000000	O
O528	1.0	0.711008	0.295766	0.066834	Biso	1.000000	O
O529	1.0	0.189147	0.224679	0.305854	Biso	1.000000	O
O530	1.0	0.462937	0.680014	0.742114	Biso	1.000000	O
O531	1.0	0.725154	0.478218	0.240572	Biso	1.000000	O
O532	1.0	0.464861	0.124394	0.998815	Biso	1.000000	O
O533	1.0	0.503770	0.650642	0.630191	Biso	1.000000	O
O534	1.0	0.827727	0.313154	0.735715	Biso	1.000000	O
O535	1.0	0.975664	0.949994	0.267125	Biso	1.000000	O
O536	1.0	0.224324	0.284279	0.097434	Biso	1.000000	O
O537	1.0	0.069375	0.491267	0.407506	Biso	1.000000	O
O538	1.0	0.138347	0.865498	0.449902	Biso	1.000000	O
O539	1.0	0.759355	0.106706	0.227240	Biso	1.000000	O
O540	1.0	0.370556	0.012673	0.491656	Biso	1.000000	O
O541	1.0	0.831655	0.848356	0.901517	Biso	1.000000	O
O542	1.0	0.888879	0.834080	0.595691	Biso	1.000000	O
O543	1.0	0.540668	0.306799	0.096744	Biso	1.000000	O
O544	1.0	0.590076	0.317873	0.136234	Biso	1.000000	O
O545	1.0	0.000818	0.047066	0.344616	Biso	1.000000	O
O546	1.0	0.984297	0.785007	0.631876	Biso	1.000000	O
O547	1.0	0.691800	0.994567	0.186272	Biso	1.000000	O
O548	1.0	0.375274	0.071268	0.846241	Biso	1.000000	O

O549	1.0	0.898197	0.375498	0.369300	Biso	1.000000	O
O550	1.0	0.838660	0.473169	0.411434	Biso	1.000000	O
O551	1.0	0.261209	0.487764	0.402699	Biso	1.000000	O
O552	1.0	0.599544	0.630511	0.692849	Biso	1.000000	O
O553	1.0	0.145992	0.640868	0.098927	Biso	1.000000	O
O554	1.0	0.153553	0.006541	0.362552	Biso	1.000000	O
O555	1.0	0.069358	0.099621	0.782276	Biso	1.000000	O
O556	1.0	0.881897	0.478802	0.711399	Biso	1.000000	O
O557	1.0	0.117095	0.076828	0.635765	Biso	1.000000	O
O558	1.0	0.833720	0.968575	0.951389	Biso	1.000000	O
O559	1.0	0.005862	0.747075	0.253130	Biso	1.000000	O
O560	1.0	0.946745	0.744772	0.787986	Biso	1.000000	O
O561	1.0	0.748106	0.026584	0.934238	Biso	1.000000	O
O562	1.0	0.691933	0.846737	0.278487	Biso	1.000000	O
O563	1.0	0.641272	0.241253	0.984462	Biso	1.000000	O
O564	1.0	0.569995	0.310867	0.276877	Biso	1.000000	O
O565	1.0	0.236373	0.273116	0.767481	Biso	1.000000	O
O566	1.0	0.912107	0.230804	0.891457	Biso	1.000000	O
O567	1.0	0.564145	0.481883	0.984240	Biso	1.000000	O
O568	1.0	0.263483	0.917525	0.978640	Biso	1.000000	O
O569	1.0	0.921655	0.867930	0.005728	Biso	1.000000	O
O570	1.0	0.702928	0.851978	0.015678	Biso	1.000000	O
O571	1.0	0.114559	0.283731	0.202606	Biso	1.000000	O
O572	1.0	0.477947	0.077593	0.519200	Biso	1.000000	O
O573	1.0	0.722959	0.489519	0.061750	Biso	1.000000	O
O574	1.0	0.125385	0.650734	0.338837	Biso	1.000000	O
O575	1.0	0.501412	0.537375	0.050020	Biso	1.000000	O
O576	1.0	0.894211	0.297877	0.428374	Biso	1.000000	O
O577	1.0	0.070998	0.760697	0.790648	Biso	1.000000	O
O578	1.0	0.844685	0.038066	0.166420	Biso	1.000000	O
O579	1.0	0.213292	0.027593	0.692479	Biso	1.000000	O
O580	1.0	0.741935	0.966421	0.904499	Biso	1.000000	O
O581	1.0	0.611612	0.701629	0.986166	Biso	1.000000	O
O582	1.0	0.279667	0.091294	0.785608	Biso	1.000000	O
O583	1.0	0.543860	0.491651	0.605301	Biso	1.000000	O
O584	1.0	0.848266	0.606236	0.660996	Biso	1.000000	O
O585	1.0	0.775971	0.210797	0.197642	Biso	1.000000	O
O586	1.0	0.149462	0.487329	0.497571	Biso	1.000000	O
O587	1.0	0.892399	0.619153	0.515101	Biso	1.000000	O
O588	1.0	0.165487	0.528515	0.673356	Biso	1.000000	O
O589	1.0	0.974940	0.300526	0.507852	Biso	1.000000	O
O590	1.0	0.433075	0.148137	0.737428	Biso	1.000000	O
O591	1.0	0.158874	0.210650	0.994045	Biso	1.000000	O
O592	1.0	0.977972	0.463625	0.592220	Biso	1.000000	O
O593	1.0	0.808196	0.566610	0.692040	Biso	1.000000	O
O594	1.0	0.981273	0.229637	0.093810	Biso	1.000000	O
O595	1.0	0.066202	0.657207	0.159626	Biso	1.000000	O
O596	1.0	0.410967	0.158681	0.268652	Biso	1.000000	O
O597	1.0	0.694306	0.326425	0.475055	Biso	1.000000	O
O598	1.0	0.782233	0.513317	0.251853	Biso	1.000000	O
O599	1.0	0.828994	0.712421	0.273041	Biso	1.000000	O
O600	1.0	0.239661	0.795507	0.563184	Biso	1.000000	O
O601	1.0	0.540644	0.714794	0.416954	Biso	1.000000	O
O602	1.0	0.926365	0.909682	0.502597	Biso	1.000000	O
O603	1.0	0.027175	0.597868	0.401104	Biso	1.000000	O
O604	1.0	0.254399	0.504474	0.229264	Biso	1.000000	O
O605	1.0	0.790061	0.720732	0.128572	Biso	1.000000	O
O606	1.0	0.191508	0.560442	0.151597	Biso	1.000000	O
O607	1.0	0.049304	0.492432	0.465239	Biso	1.000000	O
O608	1.0	0.671744	0.713994	0.967815	Biso	1.000000	O
O609	1.0	0.059710	0.677306	0.752896	Biso	1.000000	O
O610	1.0	0.965559	0.436188	0.708968	Biso	1.000000	O
O611	1.0	0.845987	0.635449	0.181978	Biso	1.000000	O
O612	1.0	0.760556	0.463185	0.290733	Biso	1.000000	O
O613	1.0	0.566898	0.959269	0.884673	Biso	1.000000	O
O614	1.0	0.603856	0.092904	0.884583	Biso	1.000000	O
O615	1.0	0.142603	0.326528	0.162605	Biso	1.000000	O
O616	1.0	0.472183	0.903095	0.558278	Biso	1.000000	O
O617	1.0	0.727855	0.775174	0.676511	Biso	1.000000	O
O618	1.0	0.726247	0.047336	0.688897	Biso	1.000000	O
O619	1.0	0.003705	0.554131	0.722355	Biso	1.000000	O
O620	1.0	0.876984	0.739977	0.305852	Biso	1.000000	O
O621	1.0	0.990410	0.496543	0.315276	Biso	1.000000	O
O622	1.0	0.745087	0.545703	0.733735	Biso	1.000000	O
O623	1.0	0.566708	0.229352	0.817730	Biso	1.000000	O
O624	1.0	0.276310	0.044743	0.451334	Biso	1.000000	O

O625	1.0	0.208587	0.186123	0.063080	Biso	1.000000	O
O626	1.0	0.915409	0.438088	0.673330	Biso	1.000000	O
O627	1.0	0.987959	0.059882	0.579914	Biso	1.000000	O
O628	1.0	0.630137	0.496589	0.883165	Biso	1.000000	O
O629	1.0	0.445059	0.728938	0.494360	Biso	1.000000	O
O630	1.0	0.941832	0.631773	0.735009	Biso	1.000000	O
O631	1.0	0.429045	0.699801	0.694088	Biso	1.000000	O
O632	1.0	0.491085	0.716581	0.454675	Biso	1.000000	O
O633	1.0	0.633745	0.639130	0.218252	Biso	1.000000	O
O634	1.0	0.503826	0.261193	0.753771	Biso	1.000000	O
O635	1.0	0.060911	0.411487	0.768599	Biso	1.000000	O
O636	1.0	0.613326	0.331290	0.056499	Biso	1.000000	O
O637	1.0	0.842241	0.187928	0.032810	Biso	1.000000	O
O638	1.0	0.545791	0.573494	0.559666	Biso	1.000000	O
O639	1.0	0.875038	0.129857	0.043298	Biso	1.000000	O
O640	1.0	0.648659	0.996904	0.959011	Biso	1.000000	O
O641	1.0	0.111542	0.150592	0.786641	Biso	1.000000	O
O642	1.0	0.569228	0.802602	0.880326	Biso	1.000000	O
O643	1.0	0.644150	0.958059	0.106804	Biso	1.000000	O
O644	1.0	0.976588	0.580962	0.644491	Biso	1.000000	O
O645	1.0	0.573155	0.079582	0.827664	Biso	1.000000	O
O646	1.0	0.207210	0.419706	0.457065	Biso	1.000000	O
O647	1.0	0.910967	0.651409	0.462644	Biso	1.000000	O
O648	1.0	0.736749	0.636257	0.621203	Biso	1.000000	O
O649	1.0	0.235725	0.764743	0.977767	Biso	1.000000	O
O650	1.0	0.766996	0.194513	0.764755	Biso	1.000000	O
O651	1.0	0.226126	0.714140	0.090576	Biso	1.000000	O
O652	1.0	0.837572	0.541541	0.642914	Biso	1.000000	O
O653	1.0	0.103913	0.254036	0.350315	Biso	1.000000	O
O654	1.0	0.756346	0.428399	0.995369	Biso	1.000000	O
O655	1.0	0.251139	0.224701	0.319427	Biso	1.000000	O
O656	1.0	0.626462	0.607423	0.164244	Biso	1.000000	O
O657	1.0	0.324458	0.565939	0.342910	Biso	1.000000	O
O658	1.0	0.772182	0.296018	0.813520	Biso	1.000000	O
O659	1.0	0.183663	0.273589	0.262837	Biso	1.000000	O
O660	1.0	0.461916	0.687844	0.805735	Biso	1.000000	O
O661	1.0	0.549387	0.363587	0.590827	Biso	1.000000	O
O662	1.0	0.489300	0.926937	0.959783	Biso	1.000000	O
O663	1.0	0.980259	0.796956	0.327704	Biso	1.000000	O
O664	1.0	0.699798	0.610639	0.959226	Biso	1.000000	O
O665	1.0	0.762966	0.832399	0.004508	Biso	1.000000	O
O666	1.0	0.274445	0.510104	0.910144	Biso	1.000000	O
O667	1.0	0.427390	0.059512	0.938230	Biso	1.000000	O
O668	1.0	0.593692	0.999850	0.926624	Biso	1.000000	O
O669	1.0	0.529062	0.102259	0.099955	Biso	1.000000	O
O670	1.0	0.261162	0.192289	0.029314	Biso	1.000000	O
O671	1.0	0.955972	0.399175	0.384881	Biso	1.000000	O
O672	1.0	0.028604	0.665575	0.385148	Biso	1.000000	O
O673	1.0	0.834109	0.487648	0.604240	Biso	1.000000	O
O674	1.0	0.716954	0.567626	0.784878	Biso	1.000000	O
O675	1.0	0.132318	0.869825	0.197059	Biso	1.000000	O
O676	1.0	0.032982	0.260869	0.071499	Biso	1.000000	O
O677	1.0	0.239163	0.609085	0.295573	Biso	1.000000	O
O678	1.0	0.890758	0.676338	0.206281	Biso	1.000000	O
O679	1.0	0.822256	0.336061	0.913022	Biso	1.000000	O
O680	1.0	0.027295	0.149148	0.802802	Biso	1.000000	O
O681	1.0	0.812882	0.490894	0.201608	Biso	1.000000	O
O682	1.0	0.917072	0.787839	0.329911	Biso	1.000000	O
O683	1.0	0.306281	0.606645	0.836003	Biso	1.000000	O
O684	1.0	0.673330	0.648296	0.070548	Biso	1.000000	O
O685	1.0	0.427380	0.097317	0.551560	Biso	1.000000	O
O686	1.0	0.629974	0.545376	0.603527	Biso	1.000000	O
O687	1.0	0.782437	0.039733	0.663729	Biso	1.000000	O
O688	1.0	0.803115	0.658353	0.744889	Biso	1.000000	O
O689	1.0	0.483269	0.973445	0.635769	Biso	1.000000	O
O690	1.0	0.928966	0.588522	0.248661	Biso	1.000000	O
O691	1.0	0.347621	0.494759	0.784015	Biso	1.000000	O
O692	1.0	0.743045	0.555301	0.903955	Biso	1.000000	O
O693	1.0	0.870542	0.009757	0.468397	Biso	1.000000	O
O694	1.0	0.220118	0.523448	0.703621	Biso	1.000000	O
O695	1.0	0.322248	0.175309	0.028366	Biso	1.000000	O
O696	1.0	0.172737	0.577816	0.095972	Biso	1.000000	O
O697	1.0	0.835606	0.924980	0.621213	Biso	1.000000	O
O698	1.0	0.593455	0.970094	0.597116	Biso	1.000000	O
O699	1.0	0.470124	0.739471	0.550253	Biso	1.000000	O
O700	1.0	0.014218	0.862617	0.428108	Biso	1.000000	O
O701	1.0	0.081376	0.658599	0.966974	Biso	1.000000	O
O702	1.0	0.030465	0.788993	0.066468	Biso	1.000000	O
O703	1.0	0.722247	0.303570	0.127335	Biso	1.000000	O
O704	1.0	0.756664	0.417441	0.739231	Biso	1.000000	O

O705	1.0	0.169172	0.441570	0.120086	Biso	1.000000	O
O706	1.0	0.968840	0.793777	0.126598	Biso	1.000000	O
O707	1.0	0.763683	0.313643	0.338354	Biso	1.000000	O
O708	1.0	0.104853	0.686238	0.683399	Biso	1.000000	O
O709	1.0	0.337391	0.375044	0.125149	Biso	1.000000	O
O710	1.0	0.075072	0.125265	0.276358	Biso	1.000000	O
O711	1.0	0.193600	0.773449	0.524143	Biso	1.000000	O
O712	1.0	0.596207	0.351472	0.771244	Biso	1.000000	O
O713	1.0	0.439241	0.155482	0.077376	Biso	1.000000	O
O714	1.0	0.427111	0.350199	0.949682	Biso	1.000000	O
O715	1.0	0.953085	0.683119	0.192922	Biso	1.000000	O
O716	1.0	0.595825	0.977334	0.252571	Biso	1.000000	O
O717	1.0	0.495014	0.594360	0.663847	Biso	1.000000	O
O718	1.0	0.157763	0.341872	0.858898	Biso	1.000000	O
O719	1.0	0.643228	0.395562	0.852195	Biso	1.000000	O
O720	1.0	0.188493	0.227404	0.755843	Biso	1.000000	O
O721	1.0	0.118462	0.367660	0.487925	Biso	1.000000	O
O722	1.0	0.725428	0.382223	0.030956	Biso	1.000000	O
O723	1.0	0.503318	0.394470	0.623214	Biso	1.000000	O
O724	1.0	0.219024	0.919201	0.551353	Biso	1.000000	O
O725	1.0	0.655486	0.030713	0.534516	Biso	1.000000	O
O726	1.0	0.017145	0.749253	0.017601	Biso	1.000000	O
O727	1.0	0.900074	0.608180	0.695721	Biso	1.000000	O
O728	1.0	0.528083	0.120294	0.508334	Biso	1.000000	O
O729	1.0	0.493502	0.945849	0.727935	Biso	1.000000	O
O730	1.0	0.304041	0.928661	0.790966	Biso	1.000000	O
O731	1.0	0.886095	0.462839	0.075441	Biso	1.000000	O
O732	1.0	0.371111	0.140139	0.852943	Biso	1.000000	O
O733	1.0	0.611536	0.400186	0.061993	Biso	1.000000	O
O734	1.0	0.531772	0.162990	0.067433	Biso	1.000000	O
O735	1.0	0.123328	0.589449	0.815082	Biso	1.000000	O
O736	1.0	0.530416	0.698701	0.300236	Biso	1.000000	O
O737	1.0	0.787979	0.010260	0.297463	Biso	1.000000	O
O738	1.0	0.371301	0.078199	0.470395	Biso	1.000000	O
O739	1.0	0.019663	0.443254	0.343953	Biso	1.000000	O
O740	1.0	0.394610	0.108650	0.770421	Biso	1.000000	O
O741	1.0	0.962108	0.040882	0.296529	Biso	1.000000	O
O742	1.0	0.773152	0.197424	0.257448	Biso	1.000000	O
O743	1.0	0.208841	0.549290	0.210235	Biso	1.000000	O
O744	1.0	0.240252	0.664270	0.781306	Biso	1.000000	O
O745	1.0	0.740858	0.174665	0.157467	Biso	1.000000	O
O746	1.0	0.104041	0.696858	0.012448	Biso	1.000000	O
O747	1.0	0.535038	0.996904	0.605242	Biso	1.000000	O
O748	1.0	0.023687	0.927945	0.232173	Biso	1.000000	O
O749	1.0	0.728950	0.664894	0.213545	Biso	1.000000	O
O750	1.0	0.700758	0.424850	0.070730	Biso	1.000000	O
O751	1.0	0.483168	0.740647	0.769401	Biso	1.000000	O
O752	1.0	0.968734	0.622448	0.324962	Biso	1.000000	O
O753	1.0	0.673182	0.039815	0.794999	Biso	1.000000	O
O754	1.0	0.079556	0.450197	0.360463	Biso	1.000000	O
O755	1.0	0.779791	0.137413	0.558259	Biso	1.000000	O
O756	1.0	0.686482	0.668259	0.727574	Biso	1.000000	O
O757	1.0	0.724627	0.763511	0.615632	Biso	1.000000	O
O758	1.0	0.921517	0.949116	0.838931	Biso	1.000000	O
O759	1.0	0.773842	0.243381	0.972485	Biso	1.000000	O
O760	1.0	0.707769	0.793804	0.895698	Biso	1.000000	O
O761	1.0	0.552514	0.174719	0.542506	Biso	1.000000	O
O762	1.0	0.780368	0.750023	0.647655	Biso	1.000000	O
O763	1.0	0.961678	0.222857	0.289914	Biso	1.000000	O
O764	1.0	0.633962	0.831834	0.301538	Biso	1.000000	O
O765	1.0	0.393128	0.531040	0.811651	Biso	1.000000	O
O766	1.0	0.682411	0.225459	0.340206	Biso	1.000000	O
O767	1.0	0.943182	0.845521	0.352884	Biso	1.000000	O
O768	1.0	0.217092	0.537148	0.785636	Biso	1.000000	O
O769	1.0	0.319315	0.754705	0.033705	Biso	1.000000	O
O770	1.0	0.469179	0.204758	0.741600	Biso	1.000000	O
O771	1.0	0.004755	0.798894	0.213303	Biso	1.000000	O
O772	1.0	0.619152	0.306843	0.988347	Biso	1.000000	O
O773	1.0	0.848895	0.991900	0.523141	Biso	1.000000	O
O774	1.0	0.318358	0.226825	0.423591	Biso	1.000000	O
O775	1.0	0.124338	0.286652	0.878823	Biso	1.000000	O
O776	1.0	0.570881	0.479583	0.865185	Biso	1.000000	O
O777	1.0	0.192251	0.637638	0.745007	Biso	1.000000	O
O778	1.0	0.600284	0.055280	0.025920	Biso	1.000000	O
O779	1.0	0.523606	0.693747	0.133492	Biso	1.000000	O
O780	1.0	0.776262	0.206167	0.921305	Biso	1.000000	O
O781	1.0	0.391584	0.345696	0.672337	Biso	1.000000	O
O782	1.0	0.886714	0.221897	0.470072	Biso	1.000000	O

O783	1.0	0.088125	0.455479	0.936077	Biso	1.000000	O
O784	1.0	0.545603	0.415390	0.665244	Biso	1.000000	O
O785	1.0	0.094297	0.344691	0.861895	Biso	1.000000	O
O786	1.0	0.211709	0.594582	0.487847	Biso	1.000000	O
O787	1.0	0.269340	0.307789	0.725599	Biso	1.000000	O
O788	1.0	0.453639	0.205504	0.901774	Biso	1.000000	O
O789	1.0	0.569944	0.017762	0.558571	Biso	1.000000	O
O790	1.0	0.748792	0.308389	0.183526	Biso	1.000000	O
O791	1.0	0.650358	0.416238	0.952023	Biso	1.000000	O
O792	1.0	0.332473	0.568134	0.282217	Biso	1.000000	O
O793	1.0	0.804800	0.076603	0.769241	Biso	1.000000	O
O794	1.0	0.913765	0.867314	0.643476	Biso	1.000000	O
O795	1.0	0.294618	0.849724	0.697009	Biso	1.000000	O
O796	1.0	0.444321	0.890707	0.818584	Biso	1.000000	O
O797	1.0	0.733841	0.260040	0.019540	Biso	1.000000	O
O798	1.0	0.784381	0.649942	0.185083	Biso	1.000000	O
O799	1.0	0.703466	0.428218	0.388503	Biso	1.000000	O
O800	1.0	0.715415	0.562461	0.849306	Biso	1.000000	O
O801	1.0	0.301220	0.003136	0.359680	Biso	1.000000	O
O802	1.0	0.574450	0.172951	0.920252	Biso	1.000000	O
O803	1.0	0.033060	0.330606	0.856782	Biso	1.000000	O
O804	1.0	0.230027	0.157609	0.831892	Biso	1.000000	O
O805	1.0	0.846846	0.251807	0.426883	Biso	1.000000	O
O806	1.0	0.961775	0.670773	0.430911	Biso	1.000000	O
O807	1.0	0.012909	0.889945	0.119852	Biso	1.000000	O
O808	1.0	0.502788	0.819052	0.519754	Biso	1.000000	O
O809	1.0	0.290516	0.954576	0.845517	Biso	1.000000	O
O810	1.0	0.148418	0.306078	0.490580	Biso	1.000000	O
O811	1.0	0.201408	0.307197	0.413794	Biso	1.000000	O
O812	1.0	0.397429	0.312113	0.991915	Biso	1.000000	O
O813	1.0	0.740638	0.408027	0.435224	Biso	1.000000	O
O814	1.0	0.625734	0.802727	0.851970	Biso	1.000000	O
O815	1.0	0.233402	0.000645	0.022140	Biso	1.000000	O
O816	1.0	0.432091	0.010606	0.894954	Biso	1.000000	O
O817	1.0	0.506598	0.104015	0.040630	Biso	1.000000	O
O818	1.0	0.035405	0.829255	0.476856	Biso	1.000000	O
O819	1.0	0.932908	0.324528	0.643707	Biso	1.000000	O
O820	1.0	0.652045	0.289296	0.364263	Biso	1.000000	O
O821	1.0	0.743282	0.822641	0.942007	Biso	1.000000	O
O822	1.0	0.013549	0.123387	0.588826	Biso	1.000000	O
O823	1.0	0.101217	0.515515	0.358899	Biso	1.000000	O
O824	1.0	0.992002	0.647442	0.641338	Biso	1.000000	O
O825	1.0	0.093848	0.443937	0.627895	Biso	1.000000	O
O826	1.0	0.054627	0.685219	0.291519	Biso	1.000000	O
O827	1.0	0.633311	0.860587	0.625661	Biso	1.000000	O
O828	1.0	0.069373	0.749429	0.851034	Biso	1.000000	O
O829	1.0	0.626250	0.395067	0.734740	Biso	1.000000	O
O830	1.0	0.165708	0.304575	0.109377	Biso	1.000000	O
O831	1.0	0.009931	0.941211	0.545857	Biso	1.000000	O
O832	1.0	0.984107	0.718176	0.651437	Biso	1.000000	O
O833	1.0	0.539771	0.671802	0.582606	Biso	1.000000	O
O834	1.0	0.754940	0.986274	0.086169	Biso	1.000000	O
O835	1.0	0.661844	0.553139	0.817255	Biso	1.000000	O
O836	1.0	0.231230	0.334139	0.365070	Biso	1.000000	O
O837	1.0	0.951391	0.864257	0.121924	Biso	1.000000	O
O838	1.0	0.495475	0.276698	0.010990	Biso	1.000000	O
O839	1.0	0.142709	0.005550	0.560105	Biso	1.000000	O
O840	1.0	0.711264	0.124846	0.189284	Biso	1.000000	O
O841	1.0	0.702038	0.497141	0.710471	Biso	1.000000	O
O842	1.0	0.899928	0.755391	0.503702	Biso	1.000000	O
O843	1.0	0.789861	0.426323	0.203657	Biso	1.000000	O
O844	1.0	0.387391	0.971322	0.864942	Biso	1.000000	O
O845	1.0	0.750064	0.053621	0.097303	Biso	1.000000	O
O846	1.0	0.080691	0.625808	0.376575	Biso	1.000000	O
O847	1.0	0.312055	0.193002	0.747511	Biso	1.000000	O
O848	1.0	0.373880	0.711037	0.520359	Biso	1.000000	O
O849	1.0	0.771203	0.968563	0.958566	Biso	1.000000	O
O850	1.0	0.863108	0.547398	0.794829	Biso	1.000000	O
O851	1.0	0.768298	0.360070	0.990820	Biso	1.000000	O
O852	1.0	0.485102	0.416649	0.743844	Biso	1.000000	O

O853	1.0	0.765089	0.411933	0.379848	Biso	1.000000	O
O854	1.0	0.219485	0.640160	0.242721	Biso	1.000000	O
O855	1.0	0.613348	0.755986	0.895037	Biso	1.000000	O
O856	1.0	0.699736	0.453808	0.974054	Biso	1.000000	O
O857	1.0	0.380226	0.380731	0.618272	Biso	1.000000	O
O858	1.0	0.305107	0.437734	0.126323	Biso	1.000000	O
O859	1.0	0.094606	0.529048	0.803300	Biso	1.000000	O
O860	1.0	0.644589	0.875080	0.004785	Biso	1.000000	O
O861	1.0	0.618915	0.568383	0.711326	Biso	1.000000	O
O862	1.0	0.144775	0.291866	0.668033	Biso	1.000000	O
O863	1.0	0.703956	0.146072	0.026544	Biso	1.000000	O
O864	1.0	0.713570	0.737555	0.861409	Biso	1.000000	O
O865	1.0	0.546003	0.513907	0.087758	Biso	1.000000	O
O866	1.0	0.889607	0.943708	0.542747	Biso	1.000000	O
O867	1.0	0.642967	0.771876	0.800056	Biso	1.000000	O
O868	1.0	0.943235	0.806716	0.491160	Biso	1.000000	O
O869	1.0	0.325072	0.908052	0.684534	Biso	1.000000	O
O870	1.0	0.815481	0.362619	0.085265	Biso	1.000000	O
O871	1.0	0.197036	0.859803	0.575475	Biso	1.000000	O
O872	1.0	0.723209	0.137865	0.969071	Biso	1.000000	O
O873	1.0	0.097416	0.808592	0.749984	Biso	1.000000	O
O874	1.0	0.564717	0.855743	0.199812	Biso	1.000000	O
O875	1.0	0.791659	0.070836	0.543880	Biso	1.000000	O
O876	1.0	0.947857	0.331306	0.374876	Biso	1.000000	O
O877	1.0	0.022940	0.260568	0.131389	Biso	1.000000	O
O878	1.0	0.112488	0.342418	0.110607	Biso	1.000000	O
O879	1.0	0.817329	0.241631	0.236074	Biso	1.000000	O
O880	1.0	0.323205	0.506674	0.309599	Biso	1.000000	O
O881	1.0	0.722096	0.358897	0.087506	Biso	1.000000	O
O882	1.0	0.801238	0.927866	0.671355	Biso	1.000000	O
O883	1.0	0.571754	0.688141	0.531782	Biso	1.000000	O
O884	1.0	0.206909	0.808643	0.016986	Biso	1.000000	O
O885	1.0	0.442003	0.303099	0.669366	Biso	1.000000	O
O886	1.0	0.840127	0.293418	0.635559	Biso	1.000000	O
O887	1.0	0.993305	0.321525	0.452674	Biso	1.000000	O
O888	1.0	0.852094	0.836999	0.546220	Biso	1.000000	O
O889	1.0	0.771225	0.178297	0.822723	Biso	1.000000	O
O890	1.0	0.168458	0.341958	0.276486	Biso	1.000000	O
O891	1.0	0.213335	0.894705	0.946122	Biso	1.000000	O
O892	1.0	0.065765	0.346586	0.749776	Biso	1.000000	O
O893	1.0	0.933034	0.575051	0.599660	Biso	1.000000	O
O894	1.0	0.912094	0.473112	0.174591	Biso	1.000000	O
O895	1.0	0.282441	0.658926	0.316961	Biso	1.000000	O
O896	1.0	0.019907	0.301786	0.910820	Biso	1.000000	O
O897	1.0	0.129306	0.068243	0.694662	Biso	1.000000	O
O898	1.0	0.910796	0.815644	0.148315	Biso	1.000000	O
O899	1.0	0.749796	0.233694	0.488553	Biso	1.000000	O
O900	1.0	0.786260	0.389234	0.591396	Biso	1.000000	O
O901	1.0	0.638929	0.402343	0.675749	Biso	1.000000	O
O902	1.0	0.766098	0.918863	0.095915	Biso	1.000000	O
O903	1.0	0.940082	0.761759	0.670324	Biso	1.000000	O
O904	1.0	0.176883	0.519566	0.574645	Biso	1.000000	O
O905	1.0	0.162193	0.585392	0.559347	Biso	1.000000	O
O906	1.0	0.225711	0.684541	0.486415	Biso	1.000000	O
O907	1.0	0.545330	0.362622	0.809626	Biso	1.000000	O
O908	1.0	0.883325	0.048960	0.517510	Biso	1.000000	O
O909	1.0	0.145623	0.961684	0.976352	Biso	1.000000	O
O910	1.0	0.154058	0.211653	0.256731	Biso	1.000000	O
O911	1.0	0.341387	0.742380	0.380327	Biso	1.000000	O
O912	1.0	0.904456	0.877196	0.551072	Biso	1.000000	O
O913	1.0	0.286309	0.792537	0.793609	Biso	1.000000	O
O914	1.0	0.995830	0.199153	0.877611	Biso	1.000000	O
O915	1.0	0.846466	0.980423	0.392062	Biso	1.000000	O
O916	1.0	0.092464	0.636927	0.484603	Biso	1.000000	O
O917	1.0	0.708477	0.977230	0.955986	Biso	1.000000	O
O918	1.0	0.233889	0.059771	0.054147	Biso	1.000000	O
O919	1.0	0.033160	0.146684	0.535276	Biso	1.000000	O
O920	1.0	0.879177	0.293828	0.155543	Biso	1.000000	O
O921	1.0	0.831310	0.376098	0.466138	Biso	1.000000	O
O922	1.0	0.734457	0.141469	0.079568	Biso	1.000000	O
O923	1.0	0.663099	0.475702	0.046172	Biso	1.000000	O
O924	1.0	0.668762	0.292518	0.953043	Biso	1.000000	O
O925	1.0	0.873235	0.237123	0.776638	Biso	1.000000	O
O926	1.0	0.526536	0.432431	0.960444	Biso	1.000000	O
O927	1.0	0.206633	0.635884	0.397470	Biso	1.000000	O
O928	1.0	0.123028	0.713937	0.369888	Biso	1.000000	O
O929	1.0	0.375513	0.766857	0.750099	Biso	1.000000	O
O930	1.0	0.096409	0.762121	0.131896	Biso	1.000000	O
O931	1.0	0.779946	0.170204	0.312824	Biso	1.000000	O

O932	1.0	0.669891	0.058454	0.176394	Biso	1.000000	O
O933	1.0	0.265290	0.669297	0.549295	Biso	1.000000	O
O934	1.0	0.126676	0.213648	0.766306	Biso	1.000000	O
O935	1.0	0.580302	0.294556	0.901721	Biso	1.000000	O
O936	1.0	0.633427	0.557955	0.119065	Biso	1.000000	O
O937	1.0	0.190845	0.975120	0.403534	Biso	1.000000	O
O938	1.0	0.518607	0.136839	0.654033	Biso	1.000000	O
O939	1.0	0.268711	0.193694	0.449684	Biso	1.000000	O
O940	1.0	0.211401	0.187045	0.125609	Biso	1.000000	O
O941	1.0	0.042261	0.805792	0.315175	Biso	1.000000	O
O942	1.0	0.045708	0.768354	0.733815	Biso	1.000000	O
O943	1.0	0.023461	0.064877	0.102430	Biso	1.000000	O
O944	1.0	0.125387	0.047076	0.054214	Biso	1.000000	O
O945	1.0	0.834763	0.887588	0.039765	Biso	1.000000	O
O946	1.0	0.061979	0.682252	0.638528	Biso	1.000000	O
O947	1.0	0.298260	0.781696	0.979377	Biso	1.000000	O
O948	1.0	0.918896	0.125281	0.361046	Biso	1.000000	O
O949	1.0	0.968334	0.929740	0.876268	Biso	1.000000	O
O950	1.0	0.823928	0.387234	0.640850	Biso	1.000000	O
O951	1.0	0.319848	0.100270	0.862452	Biso	1.000000	O
O952	1.0	0.581422	0.116403	0.956592	Biso	1.000000	O
O953	1.0	0.544316	0.203601	0.327747	Biso	1.000000	O
O954	1.0	0.903142	0.609055	0.195658	Biso	1.000000	O
O955	1.0	0.699122	0.979784	0.542266	Biso	1.000000	O
O956	1.0	0.785863	0.030209	0.050118	Biso	1.000000	O
O957	1.0	0.617021	0.264550	0.275278	Biso	1.000000	O
O958	1.0	0.125264	0.594601	0.057811	Biso	1.000000	O
O959	1.0	0.185567	0.715240	0.448085	Biso	1.000000	O
O960	1.0	0.380480	0.835380	0.741192	Biso	1.000000	O
O961	1.0	0.879182	0.121397	0.103181	Biso	1.000000	O
O962	1.0	0.217941	0.408252	0.146210	Biso	1.000000	O
O963	1.0	0.291542	0.996228	0.796183	Biso	1.000000	O
O964	1.0	0.672508	0.518147	0.312503	Biso	1.000000	O
O965	1.0	0.079174	0.322249	0.580822	Biso	1.000000	O
O966	1.0	0.679567	0.862333	0.178386	Biso	1.000000	O
O967	1.0	0.443732	0.736074	0.606288	Biso	1.000000	O
O968	1.0	0.011495	0.214815	0.818362	Biso	1.000000	O
O969	1.0	0.822429	0.063107	0.008682	Biso	1.000000	O
O970	1.0	0.953908	0.508817	0.140193	Biso	1.000000	O
O971	1.0	0.410922	0.241402	0.498251	Biso	1.000000	O
O972	1.0	0.563026	0.054219	0.978194	Biso	1.000000	O
O973	1.0	0.123591	0.201471	0.825674	Biso	1.000000	O
O974	1.0	0.165568	0.458049	0.550194	Biso	1.000000	O
O975	1.0	0.008909	0.541554	0.161030	Biso	1.000000	O
O976	1.0	0.279839	0.671573	0.829544	Biso	1.000000	O
O977	1.0	0.788325	0.048383	0.246242	Biso	1.000000	O
O978	1.0	0.865128	0.991027	0.580841	Biso	1.000000	O
O979	1.0	0.215940	0.702529	0.216548	Biso	1.000000	O
O980	1.0	0.235396	0.999084	0.083499	Biso	1.000000	O
O981	1.0	0.316844	0.096898	0.444517	Biso	1.000000	O
O982	1.0	0.199901	0.832728	0.118311	Biso	1.000000	O
O983	1.0	0.154359	0.624440	0.489180	Biso	1.000000	O
O984	1.0	0.556916	0.483878	0.664505	Biso	1.000000	O
O985	1.0	0.163246	0.711224	0.096820	Biso	1.000000	O
O986	1.0	0.490799	0.413146	0.142372	Biso	1.000000	O
O987	1.0	0.177719	0.237204	0.096342	Biso	1.000000	O
O988	1.0	0.125665	0.117773	0.062067	Biso	1.000000	O
O989	1.0	0.351563	0.632860	0.769656	Biso	1.000000	O
O990	1.0	0.018943	0.610488	0.166541	Biso	1.000000	O
O991	1.0	0.521813	0.261857	0.353656	Biso	1.000000	O
O992	1.0	0.487351	0.824287	0.461344	Biso	1.000000	O
O993	1.0	0.960207	0.092665	0.255763	Biso	1.000000	O
O994	1.0	0.259132	0.848910	0.100771	Biso	1.000000	O
O995	1.0	0.197929	0.756224	0.303567	Biso	1.000000	O
O996	1.0	0.821881	0.173891	0.225081	Biso	1.000000	O
O997	1.0	0.534086	0.969914	0.832097	Biso	1.000000	O
O998	1.0	0.591845	0.551214	0.221990	Biso	1.000000	O
O999	1.0	0.729184	0.857570	0.763384	Biso	1.000000	O
O1000	1.0	0.170298	0.043967	0.737659	Biso	1.000000	O
O1001	1.0	0.093865	0.896021	0.111818	Biso	1.000000	O
O1002	1.0	0.226828	0.627381	0.628577	Biso	1.000000	O
O1003	1.0	0.013389	0.463517	0.421980	Biso	1.000000	O
O1004	1.0	0.530014	0.580992	0.090211	Biso	1.000000	O
O1005	1.0	0.926774	0.617105	0.411175	Biso	1.000000	O
O1006	1.0	0.565793	0.817579	0.523789	Biso	1.000000	O
O1007	1.0	0.106039	0.826833	0.108798	Biso	1.000000	O
O1008	1.0	0.761511	0.817673	0.636904	Biso	1.000000	O
O1009	1.0	0.516624	0.379025	0.996963	Biso	1.000000	O
O1010	1.0	0.007580	0.363243	0.499242	Biso	1.000000	O
O1011	1.0	0.726193	0.042116	0.152348	Biso	1.000000	O

O1012	1.0	0.134306	0.688149	0.474860	Biso	1.000000	O
O1013	1.0	0.765985	0.603920	0.762155	Biso	1.000000	O
O1014	1.0	0.113229	0.532021	0.300762	Biso	1.000000	O
O1015	1.0	0.263655	0.219027	0.393565	Biso	1.000000	O
O1016	1.0	0.763124	0.298848	0.242537	Biso	1.000000	O
O1017	1.0	0.302202	0.722876	0.564246	Biso	1.000000	O
O1018	1.0	0.683474	0.532256	0.894915	Biso	1.000000	O
O1019	1.0	0.102928	0.089375	0.010669	Biso	1.000000	O
O1020	1.0	0.616323	0.020705	0.105302	Biso	1.000000	O
O1021	1.0	0.852743	0.429426	0.679572	Biso	1.000000	O
O1022	1.0	0.604193	0.273184	0.849181	Biso	1.000000	O
O1023	1.0	0.642466	0.903414	0.144082	Biso	1.000000	O
O1024	1.0	0.953796	0.380858	0.137445	Biso	1.000000	O
O1025	1.0	0.844572	0.165680	0.297560	Biso	1.000000	O
O1026	1.0	0.112095	0.338834	0.631371	Biso	1.000000	O
O1027	1.0	0.954281	0.760448	0.534578	Biso	1.000000	O
O1028	1.0	0.275245	0.378196	0.147224	Biso	1.000000	O
O1029	1.0	0.016930	0.627873	0.285278	Biso	1.000000	O
O1030	1.0	0.717578	0.276287	0.837798	Biso	1.000000	O
O1031	1.0	0.641654	0.701753	0.868512	Biso	1.000000	O
O1032	1.0	0.968803	0.150800	0.221517	Biso	1.000000	O
O1033	1.0	0.249679	0.207094	0.756640	Biso	1.000000	O
O1034	1.0	0.178349	0.292428	0.202623	Biso	1.000000	O
O1035	1.0	0.652109	0.216621	0.604100	Biso	1.000000	O
O1036	1.0	0.224726	0.958186	0.700018	Biso	1.000000	O
O1037	1.0	0.065147	0.360262	0.810105	Biso	1.000000	O
O1038	1.0	0.066069	0.019668	0.479437	Biso	1.000000	O
O1039	1.0	0.602810	0.464076	0.031358	Biso	1.000000	O
O1040	1.0	0.761698	0.127901	0.781079	Biso	1.000000	O
O1041	1.0	0.942760	0.880204	0.062541	Biso	1.000000	O
O1042	1.0	0.717965	0.273268	0.313613	Biso	1.000000	O
O1043	1.0	0.765888	0.842880	0.809877	Biso	1.000000	O
O1044	1.0	0.922556	0.987872	0.609344	Biso	1.000000	O
O1045	1.0	0.851507	0.027036	0.700233	Biso	1.000000	O
O1046	1.0	0.992869	0.705142	0.546570	Biso	1.000000	O
O1047	1.0	0.771877	0.490021	0.101806	Biso	1.000000	O
O1048	1.0	0.225200	0.539289	0.103449	Biso	1.000000	O
O1049	1.0	0.720821	0.865963	0.703489	Biso	1.000000	O
O1050	1.0	0.847955	0.726798	0.575855	Biso	1.000000	O
O1051	1.0	0.484836	0.383472	0.943467	Biso	1.000000	O
O1052	1.0	0.676541	0.183513	0.984137	Biso	1.000000	O
O1053	1.0	0.989548	0.559439	0.948805	Biso	1.000000	O
O1054	1.0	0.687166	0.527848	0.578475	Biso	1.000000	O
O1055	1.0	0.972060	0.894324	0.646855	Biso	1.000000	O
O1056	1.0	0.985190	0.686925	0.344828	Biso	1.000000	O
O1057	1.0	0.007529	0.371998	0.411498	Biso	1.000000	O
O1058	1.0	0.809219	0.623405	0.323724	Biso	1.000000	O
O1059	1.0	0.424788	0.551549	0.593225	Biso	1.000000	O
O1060	1.0	0.786857	0.505119	0.332924	Biso	1.000000	O
O1061	1.0	0.722254	0.736823	0.998903	Biso	1.000000	O
O1062	1.0	0.806728	0.362841	0.360001	Biso	1.000000	O
O1063	1.0	0.659158	0.720103	0.761638	Biso	1.000000	O
O1064	1.0	0.810775	0.686803	0.078031	Biso	1.000000	O
O1065	1.0	0.030382	0.747691	0.898857	Biso	1.000000	O
O1066	1.0	0.662372	0.932561	0.975175	Biso	1.000000	O
O1067	1.0	0.571354	0.296610	0.802210	Biso	1.000000	O
O1068	1.0	0.471441	0.426804	0.990190	Biso	1.000000	O
O1069	1.0	0.200327	0.868855	0.471186	Biso	1.000000	O
O1070	1.0	0.791136	0.210469	0.139279	Biso	1.000000	O
O1071	1.0	0.926856	0.948419	0.306184	Biso	1.000000	O
O1072	1.0	0.911046	0.452146	0.232450	Biso	1.000000	O
O1073	1.0	0.278947	0.443497	0.208454	Biso	1.000000	O
O1074	1.0	0.159683	0.033973	0.417730	Biso	1.000000	O
O1075	1.0	0.696232	0.776939	0.955869	Biso	1.000000	O
O1076	1.0	0.129428	0.479913	0.154995	Biso	1.000000	O
O1077	1.0	0.002952	0.765916	0.574237	Biso	1.000000	O
O1078	1.0	0.419717	0.054708	0.504174	Biso	1.000000	O
O1079	1.0	0.691995	0.960840	0.822090	Biso	1.000000	O
O1080	1.0	0.742446	0.857184	0.110344	Biso	1.000000	O
O1081	1.0	0.714448	0.101481	0.845408	Biso	1.000000	O

O1082	1.0	0.528689	0.171847	0.810776	Biso	1.000000	O
O1083	1.0	0.016688	0.741969	0.313079	Biso	1.000000	O
O1084	1.0	0.807426	0.996072	0.001388	Biso	1.000000	O
O1085	1.0	0.575273	0.677143	0.339887	Biso	1.000000	O
O1086	1.0	0.182328	0.017271	0.054550	Biso	1.000000	O
O1087	1.0	0.081026	0.433588	0.993603	Biso	1.000000	O
O1088	1.0	0.016689	0.198239	0.760071	Biso	1.000000	O
O1089	1.0	0.563622	0.313681	0.959111	Biso	1.000000	O
O1090	1.0	0.509057	0.486256	0.554045	Biso	1.000000	O
O1091	1.0	0.209268	0.777668	0.702840	Biso	1.000000	O
O1092	1.0	0.164254	0.233496	0.863955	Biso	1.000000	O
O1093	1.0	0.054708	0.483907	0.529153	Biso	1.000000	O
O1094	1.0	0.777320	0.348426	0.443601	Biso	1.000000	O
O1095	1.0	0.076914	0.851586	0.435149	Biso	1.000000	O
O1096	1.0	0.640621	0.271676	0.899665	Biso	1.000000	O
O1097	1.0	0.954182	0.633119	0.502435	Biso	1.000000	O
O1098	1.0	0.510991	0.267976	0.952827	Biso	1.000000	O
O1099	1.0	0.695591	0.600331	0.897457	Biso	1.000000	O
O1100	1.0	0.121697	0.848856	0.393289	Biso	1.000000	O
O1101	1.0	0.679436	0.440981	0.817727	Biso	1.000000	O
O1102	1.0	0.653300	0.986984	0.583114	Biso	1.000000	O
O1103	1.0	0.154464	0.781039	0.115068	Biso	1.000000	O
O1104	1.0	0.269619	0.189111	0.701486	Biso	1.000000	O
O1105	1.0	0.681335	0.228325	0.869684	Biso	1.000000	O
O1106	1.0	0.288523	0.784923	0.372573	Biso	1.000000	O
O1107	1.0	0.710976	0.604488	0.732116	Biso	1.000000	O
O1108	1.0	0.901206	0.843253	0.245735	Biso	1.000000	O
O1109	1.0	0.486227	0.550568	0.569222	Biso	1.000000	O
O1110	1.0	0.745729	0.958363	0.141781	Biso	1.000000	O
O1111	1.0	0.668888	0.126044	0.661265	Biso	1.000000	O
O1112	1.0	0.246904	0.734749	0.536591	Biso	1.000000	O
O1113	1.0	0.023144	0.454673	0.837223	Biso	1.000000	O
O1114	1.0	0.905556	0.739412	0.624056	Biso	1.000000	O
O1115	1.0	0.108770	0.316457	0.712840	Biso	1.000000	O
O1116	1.0	0.158637	0.747864	0.620960	Biso	1.000000	O
O1117	1.0	0.796471	0.272814	0.920579	Biso	1.000000	O
O1118	1.0	0.686589	0.148834	0.136832	Biso	1.000000	O
O1119	1.0	0.484271	0.058048	0.000942	Biso	1.000000	O
O1120	1.0	0.291064	0.085986	0.042705	Biso	1.000000	O
O1121	1.0	0.047064	0.949041	0.119085	Biso	1.000000	O
O1122	1.0	0.332520	0.495546	0.104571	Biso	1.000000	O
O1123	1.0	0.642567	0.865241	0.749712	Biso	1.000000	O
O1124	1.0	0.183934	0.581159	0.709485	Biso	1.000000	O
O1125	1.0	0.215122	0.469077	0.589888	Biso	1.000000	O
O1126	1.0	0.304715	0.302253	0.190593	Biso	1.000000	O
O1127	1.0	0.751660	0.885250	0.653434	Biso	1.000000	O
O1128	1.0	0.698190	0.035816	0.578578	Biso	1.000000	O
O1129	1.0	0.678859	0.841918	0.932295	Biso	1.000000	O
O1130	1.0	0.484318	0.539203	0.699112	Biso	1.000000	O
O1131	1.0	0.043221	0.284393	0.542549	Biso	1.000000	O
O1132	1.0	0.601431	0.749140	0.758896	Biso	1.000000	O
O1133	1.0	0.353617	0.877131	0.047763	Biso	1.000000	O
O1134	1.0	0.000630	0.565672	0.307322	Biso	1.000000	O
O1135	1.0	0.132796	0.990861	0.306371	Biso	1.000000	O
O1136	1.0	0.499648	0.992351	0.982703	Biso	1.000000	O
O1137	1.0	0.051066	0.548298	0.761721	Biso	1.000000	O
O1138	1.0	0.699332	0.040496	0.870107	Biso	1.000000	O
O1139	1.0	0.106134	0.357540	0.271717	Biso	1.000000	O
O1140	1.0	0.100472	0.261632	0.559800	Biso	1.000000	O
O1141	1.0	0.621724	0.585173	0.652832	Biso	1.000000	O
O1142	1.0	0.875714	0.667600	0.674050	Biso	1.000000	O
O1143	1.0	0.454492	0.916279	0.615733	Biso	1.000000	O
O1144	1.0	0.614762	0.328717	0.404096	Biso	1.000000	O
O1145	1.0	0.480623	0.036765	0.605986	Biso	1.000000	O
O1146	1.0	0.350436	0.495400	0.674385	Biso	1.000000	O
O1147	1.0	0.788004	0.190347	0.597012	Biso	1.000000	O
O1148	1.0	0.726373	0.088438	0.904944	Biso	1.000000	O
O1149	1.0	0.621448	0.456341	0.834452	Biso	1.000000	O
O1150	1.0	0.156277	0.268240	0.382175	Biso	1.000000	O
O1151	1.0	0.815205	0.187426	0.725722	Biso	1.000000	O
O1152	1.0	0.263909	0.075862	0.727574	Biso	1.000000	O
O1153	1.0	0.071821	0.924434	0.547669	Biso	1.000000	O
O1154	1.0	0.849914	0.582024	0.444802	Biso	1.000000	O

O1155	1.0	0.137385	0.468631	0.729113	Biso	1.000000	O
O1156	1.0	0.345782	0.970421	0.819044	Biso	1.000000	O
O1157	1.0	0.932783	0.931867	0.445340	Biso	1.000000	O
O1158	1.0	0.193823	0.644939	0.994441	Biso	1.000000	O
O1159	1.0	0.974504	0.244840	0.547353	Biso	1.000000	O
O1160	1.0	0.590414	0.546684	0.970161	Biso	1.000000	O
O1161	1.0	0.153917	0.190483	0.715573	Biso	1.000000	O
O1162	1.0	0.629246	0.881785	0.946988	Biso	1.000000	O
O1163	1.0	0.752143	0.677808	0.503836	Biso	1.000000	O
O1164	1.0	0.758341	0.162403	0.489607	Biso	1.000000	O
O1165	1.0	0.629769	0.028991	0.750385	Biso	1.000000	O
O1166	1.0	0.682716	0.598017	0.455815	Biso	1.000000	O
O1167	1.0	0.266501	0.568961	0.622454	Biso	1.000000	O
O1168	1.0	0.725196	0.521793	0.346820	Biso	1.000000	O
O1169	1.0	0.992808	0.256200	0.737268	Biso	1.000000	O
O1170	1.0	0.676025	0.288071	0.013716	Biso	1.000000	O
O1171	1.0	0.503646	0.350653	0.166899	Biso	1.000000	O
O1172	1.0	0.220594	0.224214	0.808348	Biso	1.000000	O
O1173	1.0	0.097820	0.220088	0.185561	Biso	1.000000	O
O1174	1.0	0.730270	0.744066	0.134699	Biso	1.000000	O
O1175	1.0	0.021250	0.532478	0.422395	Biso	1.000000	O
O1176	1.0	0.211763	0.444240	0.400063	Biso	1.000000	O
O1177	1.0	0.318383	0.493984	0.728728	Biso	1.000000	O
O1178	1.0	0.898000	0.406127	0.789223	Biso	1.000000	O
O1179	1.0	0.427558	0.177133	0.212363	Biso	1.000000	O
O1180	1.0	0.569635	0.449749	0.083935	Biso	1.000000	O
O1181	1.0	0.673554	0.013195	0.130384	Biso	1.000000	O
O1182	1.0	0.869292	0.105786	0.606774	Biso	1.000000	O
O1183	1.0	0.213817	0.985462	0.640987	Biso	1.000000	O
O1184	1.0	0.871857	0.010965	0.265414	Biso	1.000000	O
O1185	1.0	0.360424	0.234636	0.734287	Biso	1.000000	O
O1186	1.0	0.368023	0.211186	0.460751	Biso	1.000000	O
O1187	1.0	0.500748	0.618159	0.576646	Biso	1.000000	O
O1188	1.0	0.872892	0.096436	0.394832	Biso	1.000000	O
O1189	1.0	0.690712	0.039506	0.737683	Biso	1.000000	O
O1190	1.0	0.794594	0.251420	0.738733	Biso	1.000000	O
O1191	1.0	0.124115	0.914128	0.016052	Biso	1.000000	O
O1192	1.0	0.858857	0.225917	0.586047	Biso	1.000000	O
O1193	1.0	0.879130	0.188011	0.248290	Biso	1.000000	O
O1194	1.0	0.324981	0.359543	0.067783	Biso	1.000000	O
O1195	1.0	0.981972	0.555782	0.379539	Biso	1.000000	O
O1196	1.0	0.648903	0.328693	0.205660	Biso	1.000000	O
O1197	1.0	0.742073	0.520569	0.608730	Biso	1.000000	O
O1198	1.0	0.118818	0.472515	0.095445	Biso	1.000000	O
O1199	1.0	0.628889	0.183320	0.121432	Biso	1.000000	O
O1200	1.0	0.513184	0.232623	0.458092	Biso	1.000000	O
O1201	1.0	0.874737	0.485305	0.650935	Biso	1.000000	O
O1202	1.0	0.808469	0.826850	0.676615	Biso	1.000000	O
O1203	1.0	0.035230	0.610739	0.739014	Biso	1.000000	O
O1204	1.0	0.656253	0.835350	0.053978	Biso	1.000000	O
O1205	1.0	0.960546	0.724279	0.144397	Biso	1.000000	O
O1206	1.0	0.844887	0.493522	0.353880	Biso	1.000000	O
O1207	1.0	0.581439	0.228458	0.669600	Biso	1.000000	O
O1208	1.0	0.636404	0.023165	0.276658	Biso	1.000000	O
O1209	1.0	0.968476	0.525048	0.678798	Biso	1.000000	O
O1210	1.0	0.265399	0.535124	0.745920	Biso	1.000000	O
O1211	1.0	0.062594	0.695947	0.812170	Biso	1.000000	O
O1212	1.0	0.246670	0.721976	0.710611	Biso	1.000000	O
O1213	1.0	0.659599	0.158773	0.714959	Biso	1.000000	O
O1214	1.0	0.852153	0.700139	0.722119	Biso	1.000000	O
O1215	1.0	0.854804	0.443678	0.759724	Biso	1.000000	O
O1216	1.0	0.868490	0.528832	0.070637	Biso	1.000000	O
O1217	1.0	0.903985	0.622378	0.636267	Biso	1.000000	O
O1218	1.0	0.419490	0.698642	0.561145	Biso	1.000000	O
O1219	1.0	0.476778	0.655250	0.686379	Biso	1.000000	O
O1220	1.0	0.626492	0.966459	0.808876	Biso	1.000000	O
O1221	1.0	0.402228	0.815362	0.551126	Biso	1.000000	O
O1222	1.0	0.126320	0.017651	0.502310	Biso	1.000000	O
O1223	1.0	0.194199	0.494203	0.245364	Biso	1.000000	O

O1224	1.0	0.591904	0.439764	0.700117	Biso	1.000000	O
O1225	1.0	0.760361	0.324395	0.899932	Biso	1.000000	O
O1226	1.0	0.072690	0.741307	0.047018	Biso	1.000000	O
O1227	1.0	0.537664	0.017655	0.902917	Biso	1.000000	O
O1228	1.0	0.538471	0.643155	0.680086	Biso	1.000000	O
O1229	1.0	0.956833	0.869617	0.461860	Biso	1.000000	O
O1230	1.0	0.852069	0.712616	0.119354	Biso	1.000000	O
O1231	1.0	0.006432	0.267062	0.860196	Biso	1.000000	O
O1232	1.0	0.160574	0.418482	0.500601	Biso	1.000000	O
O1233	1.0	0.031974	0.889853	0.510776	Biso	1.000000	O
O1234	1.0	0.799660	0.564320	0.479256	Biso	1.000000	O
O1235	1.0	0.357936	0.275733	0.482116	Biso	1.000000	O
O1236	1.0	0.733150	0.611966	0.490197	Biso	1.000000	O
O1237	1.0	0.205006	0.489202	0.828348	Biso	1.000000	O
O1238	1.0	0.263305	0.880280	0.472972	Biso	1.000000	O
O1239	1.0	0.191307	0.017173	0.315373	Biso	1.000000	O
O1240	1.0	0.101149	0.822898	0.340583	Biso	1.000000	O
O1241	1.0	0.575813	0.541541	0.634148	Biso	1.000000	O
O1242	1.0	0.111085	0.710007	0.307971	Biso	1.000000	O
O1243	1.0	0.349454	0.048000	0.358085	Biso	1.000000	O
O1244	1.0	0.273010	0.784704	0.704634	Biso	1.000000	O
O1245	1.0	0.891804	0.801291	0.649307	Biso	1.000000	O
O1246	1.0	0.479212	0.878907	0.499771	Biso	1.000000	O
O1247	1.0	0.009649	0.950538	0.662436	Biso	1.000000	O
O1248	1.0	0.233978	0.703331	0.322591	Biso	1.000000	O
O1249	1.0	0.524945	0.070041	0.865749	Biso	1.000000	O
O1250	1.0	0.210802	0.505814	0.369999	Biso	1.000000	O
O1251	1.0	0.095686	0.209054	0.994278	Biso	1.000000	O
O1252	1.0	0.945322	0.680133	0.294412	Biso	1.000000	O
O1253	1.0	0.191410	0.571148	0.382236	Biso	1.000000	O
O1254	1.0	0.016489	0.598841	0.601347	Biso	1.000000	O
O1255	1.0	0.274258	0.560534	0.802260	Biso	1.000000	O
O1256	1.0	0.562572	0.555863	0.042883	Biso	1.000000	O
O1257	1.0	0.318331	0.887669	0.997561	Biso	1.000000	O
O1258	1.0	0.582438	0.991711	0.002956	Biso	1.000000	O
O1259	1.0	0.746232	0.073509	0.741496	Biso	1.000000	O
O1260	1.0	0.145622	0.400032	0.631947	Biso	1.000000	O
O1261	1.0	0.565077	0.976043	0.447624	Biso	1.000000	O
O1262	1.0	0.869479	0.164173	0.380849	Biso	1.000000	O
O1263	1.0	0.792088	0.408781	0.689548	Biso	1.000000	O
O1264	1.0	0.977119	0.580010	0.436043	Biso	1.000000	O
O1265	1.0	0.951008	0.232716	0.843469	Biso	1.000000	O
O1266	1.0	0.702190	0.324244	0.729146	Biso	1.000000	O
O1267	1.0	0.948473	0.446129	0.767745	Biso	1.000000	O
O1268	1.0	0.272342	0.438999	0.579968	Biso	1.000000	O
O1269	1.0	0.657318	0.976508	0.235625	Biso	1.000000	O
O1270	1.0	0.047537	0.955967	0.498544	Biso	1.000000	O
O1271	1.0	0.728605	0.303348	0.674482	Biso	1.000000	O
O1272	1.0	0.403839	0.943821	0.811413	Biso	1.000000	O
O1273	1.0	0.661331	0.615432	0.694987	Biso	1.000000	O
O1274	1.0	0.966671	0.835137	0.177123	Biso	1.000000	O
O1275	1.0	0.213299	0.375536	0.410783	Biso	1.000000	O
O1276	1.0	0.210848	0.855219	0.061516	Biso	1.000000	O
O1277	1.0	0.155098	0.882742	0.616812	Biso	1.000000	O
O1278	1.0	0.788874	0.623478	0.516567	Biso	1.000000	O
O1279	1.0	0.808948	0.300234	0.199966	Biso	1.000000	O
O1280	1.0	0.214739	0.568496	0.657457	Biso	1.000000	O
O1281	1.0	0.732159	0.334439	0.289424	Biso	1.000000	O
O1282	1.0	0.033282	0.517526	0.813449	Biso	1.000000	O
O1283	1.0	0.835661	0.658842	0.437683	Biso	1.000000	O
O1284	1.0	0.958233	0.437899	0.330224	Biso	1.000000	O
O1285	1.0	0.163332	0.828536	0.351289	Biso	1.000000	O
O1286	1.0	0.035622	0.602715	0.659788	Biso	1.000000	O
O1287	1.0	0.883155	0.730292	0.245437	Biso	1.000000	O
O1288	1.0	0.737333	0.196216	0.999294	Biso	1.000000	O
O1289	1.0	0.800730	0.861325	0.085342	Biso	1.000000	O
O1290	1.0	0.836186	0.510744	0.294608	Biso	1.000000	O
O1291	1.0	0.949035	0.928845	0.557334	Biso	1.000000	O
O1292	1.0	0.175271	0.753951	0.996177	Biso	1.000000	O

O1293	1.0	0.093916	0.797797	0.961597	Biso	1.000000	O
O1294	1.0	0.207758	0.164920	0.005182	Biso	1.000000	O
O1295	1.0	0.696577	0.739634	0.485639	Biso	1.000000	O
O1296	1.0	0.092208	0.678476	0.584532	Biso	1.000000	O
O1297	1.0	0.734640	0.438226	0.684697	Biso	1.000000	O
O1298	1.0	0.109068	0.770365	0.590594	Biso	1.000000	O
O1299	1.0	0.091570	0.717457	0.250468	Biso	1.000000	O
O1300	1.0	0.215432	0.899178	0.108674	Biso	1.000000	O
O1301	1.0	0.815148	0.658542	0.133588	Biso	1.000000	O
O1302	1.0	0.125681	0.094146	0.753710	Biso	1.000000	O
O1303	1.0	0.341478	0.881356	0.765893	Biso	1.000000	O
O1304	1.0	0.828484	0.557412	0.338884	Biso	1.000000	O
O1305	1.0	0.127526	0.810092	0.163978	Biso	1.000000	O
O1306	1.0	0.873470	0.605866	0.318728	Biso	1.000000	O
O1307	1.0	0.585494	0.551928	0.161713	Biso	1.000000	O
O1308	1.0	0.029903	0.672005	0.584345	Biso	1.000000	O
O1309	1.0	0.324440	0.413190	0.610781	Biso	1.000000	O
O1310	1.0	0.703633	0.578562	0.317617	Biso	1.000000	O
O1311	1.0	0.266516	0.303878	0.139445	Biso	1.000000	O
O1312	1.0	0.962494	0.582767	0.179946	Biso	1.000000	O
O1313	1.0	0.053541	0.853962	0.082716	Biso	1.000000	O
O1314	1.0	0.012735	0.571075	0.216521	Biso	1.000000	O
O1315	1.0	0.779179	0.642347	0.459220	Biso	1.000000	O
O1316	1.0	0.848888	0.542016	0.733433	Biso	1.000000	O
O1317	1.0	0.481679	0.051711	0.906609	Biso	1.000000	O
O1318	1.0	0.851847	0.857089	0.640170	Biso	1.000000	O
O1319	1.0	0.127307	0.252098	0.032361	Biso	1.000000	O
O1320	1.0	0.388523	0.645101	0.534993	Biso	1.000000	O
O1321	1.0	0.335255	0.800148	0.711809	Biso	1.000000	O
O1322	1.0	0.724010	0.648696	0.564166	Biso	1.000000	O
O1323	1.0	0.868359	0.427007	0.192749	Biso	1.000000	O
O1324	1.0	0.990523	0.499095	0.917172	Biso	1.000000	O
O1325	1.0	0.632857	0.150312	0.861621	Biso	1.000000	O
O1326	1.0	0.799149	0.769570	0.708855	Biso	1.000000	O
O1327	1.0	0.268795	0.142083	0.745633	Biso	1.000000	O
O1328	1.0	0.888956	0.144741	0.159807	Biso	1.000000	O
O1329	1.0	0.493310	0.378458	0.563878	Biso	1.000000	O
O1330	1.0	0.197286	0.694890	0.274065	Biso	1.000000	O
O1331	1.0	0.182452	0.623802	0.051668	Biso	1.000000	O
O1332	1.0	0.005638	0.677595	0.159833	Biso	1.000000	O
O1333	1.0	0.375471	0.898686	0.718020	Biso	1.000000	O
O1334	1.0	0.134103	0.257550	0.297852	Biso	1.000000	O
O1335	1.0	0.341628	0.108696	0.805927	Biso	1.000000	O
O1336	1.0	0.955543	0.087710	0.014701	Biso	1.000000	O
O1337	1.0	0.178405	0.255027	0.520248	Biso	1.000000	O
O1338	1.0	0.073675	0.892070	0.212201	Biso	1.000000	O
O1339	1.0	0.514212	0.531164	0.646666	Biso	1.000000	O
O1340	1.0	0.913327	0.684349	0.516944	Biso	1.000000	O
O1341	1.0	0.753123	0.479913	0.186718	Biso	1.000000	O
O1342	1.0	0.305790	0.440924	0.770123	Biso	1.000000	O
O1343	1.0	0.703108	0.936972	0.286340	Biso	1.000000	O
O1344	1.0	0.470474	0.003936	0.036441	Biso	1.000000	O
O1345	1.0	0.043499	0.278879	0.438099	Biso	1.000000	O
O1346	1.0	0.114991	0.690725	0.134955	Biso	1.000000	O
O1347	1.0	0.463433	0.138724	0.888930	Biso	1.000000	O
O1348	1.0	0.541929	0.963155	0.940243	Biso	1.000000	O
O1349	1.0	0.684889	0.217917	0.118597	Biso	1.000000	O
O1350	1.0	0.662354	0.892451	0.314540	Biso	1.000000	O
O1351	1.0	0.137174	0.994088	0.092373	Biso	1.000000	O
O1352	1.0	0.227903	0.778922	0.814664	Biso	1.000000	O
O1353	1.0	0.142800	0.674630	0.968772	Biso	1.000000	O
O1354	1.0	0.878363	0.161652	0.569789	Biso	1.000000	O
O1355	1.0	0.280298	0.550387	0.861421	Biso	1.000000	O
O1356	1.0	0.010910	0.520939	0.261252	Biso	1.000000	O
O1357	1.0	0.768495	0.827542	0.724068	Biso	1.000000	O
O1358	1.0	0.336385	0.128004	0.495750	Biso	1.000000	O
O1359	1.0	0.934363	0.898300	0.264454	Biso	1.000000	O
O1360	1.0	0.801860	0.701150	0.610259	Biso	1.000000	O
O1361	1.0	0.276226	0.553701	0.305675	Biso	1.000000	O
O1362	1.0	0.716986	0.277163	0.900675	Biso	1.000000	O
O1363	1.0	0.195244	0.689682	0.617329	Biso	1.000000	O
O1364	1.0	0.115297	0.376477	0.775444	Biso	1.000000	O

O1365	1.0	0.105220	0.524245	0.468857	Biso	1.000000	O
O1366	1.0	0.452322	0.118848	0.237528	Biso	1.000000	O
O1367	1.0	0.127404	0.974578	0.409875	Biso	1.000000	O
O1368	1.0	0.693183	0.257132	0.467427	Biso	1.000000	O
O1369	1.0	0.972425	0.993417	0.565437	Biso	1.000000	O
O1370	1.0	0.908819	0.970747	0.400115	Biso	1.000000	O
O1371	1.0	0.704181	0.190961	0.514639	Biso	1.000000	O
O1372	1.0	0.928465	0.272051	0.189266	Biso	1.000000	O
O1373	1.0	0.062336	0.481096	0.770260	Biso	1.000000	O
O1374	1.0	0.386274	0.537002	0.640352	Biso	1.000000	O
O1375	1.0	0.581826	0.006909	0.499355	Biso	1.000000	O
O1376	1.0	0.291522	0.902287	0.735196	Biso	1.000000	O
O1377	1.0	0.687960	0.470520	0.764187	Biso	1.000000	O
O1378	1.0	0.676967	0.990168	0.681193	Biso	1.000000	O
O1379	1.0	0.632127	0.360443	0.108043	Biso	1.000000	O
O1380	1.0	0.818207	0.857316	0.984407	Biso	1.000000	O
O1381	1.0	0.761910	0.200653	0.542469	Biso	1.000000	O
O1382	1.0	0.372526	0.955434	0.681978	Biso	1.000000	O
O1383	1.0	0.719053	0.701066	0.771119	Biso	1.000000	O
O1384	1.0	0.933846	0.892370	0.696120	Biso	1.000000	O
O1385	1.0	0.030746	0.437088	0.618969	Biso	1.000000	O
O1386	1.0	0.686766	0.955075	0.626425	Biso	1.000000	O
O1387	1.0	0.003728	0.768552	0.687292	Biso	1.000000	O
O1388	1.0	0.918379	0.887215	0.867398	Biso	1.000000	O
O1389	1.0	0.839326	0.103949	0.147602	Biso	1.000000	O
O1390	1.0	0.063479	0.336077	0.409901	Biso	1.000000	O
O1391	1.0	0.366287	0.140690	0.442672	Biso	1.000000	O
O1392	1.0	0.670079	0.299675	0.424344	Biso	1.000000	O
O1393	1.0	0.470780	0.150741	0.828140	Biso	1.000000	O
O1394	1.0	0.617149	0.188527	0.403671	Biso	1.000000	O
O1395	1.0	0.072320	0.247167	0.236537	Biso	1.000000	O
O1396	1.0	0.791883	0.947681	0.324880	Biso	1.000000	O
O1397	1.0	0.589053	0.598563	0.601734	Biso	1.000000	O
O1398	1.0	0.048414	0.321284	0.694749	Biso	1.000000	O
O1399	1.0	0.979227	0.365171	0.191216	Biso	1.000000	O
O1400	1.0	0.625364	0.995802	0.455723	Biso	1.000000	O
O1401	1.0	0.085282	0.964720	0.995090	Biso	1.000000	O
O1402	1.0	0.038238	0.201434	0.102820	Biso	1.000000	O
O1403	1.0	0.941358	0.836502	0.292833	Biso	1.000000	O
O1404	1.0	0.176533	0.115436	0.331536	Biso	1.000000	O
O1405	1.0	0.740145	0.246091	0.158534	Biso	1.000000	O
O1406	1.0	0.537408	0.052919	0.515573	Biso	1.000000	O
O1407	1.0	0.714670	0.171189	0.793947	Biso	1.000000	O
O1408	1.0	0.729498	0.526032	0.286550	Biso	1.000000	O
O1409	1.0	0.839977	0.365162	0.697545	Biso	1.000000	O
O1410	1.0	0.810475	0.314300	0.487241	Biso	1.000000	O
O1411	1.0	0.598760	0.066344	0.525536	Biso	1.000000	O
O1412	1.0	0.238024	0.685109	0.427784	Biso	1.000000	O
O1413	1.0	0.284118	0.516188	0.030303	Biso	1.000000	O
O1414	1.0	0.150507	0.402865	0.252807	Biso	1.000000	O
O1415	1.0	0.923407	0.097929	0.499707	Biso	1.000000	O
O1416	1.0	0.750202	0.063624	0.804136	Biso	1.000000	O
O1417	1.0	0.227041	0.547876	0.268265	Biso	1.000000	O
O1418	1.0	0.090319	0.306032	0.311942	Biso	1.000000	O
O1419	1.0	0.934231	0.513717	0.570088	Biso	1.000000	O
O1420	1.0	0.093902	0.830790	0.231892	Biso	1.000000	O
O1421	1.0	0.931641	0.769787	0.733103	Biso	1.000000	O
O1422	1.0	0.787244	0.063689	0.145667	Biso	1.000000	O
O1423	1.0	0.242386	0.815368	0.475230	Biso	1.000000	O
O1424	1.0	0.194701	0.091518	0.423431	Biso	1.000000	O
O1425	1.0	0.077736	0.848651	0.644123	Biso	1.000000	O
O1426	1.0	0.922538	0.361462	0.424037	Biso	1.000000	O
O1427	1.0	0.716490	0.953105	0.229569	Biso	1.000000	O
O1428	1.0	0.100692	0.726506	0.542971	Biso	1.000000	O
O1429	1.0	0.657126	0.807202	0.998443	Biso	1.000000	O
O1430	1.0	0.648605	0.649017	0.967870	Biso	1.000000	O
O1431	1.0	0.763306	0.684255	0.655869	Biso	1.000000	O
O1432	1.0	0.740169	0.087163	0.577288	Biso	1.000000	O
O1433	1.0	0.631784	0.138441	0.921136	Biso	1.000000	O
O1434	1.0	0.201630	0.742350	0.577130	Biso	1.000000	O
O1435	1.0	0.371310	0.730174	0.459635	Biso	1.000000	O
O1436	1.0	0.565413	0.990491	0.782528	Biso	1.000000	O
O1437	1.0	0.603471	0.844791	0.246898	Biso	1.000000	O
O1438	1.0	0.104089	0.910452	0.417043	Biso	1.000000	O
O1439	1.0	0.004509	0.190830	0.574296	Biso	1.000000	O
O1440	1.0	0.807273	0.927034	0.992921	Biso	1.000000	O
O1441	1.0	0.159217	0.505949	0.440142	Biso	1.000000	O
O1442	1.0	0.927089	0.254315	0.508301	Biso	1.000000	O
O1443	1.0	0.829658	0.223986	0.949934	Biso	1.000000	O
O1444	1.0	0.075154	0.748718	0.397236	Biso	1.000000	O

O1445	1.0	0.959535	0.209997	0.190905	Biso	1.000000	O
O1446	1.0	0.893158	0.564877	0.553554	Biso	1.000000	O
O1447	1.0	0.984818	0.235650	0.488447	Biso	1.000000	O
O1448	1.0	0.717686	0.642530	0.680301	Biso	1.000000	O
O1449	1.0	0.153264	0.467179	0.608178	Biso	1.000000	O
O1450	1.0	0.849746	0.407751	0.516640	Biso	1.000000	O
O1451	1.0	0.362247	0.785442	0.995837	Biso	1.000000	O
O1452	1.0	0.977793	0.482788	0.373432	Biso	1.000000	O
O1453	1.0	0.209040	0.403135	0.086257	Biso	1.000000	O
O1454	1.0	0.958118	0.671529	0.687783	Biso	1.000000	O
O1455	1.0	0.588355	0.492070	0.128529	Biso	1.000000	O
O1456	1.0	0.355950	0.208504	0.790183	Biso	1.000000	O
O1457	1.0	0.901867	0.730011	0.360593	Biso	1.000000	O
O1458	1.0	0.944757	0.318392	0.703173	Biso	1.000000	O
O1459	1.0	0.723312	0.026160	0.045814	Biso	1.000000	O
O1460	1.0	0.643462	0.674349	0.167107	Biso	1.000000	O
O1461	1.0	0.825924	0.961157	0.274374	Biso	1.000000	O
O1462	1.0	0.602314	0.925147	0.989242	Biso	1.000000	O
O1463	1.0	0.914630	0.452000	0.596627	Biso	1.000000	O
O1464	1.0	0.263759	0.098410	0.096203	Biso	1.000000	O
O1465	1.0	0.670207	0.929264	0.193417	Biso	1.000000	O
O1466	1.0	0.732255	0.050379	0.628365	Biso	1.000000	O
O1467	1.0	0.922943	0.665407	0.351032	Biso	1.000000	O
O1468	1.0	0.836820	0.346599	0.593303	Biso	1.000000	O
O1469	1.0	0.205947	0.942216	0.990828	Biso	1.000000	O
O1470	1.0	0.754790	0.196605	0.647919	Biso	1.000000	O
O1471	1.0	0.177062	0.111061	0.717439	Biso	1.000000	O
O1472	1.0	0.564118	0.098156	0.629731	Biso	1.000000	O
O1473	1.0	0.373113	0.743882	0.807968	Biso	1.000000	O
O1474	1.0	0.574484	0.230367	0.375223	Biso	1.000000	O
O1475	1.0	0.947668	0.356070	0.482184	Biso	1.000000	O
O1476	1.0	0.755310	0.649769	0.344970	Biso	1.000000	O
O1477	1.0	0.474783	0.254150	0.699626	Biso	1.000000	O
O1478	1.0	0.094809	0.314854	0.521140	Biso	1.000000	O
O1479	1.0	0.558685	0.240713	0.724858	Biso	1.000000	O
O1480	1.0	0.386959	0.110631	0.710330	Biso	1.000000	O
O1481	1.0	0.512370	0.178670	0.906260	Biso	1.000000	O
O1482	1.0	0.417892	0.875666	0.580334	Biso	1.000000	O
O1483	1.0	0.065059	0.412341	0.311187	Biso	1.000000	O
O1484	1.0	0.071250	0.958390	0.385330	Biso	1.000000	O
O1485	1.0	0.586246	0.035832	0.615082	Biso	1.000000	O
O1486	1.0	0.255146	0.669405	0.371006	Biso	1.000000	O
O1487	1.0	0.485316	0.305902	0.066273	Biso	1.000000	O
O1488	1.0	0.750582	0.873904	0.051288	Biso	1.000000	O
O1489	1.0	0.396102	0.774995	0.502187	Biso	1.000000	O
O1490	1.0	0.091990	0.898378	0.603204	Biso	1.000000	O
O1491	1.0	0.285850	0.683311	0.677309	Biso	1.000000	O
O1492	1.0	0.357003	0.906808	0.503886	Biso	1.000000	O
O1493	1.0	0.856600	0.294085	0.378982	Biso	1.000000	O
O1494	1.0	0.759483	0.794172	0.570591	Biso	1.000000	O
O1495	1.0	0.884716	0.005318	0.206502	Biso	1.000000	O
O1496	1.0	0.721438	0.605720	0.056258	Biso	1.000000	O
O1497	1.0	0.770754	0.740961	0.274343	Biso	1.000000	O
O1498	1.0	0.684588	0.624029	0.183963	Biso	1.000000	O
O1499	1.0	0.031240	0.985449	0.265393	Biso	1.000000	O
O1500	1.0	0.024664	0.268436	0.660432	Biso	1.000000	O
O1501	1.0	0.852967	0.374379	0.755692	Biso	1.000000	O
O1502	1.0	0.216025	0.169125	0.332141	Biso	1.000000	O
O1503	1.0	0.558860	0.744534	0.336855	Biso	1.000000	O
O1504	1.0	0.622348	0.824747	0.909221	Biso	1.000000	O
O1505	1.0	0.760559	0.712837	0.180976	Biso	1.000000	O
O1506	1.0	0.727594	0.718959	0.939848	Biso	1.000000	O
O1507	1.0	0.199125	0.459363	0.720004	Biso	1.000000	O
O1508	1.0	0.145815	0.592244	0.756983	Biso	1.000000	O
O1509	1.0	0.112074	0.835566	0.694952	Biso	1.000000	O
O1510	1.0	0.581538	0.695917	0.885801	Biso	1.000000	O
O1511	1.0	0.007324	0.897205	0.974843	Biso	1.000000	O
O1512	1.0	0.652154	0.157363	0.495990	Biso	1.000000	O
O1513	1.0	0.218678	0.875707	0.003769	Biso	1.000000	O
O1514	1.0	0.751063	0.895783	0.490419	Biso	1.000000	O
O1515	1.0	0.424470	0.634487	0.715611	Biso	1.000000	O

O1516	1.0	0.713945	0.316470	0.788892	Biso	1.000000	O
O1517	1.0	0.032335	0.919455	0.171990	Biso	1.000000	O
O1518	1.0	0.294886	0.705840	0.505306	Biso	1.000000	O
O1519	1.0	0.032717	0.407743	0.459458	Biso	1.000000	O
O1520	1.0	0.330567	0.664272	0.719103	Biso	1.000000	O
O1521	1.0	0.812302	0.165074	0.648349	Biso	1.000000	O
O1522	1.0	0.034099	0.896616	0.627824	Biso	1.000000	O
O1523	1.0	0.159371	0.396145	0.817194	Biso	1.000000	O
O1524	1.0	0.686105	0.777525	0.536246	Biso	1.000000	O
O1525	1.0	0.290099	0.719876	0.346287	Biso	1.000000	O
O1526	1.0	0.624050	0.332915	0.874460	Biso	1.000000	O
O1527	1.0	0.359884	0.700939	0.762282	Biso	1.000000	O
O1528	1.0	0.028485	0.641255	0.112767	Biso	1.000000	O
O1529	1.0	0.648898	0.009268	0.899248	Biso	1.000000	O
O1530	1.0	0.547712	0.222947	0.586688	Biso	1.000000	O
O1531	1.0	0.176072	0.347026	0.773113	Biso	1.000000	O
O1532	1.0	0.035233	0.139897	0.938456	Biso	1.000000	O
O1533	1.0	0.702506	0.454330	0.157328	Biso	1.000000	O
O1534	1.0	0.311638	0.503576	0.204063	Biso	1.000000	O
O1535	1.0	0.918954	0.201738	0.748554	Biso	1.000000	O
O1536	1.0	0.069999	0.383391	0.609412	Biso	1.000000	O
O1537	1.0	0.165205	0.977784	0.683052	Biso	1.000000	O
O1538	1.0	0.551125	0.507347	0.194350	Biso	1.000000	O
O1539	1.0	0.616572	0.390980	0.907077	Biso	1.000000	O
O1540	1.0	0.251045	0.575983	0.910136	Biso	1.000000	O
O1541	1.0	0.176097	0.597406	0.437523	Biso	1.000000	O
O1542	1.0	0.823825	0.705780	0.667242	Biso	1.000000	O
O1543	1.0	0.297382	0.211471	0.982007	Biso	1.000000	O
O1544	1.0	0.953106	0.155916	0.280346	Biso	1.000000	O
O1545	1.0	0.009014	0.640668	0.531180	Biso	1.000000	O
O1546	1.0	0.671226	0.678062	0.585200	Biso	1.000000	O
O1547	1.0	0.246460	0.796675	0.417411	Biso	1.000000	O
O1548	1.0	0.792202	0.474164	0.452844	Biso	1.000000	O
O1549	1.0	0.840080	0.602961	0.548981	Biso	1.000000	O
O1550	1.0	0.968402	0.498914	0.733974	Biso	1.000000	O
O1551	1.0	0.887209	0.236659	0.835243	Biso	1.000000	O
O1552	1.0	0.839799	0.196047	0.814645	Biso	1.000000	O
O1553	1.0	0.894132	0.877955	0.458274	Biso	1.000000	O
O1554	1.0	0.994852	0.842210	0.939562	Biso	1.000000	O
O1555	1.0	0.039880	0.821963	0.775614	Biso	1.000000	O
O1556	1.0	0.834215	0.319238	0.431916	Biso	1.000000	O
O1557	1.0	0.042110	0.886715	0.924389	Biso	1.000000	O
O1558	1.0	0.062286	0.797794	0.014255	Biso	1.000000	O
O1559	1.0	0.951433	0.379896	0.675652	Biso	1.000000	O
O1560	1.0	0.241453	0.596217	0.761343	Biso	1.000000	O
O1561	1.0	0.650972	0.211900	0.754849	Biso	1.000000	O
O1562	1.0	0.290134	0.881345	0.051538	Biso	1.000000	O
O1563	1.0	0.215540	0.555120	0.538212	Biso	1.000000	O
O1564	1.0	0.149802	0.703294	0.575952	Biso	1.000000	O
O1565	1.0	0.941408	0.701531	0.740347	Biso	1.000000	O
O1566	1.0	0.158901	0.411860	0.418343	Biso	1.000000	O
O1567	1.0	0.322781	0.687447	0.017166	Biso	1.000000	O
O1568	1.0	0.095472	0.706723	0.191170	Biso	1.000000	O
O1569	1.0	0.766350	0.311935	0.724743	Biso	1.000000	O
O1570	1.0	0.321834	0.533656	0.630916	Biso	1.000000	O
O1571	1.0	0.261347	0.329947	0.418332	Biso	1.000000	O
O1572	1.0	0.170619	0.620751	0.941588	Biso	1.000000	O
O1573	1.0	0.132318	0.786672	0.534403	Biso	1.000000	O
O1574	1.0	0.854861	0.235688	0.288499	Biso	1.000000	O
O1575	1.0	0.789486	0.777244	0.804416	Biso	1.000000	O
O1576	1.0	0.411351	0.030952	0.447580	Biso	1.000000	O
O1577	1.0	0.650760	0.226117	0.507760	Biso	1.000000	O
O1578	1.0	0.361618	0.427907	0.658899	Biso	1.000000	O
O1579	1.0	0.652062	0.453776	0.716123	Biso	1.000000	O
O1580	1.0	0.879080	0.216213	0.530061	Biso	1.000000	O
O1581	1.0	0.996995	0.667989	0.734726	Biso	1.000000	O
O1582	1.0	0.302465	0.473096	0.258288	Biso	1.000000	O
O1583	1.0	0.769188	0.310813	0.088242	Biso	1.000000	O
O1584	1.0	0.120667	0.887686	0.254515	Biso	1.000000	O
O1585	1.0	0.467576	0.473164	0.708997	Biso	1.000000	O
O1586	1.0	0.975613	0.194176	0.713301	Biso	1.000000	O
O1587	1.0	0.477072	0.682668	0.583380	Biso	1.000000	O
O1588	1.0	0.570762	0.108443	0.052240	Biso	1.000000	O
O1589	1.0	0.429913	0.078515	0.879968	Biso	1.000000	O
O1590	1.0	0.895962	0.998862	0.663344	Biso	1.000000	O
O1591	1.0	0.791629	0.589049	0.636473	Biso	1.000000	O
O1592	1.0	0.703012	0.711156	0.541098	Biso	1.000000	O
O1593	1.0	0.186401	0.262854	0.460128	Biso	1.000000	O
O1594	1.0	0.115068	0.581932	0.344161	Biso	1.000000	O
O1595	1.0	0.589425	0.057660	0.673009	Biso	1.000000	O
O1596	1.0	0.913055	0.269773	0.737690	Biso	1.000000	O
O1597	1.0	0.579224	0.646514	0.483855	Biso	1.000000	O
O1598	1.0	0.619975	0.525232	0.056378	Biso	1.000000	O
O1599	1.0	0.069691	0.436902	0.571825	Biso	1.000000	O
H1	1.0	0.489507	0.117589	0.591488	Biso	1.000000	H

H2	1.0	0.737437	0.081317	0.819027	Biso	1.000000	H
H3	1.0	0.818374	0.100481	0.769571	Biso	1.000000	H
H4	1.0	0.847521	0.332347	0.908438	Biso	1.000000	H
H5	1.0	0.447937	0.699420	0.823853	Biso	1.000000	H
H6	1.0	0.503829	0.736139	0.755095	Biso	1.000000	H
H7	1.0	0.715509	0.246211	0.476325	Biso	1.000000	H
H8	1.0	0.735333	0.279822	0.413711	Biso	1.000000	H
H9	1.0	0.680847	0.321922	0.495960	Biso	1.000000	H
H10	1.0	0.592994	0.919447	0.076046	Biso	1.000000	H
H11	1.0	0.678866	0.911201	0.073339	Biso	1.000000	H
H12	1.0	0.581141	0.078194	0.688394	Biso	1.000000	H
H13	1.0	0.715326	0.317614	0.657327	Biso	1.000000	H
H14	1.0	0.505116	0.804164	0.457934	Biso	1.000000	H
H15	1.0	0.477429	0.889897	0.522657	Biso	1.000000	H
H16	1.0	0.553878	0.964401	0.863644	Biso	1.000000	H
H17	1.0	0.320169	0.112658	0.509783	Biso	1.000000	H
H18	1.0	0.369138	0.167065	0.451419	Biso	1.000000	H
H19	1.0	0.552928	0.786931	0.893890	Biso	1.000000	H
H20	1.0	0.175708	0.251029	0.375122	Biso	1.000000	H
H21	1.0	0.137396	0.207396	0.415585	Biso	1.000000	H
H22	1.0	0.430374	0.223421	0.493817	Biso	1.000000	H
H23	1.0	0.347239	0.214169	0.446055	Biso	1.000000	H
H24	1.0	0.359714	0.303175	0.476219	Biso	1.000000	H
H25	1.0	0.411916	0.244691	0.423910	Biso	1.000000	H
H26	1.0	0.654867	0.073844	0.161383	Biso	1.000000	H
H27	1.0	0.665948	0.788264	0.664583	Biso	1.000000	H
H28	1.0	0.957887	0.527569	0.121996	Biso	1.000000	H
H29	1.0	0.783367	0.475493	0.476264	Biso	1.000000	H
H30	1.0	0.371838	0.893440	0.635123	Biso	1.000000	H
H31	1.0	0.744833	0.436572	0.771218	Biso	1.000000	H
H32	1.0	0.858504	0.523258	0.048482	Biso	1.000000	H
H33	1.0	0.909485	0.467269	0.066045	Biso	1.000000	H
H34	1.0	0.772819	0.413284	0.595655	Biso	1.000000	H
H35	1.0	0.840559	0.326857	0.610932	Biso	1.000000	H
H36	1.0	0.237018	0.935294	0.540711	Biso	1.000000	H
H37	1.0	0.238622	0.560280	0.093070	Biso	1.000000	H
H38	1.0	0.766068	0.474626	0.427657	Biso	1.000000	H
H39	1.0	0.691233	0.403443	0.392722	Biso	1.000000	H
H40	1.0	0.755212	0.384564	0.437018	Biso	1.000000	H
H41	1.0	0.677414	0.220928	0.600934	Biso	1.000000	H
H42	1.0	0.594696	0.109602	0.513969	Biso	1.000000	H
H43	1.0	0.796049	0.919807	0.551114	Biso	1.000000	H
H44	1.0	0.761819	0.889489	0.468725	Biso	1.000000	H
H45	1.0	0.748017	0.832234	0.552816	Biso	1.000000	H
H46	1.0	0.666606	0.502990	0.292371	Biso	1.000000	H
H47	1.0	0.750151	0.514266	0.344566	Biso	1.000000	H
H48	1.0	0.681895	0.589884	0.308643	Biso	1.000000	H
H49	1.0	0.946610	0.873025	0.086566	Biso	1.000000	H
H50	1.0	0.956074	0.808486	0.018439	Biso	1.000000	H
H51	1.0	0.879754	0.817638	0.050683	Biso	1.000000	H
H52	1.0	0.899936	0.881942	0.998629	Biso	1.000000	H
H53	1.0	0.283985	0.820560	0.789798	Biso	1.000000	H
H54	1.0	0.209182	0.796748	0.807401	Biso	1.000000	H
H55	1.0	0.289288	0.645250	0.832548	Biso	1.000000	H
H56	1.0	0.496731	0.900051	0.960774	Biso	1.000000	H
H57	1.0	0.013470	0.203972	0.959771	Biso	1.000000	H
H58	1.0	0.077527	0.171205	0.993683	Biso	1.000000	H
H59	1.0	0.613753	0.183218	0.101635	Biso	1.000000	H
H60	1.0	0.709602	0.218679	0.111853	Biso	1.000000	H
H61	1.0	0.019290	0.187217	0.877377	Biso	1.000000	H
H62	1.0	0.846954	0.751588	0.587852	Biso	1.000000	H
H63	1.0	0.026020	0.332358	0.135039	Biso	1.000000	H
H64	1.0	0.103008	0.445854	0.000974	Biso	1.000000	H
H65	1.0	0.030014	0.422793	0.928724	Biso	1.000000	H
H66	1.0	0.081506	0.456995	0.912011	Biso	1.000000	H
H67	1.0	0.912152	0.532924	0.209631	Biso	1.000000	H
H68	1.0	0.883118	0.574153	0.295863	Biso	1.000000	H
H69	1.0	0.919196	0.596433	0.226503	Biso	1.000000	H
H70	1.0	0.478445	0.198614	0.648931	Biso	1.000000	H
H71	1.0	0.824836	0.406905	0.523060	Biso	1.000000	H
H72	1.0	0.744116	0.781344	0.587411	Biso	1.000000	H

H73	1.0	0.666557	0.904989	0.890950	Biso	1.000000	H
H74	1.0	0.963679	0.752069	0.140327	Biso	1.000000	H
H75	1.0	0.961196	0.667551	0.212155	Biso	1.000000	H
H76	1.0	0.018100	0.824233	0.762721	Biso	1.000000	H
H77	1.0	0.118401	0.793810	0.755904	Biso	1.000000	H
H78	1.0	0.702030	0.849562	0.350617	Biso	1.000000	H
H79	1.0	0.699936	0.820309	0.273937	Biso	1.000000	H
H80	1.0	0.621773	0.837302	0.280016	Biso	1.000000	H
H81	1.0	0.551685	0.590065	0.102142	Biso	1.000000	H
H82	1.0	0.585676	0.543746	0.046751	Biso	1.000000	H
H83	1.0	0.499626	0.541392	0.025554	Biso	1.000000	H
H84	1.0	0.010723	0.675557	0.368838	Biso	1.000000	H
H85	1.0	0.605417	0.238245	0.666506	Biso	1.000000	H
H86	1.0	0.571744	0.265225	0.724889	Biso	1.000000	H
H87	1.0	0.644145	0.566147	0.789123	Biso	1.000000	H
H88	1.0	0.565674	0.543108	0.743127	Biso	1.000000	H
H89	1.0	0.107258	0.793026	0.223069	Biso	1.000000	H
H90	1.0	0.311172	0.534724	0.557858	Biso	1.000000	H
H91	1.0	0.278545	0.190971	0.472599	Biso	1.000000	H
H92	1.0	0.275146	0.199215	0.379409	Biso	1.000000	H
H93	1.0	0.262319	0.263241	0.465558	Biso	1.000000	H
H94	1.0	0.327494	0.243044	0.405328	Biso	1.000000	H
H95	1.0	0.226909	0.227073	0.379775	Biso	1.000000	H
H96	1.0	0.267034	0.205344	0.309776	Biso	1.000000	H
H97	1.0	0.034965	0.040978	0.617062	Biso	1.000000	H
H98	1.0	0.953799	0.114767	0.620373	Biso	1.000000	H
H99	1.0	0.629688	0.029524	0.299853	Biso	1.000000	H
H100	1.0	0.587995	0.951757	0.245114	Biso	1.000000	H
H101	1.0	0.990915	0.978621	0.425024	Biso	1.000000	H
H102	1.0	0.908425	0.007999	0.457410	Biso	1.000000	H
H103	1.0	0.717551	0.261109	0.676329	Biso	1.000000	H
H104	1.0	0.595855	0.478834	0.561221	Biso	1.000000	H
H105	1.0	0.530507	0.286163	0.956805	Biso	1.000000	H
H106	1.0	0.446176	0.218797	0.990373	Biso	1.000000	H
H107	1.0	0.515300	0.261136	0.020012	Biso	1.000000	H
H108	1.0	0.076187	0.749521	0.597227	Biso	1.000000	H
H109	1.0	0.772335	0.752591	0.296875	Biso	1.000000	H
H110	1.0	0.840704	0.761792	0.362922	Biso	1.000000	H
H111	1.0	0.672054	0.199756	0.343401	Biso	1.000000	H
H112	1.0	0.059183	0.687312	0.788541	Biso	1.000000	H
H113	1.0	0.131670	0.740128	0.836151	Biso	1.000000	H
H114	1.0	0.628852	0.535100	0.825030	Biso	1.000000	H
H115	1.0	0.851213	0.331517	0.811342	Biso	1.000000	H
H116	1.0	0.582403	0.084371	0.804839	Biso	1.000000	H
H117	1.0	0.855164	0.871823	0.045978	Biso	1.000000	H
H118	1.0	0.836045	0.836915	0.982457	Biso	1.000000	H
H119	1.0	0.877561	0.928369	0.995819	Biso	1.000000	H
H120	1.0	0.011355	0.540408	0.243071	Biso	1.000000	H
H121	1.0	0.054542	0.067095	0.653599	Biso	1.000000	H
H122	1.0	0.135089	0.000923	0.669798	Biso	1.000000	H
H123	1.0	0.131324	0.100515	0.637810	Biso	1.000000	H
H124	1.0	0.828713	0.957959	0.928971	Biso	1.000000	H
H125	1.0	0.153937	0.633470	0.635340	Biso	1.000000	H
H126	1.0	0.478926	0.438910	0.011479	Biso	1.000000	H
H127	1.0	0.634929	0.013137	0.878351	Biso	1.000000	H
H128	1.0	0.611501	0.072555	0.923293	Biso	1.000000	H
H129	1.0	0.633659	0.703121	0.845203	Biso	1.000000	H
H130	1.0	0.580929	0.667691	0.883198	Biso	1.000000	H
H131	1.0	0.640907	0.374144	0.677087	Biso	1.000000	H
H132	1.0	0.738813	0.339228	0.865314	Biso	1.000000	H
H133	1.0	0.891200	0.402848	0.369817	Biso	1.000000	H
H134	1.0	0.979539	0.392018	0.393855	Biso	1.000000	H
H135	1.0	0.950590	0.308273	0.388871	Biso	1.000000	H
H136	1.0	0.679251	0.589025	0.432610	Biso	1.000000	H
H137	1.0	0.760285	0.556539	0.458283	Biso	1.000000	H
H138	1.0	0.685543	0.531481	0.492680	Biso	1.000000	H
H139	1.0	0.343628	0.714473	0.383890	Biso	1.000000	H
H140	1.0	0.301259	0.808435	0.378236	Biso	1.000000	H
H141	1.0	0.342662	0.755021	0.310387	Biso	1.000000	H
H142	1.0	0.687619	0.011442	0.693168	Biso	1.000000	H
H143	1.0	0.702770	0.940040	0.612286	Biso	1.000000	H
H144	1.0	0.007405	0.893100	0.999398	Biso	1.000000	H
H145	1.0	0.970718	0.852194	0.937595	Biso	1.000000	H
H146	1.0	0.064866	0.874615	0.918548	Biso	1.000000	H
H147	1.0	0.917418	0.645495	0.629832	Biso	1.000000	H
H148	1.0	0.086565	0.909682	0.580943	Biso	1.000000	H
H149	1.0	0.025872	0.844175	0.586342	Biso	1.000000	H
H150	1.0	0.449486	0.020688	0.757716	Biso	1.000000	H
H151	1.0	0.479190	0.939413	0.707961	Biso	1.000000	H
H152	1.0	0.641514	0.047704	0.022602	Biso	1.000000	H
H153	1.0	0.586512	0.516434	0.141456	Biso	1.000000	H
H154	1.0	0.091920	0.227493	0.975934	Biso	1.000000	H
H155	1.0	0.149815	0.264710	0.037965	Biso	1.000000	H
H156	1.0	0.178156	0.192752	0.000055	Biso	1.000000	H
H157	1.0	0.107697	0.224514	0.162784	Biso	1.000000	H

H158	1.0	0.571581	0.654377	0.354338	Biso	1.000000	H
H159	1.0	0.582810	0.751088	0.329307	Biso	1.000000	H
H160	1.0	0.524326	0.704745	0.383732	Biso	1.000000	H
H161	1.0	0.508073	0.712489	0.295615	Biso	1.000000	H
H162	1.0	0.203411	0.727098	0.212083	Biso	1.000000	H
H163	1.0	0.143678	0.661083	0.246900	Biso	1.000000	H
H164	1.0	0.222677	0.626986	0.220972	Biso	1.000000	H
H165	1.0	0.883695	0.193524	0.472045	Biso	1.000000	H
H166	1.0	0.104700	0.735309	0.131616	Biso	1.000000	H
H167	1.0	0.174994	0.798478	0.115267	Biso	1.000000	H
H168	1.0	0.124433	0.792048	0.182782	Biso	1.000000	H
H169	1.0	0.886465	0.710702	0.227481	Biso	1.000000	H
H170	1.0	0.907877	0.675122	0.288735	Biso	1.000000	H
H171	1.0	0.152409	0.735377	0.065110	Biso	1.000000	H
H172	1.0	0.601648	0.817030	0.241225	Biso	1.000000	H
H173	1.0	0.557360	0.907498	0.251060	Biso	1.000000	H
H174	1.0	0.543339	0.845426	0.210531	Biso	1.000000	H
H175	1.0	0.578013	0.472023	0.003614	Biso	1.000000	H
H176	1.0	0.843306	0.241143	0.964667	Biso	1.000000	H
H177	1.0	0.764396	0.213761	0.899727	Biso	1.000000	H
H178	1.0	0.034710	0.950972	0.667891	Biso	1.000000	H
H179	1.0	0.547574	0.108101	0.116150	Biso	1.000000	H
H180	1.0	0.554571	0.176089	0.066434	Biso	1.000000	H
H181	1.0	0.587760	0.984768	0.793954	Biso	1.000000	H
H182	1.0	0.795154	0.188080	0.820880	Biso	1.000000	H
H183	1.0	0.703670	0.196208	0.798656	Biso	1.000000	H
H184	1.0	0.593532	0.623629	0.105146	Biso	1.000000	H
H185	1.0	0.777115	0.486994	0.315944	Biso	1.000000	H
H186	1.0	0.856282	0.526569	0.287064	Biso	1.000000	H
H187	1.0	0.742611	0.711256	0.765685	Biso	1.000000	H
H188	1.0	0.578884	0.338239	0.506668	Biso	1.000000	H
H189	1.0	0.523998	0.291074	0.542181	Biso	1.000000	H
H190	1.0	0.623152	0.253564	0.519705	Biso	1.000000	H
H191	1.0	0.545261	0.243308	0.569564	Biso	1.000000	H
H192	1.0	0.836884	0.201369	0.719478	Biso	1.000000	H
H193	1.0	0.822658	0.063722	0.983869	Biso	1.000000	H
H194	1.0	0.862290	0.034828	0.054096	Biso	1.000000	H
H195	1.0	0.733209	0.438861	0.988843	Biso	1.000000	H
H196	1.0	0.749971	0.350580	0.975492	Biso	1.000000	H
H197	1.0	0.404534	0.006356	0.437032	Biso	1.000000	H
H198	1.0	0.382387	0.988226	0.497279	Biso	1.000000	H
H199	1.0	0.170633	0.954624	0.979182	Biso	1.000000	H
H200	1.0	0.079851	0.991731	0.989093	Biso	1.000000	H
H201	1.0	0.133542	0.185816	0.907541	Biso	1.000000	H
H202	1.0	0.150180	0.255858	0.871324	Biso	1.000000	H
H203	1.0	0.043808	0.130432	0.792583	Biso	1.000000	H
H204	1.0	0.894164	0.863292	0.437084	Biso	1.000000	H
H205	1.0	0.266598	0.638649	0.308520	Biso	1.000000	H
H206	1.0	0.424492	0.726011	0.620330	Biso	1.000000	H
H207	1.0	0.461134	0.737458	0.526954	Biso	1.000000	H
H208	1.0	0.901911	0.461168	0.390089	Biso	1.000000	H
H209	1.0	0.011020	0.483864	0.977043	Biso	1.000000	H
H210	1.0	0.991808	0.568237	0.972202	Biso	1.000000	H
H211	1.0	0.001356	0.474107	0.910464	Biso	1.000000	H
H212	1.0	0.037662	0.552101	0.895100	Biso	1.000000	H
H213	1.0	0.436081	0.339223	0.135158	Biso	1.000000	H
H214	1.0	0.479535	0.398097	0.551867	Biso	1.000000	H
H215	1.0	0.572819	0.373720	0.585115	Biso	1.000000	H
H216	1.0	0.519611	0.403924	0.640989	Biso	1.000000	H
H217	1.0	0.905914	0.805508	0.170768	Biso	1.000000	H
H218	1.0	0.993867	0.791183	0.132392	Biso	1.000000	H
H219	1.0	0.937545	0.885377	0.130715	Biso	1.000000	H
H220	1.0	0.084208	0.655785	0.070624	Biso	1.000000	H
H221	1.0	0.988646	0.666170	0.060650	Biso	1.000000	H
H222	1.0	0.027265	0.572691	0.082903	Biso	1.000000	H
H223	1.0	0.225864	0.198139	0.816403	Biso	1.000000	H
H224	1.0	0.863192	0.429037	0.061713	Biso	1.000000	H
H225	1.0	0.814325	0.363852	0.109928	Biso	1.000000	H
H226	1.0	0.182399	0.619829	0.919700	Biso	1.000000	H
H227	1.0	0.225507	0.678708	0.944625	Biso	1.000000	H
H228	1.0	0.604329	0.771990	0.744479	Biso	1.000000	H
H229	1.0	0.628691	0.681403	0.802564	Biso	1.000000	H
H230	1.0	0.833589	0.682765	0.730373	Biso	1.000000	H
H231	1.0	0.294381	0.429015	0.589765	Biso	1.000000	H
H232	1.0	0.262661	0.464068	0.654571	Biso	1.000000	H
H233	1.0	0.624230	0.147230	0.027940	Biso	1.000000	H
H234	1.0	0.650225	0.087491	0.074849	Biso	1.000000	H
H235	1.0	0.054113	0.575132	0.682512	Biso	1.000000	H
H236	1.0	0.620883	0.015187	0.730668	Biso	1.000000	H
H237	1.0	0.672422	0.068054	0.796051	Biso	1.000000	H
H238	1.0	0.194735	0.964359	0.426350	Biso	1.000000	H
H239	1.0	0.826126	0.967018	0.153813	Biso	1.000000	H
H240	1.0	0.905110	0.972311	0.147032	Biso	1.000000	H

H241	1.0	0.327452	0.615213	0.848122	Biso	1.000000	H
H242	1.0	0.616639	0.046727	0.095576	Biso	1.000000	H
H243	1.0	0.862425	0.283733	0.644717	Biso	1.000000	H
H244	1.0	0.756284	0.299029	0.650557	Biso	1.000000	H
H245	1.0	0.210000	0.140536	0.992968	Biso	1.000000	H
H246	1.0	0.591946	0.324390	0.414559	Biso	1.000000	H
H247	1.0	0.660764	0.263637	0.358842	Biso	1.000000	H
H248	1.0	0.794365	0.601101	0.614325	Biso	1.000000	H
H249	1.0	0.825980	0.561777	0.709808	Biso	1.000000	H
H250	1.0	0.991975	0.865090	0.440619	Biso	1.000000	H
H251	1.0	0.035730	0.852667	0.490986	Biso	1.000000	H
H252	1.0	0.867513	0.524037	0.727213	Biso	1.000000	H
H253	1.0	0.879312	0.557590	0.811767	Biso	1.000000	H
H254	1.0	0.575374	0.525688	0.978207	Biso	1.000000	H
H255	1.0	0.013164	0.321198	0.927602	Biso	1.000000	H
H256	1.0	0.971761	0.351759	0.875333	Biso	1.000000	H
H257	1.0	0.886270	0.135652	0.183186	Biso	1.000000	H
H258	1.0	0.441216	0.104103	0.881301	Biso	1.000000	H
H259	1.0	0.448484	0.988831	0.898864	Biso	1.000000	H
H260	1.0	0.515023	0.098296	0.451575	Biso	1.000000	H
H261	1.0	0.700282	0.481161	0.246617	Biso	1.000000	H
H262	1.0	0.803034	0.512839	0.266240	Biso	1.000000	H
H263	1.0	0.241399	0.396383	0.141902	Biso	1.000000	H
H264	1.0	0.194324	0.405062	0.065871	Biso	1.000000	H
H265	1.0	0.870377	0.028730	0.717067	Biso	1.000000	H
H266	1.0	0.022299	0.293882	0.443592	Biso	1.000000	H
H267	1.0	0.919365	0.628167	0.133037	Biso	1.000000	H
H268	1.0	0.809276	0.054991	0.513657	Biso	1.000000	H
H269	1.0	0.853630	0.995086	0.455001	Biso	1.000000	H
H270	1.0	0.104733	0.487627	0.156690	Biso	1.000000	H
H271	1.0	0.105696	0.448078	0.097233	Biso	1.000000	H
H272	1.0	0.967625	0.404240	0.133843	Biso	1.000000	H
H273	1.0	0.997176	0.350335	0.179022	Biso	1.000000	H
H274	1.0	0.900197	0.046686	0.076293	Biso	1.000000	H
H275	1.0	0.296260	0.804629	0.964841	Biso	1.000000	H
H276	1.0	0.374273	0.761324	0.001385	Biso	1.000000	H
H277	1.0	0.853791	0.651090	0.367679	Biso	1.000000	H
H278	1.0	0.282722	0.237473	0.106390	Biso	1.000000	H
H279	1.0	0.978318	0.528443	0.374695	Biso	1.000000	H
H280	1.0	0.984735	0.577199	0.459468	Biso	1.000000	H
H281	1.0	0.657893	0.857627	0.069336	Biso	1.000000	H
H282	1.0	0.672947	0.797826	0.980472	Biso	1.000000	H
H283	1.0	0.560579	0.369963	0.828749	Biso	1.000000	H
H284	1.0	0.524178	0.301456	0.757742	Biso	1.000000	H
H285	1.0	0.064240	0.703010	0.744750	Biso	1.000000	H
H286	1.0	0.661716	0.680838	0.150159	Biso	1.000000	H
H287	1.0	0.694298	0.607588	0.166082	Biso	1.000000	H
H288	1.0	0.608519	0.643201	0.221505	Biso	1.000000	H
H289	1.0	0.873933	0.630246	0.306581	Biso	1.000000	H
H290	1.0	0.857387	0.591268	0.387139	Biso	1.000000	H
H291	1.0	0.711886	0.384653	0.936578	Biso	1.000000	H
H292	1.0	0.706336	0.481280	0.973224	Biso	1.000000	H
H293	1.0	0.489822	0.295302	0.043524	Biso	1.000000	H
H294	1.0	0.608278	0.158181	0.833801	Biso	1.000000	H
H295	1.0	0.173234	0.331250	0.740047	Biso	1.000000	H
H296	1.0	0.133036	0.311384	0.653797	Biso	1.000000	H
H297	1.0	0.456039	0.282912	0.681077	Biso	1.000000	H
H298	1.0	0.379338	0.255277	0.654418	Biso	1.000000	H
H299	1.0	0.389186	0.357484	0.649882	Biso	1.000000	H
H300	1.0	0.938696	0.199029	0.732867	Biso	1.000000	H
H301	1.0	0.873706	0.240838	0.695407	Biso	1.000000	H
H302	1.0	0.165350	0.845359	0.314259	Biso	1.000000	H
H303	1.0	0.096428	0.909495	0.319802	Biso	1.000000	H
H304	1.0	0.182144	0.926064	0.291849	Biso	1.000000	H
H305	1.0	0.928087	0.147214	0.161759	Biso	1.000000	H
H306	1.0	0.179645	0.300250	0.270589	Biso	1.000000	H
H307	1.0	0.301277	0.060303	0.045590	Biso	1.000000	H
H308	1.0	0.315322	0.075825	0.874319	Biso	1.000000	H
H309	1.0	0.317673	0.101966	0.798510	Biso	1.000000	H
H310	1.0	0.397424	0.074726	0.859075	Biso	1.000000	H
H311	1.0	0.386683	0.154563	0.837893	Biso	1.000000	H
H312	1.0	0.140363	0.486002	0.412253	Biso	1.000000	H
H313	1.0	0.767119	0.821998	0.613251	Biso	1.000000	H
H314	1.0	0.713906	0.738531	0.619547	Biso	1.000000	H
H315	1.0	0.026387	0.720514	0.892665	Biso	1.000000	H
H316	1.0	0.068098	0.828006	0.888982	Biso	1.000000	H
H317	1.0	0.962581	0.446705	0.527954	Biso	1.000000	H
H318	1.0	0.307926	0.182248	0.103199	Biso	1.000000	H
H319	1.0	0.366474	0.184677	0.140573	Biso	1.000000	H

H320	1.0	0.843753	0.181413	0.835557	Biso	1.000000	H
H321	1.0	0.688608	0.684403	0.618629	Biso	1.000000	H
H322	1.0	0.837392	0.168970	0.653861	Biso	1.000000	H
H323	1.0	0.759131	0.924583	0.766558	Biso	1.000000	H
H324	1.0	0.695080	0.896059	0.807894	Biso	1.000000	H
H325	1.0	0.819484	0.573388	0.165932	Biso	1.000000	H
H326	1.0	0.838216	0.146468	0.280917	Biso	1.000000	H
H327	1.0	0.892628	0.207776	0.237176	Biso	1.000000	H
H328	1.0	0.843432	0.242047	0.267005	Biso	1.000000	H
H329	1.0	0.009765	0.435259	0.824860	Biso	1.000000	H
H330	1.0	0.069878	0.490021	0.875100	Biso	1.000000	H
H331	1.0	0.997088	0.422082	0.893558	Biso	1.000000	H
H332	1.0	0.427188	0.131413	0.012957	Biso	1.000000	H
H333	1.0	0.412276	0.069369	0.048712	Biso	1.000000	H
H334	1.0	0.428004	0.177355	0.088863	Biso	1.000000	H
H335	1.0	0.878760	0.242546	0.591535	Biso	1.000000	H
H336	1.0	0.805507	0.170255	0.307945	Biso	1.000000	H
H337	1.0	0.710157	0.143110	0.273590	Biso	1.000000	H
H338	1.0	0.205717	0.868152	0.941833	Biso	1.000000	H
H339	1.0	0.218280	0.963338	0.002368	Biso	1.000000	H
H340	1.0	0.268751	0.941970	0.967130	Biso	1.000000	H
H341	1.0	0.719036	0.429466	0.666329	Biso	1.000000	H
H342	1.0	0.765452	0.396559	0.753837	Biso	1.000000	H
H343	1.0	0.037957	0.815457	0.071727	Biso	1.000000	H
H344	1.0	0.023550	0.739480	0.994919	Biso	1.000000	H
H345	1.0	0.197660	0.840221	0.142304	Biso	1.000000	H
H346	1.0	0.267976	0.846910	0.124022	Biso	1.000000	H
H347	1.0	0.232360	0.910314	0.124605	Biso	1.000000	H
H348	1.0	0.010462	0.233946	0.489829	Biso	1.000000	H
H349	1.0	0.153969	0.391561	0.653861	Biso	1.000000	H
H350	1.0	0.153474	0.483099	0.668158	Biso	1.000000	H
H351	1.0	0.507116	0.259476	0.462889	Biso	1.000000	H
H352	1.0	0.879833	0.847472	0.259442	Biso	1.000000	H
H353	1.0	0.400315	0.086507	0.707137	Biso	1.000000	H
H354	1.0	0.372248	0.106887	0.782863	Biso	1.000000	H
H355	1.0	0.558480	0.390969	0.668238	Biso	1.000000	H
H356	1.0	0.608211	0.728614	0.993040	Biso	1.000000	H
H357	1.0	0.188808	0.357303	0.283954	Biso	1.000000	H
H358	1.0	0.102543	0.337747	0.289178	Biso	1.000000	H
H359	1.0	0.599757	0.639476	0.589546	Biso	1.000000	H
H360	1.0	0.922101	0.869282	0.705113	Biso	1.000000	H
H361	1.0	0.726533	0.528643	0.725056	Biso	1.000000	H
H362	1.0	0.043647	0.120743	0.531739	Biso	1.000000	H
H363	1.0	0.615817	0.341248	0.118091	Biso	1.000000	H
H364	1.0	0.595771	0.335282	0.038766	Biso	1.000000	H
H365	1.0	0.740687	0.933813	0.153018	Biso	1.000000	H
H366	1.0	0.822634	0.960028	0.103356	Biso	1.000000	H
H367	1.0	0.194486	0.278414	0.440781	Biso	1.000000	H
H368	1.0	0.208177	0.333696	0.511340	Biso	1.000000	H
H369	1.0	0.089348	0.111949	0.002985	Biso	1.000000	H
H370	1.0	0.466518	0.142372	0.864396	Biso	1.000000	H
H371	1.0	0.469840	0.185961	0.961996	Biso	1.000000	H
H372	1.0	0.432725	0.210697	0.915495	Biso	1.000000	H
H373	1.0	0.556739	0.195064	0.307357	Biso	1.000000	H
H374	1.0	0.520740	0.284174	0.368755	Biso	1.000000	H
H375	1.0	0.232787	0.619994	0.273373	Biso	1.000000	H
H376	1.0	0.454443	0.899581	0.676952	Biso	1.000000	H
H377	1.0	0.190842	0.759446	0.696427	Biso	1.000000	H
H378	1.0	0.147110	0.434337	0.408263	Biso	1.000000	H
H379	1.0	0.188367	0.419325	0.474069	Biso	1.000000	H
H380	1.0	0.786487	0.889832	0.860286	Biso	1.000000	H
H381	1.0	0.884982	0.863090	0.857249	Biso	1.000000	H
H382	1.0	0.843047	0.823803	0.906673	Biso	1.000000	H
H383	1.0	0.657311	0.284168	0.931985	Biso	1.000000	H
H384	1.0	0.183045	0.282693	0.225388	Biso	1.000000	H
H385	1.0	0.311352	0.390626	0.619280	Biso	1.000000	H
H386	1.0	0.356562	0.454746	0.665914	Biso	1.000000	H
H387	1.0	0.371347	0.450962	0.578727	Biso	1.000000	H
H388	1.0	0.379827	0.367252	0.596356	Biso	1.000000	H
H389	1.0	0.004291	0.050605	0.226345	Biso	1.000000	H
H390	1.0	0.028649	0.491570	0.823454	Biso	1.000000	H
H391	1.0	0.651586	0.200487	0.042168	Biso	1.000000	H
H392	1.0	0.740522	0.869217	0.132659	Biso	1.000000	H
H393	1.0	0.816246	0.871477	0.067167	Biso	1.000000	H
H394	1.0	0.330663	0.788423	0.649410	Biso	1.000000	H
H395	1.0	0.558500	0.561407	0.540838	Biso	1.000000	H
H396	1.0	0.960800	0.335961	0.764578	Biso	1.000000	H
H397	1.0	0.025940	0.755259	0.581318	Biso	1.000000	H
H398	1.0	0.251972	0.977761	0.079802	Biso	1.000000	H
H399	1.0	0.244843	0.016937	0.005296	Biso	1.000000	H
H400	1.0	0.211629	0.504419	0.848365	Biso	1.000000	H
H401	1.0	0.041115	0.162784	0.156427	Biso	1.000000	H

H402	1.0	0.144758	0.542211	0.664798	Biso	1.000000	H
H403	1.0	0.210860	0.497357	0.709100	Biso	1.000000	H
H404	1.0	0.848853	0.402081	0.217726	Biso	1.000000	H
H405	1.0	0.002117	0.076061	0.112477	Biso	1.000000	H
H406	1.0	0.978687	0.953327	0.885950	Biso	1.000000	H
H407	1.0	0.934836	0.865479	0.869628	Biso	1.000000	H
H408	1.0	0.894761	0.928485	0.911217	Biso	1.000000	H
H409	1.0	0.924377	0.977474	0.838985	Biso	1.000000	H
H410	1.0	0.235154	0.456384	0.328474	Biso	1.000000	H
H411	1.0	0.271059	0.513579	0.398673	Biso	1.000000	H
H412	1.0	0.951094	0.420628	0.348427	Biso	1.000000	H
H413	1.0	0.990355	0.472201	0.393161	Biso	1.000000	H
H414	1.0	0.713930	0.724730	0.883130	Biso	1.000000	H
H415	1.0	0.618022	0.469750	0.140980	Biso	1.000000	H
H416	1.0	0.657173	0.535110	0.139327	Biso	1.000000	H
H417	1.0	0.139671	0.038350	0.491087	Biso	1.000000	H
H418	1.0	0.138427	0.929172	0.515922	Biso	1.000000	H
H419	1.0	0.200437	0.996246	0.527010	Biso	1.000000	H
H420	1.0	0.129389	0.991015	0.577304	Biso	1.000000	H
H421	1.0	0.695613	0.221057	0.068956	Biso	1.000000	H
H422	1.0	0.789644	0.342334	0.354940	Biso	1.000000	H
H423	1.0	0.430660	0.867806	0.825670	Biso	1.000000	H
H424	1.0	0.477742	0.511671	0.702236	Biso	1.000000	H
H425	1.0	0.929957	0.232707	0.595089	Biso	1.000000	H
H426	1.0	0.998324	0.280155	0.596290	Biso	1.000000	H
H427	1.0	0.020063	0.706496	0.404924	Biso	1.000000	H
H428	1.0	0.760716	0.327217	0.438705	Biso	1.000000	H
H429	1.0	0.832011	0.299996	0.493130	Biso	1.000000	H
H430	1.0	0.648092	0.347475	0.187178	Biso	1.000000	H
H431	1.0	0.610769	0.293828	0.162190	Biso	1.000000	H
H432	1.0	0.036383	0.866441	0.098080	Biso	1.000000	H
H433	1.0	0.772979	0.518101	0.104691	Biso	1.000000	H
H434	1.0	0.189024	0.038817	0.721092	Biso	1.000000	H
H435	1.0	0.167528	0.136969	0.718032	Biso	1.000000	H
H436	1.0	0.656265	0.574364	0.832522	Biso	1.000000	H
H437	1.0	0.542052	0.236146	0.126164	Biso	1.000000	H
H438	1.0	0.603737	0.291404	0.071686	Biso	1.000000	H
H439	1.0	0.575091	0.337955	0.145057	Biso	1.000000	H
H440	1.0	0.674427	0.222999	0.846593	Biso	1.000000	H
H441	1.0	0.698836	0.273368	0.917321	Biso	1.000000	H
H442	1.0	0.752758	0.208886	0.850680	Biso	1.000000	H
H443	1.0	0.115069	0.664178	0.695570	Biso	1.000000	H
H444	1.0	0.627125	0.149602	0.499660	Biso	1.000000	H
H445	1.0	0.665681	0.241710	0.492791	Biso	1.000000	H
H446	1.0	0.846702	0.179671	0.231368	Biso	1.000000	H
H447	1.0	0.815955	0.264024	0.221024	Biso	1.000000	H
H448	1.0	0.200500	0.290874	0.037680	Biso	1.000000	H
H449	1.0	0.130996	0.182717	0.706281	Biso	1.000000	H
H450	1.0	0.997180	0.341699	0.435260	Biso	1.000000	H
H451	1.0	0.070851	0.420893	0.708381	Biso	1.000000	H
H452	1.0	0.258543	0.091635	0.326046	Biso	1.000000	H
H453	1.0	0.158769	0.127063	0.346636	Biso	1.000000	H
H454	1.0	0.789511	0.824085	0.659566	Biso	1.000000	H
H455	1.0	0.792643	0.759232	0.686721	Biso	1.000000	H
H456	1.0	0.834802	0.852903	0.733100	Biso	1.000000	H
H457	1.0	0.919571	0.594060	0.613041	Biso	1.000000	H
H458	1.0	0.141866	0.308121	0.967420	Biso	1.000000	H
H459	1.0	0.181541	0.381026	0.964055	Biso	1.000000	H
H460	1.0	0.563696	0.837712	0.506229	Biso	1.000000	H
H461	1.0	0.507203	0.755124	0.550513	Biso	1.000000	H
H462	1.0	0.549767	0.829804	0.589683	Biso	1.000000	H
H463	1.0	0.160712	0.736277	0.107894	Biso	1.000000	H
H464	1.0	0.532199	0.244394	0.924815	Biso	1.000000	H
H465	1.0	0.925572	0.344564	0.627631	Biso	1.000000	H
H466	1.0	0.068419	0.933615	0.114976	Biso	1.000000	H
H467	1.0	0.984486	0.972508	0.124888	Biso	1.000000	H
H468	1.0	0.989012	0.878599	0.120585	Biso	1.000000	H
H469	1.0	0.087176	0.632995	0.976085	Biso	1.000000	H
H470	1.0	0.709210	0.940267	0.822930	Biso	1.000000	H
H471	1.0	0.599813	0.896075	0.817446	Biso	1.000000	H
H472	1.0	0.404105	0.632484	0.700476	Biso	1.000000	H
H473	1.0	0.439904	0.725481	0.694223	Biso	1.000000	H
H474	1.0	0.871746	0.465931	0.731213	Biso	1.000000	H

H475	1.0	0.377708	0.923664	0.502413	Biso	1.000000	H
H476	1.0	0.382510	0.885559	0.442101	Biso	1.000000	H
H477	1.0	0.313667	0.836639	0.511616	Biso	1.000000	H
H478	1.0	0.956038	0.420920	0.427980	Biso	1.000000	H
H479	1.0	0.298273	0.509433	0.736498	Biso	1.000000	H
H480	1.0	0.295064	0.424758	0.752714	Biso	1.000000	H
H481	1.0	0.364617	0.417898	0.752999	Biso	1.000000	H
H482	1.0	0.102275	0.606866	0.062124	Biso	1.000000	H
H483	1.0	0.047782	0.490135	0.506050	Biso	1.000000	H
H484	1.0	0.913508	0.480383	0.235198	Biso	1.000000	H
H485	1.0	0.861713	0.420331	0.169504	Biso	1.000000	H
H486	1.0	0.694912	0.251775	0.816998	Biso	1.000000	H
H487	1.0	0.994923	0.707610	0.331124	Biso	1.000000	H
H488	1.0	0.955903	0.667181	0.275070	Biso	1.000000	H
H489	1.0	0.465053	0.458825	0.687783	Biso	1.000000	H
H490	1.0	0.485267	0.507582	0.761046	Biso	1.000000	H
H491	1.0	0.484530	0.412811	0.768311	Biso	1.000000	H
H492	1.0	0.689055	0.713994	0.478470	Biso	1.000000	H
H493	1.0	0.668012	0.770398	0.552608	Biso	1.000000	H
H494	1.0	0.697413	0.110317	0.828565	Biso	1.000000	H
H495	1.0	0.680092	0.025908	0.880600	Biso	1.000000	H
H496	1.0	0.733592	0.065863	0.918061	Biso	1.000000	H
H497	1.0	0.871104	0.643722	0.258675	Biso	1.000000	H
H498	1.0	0.915412	0.679779	0.198789	Biso	1.000000	H
H499	1.0	0.988894	0.525180	0.154791	Biso	1.000000	H
H500	1.0	0.032918	0.586195	0.224268	Biso	1.000000	H
H501	1.0	0.786191	0.331369	0.083320	Biso	1.000000	H
H502	1.0	0.813681	0.779831	0.795910	Biso	1.000000	H
H503	1.0	0.295759	0.324463	0.799475	Biso	1.000000	H
H504	1.0	0.293582	0.300202	0.719138	Biso	1.000000	H
H505	1.0	0.023891	0.256128	0.048684	Biso	1.000000	H
H506	1.0	0.864263	0.838991	0.481644	Biso	1.000000	H
H507	1.0	0.855421	0.320279	0.089054	Biso	1.000000	H
H508	1.0	0.799144	0.297288	0.120175	Biso	1.000000	H
H509	1.0	0.943523	0.598766	0.421277	Biso	1.000000	H
H510	1.0	0.970464	0.689831	0.415104	Biso	1.000000	H
H511	1.0	0.385603	0.797559	0.562195	Biso	1.000000	H
H512	1.0	0.249136	0.601614	0.048118	Biso	1.000000	H
H513	1.0	0.791856	0.061976	0.651388	Biso	1.000000	H
H514	1.0	0.779752	0.028191	0.230601	Biso	1.000000	H
H515	1.0	0.821825	0.136813	0.241567	Biso	1.000000	H
H516	1.0	0.748735	0.119623	0.247021	Biso	1.000000	H
H517	1.0	0.346031	0.674784	0.018112	Biso	1.000000	H
H518	1.0	0.349745	0.728482	0.083218	Biso	1.000000	H
H519	1.0	0.709592	0.728082	0.019105	Biso	1.000000	H
H520	1.0	0.717193	0.698910	0.925598	Biso	1.000000	H
H521	1.0	0.693499	0.790966	0.622126	Biso	1.000000	H
H522	1.0	0.703848	0.878088	0.589957	Biso	1.000000	H
H523	1.0	0.463314	0.637711	0.169198	Biso	1.000000	H
H524	1.0	0.557427	0.621905	0.142166	Biso	1.000000	H
H525	1.0	0.509232	0.612750	0.103394	Biso	1.000000	H
H526	1.0	0.549271	0.698120	0.136458	Biso	1.000000	H
H527	1.0	0.338166	0.494386	0.696415	Biso	1.000000	H
H528	1.0	0.320652	0.573370	0.685431	Biso	1.000000	H
H529	1.0	0.741533	0.479491	0.209413	Biso	1.000000	H
H530	1.0	0.802730	0.502168	0.222423	Biso	1.000000	H
H531	1.0	0.109044	0.384866	0.696259	Biso	1.000000	H
H532	1.0	0.015734	0.440226	0.435718	Biso	1.000000	H
H533	1.0	0.072610	0.504198	0.469730	Biso	1.000000	H
H534	1.0	0.903837	0.727571	0.506675	Biso	1.000000	H
H535	1.0	0.942446	0.964057	0.320446	Biso	1.000000	H
H536	1.0	0.305331	0.188966	0.968865	Biso	1.000000	H
H537	1.0	0.296248	0.249143	0.058194	Biso	1.000000	H
H538	1.0	0.326788	0.173201	0.052832	Biso	1.000000	H
H539	1.0	0.402963	0.295712	0.910394	Biso	1.000000	H
H540	1.0	0.407260	0.367584	0.944730	Biso	1.000000	H
H541	1.0	0.458811	0.979546	0.041698	Biso	1.000000	H
H542	1.0	0.432229	0.987548	0.975598	Biso	1.000000	H
H543	1.0	0.094038	0.935137	0.538667	Biso	1.000000	H
H544	1.0	0.012082	0.875374	0.520670	Biso	1.000000	H
H545	1.0	0.852597	0.269916	0.366490	Biso	1.000000	H
H546	1.0	0.903633	0.324484	0.430024	Biso	1.000000	H
H547	1.0	0.861796	0.238244	0.443179	Biso	1.000000	H
H548	1.0	0.130214	0.515885	0.867830	Biso	1.000000	H
H549	1.0	0.131053	0.596806	0.792099	Biso	1.000000	H
H550	1.0	0.462192	0.033850	0.575730	Biso	1.000000	H
H551	1.0	0.442393	0.116584	0.562743	Biso	1.000000	H
H552	1.0	0.607703	0.194875	0.261679	Biso	1.000000	H
H553	1.0	0.969032	0.547138	0.663170	Biso	1.000000	H
H554	1.0	0.830624	0.378112	0.814712	Biso	1.000000	H

H555	1.0	0.916797	0.419828	0.777285	Biso	1.000000	H
H556	1.0	0.840500	0.463401	0.771473	Biso	1.000000	H
H557	1.0	0.980493	0.746958	0.258885	Biso	1.000000	H
H558	1.0	0.213545	0.159661	0.131674	Biso	1.000000	H
H559	1.0	0.370931	0.891916	0.034670	Biso	1.000000	H
H560	1.0	0.296068	0.900654	0.991252	Biso	1.000000	H
H561	1.0	0.280606	0.866157	0.070188	Biso	1.000000	H
H562	1.0	0.569519	0.307551	0.935487	Biso	1.000000	H
H563	1.0	0.265108	0.304607	0.429111	Biso	1.000000	H
H564	1.0	0.941217	0.077032	0.996391	Biso	1.000000	H
H565	1.0	0.993818	0.025049	0.005178	Biso	1.000000	H
H566	1.0	0.107511	0.088929	0.426970	Biso	1.000000	H
H567	1.0	0.216462	0.579097	0.508289	Biso	1.000000	H
H568	1.0	0.631454	0.305524	0.336954	Biso	1.000000	H
H569	1.0	0.554953	0.296015	0.261327	Biso	1.000000	H
H570	1.0	0.145147	0.609388	0.189241	Biso	1.000000	H
H571	1.0	0.212514	0.628565	0.164875	Biso	1.000000	H
H572	1.0	0.835848	0.844724	0.656697	Biso	1.000000	H
H573	1.0	0.231658	0.968556	0.722100	Biso	1.000000	H
H574	1.0	0.198084	0.969972	0.626604	Biso	1.000000	H
H575	1.0	0.234545	0.028931	0.706012	Biso	1.000000	H
H576	1.0	0.747931	0.129031	0.505943	Biso	1.000000	H
H577	1.0	0.723823	0.979639	0.549682	Biso	1.000000	H
H578	1.0	0.665884	0.974178	0.602241	Biso	1.000000	H
H579	1.0	0.666712	0.052280	0.522558	Biso	1.000000	H
H580	1.0	0.387199	0.036205	0.827992	Biso	1.000000	H
H581	1.0	0.404537	0.988253	0.876450	Biso	1.000000	H
H582	1.0	0.768954	0.911363	0.995010	Biso	1.000000	H
H583	1.0	0.787116	0.840218	0.997394	Biso	1.000000	H
H584	1.0	0.453444	0.466171	0.112829	Biso	1.000000	H
H585	1.0	0.346179	0.607355	0.760357	Biso	1.000000	H
H586	1.0	0.348482	0.651190	0.705495	Biso	1.000000	H
H587	1.0	0.315416	0.491535	0.289917	Biso	1.000000	H
H588	1.0	0.334271	0.592081	0.343125	Biso	1.000000	H
H589	1.0	0.319106	0.574367	0.261700	Biso	1.000000	H
H590	1.0	0.728551	0.362641	0.290384	Biso	1.000000	H
H591	1.0	0.681678	0.313886	0.351421	Biso	1.000000	H
H592	1.0	0.781771	0.298334	0.326551	Biso	1.000000	H
H593	1.0	0.769141	0.031946	0.920586	Biso	1.000000	H
H594	1.0	0.719909	0.954088	0.897121	Biso	1.000000	H
H595	1.0	0.847650	0.634135	0.440031	Biso	1.000000	H
H596	1.0	0.814228	0.624037	0.388395	Biso	1.000000	H
H597	1.0	0.757022	0.697209	0.419653	Biso	1.000000	H
H598	1.0	0.830001	0.692942	0.062293	Biso	1.000000	H
H599	1.0	0.852582	0.733060	0.136821	Biso	1.000000	H
H600	1.0	0.808862	0.926796	0.328983	Biso	1.000000	H
H601	1.0	0.844034	0.981574	0.273520	Biso	1.000000	H
H602	1.0	0.788887	0.027049	0.277257	Biso	1.000000	H
H603	1.0	0.993994	0.775579	0.610396	Biso	1.000000	H
H604	1.0	0.749976	0.313557	0.259532	Biso	1.000000	H
H605	1.0	0.813491	0.298488	0.175325	Biso	1.000000	H
H606	1.0	0.405363	0.730225	0.442983	Biso	1.000000	H
H607	1.0	0.433556	0.657429	0.442412	Biso	1.000000	H
H608	1.0	0.425444	0.747599	0.496278	Biso	1.000000	H
H609	1.0	0.747403	0.817611	0.965837	Biso	1.000000	H
H610	1.0	0.158830	0.763608	0.961846	Biso	1.000000	H
H611	1.0	0.306101	0.675773	0.691342	Biso	1.000000	H
H612	1.0	0.655791	0.252762	0.887741	Biso	1.000000	H
H613	1.0	0.629843	0.473009	0.592739	Biso	1.000000	H
H614	1.0	0.661274	0.553303	0.527756	Biso	1.000000	H
H615	1.0	0.018612	0.572196	0.593588	Biso	1.000000	H
H616	1.0	0.041156	0.625073	0.674687	Biso	1.000000	H
H617	1.0	0.958846	0.576535	0.626654	Biso	1.000000	H
H618	1.0	0.929836	0.099739	0.357729	Biso	1.000000	H
H619	1.0	0.891022	0.091916	0.412362	Biso	1.000000	H
H620	1.0	0.850890	0.164752	0.398253	Biso	1.000000	H
H621	1.0	0.853695	0.137665	0.322456	Biso	1.000000	H
H622	1.0	0.245944	0.397859	0.072691	Biso	1.000000	H
H623	1.0	0.301972	0.330193	0.013271	Biso	1.000000	H
H624	1.0	0.343251	0.228636	0.802420	Biso	1.000000	H
H625	1.0	0.936264	0.467888	0.778786	Biso	1.000000	H
H626	1.0	0.718851	0.874220	0.261261	Biso	1.000000	H
H627	1.0	0.275287	0.350686	0.141945	Biso	1.000000	H
H628	1.0	0.344664	0.424728	0.178866	Biso	1.000000	H

H629	1.0	0.360508	0.387496	0.123745	Biso	1.000000	H
H630	1.0	0.855961	0.310451	0.212575	Biso	1.000000	H
H631	1.0	0.352330	0.493192	0.866081	Biso	1.000000	H
H632	1.0	0.411731	0.513807	0.804032	Biso	1.000000	H
H633	1.0	0.943650	0.993093	0.596236	Biso	1.000000	H
H634	1.0	0.646424	0.173616	0.864894	Biso	1.000000	H
H635	1.0	0.592234	0.090100	0.862716	Biso	1.000000	H
H636	1.0	0.554694	0.692136	0.737194	Biso	1.000000	H
H637	1.0	0.606941	0.700026	0.675702	Biso	1.000000	H
H638	1.0	0.731298	0.117887	0.203938	Biso	1.000000	H
H639	1.0	0.100281	0.848375	0.329382	Biso	1.000000	H
H640	1.0	0.182528	0.837958	0.366030	Biso	1.000000	H
H641	1.0	0.269801	0.937440	0.845555	Biso	1.000000	H
H642	1.0	0.766961	0.643536	0.366264	Biso	1.000000	H
H643	1.0	0.733979	0.600455	0.304502	Biso	1.000000	H
H644	1.0	0.136728	0.464941	0.704390	Biso	1.000000	H
H645	1.0	0.208822	0.437272	0.707508	Biso	1.000000	H
H646	1.0	0.943965	0.115002	0.503135	Biso	1.000000	H
H647	1.0	0.593448	0.629338	0.498534	Biso	1.000000	H
H648	1.0	0.615600	0.733463	0.489041	Biso	1.000000	H
H649	1.0	0.140158	0.414092	0.822871	Biso	1.000000	H
H650	1.0	0.172483	0.325217	0.788427	Biso	1.000000	H
H651	1.0	0.639775	0.188852	0.415705	Biso	1.000000	H
H652	1.0	0.239979	0.662983	0.550152	Biso	1.000000	H
H653	1.0	0.311223	0.727625	0.501016	Biso	1.000000	H
H654	1.0	0.373353	0.636284	0.516557	Biso	1.000000	H
H655	1.0	0.336412	0.701256	0.570081	Biso	1.000000	H
H656	1.0	0.362893	0.025931	0.348986	Biso	1.000000	H
H657	1.0	0.302525	0.065177	0.312621	Biso	1.000000	H
H658	1.0	0.908859	0.182386	0.006143	Biso	1.000000	H
H659	1.0	0.828767	0.106005	0.003993	Biso	1.000000	H
H660	1.0	0.820177	0.642590	0.815936	Biso	1.000000	H
H661	1.0	0.791328	0.682536	0.750555	Biso	1.000000	H
H662	1.0	0.609738	0.159211	0.783616	Biso	1.000000	H
H663	1.0	0.862751	0.570296	0.426045	Biso	1.000000	H
H664	1.0	0.795395	0.583351	0.497052	Biso	1.000000	H
H665	1.0	0.062143	0.810222	0.563816	Biso	1.000000	H
H666	1.0	0.781870	0.909884	0.666615	Biso	1.000000	H
H667	1.0	0.762650	0.973859	0.636109	Biso	1.000000	H
H668	1.0	0.843518	0.900330	0.630670	Biso	1.000000	H
H669	1.0	0.152272	0.637327	0.684348	Biso	1.000000	H
H670	1.0	0.133156	0.569029	0.748957	Biso	1.000000	H
H671	1.0	0.127917	0.010473	0.110482	Biso	1.000000	H
H672	1.0	0.015821	0.258737	0.638721	Biso	1.000000	H
H673	1.0	0.102486	0.320403	0.098345	Biso	1.000000	H
H674	1.0	0.830042	0.930123	0.584057	Biso	1.000000	H
H675	1.0	0.065488	0.313335	0.308110	Biso	1.000000	H
H676	1.0	0.622602	0.056893	0.529948	Biso	1.000000	H
H677	1.0	0.679841	0.981544	0.342955	Biso	1.000000	H
H678	1.0	0.989600	0.658085	0.453849	Biso	1.000000	H
H679	1.0	0.106738	0.825079	0.717732	Biso	1.000000	H
H680	1.0	0.264854	0.791764	0.400299	Biso	1.000000	H
H681	1.0	0.617001	0.546108	0.218593	Biso	1.000000	H
H682	1.0	0.568007	0.485759	0.197566	Biso	1.000000	H
H683	1.0	0.538407	0.597690	0.180459	Biso	1.000000	H
H684	1.0	0.707555	0.417171	0.306220	Biso	1.000000	H
H685	1.0	0.140287	0.573486	0.550687	Biso	1.000000	H
H686	1.0	0.072806	0.830082	0.245973	Biso	1.000000	H
H687	1.0	0.131244	0.847262	0.181812	Biso	1.000000	H
H688	1.0	0.877354	0.702972	0.576238	Biso	1.000000	H
H689	1.0	0.866749	0.027552	0.347773	Biso	1.000000	H
H690	1.0	0.897788	0.945143	0.331834	Biso	1.000000	H
H691	1.0	0.311300	0.282366	0.207056	Biso	1.000000	H
H692	1.0	0.218868	0.314762	0.197834	Biso	1.000000	H
H693	1.0	0.273420	0.236343	0.184708	Biso	1.000000	H
H694	1.0	0.185061	0.506336	0.440307	Biso	1.000000	H
H695	1.0	0.743292	0.489240	0.077337	Biso	1.000000	H
H696	1.0	0.636968	0.546603	0.058460	Biso	1.000000	H
H697	1.0	0.031323	0.651296	0.288994	Biso	1.000000	H
H698	1.0	0.940880	0.303234	0.864812	Biso	1.000000	H
H699	1.0	0.914106	0.247398	0.911329	Biso	1.000000	H
H700	1.0	0.140639	0.656402	0.199550	Biso	1.000000	H
H701	1.0	0.138566	0.877453	0.524776	Biso	1.000000	H
H702	1.0	0.042001	0.112144	0.211575	Biso	1.000000	H
H703	1.0	0.085509	0.103629	0.263637	Biso	1.000000	H
H704	1.0	0.491495	0.062556	0.604190	Biso	1.000000	H
H705	1.0	0.965302	0.622832	0.578666	Biso	1.000000	H
H706	1.0	0.359127	0.705475	0.457056	Biso	1.000000	H
H707	1.0	0.327388	0.778766	0.519308	Biso	1.000000	H
H708	1.0	0.268433	0.073799	0.801840	Biso	1.000000	H
H709	1.0	0.173762	0.766900	0.302517	Biso	1.000000	H
H710	1.0	0.727835	0.111107	0.976319	Biso	1.000000	H

H711	1.0	0.363751	0.769908	0.812033	Biso	1.000000	H
H712	1.0	0.916293	0.042531	0.355645	Biso	1.000000	H
H713	1.0	0.009683	0.071699	0.335874	Biso	1.000000	H
H714	1.0	0.968162	0.984214	0.364258	Biso	1.000000	H
H715	1.0	0.541105	0.985704	0.452462	Biso	1.000000	H
H716	1.0	0.111184	0.258840	0.582203	Biso	1.000000	H
H717	1.0	0.618323	0.975536	0.591186	Biso	1.000000	H
H718	1.0	0.697921	0.604688	0.921988	Biso	1.000000	H
H719	1.0	0.762273	0.573967	0.901215	Biso	1.000000	H
H720	1.0	0.718700	0.769427	0.134806	Biso	1.000000	H
H721	1.0	0.776313	0.782304	0.188618	Biso	1.000000	H
H722	1.0	0.347081	0.502303	0.124188	Biso	1.000000	H
H723	1.0	0.254434	0.511798	0.114561	Biso	1.000000	H
H724	1.0	0.048806	0.944005	0.385974	Biso	1.000000	H
H725	1.0	0.256747	0.869406	0.787092	Biso	1.000000	H
H726	1.0	0.412945	0.850143	0.570291	Biso	1.000000	H
H727	1.0	0.497337	0.846597	0.589598	Biso	1.000000	H
H728	1.0	0.480687	0.929826	0.558502	Biso	1.000000	H
H729	1.0	0.763469	0.228696	0.468019	Biso	1.000000	H
H730	1.0	0.748935	0.159412	0.466901	Biso	1.000000	H
H731	1.0	0.149160	0.094955	0.816271	Biso	1.000000	H
H732	1.0	0.061337	0.075469	0.771747	Biso	1.000000	H
H733	1.0	0.873706	0.718065	0.748585	Biso	1.000000	H
H734	1.0	0.972269	0.739820	0.786870	Biso	1.000000	H
H735	1.0	0.017567	0.387685	0.429568	Biso	1.000000	H
H736	1.0	0.991960	0.329882	0.365341	Biso	1.000000	H
H737	1.0	0.156620	0.363379	0.842449	Biso	1.000000	H
H738	1.0	0.101503	0.273211	0.877566	Biso	1.000000	H
H739	1.0	0.811922	0.121128	0.639599	Biso	1.000000	H
H740	1.0	0.737200	0.628086	0.055272	Biso	1.000000	H
H741	1.0	0.648326	0.650999	0.077141	Biso	1.000000	H
H742	1.0	0.655412	0.577264	0.088823	Biso	1.000000	H
H743	1.0	0.002120	0.269083	0.757242	Biso	1.000000	H
H744	1.0	0.032307	0.238587	0.686047	Biso	1.000000	H
H745	1.0	0.977826	0.165838	0.713485	Biso	1.000000	H
H746	1.0	0.248189	0.527494	0.728603	Biso	1.000000	H
H747	1.0	0.403834	0.564534	0.583788	Biso	1.000000	H
H748	1.0	0.408426	0.473810	0.611967	Biso	1.000000	H
H749	1.0	0.126458	0.362651	0.630816	Biso	1.000000	H
H750	1.0	0.552761	0.781447	0.442525	Biso	1.000000	H
H751	1.0	0.564932	0.704653	0.415344	Biso	1.000000	H
H752	1.0	0.047582	0.934370	0.305432	Biso	1.000000	H
H753	1.0	0.017349	0.007685	0.257082	Biso	1.000000	H
H754	1.0	0.320176	0.271589	0.692842	Biso	1.000000	H
H755	1.0	0.338133	0.301864	0.771310	Biso	1.000000	H
H756	1.0	0.057800	0.405765	0.453708	Biso	1.000000	H
H757	1.0	0.972067	0.427942	0.481113	Biso	1.000000	H
H758	1.0	0.739634	0.124651	0.098552	Biso	1.000000	H
H759	1.0	0.684973	0.079553	0.047227	Biso	1.000000	H
H760	1.0	0.200199	0.713546	0.094551	Biso	1.000000	H
H761	1.0	0.408310	0.196007	0.212644	Biso	1.000000	H
H762	1.0	0.469759	0.113953	0.219715	Biso	1.000000	H
H763	1.0	0.472457	0.185248	0.281760	Biso	1.000000	H
H764	1.0	0.403294	0.182482	0.280098	Biso	1.000000	H
H765	1.0	0.570886	0.961015	0.060101	Biso	1.000000	H
H766	1.0	0.064790	0.810527	0.326562	Biso	1.000000	H
H767	1.0	0.012621	0.742923	0.288423	Biso	1.000000	H
H768	1.0	0.953884	0.307221	0.279572	Biso	1.000000	H
H769	1.0	0.282330	0.494454	0.046353	Biso	1.000000	H
H770	1.0	0.284553	0.518072	0.946854	Biso	1.000000	H
H771	1.0	0.238780	0.568745	0.007603	Biso	1.000000	H
H772	1.0	0.118730	0.640930	0.290431	Biso	1.000000	H
H773	1.0	0.041254	0.705940	0.302317	Biso	1.000000	H
H774	1.0	0.677221	0.840131	0.193751	Biso	1.000000	H
H775	1.0	0.934354	0.165106	0.599435	Biso	1.000000	H
H776	1.0	0.764811	0.690590	0.521689	Biso	1.000000	H
H777	1.0	0.125066	0.547706	0.283204	Biso	1.000000	H
H778	1.0	0.464757	0.138167	0.977023	Biso	1.000000	H
H779	1.0	0.484710	0.234040	0.684333	Biso	1.000000	H
H780	1.0	0.422344	0.274457	0.731614	Biso	1.000000	H

H781	1.0	0.526926	0.252572	0.745090	Biso	1.000000	H
H782	1.0	0.143820	0.966140	0.299410	Biso	1.000000	H
H783	1.0	0.199297	0.031929	0.295627	Biso	1.000000	H
H784	1.0	0.155826	0.079950	0.325121	Biso	1.000000	H
H785	1.0	0.085547	0.425853	0.301927	Biso	1.000000	H
H786	1.0	0.270231	0.587806	0.923350	Biso	1.000000	H
H787	1.0	0.274246	0.484202	0.899648	Biso	1.000000	H
H788	1.0	0.727998	0.535075	0.527487	Biso	1.000000	H
H789	1.0	0.714613	0.476907	0.698119	Biso	1.000000	H
H790	1.0	0.907581	0.684079	0.541069	Biso	1.000000	H
H791	1.0	0.298445	0.430488	0.196333	Biso	1.000000	H
H792	1.0	0.302488	0.523972	0.189241	Biso	1.000000	H
H793	1.0	0.309115	0.446221	0.263055	Biso	1.000000	H
H794	1.0	0.199233	0.106645	0.796811	Biso	1.000000	H
H795	1.0	0.186767	0.113038	0.891773	Biso	1.000000	H
H796	1.0	0.251015	0.156584	0.846376	Biso	1.000000	H
H797	1.0	0.676126	0.659056	0.504544	Biso	1.000000	H
H798	1.0	0.158276	0.749910	0.014549	Biso	1.000000	H
H799	1.0	0.260839	0.772179	0.980008	Biso	1.000000	H
O1600	1.0	0.487970	0.277844	0.625446	Biso	1.000000	O
H800	1.0	0.469645	0.289420	0.637582	Biso	1.000000	H
H801	1.0	0.479624	0.269983	0.604329	Biso	1.000000	H
O1601	1.0	0.204420	0.421220	0.220538	Biso	1.000000	O
H802	1.0	0.206166	0.448094	0.218654	Biso	1.000000	H
H803	1.0	0.183528	0.414822	0.231765	Biso	1.000000	H
O1602	1.0	0.681599	0.574086	0.395679	Biso	1.000000	O
H804	1.0	0.663324	0.560701	0.384980	Biso	1.000000	H
H805	1.0	0.685279	0.597701	0.384751	Biso	1.000000	H
O1603	1.0	0.135685	0.339631	0.035302	Biso	1.000000	O
H806	1.0	0.150727	0.320306	0.043367	Biso	1.000000	H
H807	1.0	0.132363	0.336415	0.012090	Biso	1.000000	H
O1604	1.0	0.984716	0.109864	0.434800	Biso	1.000000	O
H808	1.0	0.980281	0.134685	0.443120	Biso	1.000000	H
H809	1.0	0.998563	0.111588	0.415330	Biso	1.000000	H
O1605	1.0	0.416750	0.282430	0.291805	Biso	1.000000	O
H810	1.0	0.438562	0.283871	0.280868	Biso	1.000000	H
H811	1.0	0.418319	0.294534	0.312850	Biso	1.000000	H
O1606	1.0	0.286637	0.934213	0.438250	Biso	1.000000	O
H812	1.0	0.293592	0.954052	0.452830	Biso	1.000000	H
H813	1.0	0.277898	0.913466	0.450852	Biso	1.000000	H
O1607	1.0	0.724518	0.717521	0.341388	Biso	1.000000	O
H814	1.0	0.742845	0.735147	0.337844	Biso	1.000000	H
H815	1.0	0.733949	0.692928	0.345178	Biso	1.000000	H
O1608	1.0	0.972855	0.550433	0.796110	Biso	1.000000	O
H816	1.0	0.990364	0.534927	0.805750	Biso	1.000000	H
H817	1.0	0.980376	0.576172	0.795912	Biso	1.000000	H
O1609	1.0	0.907256	0.394210	0.896284	Biso	1.000000	O
H818	1.0	0.901129	0.412238	0.912860	Biso	1.000000	H
H819	1.0	0.898678	0.369849	0.902378	Biso	1.000000	H
O1610	1.0	0.287367	0.589433	0.703638	Biso	1.000000	O
H820	1.0	0.276118	0.612834	0.708489	Biso	1.000000	H
H821	1.0	0.279852	0.570537	0.718902	Biso	1.000000	H
O1611	1.0	0.398795	0.408865	0.123121	Biso	1.000000	O
H822	1.0	0.408739	0.424223	0.106195	Biso	1.000000	H
H823	1.0	0.415623	0.391264	0.130966	Biso	1.000000	H
O1612	1.0	0.257921	0.021050	0.606554	Biso	1.000000	O
H824	1.0	0.278831	0.006720	0.606797	Biso	1.000000	H
H825	1.0	0.241559	0.009691	0.621163	Biso	1.000000	H
O1613	1.0	0.446331	0.915514	0.343712	Biso	1.000000	O
H826	1.0	0.461359	0.916566	0.362398	Biso	1.000000	H
H827	1.0	0.423338	0.921512	0.350451	Biso	1.000000	H
O1614	1.0	0.308678	0.973899	0.609747	Biso	1.000000	O
H828	1.0	0.319621	0.967245	0.589414	Biso	1.000000	H
H829	1.0	0.292132	0.954950	0.615448	Biso	1.000000	H
O1615	1.0	0.330791	0.671313	0.458132	Biso	1.000000	O
H830	1.0	0.313976	0.682738	0.472222	Biso	1.000000	H
H831	1.0	0.338619	0.648198	0.467797	Biso	1.000000	H
O1616	1.0	0.276843	0.736147	0.129131	Biso	1.000000	O
H832	1.0	0.256893	0.726819	0.117898	Biso	1.000000	H
H833	1.0	0.286730	0.756437	0.116737	Biso	1.000000	H
O1617	1.0	0.623927	0.047347	0.336669	Biso	1.000000	O
H834	1.0	0.645934	0.037856	0.343368	Biso	1.000000	H
H835	1.0	0.610951	0.054523	0.355732	Biso	1.000000	H
O1618	1.0	0.154117	0.113527	0.914490	Biso	1.000000	O
H836	1.0	0.153446	0.096273	0.932642	Biso	1.000000	H
H837	1.0	0.155151	0.138861	0.922654	Biso	1.000000	H
O1619	1.0	0.371491	0.585089	0.572148	Biso	1.000000	O
H838	1.0	0.347385	0.579583	0.570813	Biso	1.000000	H
H839	1.0	0.376431	0.607328	0.559597	Biso	1.000000	H
O1620	1.0	0.013893	0.220998	0.406800	Biso	1.000000	O
H840	1.0	0.023175	0.242151	0.418499	Biso	1.000000	H

H841	1.0	0.031002	0.201613	0.405226	Biso	1.000000	H
O1621	1.0	0.673621	0.392748	0.529361	Biso	1.000000	O
H842	1.0	0.665509	0.367438	0.532246	Biso	1.000000	H
H843	1.0	0.694856	0.392397	0.517364	Biso	1.000000	H
O1622	1.0	0.981983	0.045913	0.466421	Biso	1.000000	O
H844	1.0	0.960740	0.034025	0.460421	Biso	1.000000	H
H845	1.0	0.983650	0.069920	0.455697	Biso	1.000000	H
O1623	1.0	0.587299	0.702696	0.140275	Biso	1.000000	O
H846	1.0	0.605945	0.691609	0.152312	Biso	1.000000	H
H847	1.0	0.595951	0.713921	0.120470	Biso	1.000000	H
O1624	1.0	0.280296	0.878737	0.361209	Biso	1.000000	O
H848	1.0	0.295111	0.867841	0.377503	Biso	1.000000	H
H849	1.0	0.263409	0.894267	0.371790	Biso	1.000000	H
O1625	1.0	0.641775	0.666567	0.331888	Biso	1.000000	O
H850	1.0	0.617536	0.669327	0.335524	Biso	1.000000	H
H851	1.0	0.650637	0.689058	0.321918	Biso	1.000000	H
O1626	1.0	0.510244	0.929461	0.082552	Biso	1.000000	O
H852	1.0	0.514234	0.912322	0.100393	Biso	1.000000	H
H853	1.0	0.501318	0.952722	0.091010	Biso	1.000000	H
O1627	1.0	0.408663	0.504711	0.247452	Biso	1.000000	O
H854	1.0	0.397187	0.480788	0.247207	Biso	1.000000	H
H855	1.0	0.432779	0.501046	0.243677	Biso	1.000000	H
O1628	1.0	0.453866	0.635149	0.064516	Biso	1.000000	O
H856	1.0	0.441897	0.658648	0.062333	Biso	1.000000	H
H857	1.0	0.469594	0.636619	0.082648	Biso	1.000000	H
O1629	1.0	0.899363	0.684346	0.907790	Biso	1.000000	O
H858	1.0	0.909609	0.689931	0.928694	Biso	1.000000	H
H859	1.0	0.914715	0.691804	0.890506	Biso	1.000000	H
O1630	1.0	0.368625	0.371801	0.759342	Biso	1.000000	O
H860	1.0	0.372840	0.378031	0.781948	Biso	1.000000	H
H861	1.0	0.365115	0.345145	0.757332	Biso	1.000000	H
O1631	1.0	0.846162	0.026598	0.787660	Biso	1.000000	O
H862	1.0	0.829569	0.045211	0.781278	Biso	1.000000	H
H863	1.0	0.866929	0.030199	0.775324	Biso	1.000000	H
O1632	1.0	0.869928	0.448097	0.285497	Biso	1.000000	O
H864	1.0	0.883569	0.449322	0.265870	Biso	1.000000	H
H865	1.0	0.856388	0.470582	0.287411	Biso	1.000000	H
O1633	1.0	0.117662	0.854443	0.549040	Biso	1.000000	O
H866	1.0	0.123407	0.828693	0.544429	Biso	1.000000	H
H867	1.0	0.093657	0.856251	0.554217	Biso	1.000000	H
O1634	1.0	0.523722	0.548455	0.932407	Biso	1.000000	O
H868	1.0	0.514242	0.552317	0.910887	Biso	1.000000	H
H869	1.0	0.539571	0.568372	0.937364	Biso	1.000000	H
O1635	1.0	0.534515	0.849137	0.035476	Biso	1.000000	O
H870	1.0	0.549108	0.845845	0.054280	Biso	1.000000	H
H871	1.0	0.538224	0.828874	0.020268	Biso	1.000000	H
O1636	1.0	0.944364	0.744596	0.268545	Biso	1.000000	O
H872	1.0	0.923339	0.745696	0.256247	Biso	1.000000	H
H873	1.0	0.945514	0.721157	0.280240	Biso	1.000000	H
O1637	1.0	0.821635	0.160320	0.424630	Biso	1.000000	O
H874	1.0	0.823669	0.146836	0.444986	Biso	1.000000	H
H875	1.0	0.807439	0.182120	0.427818	Biso	1.000000	H
O1638	1.0	0.703078	0.343015	0.627591	Biso	1.000000	O
H876	1.0	0.702985	0.368979	0.634146	Biso	1.000000	H
H877	1.0	0.719499	0.339450	0.610261	Biso	1.000000	H
O1639	1.0	0.474055	0.498170	0.234862	Biso	1.000000	O
H878	1.0	0.475535	0.524523	0.229804	Biso	1.000000	H
H879	1.0	0.482125	0.483751	0.216460	Biso	1.000000	H
O1640	1.0	0.978128	0.164003	0.067854	Biso	1.000000	O
H880	1.0	0.960154	0.145497	0.068131	Biso	1.000000	H
H881	1.0	0.999617	0.152197	0.073134	Biso	1.000000	H
O1641	1.0	0.782566	0.677862	0.887861	Biso	1.000000	O
H882	1.0	0.781130	0.650880	0.887899	Biso	1.000000	H
H883	1.0	0.776822	0.687214	0.866416	Biso	1.000000	H
O1642	1.0	0.413687	0.837029	0.126261	Biso	1.000000	O
H884	1.0	0.428263	0.816149	0.120784	Biso	1.000000	H
H885	1.0	0.408786	0.836623	0.149384	Biso	1.000000	H
O1643	1.0	0.047394	0.856359	0.271790	Biso	1.000000	O
H886	1.0	0.039999	0.880039	0.280695	Biso	1.000000	H
H887	1.0	0.043287	0.836758	0.287555	Biso	1.000000	H
O1644	1.0	0.066048	0.572280	0.496701	Biso	1.000000	O
H888	1.0	0.041390	0.572845	0.496861	Biso	1.000000	H
H889	1.0	0.074717	0.597206	0.492890	Biso	1.000000	H
O1645	1.0	0.373205	0.217772	0.673163	Biso	1.000000	O
H890	1.0	0.381311	0.192962	0.667920	Biso	1.000000	H
H891	1.0	0.372783	0.220922	0.696597	Biso	1.000000	H
O1646	1.0	0.135904	0.019597	0.832784	Biso	1.000000	O
H892	1.0	0.124129	0.012537	0.852582	Biso	1.000000	H
H893	1.0	0.128581	0.044253	0.826125	Biso	1.000000	H
O1647	1.0	0.505780	0.887669	0.137177	Biso	1.000000	O
H894	1.0	0.493167	0.889383	0.157399	Biso	1.000000	H
H895	1.0	0.519455	0.865176	0.137258	Biso	1.000000	H
O1648	1.0	0.757330	0.109735	0.340890	Biso	1.000000	O
H896	1.0	0.770315	0.105361	0.360586	Biso	1.000000	H
H897	1.0	0.766574	0.131140	0.329514	Biso	1.000000	H

O1649	1.0	0.844759	0.550733	0.187284	Biso	1.000000	O
H898	1.0	0.836045	0.526123	0.192355	Biso	1.000000	H
H899	1.0	0.868811	0.549044	0.182272	Biso	1.000000	H
O1650	1.0	0.653722	0.274579	0.116205	Biso	1.000000	O
H900	1.0	0.633498	0.271557	0.104131	Biso	1.000000	H
H901	1.0	0.665928	0.251959	0.115269	Biso	1.000000	H
H902	1.0	0.645931	0.275744	0.137896	Biso	1.000000	H
O1651	1.0	0.107986	0.154701	0.196992	Biso	1.000000	O
H903	1.0	0.115445	0.144036	0.177256	Biso	1.000000	H
H904	1.0	0.086397	0.145288	0.202733	Biso	1.000000	H
H905	1.0	0.104215	0.180095	0.192235	Biso	1.000000	H
O1652	1.0	0.201274	0.238183	0.160497	Biso	1.000000	O
H906	1.0	0.205921	0.219176	0.145265	Biso	1.000000	H
H907	1.0	0.222328	0.244880	0.169836	Biso	1.000000	H
H908	1.0	0.189539	0.224911	0.176832	Biso	1.000000	H
O1653	1.0	0.767188	0.402381	0.831897	Biso	1.000000	O
H909	1.0	0.754171	0.380315	0.832838	Biso	1.000000	H
H910	1.0	0.756721	0.417955	0.816340	Biso	1.000000	H
H911	1.0	0.763294	0.414246	0.852046	Biso	1.000000	H
O1654	1.0	0.619845	0.378693	0.366469	Biso	1.000000	O
H912	1.0	0.619598	0.357651	0.380240	Biso	1.000000	H
H913	1.0	0.596782	0.381228	0.360520	Biso	1.000000	H
H914	1.0	0.623552	0.398264	0.381389	Biso	1.000000	H
O1655	1.0	0.209731	0.422966	0.839099	Biso	1.000000	O
H915	1.0	0.207063	0.448876	0.836104	Biso	1.000000	H
H916	1.0	0.227018	0.416625	0.824169	Biso	1.000000	H
H917	1.0	0.189830	0.412019	0.830540	Biso	1.000000	H
O1656	1.0	0.024470	0.775298	0.509785	Biso	1.000000	O
H918	1.0	0.039408	0.781739	0.526850	Biso	1.000000	H
H919	1.0	0.037567	0.756845	0.499268	Biso	1.000000	H
H920	1.0	0.026541	0.796559	0.496420	Biso	1.000000	H
O1657	1.0	0.652802	0.920534	0.386002	Biso	1.000000	O
H921	1.0	0.630586	0.929804	0.382878	Biso	1.000000	H
H922	1.0	0.661466	0.933570	0.404138	Biso	1.000000	H
H923	1.0	0.664609	0.931167	0.368298	Biso	1.000000	H
O1658	1.0	0.551717	0.492175	0.789083	Biso	1.000000	O
H924	1.0	0.530196	0.490538	0.779026	Biso	1.000000	H
H925	1.0	0.565684	0.505128	0.774225	Biso	1.000000	H
H926	1.0	0.559025	0.467128	0.789168	Biso	1.000000	H
O1659	1.0	0.339051	0.352745	0.210815	Biso	1.000000	O
H927	1.0	0.332624	0.374171	0.199004	Biso	1.000000	H
H928	1.0	0.325765	0.332974	0.202592	Biso	1.000000	H
H929	1.0	0.361810	0.348088	0.204783	Biso	1.000000	H
O1660	1.0	0.350522	0.780028	0.427171	Biso	1.000000	O
H930	1.0	0.347108	0.767251	0.407371	Biso	1.000000	H
H931	1.0	0.359869	0.761248	0.440527	Biso	1.000000	H
H932	1.0	0.328581	0.783912	0.435833	Biso	1.000000	H
O1661	1.0	0.524733	0.374587	0.719608	Biso	1.000000	O
H933	1.0	0.529015	0.354835	0.734200	Biso	1.000000	H
H934	1.0	0.509126	0.390513	0.730139	Biso	1.000000	H
H935	1.0	0.512456	0.364468	0.701967	Biso	1.000000	H
O1662	1.0	0.480648	0.184834	0.043061	Biso	1.000000	O
H936	1.0	0.464493	0.170065	0.054105	Biso	1.000000	H
H937	1.0	0.478300	0.207850	0.053939	Biso	1.000000	H
H938	1.0	0.501796	0.175718	0.050436	Biso	1.000000	H
O1663	1.0	0.204892	0.088543	0.669413	Biso	1.000000	O
H939	1.0	0.225757	0.101512	0.668760	Biso	1.000000	H
H940	1.0	0.209406	0.064080	0.676653	Biso	1.000000	H
H941	1.0	0.192673	0.099112	0.686887	Biso	1.000000	H
O1664	1.0	0.058103	0.009708	0.091277	Biso	1.000000	O
H942	1.0	0.051367	0.001798	0.070351	Biso	1.000000	H
H943	1.0	0.051647	0.989580	0.104702	Biso	1.000000	H
H944	1.0	0.044676	0.030757	0.096216	Biso	1.000000	H
O1665	1.0	0.479313	0.056254	0.073613	Biso	1.000000	O
H945	1.0	0.493371	0.073304	0.062445	Biso	1.000000	H
H946	1.0	0.457632	0.067351	0.071891	Biso	1.000000	H
H947	1.0	0.478075	0.034566	0.060681	Biso	1.000000	H
O1666	1.0	0.599054	0.214348	0.164936	Biso	1.000000	O
H948	1.0	0.611158	0.200677	0.149107	Biso	1.000000	H
H949	1.0	0.613139	0.234837	0.169992	Biso	1.000000	H
H950	1.0	0.581676	0.226873	0.153482	Biso	1.000000	H
O1667	1.0	0.795990	0.506541	0.728001	Biso	1.000000	O
H951	1.0	0.778168	0.523640	0.731689	Biso	1.000000	H
H952	1.0	0.816379	0.519964	0.731057	Biso	1.000000	H
H953	1.0	0.794160	0.501414	0.705549	Biso	1.000000	H
O1668	1.0	0.412728	0.483434	0.735241	Biso	1.000000	O

H954	1.0	0.395298	0.465366	0.734733	Biso	1.000000	H
H955	1.0	0.431373	0.475866	0.722385	Biso	1.000000	H
H956	1.0	0.421081	0.482291	0.756742	Biso	1.000000	H
O1669	1.0	0.922277	0.591279	0.352769	Biso	1.000000	O
H957	1.0	0.921101	0.602558	0.373482	Biso	1.000000	H
H958	1.0	0.902962	0.598587	0.340730	Biso	1.000000	H
H959	1.0	0.940855	0.602663	0.342167	Biso	1.000000	H
O1670	1.0	0.950533	0.160008	0.895144	Biso	1.000000	O
H960	1.0	0.967498	0.176764	0.888094	Biso	1.000000	H
H961	1.0	0.961357	0.136574	0.893984	Biso	1.000000	H
H962	1.0	0.947569	0.164803	0.917540	Biso	1.000000	H
O1671	1.0	0.768582	0.284066	0.522381	Biso	1.000000	O
H963	1.0	0.783545	0.296780	0.508277	Biso	1.000000	H
H964	1.0	0.782492	0.275971	0.539703	Biso	1.000000	H
H965	1.0	0.761141	0.262921	0.510731	Biso	1.000000	H
O1672	1.0	0.689124	0.358259	0.240463	Biso	1.000000	O
H966	1.0	0.693869	0.381607	0.230918	Biso	1.000000	H
H967	1.0	0.677628	0.363076	0.260173	Biso	1.000000	H
H968	1.0	0.673117	0.346626	0.226699	Biso	1.000000	H
O1673	1.0	0.860178	0.837264	0.121802	Biso	1.000000	O
H969	1.0	0.864694	0.862519	0.117079	Biso	1.000000	H
H970	1.0	0.842140	0.837491	0.136942	Biso	1.000000	H
H971	1.0	0.879734	0.828715	0.132821	Biso	1.000000	H
O1674	1.0	0.658657	0.096151	0.977055	Biso	1.000000	O
H972	1.0	0.645253	0.084072	0.961198	Biso	1.000000	H
H973	1.0	0.643403	0.109646	0.990295	Biso	1.000000	H
H974	1.0	0.665933	0.077445	0.991616	Biso	1.000000	H
O1675	1.0	0.786766	0.869660	0.935521	Biso	1.000000	O
H975	1.0	0.798234	0.865974	0.955431	Biso	1.000000	H
H976	1.0	0.768636	0.852435	0.935712	Biso	1.000000	H
H977	1.0	0.802772	0.861381	0.920017	Biso	1.000000	H
O1676	1.0	0.180647	0.729230	0.903825	Biso	1.000000	O
H978	1.0	0.190425	0.712535	0.918899	Biso	1.000000	H
H979	1.0	0.168160	0.746199	0.916668	Biso	1.000000	H
H980	1.0	0.164002	0.715043	0.892865	Biso	1.000000	H
O1677	1.0	0.411260	0.291763	0.796257	Biso	1.000000	O
H981	1.0	0.421175	0.315473	0.799220	Biso	1.000000	H
H982	1.0	0.390794	0.295558	0.784732	Biso	1.000000	H
H983	1.0	0.425237	0.280160	0.780581	Biso	1.000000	H
O1678	1.0	0.485726	0.085043	0.676731	Biso	1.000000	O
H984	1.0	0.497685	0.064182	0.668696	Biso	1.000000	H
H985	1.0	0.497939	0.106398	0.670173	Biso	1.000000	H
H986	1.0	0.487923	0.083507	0.699556	Biso	1.000000	H
O1679	1.0	0.230078	0.785723	0.352460	Biso	1.000000	O
H987	1.0	0.217974	0.767819	0.364654	Biso	1.000000	H
H988	1.0	0.228861	0.778455	0.330425	Biso	1.000000	H
H989	1.0	0.253159	0.784674	0.358675	Biso	1.000000	H
O1680	1.0	0.079888	0.157975	0.327346	Biso	1.000000	O
H990	1.0	0.082152	0.136703	0.340671	Biso	1.000000	H
H991	1.0	0.058733	0.169227	0.331931	Biso	1.000000	H
H992	1.0	0.077985	0.147721	0.306283	Biso	1.000000	H
O1681	1.0	0.956112	0.763767	0.076800	Biso	1.000000	O
H993	1.0	0.964607	0.775944	0.095458	Biso	1.000000	H
H994	1.0	0.953144	0.782939	0.061341	Biso	1.000000	H
H995	1.0	0.933856	0.758248	0.083907	Biso	1.000000	H
O1682	1.0	0.154334	0.980258	0.740392	Biso	1.000000	O
H996	1.0	0.160212	0.005647	0.742445	Biso	1.000000	H
H997	1.0	0.130607	0.979896	0.743818	Biso	1.000000	H
H998	1.0	0.157310	0.976704	0.717822	Biso	1.000000	H
O1683	1.0	0.920713	0.272135	0.084878	Biso	1.000000	O
H999	1.0	0.899023	0.282787	0.088047	Biso	1.000000	H
H1000	1.0	0.937036	0.289531	0.092132	Biso	1.000000	H
H1001	1.0	0.921671	0.251752	0.099357	Biso	1.000000	H
O1684	1.0	0.452001	0.796815	0.776896	Biso	1.000000	O
H1002	1.0	0.457393	0.811430	0.758519	Biso	1.000000	H
H1003	1.0	0.463874	0.808805	0.793880	Biso	1.000000	H
H1004	1.0	0.463645	0.774046	0.774069	Biso	1.000000	H
O1685	1.0	0.351126	0.117294	0.009578	Biso	1.000000	O
H1005	1.0	0.337403	0.138320	0.013802	Biso	1.000000	H
H1006	1.0	0.372894	0.123963	0.017291	Biso	1.000000	H
H1007	1.0	0.354347	0.114960	0.986918	Biso	1.000000	H
O1686	1.0	0.500433	0.904519	0.028099	Biso	1.000000	O
H1008	1.0	0.502869	0.915420	0.048862	Biso	1.000000	H
H1009	1.0	0.515125	0.918696	0.014819	Biso	1.000000	H
H1010	1.0	0.511926	0.881515	0.029790	Biso	1.000000	H
O1687	1.0	0.705781	0.683209	0.826564	Biso	1.000000	O
H1011	1.0	0.719891	0.662891	0.832074	Biso	1.000000	H
H1012	1.0	0.712443	0.690047	0.805332	Biso	1.000000	H

H1013	1.0	0.711533	0.703429	0.840172	Biso	1.000000	H
O1688	1.0	0.077548	0.486986	0.670421	Biso	1.000000	O
H1014	1.0	0.085620	0.470809	0.654047	Biso	1.000000	H
H1015	1.0	0.057329	0.497341	0.661983	Biso	1.000000	H
H1016	1.0	0.071568	0.471407	0.688010	Biso	1.000000	H
Na1	1.0	0.082482	0.869766	0.761258	Biso	1.000000	Na
Na2	1.0	0.726822	0.529762	0.401937	Biso	1.000000	Na
Na3	1.0	0.371700	0.393140	0.704395	Biso	1.000000	Na
Na4	1.0	0.858583	0.341704	0.341084	Biso	1.000000	Na
Na5	1.0	0.051970	0.380126	0.264112	Biso	1.000000	Na
Na6	1.0	0.785562	0.717611	0.933170	Biso	1.000000	Na
Na7	1.0	0.600963	0.932294	0.696991	Biso	1.000000	Na
Na8	1.0	0.183078	0.671570	0.792179	Biso	1.000000	Na
Na9	1.0	0.245925	0.390264	0.194162	Biso	1.000000	Na
Na10	1.0	0.139660	0.569180	0.951818	Biso	1.000000	Na
Na11	1.0	0.458596	0.076457	0.157425	Biso	1.000000	Na
Na12	1.0	0.962695	0.227683	0.380799	Biso	1.000000	Na
Na13	1.0	0.680699	0.844461	0.536473	Biso	1.000000	Na
Na14	1.0	0.840731	0.758675	0.487875	Biso	1.000000	Na
Na15	1.0	0.820017	0.921371	0.806443	Biso	1.000000	Na
Na16	1.0	0.535383	0.245549	0.637044	Biso	1.000000	Na
Na17	1.0	0.667770	0.100449	0.219503	Biso	1.000000	Na
Na18	1.0	0.719630	0.015092	0.822182	Biso	1.000000	Na
Na19	1.0	0.233732	0.278168	0.596370	Biso	1.000000	Na
Na20	1.0	0.745886	0.147280	0.917729	Biso	1.000000	Na
Na21	1.0	0.753554	0.570672	0.250138	Biso	1.000000	Na
Na22	1.0	0.913011	0.500768	0.850892	Biso	1.000000	Na
Na23	1.0	0.883145	0.438088	0.552301	Biso	1.000000	Na
Na24	1.0	0.510803	0.727438	0.817845	Biso	1.000000	Na
Na25	1.0	0.785703	0.028707	0.452938	Biso	1.000000	Na
Na26	1.0	0.423172	0.586460	0.818607	Biso	1.000000	Na
Na27	1.0	0.799652	0.052353	0.718362	Biso	1.000000	Na
Na28	1.0	0.861895	0.785950	0.841836	Biso	1.000000	Na
H1017	1.0	0.516619	0.949816	0.837539	Biso	1.000000	H
H1018	1.0	0.419614	0.780974	0.699372	Biso	1.000000	H
H1019	1.0	0.617221	0.948819	0.825870	Biso	1.000000	H
H1020	1.0	0.649337	0.886569	0.734783	Biso	1.000000	H
H1021	1.0	0.628055	0.886288	0.634606	Biso	1.000000	H
H1022	1.0	0.529123	0.015643	0.673592	Biso	1.000000	H
H1023	1.0	0.757678	0.858706	0.646187	Biso	1.000000	H
O1689	1.0	0.383188	0.234435	0.210544	Biso	1.000000	O
H1024	1.0	0.400090	0.253950	0.212804	Biso	1.000000	H
H1025	1.0	0.362035	0.242453	0.220453	Biso	1.000000	H
O1690	1.0	0.344684	0.005921	0.106243	Biso	1.000000	O
H1026	1.0	0.333070	0.013228	0.086428	Biso	1.000000	H
H1027	1.0	0.368067	0.999499	0.101256	Biso	1.000000	H
O1691	1.0	0.384801	0.994731	0.333471	Biso	1.000000	O
H1028	1.0	0.384266	0.969380	0.341638	Biso	1.000000	H
H1029	1.0	0.398620	0.995733	0.313948	Biso	1.000000	H
O1692	1.0	0.893775	0.110184	0.702578	Biso	1.000000	O
H1030	1.0	0.911746	0.095235	0.693048	Biso	1.000000	H
H1031	1.0	0.896157	0.110261	0.726066	Biso	1.000000	H
O1693	1.0	0.981037	0.101280	0.891863	Biso	1.000000	O
H1032	1.0	0.000762	0.099128	0.877825	Biso	1.000000	H
H1033	1.0	0.980874	0.080804	0.907266	Biso	1.000000	H
O1694	1.0	0.567798	0.427109	0.788661	Biso	1.000000	O
H1034	1.0	0.555025	0.405214	0.795148	Biso	1.000000	H
H1035	1.0	0.591941	0.421706	0.789671	Biso	1.000000	H
O1695	1.0	0.174195	0.526413	0.923046	Biso	1.000000	O
H1036	1.0	0.180439	0.512460	0.942352	Biso	1.000000	H
H1037	1.0	0.159158	0.511466	0.909650	Biso	1.000000	H
O1696	1.0	0.445330	0.492838	0.975234	Biso	1.000000	O
H1038	1.0	0.453392	0.467677	0.979077	Biso	1.000000	H
H1039	1.0	0.422445	0.495414	0.983737	Biso	1.000000	H
O1697	1.0	0.141559	0.478985	0.885168	Biso	1.000000	O
H1040	1.0	0.119212	0.467704	0.883540	Biso	1.000000	H
H1041	1.0	0.158028	0.460201	0.891459	Biso	1.000000	H
O1698	1.0	0.834021	0.893602	0.718357	Biso	1.000000	O
H1042	1.0	0.853645	0.908355	0.724549	Biso	1.000000	H
H1043	1.0	0.820596	0.907252	0.702548	Biso	1.000000	H
O1699	1.0	0.312750	0.715513	0.959085	Biso	1.000000	O
H1044	1.0	0.307618	0.741496	0.963345	Biso	1.000000	H
H1045	1.0	0.318033	0.703029	0.979394	Biso	1.000000	H
O1700	1.0	0.275328	0.490889	0.680572	Biso	1.000000	O
H1046	1.0	0.255462	0.504057	0.688534	Biso	1.000000	H
H1047	1.0	0.291836	0.488500	0.697977	Biso	1.000000	H
O1701	1.0	0.287231	0.555671	0.394841	Biso	1.000000	O
H1048	1.0	0.279040	0.579121	0.403574	Biso	1.000000	H

H1049	1.0	0.301025	0.560417	0.375723	Biso	1.000000	H
O1702	1.0	0.238558	0.053483	0.881509	Biso	1.000000	O
H1050	1.0	0.227531	0.077620	0.882731	Biso	1.000000	H
H1051	1.0	0.242374	0.046991	0.858896	Biso	1.000000	H
O1703	1.0	0.879929	0.372674	0.008133	Biso	1.000000	O
H1052	1.0	0.862891	0.361000	0.994447	Biso	1.000000	H
H1053	1.0	0.868793	0.387402	0.024806	Biso	1.000000	H
O1704	1.0	0.419865	0.901708	0.437846	Biso	1.000000	O
H1054	1.0	0.436473	0.915312	0.450624	Biso	1.000000	H
H1055	1.0	0.431116	0.881739	0.426137	Biso	1.000000	H
O1705	1.0	0.601562	0.691782	0.414301	Biso	1.000000	O
H1056	1.0	0.612179	0.667402	0.413516	Biso	1.000000	H
H1057	1.0	0.615854	0.709716	0.403136	Biso	1.000000	H
O1706	1.0	0.378505	0.100380	0.287468	Biso	1.000000	O
H1058	1.0	0.384525	0.095812	0.310000	Biso	1.000000	H
H1059	1.0	0.389125	0.123299	0.280174	Biso	1.000000	H
O1707	1.0	0.669544	0.144308	0.270174	Biso	1.000000	O
H1060	1.0	0.657136	0.165568	0.261726	Biso	1.000000	H
H1061	1.0	0.654943	0.130155	0.284629	Biso	1.000000	H
O1708	1.0	0.110146	0.444129	0.824686	Biso	1.000000	O
H1062	1.0	0.097518	0.450995	0.805325	Biso	1.000000	H
H1063	1.0	0.096682	0.449770	0.843834	Biso	1.000000	H
O1709	1.0	0.437485	0.052713	0.781009	Biso	1.000000	O
H1064	1.0	0.424196	0.074856	0.776372	Biso	1.000000	H
H1065	1.0	0.425116	0.036942	0.796082	Biso	1.000000	H
O1710	1.0	0.637893	0.152769	0.203629	Biso	1.000000	O
H1066	1.0	0.628931	0.167545	0.221430	Biso	1.000000	H
H1067	1.0	0.653271	0.167879	0.190733	Biso	1.000000	H
O1711	1.0	0.850811	0.788328	0.785058	Biso	1.000000	O
H1068	1.0	0.864573	0.768068	0.776657	Biso	1.000000	H
H1069	1.0	0.846185	0.805986	0.767752	Biso	1.000000	H
O1712	1.0	0.827791	0.440818	0.862754	Biso	1.000000	O
H1070	1.0	0.832262	0.428772	0.842069	Biso	1.000000	H
H1071	1.0	0.838635	0.465090	0.863099	Biso	1.000000	H
O1713	1.0	0.268082	0.784117	0.300476	Biso	1.000000	O
H1072	1.0	0.264410	0.767989	0.281868	Biso	1.000000	H
H1073	1.0	0.291046	0.780706	0.308553	Biso	1.000000	H
O1714	1.0	0.645396	0.248864	0.661180	Biso	1.000000	O
H1074	1.0	0.667524	0.244946	0.671023	Biso	1.000000	H
H1075	1.0	0.645162	0.238091	0.639540	Biso	1.000000	H
O1715	1.0	0.525269	0.760254	0.911170	Biso	1.000000	O
H1076	1.0	0.519963	0.752842	0.933288	Biso	1.000000	H
H1077	1.0	0.520802	0.739628	0.896532	Biso	1.000000	H
O1716	1.0	0.705981	0.282725	0.561945	Biso	1.000000	O
H1078	1.0	0.708862	0.259193	0.573222	Biso	1.000000	H
H1079	1.0	0.722412	0.300602	0.570079	Biso	1.000000	H
O1717	1.0	0.402011	0.571226	0.397241	Biso	1.000000	O
H1080	1.0	0.381548	0.559325	0.405335	Biso	1.000000	H
H1081	1.0	0.407696	0.561053	0.376068	Biso	1.000000	H
O1718	1.0	0.223607	0.509606	0.454404	Biso	1.000000	O
H1082	1.0	0.233137	0.511469	0.476108	Biso	1.000000	H
H1083	1.0	0.241423	0.503060	0.439120	Biso	1.000000	H
O1719	1.0	0.064037	0.624181	0.828381	Biso	1.000000	O
H1084	1.0	0.063737	0.650850	0.824549	Biso	1.000000	H
H1085	1.0	0.086357	0.614367	0.823152	Biso	1.000000	H
O1720	1.0	0.232501	0.217722	0.574744	Biso	1.000000	O
H1086	1.0	0.256180	0.210247	0.575716	Biso	1.000000	H
H1087	1.0	0.222649	0.209344	0.554385	Biso	1.000000	H
O1721	1.0	0.268050	0.309092	0.558297	Biso	1.000000	O
H1088	1.0	0.288139	0.295253	0.551862	Biso	1.000000	H
H1089	1.0	0.270470	0.334915	0.551721	Biso	1.000000	H
O1722	1.0	0.932536	0.863560	0.943485	Biso	1.000000	O
H1090	1.0	0.913441	0.876382	0.933598	Biso	1.000000	H
H1091	1.0	0.931724	0.866806	0.966898	Biso	1.000000	H
O1723	1.0	0.087477	0.410960	0.110176	Biso	1.000000	O
H1092	1.0	0.095441	0.385426	0.108833	Biso	1.000000	H
H1093	1.0	0.063461	0.411868	0.104865	Biso	1.000000	H
O1724	1.0	0.367818	0.544871	0.209920	Biso	1.000000	O
H1094	1.0	0.384409	0.533018	0.223984	Biso	1.000000	H
H1095	1.0	0.346346	0.531694	0.211446	Biso	1.000000	H

O1725	1.0	0.307566	0.433550	0.986981	Biso	1.000000	O
H1096	1.0	0.322884	0.443305	0.970565	Biso	1.000000	H
H1097	1.0	0.320608	0.425080	0.005592	Biso	1.000000	H
O1726	1.0	0.252646	0.747764	0.916360	Biso	1.000000	O
H1098	1.0	0.275955	0.755589	0.912795	Biso	1.000000	H
H1099	1.0	0.246278	0.752797	0.938730	Biso	1.000000	H
O1727	1.0	0.035171	0.099303	0.853934	Biso	1.000000	O
H1100	1.0	0.031481	0.117264	0.836656	Biso	1.000000	H
H1101	1.0	0.057766	0.102666	0.862924	Biso	1.000000	H
O1728	1.0	0.644876	0.710926	0.669958	Biso	1.000000	O
H1102	1.0	0.664697	0.701547	0.658554	Biso	1.000000	H
H1103	1.0	0.649295	0.736079	0.677497	Biso	1.000000	H
O1729	1.0	0.300248	0.606693	0.940178	Biso	1.000000	O
H1104	1.0	0.320798	0.597847	0.929663	Biso	1.000000	H
H1105	1.0	0.304209	0.609096	0.963373	Biso	1.000000	H
O1730	1.0	0.368159	0.048431	0.606920	Biso	1.000000	O
H1106	1.0	0.358954	0.041459	0.585891	Biso	1.000000	H
H1107	1.0	0.355358	0.036114	0.623985	Biso	1.000000	H
O1731	1.0	0.475372	0.736677	0.292621	Biso	1.000000	O
H1108	1.0	0.455274	0.723654	0.300221	Biso	1.000000	H
H1109	1.0	0.468866	0.760754	0.283895	Biso	1.000000	H
O1732	1.0	0.189724	0.432772	0.907657	Biso	1.000000	O
H1110	1.0	0.177602	0.415422	0.921546	Biso	1.000000	H
H1111	1.0	0.211446	0.422096	0.901487	Biso	1.000000	H
O1733	1.0	0.773393	0.703494	0.828461	Biso	1.000000	O
H1112	1.0	0.784480	0.683287	0.816923	Biso	1.000000	H
H1113	1.0	0.782541	0.727119	0.821059	Biso	1.000000	H
O1734	1.0	0.425942	0.214630	0.399452	Biso	1.000000	O
H1114	1.0	0.440032	0.226500	0.383090	Biso	1.000000	H
H1115	1.0	0.437300	0.192481	0.407501	Biso	1.000000	H
O1735	1.0	0.266733	0.591803	0.081997	Biso	1.000000	O
H1116	1.0	0.288025	0.580008	0.076013	Biso	1.000000	H
H1117	1.0	0.269875	0.606069	0.101812	Biso	1.000000	H
O1736	1.0	0.544602	0.501492	0.386882	Biso	1.000000	O
H1118	1.0	0.537800	0.514988	0.406264	Biso	1.000000	H
H1119	1.0	0.534991	0.513504	0.367847	Biso	1.000000	H
O1737	1.0	0.832891	0.516993	0.931322	Biso	1.000000	O
H1120	1.0	0.826269	0.499981	0.948527	Biso	1.000000	H
H1121	1.0	0.834100	0.542135	0.939909	Biso	1.000000	H
O1738	1.0	0.278821	0.287969	0.333929	Biso	1.000000	O
H1122	1.0	0.269002	0.264041	0.328252	Biso	1.000000	H
H1123	1.0	0.261944	0.302999	0.345060	Biso	1.000000	H
O1739	1.0	0.217457	0.326847	0.885093	Biso	1.000000	O
H1124	1.0	0.195966	0.333231	0.874946	Biso	1.000000	H
H1125	1.0	0.226993	0.305094	0.874467	Biso	1.000000	H
O1740	1.0	0.252438	0.409241	0.798937	Biso	1.000000	O
H1126	1.0	0.250051	0.384872	0.788989	Biso	1.000000	H
H1127	1.0	0.270655	0.422788	0.788302	Biso	1.000000	H
O1741	1.0	0.335709	0.828901	0.285424	Biso	1.000000	O
H1128	1.0	0.322911	0.822312	0.266089	Biso	1.000000	H
H1129	1.0	0.334025	0.808922	0.301234	Biso	1.000000	H
O1742	1.0	0.268230	0.383842	0.537794	Biso	1.000000	O
H1130	1.0	0.244442	0.377469	0.534988	Biso	1.000000	H
H1131	1.0	0.270277	0.403681	0.553700	Biso	1.000000	H
O1743	1.0	0.064765	0.928596	0.781278	Biso	1.000000	O
H1132	1.0	0.071465	0.933375	0.803600	Biso	1.000000	H
H1133	1.0	0.040284	0.930969	0.779303	Biso	1.000000	H
O1744	1.0	0.900135	0.570742	0.906485	Biso	1.000000	O
H1134	1.0	0.905399	0.544676	0.910171	Biso	1.000000	H
H1135	1.0	0.915708	0.586159	0.918881	Biso	1.000000	H
O1745	1.0	0.714899	0.955658	0.482901	Biso	1.000000	O
H1136	1.0	0.727483	0.932526	0.484907	Biso	1.000000	H
H1137	1.0	0.705673	0.962550	0.503942	Biso	1.000000	H
O1746	1.0	0.856671	0.275679	0.985607	Biso	1.000000	O
H1138	1.0	0.854782	0.268980	0.008395	Biso	1.000000	H
H1139	1.0	0.847489	0.300480	0.982323	Biso	1.000000	H
O1747	1.0	0.557575	0.107352	0.289799	Biso	1.000000	O
H1140	1.0	0.536900	0.109810	0.302482	Biso	1.000000	H
H1141	1.0	0.557765	0.083320	0.278999	Biso	1.000000	H
O1748	1.0	0.417665	0.968502	0.567339	Biso	1.000000	O
H1142	1.0	0.408606	0.966306	0.589204	Biso	1.000000	H
H1143	1.0	0.427787	0.992943	0.564562	Biso	1.000000	H
O1749	1.0	0.117198	0.536554	0.732638	Biso	1.000000	O
H1144	1.0	0.122229	0.510177	0.730816	Biso	1.000000	H

H1145	1.0	0.097686	0.542535	0.719183	Biso	1.000000	H
O1750	1.0	0.321508	0.360870	0.696939	Biso	1.000000	O
H1146	1.0	0.324110	0.334571	0.701778	Biso	1.000000	H
H1147	1.0	0.303097	0.371063	0.709872	Biso	1.000000	H
O1751	1.0	0.321789	0.426955	0.829018	Biso	1.000000	O
H1148	1.0	0.336353	0.447238	0.836029	Biso	1.000000	H
H1149	1.0	0.314316	0.431439	0.806875	Biso	1.000000	H
O1752	1.0	0.612552	0.778614	0.491250	Biso	1.000000	O
H1150	1.0	0.594286	0.789777	0.503758	Biso	1.000000	H
H1151	1.0	0.632675	0.794148	0.492748	Biso	1.000000	H
O1753	1.0	0.399034	0.850778	0.275705	Biso	1.000000	O
H1152	1.0	0.375262	0.843648	0.276582	Biso	1.000000	H
H1153	1.0	0.408051	0.852362	0.297626	Biso	1.000000	H
O1754	1.0	0.614789	0.995475	0.695424	Biso	1.000000	O
H1154	1.0	0.638308	0.992607	0.688777	Biso	1.000000	H
H1155	1.0	0.605158	0.017292	0.684978	Biso	1.000000	H
O1755	1.0	0.833463	0.044558	0.336256	Biso	1.000000	O
H1156	1.0	0.814043	0.035888	0.323835	Biso	1.000000	H
H1157	1.0	0.833530	0.071582	0.336723	Biso	1.000000	H
O1756	1.0	0.740599	0.628154	0.835453	Biso	1.000000	O
H1158	1.0	0.762976	0.628213	0.825533	Biso	1.000000	H
H1159	1.0	0.732992	0.602655	0.838339	Biso	1.000000	H
O1757	1.0	0.748895	0.887956	0.875227	Biso	1.000000	O
H1160	1.0	0.732593	0.888434	0.857527	Biso	1.000000	H
H1161	1.0	0.737227	0.892429	0.895645	Biso	1.000000	H
O1758	1.0	0.693701	0.054029	0.406977	Biso	1.000000	O
H1162	1.0	0.676644	0.055305	0.423985	Biso	1.000000	H
H1163	1.0	0.697657	0.078778	0.398282	Biso	1.000000	H
O1759	1.0	0.357881	0.335636	0.371868	Biso	1.000000	O
H1164	1.0	0.347025	0.349486	0.389265	Biso	1.000000	H
H1165	1.0	0.350167	0.309970	0.372366	Biso	1.000000	H
O1760	1.0	0.834650	0.839113	0.378222	Biso	1.000000	O
H1166	1.0	0.843642	0.817314	0.367238	Biso	1.000000	H
H1167	1.0	0.825196	0.831817	0.399065	Biso	1.000000	H
O1761	1.0	0.397130	0.635041	0.304216	Biso	1.000000	O
H1168	1.0	0.376978	0.636352	0.317770	Biso	1.000000	H
H1169	1.0	0.401412	0.609315	0.298246	Biso	1.000000	H
O1762	1.0	0.101989	0.189148	0.634861	Biso	1.000000	O
H1170	1.0	0.085270	0.180377	0.619294	Biso	1.000000	H
H1171	1.0	0.093455	0.185450	0.656763	Biso	1.000000	H
O1763	1.0	0.477037	0.590222	0.327652	Biso	1.000000	O
H1172	1.0	0.474595	0.598366	0.305276	Biso	1.000000	H
H1173	1.0	0.474309	0.611391	0.342091	Biso	1.000000	H
O1764	1.0	0.283899	0.889643	0.249911	Biso	1.000000	O
H1174	1.0	0.279627	0.877861	0.270749	Biso	1.000000	H
H1175	1.0	0.278779	0.916025	0.251412	Biso	1.000000	H
O1765	1.0	0.457950	0.780325	0.120196	Biso	1.000000	O
H1176	1.0	0.441480	0.760402	0.122660	Biso	1.000000	H
H1177	1.0	0.476810	0.776217	0.134970	Biso	1.000000	H
O1766	1.0	0.817558	0.127694	0.481979	Biso	1.000000	O
H1178	1.0	0.813713	0.101082	0.483850	Biso	1.000000	H
H1179	1.0	0.796441	0.140879	0.486000	Biso	1.000000	H
O1767	1.0	0.464382	0.347242	0.885426	Biso	1.000000	O
H1180	1.0	0.482803	0.337869	0.872037	Biso	1.000000	H
H1181	1.0	0.473542	0.357062	0.905586	Biso	1.000000	H
O1768	1.0	0.215778	0.101069	0.972871	Biso	1.000000	O
H1182	1.0	0.192833	0.091241	0.971735	Biso	1.000000	H
H1183	1.0	0.231775	0.080654	0.975087	Biso	1.000000	H
O1769	1.0	0.889215	0.788258	0.206176	Biso	1.000000	O
H1184	1.0	0.893027	0.809924	0.219804	Biso	1.000000	H
H1185	1.0	0.887138	0.766262	0.219743	Biso	1.000000	H
O1770	1.0	0.508544	0.561915	0.871579	Biso	1.000000	O
H1186	1.0	0.494301	0.541176	0.865001	Biso	1.000000	H
H1187	1.0	0.526408	0.565561	0.855626	Biso	1.000000	H
O1771	1.0	0.220070	0.117131	0.140483	Biso	1.000000	O
H1188	1.0	0.238737	0.110598	0.126154	Biso	1.000000	H
H1189	1.0	0.201712	0.099361	0.137718	Biso	1.000000	H
O1772	1.0	0.262447	0.088166	0.583885	Biso	1.000000	O
H1190	1.0	0.239292	0.097343	0.582526	Biso	1.000000	H
H1191	1.0	0.262402	0.063294	0.593121	Biso	1.000000	H
O1773	1.0	0.826757	0.046111	0.414389	Biso	1.000000	O
H1192	1.0	0.831695	0.022219	0.404419	Biso	1.000000	H
H1193	1.0	0.844458	0.063786	0.408747	Biso	1.000000	H
O1774	1.0	0.756948	0.083297	0.458108	Biso	1.000000	O
H1194	1.0	0.749798	0.085442	0.480612	Biso	1.000000	H
H1195	1.0	0.747973	0.104106	0.445739	Biso	1.000000	H
O1775	1.0	0.543154	0.827898	0.144308	Biso	1.000000	O
H1196	1.0	0.553708	0.836112	0.164393	Biso	1.000000	H
H1197	1.0	0.533237	0.803418	0.147471	Biso	1.000000	H

O1776	1.0	0.957128	0.534854	0.513100	Biso	1.000000	O
H1198	1.0	0.951028	0.526913	0.534887	Biso	1.000000	H
H1199	1.0	0.948534	0.516990	0.497416	Biso	1.000000	H
O1777	1.0	0.992699	0.447965	0.987499	Biso	1.000000	O
H1200	1.0	0.969646	0.438488	0.988881	Biso	1.000000	H
H1201	1.0	0.006844	0.430142	0.976033	Biso	1.000000	H
O1778	1.0	0.428283	0.544352	0.486974	Biso	1.000000	O
H1202	1.0	0.431980	0.568121	0.497637	Biso	1.000000	H
H1203	1.0	0.440447	0.543916	0.466450	Biso	1.000000	H
O1779	1.0	0.082834	0.648158	0.903419	Biso	1.000000	O
H1204	1.0	0.078628	0.654339	0.926035	Biso	1.000000	H
H1205	1.0	0.089039	0.622072	0.901702	Biso	1.000000	H
O1780	1.0	0.781737	0.133593	0.872419	Biso	1.000000	O
H1206	1.0	0.774311	0.149851	0.854960	Biso	1.000000	H
H1207	1.0	0.785301	0.108662	0.863969	Biso	1.000000	H
O1781	1.0	0.911479	0.271362	0.948010	Biso	1.000000	O
H1208	1.0	0.890672	0.270522	0.960659	Biso	1.000000	H
H1209	1.0	0.930900	0.270228	0.962520	Biso	1.000000	H
O1782	1.0	0.108364	0.190961	0.543901	Biso	1.000000	O
H1210	1.0	0.109109	0.189958	0.520330	Biso	1.000000	H
H1211	1.0	0.108231	0.216761	0.550933	Biso	1.000000	H
O1783	1.0	0.071084	0.426853	0.174284	Biso	1.000000	O
H1212	1.0	0.079395	0.419204	0.153096	Biso	1.000000	H
H1213	1.0	0.066461	0.453400	0.174077	Biso	1.000000	H
O1784	1.0	0.690214	0.685601	0.126043	Biso	1.000000	O
H1214	1.0	0.689908	0.673967	0.104746	Biso	1.000000	H
H1215	1.0	0.704595	0.707548	0.125431	Biso	1.000000	H
O1785	1.0	0.670663	0.190257	0.441291	Biso	1.000000	O
H1216	1.0	0.677357	0.214889	0.448594	Biso	1.000000	H
H1217	1.0	0.662411	0.175915	0.459668	Biso	1.000000	H
O1786	1.0	0.351299	0.412109	0.031731	Biso	1.000000	O
H1218	1.0	0.373927	0.407316	0.023329	Biso	1.000000	H
H1219	1.0	0.344245	0.391475	0.045397	Biso	1.000000	H
O1787	1.0	0.876700	0.166995	0.663273	Biso	1.000000	O
H1220	1.0	0.896283	0.183299	0.661490	Biso	1.000000	H
H1221	1.0	0.882287	0.145990	0.677129	Biso	1.000000	H
O1788	1.0	0.559259	0.383537	0.356174	Biso	1.000000	O
H1222	1.0	0.547461	0.377988	0.336025	Biso	1.000000	H
H1223	1.0	0.545880	0.400943	0.368902	Biso	1.000000	H
H1224	1.0	0.980393	0.977939	0.659465	Biso	1.000000	H
H1225	1.0	0.935822	0.765232	0.709018	Biso	1.000000	H
O1789	1.0	0.438424	0.939567	0.054278	Biso	1.000000	O
H1226	1.0	0.423772	0.928558	0.037909	Biso	1.000000	H
H1227	1.0	0.445098	0.920626	0.069854	Biso	1.000000	H
O1790	1.0	0.354678	0.462393	0.408549	Biso	1.000000	O
H1228	1.0	0.335179	0.450508	0.418605	Biso	1.000000	H
H1229	1.0	0.354510	0.457457	0.385351	Biso	1.000000	H
O1791	1.0	0.963848	0.317354	0.105019	Biso	1.000000	O
H1230	1.0	0.987144	0.319065	0.097416	Biso	1.000000	H
H1231	1.0	0.958085	0.339699	0.117097	Biso	1.000000	H
O1792	1.0	0.334745	0.739785	0.165572	Biso	1.000000	O
H1232	1.0	0.348872	0.718078	0.161694	Biso	1.000000	H
H1233	1.0	0.313685	0.737377	0.153473	Biso	1.000000	H
O1793	1.0	0.327348	0.067700	0.703260	Biso	1.000000	O
H1234	1.0	0.346554	0.084657	0.703338	Biso	1.000000	H
H1235	1.0	0.329654	0.050314	0.685327	Biso	1.000000	H
O1794	1.0	0.535257	0.369892	0.297380	Biso	1.000000	O
H1236	1.0	0.512725	0.369557	0.287793	Biso	1.000000	H
H1237	1.0	0.548087	0.348548	0.289704	Biso	1.000000	H
O1795	1.0	0.361339	0.541851	0.482484	Biso	1.000000	O
H1238	1.0	0.355839	0.538178	0.459706	Biso	1.000000	H
H1239	1.0	0.385817	0.541192	0.485303	Biso	1.000000	H
O1796	1.0	0.625655	0.930918	0.643518	Biso	1.000000	O
H1240	1.0	0.609883	0.945116	0.630271	Biso	1.000000	H
H1241	1.0	0.648577	0.940048	0.640003	Biso	1.000000	H
O1797	1.0	0.881823	0.106184	0.931859	Biso	1.000000	O
H1242	1.0	0.860529	0.095382	0.939120	Biso	1.000000	H
H1243	1.0	0.879588	0.133073	0.930782	Biso	1.000000	H
O1798	1.0	0.298988	0.609745	0.535482	Biso	1.000000	O
H1244	1.0	0.297925	0.589041	0.550619	Biso	1.000000	H
H1245	1.0	0.286286	0.630651	0.544198	Biso	1.000000	H
O1799	1.0	0.516365	0.203934	0.116286	Biso	1.000000	O
H1246	1.0	0.523564	0.186645	0.099507	Biso	1.000000	H
H1247	1.0	0.496178	0.217092	0.109085	Biso	1.000000	H
O1800	1.0	0.101388	0.094425	0.875225	Biso	1.000000	O
H1248	1.0	0.120286	0.102286	0.888746	Biso	1.000000	H
H1249	1.0	0.108936	0.092150	0.852848	Biso	1.000000	H
O1801	1.0	0.386387	0.217428	0.295247	Biso	1.000000	O
H1250	1.0	0.397219	0.241472	0.292286	Biso	1.000000	H

H1251	1.0	0.380902	0.213952	0.318051	Biso	1.000000	H
O1802	1.0	0.196919	0.736290	0.380355	Biso	1.000000	O
H1252	1.0	0.183615	0.721470	0.365280	Biso	1.000000	H
H1253	1.0	0.189864	0.730828	0.402458	Biso	1.000000	H
O1803	1.0	0.407178	0.981018	0.100474	Biso	1.000000	O
H1254	1.0	0.418792	0.968178	0.082933	Biso	1.000000	H
H1255	1.0	0.405538	0.964655	0.119189	Biso	1.000000	H
O1804	1.0	0.262558	0.433323	0.488770	Biso	1.000000	O
H1256	1.0	0.266499	0.414698	0.505448	Biso	1.000000	H
H1257	1.0	0.241636	0.427618	0.477309	Biso	1.000000	H
O1805	1.0	0.036458	0.398170	0.029244	Biso	1.000000	O
H1258	1.0	0.052637	0.414674	0.018776	Biso	1.000000	H
H1259	1.0	0.047667	0.375014	0.035007	Biso	1.000000	H
O1806	1.0	0.920337	0.012805	0.090602	Biso	1.000000	O
H1260	1.0	0.909355	0.007894	0.111290	Biso	1.000000	H
H1261	1.0	0.922953	0.989636	0.078711	Biso	1.000000	H
O1807	1.0	0.812366	0.749750	0.974816	Biso	1.000000	O
H1262	1.0	0.827519	0.770889	0.972353	Biso	1.000000	H
H1263	1.0	0.799635	0.752264	0.994905	Biso	1.000000	H
O1808	1.0	0.911682	0.222094	0.124915	Biso	1.000000	O
H1264	1.0	0.888013	0.226480	0.130333	Biso	1.000000	H
H1265	1.0	0.920958	0.203291	0.139358	Biso	1.000000	H
O1809	1.0	0.728057	0.249583	0.385625	Biso	1.000000	O
H1266	1.0	0.708090	0.240611	0.374201	Biso	1.000000	H
H1267	1.0	0.748187	0.237703	0.376776	Biso	1.000000	H
O1810	1.0	0.203872	0.263107	0.643141	Biso	1.000000	O
H1268	1.0	0.209201	0.241344	0.656173	Biso	1.000000	H
H1269	1.0	0.183942	0.275387	0.651990	Biso	1.000000	H
O1811	1.0	0.068632	0.247824	0.878639	Biso	1.000000	O
H1270	1.0	0.069906	0.220948	0.880827	Biso	1.000000	H
H1271	1.0	0.046483	0.254720	0.870182	Biso	1.000000	H
O1812	1.0	0.390343	0.813735	0.047998	Biso	1.000000	O
H1272	1.0	0.381765	0.801845	0.028462	Biso	1.000000	H
H1273	1.0	0.378320	0.836980	0.051549	Biso	1.000000	H
O1813	1.0	0.733377	0.052102	0.309872	Biso	1.000000	O
H1274	1.0	0.741302	0.074726	0.320323	Biso	1.000000	H
H1275	1.0	0.752509	0.035628	0.306012	Biso	1.000000	H
O1814	1.0	0.645830	0.064944	0.454144	Biso	1.000000	O
H1276	1.0	0.630968	0.085608	0.448741	Biso	1.000000	H
H1277	1.0	0.659866	0.071633	0.472649	Biso	1.000000	H
O1815	1.0	0.934212	0.175413	0.806059	Biso	1.000000	O
H1278	1.0	0.928092	0.183938	0.784444	Biso	1.000000	H
H1279	1.0	0.943245	0.196115	0.818527	Biso	1.000000	H
O1816	1.0	0.921682	0.494243	0.411548	Biso	1.000000	O
H1280	1.0	0.942772	0.490346	0.399799	Biso	1.000000	H
H1281	1.0	0.925591	0.490756	0.434646	Biso	1.000000	H
O1817	1.0	0.747746	0.008147	0.414233	Biso	1.000000	O
H1282	1.0	0.744301	0.988462	0.398404	Biso	1.000000	H
H1283	1.0	0.728477	0.025010	0.413815	Biso	1.000000	H
O1818	1.0	0.366163	0.159178	0.913960	Biso	1.000000	O
H1284	1.0	0.368805	0.155321	0.890742	Biso	1.000000	H
H1285	1.0	0.378238	0.181570	0.920376	Biso	1.000000	H
O1819	1.0	0.320936	0.404089	0.265063	Biso	1.000000	O
H1286	1.0	0.302919	0.387812	0.257460	Biso	1.000000	H
H1287	1.0	0.328110	0.396313	0.286594	Biso	1.000000	H
O1820	1.0	0.919521	0.505198	0.793713	Biso	1.000000	O
H1288	1.0	0.938134	0.522907	0.792913	Biso	1.000000	H
H1289	1.0	0.898239	0.518077	0.789742	Biso	1.000000	H
O1821	1.0	0.109092	0.068637	0.572762	Biso	1.000000	O
H1290	1.0	0.121891	0.046305	0.567594	Biso	1.000000	H
H1291	1.0	0.109718	0.072896	0.596056	Biso	1.000000	H
O1822	1.0	0.635992	0.369406	0.456577	Biso	1.000000	O
H1292	1.0	0.657661	0.359992	0.464288	Biso	1.000000	H
H1293	1.0	0.627656	0.353554	0.439208	Biso	1.000000	H
O1823	1.0	0.862075	0.697961	0.977328	Biso	1.000000	O
H1294	1.0	0.850012	0.720424	0.971077	Biso	1.000000	H
H1295	1.0	0.885843	0.700274	0.971367	Biso	1.000000	H
O1824	1.0	0.807656	0.369063	0.146954	Biso	1.000000	O
H1296	1.0	0.808990	0.342088	0.147716	Biso	1.000000	H
H1297	1.0	0.784312	0.376831	0.150421	Biso	1.000000	H

O1825	1.0	0.776180	0.562720	0.387151	Biso	1.000000	O
H1298	1.0	0.791457	0.558706	0.368961	Biso	1.000000	H
H1299	1.0	0.778913	0.587946	0.395209	Biso	1.000000	H
O1826	1.0	0.089189	0.178907	0.698008	Biso	1.000000	O
H1300	1.0	0.079390	0.154536	0.702031	Biso	1.000000	H
H1301	1.0	0.071900	0.197885	0.700952	Biso	1.000000	H
O1827	1.0	0.301232	0.352910	0.635368	Biso	1.000000	O
H1302	1.0	0.314062	0.332920	0.625292	Biso	1.000000	H
H1303	1.0	0.307317	0.354694	0.658181	Biso	1.000000	H
O1828	1.0	0.105718	0.075457	0.242530	Biso	1.000000	O
H1304	1.0	0.099116	0.049644	0.245543	Biso	1.000000	H
H1305	1.0	0.128738	0.076530	0.234114	Biso	1.000000	H
O1829	1.0	0.267648	0.384010	0.958336	Biso	1.000000	O
H1306	1.0	0.247543	0.379052	0.971300	Biso	1.000000	H
H1307	1.0	0.282575	0.400786	0.970098	Biso	1.000000	H
O1830	1.0	0.730758	0.910055	0.596111	Biso	1.000000	O
H1308	1.0	0.745761	0.912933	0.577552	Biso	1.000000	H
H1309	1.0	0.744079	0.903984	0.615250	Biso	1.000000	H
O1831	1.0	0.421668	0.316573	0.348731	Biso	1.000000	O
H1310	1.0	0.437793	0.336720	0.351789	Biso	1.000000	H
H1311	1.0	0.399878	0.323600	0.357922	Biso	1.000000	H
O1832	1.0	0.809579	0.815468	0.434439	Biso	1.000000	O
H1312	1.0	0.797758	0.792193	0.438445	Biso	1.000000	H
H1313	1.0	0.824508	0.820928	0.452606	Biso	1.000000	H
O1833	1.0	0.718801	0.522710	0.965276	Biso	1.000000	O
H1314	1.0	0.725815	0.532910	0.986071	Biso	1.000000	H
H1315	1.0	0.729665	0.536494	0.947843	Biso	1.000000	H
O1834	1.0	0.466037	0.480102	0.442407	Biso	1.000000	O
H1316	1.0	0.465231	0.506038	0.435814	Biso	1.000000	H
H1317	1.0	0.483038	0.476975	0.459284	Biso	1.000000	H
O1835	1.0	0.214700	0.056214	0.267498	Biso	1.000000	O
H1318	1.0	0.229934	0.042584	0.253260	Biso	1.000000	H
H1319	1.0	0.227413	0.075100	0.279204	Biso	1.000000	H
O1836	1.0	0.249055	0.161587	0.230842	Biso	1.000000	O
H1320	1.0	0.243496	0.186453	0.238408	Biso	1.000000	H
H1321	1.0	0.228807	0.149852	0.222091	Biso	1.000000	H
O1837	1.0	0.368430	0.229791	0.147546	Biso	1.000000	O
H1322	1.0	0.374699	0.229161	0.170361	Biso	1.000000	H
H1323	1.0	0.357097	0.253161	0.142754	Biso	1.000000	H
O1838	1.0	0.456596	0.436140	0.655239	Biso	1.000000	O
H1324	1.0	0.446240	0.454930	0.641473	Biso	1.000000	H
H1325	1.0	0.471120	0.420231	0.642169	Biso	1.000000	H
O1839	1.0	0.604613	0.615559	0.429958	Biso	1.000000	O
H1326	1.0	0.594678	0.629065	0.448052	Biso	1.000000	H
H1327	1.0	0.610811	0.590662	0.436978	Biso	1.000000	H
O1840	1.0	0.994595	0.990513	0.900204	Biso	1.000000	O
H1328	1.0	0.986024	0.011481	0.912633	Biso	1.000000	H
H1329	1.0	0.011990	0.999078	0.885239	Biso	1.000000	H
O1841	1.0	0.306281	0.351283	0.826840	Biso	1.000000	O
H1330	1.0	0.306584	0.378188	0.829061	Biso	1.000000	H
H1331	1.0	0.318292	0.340104	0.844991	Biso	1.000000	H
O1842	1.0	0.203254	0.811924	0.260475	Biso	1.000000	O
H1332	1.0	0.205810	0.791042	0.275254	Biso	1.000000	H
H1333	1.0	0.188564	0.830515	0.270263	Biso	1.000000	H
O1843	1.0	0.548388	0.777455	0.820788	Biso	1.000000	O
H1334	1.0	0.549599	0.797326	0.804836	Biso	1.000000	H
H1335	1.0	0.554773	0.787055	0.841982	Biso	1.000000	H
O1844	1.0	0.406697	0.218293	0.095162	Biso	1.000000	O
H1336	1.0	0.395041	0.222387	0.115648	Biso	1.000000	H
H1337	1.0	0.392136	0.226002	0.077346	Biso	1.000000	H
O1845	1.0	0.149060	0.764135	0.769057	Biso	1.000000	O
H1338	1.0	0.137419	0.752142	0.787033	Biso	1.000000	H
H1339	1.0	0.161598	0.745618	0.756749	Biso	1.000000	H
O1846	1.0	0.785027	0.754513	0.462431	Biso	1.000000	O
H1340	1.0	0.766934	0.753494	0.478444	Biso	1.000000	H
H1341	1.0	0.783893	0.732647	0.448606	Biso	1.000000	H
O1847	1.0	0.250174	0.147615	0.933018	Biso	1.000000	O
H1342	1.0	0.237537	0.129996	0.946210	Biso	1.000000	H
H1343	1.0	0.235391	0.168035	0.926775	Biso	1.000000	H
O1848	1.0	0.153304	0.928280	0.115342	Biso	1.000000	O
H1344	1.0	0.176506	0.920734	0.110804	Biso	1.000000	H
H1345	1.0	0.149254	0.952800	0.106200	Biso	1.000000	H
O1849	1.0	0.555412	0.719886	0.243002	Biso	1.000000	O
H1346	1.0	0.562939	0.698150	0.230968	Biso	1.000000	H
H1347	1.0	0.547490	0.712291	0.264343	Biso	1.000000	H
O1850	1.0	0.742914	0.394070	0.141127	Biso	1.000000	O
H1348	1.0	0.728242	0.414430	0.147748	Biso	1.000000	H

H1349	1.0	0.733774	0.382496	0.121679	Biso	1.000000	H
O1851	1.0	0.232223	0.013798	0.544710	Biso	1.000000	O
H1350	1.0	0.247585	0.993744	0.538858	Biso	1.000000	H
H1351	1.0	0.235421	0.020180	0.567436	Biso	1.000000	H
O1852	1.0	0.271996	0.957673	0.528171	Biso	1.000000	O
H1352	1.0	0.290536	0.948473	0.541501	Biso	1.000000	H
H1353	1.0	0.280986	0.967276	0.507859	Biso	1.000000	H
O1853	1.0	0.436064	0.707729	0.066298	Biso	1.000000	O
H1354	1.0	0.426078	0.714573	0.087029	Biso	1.000000	H
H1355	1.0	0.460582	0.710329	0.067504	Biso	1.000000	H
O1854	1.0	0.140625	0.461987	0.007191	Biso	1.000000	O
H1356	1.0	0.137397	0.484645	0.019681	Biso	1.000000	H
H1357	1.0	0.157225	0.466393	0.990168	Biso	1.000000	H
O1855	1.0	0.377671	0.863071	0.914820	Biso	1.000000	O
H1358	1.0	0.378811	0.858100	0.937989	Biso	1.000000	H
H1359	1.0	0.391628	0.845153	0.903251	Biso	1.000000	H
O1856	1.0	0.526341	0.907038	0.276705	Biso	1.000000	O
H1360	1.0	0.507674	0.924539	0.278817	Biso	1.000000	H
H1361	1.0	0.518555	0.882245	0.282425	Biso	1.000000	H
O1857	1.0	0.321431	0.062628	0.952573	Biso	1.000000	O
H1362	1.0	0.341791	0.077821	0.951345	Biso	1.000000	H
H1363	1.0	0.314676	0.055167	0.930834	Biso	1.000000	H
O1858	1.0	0.223102	0.295007	0.943962	Biso	1.000000	O
H1364	1.0	0.220682	0.310200	0.924583	Biso	1.000000	H
H1365	1.0	0.240952	0.276760	0.940594	Biso	1.000000	H
O1859	1.0	0.025337	0.190088	0.338075	Biso	1.000000	O
H1366	1.0	0.002565	0.179706	0.338051	Biso	1.000000	H
H1367	1.0	0.024112	0.216758	0.341721	Biso	1.000000	H
O1860	1.0	0.153492	0.875958	0.891608	Biso	1.000000	O
H1368	1.0	0.165499	0.899408	0.889218	Biso	1.000000	H
H1369	1.0	0.167127	0.858908	0.904457	Biso	1.000000	H
O1861	1.0	0.342062	0.036401	0.548821	Biso	1.000000	O
H1370	1.0	0.350879	0.022377	0.530498	Biso	1.000000	H
H1371	1.0	0.324645	0.053491	0.541308	Biso	1.000000	H
O1862	1.0	0.534061	0.850754	0.657528	Biso	1.000000	O
H1372	1.0	0.524627	0.873693	0.648906	Biso	1.000000	H
H1373	1.0	0.547234	0.838375	0.640760	Biso	1.000000	H
O1863	1.0	0.302754	0.041121	0.894573	Biso	1.000000	O
H1374	1.0	0.278547	0.044800	0.891386	Biso	1.000000	H
H1375	1.0	0.307507	0.014692	0.896510	Biso	1.000000	H
O1864	1.0	0.219825	0.917409	0.290455	Biso	1.000000	O
H1376	1.0	0.231524	0.894527	0.296146	Biso	1.000000	H
H1377	1.0	0.235796	0.934645	0.280610	Biso	1.000000	H
O1865	1.0	0.880425	0.152486	0.480719	Biso	1.000000	O
H1378	1.0	0.857510	0.142768	0.482757	Biso	1.000000	H
H1379	1.0	0.896651	0.133020	0.485912	Biso	1.000000	H
O1866	1.0	0.588118	0.205307	0.071078	Biso	1.000000	O
H1380	1.0	0.603609	0.199642	0.053395	Biso	1.000000	H
H1381	1.0	0.592446	0.230350	0.078929	Biso	1.000000	H
O1867	1.0	0.421162	0.719292	0.848414	Biso	1.000000	O
H1382	1.0	0.401311	0.728992	0.837265	Biso	1.000000	H
H1383	1.0	0.416797	0.718462	0.871626	Biso	1.000000	H
O1868	1.0	0.934251	0.208604	0.661690	Biso	1.000000	O
H1384	1.0	0.950721	0.203005	0.678559	Biso	1.000000	H
H1385	1.0	0.944908	0.206077	0.640524	Biso	1.000000	H
O1869	1.0	0.323698	0.554639	0.067511	Biso	1.000000	O
H1386	1.0	0.311120	0.542411	0.050248	Biso	1.000000	H
H1387	1.0	0.330485	0.536340	0.083616	Biso	1.000000	H
O1870	1.0	0.529644	0.862584	0.880985	Biso	1.000000	O
H1388	1.0	0.543751	0.841042	0.876417	Biso	1.000000	H
H1389	1.0	0.539906	0.877150	0.898268	Biso	1.000000	H
O1871	1.0	0.347935	0.306035	0.143644	Biso	1.000000	O
H1390	1.0	0.330382	0.300775	0.159570	Biso	1.000000	H
H1391	1.0	0.344642	0.330890	0.134925	Biso	1.000000	H
O1872	1.0	0.436481	0.241452	0.849481	Biso	1.000000	O
H1392	1.0	0.444639	0.228535	0.868682	Biso	1.000000	H
H1393	1.0	0.455676	0.251134	0.837312	Biso	1.000000	H
O1873	1.0	0.932112	0.952842	0.055701	Biso	1.000000	O
H1394	1.0	0.915153	0.955049	0.038676	Biso	1.000000	H
H1395	1.0	0.936981	0.926720	0.059581	Biso	1.000000	H
O1874	1.0	0.138007	0.132056	0.147867	Biso	1.000000	O

H1396	1.0	0.144995	0.148492	0.130368	Biso	1.000000	H
H1397	1.0	0.149783	0.108420	0.145857	Biso	1.000000	H
O1875	1.0	0.635664	0.529624	0.363257	Biso	1.000000	O
H1398	1.0	0.614515	0.541589	0.357068	Biso	1.000000	H
H1399	1.0	0.648452	0.523253	0.343863	Biso	1.000000	H
O1876	1.0	0.308399	0.236113	0.670905	Biso	1.000000	O
H1400	1.0	0.331053	0.225894	0.668163	Biso	1.000000	H
H1401	1.0	0.293641	0.217336	0.680324	Biso	1.000000	H
O1877	1.0	0.006198	0.851767	0.686301	Biso	1.000000	O
H1402	1.0	0.994335	0.867798	0.671065	Biso	1.000000	H
H1403	1.0	0.018389	0.832607	0.674428	Biso	1.000000	H
O1878	1.0	0.029263	0.331104	0.302027	Biso	1.000000	O
H1404	1.0	0.009298	0.318981	0.293086	Biso	1.000000	H
H1405	1.0	0.026569	0.333952	0.325349	Biso	1.000000	H
O1879	1.0	0.395471	0.707559	0.640585	Biso	1.000000	O
H1406	1.0	0.377020	0.689950	0.637619	Biso	1.000000	H
H1407	1.0	0.406394	0.703045	0.661372	Biso	1.000000	H
O1880	1.0	0.454675	0.077915	0.424769	Biso	1.000000	O
H1408	1.0	0.473737	0.077395	0.439734	Biso	1.000000	H
H1409	1.0	0.438143	0.059236	0.431149	Biso	1.000000	H
O1881	1.0	0.894455	0.267076	0.664368	Biso	1.000000	O
H1410	1.0	0.909066	0.245369	0.662853	Biso	1.000000	H
H1411	1.0	0.907522	0.289304	0.659470	Biso	1.000000	H
O1882	1.0	0.372857	0.678335	0.156032	Biso	1.000000	O
H1412	1.0	0.377656	0.660880	0.173454	Biso	1.000000	H
H1413	1.0	0.362873	0.665113	0.137802	Biso	1.000000	H
O1883	1.0	0.471574	0.646198	0.370591	Biso	1.000000	O
H1414	1.0	0.483871	0.630532	0.385801	Biso	1.000000	H
H1415	1.0	0.485257	0.667835	0.365249	Biso	1.000000	H
O1884	1.0	0.555560	0.063326	0.202085	Biso	1.000000	O
H1416	1.0	0.551520	0.052487	0.223352	Biso	1.000000	H
H1417	1.0	0.561921	0.043849	0.186898	Biso	1.000000	H
O1885	1.0	0.497599	0.455422	0.042332	Biso	1.000000	O
H1418	1.0	0.519613	0.443294	0.043395	Biso	1.000000	H
H1419	1.0	0.500381	0.481969	0.045883	Biso	1.000000	H
O1886	1.0	0.357403	0.458819	0.948945	Biso	1.000000	O
H1420	1.0	0.354347	0.475542	0.930639	Biso	1.000000	H
H1421	1.0	0.368716	0.471953	0.966500	Biso	1.000000	H
O1887	1.0	0.751443	0.671492	0.049112	Biso	1.000000	O
H1422	1.0	0.734529	0.691023	0.051162	Biso	1.000000	H
H1423	1.0	0.772811	0.679989	0.058266	Biso	1.000000	H
O1888	1.0	0.413551	0.473015	0.508324	Biso	1.000000	O
H1424	1.0	0.420553	0.498708	0.505361	Biso	1.000000	H
H1425	1.0	0.409016	0.461839	0.487281	Biso	1.000000	H
H1426	1.0	0.959663	0.867155	0.350355	Biso	1.000000	H
H1427	1.0	0.955030	0.791210	0.473486	Biso	1.000000	H
H1428	1.0	0.829325	0.959354	0.392342	Biso	1.000000	H
O1889	1.0	0.256557	0.127199	0.666546	Biso	1.000000	O
H1429	1.0	0.261450	0.147962	0.680910	Biso	1.000000	H
H1430	1.0	0.271338	0.128408	0.647686	Biso	1.000000	H
O1890	1.0	0.540594	0.796967	0.989702	Biso	1.000000	O
H1431	1.0	0.525562	0.776968	0.982982	Biso	1.000000	H
H1432	1.0	0.563642	0.787561	0.991460	Biso	1.000000	H
O1891	1.0	0.930638	0.420785	0.999094	Biso	1.000000	O
H1433	1.0	0.920142	0.445192	0.997712	Biso	1.000000	H
H1434	1.0	0.913094	0.401830	0.000204	Biso	1.000000	H
O1892	1.0	0.883919	0.296496	0.310042	Biso	1.000000	O
H1435	1.0	0.897709	0.308605	0.293579	Biso	1.000000	H
H1436	1.0	0.873857	0.274081	0.301033	Biso	1.000000	H
O1893	1.0	0.559250	0.993287	0.083751	Biso	1.000000	O
H1437	1.0	0.580021	0.003981	0.092395	Biso	1.000000	H
H1438	1.0	0.546681	0.981355	0.101178	Biso	1.000000	H
O1894	1.0	0.417441	0.303013	0.534203	Biso	1.000000	O
H1439	1.0	0.441495	0.308959	0.533809	Biso	1.000000	H
H1440	1.0	0.413791	0.278911	0.524110	Biso	1.000000	H
O1895	1.0	0.571431	0.844570	0.089025	Biso	1.000000	O
H1441	1.0	0.583691	0.867787	0.091922	Biso	1.000000	H
H1442	1.0	0.561346	0.837118	0.109552	Biso	1.000000	H
O1896	1.0	0.963083	0.011663	0.735009	Biso	1.000000	O
H1443	1.0	0.981639	0.022209	0.722484	Biso	1.000000	H
H1444	1.0	0.962869	0.984814	0.732305	Biso	1.000000	H
O1897	1.0	0.537731	0.403749	0.448922	Biso	1.000000	O
H1445	1.0	0.553269	0.419322	0.461209	Biso	1.000000	H
H1446	1.0	0.533839	0.414749	0.427692	Biso	1.000000	H
O1898	1.0	0.380031	0.925142	0.355612	Biso	1.000000	O
H1447	1.0	0.367059	0.909546	0.340867	Biso	1.000000	H
H1448	1.0	0.365520	0.933299	0.373312	Biso	1.000000	H
O1899	1.0	0.848863	0.854946	0.281854	Biso	1.000000	O
H1449	1.0	0.847551	0.871393	0.300538	Biso	1.000000	H
H1450	1.0	0.826101	0.847772	0.275270	Biso	1.000000	H

O1900	1.0	0.888097	0.968695	0.010756	Biso	1.000000	O
H1451	1.0	0.875265	0.987391	0.022576	Biso	1.000000	H
H1452	1.0	0.894502	0.978476	0.989630	Biso	1.000000	H
O1901	1.0	0.836068	0.960351	0.763351	Biso	1.000000	O
H1453	1.0	0.855448	0.952431	0.750498	Biso	1.000000	H
H1454	1.0	0.840157	0.985202	0.771763	Biso	1.000000	H
O1902	1.0	0.587854	0.588527	0.276528	Biso	1.000000	O
H1455	1.0	0.589245	0.573653	0.256870	Biso	1.000000	H
H1456	1.0	0.568723	0.605488	0.274947	Biso	1.000000	H
O1903	1.0	0.781493	0.213678	0.366134	Biso	1.000000	O
H1457	1.0	0.777574	0.194520	0.349916	Biso	1.000000	H
H1458	1.0	0.804225	0.223721	0.363507	Biso	1.000000	H
O1904	1.0	0.477772	0.365525	0.262809	Biso	1.000000	O
H1459	1.0	0.454648	0.374090	0.266195	Biso	1.000000	H
H1460	1.0	0.489272	0.382155	0.247813	Biso	1.000000	H
O1905	1.0	0.066910	0.502530	0.171149	Biso	1.000000	O
H1461	1.0	0.077680	0.523404	0.182040	Biso	1.000000	H
H1462	1.0	0.043319	0.508536	0.166693	Biso	1.000000	H
O1906	1.0	0.459188	0.435253	0.392102	Biso	1.000000	O
H1463	1.0	0.461916	0.449495	0.411987	Biso	1.000000	H
H1464	1.0	0.442832	0.447578	0.378095	Biso	1.000000	H
O1907	1.0	0.742313	0.212975	0.086352	Biso	1.000000	O
H1465	1.0	0.742522	0.186127	0.083631	Biso	1.000000	H
H1466	1.0	0.765494	0.222128	0.087358	Biso	1.000000	H
O1908	1.0	0.773051	0.504841	0.833204	Biso	1.000000	O
H1467	1.0	0.774331	0.497587	0.855902	Biso	1.000000	H
H1468	1.0	0.749438	0.505541	0.826418	Biso	1.000000	H
O1909	1.0	0.134668	0.898951	0.760553	Biso	1.000000	O
H1469	1.0	0.136011	0.916490	0.778462	Biso	1.000000	H
H1470	1.0	0.156146	0.898627	0.748957	Biso	1.000000	H
O1910	1.0	0.353718	0.671367	0.396753	Biso	1.000000	O
H1471	1.0	0.341747	0.669667	0.417329	Biso	1.000000	H
H1472	1.0	0.377205	0.663880	0.399768	Biso	1.000000	H
O1911	1.0	0.114271	0.566729	0.651758	Biso	1.000000	O
H1473	1.0	0.098285	0.572672	0.668960	Biso	1.000000	H
H1474	1.0	0.116678	0.588024	0.637409	Biso	1.000000	H
O1912	1.0	0.752072	0.328122	0.585011	Biso	1.000000	O
H1475	1.0	0.761737	0.313281	0.602427	Biso	1.000000	H
H1476	1.0	0.763131	0.352272	0.584421	Biso	1.000000	H
O1913	1.0	0.557392	0.943986	0.139126	Biso	1.000000	O
H1477	1.0	0.538673	0.928264	0.146033	Biso	1.000000	H
H1478	1.0	0.572548	0.929796	0.125232	Biso	1.000000	H
O1914	1.0	0.417749	0.653058	0.244453	Biso	1.000000	O
H1479	1.0	0.408550	0.651080	0.266280	Biso	1.000000	H
H1480	1.0	0.402561	0.640806	0.229246	Biso	1.000000	H
O1915	1.0	0.459284	0.765146	0.692106	Biso	1.000000	O
H1481	1.0	0.455758	0.776384	0.670914	Biso	1.000000	H
H1482	1.0	0.483070	0.767208	0.698076	Biso	1.000000	H
O1916	1.0	0.694851	0.715540	0.053125	Biso	1.000000	O
H1483	1.0	0.682850	0.692831	0.058776	Biso	1.000000	H
H1484	1.0	0.684869	0.736382	0.064721	Biso	1.000000	H
O1917	1.0	0.798668	0.140565	0.944512	Biso	1.000000	O
H1485	1.0	0.799882	0.165536	0.935557	Biso	1.000000	H
H1486	1.0	0.810687	0.139802	0.965107	Biso	1.000000	H
O1918	1.0	0.376756	0.599498	0.061961	Biso	1.000000	O
H1487	1.0	0.397306	0.584935	0.064890	Biso	1.000000	H
H1488	1.0	0.357164	0.583084	0.062233	Biso	1.000000	H
O1919	1.0	0.237026	0.066725	0.498869	Biso	1.000000	O
H1489	1.0	0.220730	0.086870	0.500972	Biso	1.000000	H
H1490	1.0	0.232206	0.047741	0.515021	Biso	1.000000	H
O1920	1.0	0.726792	0.877201	0.369218	Biso	1.000000	O
H1491	1.0	0.718271	0.872133	0.390915	Biso	1.000000	H
H1492	1.0	0.732462	0.903422	0.367373	Biso	1.000000	H
O1921	1.0	0.436362	0.450619	0.080721	Biso	1.000000	O
H1493	1.0	0.454820	0.448630	0.065169	Biso	1.000000	H
H1494	1.0	0.416370	0.460563	0.069967	Biso	1.000000	H
O1922	1.0	0.341340	0.636495	0.341112	Biso	1.000000	O
H1495	1.0	0.345384	0.649171	0.361589	Biso	1.000000	H
H1496	1.0	0.320238	0.645374	0.331673	Biso	1.000000	H
O1923	1.0	0.620163	0.762900	0.320871	Biso	1.000000	O
H1497	1.0	0.622740	0.788436	0.313546	Biso	1.000000	H

H1498	1.0	0.638658	0.747918	0.312350	Biso	1.000000	H
O1924	1.0	0.415315	0.992149	0.274650	Biso	1.000000	O
H1499	1.0	0.427797	0.013285	0.266063	Biso	1.000000	H
H1500	1.0	0.391919	0.993273	0.267250	Biso	1.000000	H
O1925	1.0	0.104240	0.519564	0.602359	Biso	1.000000	O
H1501	1.0	0.120593	0.499506	0.604693	Biso	1.000000	H
H1502	1.0	0.106624	0.536789	0.620399	Biso	1.000000	H
O1926	1.0	0.762665	0.982517	0.844894	Biso	1.000000	O
H1503	1.0	0.787153	0.981821	0.842147	Biso	1.000000	H
H1504	1.0	0.756977	0.976430	0.867230	Biso	1.000000	H
O1927	1.0	0.579710	0.902033	0.580383	Biso	1.000000	O
H1505	1.0	0.587975	0.926523	0.586478	Biso	1.000000	H
H1506	1.0	0.597954	0.884004	0.582502	Biso	1.000000	H
O1928	1.0	0.438723	0.857873	0.674347	Biso	1.000000	O
H1507	1.0	0.443840	0.835896	0.661513	Biso	1.000000	H
H1508	1.0	0.416452	0.867279	0.668406	Biso	1.000000	H
O1929	1.0	0.375410	0.143961	0.547030	Biso	1.000000	O
H1509	1.0	0.362257	0.139515	0.527452	Biso	1.000000	H
H1510	1.0	0.394943	0.127463	0.547353	Biso	1.000000	H
O1930	1.0	0.225742	0.336077	0.684265	Biso	1.000000	O
H1511	1.0	0.204531	0.331104	0.695497	Biso	1.000000	H
H1512	1.0	0.244355	0.324549	0.696030	Biso	1.000000	H
O1931	1.0	0.333780	0.387180	0.328037	Biso	1.000000	O
H1513	1.0	0.342531	0.411314	0.334578	Biso	1.000000	H
H1514	1.0	0.345212	0.367760	0.340272	Biso	1.000000	H
O1932	1.0	0.296277	0.584232	0.230273	Biso	1.000000	O
H1515	1.0	0.288617	0.571940	0.210579	Biso	1.000000	H
H1516	1.0	0.302389	0.609804	0.225355	Biso	1.000000	H
O1933	1.0	0.139129	0.684422	0.882422	Biso	1.000000	O
H1517	1.0	0.147027	0.670567	0.863625	Biso	1.000000	H
H1518	1.0	0.117349	0.674608	0.889432	Biso	1.000000	H
O1934	1.0	0.071720	0.859900	0.818808	Biso	1.000000	O
H1519	1.0	0.057995	0.843383	0.805525	Biso	1.000000	H
H1520	1.0	0.057238	0.872860	0.834195	Biso	1.000000	H
O1935	1.0	0.581074	0.561196	0.336154	Biso	1.000000	O
H1521	1.0	0.586586	0.570277	0.314563	Biso	1.000000	H
H1522	1.0	0.559079	0.548982	0.335697	Biso	1.000000	H
O1936	1.0	0.786403	0.222787	0.433948	Biso	1.000000	O
H1523	1.0	0.775813	0.221239	0.412680	Biso	1.000000	H
H1524	1.0	0.808317	0.234990	0.432011	Biso	1.000000	H
O1937	1.0	0.629424	0.751418	0.385346	Biso	1.000000	O
H1525	1.0	0.653241	0.758386	0.386125	Biso	1.000000	H
H1526	1.0	0.620824	0.754986	0.363453	Biso	1.000000	H
O1938	1.0	0.267996	0.682895	0.174126	Biso	1.000000	O
H1527	1.0	0.248135	0.687949	0.187403	Biso	1.000000	H
H1528	1.0	0.271259	0.703091	0.158757	Biso	1.000000	H
O1939	1.0	0.442343	0.187830	0.628674	Biso	1.000000	O
H1529	1.0	0.430194	0.207300	0.617152	Biso	1.000000	H
H1530	1.0	0.426374	0.173148	0.641286	Biso	1.000000	H
O1940	1.0	0.713770	0.577199	0.142606	Biso	1.000000	O
H1531	1.0	0.731202	0.574013	0.126146	Biso	1.000000	H
H1532	1.0	0.699475	0.555187	0.143270	Biso	1.000000	H
O1941	1.0	0.624348	0.449746	0.626531	Biso	1.000000	O
H1533	1.0	0.624992	0.428637	0.641256	Biso	1.000000	H
H1534	1.0	0.636335	0.470593	0.636229	Biso	1.000000	H
O1942	1.0	0.125421	0.669535	0.780145	Biso	1.000000	O
H1535	1.0	0.104721	0.665243	0.767875	Biso	1.000000	H
H1536	1.0	0.121825	0.689739	0.795435	Biso	1.000000	H
O1943	1.0	0.198219	0.127914	0.200959	Biso	1.000000	O
H1537	1.0	0.207964	0.123520	0.179625	Biso	1.000000	H
H1538	1.0	0.186290	0.105739	0.208151	Biso	1.000000	H
O1944	1.0	0.360871	0.347186	0.467103	Biso	1.000000	O
H1539	1.0	0.346509	0.353583	0.448752	Biso	1.000000	H
H1540	1.0	0.383615	0.356773	0.463470	Biso	1.000000	H
O1945	1.0	0.179278	0.841559	0.692786	Biso	1.000000	O
H1541	1.0	0.190831	0.818573	0.698435	Biso	1.000000	H
H1542	1.0	0.154971	0.838418	0.695688	Biso	1.000000	H
O1946	1.0	0.776259	0.837002	0.342084	Biso	1.000000	O
H1543	1.0	0.757873	0.851640	0.351256	Biso	1.000000	H
H1544	1.0	0.797090	0.842069	0.353915	Biso	1.000000	H
O1947	1.0	0.624923	0.831201	0.552358	Biso	1.000000	O
H1545	1.0	0.628728	0.807724	0.563469	Biso	1.000000	H
H1546	1.0	0.603167	0.830447	0.541260	Biso	1.000000	H
O1948	1.0	0.807323	0.588427	0.235763	Biso	1.000000	O
H1547	1.0	0.815227	0.570286	0.219989	Biso	1.000000	H
H1548	1.0	0.826089	0.604352	0.242176	Biso	1.000000	H
O1949	1.0	0.396294	0.577043	0.953957	Biso	1.000000	O
H1549	1.0	0.407639	0.599655	0.960978	Biso	1.000000	H
H1550	1.0	0.378293	0.570921	0.969174	Biso	1.000000	H
O1950	1.0	0.355373	0.454716	0.344319	Biso	1.000000	O
H1551	1.0	0.343643	0.473801	0.331938	Biso	1.000000	H
H1552	1.0	0.379477	0.460382	0.345064	Biso	1.000000	H

O1951	1.0	0.971025	0.118795	0.783153	Biso	1.000000	O
H1553	1.0	0.954084	0.136588	0.790416	Biso	1.000000	H
H1554	1.0	0.993193	0.126097	0.791297	Biso	1.000000	H
O1952	1.0	0.008346	0.820520	0.831729	Biso	1.000000	O
H1555	1.0	0.019274	0.816014	0.810946	Biso	1.000000	H
H1556	1.0	0.024963	0.818165	0.849045	Biso	1.000000	H
O1953	1.0	0.315305	0.961558	0.307020	Biso	1.000000	O
H1557	1.0	0.322203	0.936253	0.312036	Biso	1.000000	H
H1558	1.0	0.311258	0.975074	0.327086	Biso	1.000000	H
O1954	1.0	0.502887	0.740950	0.969659	Biso	1.000000	O
H1559	1.0	0.478800	0.744674	0.965767	Biso	1.000000	H
H1560	1.0	0.506043	0.719827	0.984069	Biso	1.000000	H
O1955	1.0	0.114008	0.031424	0.142896	Biso	1.000000	O
H1561	1.0	0.119623	0.020434	0.163773	Biso	1.000000	H
H1562	1.0	0.096627	0.050298	0.145858	Biso	1.000000	H
O1956	1.0	0.236747	0.997765	0.359869	Biso	1.000000	O
H1563	1.0	0.222240	0.988377	0.377101	Biso	1.000000	H
H1564	1.0	0.223016	0.003600	0.340940	Biso	1.000000	H
O1957	1.0	0.978168	0.940489	0.718034	Biso	1.000000	O
H1565	1.0	0.991602	0.944513	0.698560	Biso	1.000000	H
H1566	1.0	0.962275	0.920258	0.714339	Biso	1.000000	H
O1958	1.0	0.006226	0.726444	0.771365	Biso	1.000000	O
H1567	1.0	0.005519	0.703185	0.759361	Biso	1.000000	H
H1568	1.0	0.022176	0.743326	0.761036	Biso	1.000000	H
O1959	1.0	0.464829	0.504182	0.868531	Biso	1.000000	O
H1569	1.0	0.448285	0.486421	0.860409	Biso	1.000000	H
H1570	1.0	0.456690	0.514863	0.888758	Biso	1.000000	H
O1960	1.0	0.772247	0.562647	0.107444	Biso	1.000000	O
H1571	1.0	0.784307	0.572683	0.126068	Biso	1.000000	H
H1572	1.0	0.778442	0.576551	0.088096	Biso	1.000000	H
O1961	1.0	0.065003	0.172026	0.405625	Biso	1.000000	O
H1573	1.0	0.085602	0.186633	0.403223	Biso	1.000000	H
H1574	1.0	0.064496	0.160845	0.427103	Biso	1.000000	H
O1962	1.0	0.365211	0.769473	0.094384	Biso	1.000000	O
H1575	1.0	0.342792	0.779871	0.098169	Biso	1.000000	H
H1576	1.0	0.377222	0.784680	0.078620	Biso	1.000000	H
O1963	1.0	0.690352	0.062512	0.259546	Biso	1.000000	O
H1577	1.0	0.670836	0.047744	0.266023	Biso	1.000000	H
H1578	1.0	0.707972	0.060484	0.275964	Biso	1.000000	H
O1964	1.0	0.405442	0.448119	0.449276	Biso	1.000000	O
H1579	1.0	0.426243	0.461314	0.443976	Biso	1.000000	H
H1580	1.0	0.388101	0.454087	0.433327	Biso	1.000000	H
O1965	1.0	0.728970	0.949062	0.740812	Biso	1.000000	O
H1581	1.0	0.723892	0.941262	0.718747	Biso	1.000000	H
H1582	1.0	0.708919	0.960311	0.750418	Biso	1.000000	H
O1966	1.0	0.412094	0.588476	0.762757	Biso	1.000000	O
H1583	1.0	0.389534	0.578827	0.758299	Biso	1.000000	H
H1584	1.0	0.417917	0.607139	0.746621	Biso	1.000000	H
O1967	1.0	0.989355	0.679560	0.825495	Biso	1.000000	O
H1585	1.0	0.992471	0.655196	0.815721	Biso	1.000000	H
H1586	1.0	0.001300	0.698120	0.812706	Biso	1.000000	H
O1968	1.0	0.092791	0.257099	0.945193	Biso	1.000000	O
H1587	1.0	0.116559	0.263244	0.941888	Biso	1.000000	H
H1588	1.0	0.081779	0.253861	0.924269	Biso	1.000000	H
O1969	1.0	0.570242	0.752261	0.641265	Biso	1.000000	O
H1589	1.0	0.574822	0.731914	0.656166	Biso	1.000000	H
H1590	1.0	0.571261	0.743172	0.619063	Biso	1.000000	H
O1970	1.0	0.302337	0.618356	0.003790	Biso	1.000000	O
H1591	1.0	0.278966	0.615113	0.010779	Biso	1.000000	H
H1592	1.0	0.309561	0.643676	0.008304	Biso	1.000000	H
O1971	1.0	0.217711	0.880053	0.786634	Biso	1.000000	O
H1593	1.0	0.202584	0.859360	0.791212	Biso	1.000000	H
H1594	1.0	0.210686	0.892183	0.766648	Biso	1.000000	H
O1972	1.0	0.315904	0.651980	0.212039	Biso	1.000000	O
H1595	1.0	0.316082	0.666059	0.232181	Biso	1.000000	H
H1596	1.0	0.299629	0.662945	0.197115	Biso	1.000000	H
O1973	1.0	0.694044	0.413933	0.640373	Biso	1.000000	O
H1597	1.0	0.689289	0.424163	0.619011	Biso	1.000000	H
H1598	1.0	0.672883	0.410492	0.652115	Biso	1.000000	H

O1974	1.0	0.978144	0.048138	0.936098	Biso	1.000000	O
H1599	1.0	0.955362	0.051849	0.944537	Biso	1.000000	H
H1600	1.0	0.994076	0.046436	0.954049	Biso	1.000000	H
O1975	1.0	0.534801	0.634511	0.268921	Biso	1.000000	O
H1601	1.0	0.512049	0.624189	0.267569	Biso	1.000000	H
H1602	1.0	0.534236	0.657456	0.281377	Biso	1.000000	H
O1976	1.0	0.070182	0.953881	0.931653	Biso	1.000000	O
H1603	1.0	0.057493	0.931310	0.927056	Biso	1.000000	H
H1604	1.0	0.072930	0.956580	0.954984	Biso	1.000000	H
O1977	1.0	0.502290	0.838488	0.288598	Biso	1.000000	O
H1605	1.0	0.512678	0.828425	0.308115	Biso	1.000000	H
H1606	1.0	0.481390	0.825128	0.284025	Biso	1.000000	H
O1978	1.0	0.720126	0.897954	0.933908	Biso	1.000000	O
H1607	1.0	0.705873	0.875896	0.933928	Biso	1.000000	H
H1608	1.0	0.727431	0.903349	0.955948	Biso	1.000000	H
O1979	1.0	0.940697	0.711510	0.863571	Biso	1.000000	O
H1609	1.0	0.957417	0.699323	0.849870	Biso	1.000000	H
H1610	1.0	0.930482	0.732066	0.851770	Biso	1.000000	H
O1980	1.0	0.237762	0.689439	0.876386	Biso	1.000000	O
H1611	1.0	0.246102	0.706972	0.892474	Biso	1.000000	H
H1612	1.0	0.256054	0.682860	0.861638	Biso	1.000000	H
O1981	1.0	0.247464	0.013843	0.224196	Biso	1.000000	O
H1613	1.0	0.235991	0.995381	0.210911	Biso	1.000000	H
H1614	1.0	0.259958	0.030844	0.210281	Biso	1.000000	H
O1982	1.0	0.288139	0.622724	0.137429	Biso	1.000000	O
H1615	1.0	0.309403	0.630665	0.127689	Biso	1.000000	H
H1616	1.0	0.278801	0.642890	0.150354	Biso	1.000000	H
O1983	1.0	0.640934	0.342664	0.605754	Biso	1.000000	O
H1617	1.0	0.632536	0.317743	0.601401	Biso	1.000000	H
H1618	1.0	0.663649	0.341156	0.614853	Biso	1.000000	H
O1984	1.0	0.020811	0.122382	0.383685	Biso	1.000000	O
H1619	1.0	0.037142	0.141636	0.389176	Biso	1.000000	H
H1620	1.0	0.019686	0.119654	0.360235	Biso	1.000000	H
O1985	1.0	0.437440	0.754543	0.963695	Biso	1.000000	O
H1621	1.0	0.423037	0.744483	0.980717	Biso	1.000000	H
H1622	1.0	0.441606	0.780758	0.967827	Biso	1.000000	H
O1986	1.0	0.207447	0.624621	0.888694	Biso	1.000000	O
H1623	1.0	0.223560	0.606580	0.897122	Biso	1.000000	H
H1624	1.0	0.218828	0.648298	0.885388	Biso	1.000000	H
O1987	1.0	0.801315	0.567172	0.016264	Biso	1.000000	O
H1625	1.0	0.777946	0.558539	0.016684	Biso	1.000000	H
H1626	1.0	0.805073	0.584300	0.034160	Biso	1.000000	H
O1988	1.0	0.368561	0.776666	0.932579	Biso	1.000000	O
H1627	1.0	0.365621	0.779395	0.955886	Biso	1.000000	H
H1628	1.0	0.346507	0.773722	0.922333	Biso	1.000000	H
O1989	1.0	0.824691	0.108474	0.702833	Biso	1.000000	O
H1629	1.0	0.817590	0.134212	0.700429	Biso	1.000000	H
H1630	1.0	0.849267	0.106974	0.701356	Biso	1.000000	H
O1990	1.0	0.966059	0.095170	0.121094	Biso	1.000000	O
H1631	1.0	0.954571	0.099935	0.100632	Biso	1.000000	H
H1632	1.0	0.962936	0.116286	0.135517	Biso	1.000000	H
O1991	1.0	0.283231	0.097584	0.235642	Biso	1.000000	O
H1633	1.0	0.281294	0.084675	0.214993	Biso	1.000000	H
H1634	1.0	0.271905	0.121537	0.234188	Biso	1.000000	H
O1992	1.0	0.321836	0.063058	0.278121	Biso	1.000000	O
H1635	1.0	0.307692	0.075390	0.262068	Biso	1.000000	H
H1636	1.0	0.342181	0.077726	0.281859	Biso	1.000000	H
O1993	1.0	0.731473	0.068440	0.987824	Biso	1.000000	O
H1637	1.0	0.739178	0.052345	0.970358	Biso	1.000000	H
H1638	1.0	0.729118	0.053997	0.007642	Biso	1.000000	H
O1994	1.0	0.201894	0.371460	0.531166	Biso	1.000000	O
H1639	1.0	0.193755	0.368787	0.553318	Biso	1.000000	H
H1640	1.0	0.188343	0.390021	0.519936	Biso	1.000000	H
O1995	1.0	0.293964	0.189392	0.572651	Biso	1.000000	O
H1641	1.0	0.316215	0.200976	0.571488	Biso	1.000000	H
H1642	1.0	0.293555	0.171666	0.590462	Biso	1.000000	H
O1996	1.0	0.413176	0.807443	0.890395	Biso	1.000000	O
H1643	1.0	0.400990	0.792296	0.906082	Biso	1.000000	H
H1644	1.0	0.437349	0.806397	0.894983	Biso	1.000000	H
O1997	1.0	0.527575	0.780833	0.686509	Biso	1.000000	O
H1645	1.0	0.541369	0.768543	0.670157	Biso	1.000000	H
H1646	1.0	0.524894	0.806908	0.680851	Biso	1.000000	H
O1998	1.0	0.308284	0.015653	0.052689	Biso	1.000000	O
H1647	1.0	0.319745	0.014136	0.031837	Biso	1.000000	H
H1648	1.0	0.297202	0.992096	0.057322	Biso	1.000000	H
O1999	1.0	0.464072	0.148215	0.578722	Biso	1.000000	O
H1649	1.0	0.456357	0.162368	0.597423	Biso	1.000000	H
H1650	1.0	0.472573	0.165158	0.562235	Biso	1.000000	H
O2000	1.0	0.837688	0.909425	0.484617	Biso	1.000000	O
H1651	1.0	0.856787	0.897708	0.473746	Biso	1.000000	H
H1652	1.0	0.824767	0.890691	0.496299	Biso	1.000000	H
O2001	1.0	0.340402	0.303997	0.605915	Biso	1.000000	O
H1653	1.0	0.351222	0.316716	0.587852	Biso	1.000000	H

H1654	1.0	0.357679	0.294593	0.620616	Biso	1.000000	H
O2002	1.0	0.692762	0.773425	0.376481	Biso	1.000000	O
H1655	1.0	0.690636	0.794410	0.361749	Biso	1.000000	H
H1656	1.0	0.704030	0.752997	0.365412	Biso	1.000000	H
O2003	1.0	0.655888	0.927683	0.718957	Biso	1.000000	O
H1657	1.0	0.673512	0.931061	0.702713	Biso	1.000000	H
H1658	1.0	0.658408	0.946359	0.735845	Biso	1.000000	H
O2004	1.0	0.080158	0.949836	0.323792	Biso	1.000000	O
H1659	1.0	0.097240	0.967306	0.316236	Biso	1.000000	H
H1660	1.0	0.075974	0.953853	0.346780	Biso	1.000000	H
O2005	1.0	0.901263	0.834472	0.718086	Biso	1.000000	O
H1661	1.0	0.912539	0.812238	0.726064	Biso	1.000000	H
H1662	1.0	0.877233	0.833236	0.723289	Biso	1.000000	H
O2006	1.0	0.656832	0.367800	0.290818	Biso	1.000000	O
H1663	1.0	0.662576	0.387896	0.305613	Biso	1.000000	H
H1664	1.0	0.642397	0.349935	0.301899	Biso	1.000000	H
O2007	1.0	0.282158	0.949884	0.072124	Biso	1.000000	O
H1665	1.0	0.281743	0.926234	0.060708	Biso	1.000000	H
H1666	1.0	0.294815	0.947176	0.092237	Biso	1.000000	H
O2008	1.0	0.874631	0.896327	0.927464	Biso	1.000000	O
H1667	1.0	0.870438	0.898957	0.950603	Biso	1.000000	H
H1668	1.0	0.858267	0.879099	0.918221	Biso	1.000000	H
O2009	1.0	0.729352	0.384204	0.494313	Biso	1.000000	O
H1669	1.0	0.733584	0.398919	0.474940	Biso	1.000000	H
H1670	1.0	0.718360	0.361019	0.488272	Biso	1.000000	H
O2010	1.0	0.460683	0.541984	0.756867	Biso	1.000000	O
H1671	1.0	0.442619	0.559977	0.760241	Biso	1.000000	H
H1672	1.0	0.469670	0.544665	0.735016	Biso	1.000000	H
O2011	1.0	0.717627	0.217892	0.590709	Biso	1.000000	O
H1673	1.0	0.729384	0.210061	0.610292	Biso	1.000000	H
H1674	1.0	0.731116	0.211300	0.571811	Biso	1.000000	H
O2012	1.0	0.448936	0.316430	0.429554	Biso	1.000000	O
H1675	1.0	0.438237	0.339674	0.435899	Biso	1.000000	H
H1676	1.0	0.432525	0.296331	0.431100	Biso	1.000000	H
O2013	1.0	0.393769	0.045166	0.021827	Biso	1.000000	O
H1677	1.0	0.410520	0.033911	0.007564	Biso	1.000000	H
H1678	1.0	0.371402	0.035453	0.016638	Biso	1.000000	H
O2014	1.0	0.286622	0.167642	0.298079	Biso	1.000000	O
H1679	1.0	0.274364	0.146312	0.289565	Biso	1.000000	H
H1680	1.0	0.304305	0.174817	0.282869	Biso	1.000000	H
O2015	1.0	0.272411	0.393751	0.730845	Biso	1.000000	O
H1681	1.0	0.259088	0.376639	0.743924	Biso	1.000000	H
H1682	1.0	0.258242	0.404116	0.713780	Biso	1.000000	H
O2016	1.0	0.856094	0.508941	0.870142	Biso	1.000000	O
H1683	1.0	0.844120	0.528664	0.858783	Biso	1.000000	H
H1684	1.0	0.851687	0.510879	0.893297	Biso	1.000000	H
O2017	1.0	0.664872	0.503695	0.472061	Biso	1.000000	O
H1685	1.0	0.668084	0.494913	0.449956	Biso	1.000000	H
H1686	1.0	0.655318	0.483834	0.485197	Biso	1.000000	H
O2018	1.0	0.406942	0.954267	0.505729	Biso	1.000000	O
H1687	1.0	0.428275	0.950626	0.494322	Biso	1.000000	H
H1688	1.0	0.411290	0.956653	0.528860	Biso	1.000000	H
O2019	1.0	0.548963	0.138433	0.370464	Biso	1.000000	O
H1689	1.0	0.555895	0.162553	0.362134	Biso	1.000000	H
H1690	1.0	0.531084	0.128323	0.356815	Biso	1.000000	H
O2020	1.0	0.499005	0.477621	0.293478	Biso	1.000000	O
H1691	1.0	0.491537	0.485140	0.271967	Biso	1.000000	H
H1692	1.0	0.484895	0.457279	0.301170	Biso	1.000000	H
O2021	1.0	0.164473	0.198233	0.441945	Biso	1.000000	O
H1693	1.0	0.173839	0.221667	0.449555	Biso	1.000000	H
H1694	1.0	0.182844	0.181000	0.437306	Biso	1.000000	H
O2022	1.0	0.606046	0.914695	0.851796	Biso	1.000000	O
H1695	1.0	0.591100	0.929626	0.865303	Biso	1.000000	H
H1696	1.0	0.622777	0.902370	0.865390	Biso	1.000000	H
O2023	1.0	0.596743	0.458765	0.200174	Biso	1.000000	O
H1697	1.0	0.604166	0.447275	0.220317	Biso	1.000000	H
H1698	1.0	0.613948	0.455116	0.183570	Biso	1.000000	H
O2024	1.0	0.838581	0.713048	0.906315	Biso	1.000000	O
H1699	1.0	0.823307	0.696507	0.894710	Biso	1.000000	H
H1700	1.0	0.861056	0.701972	0.907259	Biso	1.000000	H
O2025	1.0	0.063713	0.607945	0.242922	Biso	1.000000	O
H1701	1.0	0.045960	0.613623	0.258536	Biso	1.000000	H
H1702	1.0	0.084234	0.621418	0.248658	Biso	1.000000	H
O2026	1.0	0.501518	0.119544	0.327204	Biso	1.000000	O
H1703	1.0	0.486074	0.102596	0.338136	Biso	1.000000	H
H1704	1.0	0.490069	0.143036	0.323181	Biso	1.000000	H
O2027	1.0	0.409568	0.206891	0.004863	Biso	1.000000	O
H1705	1.0	0.407852	0.181355	0.012419	Biso	1.000000	H
H1706	1.0	0.396196	0.222950	0.018883	Biso	1.000000	H
O2028	1.0	0.237081	0.987901	0.756926	Biso	1.000000	O
H1707	1.0	0.258885	0.989858	0.767818	Biso	1.000000	H
H1708	1.0	0.219434	0.998026	0.770838	Biso	1.000000	H
O2029	1.0	0.929136	0.477475	0.479907	Biso	1.000000	O
H1709	1.0	0.929210	0.471445	0.502909	Biso	1.000000	H
H1710	1.0	0.934852	0.455417	0.467415	Biso	1.000000	H

O2030	1.0	0.567243	0.109889	0.709348	Biso	1.000000	O
H1711	1.0	0.559432	0.135170	0.705627	Biso	1.000000	H
H1712	1.0	0.567270	0.104741	0.732513	Biso	1.000000	H
O2031	1.0	0.559874	0.658548	0.981580	Biso	1.000000	O
H1713	1.0	0.539258	0.668740	0.990992	Biso	1.000000	H
H1714	1.0	0.578161	0.676516	0.983697	Biso	1.000000	H
O2032	1.0	0.673302	0.114808	0.795044	Biso	1.000000	O
H1715	1.0	0.690130	0.134406	0.792830	Biso	1.000000	H
H1716	1.0	0.652045	0.122870	0.785367	Biso	1.000000	H
O2033	1.0	0.293838	0.843553	0.945707	Biso	1.000000	O
H1717	1.0	0.283936	0.854524	0.926335	Biso	1.000000	H
H1718	1.0	0.304739	0.862852	0.958517	Biso	1.000000	H
O2034	1.0	0.898360	0.034139	0.744453	Biso	1.000000	O
H1719	1.0	0.920938	0.024484	0.740087	Biso	1.000000	H
H1720	1.0	0.900126	0.059265	0.752981	Biso	1.000000	H
O2035	1.0	0.008009	0.882572	0.037041	Biso	1.000000	O
H1721	1.0	0.025045	0.872274	0.051545	Biso	1.000000	H
H1722	1.0	0.986846	0.885905	0.048803	Biso	1.000000	H
O2036	1.0	0.896323	0.992699	0.951182	Biso	1.000000	O
H1723	1.0	0.872535	0.986026	0.948955	Biso	1.000000	H
H1724	1.0	0.909690	0.978831	0.935478	Biso	1.000000	H
O2037	1.0	0.783890	0.404443	0.528414	Biso	1.000000	O
H1725	1.0	0.764413	0.395640	0.516147	Biso	1.000000	H
H1726	1.0	0.781027	0.398078	0.551183	Biso	1.000000	H
O2038	1.0	0.454436	0.703491	0.211971	Biso	1.000000	O
H1727	1.0	0.464787	0.688747	0.194852	Biso	1.000000	H
H1728	1.0	0.441951	0.687244	0.226565	Biso	1.000000	H
O2039	1.0	0.426115	0.048564	0.118767	Biso	1.000000	O
H1729	1.0	0.420173	0.064637	0.100668	Biso	1.000000	H
H1730	1.0	0.419202	0.023215	0.113942	Biso	1.000000	H
O2040	1.0	0.618498	0.612664	0.522746	Biso	1.000000	O
H1731	1.0	0.623455	0.587987	0.531123	Biso	1.000000	H
H1732	1.0	0.638007	0.628743	0.526126	Biso	1.000000	H
O2041	1.0	0.228911	0.801135	0.878790	Biso	1.000000	O
H1733	1.0	0.238119	0.780466	0.891186	Biso	1.000000	H
H1734	1.0	0.226868	0.793983	0.856119	Biso	1.000000	H
O2042	1.0	0.261261	0.978975	0.944257	Biso	1.000000	O
H1735	1.0	0.280249	0.975659	0.929480	Biso	1.000000	H
H1736	1.0	0.240890	0.984595	0.931894	Biso	1.000000	H
O2043	1.0	0.860884	0.492451	0.534137	Biso	1.000000	O
H1737	1.0	0.840534	0.504295	0.542553	Biso	1.000000	H
H1738	1.0	0.871203	0.508387	0.517836	Biso	1.000000	H
O2044	1.0	0.571877	0.178167	0.274327	Biso	1.000000	O
H1739	1.0	0.556783	0.185513	0.256801	Biso	1.000000	H
H1740	1.0	0.568952	0.151825	0.278808	Biso	1.000000	H
O2045	1.0	0.028124	0.343110	0.096578	Biso	1.000000	O
H1741	1.0	0.041414	0.335631	0.077804	Biso	1.000000	H
H1742	1.0	0.024939	0.369911	0.096262	Biso	1.000000	H
O2046	1.0	0.095661	0.849283	0.912825	Biso	1.000000	O
H1743	1.0	0.100387	0.829869	0.928609	Biso	1.000000	H
H1744	1.0	0.116854	0.860508	0.905780	Biso	1.000000	H
O2047	1.0	0.563987	0.010575	0.389752	Biso	1.000000	O
H1745	1.0	0.563842	0.000350	0.411595	Biso	1.000000	H
H1746	1.0	0.572568	0.035906	0.390346	Biso	1.000000	H
O2048	1.0	0.471562	0.955413	0.903801	Biso	1.000000	O
H1747	1.0	0.481018	0.942040	0.885397	Biso	1.000000	H
H1748	1.0	0.479424	0.944081	0.923860	Biso	1.000000	H
O2049	1.0	0.452847	0.799126	0.636418	Biso	1.000000	O
H1749	1.0	0.448923	0.776528	0.624030	Biso	1.000000	H
H1750	1.0	0.462160	0.818163	0.622232	Biso	1.000000	H
O2050	1.0	0.400241	0.216494	0.939074	Biso	1.000000	O
H1751	1.0	0.395517	0.242791	0.936020	Biso	1.000000	H
H1752	1.0	0.401030	0.210920	0.962151	Biso	1.000000	H
O2051	1.0	0.029783	0.900243	0.862679	Biso	1.000000	O
H1753	1.0	0.034632	0.892943	0.884921	Biso	1.000000	H
H1754	1.0	0.006966	0.910406	0.861430	Biso	1.000000	H
O2052	1.0	0.076781	0.950284	0.676690	Biso	1.000000	O
H1755	1.0	0.096308	0.933867	0.675157	Biso	1.000000	H
H1756	1.0	0.084173	0.975947	0.674496	Biso	1.000000	H
O2053	1.0	0.617327	0.807081	0.722647	Biso	1.000000	O
H1757	1.0	0.627678	0.828250	0.733469	Biso	1.000000	H
H1758	1.0	0.633738	0.795691	0.708107	Biso	1.000000	H
O2054	1.0	0.702866	0.118248	0.377172	Biso	1.000000	O
H1759	1.0	0.685511	0.134170	0.367800	Biso	1.000000	H
H1760	1.0	0.721425	0.115401	0.361832	Biso	1.000000	H
O2055	1.0	0.255714	0.929396	0.621449	Biso	1.000000	O
H1761	1.0	0.258301	0.916755	0.642159	Biso	1.000000	H
H1762	1.0	0.240025	0.950062	0.623896	Biso	1.000000	H
O2056	1.0	0.397846	0.095440	0.349328	Biso	1.000000	O
H1763	1.0	0.382111	0.075656	0.354973	Biso	1.000000	H
H1764	1.0	0.420972	0.087016	0.352957	Biso	1.000000	H
O2057	1.0	0.548048	0.027015	0.727492	Biso	1.000000	O
H1765	1.0	0.559760	0.013788	0.744752	Biso	1.000000	H

H1766	1.0	0.564214	0.033881	0.710710	Biso	1.000000	H
O2058	1.0	0.010399	0.348094	0.959466	Biso	1.000000	O
H1767	1.0	0.994960	0.351032	0.977687	Biso	1.000000	H
H1768	1.0	0.021222	0.371776	0.954764	Biso	1.000000	H
O2059	1.0	0.973398	0.868660	0.793545	Biso	1.000000	O
H1769	1.0	0.974423	0.854067	0.773708	Biso	1.000000	H
H1770	1.0	0.983872	0.854813	0.811160	Biso	1.000000	H
O2060	1.0	0.981681	0.403986	0.810601	Biso	1.000000	O
H1771	1.0	0.966730	0.418839	0.797033	Biso	1.000000	H
H1772	1.0	0.988455	0.381734	0.798883	Biso	1.000000	H
O2061	1.0	0.426645	0.947251	0.963604	Biso	1.000000	O
H1773	1.0	0.413998	0.943713	0.943582	Biso	1.000000	H
H1774	1.0	0.449493	0.937537	0.960971	Biso	1.000000	H
O2062	1.0	0.119671	0.450676	0.293006	Biso	1.000000	O
H1775	1.0	0.116058	0.476645	0.287449	Biso	1.000000	H
H1776	1.0	0.135190	0.449024	0.311288	Biso	1.000000	H
O2063	1.0	0.810031	0.534751	0.560631	Biso	1.000000	O
H1777	1.0	0.817553	0.560474	0.559803	Biso	1.000000	H
H1778	1.0	0.786213	0.533394	0.554618	Biso	1.000000	H
O2064	1.0	0.534147	0.268522	0.238828	Biso	1.000000	O
H1779	1.0	0.534041	0.241496	0.239125	Biso	1.000000	H
H1780	1.0	0.529279	0.277241	0.216984	Biso	1.000000	H
O2065	1.0	0.190446	0.121645	0.505257	Biso	1.000000	O
H1781	1.0	0.173302	0.122248	0.522211	Biso	1.000000	H
H1782	1.0	0.201626	0.145694	0.504006	Biso	1.000000	H
O2066	1.0	0.263098	0.668297	0.945985	Biso	1.000000	O
H1783	1.0	0.279114	0.688439	0.949568	Biso	1.000000	H
H1784	1.0	0.275442	0.645409	0.941742	Biso	1.000000	H
O2067	1.0	0.867634	0.774695	0.040219	Biso	1.000000	O
H1785	1.0	0.872813	0.748275	0.040635	Biso	1.000000	H
H1786	1.0	0.848259	0.779639	0.054166	Biso	1.000000	H
O2068	1.0	0.645581	0.610615	0.856284	Biso	1.000000	O
H1787	1.0	0.623549	0.615767	0.865888	Biso	1.000000	H
H1788	1.0	0.662690	0.609693	0.873262	Biso	1.000000	H
O2069	1.0	0.356278	0.772108	0.576353	Biso	1.000000	O
H1789	1.0	0.359243	0.774006	0.599721	Biso	1.000000	H
H1790	1.0	0.336794	0.756443	0.571643	Biso	1.000000	H
O2070	1.0	0.299760	0.789677	0.089801	Biso	1.000000	O
H1791	1.0	0.284659	0.810905	0.091935	Biso	1.000000	H
H1792	1.0	0.304897	0.785601	0.066998	Biso	1.000000	H
O2071	1.0	0.162107	0.445778	0.341652	Biso	1.000000	O
H1793	1.0	0.153525	0.455546	0.362063	Biso	1.000000	H
H1794	1.0	0.167155	0.419471	0.344099	Biso	1.000000	H
O2072	1.0	0.457420	0.258104	0.157976	Biso	1.000000	O
H1795	1.0	0.480552	0.263828	0.164458	Biso	1.000000	H
H1796	1.0	0.454968	0.231380	0.155339	Biso	1.000000	H
O2073	1.0	0.457856	0.153816	0.423995	Biso	1.000000	O
H1797	1.0	0.447624	0.129315	0.422150	Biso	1.000000	H
H1798	1.0	0.482312	0.151278	0.426093	Biso	1.000000	H
O2074	1.0	0.236854	0.917036	0.396944	Biso	1.000000	O
H1799	1.0	0.254510	0.925172	0.411810	Biso	1.000000	H
H1800	1.0	0.219313	0.935978	0.395627	Biso	1.000000	H
O2075	1.0	0.226226	0.022609	0.143551	Biso	1.000000	O
H1801	1.0	0.230918	0.013698	0.121731	Biso	1.000000	H
H1802	1.0	0.224205	0.001548	0.158214	Biso	1.000000	H
O2076	1.0	0.757133	0.010312	0.498392	Biso	1.000000	O
H1803	1.0	0.741610	0.991359	0.490485	Biso	1.000000	H
H1804	1.0	0.762280	0.005663	0.521109	Biso	1.000000	H
O2077	1.0	0.560771	0.893424	0.931044	Biso	1.000000	O
H1805	1.0	0.554332	0.919293	0.934023	Biso	1.000000	H
H1806	1.0	0.584665	0.890370	0.936251	Biso	1.000000	H
O2078	1.0	0.456811	0.848451	0.410139	Biso	1.000000	O
H1807	1.0	0.468301	0.838583	0.429158	Biso	1.000000	H
H1808	1.0	0.457385	0.830115	0.392812	Biso	1.000000	H
O2079	1.0	0.341438	0.507417	0.901122	Biso	1.000000	O
H1809	1.0	0.317352	0.505079	0.905757	Biso	1.000000	H
H1810	1.0	0.348147	0.533340	0.902957	Biso	1.000000	H
O2080	1.0	0.612125	0.452297	0.337115	Biso	1.000000	O
H1811	1.0	0.609804	0.475766	0.348609	Biso	1.000000	H
H1812	1.0	0.592439	0.448459	0.323301	Biso	1.000000	H
O2081	1.0	0.021798	0.280490	0.791167	Biso	1.000000	O
H1813	1.0	0.016312	0.259603	0.805195	Biso	1.000000	H
H1814	1.0	0.043207	0.291401	0.797984	Biso	1.000000	H
O2082	1.0	0.373945	0.346296	0.563408	Biso	1.000000	O
H1815	1.0	0.390506	0.330598	0.552549	Biso	1.000000	H
H1816	1.0	0.360816	0.359805	0.547287	Biso	1.000000	H
O2083	1.0	0.599954	0.359954	0.236058	Biso	1.000000	O
H1817	1.0	0.588175	0.343250	0.250794	Biso	1.000000	H
H1818	1.0	0.617714	0.346394	0.224745	Biso	1.000000	H

O2084	1.0	0.774585	0.766611	0.026576	Biso	1.000000	O
H1819	1.0	0.755660	0.751651	0.018936	Biso	1.000000	H
H1820	1.0	0.771356	0.792283	0.019868	Biso	1.000000	H
O2085	1.0	0.422275	0.051388	0.701583	Biso	1.000000	O
H1821	1.0	0.435361	0.033214	0.713761	Biso	1.000000	H
H1822	1.0	0.423683	0.045750	0.678545	Biso	1.000000	H
O2086	1.0	0.259260	0.257267	0.502663	Biso	1.000000	O
H1823	1.0	0.280064	0.257512	0.515335	Biso	1.000000	H
H1824	1.0	0.246090	0.279444	0.507484	Biso	1.000000	H
O2087	1.0	0.530828	0.809976	0.341735	Biso	1.000000	O
H1825	1.0	0.540755	0.785253	0.340904	Biso	1.000000	H
H1826	1.0	0.548674	0.828298	0.344795	Biso	1.000000	H
O2088	1.0	0.427686	0.409035	0.710231	Biso	1.000000	O
H1827	1.0	0.446968	0.407706	0.724896	Biso	1.000000	H
H1828	1.0	0.435692	0.413438	0.688243	Biso	1.000000	H
H1829	1.0	0.170589	0.496046	0.235055	Biso	1.000000	H
H1830	1.0	0.136364	0.422496	0.264341	Biso	1.000000	H
H1831	1.0	0.238072	0.168835	0.437636	Biso	1.000000	H
H1832	1.0	0.287105	0.871352	0.479277	Biso	1.000000	H
H1833	1.0	0.183765	0.295726	0.398714	Biso	1.000000	H
H1834	1.0	0.521118	0.482324	0.188076	Biso	1.000000	H
O2089	1.0	0.674869	0.207344	0.182313	Biso	1.000000	O
H1835	1.0	0.675317	0.228715	0.196753	Biso	1.000000	H
H1836	1.0	0.683252	0.214593	0.161043	Biso	1.000000	H
O2090	1.0	0.860770	0.879420	0.792368	Biso	1.000000	O
H1837	1.0	0.882800	0.889570	0.786530	Biso	1.000000	H
H1838	1.0	0.862953	0.864514	0.811941	Biso	1.000000	H
O2091	1.0	0.553103	0.034800	0.260818	Biso	1.000000	O
H1839	1.0	0.567869	0.013379	0.258073	Biso	1.000000	H
H1840	1.0	0.533740	0.027900	0.274131	Biso	1.000000	H
O2092	1.0	0.382979	0.499514	0.998611	Biso	1.000000	O
H1841	1.0	0.370418	0.522712	0.997156	Biso	1.000000	H
H1842	1.0	0.382054	0.490238	0.020757	Biso	1.000000	H
O2093	1.0	0.777608	0.360800	0.771081	Biso	1.000000	O
H1843	1.0	0.770027	0.342004	0.755755	Biso	1.000000	H
H1844	1.0	0.796947	0.351405	0.783215	Biso	1.000000	H
O2094	1.0	0.196641	0.856732	0.176529	Biso	1.000000	O
H1845	1.0	0.175116	0.862790	0.186762	Biso	1.000000	H
H1846	1.0	0.213569	0.851908	0.193165	Biso	1.000000	H
O2095	1.0	0.362881	0.121108	0.607140	Biso	1.000000	O
H1847	1.0	0.366358	0.094353	0.606743	Biso	1.000000	H
H1848	1.0	0.367549	0.131354	0.585766	Biso	1.000000	H
O2096	1.0	0.476729	0.568304	0.216221	Biso	1.000000	O
H1849	1.0	0.500282	0.570634	0.209525	Biso	1.000000	H
H1850	1.0	0.462128	0.579464	0.199888	Biso	1.000000	H
O2097	1.0	0.031776	0.773979	0.128898	Biso	1.000000	O
H1851	1.0	0.054931	0.766835	0.134110	Biso	1.000000	H
H1852	1.0	0.029725	0.777798	0.105621	Biso	1.000000	H
O2098	1.0	0.593606	0.859233	0.406585	Biso	1.000000	O
H1853	1.0	0.591662	0.834199	0.415285	Biso	1.000000	H
H1854	1.0	0.591159	0.858484	0.383114	Biso	1.000000	H
O2099	1.0	0.332123	0.266868	0.371077	Biso	1.000000	O
H1855	1.0	0.345897	0.247107	0.361832	Biso	1.000000	H
H1856	1.0	0.313458	0.272533	0.356469	Biso	1.000000	H
O2100	1.0	0.415399	0.891659	0.224621	Biso	1.000000	O
H1857	1.0	0.401370	0.913814	0.223041	Biso	1.000000	H
H1858	1.0	0.410190	0.878582	0.244663	Biso	1.000000	H
O2101	1.0	0.375511	0.618397	0.201357	Biso	1.000000	O
H1859	1.0	0.373089	0.591754	0.204596	Biso	1.000000	H
H1860	1.0	0.353782	0.630486	0.204998	Biso	1.000000	H
O2102	1.0	0.268918	0.358424	0.245142	Biso	1.000000	O
H1861	1.0	0.259697	0.341734	0.228812	Biso	1.000000	H
H1862	1.0	0.250823	0.366598	0.259496	Biso	1.000000	H
O2103	1.0	0.371323	0.629624	0.679961	Biso	1.000000	O
H1863	1.0	0.363532	0.603982	0.680317	Biso	1.000000	H
H1864	1.0	0.362262	0.642082	0.660900	Biso	1.000000	H
O2104	1.0	0.887624	0.446494	0.936890	Biso	1.000000	O
H1865	1.0	0.864768	0.436737	0.934432	Biso	1.000000	H
H1866	1.0	0.889513	0.459295	0.957595	Biso	1.000000	H
O2105	1.0	0.738707	0.891205	0.165445	Biso	1.000000	O
H1867	1.0	0.756413	0.887264	0.181508	Biso	1.000000	H
H1868	1.0	0.717933	0.879153	0.172597	Biso	1.000000	H
O2106	1.0	0.293606	0.183023	0.506989	Biso	1.000000	O
H1869	1.0	0.290879	0.186613	0.530229	Biso	1.000000	H
H1870	1.0	0.311588	0.165083	0.503046	Biso	1.000000	H
O2107	1.0	0.102256	0.595606	0.993879	Biso	1.000000	O
H1871	1.0	0.086489	0.575153	0.997104	Biso	1.000000	H
H1872	1.0	0.115200	0.599620	0.013657	Biso	1.000000	H
O2108	1.0	0.226982	0.305097	0.009108	Biso	1.000000	O
H1873	1.0	0.249025	0.315784	0.014111	Biso	1.000000	H

H1874	1.0	0.226142	0.298426	0.986256	Biso	1.000000	H
O2109	1.0	0.919757	0.379642	0.602118	Biso	1.000000	O
H1875	1.0	0.920450	0.373079	0.579237	Biso	1.000000	H
H1876	1.0	0.914318	0.405884	0.604341	Biso	1.000000	H
O2110	1.0	0.490338	0.400632	0.807500	Biso	1.000000	O
H1877	1.0	0.509546	0.383680	0.807231	Biso	1.000000	H
H1878	1.0	0.494390	0.420179	0.823329	Biso	1.000000	H
O2111	1.0	0.197966	0.714946	0.830002	Biso	1.000000	O
H1879	1.0	0.210829	0.704445	0.847926	Biso	1.000000	H
H1880	1.0	0.204524	0.740750	0.826861	Biso	1.000000	H
O2112	1.0	0.855053	0.649109	0.835297	Biso	1.000000	O
H1881	1.0	0.854967	0.639736	0.857431	Biso	1.000000	H
H1882	1.0	0.875682	0.641117	0.824407	Biso	1.000000	H
O2113	1.0	0.467773	0.254294	0.568255	Biso	1.000000	O
H1883	1.0	0.475047	0.231657	0.557400	Biso	1.000000	H
H1884	1.0	0.474625	0.275735	0.555470	Biso	1.000000	H
O2114	1.0	0.533570	0.564293	0.736085	Biso	1.000000	O
H1885	1.0	0.525281	0.587903	0.744396	Biso	1.000000	H
H1886	1.0	0.516661	0.553479	0.721734	Biso	1.000000	H
O2115	1.0	0.326243	0.262930	0.825252	Biso	1.000000	O
H1887	1.0	0.333070	0.280025	0.842322	Biso	1.000000	H
H1888	1.0	0.311579	0.275773	0.809947	Biso	1.000000	H
O2116	1.0	0.948988	0.668873	0.068347	Biso	1.000000	O
H1889	1.0	0.939711	0.672394	0.089993	Biso	1.000000	H
H1890	1.0	0.933216	0.654334	0.055387	Biso	1.000000	H
O2117	1.0	0.161493	0.181884	0.935462	Biso	1.000000	O
H1891	1.0	0.182109	0.192185	0.926142	Biso	1.000000	H
H1892	1.0	0.158918	0.190738	0.957620	Biso	1.000000	H
O2118	1.0	0.531318	0.599779	0.466879	Biso	1.000000	O
H1893	1.0	0.549670	0.616883	0.471931	Biso	1.000000	H
H1894	1.0	0.518267	0.594787	0.486420	Biso	1.000000	H
O2119	1.0	0.481410	0.388350	0.483333	Biso	1.000000	O
H1895	1.0	0.478206	0.406095	0.500865	Biso	1.000000	H
H1896	1.0	0.502258	0.394129	0.471778	Biso	1.000000	H
O2120	1.0	0.660480	0.482926	0.257927	Biso	1.000000	O
H1897	1.0	0.643899	0.462928	0.258375	Biso	1.000000	H
H1898	1.0	0.654119	0.500954	0.241433	Biso	1.000000	H
O2121	1.0	0.101056	0.585924	0.132373	Biso	1.000000	O
H1899	1.0	0.097775	0.581716	0.155470	Biso	1.000000	H
H1900	1.0	0.115625	0.607434	0.129220	Biso	1.000000	H
O2122	1.0	0.809308	0.658131	0.958542	Biso	1.000000	O
H1901	1.0	0.829389	0.670902	0.966503	Biso	1.000000	H
H1902	1.0	0.814818	0.632276	0.954121	Biso	1.000000	H
O2123	1.0	0.349089	0.693433	0.908033	Biso	1.000000	O
H1903	1.0	0.336323	0.701004	0.927110	Biso	1.000000	H
H1904	1.0	0.340013	0.705888	0.888977	Biso	1.000000	H
O2124	1.0	0.276761	0.198328	0.125309	Biso	1.000000	O
H1905	1.0	0.252966	0.192075	0.122351	Biso	1.000000	H
H1906	1.0	0.282895	0.195155	0.147996	Biso	1.000000	H
O2125	1.0	0.402026	0.831942	0.188267	Biso	1.000000	O
H1907	1.0	0.412242	0.810170	0.198267	Biso	1.000000	H
H1908	1.0	0.406732	0.853738	0.201475	Biso	1.000000	H
O2126	1.0	0.430118	0.286133	0.210982	Biso	1.000000	O
H1909	1.0	0.441793	0.276146	0.192116	Biso	1.000000	H
H1910	1.0	0.446001	0.288331	0.228932	Biso	1.000000	H
O2127	1.0	0.319118	0.367198	0.419129	Biso	1.000000	O
H1911	1.0	0.312688	0.392883	0.423145	Biso	1.000000	H
H1912	1.0	0.298936	0.351664	0.419056	Biso	1.000000	H
O2128	1.0	0.018127	0.522669	0.583687	Biso	1.000000	O
H1913	1.0	0.033747	0.516445	0.566254	Biso	1.000000	H
H1914	1.0	0.000613	0.503711	0.585129	Biso	1.000000	H
O2129	1.0	0.174120	0.375747	0.347653	Biso	1.000000	O
H1915	1.0	0.190294	0.355351	0.348186	Biso	1.000000	H
H1916	1.0	0.151374	0.366146	0.351251	Biso	1.000000	H
O2130	1.0	0.679411	0.090129	0.505535	Biso	1.000000	O
H1917	1.0	0.668057	0.113788	0.502045	Biso	1.000000	H
H1918	1.0	0.703288	0.094477	0.510070	Biso	1.000000	H
O2131	1.0	0.725808	0.146331	0.433323	Biso	1.000000	O
H1919	1.0	0.722649	0.134906	0.412152	Biso	1.000000	H

H1920	1.0	0.707291	0.163516	0.437549	Biso	1.000000	H
O2132	1.0	0.095411	0.003090	0.252821	Biso	1.000000	O
H1921	1.0	0.108566	0.999066	0.272470	Biso	1.000000	H
H1922	1.0	0.071751	0.997095	0.256941	Biso	1.000000	H
O2133	1.0	0.499407	0.784552	0.069450	Biso	1.000000	O
H1923	1.0	0.490052	0.802235	0.054009	Biso	1.000000	H
H1924	1.0	0.485588	0.784396	0.088995	Biso	1.000000	H
O2134	1.0	0.718367	0.682646	0.418634	Biso	1.000000	O
H1925	1.0	0.708835	0.668618	0.400644	Biso	1.000000	H
H1926	1.0	0.702997	0.681541	0.437063	Biso	1.000000	H
O2135	1.0	0.247856	0.042148	0.820117	Biso	1.000000	O
H1927	1.0	0.264166	0.023856	0.812484	Biso	1.000000	H
H1928	1.0	0.225544	0.036316	0.811443	Biso	1.000000	H
O2136	1.0	0.408054	0.833101	0.830948	Biso	1.000000	O
H1929	1.0	0.385946	0.825633	0.822767	Biso	1.000000	H
H1930	1.0	0.411149	0.823355	0.852756	Biso	1.000000	H
O2137	1.0	0.858662	0.626930	0.896722	Biso	1.000000	O
H1931	1.0	0.873252	0.647436	0.903159	Biso	1.000000	H
H1932	1.0	0.869777	0.603493	0.901739	Biso	1.000000	H
O2138	1.0	0.199099	0.930780	0.892918	Biso	1.000000	O
H1933	1.0	0.205682	0.917085	0.912261	Biso	1.000000	H
H1934	1.0	0.200217	0.957311	0.897292	Biso	1.000000	H
H1935	1.0	0.872638	0.297010	0.520517	Biso	1.000000	H
H1936	1.0	0.960436	0.386107	0.600684	Biso	1.000000	H
H1937	1.0	0.041629	0.265581	0.524379	Biso	1.000000	H
O2139	1.0	0.665072	0.772690	0.085199	Biso	1.000000	O
H1938	1.0	0.676982	0.780441	0.104722	Biso	1.000000	H
H1939	1.0	0.659565	0.794415	0.072190	Biso	1.000000	H
O2140	1.0	0.356046	0.143182	0.378866	Biso	1.000000	O
H1940	1.0	0.371742	0.126752	0.367663	Biso	1.000000	H
H1941	1.0	0.361019	0.143033	0.401977	Biso	1.000000	H
O2141	1.0	0.863535	0.697180	0.040298	Biso	1.000000	O
H1942	1.0	0.878195	0.676300	0.045567	Biso	1.000000	H
H1943	1.0	0.860050	0.698174	0.016954	Biso	1.000000	H
O2142	1.0	0.462557	0.074343	0.361594	Biso	1.000000	O
H1944	1.0	0.476134	0.052161	0.357986	Biso	1.000000	H
H1945	1.0	0.459334	0.078003	0.384770	Biso	1.000000	H
O2143	1.0	0.708776	0.777222	0.268796	Biso	1.000000	O
H1946	1.0	0.731214	0.766046	0.267953	Biso	1.000000	H
H1947	1.0	0.693292	0.760567	0.280018	Biso	1.000000	H
O2144	1.0	0.598999	0.775395	0.231749	Biso	1.000000	O
H1948	1.0	0.617756	0.762505	0.221350	Biso	1.000000	H
H1949	1.0	0.581146	0.757686	0.236848	Biso	1.000000	H
O2145	1.0	0.506670	0.685201	0.011260	Biso	1.000000	O
H1950	1.0	0.506194	0.693396	0.033741	Biso	1.000000	H
H1951	1.0	0.494933	0.661529	0.009354	Biso	1.000000	H
O2146	1.0	0.108586	0.517866	0.961413	Biso	1.000000	O
H1952	1.0	0.093979	0.521620	0.980141	Biso	1.000000	H
H1953	1.0	0.101817	0.495330	0.950113	Biso	1.000000	H
O2147	1.0	0.794276	0.601401	0.888434	Biso	1.000000	O
H1954	1.0	0.814842	0.610729	0.898601	Biso	1.000000	H
H1955	1.0	0.800178	0.587278	0.869125	Biso	1.000000	H
O2148	1.0	0.238391	0.910714	0.844261	Biso	1.000000	O
H1956	1.0	0.220977	0.918763	0.859422	Biso	1.000000	H
H1957	1.0	0.227747	0.901554	0.824535	Biso	1.000000	H
O2149	1.0	0.329099	0.838996	0.875270	Biso	1.000000	O
H1958	1.0	0.306841	0.850029	0.878512	Biso	1.000000	H
H1959	1.0	0.345140	0.849951	0.890428	Biso	1.000000	H
O2150	1.0	0.523567	0.136850	0.433528	Biso	1.000000	O
H1960	1.0	0.533839	0.151831	0.450534	Biso	1.000000	H
H1961	1.0	0.536678	0.139381	0.413664	Biso	1.000000	H
O2151	1.0	0.812633	0.839196	0.160797	Biso	1.000000	O
H1962	1.0	0.797102	0.818322	0.158809	Biso	1.000000	H
H1963	1.0	0.805290	0.855216	0.178458	Biso	1.000000	H
O2152	1.0	0.334745	0.189324	0.258633	Biso	1.000000	O
H1964	1.0	0.354641	0.196976	0.270872	Biso	1.000000	H
H1965	1.0	0.341840	0.175913	0.239302	Biso	1.000000	H
O2153	1.0	0.692900	0.791534	0.439717	Biso	1.000000	O
H1966	1.0	0.695544	0.770619	0.454449	Biso	1.000000	H
H1967	1.0	0.693995	0.782742	0.417428	Biso	1.000000	H
O2154	1.0	0.313061	0.841523	0.600245	Biso	1.000000	O
H1968	1.0	0.309467	0.828047	0.620408	Biso	1.000000	H
H1969	1.0	0.291786	0.852557	0.593192	Biso	1.000000	H
O2155	1.0	0.319402	0.940846	0.124794	Biso	1.000000	O
H1970	1.0	0.329908	0.965114	0.122183	Biso	1.000000	H
H1971	1.0	0.336375	0.921553	0.121721	Biso	1.000000	H

O2156	1.0	0.227982	0.409111	0.684843	Biso	1.000000	O
H1972	1.0	0.225547	0.382547	0.681162	Biso	1.000000	H
H1973	1.0	0.235209	0.421114	0.664864	Biso	1.000000	H
O2157	1.0	0.416481	0.642733	0.407595	Biso	1.000000	O
H1974	1.0	0.409502	0.616891	0.405797	Biso	1.000000	H
H1975	1.0	0.434569	0.647698	0.392151	Biso	1.000000	H
O2158	1.0	0.911434	0.626038	0.805990	Biso	1.000000	O
H1976	1.0	0.903995	0.613980	0.786108	Biso	1.000000	H
H1977	1.0	0.933044	0.638336	0.802238	Biso	1.000000	H
O2159	1.0	0.475648	0.887459	0.195567	Biso	1.000000	O
H1978	1.0	0.453863	0.889510	0.206482	Biso	1.000000	H
H1979	1.0	0.488986	0.867930	0.205730	Biso	1.000000	H
O2160	1.0	0.071176	0.073202	0.436848	Biso	1.000000	O
H1980	1.0	0.061803	0.062491	0.417126	Biso	1.000000	H
H1981	1.0	0.068176	0.055880	0.454732	Biso	1.000000	H
O2161	1.0	0.529839	0.166484	0.170917	Biso	1.000000	O
H1982	1.0	0.547268	0.149417	0.163385	Biso	1.000000	H
H1983	1.0	0.521638	0.181213	0.152754	Biso	1.000000	H
O2162	1.0	0.378246	0.447932	0.174308	Biso	1.000000	O
H1984	1.0	0.376601	0.473845	0.167779	Biso	1.000000	H
H1985	1.0	0.387975	0.433453	0.156691	Biso	1.000000	H
O2163	1.0	0.800316	0.068929	0.843683	Biso	1.000000	O
H1986	1.0	0.821578	0.069033	0.831729	Biso	1.000000	H
H1987	1.0	0.781614	0.064778	0.828735	Biso	1.000000	H
O2164	1.0	0.410875	0.391529	0.877974	Biso	1.000000	O
H1988	1.0	0.397417	0.390307	0.897718	Biso	1.000000	H
H1989	1.0	0.430959	0.376082	0.880376	Biso	1.000000	H
O2165	1.0	0.601439	0.769698	0.004580	Biso	1.000000	O
H1990	1.0	0.622343	0.783788	0.002250	Biso	1.000000	H
H1991	1.0	0.592760	0.772636	0.026518	Biso	1.000000	H
O2166	1.0	0.981924	0.121579	0.719167	Biso	1.000000	O
H1992	1.0	0.977466	0.118140	0.742180	Biso	1.000000	H
H1993	1.0	0.970162	0.102473	0.706840	Biso	1.000000	H
O2167	1.0	0.345079	0.807594	0.819863	Biso	1.000000	O
H1994	1.0	0.340311	0.820006	0.840323	Biso	1.000000	H
H1995	1.0	0.323893	0.802445	0.808652	Biso	1.000000	H
O2168	1.0	0.300867	0.799880	0.236421	Biso	1.000000	O
H1996	1.0	0.289826	0.776090	0.240140	Biso	1.000000	H
H1997	1.0	0.284587	0.817333	0.227367	Biso	1.000000	H
O2169	1.0	0.597389	0.948535	0.373954	Biso	1.000000	O
H1998	1.0	0.590819	0.939743	0.352544	Biso	1.000000	H
H1999	1.0	0.585580	0.971633	0.378708	Biso	1.000000	H
O2170	1.0	0.540907	0.735195	0.736704	Biso	1.000000	O
H2000	1.0	0.536291	0.752002	0.718758	Biso	1.000000	H
H2001	1.0	0.562443	0.741769	0.746666	Biso	1.000000	H
O2171	1.0	0.993586	0.585903	0.008345	Biso	1.000000	O
H2002	1.0	0.002097	0.583446	0.030388	Biso	1.000000	H
H2003	1.0	0.999673	0.610218	0.999840	Biso	1.000000	H
O2172	1.0	0.636381	0.101275	0.137593	Biso	1.000000	O
H2004	1.0	0.630472	0.097478	0.114925	Biso	1.000000	H
H2005	1.0	0.652985	0.121167	0.139279	Biso	1.000000	H
O2173	1.0	0.377982	0.439097	0.238948	Biso	1.000000	O
H2006	1.0	0.356762	0.427907	0.245956	Biso	1.000000	H
H2007	1.0	0.377859	0.441848	0.215474	Biso	1.000000	H
O2174	1.0	0.901208	0.761127	0.103892	Biso	1.000000	O
H2008	1.0	0.903184	0.777760	0.122395	Biso	1.000000	H
H2009	1.0	0.881941	0.744645	0.107038	Biso	1.000000	H
O2175	1.0	0.379030	0.387758	0.821790	Biso	1.000000	O
H2010	1.0	0.356737	0.399102	0.823742	Biso	1.000000	H
H2011	1.0	0.389887	0.387137	0.842971	Biso	1.000000	H
O2176	1.0	0.358143	0.894240	0.599728	Biso	1.000000	O
H2012	1.0	0.379982	0.887494	0.590476	Biso	1.000000	H
H2013	1.0	0.342861	0.873069	0.598529	Biso	1.000000	H
O2177	1.0	0.440449	0.615523	0.506038	Biso	1.000000	O
H2014	1.0	0.422146	0.624094	0.519972	Biso	1.000000	H
H2015	1.0	0.443914	0.633279	0.488557	Biso	1.000000	H
O2178	1.0	0.796244	0.933178	0.402454	Biso	1.000000	O
H2016	1.0	0.789671	0.910882	0.414216	Biso	1.000000	H
H2017	1.0	0.777406	0.941505	0.389070	Biso	1.000000	H
O2179	1.0	0.827825	0.414784	0.922253	Biso	1.000000	O

H2018	1.0	0.822895	0.388318	0.921423	Biso	1.000000	H
H2019	1.0	0.826114	0.425258	0.900562	Biso	1.000000	H
O2180	1.0	0.035285	0.567131	0.860609	Biso	1.000000	O
H2020	1.0	0.031833	0.549014	0.843412	Biso	1.000000	H
H2021	1.0	0.044883	0.589733	0.851491	Biso	1.000000	H
O2181	1.0	0.508483	0.603071	0.407128	Biso	1.000000	O
H2022	1.0	0.528559	0.606085	0.393676	Biso	1.000000	H
H2023	1.0	0.514989	0.603504	0.429887	Biso	1.000000	H
O2182	1.0	0.938660	0.200132	0.023155	Biso	1.000000	O
H2024	1.0	0.942645	0.226088	0.017791	Biso	1.000000	H
H2025	1.0	0.956690	0.191253	0.037267	Biso	1.000000	H
O2183	1.0	0.516538	0.481480	0.488506	Biso	1.000000	O
H2026	1.0	0.511585	0.485229	0.511387	Biso	1.000000	H
H2027	1.0	0.538720	0.469955	0.486228	Biso	1.000000	H
O2184	1.0	0.256172	0.501287	0.514658	Biso	1.000000	O
H2028	1.0	0.279238	0.508701	0.519941	Biso	1.000000	H
H2029	1.0	0.256148	0.475742	0.506949	Biso	1.000000	H
O2185	1.0	0.928430	0.702899	0.963847	Biso	1.000000	O
H2030	1.0	0.948168	0.692563	0.952951	Biso	1.000000	H
H2031	1.0	0.935043	0.712302	0.985046	Biso	1.000000	H
O2186	1.0	0.559518	0.440116	0.296632	Biso	1.000000	O
H2032	1.0	0.551370	0.414614	0.295958	Biso	1.000000	H
H2033	1.0	0.540412	0.457063	0.294709	Biso	1.000000	H
O2187	1.0	0.336516	0.382791	0.520406	Biso	1.000000	O
H2034	1.0	0.343000	0.369611	0.500759	Biso	1.000000	H
H2035	1.0	0.312178	0.380515	0.523662	Biso	1.000000	H
O2188	1.0	0.092419	0.565083	0.195399	Biso	1.000000	O
H2036	1.0	0.081671	0.579178	0.212708	Biso	1.000000	H
H2037	1.0	0.116925	0.566380	0.197800	Biso	1.000000	H
O2189	1.0	0.920325	0.090311	0.436319	Biso	1.000000	O
H2038	1.0	0.918707	0.090945	0.459859	Biso	1.000000	H
H2039	1.0	0.943344	0.096449	0.429757	Biso	1.000000	H
O2190	1.0	0.694779	0.589627	0.242962	Biso	1.000000	O
H2040	1.0	0.678965	0.599204	0.259027	Biso	1.000000	H
H2041	1.0	0.693773	0.604942	0.223543	Biso	1.000000	H
O2191	1.0	0.738004	0.545544	0.024083	Biso	1.000000	O
H2042	1.0	0.732894	0.567648	0.036751	Biso	1.000000	H
H2043	1.0	0.732672	0.523512	0.036764	Biso	1.000000	H
O2192	1.0	0.147734	0.394743	0.696620	Biso	1.000000	O
H2044	1.0	0.157283	0.395470	0.718367	Biso	1.000000	H
H2045	1.0	0.147321	0.419722	0.687616	Biso	1.000000	H
O2193	1.0	0.571554	0.112825	0.153616	Biso	1.000000	O
H2046	1.0	0.566895	0.096286	0.171740	Biso	1.000000	H
H2047	1.0	0.595764	0.112875	0.149109	Biso	1.000000	H
O2194	1.0	0.778018	0.495359	0.897617	Biso	1.000000	O
H2048	1.0	0.763424	0.516555	0.902025	Biso	1.000000	H
H2049	1.0	0.799800	0.498989	0.908219	Biso	1.000000	H
O2195	1.0	0.961329	0.046832	0.791467	Biso	1.000000	O
H2050	1.0	0.960281	0.035019	0.770267	Biso	1.000000	H
H2051	1.0	0.964974	0.073385	0.788779	Biso	1.000000	H
O2196	1.0	0.999700	0.345669	0.771065	Biso	1.000000	O
H2052	1.0	0.003109	0.322373	0.782578	Biso	1.000000	H
H2053	1.0	0.019356	0.350805	0.757537	Biso	1.000000	H
O2197	1.0	0.172549	0.290882	0.814936	Biso	1.000000	O
H2054	1.0	0.176586	0.266728	0.824792	Biso	1.000000	H
H2055	1.0	0.169225	0.309359	0.831859	Biso	1.000000	H
O2198	1.0	0.506670	0.318024	0.393409	Biso	1.000000	O
H2056	1.0	0.524955	0.324038	0.408346	Biso	1.000000	H
H2057	1.0	0.485627	0.315122	0.405455	Biso	1.000000	H
O2199	1.0	0.380364	0.653318	0.015695	Biso	1.000000	O
H2058	1.0	0.396632	0.647101	0.998810	Biso	1.000000	H
H2059	1.0	0.380929	0.634360	0.032505	Biso	1.000000	H
O2200	1.0	0.577927	0.621264	0.782120	Biso	1.000000	O
H2060	1.0	0.598116	0.606117	0.779140	Biso	1.000000	H
H2061	1.0	0.575204	0.637765	0.763616	Biso	1.000000	H
O2201	1.0	0.899844	0.842902	0.401923	Biso	1.000000	O
H2062	1.0	0.916906	0.846278	0.385141	Biso	1.000000	H
H2063	1.0	0.877374	0.845516	0.392470	Biso	1.000000	H
O2202	1.0	0.877514	0.548090	0.395032	Biso	1.000000	O
H2064	1.0	0.863609	0.536465	0.378397	Biso	1.000000	H
H2065	1.0	0.894922	0.530743	0.402109	Biso	1.000000	H
O2203	1.0	0.132645	0.840140	0.839547	Biso	1.000000	O
H2066	1.0	0.110260	0.847764	0.832213	Biso	1.000000	H
H2067	1.0	0.139908	0.856149	0.857247	Biso	1.000000	H
O2204	1.0	0.156338	0.936232	0.179026	Biso	1.000000	O
H2068	1.0	0.148557	0.912725	0.187985	Biso	1.000000	H
H2069	1.0	0.155163	0.935278	0.155471	Biso	1.000000	H
O2205	1.0	0.094413	0.394643	0.434679	Biso	1.000000	O
H2070	1.0	0.084256	0.374814	0.421923	Biso	1.000000	H
H2071	1.0	0.116812	0.400343	0.426147	Biso	1.000000	H

O2206	1.0	0.472420	0.618829	0.807555	Biso	1.000000	O
H2072	1.0	0.487943	0.612483	0.790074	Biso	1.000000	H
H2073	1.0	0.467870	0.645387	0.807123	Biso	1.000000	H
O2207	1.0	0.106270	0.194222	0.479699	Biso	1.000000	O
H2074	1.0	0.127947	0.194337	0.468446	Biso	1.000000	H
H2075	1.0	0.093227	0.172593	0.473026	Biso	1.000000	H
O2208	1.0	0.267330	0.738428	0.248561	Biso	1.000000	O
H2076	1.0	0.283672	0.719208	0.254114	Biso	1.000000	H
H2077	1.0	0.249797	0.727946	0.234718	Biso	1.000000	H
O2209	1.0	0.135064	0.774838	0.300370	Biso	1.000000	O
H2078	1.0	0.119467	0.793829	0.308067	Biso	1.000000	H
H2079	1.0	0.124756	0.750464	0.302975	Biso	1.000000	H
O2210	1.0	0.448415	0.800532	0.267531	Biso	1.000000	O
H2080	1.0	0.430998	0.818890	0.272245	Biso	1.000000	H
H2081	1.0	0.442903	0.787761	0.247415	Biso	1.000000	H
O2211	1.0	0.358609	0.635116	0.867200	Biso	1.000000	O
H2082	1.0	0.357321	0.655715	0.882427	Biso	1.000000	H
H2083	1.0	0.374993	0.640914	0.850302	Biso	1.000000	H
O2212	1.0	0.503673	0.756059	0.217374	Biso	1.000000	O
H2084	1.0	0.484646	0.738906	0.218450	Biso	1.000000	H
H2085	1.0	0.523426	0.744772	0.227502	Biso	1.000000	H
O2213	1.0	0.676637	0.683119	0.249385	Biso	1.000000	O
H2086	1.0	0.659136	0.665448	0.243186	Biso	1.000000	H
H2087	1.0	0.697744	0.676805	0.238493	Biso	1.000000	H
O2214	1.0	0.295116	0.226195	0.874803	Biso	1.000000	O
H2088	1.0	0.309480	0.234084	0.856900	Biso	1.000000	H
H2089	1.0	0.291512	0.199495	0.873554	Biso	1.000000	H
O2215	1.0	0.410979	0.303880	0.873486	Biso	1.000000	O
H2090	1.0	0.417779	0.280674	0.863285	Biso	1.000000	H
H2091	1.0	0.430522	0.320252	0.875208	Biso	1.000000	H
O2216	1.0	0.774752	0.476483	0.512534	Biso	1.000000	O
H2092	1.0	0.762117	0.492559	0.527154	Biso	1.000000	H
H2093	1.0	0.776930	0.451916	0.522152	Biso	1.000000	H
O2217	1.0	0.978577	0.960100	0.959920	Biso	1.000000	O
H2094	1.0	0.988439	0.935964	0.964800	Biso	1.000000	H
H2095	1.0	0.985591	0.967909	0.938349	Biso	1.000000	H
O2218	1.0	0.749426	0.646314	0.986745	Biso	1.000000	O
H2096	1.0	0.750634	0.655783	0.008816	Biso	1.000000	H
H2097	1.0	0.771087	0.650185	0.975983	Biso	1.000000	H
O2219	1.0	0.486318	0.912028	0.397210	Biso	1.000000	O
H2098	1.0	0.509028	0.911806	0.406415	Biso	1.000000	H
H2099	1.0	0.475644	0.887994	0.400710	Biso	1.000000	H
O2220	1.0	0.172611	0.067144	0.139456	Biso	1.000000	O
H2100	1.0	0.191642	0.050117	0.141534	Biso	1.000000	H
H2101	1.0	0.151530	0.053116	0.139261	Biso	1.000000	H
O2221	1.0	0.897388	0.123265	0.834662	Biso	1.000000	O
H2102	1.0	0.877810	0.134378	0.845241	Biso	1.000000	H
H2103	1.0	0.911552	0.142750	0.825509	Biso	1.000000	H
O2222	1.0	0.549917	0.927310	0.527683	Biso	1.000000	O
H2104	1.0	0.531138	0.943324	0.533894	Biso	1.000000	H
H2105	1.0	0.559420	0.915682	0.546947	Biso	1.000000	H
O2223	1.0	0.012284	0.248507	0.013277	Biso	1.000000	O
H2106	1.0	0.012590	0.224463	0.002502	Biso	1.000000	H
H2107	1.0	0.028045	0.265221	0.002484	Biso	1.000000	H
O2224	1.0	0.144450	0.127069	0.551586	Biso	1.000000	O
H2108	1.0	0.132330	0.150601	0.551077	Biso	1.000000	H
H2109	1.0	0.129009	0.107354	0.558089	Biso	1.000000	H
O2225	1.0	0.873832	0.180422	0.919614	Biso	1.000000	O
H2110	1.0	0.889839	0.197910	0.910172	Biso	1.000000	H
H2111	1.0	0.858681	0.193550	0.934288	Biso	1.000000	H
O2226	1.0	0.049905	0.120989	0.706698	Biso	1.000000	O
H2112	1.0	0.057046	0.097873	0.696556	Biso	1.000000	H
H2113	1.0	0.025763	0.119849	0.711418	Biso	1.000000	H
O2227	1.0	0.486860	0.313119	0.534481	Biso	1.000000	O
H2114	1.0	0.488646	0.338009	0.543519	Biso	1.000000	H
H2115	1.0	0.489872	0.314224	0.511080	Biso	1.000000	H
O2228	1.0	0.650642	0.609307	0.291682	Biso	1.000000	O
H2116	1.0	0.647431	0.630722	0.305745	Biso	1.000000	H
H2117	1.0	0.628691	0.600355	0.284287	Biso	1.000000	H
O2229	1.0	0.453456	0.882110	0.093655	Biso	1.000000	O
H2118	1.0	0.436251	0.867130	0.104374	Biso	1.000000	H
H2119	1.0	0.472390	0.886069	0.108377	Biso	1.000000	H
O2230	1.0	0.674538	0.957388	0.431239	Biso	1.000000	O
H2120	1.0	0.657022	0.974106	0.439172	Biso	1.000000	H
H2121	1.0	0.692895	0.956133	0.446959	Biso	1.000000	H
O2231	1.0	0.081486	0.940978	0.843103	Biso	1.000000	O
H2122	1.0	0.062327	0.926675	0.851156	Biso	1.000000	H
H2123	1.0	0.086743	0.960798	0.858342	Biso	1.000000	H
O2232	1.0	0.378039	0.313532	0.072531	Biso	1.000000	O
H2124	1.0	0.386281	0.311346	0.094689	Biso	1.000000	H
H2125	1.0	0.357948	0.329204	0.072176	Biso	1.000000	H
O2233	1.0	0.997185	0.262798	0.345730	Biso	1.000000	O
H2126	1.0	0.009167	0.283614	0.355482	Biso	1.000000	H
H2127	1.0	0.982435	0.271857	0.328551	Biso	1.000000	H

O2234	1.0	0.554174	0.686952	0.807545	Biso	1.000000	O
H2128	1.0	0.576452	0.698326	0.809504	Biso	1.000000	H
H2129	1.0	0.556704	0.660875	0.801828	Biso	1.000000	H
O2235	1.0	0.277117	0.845667	0.161216	Biso	1.000000	O
H2130	1.0	0.263919	0.840345	0.180603	Biso	1.000000	H
H2131	1.0	0.299785	0.835380	0.163616	Biso	1.000000	H
O2236	1.0	0.138452	0.149551	0.282774	Biso	1.000000	O
H2132	1.0	0.152296	0.132792	0.295707	Biso	1.000000	H
H2133	1.0	0.117073	0.137514	0.277484	Biso	1.000000	H
O2237	1.0	0.121099	0.238970	0.129825	Biso	1.000000	O
H2134	1.0	0.142519	0.232996	0.119355	Biso	1.000000	H
H2135	1.0	0.107006	0.253199	0.114969	Biso	1.000000	H
O2238	1.0	0.546119	0.907817	0.426465	Biso	1.000000	O
H2136	1.0	0.563817	0.892716	0.416656	Biso	1.000000	H
H2137	1.0	0.554892	0.932580	0.430819	Biso	1.000000	H
H2138	1.0	0.079716	0.651347	0.467129	Biso	1.000000	H
H2139	1.0	0.983432	0.565015	0.504577	Biso	1.000000	H
H2140	1.0	0.229425	0.532135	0.532820	Biso	1.000000	H
H2141	1.0	0.092960	0.545430	0.480438	Biso	1.000000	H
O2239	1.0	0.686327	0.437205	0.579230	Biso	1.000000	O
H2142	1.0	0.671721	0.458728	0.576325	Biso	1.000000	H
H2143	1.0	0.681459	0.418952	0.562462	Biso	1.000000	H
O2240	1.0	0.019003	0.074778	0.196701	Biso	1.000000	O
H2144	1.0	0.037346	0.073570	0.180963	Biso	1.000000	H
H2145	1.0	0.998032	0.066494	0.186604	Biso	1.000000	H
O2241	1.0	0.955933	0.277011	0.416340	Biso	1.000000	O
H2146	1.0	0.971328	0.290201	0.430736	Biso	1.000000	H
H2147	1.0	0.932703	0.280801	0.423542	Biso	1.000000	H
O2242	1.0	0.581533	0.535708	0.516390	Biso	1.000000	O
H2148	1.0	0.575022	0.511036	0.523747	Biso	1.000000	H
H2149	1.0	0.604075	0.541592	0.524467	Biso	1.000000	H
O2243	1.0	0.525683	0.432069	0.390912	Biso	1.000000	O
H2150	1.0	0.534128	0.457327	0.388612	Biso	1.000000	H
H2151	1.0	0.501069	0.432131	0.389402	Biso	1.000000	H
O2244	1.0	0.914394	0.676522	0.123829	Biso	1.000000	O
H2152	1.0	0.891316	0.685930	0.122466	Biso	1.000000	H
H2153	1.0	0.929138	0.695736	0.132568	Biso	1.000000	H
O2245	1.0	0.468521	0.237454	0.079423	Biso	1.000000	O
H2154	1.0	0.445816	0.234135	0.088170	Biso	1.000000	H
H2155	1.0	0.473315	0.263848	0.077230	Biso	1.000000	H
O2246	1.0	0.826308	0.782617	0.610504	Biso	1.000000	O
H2156	1.0	0.818769	0.806184	0.601474	Biso	1.000000	H
H2157	1.0	0.809730	0.773803	0.626189	Biso	1.000000	H
O2247	1.0	0.422021	0.768116	0.215599	Biso	1.000000	O
H2158	1.0	0.397907	0.763851	0.218873	Biso	1.000000	H
H2159	1.0	0.433361	0.744491	0.211911	Biso	1.000000	H
O2248	1.0	0.832635	0.696801	0.498848	Biso	1.000000	O
H2160	1.0	0.838968	0.679319	0.481903	Biso	1.000000	H
H2161	1.0	0.828632	0.683395	0.518976	Biso	1.000000	H
O2249	1.0	0.297264	0.436756	0.433306	Biso	1.000000	O
H2162	1.0	0.288099	0.434907	0.455152	Biso	1.000000	H
H2163	1.0	0.285164	0.455960	0.421402	Biso	1.000000	H
O2250	1.0	0.327025	0.719819	0.851600	Biso	1.000000	O
H2164	1.0	0.310451	0.702137	0.843412	Biso	1.000000	H
H2165	1.0	0.342713	0.726422	0.834330	Biso	1.000000	H
O2251	1.0	0.023599	0.106863	0.319915	Biso	1.000000	O
H2166	1.0	0.016963	0.117526	0.299183	Biso	1.000000	H
H2167	1.0	0.046438	0.096840	0.318247	Biso	1.000000	H
O2252	1.0	0.170859	0.622945	0.299934	Biso	1.000000	O
H2168	1.0	0.158011	0.636606	0.316166	Biso	1.000000	H
H2169	1.0	0.194845	0.623716	0.305384	Biso	1.000000	H
O2253	1.0	0.398023	0.345829	0.191917	Biso	1.000000	O
H2170	1.0	0.413507	0.358430	0.177209	Biso	1.000000	H
H2171	1.0	0.409314	0.324414	0.201431	Biso	1.000000	H
O2254	1.0	0.025622	0.512171	0.647551	Biso	1.000000	O
H2172	1.0	0.023824	0.516423	0.624313	Biso	1.000000	H
H2173	1.0	0.003306	0.514336	0.657422	Biso	1.000000	H
O2255	1.0	0.004407	0.467423	0.052202	Biso	1.000000	O
H2174	1.0	0.026288	0.479215	0.055739	Biso	1.000000	H
H2175	1.0	0.003780	0.456692	0.030553	Biso	1.000000	H
O2256	1.0	0.870591	0.081954	0.278928	Biso	1.000000	O
H2176	1.0	0.850528	0.092419	0.268687	Biso	1.000000	H
H2177	1.0	0.872019	0.090621	0.301238	Biso	1.000000	H
O2257	1.0	0.468082	0.370728	0.360456	Biso	1.000000	O
H2178	1.0	0.463874	0.391183	0.375346	Biso	1.000000	H
H2179	1.0	0.485198	0.354324	0.369598	Biso	1.000000	H
O2258	1.0	0.736837	0.557506	0.662459	Biso	1.000000	O
H2180	1.0	0.740660	0.556714	0.685761	Biso	1.000000	H
H2181	1.0	0.752721	0.574797	0.652558	Biso	1.000000	H

O2259	1.0	0.977346	0.988322	0.018197	Biso	1.000000	O
H2182	1.0	0.975054	0.977184	0.996810	Biso	1.000000	H
H2183	1.0	0.961388	0.976699	0.033054	Biso	1.000000	H
O2260	1.0	0.342541	0.316332	0.872589	Biso	1.000000	O
H2184	1.0	0.367066	0.313492	0.872693	Biso	1.000000	H
H2185	1.0	0.334641	0.321176	0.894539	Biso	1.000000	H
O2261	1.0	0.494639	0.917071	0.852761	Biso	1.000000	O
H2186	1.0	0.507819	0.896932	0.862174	Biso	1.000000	H
H2187	1.0	0.474867	0.906900	0.841800	Biso	1.000000	H
O2262	1.0	0.259339	0.857762	0.303258	Biso	1.000000	O
H2188	1.0	0.265702	0.864636	0.325253	Biso	1.000000	H
H2189	1.0	0.261194	0.830969	0.300710	Biso	1.000000	H
O2263	1.0	0.998744	0.934743	0.778670	Biso	1.000000	O
H2190	1.0	0.991628	0.937686	0.756223	Biso	1.000000	H
H2191	1.0	0.989268	0.911882	0.787403	Biso	1.000000	H
O2264	1.0	0.054978	0.034765	0.381530	Biso	1.000000	O
H2192	1.0	0.060281	0.008372	0.381191	Biso	1.000000	H
H2193	1.0	0.033842	0.039085	0.369968	Biso	1.000000	H
O2265	1.0	0.066548	0.535240	0.009057	Biso	1.000000	O
H2194	1.0	0.048832	0.524202	0.995767	Biso	1.000000	H
H2195	1.0	0.066662	0.523502	0.030313	Biso	1.000000	H
O2266	1.0	0.922362	0.500579	0.908294	Biso	1.000000	O
H2196	1.0	0.946188	0.498433	0.914095	Biso	1.000000	H
H2197	1.0	0.909715	0.480296	0.918133	Biso	1.000000	H
O2267	1.0	0.351141	0.563123	0.745529	Biso	1.000000	O
H2198	1.0	0.348872	0.565037	0.722090	Biso	1.000000	H
H2199	1.0	0.342884	0.539008	0.752679	Biso	1.000000	H
O2268	1.0	0.564143	0.301572	0.623350	Biso	1.000000	O
H2200	1.0	0.555402	0.322054	0.610424	Biso	1.000000	H
H2201	1.0	0.584320	0.291482	0.613032	Biso	1.000000	H
O2269	1.0	0.360251	0.448981	0.541872	Biso	1.000000	O
H2202	1.0	0.351748	0.425650	0.533171	Biso	1.000000	H
H2203	1.0	0.380268	0.456568	0.529781	Biso	1.000000	H
O2270	1.0	0.250707	0.790626	0.625594	Biso	1.000000	O
H2204	1.0	0.234310	0.809530	0.631782	Biso	1.000000	H
H2205	1.0	0.272014	0.795123	0.636810	Biso	1.000000	H
O2271	1.0	0.983857	0.850838	0.534264	Biso	1.000000	O
H2206	1.0	0.965744	0.838694	0.522263	Biso	1.000000	H
H2207	1.0	0.979423	0.848816	0.557410	Biso	1.000000	H
O2272	1.0	0.376596	0.103670	0.954265	Biso	1.000000	O
H2208	1.0	0.375496	0.123308	0.938086	Biso	1.000000	H
H2209	1.0	0.396398	0.088236	0.950225	Biso	1.000000	H
O2273	1.0	0.312484	0.771152	0.894901	Biso	1.000000	O
H2210	1.0	0.317954	0.796415	0.888342	Biso	1.000000	H
H2211	1.0	0.317461	0.754178	0.877167	Biso	1.000000	H
O2274	1.0	0.578068	0.453270	0.477859	Biso	1.000000	O
H2212	1.0	0.596020	0.452124	0.494008	Biso	1.000000	H
H2213	1.0	0.586059	0.466906	0.458974	Biso	1.000000	H
O2275	1.0	0.860923	0.800897	0.980073	Biso	1.000000	O
H2214	1.0	0.880595	0.798164	0.966041	Biso	1.000000	H
H2215	1.0	0.866263	0.791391	0.001563	Biso	1.000000	H
O2276	1.0	0.459314	0.197619	0.476854	Biso	1.000000	O
H2216	1.0	0.478953	0.211694	0.469591	Biso	1.000000	H
H2217	1.0	0.453661	0.178874	0.460737	Biso	1.000000	H
O2277	1.0	0.454293	0.834691	0.043493	Biso	1.000000	O
H2218	1.0	0.456835	0.853311	0.060421	Biso	1.000000	H
H2219	1.0	0.431760	0.823790	0.044768	Biso	1.000000	H
O2278	1.0	0.986081	0.006542	0.101749	Biso	1.000000	O
H2220	1.0	0.962587	0.010026	0.095247	Biso	1.000000	H
H2221	1.0	0.997734	0.030362	0.101476	Biso	1.000000	H
O2279	1.0	0.553764	0.334012	0.438386	Biso	1.000000	O
H2222	1.0	0.562298	0.325103	0.459116	Biso	1.000000	H
H2223	1.0	0.549106	0.360500	0.439866	Biso	1.000000	H
O2280	1.0	0.649695	0.384510	0.164443	Biso	1.000000	O
H2224	1.0	0.645697	0.411146	0.163272	Biso	1.000000	H
H2225	1.0	0.646612	0.373579	0.143064	Biso	1.000000	H
O2281	1.0	0.140860	0.772027	0.864094	Biso	1.000000	O
H2226	1.0	0.139787	0.796999	0.855125	Biso	1.000000	H
H2227	1.0	0.124790	0.769833	0.881891	Biso	1.000000	H
O2282	1.0	0.976948	0.370163	0.013584	Biso	1.000000	O
H2228	1.0	0.998075	0.380712	0.021544	Biso	1.000000	H
H2229	1.0	0.960851	0.390042	0.009291	Biso	1.000000	H
O2283	1.0	0.137758	0.948535	0.808199	Biso	1.000000	O
H2230	1.0	0.118713	0.939967	0.821193	Biso	1.000000	H
H2231	1.0	0.143522	0.973847	0.814372	Biso	1.000000	H
O2284	1.0	0.524420	0.878980	0.734103	Biso	1.000000	O
H2232	1.0	0.514483	0.903689	0.733070	Biso	1.000000	H
H2233	1.0	0.545946	0.878891	0.722586	Biso	1.000000	H
O2285	1.0	0.450327	0.025925	0.191484	Biso	1.000000	O
H2234	1.0	0.456525	0.000472	0.186200	Biso	1.000000	H
H2235	1.0	0.454421	0.030442	0.214417	Biso	1.000000	H

O2286	1.0	0.430732	0.336685	0.042008	Biso	1.000000	O
H2236	1.0	0.411438	0.329241	0.055193	Biso	1.000000	H
H2237	1.0	0.451422	0.326468	0.051250	Biso	1.000000	H
O2287	1.0	0.905016	0.565423	0.842867	Biso	1.000000	O
H2238	1.0	0.912340	0.587870	0.831746	Biso	1.000000	H
H2239	1.0	0.902414	0.570564	0.865900	Biso	1.000000	H
O2288	1.0	0.136443	0.522115	0.045528	Biso	1.000000	O
H2240	1.0	0.129254	0.547376	0.050341	Biso	1.000000	H
H2241	1.0	0.132006	0.506295	0.064184	Biso	1.000000	H
H2242	1.0	0.258303	0.051162	0.468224	Biso	1.000000	H
H2243	1.0	0.714870	0.648604	0.198462	Biso	1.000000	H
H2244	1.0	0.720967	0.622813	0.968851	Biso	1.000000	H
H2245	1.0	0.722742	0.467660	0.167878	Biso	1.000000	H
H2246	1.0	0.945241	0.157598	0.349458	Biso	1.000000	H
H2247	1.0	0.995813	0.040247	0.502430	Biso	1.000000	H
H2248	1.0	0.632159	0.023199	0.456605	Biso	1.000000	H
H2249	1.0	0.089614	0.021181	0.489907	Biso	1.000000	H
H2250	1.0	0.270023	0.454546	0.990734	Biso	1.000000	H
O2289	1.0	0.179638	0.707445	0.739804	Biso	1.000000	O
H2251	1.0	0.184394	0.680935	0.739068	Biso	1.000000	H
H2252	1.0	0.171553	0.715656	0.718697	Biso	1.000000	H
O2290	1.0	0.510298	0.410295	0.224681	Biso	1.000000	O
H2253	1.0	0.530349	0.398981	0.215127	Biso	1.000000	H
H2254	1.0	0.504058	0.432258	0.212293	Biso	1.000000	H
O2291	1.0	0.532307	0.531178	0.443129	Biso	1.000000	O
H2255	1.0	0.526364	0.514398	0.460732	Biso	1.000000	H
H2256	1.0	0.534194	0.556374	0.451475	Biso	1.000000	H
O2292	1.0	0.108812	0.549730	0.543632	Biso	1.000000	O
H2257	1.0	0.088367	0.553860	0.530937	Biso	1.000000	H
H2258	1.0	0.102989	0.536954	0.563665	Biso	1.000000	H
O2293	1.0	0.558653	0.380647	0.188281	Biso	1.000000	O
H2259	1.0	0.576223	0.375779	0.204288	Biso	1.000000	H
H2260	1.0	0.568733	0.392148	0.169227	Biso	1.000000	H
O2294	1.0	0.286099	0.555750	0.167910	Biso	1.000000	O
H2261	1.0	0.279455	0.537286	0.151890	Biso	1.000000	H
H2262	1.0	0.286669	0.580175	0.157823	Biso	1.000000	H
O2295	1.0	0.553624	0.871820	0.484699	Biso	1.000000	O
H2263	1.0	0.547845	0.879688	0.462811	Biso	1.000000	H
H2264	1.0	0.548491	0.891740	0.499874	Biso	1.000000	H
O2296	1.0	0.814662	0.789315	0.075612	Biso	1.000000	O
H2265	1.0	0.808539	0.814722	0.081132	Biso	1.000000	H
H2266	1.0	0.797381	0.778744	0.061530	Biso	1.000000	H
O2297	1.0	0.488597	0.984887	0.117929	Biso	1.000000	O
H2267	1.0	0.494829	0.010218	0.123602	Biso	1.000000	H
H2268	1.0	0.477358	0.973200	0.136288	Biso	1.000000	H
O2298	1.0	0.903566	0.369801	0.094314	Biso	1.000000	O
H2269	1.0	0.911254	0.357454	0.074654	Biso	1.000000	H
H2270	1.0	0.923027	0.375928	0.107787	Biso	1.000000	H
O2299	1.0	0.504132	0.002324	0.453557	Biso	1.000000	O
H2271	1.0	0.504897	0.028808	0.458212	Biso	1.000000	H
H2272	1.0	0.489198	0.997886	0.435182	Biso	1.000000	H
O2300	1.0	0.419441	0.704996	0.314960	Biso	1.000000	O
H2273	1.0	0.417660	0.714321	0.337042	Biso	1.000000	H
H2274	1.0	0.414661	0.678481	0.314736	Biso	1.000000	H
O2301	1.0	0.298243	0.135349	0.616902	Biso	1.000000	O
H2275	1.0	0.286397	0.116786	0.604025	Biso	1.000000	H
H2276	1.0	0.322531	0.130811	0.615849	Biso	1.000000	H
O2302	1.0	0.203219	0.650677	0.132133	Biso	1.000000	O
H2277	1.0	0.180767	0.648209	0.122607	Biso	1.000000	H
H2278	1.0	0.214328	0.672594	0.123321	Biso	1.000000	H
O2303	1.0	0.719568	0.342850	0.949534	Biso	1.000000	O
H2279	1.0	0.701258	0.324876	0.951440	Biso	1.000000	H
H2280	1.0	0.733740	0.336488	0.931037	Biso	1.000000	H
O2304	1.0	0.783347	0.776360	0.910151	Biso	1.000000	O
H2281	1.0	0.766653	0.792326	0.920514	Biso	1.000000	H
H2282	1.0	0.793055	0.789139	0.891549	Biso	1.000000	H
O2305	1.0	0.995235	0.320998	0.582285	Biso	1.000000	O
H2283	1.0	0.995198	0.347488	0.586979	Biso	1.000000	H
H2284	1.0	0.008434	0.316368	0.562766	Biso	1.000000	H
O2306	1.0	0.919317	0.776960	0.837810	Biso	1.000000	O
H2285	1.0	0.931625	0.768679	0.818683	Biso	1.000000	H
H2286	1.0	0.931546	0.797329	0.847994	Biso	1.000000	H
O2307	1.0	0.341951	0.663856	0.632270	Biso	1.000000	O
H2287	1.0	0.346171	0.669073	0.609471	Biso	1.000000	H
H2288	1.0	0.318334	0.669062	0.637317	Biso	1.000000	H
O2308	1.0	0.196557	0.946962	0.460093	Biso	1.000000	O
H2289	1.0	0.202386	0.921139	0.464274	Biso	1.000000	H
H2290	1.0	0.193733	0.959979	0.480596	Biso	1.000000	H
O2309	1.0	0.472644	0.124425	0.122759	Biso	1.000000	O

H2291	1.0	0.495095	0.117850	0.114858	Biso	1.000000	H
H2292	1.0	0.459543	0.135397	0.105211	Biso	1.000000	H
O2310	1.0	0.100454	0.575840	0.909713	Biso	1.000000	O
H2293	1.0	0.109008	0.564631	0.889859	Biso	1.000000	H
H2294	1.0	0.079724	0.563280	0.916288	Biso	1.000000	H
O2311	1.0	0.352485	0.578821	0.906845	Biso	1.000000	O
H2295	1.0	0.356367	0.598274	0.890888	Biso	1.000000	H
H2296	1.0	0.370788	0.578937	0.922660	Biso	1.000000	H
O2312	1.0	0.488493	0.353927	0.675022	Biso	1.000000	O
H2297	1.0	0.470726	0.335342	0.672875	Biso	1.000000	H
H2298	1.0	0.492572	0.365522	0.654066	Biso	1.000000	H
O2313	1.0	0.610278	0.728960	0.084782	Biso	1.000000	O
H2299	1.0	0.631483	0.742615	0.086517	Biso	1.000000	H
H2300	1.0	0.593004	0.744920	0.075320	Biso	1.000000	H
O2314	1.0	0.675310	0.492676	0.407902	Biso	1.000000	O
H2301	1.0	0.657528	0.503041	0.394282	Biso	1.000000	H
H2302	1.0	0.681513	0.468171	0.399903	Biso	1.000000	H
O2315	1.0	0.279679	0.722260	0.622454	Biso	1.000000	O
H2303	1.0	0.265791	0.744307	0.625597	Biso	1.000000	H
H2304	1.0	0.282153	0.709245	0.642999	Biso	1.000000	H
O2316	1.0	0.059877	0.676411	0.444191	Biso	1.000000	O
H2305	1.0	0.039072	0.666678	0.453594	Biso	1.000000	H
H2306	1.0	0.057438	0.677981	0.420750	Biso	1.000000	H
O2317	1.0	0.052670	0.297542	0.985591	Biso	1.000000	O
H2307	1.0	0.038518	0.314997	0.973709	Biso	1.000000	H
H2308	1.0	0.067951	0.285082	0.970600	Biso	1.000000	H
O2318	1.0	0.587960	0.079780	0.388768	Biso	1.000000	O
H2309	1.0	0.596748	0.089704	0.409042	Biso	1.000000	H
H2310	1.0	0.573631	0.098291	0.378392	Biso	1.000000	H
O2319	1.0	0.005873	0.616459	0.796186	Biso	1.000000	O
H2311	1.0	0.012273	0.617129	0.773406	Biso	1.000000	H
H2312	1.0	0.026190	0.616702	0.809563	Biso	1.000000	H
O2320	1.0	0.783357	0.993407	0.732598	Biso	1.000000	O
H2313	1.0	0.802104	0.978578	0.740812	Biso	1.000000	H
H2314	1.0	0.762423	0.979240	0.734245	Biso	1.000000	H
O2321	1.0	0.578578	0.399235	0.129124	Biso	1.000000	O
H2315	1.0	0.572449	0.415914	0.111508	Biso	1.000000	H
H2316	1.0	0.600575	0.388137	0.124644	Biso	1.000000	H
O2322	1.0	0.465983	0.435261	0.533419	Biso	1.000000	O
H2317	1.0	0.444891	0.445908	0.525470	Biso	1.000000	H
H2318	1.0	0.480670	0.455025	0.541272	Biso	1.000000	H
O2323	1.0	0.674585	0.426009	0.329821	Biso	1.000000	O
H2319	1.0	0.685373	0.429199	0.350857	Biso	1.000000	H
H2320	1.0	0.652440	0.437905	0.330022	Biso	1.000000	H
O2324	1.0	0.810576	0.610034	0.067740	Biso	1.000000	O
H2321	1.0	0.832245	0.604239	0.077811	Biso	1.000000	H
H2322	1.0	0.806348	0.636603	0.069292	Biso	1.000000	H
O2325	1.0	0.703138	0.937481	0.053821	Biso	1.000000	O
H2323	1.0	0.723808	0.949276	0.061544	Biso	1.000000	H
H2324	1.0	0.693065	0.952638	0.036826	Biso	1.000000	H
O2326	1.0	0.129347	0.252284	0.615726	Biso	1.000000	O
H2325	1.0	0.135115	0.265916	0.635342	Biso	1.000000	H
H2326	1.0	0.120869	0.227786	0.621524	Biso	1.000000	H
O2327	1.0	0.411877	0.110569	0.169899	Biso	1.000000	O
H2327	1.0	0.398884	0.128687	0.157568	Biso	1.000000	H
H2328	1.0	0.400157	0.105077	0.190101	Biso	1.000000	H
O2328	1.0	0.011921	0.000596	0.804136	Biso	1.000000	O
H2329	1.0	0.006276	0.975410	0.797491	Biso	1.000000	H
H2330	1.0	0.991757	0.016101	0.802956	Biso	1.000000	H
O2329	1.0	0.890093	0.932364	0.732588	Biso	1.000000	O
H2331	1.0	0.900972	0.927542	0.753344	Biso	1.000000	H
H2332	1.0	0.904259	0.922820	0.715160	Biso	1.000000	H
O2330	1.0	0.208810	0.198540	0.677774	Biso	1.000000	O
H2333	1.0	0.207948	0.186174	0.656808	Biso	1.000000	H
H2334	1.0	0.188141	0.193235	0.689787	Biso	1.000000	H
O2331	1.0	0.568062	0.617091	0.376002	Biso	1.000000	O
H2335	1.0	0.574070	0.596452	0.361889	Biso	1.000000	H
H2336	1.0	0.581866	0.616188	0.395537	Biso	1.000000	H
O2332	1.0	0.568410	0.653301	0.215391	Biso	1.000000	O
H2337	1.0	0.555032	0.642652	0.232899	Biso	1.000000	H
H2338	1.0	0.558737	0.646004	0.194641	Biso	1.000000	H
O2333	1.0	0.450815	0.186196	0.153748	Biso	1.000000	O
H2339	1.0	0.459080	0.163298	0.144018	Biso	1.000000	H
H2340	1.0	0.441184	0.180829	0.174960	Biso	1.000000	H
O2334	1.0	0.598020	0.488356	0.424285	Biso	1.000000	O
H2341	1.0	0.579700	0.491854	0.408787	Biso	1.000000	H
H2342	1.0	0.603634	0.512066	0.434264	Biso	1.000000	H
O2335	1.0	0.926473	0.332212	0.038226	Biso	1.000000	O
H2343	1.0	0.947432	0.344158	0.031449	Biso	1.000000	H
H2344	1.0	0.907443	0.343494	0.026897	Biso	1.000000	H
O2336	1.0	0.969368	0.763124	0.449944	Biso	1.000000	O
H2345	1.0	0.987176	0.749458	0.438802	Biso	1.000000	H
H2346	1.0	0.949280	0.764122	0.436282	Biso	1.000000	H
O2337	1.0	0.807813	0.851643	0.590943	Biso	1.000000	O
H2347	1.0	0.822937	0.849046	0.572441	Biso	1.000000	H

H2348	1.0	0.820722	0.859741	0.609766	Biso	1.000000	H
O2338	1.0	0.344694	0.645055	0.105789	Biso	1.000000	O
H2349	1.0	0.340395	0.668081	0.094133	Biso	1.000000	H
H2350	1.0	0.357272	0.628084	0.091915	Biso	1.000000	H
H2351	1.0	0.870108	0.037586	0.273659	Biso	1.000000	H
H2352	1.0	0.839010	0.443932	0.596472	Biso	1.000000	H
H2353	1.0	0.820855	0.502306	0.587309	Biso	1.000000	H
H2354	1.0	0.953308	0.844037	0.471993	Biso	1.000000	H
H2355	1.0	0.911018	0.463831	0.618374	Biso	1.000000	H
H2356	1.0	0.004566	0.011686	0.458821	Biso	1.000000	H
H2357	1.0	0.738771	0.532489	0.630908	Biso	1.000000	H
O2339	1.0	0.592545	0.789296	0.429755	Biso	1.000000	O
H2358	1.0	0.604783	0.774590	0.413792	Biso	1.000000	H
H2359	1.0	0.602141	0.784961	0.451159	Biso	1.000000	H
O2340	1.0	0.836906	0.923558	0.220681	Biso	1.000000	O
H2360	1.0	0.836583	0.941713	0.203203	Biso	1.000000	H
H2361	1.0	0.834743	0.936270	0.241400	Biso	1.000000	H
O2341	1.0	0.812465	0.276768	0.565739	Biso	1.000000	O
H2362	1.0	0.818100	0.301075	0.574539	Biso	1.000000	H
H2363	1.0	0.826540	0.257950	0.576016	Biso	1.000000	H
O2342	1.0	0.267700	0.627048	0.479770	Biso	1.000000	O
H2364	1.0	0.246879	0.612745	0.481778	Biso	1.000000	H
H2365	1.0	0.282658	0.621253	0.497836	Biso	1.000000	H
O2343	1.0	0.569029	0.015322	0.154543	Biso	1.000000	O
H2366	1.0	0.564434	0.988796	0.153445	Biso	1.000000	H
H2367	1.0	0.587906	0.021349	0.140299	Biso	1.000000	H
O2344	1.0	0.862309	0.407000	0.133323	Biso	1.000000	O
H2368	1.0	0.842337	0.392275	0.138460	Biso	1.000000	H
H2369	1.0	0.876224	0.393707	0.117674	Biso	1.000000	H
O2345	1.0	0.076141	0.503990	0.254048	Biso	1.000000	O
H2370	1.0	0.088074	0.517066	0.271257	Biso	1.000000	H
H2371	1.0	0.052059	0.509658	0.255268	Biso	1.000000	H
O2346	1.0	0.337295	0.013282	0.656902	Biso	1.000000	O
H2372	1.0	0.350226	0.995471	0.669630	Biso	1.000000	H
H2373	1.0	0.323750	0.000120	0.640878	Biso	1.000000	H
O2347	1.0	0.519455	0.532448	0.333655	Biso	1.000000	O
H2374	1.0	0.504334	0.553706	0.331879	Biso	1.000000	H
H2375	1.0	0.511965	0.512979	0.318941	Biso	1.000000	H
O2348	1.0	0.467882	0.838029	0.731460	Biso	1.000000	O
H2376	1.0	0.489295	0.851408	0.732319	Biso	1.000000	H
H2377	1.0	0.456090	0.844180	0.711444	Biso	1.000000	H
O2349	1.0	0.085134	0.090888	0.346223	Biso	1.000000	O
H2378	1.0	0.104923	0.079197	0.336520	Biso	1.000000	H
H2379	1.0	0.075273	0.074234	0.362233	Biso	1.000000	H
O2350	1.0	0.919028	0.201352	0.413818	Biso	1.000000	O
H2380	1.0	0.908600	0.211628	0.433228	Biso	1.000000	H
H2381	1.0	0.901689	0.189840	0.400377	Biso	1.000000	H
O2351	1.0	0.920026	0.912020	0.785600	Biso	1.000000	O
H2382	1.0	0.938699	0.894548	0.787826	Biso	1.000000	H
H2383	1.0	0.919737	0.928688	0.804173	Biso	1.000000	H
O2352	1.0	0.193790	0.902211	0.728608	Biso	1.000000	O
H2384	1.0	0.205370	0.922390	0.717487	Biso	1.000000	H
H2385	1.0	0.191227	0.881269	0.713890	Biso	1.000000	H
O2353	1.0	0.111343	0.354346	0.355308	Biso	1.000000	O
H2386	1.0	0.104171	0.335820	0.339555	Biso	1.000000	H
H2387	1.0	0.091544	0.367103	0.363906	Biso	1.000000	H
O2354	1.0	0.367808	0.889643	0.110603	Biso	1.000000	O
H2388	1.0	0.381376	0.868282	0.116962	Biso	1.000000	H
H2389	1.0	0.361290	0.887218	0.087943	Biso	1.000000	H
O2355	1.0	0.970954	0.644201	0.242721	Biso	1.000000	O
H2390	1.0	0.955837	0.622856	0.243344	Biso	1.000000	H
H2391	1.0	0.991138	0.638928	0.255475	Biso	1.000000	H
O2356	1.0	0.177101	0.288382	0.577469	Biso	1.000000	O
H2392	1.0	0.162024	0.274543	0.591707	Biso	1.000000	H
H2393	1.0	0.174722	0.314838	0.581726	Biso	1.000000	H
O2357	1.0	0.828947	0.978015	0.832994	Biso	1.000000	O
H2394	1.0	0.841814	0.981971	0.852827	Biso	1.000000	H
H2395	1.0	0.834857	0.997058	0.817234	Biso	1.000000	H
O2358	1.0	0.948831	0.070781	0.682455	Biso	1.000000	O
H2396	1.0	0.957454	0.082150	0.662700	Biso	1.000000	H
H2397	1.0	0.951404	0.043944	0.681105	Biso	1.000000	H
O2359	1.0	0.245324	0.391472	0.899124	Biso	1.000000	O
H2398	1.0	0.236854	0.367498	0.891839	Biso	1.000000	H
H2399	1.0	0.254439	0.389061	0.920949	Biso	1.000000	H
O2360	1.0	0.334486	0.886821	0.207402	Biso	1.000000	O
H2400	1.0	0.316331	0.886164	0.223364	Biso	1.000000	H
H2401	1.0	0.334809	0.863565	0.195382	Biso	1.000000	H
O2361	1.0	0.142348	0.012912	0.931256	Biso	1.000000	O
H2402	1.0	0.125811	0.008434	0.914192	Biso	1.000000	H
H2403	1.0	0.142360	0.992078	0.946289	Biso	1.000000	H
O2362	1.0	0.414077	0.714527	0.911818	Biso	1.000000	O

H2404	1.0	0.421542	0.731057	0.929065	Biso	1.000000	H
H2405	1.0	0.389894	0.709862	0.914048	Biso	1.000000	H
O2363	1.0	0.465835	0.799391	0.361997	Biso	1.000000	O
H2406	1.0	0.489618	0.800602	0.355836	Biso	1.000000	H
H2407	1.0	0.458498	0.773590	0.362332	Biso	1.000000	H
O2364	1.0	0.781806	0.489543	0.671060	Biso	1.000000	O
H2408	1.0	0.780679	0.501803	0.650057	Biso	1.000000	H
H2409	1.0	0.764603	0.470215	0.672143	Biso	1.000000	H
O2365	1.0	0.398070	0.644093	0.815271	Biso	1.000000	O
H2410	1.0	0.382510	0.640425	0.797245	Biso	1.000000	H
H2411	1.0	0.414338	0.663386	0.809721	Biso	1.000000	H
O2366	1.0	0.778417	0.727854	0.757759	Biso	1.000000	O
H2412	1.0	0.786465	0.739350	0.737839	Biso	1.000000	H
H2413	1.0	0.781155	0.745156	0.775697	Biso	1.000000	H
O2367	1.0	0.651056	0.326839	0.688692	Biso	1.000000	O
H2414	1.0	0.671443	0.326403	0.701967	Biso	1.000000	H
H2415	1.0	0.651974	0.306764	0.672917	Biso	1.000000	H
O2368	1.0	0.571374	0.348534	0.673852	Biso	1.000000	O
H2416	1.0	0.579676	0.333320	0.691665	Biso	1.000000	H
H2417	1.0	0.569273	0.333342	0.654439	Biso	1.000000	H
O2369	1.0	0.420630	0.471626	0.348642	Biso	1.000000	O
H2418	1.0	0.423922	0.498222	0.345874	Biso	1.000000	H
H2419	1.0	0.434666	0.458265	0.333136	Biso	1.000000	H
O2370	1.0	0.568205	0.779076	0.061180	Biso	1.000000	O
H2420	1.0	0.543613	0.777052	0.061349	Biso	1.000000	H
H2421	1.0	0.574965	0.801926	0.071997	Biso	1.000000	H
O2371	1.0	0.433672	0.541743	0.131488	Biso	1.000000	O
H2422	1.0	0.435230	0.550195	0.109123	Biso	1.000000	H
H2423	1.0	0.446681	0.518972	0.134059	Biso	1.000000	H
O2372	1.0	0.183660	0.037220	0.604971	Biso	1.000000	O
H2424	1.0	0.194487	0.019008	0.618995	Biso	1.000000	H
H2425	1.0	0.169318	0.024517	0.589301	Biso	1.000000	H
O2373	1.0	0.144027	0.573994	0.260560	Biso	1.000000	O
H2426	1.0	0.155469	0.593079	0.273185	Biso	1.000000	H
H2427	1.0	0.148454	0.577970	0.237608	Biso	1.000000	H
O2374	1.0	0.503212	0.015407	0.360327	Biso	1.000000	O
H2428	1.0	0.526314	0.014008	0.368498	Biso	1.000000	H
H2429	1.0	0.488150	0.001913	0.374831	Biso	1.000000	H
O2375	1.0	0.943359	0.609901	0.940194	Biso	1.000000	O
H2430	1.0	0.954289	0.633889	0.937203	Biso	1.000000	H
H2431	1.0	0.960553	0.590635	0.941994	Biso	1.000000	H
O2376	1.0	0.624999	0.422658	0.411420	Biso	1.000000	O
H2432	1.0	0.629816	0.409146	0.431331	Biso	1.000000	H
H2433	1.0	0.617045	0.447513	0.416715	Biso	1.000000	H
O2377	1.0	0.905295	0.439005	0.845687	Biso	1.000000	O
H2434	1.0	0.904402	0.424961	0.825545	Biso	1.000000	H
H2435	1.0	0.903189	0.422087	0.863979	Biso	1.000000	H
O2378	1.0	0.546356	0.946749	0.683392	Biso	1.000000	O
H2436	1.0	0.537186	0.936610	0.663356	Biso	1.000000	H
H2437	1.0	0.528100	0.950217	0.698966	Biso	1.000000	H
O2379	1.0	0.882891	0.323936	0.550444	Biso	1.000000	O
H2438	1.0	0.896623	0.344456	0.542497	Biso	1.000000	H
H2439	1.0	0.865313	0.333469	0.564753	Biso	1.000000	H
O2380	1.0	0.375639	0.472942	0.058065	Biso	1.000000	O
H2440	1.0	0.361652	0.483129	0.075345	Biso	1.000000	H
H2441	1.0	0.364891	0.450770	0.049325	Biso	1.000000	H
O2381	1.0	0.912321	0.793845	0.937921	Biso	1.000000	O
H2442	1.0	0.930792	0.777752	0.931053	Biso	1.000000	H
H2443	1.0	0.920480	0.819276	0.939613	Biso	1.000000	H
O2382	1.0	0.617369	0.276603	0.591365	Biso	1.000000	O
H2444	1.0	0.630665	0.254953	0.597503	Biso	1.000000	H
H2445	1.0	0.613104	0.276060	0.568130	Biso	1.000000	H
O2383	1.0	0.373794	0.332206	0.269334	Biso	1.000000	O
H2446	1.0	0.389034	0.312338	0.275924	Biso	1.000000	H
H2447	1.0	0.350650	0.325684	0.275169	Biso	1.000000	H
O2384	1.0	0.373520	0.209909	0.357759	Biso	1.000000	O
H2448	1.0	0.364215	0.185648	0.363137	Biso	1.000000	H
H2449	1.0	0.392551	0.215322	0.372007	Biso	1.000000	H
O2385	1.0	0.016362	0.921242	0.391894	Biso	1.000000	O
H2450	1.0	0.015199	0.899078	0.405354	Biso	1.000000	H
H2451	1.0	0.998307	0.938259	0.398034	Biso	1.000000	H
O2386	1.0	0.048013	0.035652	0.758785	Biso	1.000000	O
H2452	1.0	0.035396	0.023461	0.776041	Biso	1.000000	H
H2453	1.0	0.036583	0.031568	0.738183	Biso	1.000000	H
O2387	1.0	0.410848	0.184249	0.817668	Biso	1.000000	O
H2454	1.0	0.390422	0.192153	0.806387	Biso	1.000000	H
H2455	1.0	0.419974	0.204965	0.830059	Biso	1.000000	H
O2388	1.0	0.311445	0.969368	0.903550	Biso	1.000000	O
H2456	1.0	0.304138	0.961143	0.882188	Biso	1.000000	H
H2457	1.0	0.332880	0.957544	0.908997	Biso	1.000000	H
H2458	1.0	0.740806	0.304629	0.900810	Biso	1.000000	H
H2459	1.0	0.143623	0.397478	0.495761	Biso	1.000000	H
H2460	1.0	0.260797	0.896335	0.594431	Biso	1.000000	H
H2461	1.0	0.244664	0.795283	0.587439	Biso	1.000000	H
H2462	1.0	0.422112	0.995517	0.641315	Biso	1.000000	H

H2463	1.0	0.849349	0.270962	0.064735	Biso	1.000000	H
H2464	1.0	0.108103	0.380680	0.468305	Biso	1.000000	H
H2465	1.0	0.246643	0.927880	0.689310	Biso	1.000000	H
O2389	1.0	0.071850	0.432258	0.238608	Biso	1.000000	O
H2466	1.0	0.074826	0.457879	0.245560	Biso	1.000000	H
H2467	1.0	0.073263	0.430934	0.215078	Biso	1.000000	H
O2390	1.0	0.267256	0.958835	0.263019	Biso	1.000000	O
H2468	1.0	0.284619	0.964495	0.279032	Biso	1.000000	H
H2469	1.0	0.262190	0.981044	0.250473	Biso	1.000000	H
O2391	1.0	0.150372	0.138910	0.641621	Biso	1.000000	O
H2470	1.0	0.171286	0.149273	0.632983	Biso	1.000000	H
H2471	1.0	0.132246	0.157086	0.639552	Biso	1.000000	H
O2392	1.0	0.774565	0.764308	0.333070	Biso	1.000000	O
H2472	1.0	0.790266	0.752710	0.348189	Biso	1.000000	H
H2473	1.0	0.774481	0.791050	0.336508	Biso	1.000000	H
O2393	1.0	0.975068	0.798878	0.988195	Biso	1.000000	O
H2474	1.0	0.981675	0.809673	0.967506	Biso	1.000000	H
H2475	1.0	0.989481	0.777625	0.992928	Biso	1.000000	H
O2394	1.0	0.094847	0.279674	0.082243	Biso	1.000000	O
H2476	1.0	0.070741	0.276576	0.078052	Biso	1.000000	H
H2477	1.0	0.107733	0.270849	0.063657	Biso	1.000000	H
O2395	1.0	0.488668	0.837558	0.808992	Biso	1.000000	O
H2478	1.0	0.477947	0.861033	0.814615	Biso	1.000000	H
H2479	1.0	0.512631	0.841672	0.804723	Biso	1.000000	H
O2396	1.0	0.473579	0.293280	0.259477	Biso	1.000000	O
H2480	1.0	0.476147	0.320073	0.261367	Biso	1.000000	H
H2481	1.0	0.495537	0.282074	0.255039	Biso	1.000000	H
O2397	1.0	0.048071	0.550768	0.109712	Biso	1.000000	O
H2482	1.0	0.069018	0.562758	0.116460	Biso	1.000000	H
H2483	1.0	0.033980	0.546052	0.128635	Biso	1.000000	H
O2398	1.0	0.981905	0.846508	0.598525	Biso	1.000000	O
H2484	1.0	0.980990	0.822793	0.609811	Biso	1.000000	H
H2485	1.0	0.977224	0.866503	0.613758	Biso	1.000000	H
O2399	1.0	0.372524	0.247720	0.045785	Biso	1.000000	O
H2486	1.0	0.348179	0.244185	0.043608	Biso	1.000000	H
H2487	1.0	0.377055	0.272651	0.053805	Biso	1.000000	H
O2400	1.0	0.246212	0.841878	0.219209	Biso	1.000000	O
H2488	1.0	0.257593	0.862506	0.229879	Biso	1.000000	H
H2489	1.0	0.230945	0.829834	0.234470	Biso	1.000000	H
O2401	1.0	0.582564	0.869325	0.697762	Biso	1.000000	O
H2490	1.0	0.593630	0.847205	0.706231	Biso	1.000000	H
H2491	1.0	0.565038	0.862090	0.682408	Biso	1.000000	H
O2402	1.0	0.334752	0.954854	0.396429	Biso	1.000000	O
H2492	1.0	0.318958	0.943532	0.411621	Biso	1.000000	H
H2493	1.0	0.322625	0.972160	0.382505	Biso	1.000000	H
O2403	1.0	0.911183	0.274511	0.592871	Biso	1.000000	O
H2494	1.0	0.903152	0.291185	0.575964	Biso	1.000000	H
H2495	1.0	0.921251	0.288963	0.610332	Biso	1.000000	H
O2404	1.0	0.190520	0.022972	0.794138	Biso	1.000000	O
H2496	1.0	0.172024	0.021625	0.809702	Biso	1.000000	H
H2497	1.0	0.182988	0.036267	0.774897	Biso	1.000000	H
O2405	1.0	0.348250	0.154061	0.201320	Biso	1.000000	O
H2498	1.0	0.354537	0.154550	0.178507	Biso	1.000000	H
H2499	1.0	0.354596	0.130236	0.210667	Biso	1.000000	H
O2406	1.0	0.063119	0.500479	0.066301	Biso	1.000000	O
H2500	1.0	0.083504	0.487991	0.073890	Biso	1.000000	H
H2501	1.0	0.056376	0.519266	0.081988	Biso	1.000000	H
O2407	1.0	0.649584	0.733379	0.207620	Biso	1.000000	O
H2502	1.0	0.644619	0.716811	0.189592	Biso	1.000000	H
H2503	1.0	0.660098	0.719343	0.225097	Biso	1.000000	H
O2408	1.0	0.616856	0.078349	0.236851	Biso	1.000000	O
H2504	1.0	0.619509	0.058255	0.252427	Biso	1.000000	H
H2505	1.0	0.595058	0.075978	0.226005	Biso	1.000000	H
O2409	1.0	0.414392	0.400543	0.015676	Biso	1.000000	O
H2506	1.0	0.421922	0.378096	0.026669	Biso	1.000000	H
H2507	1.0	0.433319	0.411471	0.003933	Biso	1.000000	H
O2410	1.0	0.660386	0.506020	0.648922	Biso	1.000000	O
H2508	1.0	0.680785	0.515642	0.659187	Biso	1.000000	H
H2509	1.0	0.653421	0.522699	0.631593	Biso	1.000000	H
O2411	1.0	0.673179	0.723816	0.301444	Biso	1.000000	O
H2510	1.0	0.692651	0.720092	0.315555	Biso	1.000000	H
H2511	1.0	0.675214	0.707853	0.282505	Biso	1.000000	H
O2412	1.0	0.893755	0.101804	0.768880	Biso	1.000000	O
H2512	1.0	0.902264	0.101806	0.791028	Biso	1.000000	H
H2513	1.0	0.872892	0.116170	0.767837	Biso	1.000000	H

O2413	1.0	0.312405	0.688876	0.266979	Biso	1.000000	O
H2514	1.0	0.302673	0.675408	0.285195	Biso	1.000000	H
H2515	1.0	0.332024	0.702939	0.274310	Biso	1.000000	H
O2414	1.0	0.324221	0.846594	0.399676	Biso	1.000000	O
H2516	1.0	0.341946	0.838858	0.384723	Biso	1.000000	H
H2517	1.0	0.334409	0.858843	0.418313	Biso	1.000000	H
O2415	1.0	0.285115	0.264137	0.616994	Biso	1.000000	O
H2518	1.0	0.291249	0.252528	0.637478	Biso	1.000000	H
H2519	1.0	0.304485	0.277749	0.608503	Biso	1.000000	H
O2416	1.0	0.206664	0.169613	0.618317	Biso	1.000000	O
H2520	1.0	0.207063	0.146291	0.606396	Biso	1.000000	H
H2521	1.0	0.214897	0.189528	0.604443	Biso	1.000000	H
O2417	1.0	0.191337	0.471645	0.964494	Biso	1.000000	O
H2522	1.0	0.192516	0.457000	0.944694	Biso	1.000000	H
H2523	1.0	0.213025	0.470515	0.975687	Biso	1.000000	H
O2418	1.0	0.348063	0.612791	0.493883	Biso	1.000000	O
H2524	1.0	0.329607	0.611891	0.509516	Biso	1.000000	H
H2525	1.0	0.354189	0.587641	0.487531	Biso	1.000000	H
O2419	1.0	0.366644	0.819663	0.358776	Biso	1.000000	O
H2526	1.0	0.387677	0.828896	0.349458	Biso	1.000000	H
H2527	1.0	0.356641	0.801408	0.344243	Biso	1.000000	H
O2420	1.0	0.508611	0.630416	0.753273	Biso	1.000000	O
H2528	1.0	0.529930	0.642417	0.747707	Biso	1.000000	H
H2529	1.0	0.490202	0.648279	0.751423	Biso	1.000000	H
O2421	1.0	0.517759	0.281349	0.178385	Biso	1.000000	O
H2530	1.0	0.534463	0.272035	0.163048	Biso	1.000000	H
H2531	1.0	0.513277	0.307437	0.173942	Biso	1.000000	H
O2422	1.0	0.681064	0.948449	0.882507	Biso	1.000000	O
H2532	1.0	0.684800	0.951867	0.859375	Biso	1.000000	H
H2533	1.0	0.668010	0.969246	0.890943	Biso	1.000000	H
O2423	1.0	0.990508	0.892664	0.338644	Biso	1.000000	O
H2534	1.0	0.000650	0.902768	0.358262	Biso	1.000000	H
H2535	1.0	0.002597	0.902049	0.319778	Biso	1.000000	H
O2424	1.0	0.859431	0.723065	0.824832	Biso	1.000000	O
H2536	1.0	0.856585	0.697388	0.831673	Biso	1.000000	H
H2537	1.0	0.870877	0.723722	0.803940	Biso	1.000000	H
O2425	1.0	0.814384	0.970435	0.455935	Biso	1.000000	O
H2538	1.0	0.806807	0.959435	0.435637	Biso	1.000000	H
H2539	1.0	0.821423	0.950868	0.470756	Biso	1.000000	H
O2426	1.0	0.069819	0.033545	0.983216	Biso	1.000000	O
H2540	1.0	0.045811	0.037211	0.987568	Biso	1.000000	H
H2541	1.0	0.082665	0.054211	0.992173	Biso	1.000000	H
O2427	1.0	0.696979	0.675468	0.897931	Biso	1.000000	O
H2542	1.0	0.676378	0.682782	0.886639	Biso	1.000000	H
H2543	1.0	0.699204	0.648555	0.897364	Biso	1.000000	H
O2428	1.0	0.174646	0.196219	0.199023	Biso	1.000000	O
H2544	1.0	0.181476	0.170249	0.198792	Biso	1.000000	H
H2545	1.0	0.165734	0.202531	0.220324	Biso	1.000000	H
O2429	1.0	0.344746	0.948873	0.973567	Biso	1.000000	O
H2546	1.0	0.356478	0.944665	0.953139	Biso	1.000000	H
H2547	1.0	0.337497	0.925183	0.982562	Biso	1.000000	H
O2430	1.0	0.463216	0.942002	0.473465	Biso	1.000000	O
H2548	1.0	0.473392	0.919815	0.482786	Biso	1.000000	H
H2549	1.0	0.480714	0.960564	0.469742	Biso	1.000000	H
O2431	1.0	0.661540	0.318041	0.527973	Biso	1.000000	O
H2550	1.0	0.677318	0.305135	0.542185	Biso	1.000000	H
H2551	1.0	0.639395	0.306229	0.529243	Biso	1.000000	H
O2432	1.0	0.181800	0.361324	0.591597	Biso	1.000000	O
H2552	1.0	0.167848	0.377509	0.604973	Biso	1.000000	H
H2553	1.0	0.202446	0.355002	0.603265	Biso	1.000000	H
O2433	1.0	0.468681	0.948162	0.289476	Biso	1.000000	O
H2554	1.0	0.462592	0.934405	0.308933	Biso	1.000000	H
H2555	1.0	0.448679	0.960527	0.280870	Biso	1.000000	H
O2434	1.0	0.592725	0.086314	0.768620	Biso	1.000000	O
H2556	1.0	0.605809	0.065171	0.760916	Biso	1.000000	H
H2557	1.0	0.605219	0.109119	0.764437	Biso	1.000000	H
O2435	1.0	0.064854	0.731098	0.488442	Biso	1.000000	O
H2558	1.0	0.080509	0.726246	0.506177	Biso	1.000000	H
H2559	1.0	0.067083	0.711716	0.472134	Biso	1.000000	H
O2436	1.0	0.038288	0.025057	0.858898	Biso	1.000000	O
H2560	1.0	0.036075	0.051955	0.857966	Biso	1.000000	H
H2561	1.0	0.030286	0.014292	0.838655	Biso	1.000000	H
O2437	1.0	0.895274	0.068281	0.877292	Biso	1.000000	O
H2562	1.0	0.897065	0.086778	0.860170	Biso	1.000000	H
H2563	1.0	0.891856	0.080670	0.898006	Biso	1.000000	H
O2438	1.0	0.956374	0.827244	0.870569	Biso	1.000000	O
H2564	1.0	0.959474	0.810154	0.888609	Biso	1.000000	H
H2565	1.0	0.976337	0.826663	0.856723	Biso	1.000000	H
O2439	1.0	0.222128	0.478283	0.167155	Biso	1.000000	O
H2566	1.0	0.241626	0.487110	0.154932	Biso	1.000000	H
H2567	1.0	0.218249	0.452176	0.162306	Biso	1.000000	H
O2440	1.0	0.510890	0.049819	0.137997	Biso	1.000000	O
H2568	1.0	0.517593	0.069064	0.122720	Biso	1.000000	H
H2569	1.0	0.531025	0.037807	0.146700	Biso	1.000000	H
O2441	1.0	0.366674	0.772600	0.641147	Biso	1.000000	O

H2570	1.0	0.374983	0.747383	0.638162	Biso	1.000000	H
H2571	1.0	0.379634	0.784736	0.658200	Biso	1.000000	H
O2442	1.0	0.661474	0.830944	0.484515	Biso	1.000000	O
H2572	1.0	0.673433	0.816290	0.468323	Biso	1.000000	H
H2573	1.0	0.650844	0.852224	0.474112	Biso	1.000000	H
O2443	1.0	0.329191	0.936537	0.556228	Biso	1.000000	O
H2574	1.0	0.338434	0.928640	0.535465	Biso	1.000000	H
H2575	1.0	0.339586	0.922172	0.573566	Biso	1.000000	H
O2444	1.0	0.426053	0.638665	0.970353	Biso	1.000000	O
H2576	1.0	0.446792	0.634449	0.982578	Biso	1.000000	H
H2577	1.0	0.431657	0.643747	0.947806	Biso	1.000000	H
O2445	1.0	0.488984	0.547760	0.988510	Biso	1.000000	O
H2578	1.0	0.471547	0.529043	0.985126	Biso	1.000000	H
H2579	1.0	0.503168	0.549468	0.969264	Biso	1.000000	H
O2446	1.0	0.743733	0.553695	0.197302	Biso	1.000000	O
H2580	1.0	0.736033	0.566056	0.177652	Biso	1.000000	H
H2581	1.0	0.747559	0.527494	0.192813	Biso	1.000000	H
O2447	1.0	0.888630	0.124261	0.220514	Biso	1.000000	O
H2582	1.0	0.886590	0.148142	0.231391	Biso	1.000000	H
H2583	1.0	0.893148	0.104985	0.236480	Biso	1.000000	H
O2448	1.0	0.781626	0.842780	0.276764	Biso	1.000000	O
H2584	1.0	0.765245	0.861184	0.269485	Biso	1.000000	H
H2585	1.0	0.779465	0.839337	0.300075	Biso	1.000000	H
O2449	1.0	0.039742	0.982458	0.040318	Biso	1.000000	O
H2586	1.0	0.053393	0.971487	0.023158	Biso	1.000000	H
H2587	1.0	0.016151	0.983416	0.033488	Biso	1.000000	H
O2450	1.0	0.399986	0.905984	0.009811	Biso	1.000000	O
H2588	1.0	0.396573	0.882791	0.998142	Biso	1.000000	H
H2589	1.0	0.406231	0.925445	0.994564	Biso	1.000000	H
O2451	1.0	0.435413	0.354292	0.802647	Biso	1.000000	O
H2590	1.0	0.416622	0.370465	0.808501	Biso	1.000000	H
H2591	1.0	0.456495	0.368287	0.803421	Biso	1.000000	H
O2452	1.0	0.477799	0.619118	0.006490	Biso	1.000000	O
H2592	1.0	0.482867	0.593422	0.001015	Biso	1.000000	H
H2593	1.0	0.469591	0.620437	0.028713	Biso	1.000000	H
O2453	1.0	0.201918	0.654299	0.542782	Biso	1.000000	O
H2594	1.0	0.204298	0.662924	0.520536	Biso	1.000000	H
H2595	1.0	0.183604	0.636249	0.543998	Biso	1.000000	H
O2454	1.0	0.761424	0.981377	0.561438	Biso	1.000000	O
H2596	1.0	0.769820	0.983507	0.583548	Biso	1.000000	H
H2597	1.0	0.767092	0.956974	0.552865	Biso	1.000000	H
O2455	1.0	0.021618	0.417049	0.099836	Biso	1.000000	O
H2598	1.0	0.015480	0.430293	0.080122	Biso	1.000000	H
H2599	1.0	0.010911	0.428934	0.118388	Biso	1.000000	H
O2456	1.0	0.550758	0.178509	0.991990	Biso	1.000000	O
H2600	1.0	0.541312	0.154281	0.986723	Biso	1.000000	H
H2601	1.0	0.573685	0.180265	0.983431	Biso	1.000000	H
O2457	1.0	0.436858	0.532851	0.921173	Biso	1.000000	O
H2602	1.0	0.444501	0.514596	0.936963	Biso	1.000000	H
H2603	1.0	0.422065	0.550763	0.931755	Biso	1.000000	H
O2458	1.0	0.386242	0.982343	0.740748	Biso	1.000000	O
H2604	1.0	0.409894	0.989820	0.742180	Biso	1.000000	H
H2605	1.0	0.381316	0.974008	0.718801	Biso	1.000000	H
O2459	1.0	0.981633	0.826541	0.742244	Biso	1.000000	O
H2606	1.0	0.988967	0.836429	0.721434	Biso	1.000000	H
H2607	1.0	0.965837	0.806184	0.738695	Biso	1.000000	H
O2460	1.0	0.510337	0.452895	0.851135	Biso	1.000000	O
H2608	1.0	0.494370	0.471928	0.858011	Biso	1.000000	H
H2609	1.0	0.533383	0.461714	0.854517	Biso	1.000000	H
O2461	1.0	0.685644	0.025214	0.348736	Biso	1.000000	O
H2610	1.0	0.690076	0.034106	0.370610	Biso	1.000000	H
H2611	1.0	0.702610	0.035218	0.334005	Biso	1.000000	H
O2462	1.0	0.294502	0.167396	0.362009	Biso	1.000000	O
H2612	1.0	0.292876	0.165768	0.338507	Biso	1.000000	H
H2613	1.0	0.315776	0.156426	0.369140	Biso	1.000000	H
O2463	1.0	0.356579	0.758970	0.224232	Biso	1.000000	O
H2614	1.0	0.340144	0.777636	0.230865	Biso	1.000000	H
H2615	1.0	0.349580	0.748102	0.203691	Biso	1.000000	H
O2464	1.0	0.494089	0.069444	0.734382	Biso	1.000000	O
H2616	1.0	0.483161	0.069019	0.755531	Biso	1.000000	H
H2617	1.0	0.514283	0.053941	0.734920	Biso	1.000000	H
O2465	1.0	0.134617	0.002760	0.199500	Biso	1.000000	O
H2618	1.0	0.119531	0.000619	0.218074	Biso	1.000000	H
H2619	1.0	0.143032	0.978265	0.193613	Biso	1.000000	H
O2466	1.0	0.792495	0.104550	0.392420	Biso	1.000000	O
H2620	1.0	0.801833	0.081414	0.400726	Biso	1.000000	H
H2621	1.0	0.801966	0.125342	0.404471	Biso	1.000000	H

O2467	1.0	0.181570	0.946484	0.598510	Biso	1.000000	O
H2622	1.0	0.169338	0.924591	0.605891	Biso	1.000000	H
H2623	1.0	0.194572	0.940338	0.579189	Biso	1.000000	H
O2468	1.0	0.834317	0.584668	0.962820	Biso	1.000000	O
H2624	1.0	0.820495	0.579024	0.981731	Biso	1.000000	H
H2625	1.0	0.857584	0.589154	0.969594	Biso	1.000000	H
O2469	1.0	0.197535	0.105684	0.587451	Biso	1.000000	O
H2626	1.0	0.179052	0.115056	0.574142	Biso	1.000000	H
H2627	1.0	0.191568	0.081308	0.595898	Biso	1.000000	H
O2470	1.0	0.943795	0.170079	0.953692	Biso	1.000000	O
H2628	1.0	0.960773	0.178559	0.969125	Biso	1.000000	H
H2629	1.0	0.922025	0.167703	0.964586	Biso	1.000000	H
O2471	1.0	0.683173	0.362484	0.395580	Biso	1.000000	O
H2630	1.0	0.676501	0.341602	0.409134	Biso	1.000000	H
H2631	1.0	0.692083	0.353283	0.375096	Biso	1.000000	H
O2472	1.0	0.364148	0.730376	0.284704	Biso	1.000000	O
H2632	1.0	0.367239	0.740804	0.263135	Biso	1.000000	H
H2633	1.0	0.385997	0.722662	0.293331	Biso	1.000000	H
O2473	1.0	0.397365	0.563864	0.284549	Biso	1.000000	O
H2634	1.0	0.404705	0.542924	0.271384	Biso	1.000000	H
H2635	1.0	0.373126	0.567642	0.281696	Biso	1.000000	H
O2474	1.0	0.584026	0.623294	0.880584	Biso	1.000000	O
H2636	1.0	0.577658	0.615353	0.902300	Biso	1.000000	H
H2637	1.0	0.572802	0.607604	0.864651	Biso	1.000000	H
O2475	1.0	0.583735	0.931715	0.749399	Biso	1.000000	O
H2638	1.0	0.577044	0.955022	0.759486	Biso	1.000000	H
H2639	1.0	0.588525	0.913409	0.766143	Biso	1.000000	H
O2476	1.0	0.104768	0.974996	0.605705	Biso	1.000000	O
H2640	1.0	0.109092	0.987824	0.626059	Biso	1.000000	H
H2641	1.0	0.102366	0.948503	0.609780	Biso	1.000000	H
O2477	1.0	0.785293	0.880307	0.208991	Biso	1.000000	O
H2642	1.0	0.805372	0.894859	0.214124	Biso	1.000000	H
H2643	1.0	0.768897	0.882639	0.226501	Biso	1.000000	H
O2478	1.0	0.259879	0.042897	0.976261	Biso	1.000000	O
H2644	1.0	0.281391	0.055449	0.972636	Biso	1.000000	H
H2645	1.0	0.259440	0.019776	0.964048	Biso	1.000000	H
O2479	1.0	0.897612	0.593771	0.985755	Biso	1.000000	O
H2646	1.0	0.909066	0.572440	0.995238	Biso	1.000000	H
H2647	1.0	0.911899	0.604644	0.969027	Biso	1.000000	H
O2480	1.0	0.699934	0.808011	0.132908	Biso	1.000000	O
H2648	1.0	0.689336	0.821401	0.150720	Biso	1.000000	H
H2649	1.0	0.714981	0.824655	0.121146	Biso	1.000000	H
O2481	1.0	0.807579	0.939627	0.899156	Biso	1.000000	O
H2650	1.0	0.783256	0.944084	0.899000	Biso	1.000000	H
H2651	1.0	0.813434	0.922577	0.881723	Biso	1.000000	H
O2482	1.0	0.550307	0.842296	0.785185	Biso	1.000000	O
H2652	1.0	0.540232	0.852165	0.765444	Biso	1.000000	H
H2653	1.0	0.570486	0.856549	0.790591	Biso	1.000000	H
O2483	1.0	0.410631	0.728260	0.123411	Biso	1.000000	O
H2654	1.0	0.400571	0.709821	0.137728	Biso	1.000000	H
H2655	1.0	0.392955	0.744434	0.114960	Biso	1.000000	H
O2484	1.0	0.215312	0.209423	0.908449	Biso	1.000000	O
H2656	1.0	0.234559	0.223264	0.916915	Biso	1.000000	H
H2657	1.0	0.205380	0.222938	0.890357	Biso	1.000000	H
O2485	1.0	0.560739	0.577740	0.832584	Biso	1.000000	O
H2658	1.0	0.577236	0.557671	0.831744	Biso	1.000000	H
H2659	1.0	0.563640	0.593768	0.813784	Biso	1.000000	H
O2486	1.0	0.970988	0.494389	0.854934	Biso	1.000000	O
H2660	1.0	0.988622	0.478650	0.845802	Biso	1.000000	H
H2661	1.0	0.976845	0.500825	0.877155	Biso	1.000000	H
O2487	1.0	0.022306	0.678279	0.884940	Biso	1.000000	O
H2662	1.0	0.043739	0.664970	0.886079	Biso	1.000000	H
H2663	1.0	0.012498	0.675189	0.863457	Biso	1.000000	H
O2488	1.0	0.770856	0.874847	0.432855	Biso	1.000000	O
H2664	1.0	0.747058	0.868406	0.430241	Biso	1.000000	H
H2665	1.0	0.784406	0.852270	0.433327	Biso	1.000000	H
H2666	1.0	0.043875	0.114065	0.932108	Biso	1.000000	H
H2667	1.0	0.941500	0.101769	0.046859	Biso	1.000000	H
H2668	1.0	0.027806	0.158735	0.196665	Biso	1.000000	H
H2669	1.0	0.214816	0.344580	0.770879	Biso	1.000000	H
H2670	1.0	0.975666	0.203859	0.085055	Biso	1.000000	H
H2671	1.0	0.287026	0.073106	0.717005	Biso	1.000000	H
H2672	1.0	0.131183	0.885853	0.607562	Biso	1.000000	H
O2489	1.0	0.800333	0.277679	0.298119	Biso	1.000000	O
H2673	1.0	0.790174	0.279968	0.276710	Biso	1.000000	H
H2674	1.0	0.820310	0.261895	0.296824	Biso	1.000000	H
O2490	1.0	0.269005	0.244459	0.932329	Biso	1.000000	O
H2675	1.0	0.280444	0.233882	0.951084	Biso	1.000000	H
H2676	1.0	0.282579	0.240128	0.912994	Biso	1.000000	H

O2491	1.0	0.020794	0.021930	0.700584	Biso	1.000000	O
H2677	1.0	0.037894	0.037523	0.690395	Biso	1.000000	H
H2678	1.0	0.015112	0.001449	0.686179	Biso	1.000000	H
O2492	1.0	0.470578	0.766573	0.839108	Biso	1.000000	O
H2679	1.0	0.473742	0.785558	0.855628	Biso	1.000000	H
H2680	1.0	0.451032	0.751350	0.844634	Biso	1.000000	H
O2493	1.0	0.322132	0.341799	0.939075	Biso	1.000000	O
H2681	1.0	0.326029	0.324603	0.956894	Biso	1.000000	H
H2682	1.0	0.300754	0.354716	0.942441	Biso	1.000000	H
O2494	1.0	0.391781	0.844918	0.975911	Biso	1.000000	O
H2683	1.0	0.415584	0.838145	0.974116	Biso	1.000000	H
H2684	1.0	0.378658	0.823247	0.982326	Biso	1.000000	H
O2495	1.0	0.814914	0.728700	0.372679	Biso	1.000000	O
H2685	1.0	0.802489	0.722395	0.392305	Biso	1.000000	H
H2686	1.0	0.830033	0.708359	0.367004	Biso	1.000000	H
O2496	1.0	0.331600	0.891613	0.320734	Biso	1.000000	O
H2687	1.0	0.312910	0.887021	0.335600	Biso	1.000000	H
H2688	1.0	0.334375	0.870325	0.306434	Biso	1.000000	H
O2497	1.0	0.439993	0.564512	0.070678	Biso	1.000000	O
H2689	1.0	0.459183	0.550547	0.062251	Biso	1.000000	H
H2690	1.0	0.444659	0.590984	0.068992	Biso	1.000000	H
O2498	1.0	0.400388	0.725703	0.012525	Biso	1.000000	O
H2691	1.0	0.391377	0.700961	0.008542	Biso	1.000000	H
H2692	1.0	0.414111	0.725400	0.032130	Biso	1.000000	H
O2499	1.0	0.056966	0.081339	0.532560	Biso	1.000000	O
H2693	1.0	0.075742	0.074051	0.546475	Biso	1.000000	H
H2694	1.0	0.043164	0.059665	0.527619	Biso	1.000000	H
O2500	1.0	0.277023	0.449457	0.876334	Biso	1.000000	O
H2695	1.0	0.265609	0.427674	0.885046	Biso	1.000000	H
H2696	1.0	0.292450	0.441902	0.859144	Biso	1.000000	H
O2501	1.0	0.981736	0.670286	0.937528	Biso	1.000000	O
H2697	1.0	0.996116	0.665826	0.956298	Biso	1.000000	H
H2698	1.0	0.995820	0.673846	0.918408	Biso	1.000000	H
O2502	1.0	0.057622	0.173237	0.886417	Biso	1.000000	O
H2699	1.0	0.049255	0.159900	0.905315	Biso	1.000000	H
H2700	1.0	0.082285	0.173158	0.886549	Biso	1.000000	H
O2503	1.0	0.815930	0.560296	0.839134	Biso	1.000000	O
H2701	1.0	0.797926	0.542376	0.835207	Biso	1.000000	H
H2702	1.0	0.831458	0.560417	0.820802	Biso	1.000000	H
O2504	1.0	0.411127	0.735701	0.373184	Biso	1.000000	O
H2703	1.0	0.419739	0.732941	0.395165	Biso	1.000000	H
H2704	1.0	0.386875	0.740547	0.373902	Biso	1.000000	H
O2505	1.0	0.837099	0.894959	0.335824	Biso	1.000000	O
H2705	1.0	0.836457	0.876131	0.352743	Biso	1.000000	H
H2706	1.0	0.854775	0.912952	0.340730	Biso	1.000000	H
O2506	1.0	0.209028	0.456100	0.297784	Biso	1.000000	O
H2707	1.0	0.202194	0.469290	0.278255	Biso	1.000000	H
H2708	1.0	0.189761	0.454057	0.312407	Biso	1.000000	H
O2507	1.0	0.425432	0.544968	0.340359	Biso	1.000000	O
H2709	1.0	0.446620	0.558744	0.339278	Biso	1.000000	H
H2710	1.0	0.412145	0.549833	0.320939	Biso	1.000000	H
O2508	1.0	0.456480	0.250775	0.352910	Biso	1.000000	O
H2711	1.0	0.480589	0.256476	0.353054	Biso	1.000000	H
H2712	1.0	0.443547	0.273773	0.352200	Biso	1.000000	H
O2509	1.0	0.220032	0.964503	0.182717	Biso	1.000000	O
H2713	1.0	0.235855	0.946492	0.173751	Biso	1.000000	H
H2714	1.0	0.197595	0.953405	0.184165	Biso	1.000000	H
O2510	1.0	0.349232	0.994902	0.260858	Biso	1.000000	O
H2715	1.0	0.338693	0.980115	0.277842	Biso	1.000000	H
H2716	1.0	0.340637	0.020211	0.261842	Biso	1.000000	H
O2511	1.0	0.838823	0.506317	0.015624	Biso	1.000000	O
H2717	1.0	0.825319	0.528932	0.015332	Biso	1.000000	H
H2718	1.0	0.827722	0.487367	0.002576	Biso	1.000000	H
O2512	1.0	0.948441	0.270611	0.005026	Biso	1.000000	O
H2719	1.0	0.940434	0.291501	0.017890	Biso	1.000000	H
H2720	1.0	0.972660	0.267337	0.008449	Biso	1.000000	H
O2513	1.0	0.275943	0.058215	0.180312	Biso	1.000000	O
H2721	1.0	0.258861	0.051962	0.164190	Biso	1.000000	H
H2722	1.0	0.298287	0.057560	0.170345	Biso	1.000000	H
O2514	1.0	0.208543	0.849456	0.391715	Biso	1.000000	O
H2723	1.0	0.222408	0.831436	0.403261	Biso	1.000000	H
H2724	1.0	0.217864	0.874223	0.394855	Biso	1.000000	H
O2515	1.0	0.324527	0.257932	0.237134	Biso	1.000000	O
H2725	1.0	0.322038	0.232677	0.245197	Biso	1.000000	H

H2726	1.0	0.319594	0.275427	0.254489	Biso	1.000000	H
O2516	1.0	0.067038	0.335753	0.040188	Biso	1.000000	O
H2727	1.0	0.091545	0.337063	0.042581	Biso	1.000000	H
H2728	1.0	0.061418	0.319565	0.022071	Biso	1.000000	H
O2517	1.0	0.898360	0.484980	0.991341	Biso	1.000000	O
H2729	1.0	0.876745	0.491327	0.001262	Biso	1.000000	H
H2730	1.0	0.914008	0.505690	0.993745	Biso	1.000000	H
O2518	1.0	0.128756	0.620362	0.607571	Biso	1.000000	O
H2731	1.0	0.112878	0.639190	0.600094	Biso	1.000000	H
H2732	1.0	0.139521	0.608639	0.588973	Biso	1.000000	H
O2519	1.0	0.464784	0.980838	0.403945	Biso	1.000000	O
H2733	1.0	0.440328	0.981511	0.406942	Biso	1.000000	H
H2734	1.0	0.472228	0.955164	0.402012	Biso	1.000000	H
O2520	1.0	0.374302	0.388950	0.932304	Biso	1.000000	O
H2735	1.0	0.368504	0.414025	0.939152	Biso	1.000000	H
H2736	1.0	0.354498	0.373009	0.934339	Biso	1.000000	H
O2521	1.0	0.291510	0.191818	0.187847	Biso	1.000000	O
H2737	1.0	0.275074	0.180413	0.202351	Biso	1.000000	H
H2738	1.0	0.313850	0.181386	0.191974	Biso	1.000000	H
O2522	1.0	0.295212	0.085620	0.527938	Biso	1.000000	O
H2739	1.0	0.276559	0.078711	0.513726	Biso	1.000000	H
H2740	1.0	0.286619	0.089867	0.549743	Biso	1.000000	H
O2523	1.0	0.302897	0.812003	0.541171	Biso	1.000000	O
H2741	1.0	0.278988	0.807713	0.545588	Biso	1.000000	H
H2742	1.0	0.314294	0.819373	0.561084	Biso	1.000000	H
O2524	1.0	0.605086	0.122371	0.441445	Biso	1.000000	O
H2743	1.0	0.607146	0.145281	0.429082	Biso	1.000000	H
H2744	1.0	0.598827	0.128176	0.463698	Biso	1.000000	H
O2525	1.0	0.158533	0.730401	0.682948	Biso	1.000000	O
H2745	1.0	0.138484	0.714856	0.685105	Biso	1.000000	H
H2746	1.0	0.160706	0.738623	0.660565	Biso	1.000000	H
O2526	1.0	0.223914	0.382961	0.287765	Biso	1.000000	O
H2747	1.0	0.238672	0.380288	0.306526	Biso	1.000000	H
H2748	1.0	0.216621	0.408692	0.285917	Biso	1.000000	H
O2527	1.0	0.375484	0.090721	0.222169	Biso	1.000000	O
H2749	1.0	0.375851	0.065153	0.214526	Biso	1.000000	H
H2750	1.0	0.377708	0.091066	0.245669	Biso	1.000000	H
O2528	1.0	0.612197	0.429785	0.256573	Biso	1.000000	O
H2751	1.0	0.610465	0.403459	0.251483	Biso	1.000000	H
H2752	1.0	0.594249	0.436637	0.271611	Biso	1.000000	H
O2529	1.0	0.765772	0.999389	0.205642	Biso	1.000000	O
H2753	1.0	0.750178	0.982015	0.215847	Biso	1.000000	H
H2754	1.0	0.763003	0.998333	0.182213	Biso	1.000000	H
O2530	1.0	0.356678	0.213471	0.564158	Biso	1.000000	O
H2755	1.0	0.374795	0.225464	0.576271	Biso	1.000000	H
H2756	1.0	0.364683	0.189794	0.555742	Biso	1.000000	H
O2531	1.0	0.318955	0.266936	0.533832	Biso	1.000000	O
H2757	1.0	0.331321	0.248830	0.546751	Biso	1.000000	H
H2758	1.0	0.331973	0.272954	0.514490	Biso	1.000000	H
O2532	1.0	0.337956	0.053426	0.153732	Biso	1.000000	O
H2759	1.0	0.354281	0.045974	0.170179	Biso	1.000000	H
H2760	1.0	0.338659	0.035922	0.135765	Biso	1.000000	H
O2533	1.0	0.214132	0.193248	0.514122	Biso	1.000000	O
H2761	1.0	0.235391	0.202602	0.505379	Biso	1.000000	H
H2762	1.0	0.197564	0.213255	0.514785	Biso	1.000000	H
O2534	1.0	0.898481	0.633625	0.041036	Biso	1.000000	O
H2763	1.0	0.896581	0.620375	0.020549	Biso	1.000000	H
H2764	1.0	0.888704	0.618648	0.058308	Biso	1.000000	H
O2535	1.0	0.421976	0.377066	0.456339	Biso	1.000000	O
H2765	1.0	0.413689	0.402019	0.451936	Biso	1.000000	H
H2766	1.0	0.443574	0.378544	0.467658	Biso	1.000000	H
O2536	1.0	0.181305	0.762478	0.217208	Biso	1.000000	O
H2767	1.0	0.156738	0.763857	0.215508	Biso	1.000000	H
H2768	1.0	0.189433	0.782377	0.231157	Biso	1.000000	H
O2537	1.0	0.844002	0.947733	0.073563	Biso	1.000000	O
H2769	1.0	0.847800	0.970426	0.061272	Biso	1.000000	H
H2770	1.0	0.839950	0.927237	0.058676	Biso	1.000000	H
O2538	1.0	0.373646	0.935324	0.917351	Biso	1.000000	O
H2771	1.0	0.379977	0.947846	0.897336	Biso	1.000000	H
H2772	1.0	0.375570	0.908587	0.914431	Biso	1.000000	H
H2773	1.0	0.307028	0.774909	0.475030	Biso	1.000000	H
H2774	1.0	0.584394	0.932524	0.504327	Biso	1.000000	H

O2539	1.0	0.036095	0.733076	0.960982	Biso	1.000000	O
H2775	1.0	0.027515	0.738615	0.939394	Biso	1.000000	H
H2776	1.0	0.060593	0.730125	0.960088	Biso	1.000000	H
O2540	1.0	0.055238	0.890507	0.715493	Biso	1.000000	O
H2777	1.0	0.036194	0.877866	0.705344	Biso	1.000000	H
H2778	1.0	0.059583	0.913776	0.704230	Biso	1.000000	H
O2541	1.0	0.333026	0.023310	0.755463	Biso	1.000000	O
H2779	1.0	0.353987	0.009526	0.752332	Biso	1.000000	H
H2780	1.0	0.330111	0.040922	0.737784	Biso	1.000000	H
O2542	1.0	0.678107	0.801186	0.211756	Biso	1.000000	O
H2781	1.0	0.666384	0.778336	0.206008	Biso	1.000000	H
H2782	1.0	0.691918	0.796996	0.230960	Biso	1.000000	H
O2543	1.0	0.518082	0.703458	0.870588	Biso	1.000000	O
H2783	1.0	0.542210	0.701018	0.874991	Biso	1.000000	H
H2784	1.0	0.507578	0.679006	0.870801	Biso	1.000000	H
O2544	1.0	0.348053	0.534343	0.419974	Biso	1.000000	O
H2785	1.0	0.325819	0.542160	0.412374	Biso	1.000000	H
H2786	1.0	0.351286	0.508117	0.415172	Biso	1.000000	H
O2545	1.0	0.915342	0.770219	0.413971	Biso	1.000000	O
H2787	1.0	0.912868	0.757145	0.393456	Biso	1.000000	H
H2788	1.0	0.909877	0.796345	0.410932	Biso	1.000000	H
O2546	1.0	0.330855	0.304416	0.997992	Biso	1.000000	O
H2789	1.0	0.355462	0.306143	0.997464	Biso	1.000000	H
H2790	1.0	0.324218	0.280738	0.007438	Biso	1.000000	H
O2547	1.0	0.064246	0.140095	0.464274	Biso	1.000000	O
H2791	1.0	0.051336	0.139931	0.484378	Biso	1.000000	H
H2792	1.0	0.065480	0.114990	0.455610	Biso	1.000000	H
O2548	1.0	0.839262	0.142349	0.770650	Biso	1.000000	O
H2793	1.0	0.830087	0.153910	0.751211	Biso	1.000000	H
H2794	1.0	0.839772	0.160438	0.788177	Biso	1.000000	H
O2549	1.0	0.668614	0.907954	0.529011	Biso	1.000000	O
H2795	1.0	0.650205	0.916393	0.515144	Biso	1.000000	H
H2796	1.0	0.680021	0.929340	0.538455	Biso	1.000000	H
O2550	1.0	0.622664	0.114584	0.310795	Biso	1.000000	O
H2797	1.0	0.626543	0.089848	0.319549	Biso	1.000000	H
H2798	1.0	0.600001	0.115866	0.301552	Biso	1.000000	H
O2551	1.0	0.419357	0.455862	0.849814	Biso	1.000000	O
H2799	1.0	0.419249	0.431821	0.860595	Biso	1.000000	H
H2800	1.0	0.396410	0.465739	0.849172	Biso	1.000000	H
O2552	1.0	0.374171	0.516763	0.150826	Biso	1.000000	O
H2801	1.0	0.369436	0.529240	0.171262	Biso	1.000000	H
H2802	1.0	0.395525	0.525821	0.142060	Biso	1.000000	H
O2553	1.0	0.703633	0.863092	0.427532	Biso	1.000000	O
H2803	1.0	0.686404	0.877228	0.439054	Biso	1.000000	H
H2804	1.0	0.700420	0.836720	0.431690	Biso	1.000000	H
O2554	1.0	0.392918	0.150621	0.657816	Biso	1.000000	O
H2805	1.0	0.381578	0.140313	0.638891	Biso	1.000000	H
H2806	1.0	0.390979	0.133338	0.675862	Biso	1.000000	H
O2555	1.0	0.893484	0.893183	0.197339	Biso	1.000000	O
H2807	1.0	0.897820	0.871133	0.210341	Biso	1.000000	H
H2808	1.0	0.873060	0.905430	0.205124	Biso	1.000000	H
O2556	1.0	0.516922	0.320730	0.853483	Biso	1.000000	O
H2809	1.0	0.526896	0.336031	0.836534	Biso	1.000000	H
H2810	1.0	0.534670	0.311992	0.867983	Biso	1.000000	H
O2557	1.0	0.998043	0.393288	0.246622	Biso	1.000000	O
H2811	1.0	0.993025	0.381426	0.225969	Biso	1.000000	H
H2812	1.0	0.977242	0.395891	0.259095	Biso	1.000000	H
O2558	1.0	0.459413	0.955115	0.170749	Biso	1.000000	O
H2813	1.0	0.436683	0.948695	0.163505	Biso	1.000000	H
H2814	1.0	0.469253	0.934140	0.182272	Biso	1.000000	H
O2559	1.0	0.170777	0.553914	0.996539	Biso	1.000000	O
H2815	1.0	0.194934	0.548471	0.996345	Biso	1.000000	H
H2816	1.0	0.160083	0.540811	0.014462	Biso	1.000000	H
O2560	1.0	0.413835	0.302924	0.128555	Biso	1.000000	O
H2817	1.0	0.391823	0.301715	0.139146	Biso	1.000000	H
H2818	1.0	0.428702	0.284058	0.137677	Biso	1.000000	H
O2561	1.0	0.236195	0.233067	0.240534	Biso	1.000000	O
H2819	1.0	0.221935	0.251429	0.251197	Biso	1.000000	H
H2820	1.0	0.244550	0.243132	0.220146	Biso	1.000000	H

O2562	1.0	0.802822	0.030578	0.897897	Biso	1.000000	O
H2821	1.0	0.822256	0.014098	0.895893	Biso	1.000000	H
H2822	1.0	0.799867	0.044373	0.877804	Biso	1.000000	H
O2563	1.0	0.306079	0.988063	0.477911	Biso	1.000000	O
H2823	1.0	0.294364	0.008735	0.467639	Biso	1.000000	H
H2824	1.0	0.329383	0.995152	0.482524	Biso	1.000000	H
O2564	1.0	0.512125	0.762954	0.154491	Biso	1.000000	O
H2825	1.0	0.517411	0.738344	0.146142	Biso	1.000000	H
H2826	1.0	0.509486	0.761735	0.177928	Biso	1.000000	H
O2565	1.0	0.425469	0.038729	0.638603	Biso	1.000000	O
H2827	1.0	0.406155	0.046201	0.625458	Biso	1.000000	H
H2828	1.0	0.446072	0.040344	0.625710	Biso	1.000000	H
O2566	1.0	0.918761	0.247444	0.348281	Biso	1.000000	O
H2829	1.0	0.909140	0.227980	0.334741	Biso	1.000000	H
H2830	1.0	0.909922	0.271435	0.341454	Biso	1.000000	H
O2567	1.0	0.178434	0.821741	0.796587	Biso	1.000000	O
H2831	1.0	0.168081	0.803078	0.782689	Biso	1.000000	H
H2832	1.0	0.161853	0.830482	0.812300	Biso	1.000000	H
O2568	1.0	0.135705	0.497378	0.217855	Biso	1.000000	O
H2833	1.0	0.112979	0.500349	0.226646	Biso	1.000000	H
H2834	1.0	0.134252	0.489137	0.195426	Biso	1.000000	H
O2569	1.0	0.262252	0.918042	0.151868	Biso	1.000000	O
H2835	1.0	0.267725	0.893040	0.159149	Biso	1.000000	H
H2836	1.0	0.282612	0.929953	0.143548	Biso	1.000000	H
O2570	1.0	0.874330	0.902519	0.113858	Biso	1.000000	O
H2837	1.0	0.891042	0.915468	0.127026	Biso	1.000000	H
H2838	1.0	0.863898	0.919936	0.098827	Biso	1.000000	H
O2571	1.0	0.367428	0.949917	0.211009	Biso	1.000000	O
H2839	1.0	0.353740	0.927548	0.209017	Biso	1.000000	H
H2840	1.0	0.359445	0.964529	0.229332	Biso	1.000000	H
O2572	1.0	0.656719	0.536505	0.213181	Biso	1.000000	O
H2841	1.0	0.672747	0.553120	0.223729	Biso	1.000000	H
H2842	1.0	0.664246	0.531491	0.191139	Biso	1.000000	H
O2573	1.0	0.208493	0.374241	0.992484	Biso	1.000000	O
H2843	1.0	0.197608	0.388342	0.009712	Biso	1.000000	H
H2844	1.0	0.213299	0.349210	0.000105	Biso	1.000000	H
O2574	1.0	0.167062	0.638092	0.837985	Biso	1.000000	O
H2845	1.0	0.150595	0.619394	0.831496	Biso	1.000000	H
H2846	1.0	0.180784	0.628521	0.855725	Biso	1.000000	H
O2575	1.0	0.920303	0.059694	0.965707	Biso	1.000000	O
H2847	1.0	0.906817	0.078400	0.954590	Biso	1.000000	H
H2848	1.0	0.910637	0.035239	0.961773	Biso	1.000000	H
O2576	1.0	0.497762	0.302242	0.471301	Biso	1.000000	O
H2849	1.0	0.478152	0.307556	0.457764	Biso	1.000000	H
H2850	1.0	0.516451	0.318357	0.465048	Biso	1.000000	H
O2577	1.0	0.499064	0.970362	0.556586	Biso	1.000000	O
H2851	1.0	0.512458	0.980034	0.574510	Biso	1.000000	H
H2852	1.0	0.481748	0.988375	0.550668	Biso	1.000000	H
O2578	1.0	0.483237	0.273170	0.815301	Biso	1.000000	O
H2853	1.0	0.496516	0.289788	0.828900	Biso	1.000000	H
H2854	1.0	0.495337	0.268558	0.795136	Biso	1.000000	H
O2579	1.0	0.860839	0.735125	0.439547	Biso	1.000000	O
H2855	1.0	0.860632	0.708780	0.434276	Biso	1.000000	H
H2856	1.0	0.882005	0.746110	0.432147	Biso	1.000000	H
O2580	1.0	0.039023	0.999384	0.607560	Biso	1.000000	O
H2857	1.0	0.023067	0.979149	0.610979	Biso	1.000000	H
H2858	1.0	0.061602	0.989263	0.604086	Biso	1.000000	H
O2581	1.0	0.509543	0.830217	0.225064	Biso	1.000000	O
H2859	1.0	0.505829	0.804008	0.220524	Biso	1.000000	H
H2860	1.0	0.507348	0.834612	0.248252	Biso	1.000000	H
O2582	1.0	0.470716	0.612458	0.266459	Biso	1.000000	O
H2861	1.0	0.452805	0.629654	0.260313	Biso	1.000000	H
H2862	1.0	0.472691	0.593098	0.250102	Biso	1.000000	H
O2583	1.0	0.614675	0.020690	0.847954	Biso	1.000000	O
H2863	1.0	0.602684	0.041285	0.837860	Biso	1.000000	H
H2864	1.0	0.620651	0.002461	0.831496	Biso	1.000000	H
O2584	1.0	0.963283	0.048571	0.169981	Biso	1.000000	O
H2865	1.0	0.962593	0.064834	0.151145	Biso	1.000000	H
H2866	1.0	0.945715	0.029733	0.168027	Biso	1.000000	H
O2585	1.0	0.777880	0.790210	0.226149	Biso	1.000000	O
H2867	1.0	0.775329	0.770814	0.242398	Biso	1.000000	H
H2868	1.0	0.781515	0.813962	0.236860	Biso	1.000000	H
O2586	1.0	0.415152	0.390480	0.271516	Biso	1.000000	O
H2869	1.0	0.400184	0.369009	0.272132	Biso	1.000000	H
H2870	1.0	0.404347	0.410411	0.259385	Biso	1.000000	H

O2587	1.0	0.540143	0.232875	0.030979	Biso	1.000000	O
H2871	1.0	0.555762	0.229332	0.048979	Biso	1.000000	H
H2872	1.0	0.543485	0.213250	0.015072	Biso	1.000000	H
O2588	1.0	0.188094	0.827231	0.925961	Biso	1.000000	O
H2873	1.0	0.203483	0.817952	0.909396	Biso	1.000000	H
H2874	1.0	0.172892	0.807400	0.932705	Biso	1.000000	H
H2875	1.0	0.524023	0.363106	0.177666	Biso	1.000000	H
H2876	1.0	0.147462	0.186042	0.265686	Biso	1.000000	H
H2877	1.0	0.116985	0.908125	0.115090	Biso	1.000000	H
H2878	1.0	0.245485	0.354108	0.353809	Biso	1.000000	H
H2879	1.0	0.170419	0.089919	0.483661	Biso	1.000000	H
H2880	1.0	0.278890	0.438375	0.068783	Biso	1.000000	H
O2589	1.0	0.829781	0.341126	0.975562	Biso	1.000000	O
H2881	1.0	0.828849	0.341242	0.951982	Biso	1.000000	H
H2882	1.0	0.807451	0.346813	0.984263	Biso	1.000000	H
O2590	1.0	0.607619	0.396529	0.580374	Biso	1.000000	O
H2883	1.0	0.621364	0.376247	0.588758	Biso	1.000000	H
H2884	1.0	0.612835	0.419201	0.592215	Biso	1.000000	H
O2591	1.0	0.846192	0.156040	0.866265	Biso	1.000000	O
H2885	1.0	0.823541	0.146669	0.870768	Biso	1.000000	H
H2886	1.0	0.856689	0.164235	0.886384	Biso	1.000000	H
O2592	1.0	0.344164	0.560390	0.990334	Biso	1.000000	O
H2887	1.0	0.326071	0.545395	0.981074	Biso	1.000000	H
H2888	1.0	0.334488	0.582175	0.000793	Biso	1.000000	H
O2593	1.0	0.848084	0.764395	0.161086	Biso	1.000000	O
H2889	1.0	0.863118	0.773430	0.178046	Biso	1.000000	H
H2890	1.0	0.825334	0.773963	0.164719	Biso	1.000000	H
O2594	1.0	0.854864	0.377963	0.295740	Biso	1.000000	O
H2891	1.0	0.862119	0.403765	0.296819	Biso	1.000000	H
H2892	1.0	0.846789	0.372447	0.273970	Biso	1.000000	H
O2595	1.0	0.987527	0.445832	0.149242	Biso	1.000000	O
H2893	1.0	0.994909	0.447540	0.171708	Biso	1.000000	H
H2894	1.0	0.975106	0.468212	0.143421	Biso	1.000000	H
O2596	1.0	0.637731	0.453941	0.513216	Biso	1.000000	O
H2895	1.0	0.635148	0.467660	0.533396	Biso	1.000000	H
H2896	1.0	0.650126	0.431056	0.517329	Biso	1.000000	H
O2597	1.0	0.267039	0.861963	0.886075	Biso	1.000000	O
H2897	1.0	0.252480	0.840221	0.884498	Biso	1.000000	H
H2898	1.0	0.258407	0.881445	0.871957	Biso	1.000000	H
O2598	1.0	0.492538	0.634684	0.868571	Biso	1.000000	O
H2899	1.0	0.485778	0.632854	0.845932	Biso	1.000000	H
H2900	1.0	0.500198	0.610510	0.876164	Biso	1.000000	H
O2599	1.0	0.751406	0.430702	0.882766	Biso	1.000000	O
H2901	1.0	0.762554	0.453284	0.890136	Biso	1.000000	H
H2902	1.0	0.735202	0.422547	0.899069	Biso	1.000000	H
O2600	1.0	0.425772	0.585076	0.873500	Biso	1.000000	O
H2903	1.0	0.430483	0.564039	0.887613	Biso	1.000000	H
H2904	1.0	0.428418	0.608064	0.885652	Biso	1.000000	H
O2601	1.0	0.051098	0.239748	0.491070	Biso	1.000000	O
H2905	1.0	0.049300	0.253433	0.470794	Biso	1.000000	H
H2906	1.0	0.070564	0.223187	0.490124	Biso	1.000000	H
O2602	1.0	0.168112	0.404011	0.037667	Biso	1.000000	O
H2907	1.0	0.156396	0.424823	0.027616	Biso	1.000000	H
H2908	1.0	0.153019	0.382658	0.038536	Biso	1.000000	H
O2603	1.0	0.528016	0.196364	0.230018	Biso	1.000000	O
H2909	1.0	0.505829	0.191399	0.239371	Biso	1.000000	H
H2910	1.0	0.528953	0.186300	0.208137	Biso	1.000000	H
O2604	1.0	0.236118	0.337228	0.621928	Biso	1.000000	O
H2911	1.0	0.259909	0.344286	0.621071	Biso	1.000000	H
H2912	1.0	0.229248	0.334125	0.644427	Biso	1.000000	H
O2605	1.0	0.822291	0.072360	0.946035	Biso	1.000000	O
H2913	1.0	0.810287	0.095768	0.943330	Biso	1.000000	H
H2914	1.0	0.816882	0.055900	0.928048	Biso	1.000000	H
O2606	1.0	0.939127	0.404127	0.274860	Biso	1.000000	O
H2915	1.0	0.943093	0.415382	0.295976	Biso	1.000000	H
H2916	1.0	0.927941	0.421982	0.260742	Biso	1.000000	H
O2607	1.0	0.670695	0.672735	0.466122	Biso	1.000000	O
H2917	1.0	0.648116	0.682541	0.470225	Biso	1.000000	H
H2918	1.0	0.669050	0.646663	0.460102	Biso	1.000000	H
O2608	1.0	0.488407	0.194761	0.536012	Biso	1.000000	O
H2919	1.0	0.478914	0.192624	0.514313	Biso	1.000000	H
H2920	1.0	0.512071	0.187163	0.535659	Biso	1.000000	H
O2609	1.0	0.093099	0.997188	0.885843	Biso	1.000000	O
H2921	1.0	0.073916	0.010424	0.876546	Biso	1.000000	H

H2922	1.0	0.085199	0.980996	0.903160	Biso	1.000000	H
O2610	1.0	0.921518	0.329850	0.265232	Biso	1.000000	O
H2923	1.0	0.906183	0.325579	0.247132	Biso	1.000000	H
H2924	1.0	0.924893	0.356424	0.268090	Biso	1.000000	H
O2611	1.0	0.057265	0.361184	0.206824	Biso	1.000000	O
H2925	1.0	0.064090	0.384542	0.196912	Biso	1.000000	H
H2926	1.0	0.046591	0.345459	0.190575	Biso	1.000000	H
O2612	1.0	0.938008	0.541457	0.009712	Biso	1.000000	O
H2927	1.0	0.939391	0.525807	0.028906	Biso	1.000000	H
H2928	1.0	0.957731	0.557652	0.008804	Biso	1.000000	H
O2613	1.0	0.886820	0.323506	0.907032	Biso	1.000000	O
H2929	1.0	0.896162	0.309546	0.925153	Biso	1.000000	H
H2930	1.0	0.896909	0.314765	0.886898	Biso	1.000000	H
O2614	1.0	0.322805	0.509882	0.528582	Biso	1.000000	O
H2931	1.0	0.334066	0.486908	0.534776	Biso	1.000000	H
H2932	1.0	0.335776	0.522104	0.511583	Biso	1.000000	H
O2615	1.0	0.424457	0.846648	0.333594	Biso	1.000000	O
H2933	1.0	0.439502	0.828363	0.343330	Biso	1.000000	H
H2934	1.0	0.433972	0.871368	0.336461	Biso	1.000000	H
O2616	1.0	0.972973	0.141477	0.509136	Biso	1.000000	O
H2935	1.0	0.996010	0.143380	0.517400	Biso	1.000000	H
H2936	1.0	0.970880	0.156171	0.489433	Biso	1.000000	H
O2617	1.0	0.397338	0.937910	0.152220	Biso	1.000000	O
H2937	1.0	0.384759	0.941651	0.172252	Biso	1.000000	H
H2938	1.0	0.386402	0.918631	0.139413	Biso	1.000000	H
O2618	1.0	0.437497	0.601363	0.171495	Biso	1.000000	O
H2939	1.0	0.435711	0.580873	0.156199	Biso	1.000000	H
H2940	1.0	0.415122	0.607299	0.179960	Biso	1.000000	H
O2619	1.0	0.440099	0.647425	0.907720	Biso	1.000000	O
H2941	1.0	0.431514	0.672765	0.907760	Biso	1.000000	H
H2942	1.0	0.461097	0.646385	0.895377	Biso	1.000000	H
O2620	1.0	0.338338	0.817952	0.171591	Biso	1.000000	O
H2943	1.0	0.361934	0.823485	0.176477	Biso	1.000000	H
H2944	1.0	0.335724	0.791375	0.168104	Biso	1.000000	H
O2621	1.0	0.652524	0.161503	0.352365	Biso	1.000000	O
H2945	1.0	0.640370	0.145342	0.337447	Biso	1.000000	H
H2946	1.0	0.637681	0.168324	0.370244	Biso	1.000000	H
O2622	1.0	0.585701	0.856235	0.342422	Biso	1.000000	O
H2947	1.0	0.582712	0.881553	0.334670	Biso	1.000000	H
H2948	1.0	0.602109	0.843464	0.328783	Biso	1.000000	H
O2623	1.0	0.530014	0.398810	0.892756	Biso	1.000000	O
H2949	1.0	0.520277	0.417409	0.878389	Biso	1.000000	H
H2950	1.0	0.514158	0.393440	0.910212	Biso	1.000000	H
O2624	1.0	0.584803	0.923266	0.315706	Biso	1.000000	O
H2951	1.0	0.566412	0.922453	0.300000	Biso	1.000000	H
H2952	1.0	0.605168	0.932799	0.305318	Biso	1.000000	H
O2625	1.0	0.821837	0.492267	0.790837	Biso	1.000000	O
H2953	1.0	0.837397	0.513061	0.793194	Biso	1.000000	H
H2954	1.0	0.803394	0.494824	0.806344	Biso	1.000000	H
O2626	1.0	0.969830	0.395521	0.874593	Biso	1.000000	O
H2955	1.0	0.971694	0.400543	0.851475	Biso	1.000000	H
H2956	1.0	0.946249	0.397694	0.881240	Biso	1.000000	H
O2627	1.0	0.382387	0.023849	0.195246	Biso	1.000000	O
H2957	1.0	0.376233	0.998213	0.199853	Biso	1.000000	H
H2958	1.0	0.406510	0.025275	0.190501	Biso	1.000000	H
O2628	1.0	0.872653	0.596374	0.090503	Biso	1.000000	O
H2959	1.0	0.888660	0.598376	0.108371	Biso	1.000000	H
H2960	1.0	0.871770	0.570753	0.083029	Biso	1.000000	H
O2629	1.0	0.000842	0.459044	0.212398	Biso	1.000000	O
H2961	1.0	0.002927	0.436278	0.224960	Biso	1.000000	H
H2962	1.0	0.005290	0.480310	0.226326	Biso	1.000000	H
O2630	1.0	0.692991	0.171557	0.907490	Biso	1.000000	O
H2963	1.0	0.688885	0.193909	0.894817	Biso	1.000000	H
H2964	1.0	0.672471	0.164849	0.919200	Biso	1.000000	H
O2631	1.0	0.861306	0.781020	0.898252	Biso	1.000000	O
H2965	1.0	0.881103	0.784507	0.911992	Biso	1.000000	H
H2966	1.0	0.851014	0.757054	0.902954	Biso	1.000000	H
O2632	1.0	0.567470	0.604039	0.940128	Biso	1.000000	O
H2967	1.0	0.578112	0.584395	0.952736	Biso	1.000000	H
H2968	1.0	0.563630	0.625556	0.953929	Biso	1.000000	H
O2633	1.0	0.497890	0.105891	0.193274	Biso	1.000000	O
H2969	1.0	0.506357	0.129040	0.184177	Biso	1.000000	H
H2970	1.0	0.516831	0.089356	0.197744	Biso	1.000000	H
O2634	1.0	0.061786	0.076397	0.926472	Biso	1.000000	O
H2971	1.0	0.076627	0.077323	0.907643	Biso	1.000000	H
H2972	1.0	0.067162	0.054558	0.939388	Biso	1.000000	H
O2635	1.0	0.636823	0.766149	0.579680	Biso	1.000000	O
H2973	1.0	0.646673	0.769306	0.601137	Biso	1.000000	H
H2974	1.0	0.616456	0.751015	0.581287	Biso	1.000000	H
O2636	1.0	0.011814	0.231736	0.931462	Biso	1.000000	O
H2975	1.0	0.005467	0.219634	0.911256	Biso	1.000000	H
H2976	1.0	0.016352	0.257893	0.927402	Biso	1.000000	H
O2637	1.0	0.514441	0.857149	0.963320	Biso	1.000000	O
H2977	1.0	0.531513	0.869506	0.950145	Biso	1.000000	H
H2978	1.0	0.524067	0.834772	0.972829	Biso	1.000000	H

O2638	1.0	0.479353	0.190490	0.319396	Biso	1.000000	O
H2979	1.0	0.466091	0.209308	0.330619	Biso	1.000000	H
H2980	1.0	0.503370	0.195162	0.322867	Biso	1.000000	H
H2981	1.0	0.498141	0.510660	0.561570	Biso	1.000000	H
H2982	1.0	0.245943	0.195727	0.693104	Biso	1.000000	H
H2983	1.0	0.622642	0.541411	0.102274	Biso	1.000000	H
H2984	1.0	0.558663	0.746534	0.569446	Biso	1.000000	H
H2985	1.0	0.081617	0.175773	0.570043	Biso	1.000000	H
H2986	1.0	0.177051	0.264618	0.543408	Biso	1.000000	H
H2987	1.0	0.475819	0.600520	0.514721	Biso	1.000000	H
H2988	1.0	0.573007	0.589898	0.583821	Biso	1.000000	H
H2989	1.0	0.157841	0.951990	0.676256	Biso	1.000000	H
H2990	1.0	0.807732	0.062512	0.562124	Biso	1.000000	H
H2991	1.0	0.271765	0.624605	0.440055	Biso	1.000000	H
H2992	1.0	0.888487	0.989379	0.591623	Biso	1.000000	H
H2993	1.0	0.845144	0.027693	0.584772	Biso	1.000000	H
H2994	1.0	0.645862	0.672384	0.584024	Biso	1.000000	H
O2639	1.0	0.702227	0.417552	0.215273	Biso	1.000000	O
H2995	1.0	0.711649	0.438540	0.227095	Biso	1.000000	H
H2996	1.0	0.699968	0.424023	0.192465	Biso	1.000000	H
O2640	1.0	0.612436	0.548825	0.458738	Biso	1.000000	O
H2997	1.0	0.596913	0.547884	0.477056	Biso	1.000000	H
H2998	1.0	0.633903	0.537478	0.464817	Biso	1.000000	H
O2641	1.0	0.692224	0.641187	0.370214	Biso	1.000000	O
H2999	1.0	0.712870	0.640395	0.357320	Biso	1.000000	H
H3000	1.0	0.673401	0.649158	0.356648	Biso	1.000000	H
O2642	1.0	0.750965	0.449003	0.595882	Biso	1.000000	O
H3001	1.0	0.727964	0.442886	0.589249	Biso	1.000000	H
H3002	1.0	0.752104	0.475112	0.601887	Biso	1.000000	H
O2643	1.0	0.455449	0.824660	0.979579	Biso	1.000000	O
H3003	1.0	0.456998	0.828263	0.002918	Biso	1.000000	H
H3004	1.0	0.474440	0.836958	0.969025	Biso	1.000000	H
O2644	1.0	0.153036	0.072979	0.967810	Biso	1.000000	O
H3005	1.0	0.148041	0.049588	0.956996	Biso	1.000000	H
H3006	1.0	0.135266	0.078453	0.983460	Biso	1.000000	H
O2645	1.0	0.455698	0.046087	0.253026	Biso	1.000000	O
H3007	1.0	0.453469	0.072878	0.250744	Biso	1.000000	H
H3008	1.0	0.472802	0.040530	0.269322	Biso	1.000000	H
O2646	1.0	0.609343	0.654453	0.079780	Biso	1.000000	O
H3009	1.0	0.600921	0.644012	0.059562	Biso	1.000000	H
H3010	1.0	0.606692	0.681322	0.079393	Biso	1.000000	H
O2647	1.0	0.408952	0.241514	0.597453	Biso	1.000000	O
H3011	1.0	0.403148	0.261226	0.612612	Biso	1.000000	H
H3012	1.0	0.428241	0.249186	0.584367	Biso	1.000000	H
O2648	1.0	0.018268	0.652423	0.990524	Biso	1.000000	O
H3013	1.0	0.040895	0.656250	0.981750	Biso	1.000000	H
H3014	1.0	0.017430	0.661532	0.012726	Biso	1.000000	H
O2649	1.0	0.945375	0.499425	0.061958	Biso	1.000000	O
H3015	1.0	0.966601	0.485967	0.059437	Biso	1.000000	H
H3016	1.0	0.949123	0.521339	0.075295	Biso	1.000000	H
O2650	1.0	0.810095	0.461330	0.970372	Biso	1.000000	O
H3017	1.0	0.788902	0.453411	0.980263	Biso	1.000000	H
H3018	1.0	0.816258	0.444094	0.953179	Biso	1.000000	H
O2651	1.0	0.168013	0.065744	0.221111	Biso	1.000000	O
H3019	1.0	0.184318	0.060590	0.238233	Biso	1.000000	H
H3020	1.0	0.161161	0.042550	0.210923	Biso	1.000000	H
O2652	1.0	0.458096	0.551660	0.428193	Biso	1.000000	O
H3021	1.0	0.477471	0.566218	0.421005	Biso	1.000000	H
H3022	1.0	0.437928	0.559609	0.416517	Biso	1.000000	H
O2653	1.0	0.741331	0.950328	0.365938	Biso	1.000000	O
H3023	1.0	0.719077	0.953602	0.356176	Biso	1.000000	H
H3024	1.0	0.758605	0.951150	0.349111	Biso	1.000000	H
O2654	1.0	0.922005	0.931999	0.150525	Biso	1.000000	O
H3025	1.0	0.916078	0.918166	0.169987	Biso	1.000000	H
H3026	1.0	0.946214	0.937167	0.150537	Biso	1.000000	H
O2655	1.0	0.136465	0.145606	0.372953	Biso	1.000000	O
H3027	1.0	0.132186	0.170633	0.380867	Biso	1.000000	H
H3028	1.0	0.132691	0.127868	0.390386	Biso	1.000000	H
O2656	1.0	0.596701	0.302834	0.720797	Biso	1.000000	O
H3029	1.0	0.594234	0.318333	0.739986	Biso	1.000000	H
H3030	1.0	0.617936	0.308764	0.709973	Biso	1.000000	H
O2657	1.0	0.964455	0.762200	0.905969	Biso	1.000000	O
H3031	1.0	0.955246	0.742258	0.892703	Biso	1.000000	H
H3032	1.0	0.989080	0.760683	0.906049	Biso	1.000000	H
O2658	1.0	0.577446	0.820301	0.616576	Biso	1.000000	O
H3033	1.0	0.598743	0.833331	0.620073	Biso	1.000000	H
H3034	1.0	0.578470	0.795991	0.626843	Biso	1.000000	H
O2659	1.0	0.093688	0.983201	0.748577	Biso	1.000000	O
H3035	1.0	0.078817	0.004557	0.751182	Biso	1.000000	H
H3036	1.0	0.083407	0.961573	0.758750	Biso	1.000000	H
O2660	1.0	0.206532	0.000434	0.912162	Biso	1.000000	O
H3037	1.0	0.217090	0.020866	0.900474	Biso	1.000000	H
H3038	1.0	0.183982	0.007810	0.919222	Biso	1.000000	H
O2661	1.0	0.064233	0.081264	0.148715	Biso	1.000000	O
H3039	1.0	0.067024	0.108117	0.148444	Biso	1.000000	H

H3040	1.0	0.048931	0.073876	0.131370	Biso	1.000000	H
O2662	1.0	0.278764	0.154019	0.874751	Biso	1.000000	O
H3041	1.0	0.267707	0.149955	0.895542	Biso	1.000000	H
H3042	1.0	0.293954	0.133389	0.870147	Biso	1.000000	H
O2663	1.0	0.648876	0.643287	0.799050	Biso	1.000000	O
H3043	1.0	0.648249	0.632287	0.820596	Biso	1.000000	H
H3044	1.0	0.648215	0.623702	0.782800	Biso	1.000000	H
O2664	1.0	0.160841	0.271008	0.956736	Biso	1.000000	O
H3045	1.0	0.183432	0.279206	0.950540	Biso	1.000000	H
H3046	1.0	0.162559	0.248601	0.969828	Biso	1.000000	H
O2665	1.0	0.133668	0.909892	0.673205	Biso	1.000000	O
H3047	1.0	0.142639	0.900413	0.652841	Biso	1.000000	H
H3048	1.0	0.128009	0.889194	0.687380	Biso	1.000000	H
O2666	1.0	0.499547	0.013891	0.295608	Biso	1.000000	O
H3049	1.0	0.490212	0.989335	0.291432	Biso	1.000000	H
H3050	1.0	0.502018	0.017487	0.318875	Biso	1.000000	H
O2667	1.0	0.845173	0.229368	0.351841	Biso	1.000000	O
H3051	1.0	0.848039	0.230621	0.328429	Biso	1.000000	H
H3052	1.0	0.855712	0.206801	0.360027	Biso	1.000000	H
O2668	1.0	0.966840	0.177555	0.454451	Biso	1.000000	O
H3053	1.0	0.974938	0.200392	0.464413	Biso	1.000000	H
H3054	1.0	0.949663	0.183443	0.438316	Biso	1.000000	H
O2669	1.0	0.480764	0.810303	0.891641	Biso	1.000000	O
H3055	1.0	0.493344	0.833199	0.888109	Biso	1.000000	H
H3056	1.0	0.495594	0.791872	0.901469	Biso	1.000000	H
O2670	1.0	0.591272	0.619667	0.027355	Biso	1.000000	O
H3057	1.0	0.580596	0.595614	0.030748	Biso	1.000000	H
H3058	1.0	0.580332	0.632284	0.009303	Biso	1.000000	H
O2671	1.0	0.309502	0.311246	0.279563	Biso	1.000000	O
H3059	1.0	0.293715	0.327974	0.268824	Biso	1.000000	H
H3060	1.0	0.300137	0.303570	0.300337	Biso	1.000000	H
O2672	1.0	0.931720	0.018911	0.843457	Biso	1.000000	O
H3061	1.0	0.942518	0.030754	0.824931	Biso	1.000000	H
H3062	1.0	0.920946	0.037731	0.856895	Biso	1.000000	H
O2673	1.0	0.338550	0.014565	0.997411	Biso	1.000000	O
H3063	1.0	0.331304	0.031171	0.980132	Biso	1.000000	H
H3064	1.0	0.341618	0.989657	0.988731	Biso	1.000000	H
O2674	1.0	0.889888	0.324098	0.810153	Biso	1.000000	O
H3065	1.0	0.907137	0.330806	0.794338	Biso	1.000000	H
H3066	1.0	0.900698	0.313694	0.829318	Biso	1.000000	H
O2675	1.0	0.239780	0.265829	0.857990	Biso	1.000000	O
H3067	1.0	0.260456	0.253775	0.865385	Biso	1.000000	H
H3068	1.0	0.230436	0.252089	0.839740	Biso	1.000000	H
O2676	1.0	0.267069	0.385791	0.337556	Biso	1.000000	O
H3069	1.0	0.291401	0.383720	0.334149	Biso	1.000000	H
H3070	1.0	0.261406	0.411287	0.343212	Biso	1.000000	H
O2677	1.0	0.634408	0.886726	0.452702	Biso	1.000000	O
H3071	1.0	0.622896	0.905757	0.465331	Biso	1.000000	H
H3072	1.0	0.619093	0.877174	0.436194	Biso	1.000000	H
O2678	1.0	0.456668	0.423225	0.313674	Biso	1.000000	O
H3073	1.0	0.440945	0.412830	0.297921	Biso	1.000000	H
H3074	1.0	0.463748	0.403889	0.328707	Biso	1.000000	H
O2679	1.0	0.958006	0.561026	0.096710	Biso	1.000000	O
H3075	1.0	0.943106	0.577194	0.109135	Biso	1.000000	H
H3076	1.0	0.975438	0.575870	0.086188	Biso	1.000000	H
O2680	1.0	0.502788	0.712083	0.072011	Biso	1.000000	O
H3077	1.0	0.512579	0.705053	0.092781	Biso	1.000000	H
H3078	1.0	0.503760	0.738950	0.069589	Biso	1.000000	H
O2681	1.0	0.864818	0.000932	0.883538	Biso	1.000000	O
H3079	1.0	0.881192	0.982203	0.890173	Biso	1.000000	H
H3080	1.0	0.876264	0.024662	0.880765	Biso	1.000000	H
O2682	1.0	0.530662	0.917479	0.625304	Biso	1.000000	O
H3081	1.0	0.546144	0.909914	0.608164	Biso	1.000000	H
H3082	1.0	0.521525	0.941935	0.620349	Biso	1.000000	H
O2683	1.0	0.955323	0.718981	0.020738	Biso	1.000000	O
H3083	1.0	0.978462	0.728303	0.020056	Biso	1.000000	H
H3084	1.0	0.953200	0.700856	0.038123	Biso	1.000000	H
O2684	1.0	0.924235	0.381561	0.537355	Biso	1.000000	O
H3085	1.0	0.929864	0.370590	0.516472	Biso	1.000000	H
H3086	1.0	0.933392	0.406624	0.538467	Biso	1.000000	H
O2685	1.0	0.597944	0.372184	0.518388	Biso	1.000000	O
H3087	1.0	0.612557	0.381617	0.501255	Biso	1.000000	H
H3088	1.0	0.602701	0.385380	0.538471	Biso	1.000000	H
O2686	1.0	0.396388	0.968149	0.420892	Biso	1.000000	O
H3089	1.0	0.373545	0.966666	0.412090	Biso	1.000000	H
H3090	1.0	0.403997	0.943435	0.427079	Biso	1.000000	H
O2687	1.0	0.436826	0.475518	0.790608	Biso	1.000000	O
H3091	1.0	0.461021	0.476226	0.786063	Biso	1.000000	H
H3092	1.0	0.432988	0.463160	0.811269	Biso	1.000000	H
O2688	1.0	0.314901	0.150774	0.954332	Biso	1.000000	O
H3093	1.0	0.292783	0.144913	0.945233	Biso	1.000000	H
H3094	1.0	0.331457	0.152729	0.936927	Biso	1.000000	H

H3095	1.0	0.243809	0.664470	0.486582	Biso	1.000000	H
H3096	1.0	0.332449	0.088621	0.130077	Biso	1.000000	H
H3097	1.0	0.545071	0.017023	0.053910	Biso	1.000000	H
H3098	1.0	0.461603	0.553517	0.576516	Biso	1.000000	H
H3099	1.0	0.293838	0.722527	0.587918	Biso	1.000000	H
H3100	1.0	0.275622	0.997747	0.359947	Biso	1.000000	H
H3101	1.0	0.430522	0.041489	0.957152	Biso	1.000000	H
H3102	1.0	0.288373	0.100983	0.103722	Biso	1.000000	H
H3103	1.0	0.448302	0.160504	0.819766	Biso	1.000000	H
H3104	1.0	0.391423	0.961291	0.665607	Biso	1.000000	H
H3105	1.0	0.564606	0.481248	0.889267	Biso	1.000000	H
H3106	1.0	0.461539	0.371241	0.946552	Biso	1.000000	H
H3107	1.0	0.541065	0.507636	0.928458	Biso	1.000000	H
H3108	1.0	0.562236	0.387956	0.872544	Biso	1.000000	H
H3109	1.0	0.408769	0.321987	0.012352	Biso	1.000000	H
H3110	1.0	0.566890	0.270022	0.903659	Biso	1.000000	H
H3111	1.0	0.330902	0.366260	0.091693	Biso	1.000000	H
H3112	1.0	0.306774	0.010326	0.779918	Biso	1.000000	H
H3113	1.0	0.202937	0.105438	0.746666	Biso	1.000000	H
H3114	1.0	0.197463	0.846410	0.657164	Biso	1.000000	H
H3115	1.0	0.202798	0.528716	0.894097	Biso	1.000000	H
